# Genetic neurodevelopmental clustering and dyslexia

**DOI:** 10.1038/s41380-024-02649-8

**Published:** 2024-07-15

**Authors:** Austeja Ciulkinyte, Hayley S. Mountford, Pierre Fontanillas, 23andMe Research Team, Pierre Fontanillas, Stella Aslibekyan, Adam Auton, Elizabeth Babalola, Robert K. Bell, Jessica Bielenberg, Jonathan Bowes, Katarzyna Bryc, Ninad S. Chaudhary, Daniella Coker, Sayantan Das, Emily DelloRusso, Sarah L. Elson, Nicholas Eriksson, Teresa Filshtein, Will Freyman, Zach Fuller, Chris German, Julie M. Granka, Karl Heilbron, Alejandro Hernandez, Barry Hicks, David A. Hinds, Ethan M. Jewett, Yunxuan Jiang, Katelyn Kukar, Alan Kwong, Yanyu Liang, Keng-Han Lin, Bianca A. Llamas, Matthew H. McIntyre, Steven J. Micheletti, Meghan E. Moreno, Priyanka Nandakumar, Dominique T. Nguyen, Jared O’Connell, Aaron A. Petrakovitz, G. David Poznik, Alexandra Reynoso, Shubham Saini, Morgan Schumacher, Leah Selcer, Anjali J. Shastri, Janie F. Shelton, Jingchunzi Shi, Suyash Shringarpure, Qiaojuan Jane Su, Susana A. Tat, Vinh Tran, Joyce Y. Tung, Xin Wang, Wei Wang, Catherine H. Weldon, Peter Wilton, Corinna D. Wong, Timothy C. Bates, Nicholas G. Martin, Simon E. Fisher, Michelle Luciano

**Affiliations:** 1 Translational Neuroscience PhD Programme, University of Edinburgh, Edinburgh, UK; 2 School of Philosophy, Psychology and Language Sciences, University of Edinburgh, Edinburgh, UK; 3 23andMe, Inc., Sunnyvale, CA USA; 4 Genetic Epidemiology, QIMR Berghofer Medical Research Institute, Herston, QLD Australia; 5 Language and Genetics Department, Max Planck Institute for Psycholinguistics, Nijmegen, Netherlands; 6 Donders Institute for Brain, Cognition and Behaviour, Radboud University, Nijmegen, Netherlands

**Keywords:** Genetics, Psychiatric disorders, Neuroscience, ADHD

## Abstract

Dyslexia is a learning difficulty with neurodevelopmental origins, manifesting as reduced accuracy and speed in reading and spelling. It is substantially heritable and frequently co-occurs with other neurodevelopmental conditions, particularly attention deficit-hyperactivity disorder (ADHD). Here, we investigate the genetic structure underlying dyslexia and a range of psychiatric traits using results from genome-wide association studies of dyslexia, ADHD, autism, anorexia nervosa, anxiety, bipolar disorder, major depressive disorder, obsessive compulsive disorder, schizophrenia, and Tourette syndrome. Genomic Structural Equation Modelling (GenomicSEM) showed heightened support for a model consisting of five correlated latent genomic factors described as: F1) compulsive disorders (including obsessive-compulsive disorder, anorexia nervosa, Tourette syndrome), F2) psychotic disorders (including bipolar disorder, schizophrenia), F3) internalising disorders (including anxiety disorder, major depressive disorder), F4) neurodevelopmental traits (including autism, ADHD), and F5) attention and learning difficulties (including ADHD, dyslexia). ADHD loaded more strongly on the attention and learning difficulties latent factor (F5) than on the neurodevelopmental traits latent factor (F4). The attention and learning difficulties latent factor (F5) was positively correlated with internalising disorders (.40), neurodevelopmental traits (.25) and psychotic disorders (.17) latent factors, and negatively correlated with the compulsive disorders (–.16) latent factor. These factor correlations are mirrored in genetic correlations observed between the attention and learning difficulties latent factor and other cognitive, psychological and wellbeing traits. We further investigated genetic variants underlying both dyslexia and ADHD, which implicated 49 loci (40 not previously found in GWAS of the individual traits) mapping to 174 genes (121 not found in GWAS of individual traits) as potential pleiotropic variants. Our study confirms the increased genetic relation between dyslexia and ADHD versus other psychiatric traits and uncovers novel pleiotropic variants affecting both traits. In future, analyses including additional co-occurring traits such as dyscalculia and dyspraxia will allow a clearer definition of the attention and learning difficulties latent factor, yielding further insights into factor structure and pleiotropic effects.

## Introduction

Dyslexia is classed as a specific learning disorder in the DSM-V [1] and is defined by persistent difficulty with accurate and/or fluent word reading and poor spelling ability [2]. It is present in 5–10% of children worldwide [3, 4], and is the most common specific learning difficulty. While there are no universal diagnostic criteria, dyslexia is typically identified when reading and writing abilities fall below expectations, considering the individual’s age, exposure to effective education and other cognitive abilities [5]. Dyslexia is typically identified in childhood, but persists throughout adulthood [5]. It has been viewed as a neurodevelopmental disorder, linked to structural, connective and functional abnormalities in brain regions involved in visual and auditory processing [6]. Here, we refer to it as a specific learning difficulty in line with the 2009 Rose Report on dyslexia and reading difficulties [2].

Twin studies of dyslexia estimate its heritability at 60–70% [7, 8], which suggests a substantial genetic component. However, the genetic background of dyslexia is complex and multifactorial: individual genes contributing to dyslexia have only a small effect each, and likely act together in an additive manner [9]. Discovery of such genes requires very large sample sizes, thus previous genome-wide association studies (GWAS) have struggled to identify genomic loci predisposing to dyslexia due to low statistical power [10]. Through collaboration with the personal genetics company 23andMe, Inc., Doust and colleagues published the largest dyslexia GWAS to date, comprising over 1.1 million individuals (51,800 dyslexia cases) and discovering 42 significantly associated genomic loci [11]. This dataset enables further study of the genetic background of dyslexia.

Genetic factors underlying neurodevelopmental and psychiatric traits often overlap between disorders: of 208 genes associated with at least one psychiatric disorder, it was reported that approximately half of them are also associated with another disorder [12]. Likewise, evidence from family- and population-based studies points to considerable overlap among genetic influences shared across neurodevelopmental traits [13–16], including dyslexia-ADHD and dyslexia-autism overlaps [17–19]. This may contribute to frequent co-occurrence of dyslexia with other neurodevelopmental differences – in particular, 25–40% of individuals with dyslexia are diagnosed with attention deficit-hyperactivity disorder (ADHD) and vice versa [20, 21]. In contrast, associations between autism (AUT) and dyslexia are complex. Certain traits, such as atypical sensory processing and spatial attention alterations, are shared between autism and dyslexia [17]. Yet, some studies show that autism is linked to better reading skills [22–24], and others suggest dyslexia is no more prevalent among autistic individuals than in the general population [25].

Previous genetic correlation studies have been mostly limited to pairwise comparisons [13]. Recent developments in structural equation modelling methods to study multiple phenotypes with overlapping genomic influences permit a more complex quantitative analysis of genetic correlations between individual psychiatric traits/disorders [26]. Such studies aim to construct a structural model where traits are clustered based on their genetic similarity using the correlational structure between genome-wide single-nucleotide polymorphism (SNP) associations for each trait that give rise to genetic correlations. Each cluster is described by a single latent factor, which represents the shared genetic (SNP) liability within the cluster. In 2019, structural modelling of 8 psychiatric disorders proposed a three-factor model describing three clusters of genetically correlated disorders [27], while a 2022 follow-up analysis of 11 psychiatric disorders (which added problematic alcohol use, post-traumatic stress disorder (PTSD), and anxiety) proposed a four-cluster model [28]. Broadly, these clusters are: 1) Early-onset neurodevelopmental disorders: ADHD, autism, Tourette syndrome, major depressive disorder, plus problematic alcohol use and PTSD in the four-factor model; 2) Disorders with compulsive behaviours: obsessive-compulsive disorder, anorexia nervosa, Tourette syndrome; 3) Mood and psychotic disorders: bipolar disorder, schizophrenia, (and major depressive disorder in three-factor model), and; 4) Internalising disorders (four-factor model): anxiety disorder, major depressive disorder.

To understand whether and where dyslexia is located among these broad genetic clusters, here we expand and adapt current genomic structural models to include dyslexia. Based on the frequent co-occurrence of ADHD and dyslexia [20, 29], correlation between general reading ability and ADHD [29, 30], and significant ADHD polygenic score prediction of dyslexia and reading achievement [10, 31], we expected that dyslexia would fall under an early-onset neurodevelopmental disorder factor. However, we made no prediction of how correlated dyslexia would be with this neurodevelopmental factor given that the nature of the latent factor itself depends on the range of variables included in the analysis. For instance, an earlier GenomicSEM analysis of 8 traits identified a neurodevelopmental factor through loadings from Tourette syndrome, major depressive disorder, ADHD and autism [27]. However, a later study [28] adding problematic alcohol use, PTSD, and anxiety found that problematic alcohol use and PTSD also loaded on the neurodevelopmental factor, while the loading of major depressive disorder on this factor reduced from .60 (in the model with 8 traits) to just .20. In another study [32], addition of alcohol dependence, nicotine dependence and cannabis use disorder to the original 8 traits resulted in a neurodevelopmental factor clustering primarily with major depression, alcohol and nicotine dependence with relatively weak loadings from any of the developmental disorders, thus changing the nature of the factor. Because developmental traits are linked to social outcomes [33–35], this factor may capture variance in causal pathways between childhood traits (e.g., ADHD) and adult outcomes (e.g., alcohol dependence). The aim of the present study was to more clearly delineate neurodevelopmental genomic factors influencing phenotypes arising in childhood, so we excluded substance use dependence and PTSD to avoid a neurodevelopmental factor that would be largely correlated with adult outcomes. In the case of dyslexia for example, a dyslexic child may disengage with school, develop low self-esteem or feel alienated [36], putting them at greater risk of substance use [37] and this causal pathway would be reflected in their genetic covariance. This is an example of vertical pleiotropy, where genetic variants act on a trait downstream of an associated outcome, which we wished to minimise.

In the present study, we focussed on 10 developmental/psychiatric traits and expected to observe a four latent factor model – compulsive, psychotic, neurodevelopmental, and internalising – with dyslexia clustering especially with ADHD for which it is known to be moderately genetically correlated (*r* = 0.53) [11]. Having observed evidence of shared genetic influence on dyslexia and ADHD, we followed up our analysis with targeted investigations of genomic loci associated with both traits, which provided the strongest evidence thus far of pleiotropic effects.

## Methods

### Samples

To construct the genomic structural model, we sourced publicly available GWAS summary statistics for 10 neurodevelopmental/psychiatric traits (Table 1, Supplementary Table 1). GWAS summary statistics for all traits, except dyslexia, were obtained from multi-cohort case-control meta-analyses. The dyslexia summary statistics came from a single analysis of 23andMe, Inc, participants in which genomic inflation was controlled. The combined sample amounted to 453,408 cases and 2,374,026 controls, and included some sample overlap, for example, iPSYCH was part of the ADHD, AUT, and BPD GWAS (Supplementary Table 1). Consistent with previous genomicSEM investigations, data were restricted to participants with European ancestry as these currently have adequate sample sizes.

**Table 1:** Sources and description of GWAS summary statistics used for GenomicSEM modelling.

Disorder	Abbreviation	*N* (cohorts)	*N* (cases)	*N* (controls)
Attention deficit/hyperactivity disorder [31]	ADHD	13	38,691	186,843
Anorexia nervosa [75]	AN	33	16,992	55,525
Anxiety disorder [76]	ANX	2	53,978	221,844
Autism spectrum disorder [77]	AUT	6	18,381	27,969
Bipolar disorder [78]	BIP	57	41,917	371,549
Dyslexia [11]	DYX	1	51,800	1,087,070
Major depressive disorder [79]	MDD	3	170,756	329,443
Obsessive-compulsive disorder [80]	OCD	2	2688	7037
Schizophrenia [81]	SCZ	90	53,386	77,258
Tourette syndrome [82]	TS	4	4819	9488

### Data standardisation and quality control

To ensure all data were uniform and reliable, all GWAS summary data were aligned to the 1000 Genomes European reference genome build 37 [38] and filtered to imputation quality score >0.9, minor allele frequency >0.05 using the *sumstats* and *munge* functions in the GenomicSEM R package [39]. Any SNPs not commonly shared between all 10 studies were excluded. After quality control, 3,959,995 SNPs remained for further analysis.

### Statistical analysis

#### GenomicSEM

SNP-based heritability and pairwise genetic correlations (r_g_) between disorders were obtained using the linkage disequilibrium score regression (LDSC) [40] function in the GenomicSEM package and based on 830,359 high quality HapMap SNPs (Supplementary Table 2). To reveal clusters of traits with shared genetic liability, we synthesised LDSC outputs into a genomic structural model. Our initial model was guided by a previously published three latent factor structural model of 8 psychiatric disorders [27], all of which were also included in the present study. However, fit of this model was poor; and given that anxiety and dyslexia were new to this model, we further investigated the factor structure underlying these genetic relationships using an exploratory factor analysis (EFA) of the genetic covariance matrix with *promax* rotation. Goodness of fit of the confirmatory and exploratory models was evaluated by the standard fit statistics using recommended criteria: lower Akaike’s Information Criterion (AIC), Comparative Fit Index (CFI) in the range of 0.97 and 1 (good; 0.95–0.97, acceptable), and Standardised Root Mean Squared Residual (SRMR) < 0.05 (good; 0.05–0.1, acceptable) [41].

#### Genetic correlations between the attention and learning difficulties latent factor and other traits

Following the identification of the attention and learning difficulties latent factor (F5) through modelling of ADHD and dyslexia, we sought to identify how this factor correlates with other traits. We regressed F5 on all 3,959,995 quality-controlled SNPs using the *userGWAS* function in the GenomicSEM R package. The resulting summary statistics were used to calculate genetic correlations of F5 with 1457 traits using batch LDSC v1.01 within the CTG-VL platform (https://vl.genoma.io/analyses/ldscore). GWAS summary statistics for all traits were obtained via the CTG-VL platform, except for the 10 conditions described in this paper, for which we uploaded the same summary statistics included in the present study for consistency (Table 1, Supplementary Table 3). Correlations were considered significant at a Bonferroni corrected threshold of *p* < 3.383 × 10^−5^ from 1468 tests.

#### Identification of common dyslexia and ADHD variants

Given the frequent co-occurrence of ADHD and dyslexia [20, 29] and the strong genetic overlap we find in this study, we sought to discover pleiotropic genetic loci that are significantly associated with both traits. We filtered dyslexia and ADHD datasets to 3,956,700 shared SNPs and calculated an overall effect size (r) and degree of sharedness (Θ) for each SNP using a polar coordinate transformation method PolarMorphism [42]. By taking Θ into account, we were able to correct for inflation in effect size due to vertical pleiotropy and sample overlap [42]. SNPs where FDR-adjusted *p*-values (*q*-value) for r and Θ were < 0.05 were deemed significant and brought forward for gene mapping. Functional annotation and gene mapping was performed using FUMA v1.5.2 (https://fuma.ctglab.nl) [43]. We clumped linkage-independent genomic regions (r^2^ threshold = 0.4, maximum LD distance = 500 kb, maximum *p*-value for lead SNPs = 5 × 10^−8^, maximum *p*-value cut-off = 5 × 10^−2^). The MHC region was considered as one locus. The 1000 Genomes European population was used as reference [38] (GRCh37 release).

## Results

### Genetic Clustering

**Fig. 1:**
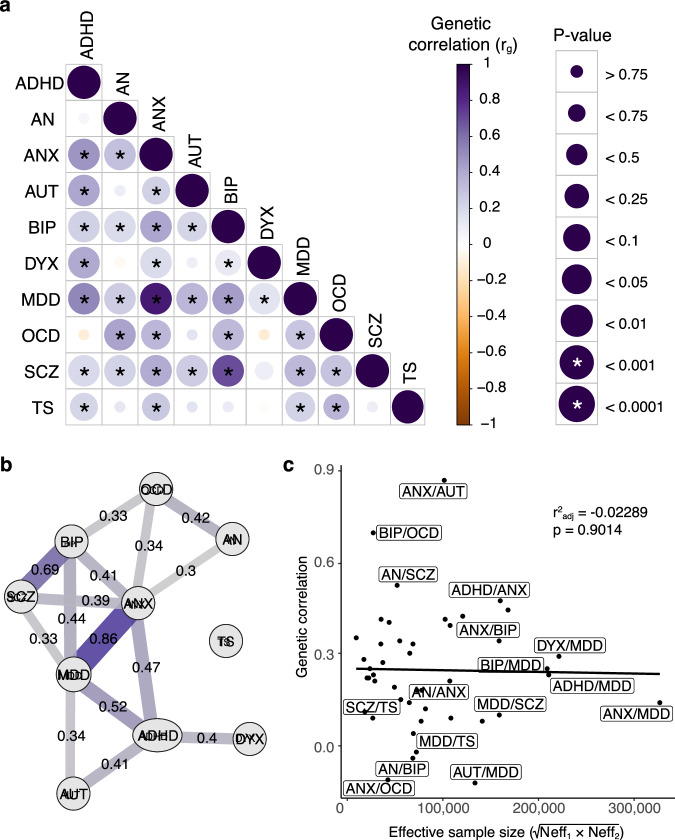
Genetic relationships between ten neurodevelopmental and psychiatric disorders. **a** Pairwise genetic correlations detected using LDSC. Colour intensity scales with correlation coefficient (r_g_), radius of circles scales with significance of *p*-values. Asterisks denote statistically significant (*p* ≤ 0.001) correlations after Bonferroni correction. **b** Path diagram of genetic correlations. Each edge connecting two phenotype nodes represents genetic correlation between those traits. Width and colour intensity of edges scale with correlation coefficient (r_g_). Only pairs where r_g_ > 0.3 and correlation is statistically significant (*p* ≤ 0.05) after Bonferroni correction are displayed. **c** Regression of effective sample size on estimated genetic correlation for each pair of traits. Selected pairs where r_g_ > 0.3, or r_g_ < 0.1, or effective sample size >100,000 are labelled.

Heritability Z-scores were > 4, LDSC intercepts approximately 1, and ratios close to 0, indicating that linkage disequilibrium scores reflected polygenic heritability. The strongest genetic correlations were observed between anxiety (ANX) and major depressive disorder (MDD) (r_g_ = 0.86 ± 0.05), then bipolar disorder (BIP) and schizophrenia (SCZ) (r_g_ = 0.69 ± 0.03), with moderate correlations ranging between 0.40 and 0.45 for pairings of ANX with ADHD and BIP, for MDD with ADHD, BIP and SCZ, for anorexia nervosa (AN) and obsessive-compulsive disorder (OCD), and ADHD and dyslexia (DYX) (see Fig. 1a, b, Supplementary Table 2). A regression of effective sample size on estimated genetic correlation for each pair of disorders indicated that there were no effects of sample size on genomic correlation (R^2^_adj_ = –0.02, *p* = 0.90, Fig. 1c).

**Fig. 2:**
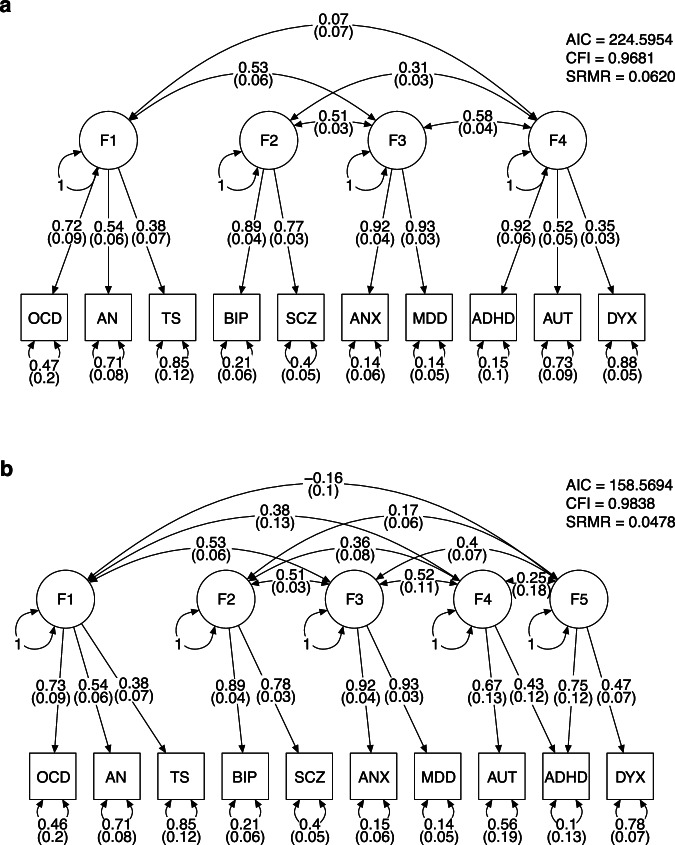
Structural models of 10 neurodevelopmental and psychiatric disorders. Each genetic factor (F1–F5) represents shared genetic liability. Single-headed arrows represent standardised loading parameters, which indicate covariance of the latent factor with a given parameter. Standard errors are given in parentheses. Double-headed arrows connecting factors represent pairwise correlation. Double-headed arrows connecting a component to itself represent residual variance, i.e., variability that is unexplained by factor loading. Factor residuals are fixed for scaling. **a** Confirmatory model based on exploratory factor analysis. **b** Modified confirmatory model that separates DYX and ADHD into a separate cluster of learning difficulties, based on observed genetic correlations.

The initial model was guided by a previously published model, which included 8 of the 10 traits studied here [28]. We expanded it by modelling ANX to load on the same two latent factors as MDD due to their strong correlation. Similarly, we modelled DYX to load on the same factor as ADHD (F1: OCD, AN, TS; F2: BIP, SCZ, MDD, ANX; F3: TS, MDD, ANX, ADHD, AUT, DYX). However, the fit of this model was poor (AIC = 381.40, CFI = 0.933, SRMR = 0.077). An EFA identified a structure of four correlated factors, which together explain 61.3% of underlying variance. The model identified by EFA clusters ANX and MDD under a common factor of internalising disorders (F1: OCD, AN, TS; F2: BIP, SCZ; F3: ANX, MDD; F4: ADHD, AUT, DYX). This is in line with the newer report of an 11-disorder structural model [28]. We thus constructed a confirmatory model using the EFA output (Fig. 2a) and observed a dramatic improvement in model fit (AIC = 224.60, CFI = 0.968, SRMR = 0.062). Having observed a strong genetic correlation between DYX and ADHD but not between DYX and AUT, we modified the confirmatory model by modelling DYX and ADHD to load on a fifth factor (F5) of learning difficulties (Fig. 2b). This model (F1: OCD, AN, TS; F2: BIP, SCZ; F3: ANX, MDD; F4: ADHD, AUT; F5: ADHD, DYX) had the best fit of all the estimated models (AIC = 158.57, CFI = 0.984, SRMR = 0.048).

### Correlations between the attention and learning difficulties latent factor and other traits

**Fig. 3:**
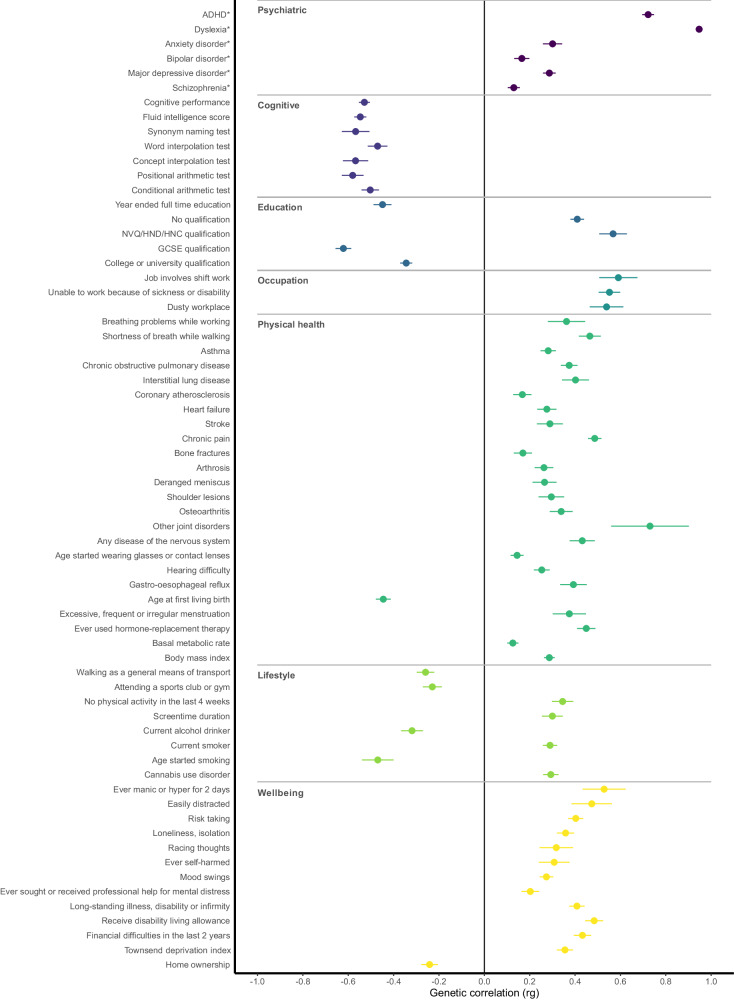
Genomic correlations between the learning difficulties latent factor and other traits. Only 66 out of 330 significant correlations after Bonferroni correction are shown. Traits marked with an asterisk are traits used in this study for GenomicSEM modelling. Error bars represent SEM.

LDSC correlation between the learning difficulties latent factor (F5) and 1457 other traits resulted in 330 significant correlations (Supplementary Table 3). Traits were clustered broadly into “Psychiatric”, “Cognitive”, “Education”, “Occupation”, “Physical health”, “Lifestyle” and “Wellbeing” categories. Of the 330 significantly correlated traits, 66 selected traits are presented in Fig. 3. As expected, the strongest associations of the learning difficulties latent factor were observed with dyslexia (0.95) and ADHD (0.72), and linguistic and mathematical test performance such as concept (–0.57) and word (–0.47) interpolation and positional (–0.58) and conditional (–0.50) arithmetic tests. In addition, we observe moderate correlations with some manifestations of mania or irritability, such as being easily distracted (0.47) or racing thoughts (0.31).

Other notable associations are linked to educational and occupational achievement: we observe strong negative correlations between the learning difficulties latent factor and GCSE qualification (–0.62), year ended full-time education (–0.44) and strong positive correlations with shift working (0.59) or being unable to work at all due to sickness or disability (0.55). Several interesting associations emerge with traits relating to physical health, particularly joint disorders (0.73), chronic pain (0.49) and various pulmonary illnesses; lifestyle – smoking (0.29), cannabis use disorder (0.29), regular physical activity (–0.22); and socioeconomic status, such as financial difficulties (0.43), and home ownership (–0.24).

### Identification of shared variants contributing to dyslexia and ADHD

**Fig. 4:**
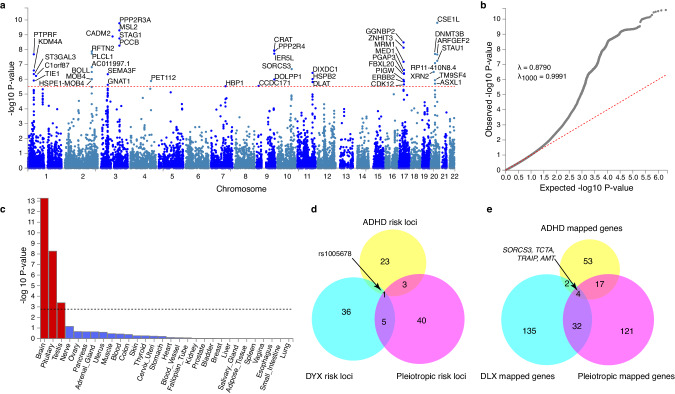
Investigating pleiotropic genomic loci influencing dyslexia and ADHD. **a** Manhattan plot of pleiotropy effect size *p*-values across SNPs shared between dyslexia and ADHD GWAS datasets. Dotted line represents Bonferroni-significant *p*-value (3.081 × 10^–6^). **b** Quantile-quantile plot displaying the observed vs expected statistics under the null hypothesis. **c** MAGMA tissue expression analysis of all SNPs using GTEx v8 dataset with 30 general tissue types. Dotted line represents Bonferroni-significant *p*-value. **d** Venn diagram of significantly associated genomic loci identified in single phenotype GWAS studies and in the pleiotropy analysis. **e** Venn diagram of genes mapped to significantly associated SNPs in single phenotype GWAS studies and in the pleiotropy analysis.

From the overlapping SNPs available for DYX and ADHD, 1 566 SNPs met the significance criteria for overall effect size and degree of sharedness. These 1 566 pre-defined significant SNPs were clumped into 51 lead SNPs belonging to 49 genomic risk loci (Fig. 4a, Supplementary Table 4). Overall and standardised genomic inflation factors were close to 1 (λ = 0.879, λ_1000_ = 0.999), indicating that bulk inflation and excess false positive rates were minimal (Fig. 4b). MAGMA tissue expression analysis indicated that these SNPs are associated with genes showing enriched expression in brain tissues (Fig. 4c). Six out of 49 pleiotropic loci were previously reported as associated with dyslexia [11], and one of these 6 (lead SNP rs1005678 on chromosome 3) was also found in the ADHD GWAS [31]. A further 3 of the pleiotropic loci were reported for ADHD alone [31] (Fig. 4d, Supplementary Table 4). Forty-nine pleiotropic loci were mapped to 174 protein coding genes (Supplementary Table 5). Gene Ontology analysis [44–46] indicated an enrichment in genes involved in protein modification and metabolism, and in development (Table 2, Supplementary Table 6). Thirty-six out of the 174 pleiotropic protein-coding genes have been previously associated with dyslexia (i.e., from 173 genes mapped to the 42 significant loci from the dyslexia GWAS) [11], and 21 with ADHD [31]. Of those, four genes, *TCTA* (T-cell leukaemia translocation altered), *AMT* (aminomethyltransferase), *TRAIP* (TRAF interacting protein) and *SORCS3* (sortilin-related receptor 3) had been associated with both traits in prior literature (Fig. 4e, Supplementary Table 5).

**Table 2:** Results of Gene Ontology enrichment analysis for 174 genes mapped to putative pleiotropic loci influencing dyslexia and ADHD.

Gene Ontology term	Adjusted *p*-value
Molecular functions	
protein binding	2.77 × 10^–4^
hyalurononglucosaminidase activity	2.10 × 10^–2^
peptide-O-fucosyltransferase activity	4.99 × 10^–2^
Biological processes	
glycosaminoglycan catabolic process	1.95 × 10^–3^
macromolecule modification	4.69 × 10^–3^
protein modification process	4.82 × 10^–3^
aminoglycan catabolic process	5.55 × 10^–3^
developmental process	8.02 × 10^–3^
anatomical structure development	2.36 × 10^–2^
multicellular organism development	2.42 × 10^–2^

## Discussion

By extending existing multivariate genomic models of neurodevelopmental and psychiatric traits to include dyslexia, our work has yielded three key findings: (1) that a five latent factor model, composed of internalising (F1), psychotic (F2), compulsive (F3), neurodevelopmental (F4), and attention and learning latent traits (F5), effectively describes the genetic relationships between these diagnoses; (2) that ADHD aligns more with dyslexia and to a learning difficulties latent factor than a neurodevelopmental one, and; (3) we identify a set of pleiotropic genetic loci associated with the presence of both dyslexia and ADHD.

Genetic correlations between the psychiatric disorders were concordant with those of previous analyses [27, 28]. Most pairs of disorders displayed a statistically significant genetic correlation, varying in magnitude, as observed previously. This supports the concept of a complex, interlinked network of shared genetic liabilities across psychiatric disorders.

Based on the frequent co-occurrence of dyslexia and ADHD, and, albeit less often, with autism, we initially hypothesised a structural model that includes dyslexia alongside other neurodevelopmental disorders. The moderate genetic correlation (0.40) between dyslexia and ADHD matches estimates derived from a meta-analysis of twin studies for reading ability indicators and ADHD symptoms [30]. This result was in line with our original hypothesis. However, genetic correlation between dyslexia and autism was found to be statistically non-significant (*p* = 0.082), and accordingly, an improvement in model fit was observed when dyslexia and autism were modelled as loading on distinct latent factors. By contrast with dyslexia, ADHD loaded on both the neurodevelopmental and the attention and learning difficulties factors (F4 and F5), with its correlation being lower on the former than on the latter (0.43 vs 0.75). This suggests that the genetic architecture influencing ADHD shows greater overlap with dyslexia than with autism. We propose four non-mutually exclusive explanations: (a) ADHD is a complex, biologically heterogeneous disorder that shares genetic influences with both neurodevelopmental and learning difficulties to varying degrees; (b) vertical (or spurious) pleiotropy is present, meaning that ADHD has a causal effect on dyslexia or vice-versa; (c) current diagnostic criteria for dyslexia, ADHD and autism are insufficient to categorically define genetically distinct populations, and suggest different degrees of overlap between the continuous traits underlying the diagnoses, and; (d) co-occurrence of dyslexia is higher than autism within the GWAS sample ascertained for ADHD [31] and/or ADHD occurs more frequently than autism in the dyslexic cases included in the 23andMe GWAS.

The shared and unique genetic architecture identified in this study is broadly in line with previous genomic structural models [27, 28] and highlights the complexity of behavioural and psychiatric genetics. We observed strong positive correlations between the five latent factors in our structural model, which supports the concept of general genetic risk factors that contribute to all 10 neurodevelopmental and psychiatric traits in this panel. However, we also observe a range of residual variances, which represent specific genetic factors that confer distinguishable phenotypes to each developmental trait/psychiatric disorder. Dyslexia and autism have larger residual estimates than ADHD (0.78, 0.56 and 0.10, respectively). This suggests that (a) shared genetic factors explain a larger part of total genetic influence for ADHD than for either dyslexia or autism; (b) both dyslexia and autism have a substantial proportion of risk factors not shared with ADHD that pertain more specifically to biological mechanisms related to these traits’ core features (e.g., respective reading subskills and social communication). Additionally, a recent bivariate causal mixture model analysis of bipolar disorder, depression, schizophrenia, and ADHD showed that ADHD is the least polygenic of these traits (5600 causal variants) [47]. If polygenicity is low but pleiotropy high then one would expect lower residual variance for ADHD. Thus, the finding of substantial residual variance in dyslexia and autism could indicate higher polygenicity for these traits compared to ADHD. Intelligence, a trait genetically correlated with dyslexia, ADHD, and autism, for instance, showed high polygenicity (~11,500 variants) using this mixed effect modelling [47]. Such an approach should be applied to dyslexia and autism in future.

We observed that the neurodevelopmental latent factor was more strongly correlated than the attention and learning factor with the other latent factors, often showing correlations that were twice as high. This aligns with findings from a study that dissected the shared and unique genetic background of ADHD and autism spectrum disorder [48]. That study found that the shared genomic portion was strongly associated with psychiatric traits (e.g., depressive symptoms) whereas the distinct part was strongly associated with cognitive traits (e.g., educational attainment, childhood IQ). This distinct part is likely captured in the attention and learning difficulties factor. This is supported by stronger genomic correlations between the learning difficulties factor and cognitive and educational traits: cognitive performance (–0.53), fluid intelligence (–0.55), absence of higher qualifications (0.41); and weaker correlations between the learning difficulties factor and psychiatric traits: major depressive disorder (0.29), anxiety disorder (0.30), self-harming behaviours (0.30) or mood swings (0.27).

Further extension of the genomic structural model including other relevant traits should help to clarify the nature of the attention and learning factor and the large residual variance in dyslexia. Specifically, additional specific learning difficulties, such as dyscalculia, and other developmental disorders that co-occur with dyslexia, namely developmental language disorder and dyspraxia [49] should be prioritised for inclusion. This is particularly evidenced by the strong correlations between the attention and learning difficulties latent factor and performance in linguistic and mathematical tests. At present, no large GWAS have been reported for these traits, precluding their use for GenomicSEM. Multivariate genetic modelling in 12-year-old twins [50] showed that genetic influences on reading, mathematics, and language difficulties each overlapped largely with the genetic influences on general cognitive ability/low ability, so an analysis that further incorporates general cognitive ability may show that it correlates strongly with the attention and learning difficulties factor.

Our targeted approach to identify pleiotropic loci associated with both dyslexia and ADHD uncovered 49 shared genomic loci. Importantly, 43 of these loci were not among genome-wide significant associations of the prior source GWAS of each separate trait, and thus represent newly identified pleiotropic loci. The 49 putative pleiotropic loci were mapped to 174 protein-coding genes, with gene ontology analysis suggesting enrichment of genes involved in development. This is consistent with the neurodevelopmental origins of both dyslexia and ADHD, both manifesting from changes in structure, connectivity and function of the brain [51, 52]. We also detected enrichment of genes involved in protein modification and metabolism. While it is known that post-translational modification has a major role in neurodevelopment in general [53], any specific links to dyslexia and ADHD require further investigation.

Of the 49 significant SNPs, 13 showed no associations in the GWAS Catalogue with primary phenotypes (developmental, cognitive, attainment) related to dyslexia or ADHD. One might place lower confidence in these findings given that variants shared between dyslexia and ADHD would likely have generalised effects that can be detected in related traits such as educational attainment or cognitive function which have well-powered GWAS. Six SNPs were associated with dyslexia in the prior GWAS, with one of these also significant in the previous ADHD GWAS. GWAS Catalogue look-up showed a further two of these SNPs associated with ADHD or a combined phenotype including ADHD, and two others with the related traits of educational attainment and cognitive function (including processing speed). There were no reported associations with relevant traits for rs73175930 in *AUTS2*. However, ADHD is a core feature of individuals with AUTS2 syndrome arising from pathogenic variants in this gene [54]. For the three SNPs previously identified in the ADHD GWAS but not the dyslexia GWAS, all were reported to associate with educational attainment and/or cognitive function. For all other significant SNPs, there was a mixture of reported associations with primary traits, secondary traits (risk taking, externalising behaviours, psychiatric, neuroticism), and other traits (many medical health outcomes that may be downstream outcomes related to lower socio-economic status of those with dyslexia and ADHD). These variants may therefore be associated with a whole range of behaviours due to their primary effect on attention and learning processes which we suggest define the covariation between dyslexia and ADHD. This hypothesis might be further tested in extended genomicSEM models that include a host of variables that we identify here (e.g., risk-taking, externalising behaviours, educational attainment) as being previously associated with our significant SNPs for combined dyslexia and ADHD. Downstream outcomes can be confirmed by Mendelian Randomization methods in cases where confounding by socio-economic status is unproblematic.

Four genes – SORCS3, TCTA, TRAIP and AMT – shown to be pleiotropic had previously been associated with dyslexia and ADHD in individual GWAS studies and are very strong pleiotropic candidate genes [11, 31]. The *SORCS3* protein is abundant in the central nervous system [52]. It has a primary role in sorting intracellular proteins between organelles and the plasma membrane, and a secondary role in cell signalling. Mouse studies of the murine orthologue of *SORCS3* have implicated it in long-term synaptic depression via aberrant glutamate signalling [55]. *SORCS3*-deficient mice have decreased synaptic plasticity and deficits in spatial learning and memory. This is consistent with the proposed theories of reduced visual and spatial learning abilities in children with dyslexia and ADHD [56, 57]. *SORCS3* has previously been suggested as a pleiotropic gene associated with ADHD, autism, schizophrenia, bipolar, and MDD [58]. In addition, *SORCS3* mutations have been linked with intellectual delay [59], multiple sclerosis [60] and Alzheimer’s disease [61]. *TCTA* has a role in regulating processes related to dissolution and absorption of bone [62], so is not an obvious candidate gene for involvement in brain-related phenotypes. However, in GWAS, variants at this locus have been associated with relevant traits of very high intelligence, cognitive function, and household income [63–65], and less relevant traits like cardiovascular disease, Crohn’s disease, inflammatory bowel disease (from NHGRI-EBI GWAS Catalogue [66]). *TRAIP*, part of the RING finger protein gene group, is linked to the ubiquitination pathway protecting genome integrity following replication stress [67]. Variants in this gene have been associated with more than 40 phenotypes, but notably the most strongly associated SNPs (*P* ≤ 7 × 10^−18^) in this gene have been from a meta-analysis combining ADHD, AUT and intelligence [68] for multiple studies of intelligence/cognitive function including the cognitive component of education attainment [69–72], and externalising behaviour [73]. Thus, the gene may potentially be involved in general learning processes that can be affected in both ADHD and dyslexia. The *AMT* gene encodes a critical component of the glycine cleavage system contributing to normal development and function of neurons [74]. It is strongly associated (*P* = 5 × 10^−82^) with educational attainment [71], an outcome that is negatively correlated with both ADHD and dyslexia, and could be prioritised as a candidate gene involved in learning given its lack of strong association with other phenotypes. For the remaining 170 pleiotropic mapped genes (32 previously found for dyslexia and 17 for ADHD), and particularly the 121 that were not identified in previous dyslexia and ADHD screens, future research is needed to understand the extent of their overlap with both general and specific cognitive abilities.

There are a number of limitations to this study: (1) the GWAS included here had variable sample sizes. Lack of power in small GWAS datasets (i.e., AN, TS) results in decreased effect sizes, and thus could contribute to reduced strengths of genetic correlations observed. (2) We have been unable to control for causal relationships and diagnostic overlap in building our structural models, which may potentially inflate genetic correlations. (3) The dyslexia, ADHD, ANX and MDD GWAS was based on self-reported diagnoses, which may have confounded results through misclassification of cases versus controls. (4) All participants of the GWAS included in this study were adult individuals of European ancestry, limiting the applicability of results across ancestries and across life stages. Future investigations into psychiatric conditions in non-European samples or at earlier neurodevelopmental stages will likely uncover different genomic correlations, allowing for a more comprehensive understanding of the relationships between them.

In sum, our analysis of genetic relationships of 10 developmental traits, which includes dyslexia for the first time, and psychiatric disorders has shown the emergence of an attention and learning difficulties factor that is only modestly correlated with a separate neurodevelopmental factor. In this model, ADHD aligns more closely with dyslexia than autism, suggesting that ADHD may be better termed as a learning difficulty than a psychiatric disorder, and highlighting the importance of it being managed within education and later employment. To explain the large residual variance in dyslexia, extension of the genomicSEM model to include other co-occurring developmental traits and a range of other cognitive abilities will be informative, once reliable GWAS of these are available. Finally, we discovered 49 potentially pleiotropic genomic risk loci, 43 of which are novel, influencing the development of both dyslexia and ADHD, and further confirm *SORCS3* and *TRAIP* as putative pleiotropic genes that likely have broad associations with neuropsychiatric traits potentially through learning pathways. Future GWAS investigations of individuals with co-occurring dyslexia and ADHD will help to validate our pleiotropy analyses and determine whether the identified variant effects are larger when both traits are present.

## Supplementary information

Supplementary Table 1

Supplementary Table 2

Supplementary Table 3

Supplementary Table 4

Supplementary Table 5

Supplementary Table 6

## Data Availability

Supplementary information is available at *Molecular Psychiatry’s* website. The full dyslexia GWAS summary statistics for the 23andMe discovery dataset will be made available through 23andMe to qualified researchers under an agreement with 23andMe that protects the privacy of the 23andMe participants. Datasets will be made available at no cost for academic use. Please visit https://research.23andme.com/collaborate/#dataset-access/ for more information and to apply to access the data. Note that 23andMe participants provided informed consent and volunteered to participate in the research online, under a protocol approved by the external AAHRPP-accredited IRB, Ethical & Independent (E&I) Review Services. As of 2022, E&I Review Services is part of Salus IRB (https://www.versiticlinicaltrials.org/salusirb).

## Code availability

All original code used to conduct the analyses in this paper is available at https://github.com/AustejaCiulkinyte/DyslexiaSEM.
