# Supplementary material for: Genetic neurodevelopmental clustering and dyslexia
Source: Mol Psychiatry. 2024 Jul 15;30(1):140–50. doi: 10.1038/s41380-024-02649-8 (PMC11649571; doi:10.1038/s41380-024-02649-8)
Supplement: Supplementary file 1 — Supplementary Table 1 [file 41380_2024_2649_MOESM1_ESM.pdf]

Supplementary Table 1. Sources and description of GWAS summary statistics used in this analysis.

Disorder	Abbreviation	N(cohorts)	N(cases)	N(controls)	N(total)	N(eff)	Reference
Attention deficit/hyperactivity disorder	ADHD	12	38,691	186,843	225,534	**103136**	DOI: 10.1038/s41588-022-01285-8
Anorexia nervosa	AN	33	16,992	55,525	72,517	**46322**	DOI: 10.1038/s41588-019-0439-2
Anxiety disorder*	ANX	2	53,978	221,844	275,822	**248239**	DOI: 10.1101/2022.04.12.22273763
Autism spectrum disorder	AUT	6	18,381	27,969	46,350	**41457**	DOI: 10.1038/s41588-019-0344-8
Bipolar disorder	BIP	57	41,917	371,549	413,466	**101574**	DOI: 10.1038/s41588-021-00857-4
Dyslexia	DYX	1	51,800	1,087,070	1,138,870	**113887**	DOI: 10.1038/s41588-022-01192-y
Major depressive disorder	MDD	3	170,756	329,443	500,199	**430424**	DOI: 10.1038/s41593-018-0326-7
Obsessive-compulsive disorder	OCD	2	2,688	7,037	9,725	**7281**	DOI: 10.1038/mp.2017.154
Schizophrenia	SCZ	90	53,386	77,258	130,644	**58746**	DOI: 10.1038/s41586-022-04434-5
Tourette syndrome	TS	4	4,819	9,488	14,307	**12140**	DOI: 10.1176/appi.ajp.2018.18070857
**Total**			453,408	2,374,026			

*These sumstats are a meta-analysis of two anxiety GWAS studies published previously:

DOI: 10.1038/s41380-019-0559-1 DOI: 10.1176/appi.ajp.2019.19030256
