# Supplementary material for: Genetic neurodevelopmental clustering and dyslexia
Source: Mol Psychiatry. 2024 Jul 15;30(1):140–50. doi: 10.1038/s41380-024-02649-8 (PMC11649571; doi:10.1038/s41380-024-02649-8)
Supplement: Supplementary file 2 — Supplementary Table 2 [file 41380_2024_2649_MOESM2_ESM.pdf]

Supplementary Table 2. SNP heritabilities, pairwise genetic correlations, standard error of correlations, p-values of correlations and significance after Bonferroni correction for 10 psychiatric disorders.

SNP heritability in liability scale (SE)	
ADHD	0.1733 (0.0104)
AN	0.1552 (0.0119)
ANX	0.0507 (0.0036)
ASD	0.1287 (0.0115)
BIP	0.1844 (0.0087)
DYX	0.1666 (0.0083)
MDD	0.1068 (0.0056)
OCD	0.3228 (0.0549)
SCZ	0.4494 (0.0189)
TS	0.2319 (0.0287)

Correlation (SE)	ADHD	AN	ANX	ASD	BIP	DYX	MDD	OCD	SCZ	TS
ADHD	1.00E+00									
AN	0.0424 (0.0414)	1.00E+00								
ANX	0.4676 (0.0442)	0.3 (0.0475)	1.00E+00							
ASD	0.4087 (0.0572)	0.0891 (0.0577)	0.2308 (0.0561)	1.00E+00						
BIP	0.2344 (0.0338)	0.1809 (0.0369)	0.4068 (0.0423)	0.2061 (0.0463)	1.00E+00					
DYX	0.3997 (0.0343)	-0.0441 (0.0357)	0.1828 (0.0378)	0.0765 (0.044)	0.114 (0.0319)	1.00E+00				
MDD	0.5195 (0.0361)	0.2547 (0.0373)	0.8574 (0.0459)	0.3398 (0.0391)	0.441 (0.0353)	0.1437 (0.0276)	1.00E+00			
OCD	-0.1131 (0.0698)	0.4187 (0.0782)	0.3409 (0.0783)	0.1413 (0.0934)	0.3308 (0.0622)	-0.1248 (0.0584)	0.2822 (0.0595)	1.00E+00		
SCZ	0.19 (0.0292)	0.2231 (0.0365)	0.3948 (0.042)	0.2453 (0.0408)	0.6878 (0.0333)	0.0792 (0.0281)	0.3282 (0.0329)	0.2948 (0.0617)	1.00E+00	
TS	0.2051 (0.0544)	0.1209 (0.0695)	0.2666 (0.0706)	0.1497 (0.0654)	0.1026 (0.0506)	-0.0184 (0.0498)	0.2156 (0.0466)	0.351 (0.1029)	0.0946 (0.0438)	1.00E+00

Correlation	ADHD	AN	ANX	ASD	BIP	DYX	MDD	OCD	SCZ	TS
ADHD	1.00									
AN	0.04	1.00								
ANX	0.47	0.30	1.00							
ASD	0.41	0.09	0.23	1.00						
BIP	0.23	0.18	0.41	0.21	1.00					
DYX	0.40	-0.04	0.18	0.08	0.11	1.00				
MDD	0.52	0.25	0.86	0.34	0.44	0.14	1.00			
OCD	-0.11	0.42	0.34	0.14	0.33	-0.12	0.28	1.00		
SCZ	0.19	0.22	0.39	0.25	0.69	0.08	0.33	0.29	1.00	
TS	0.21	0.12	0.27	0.15	0.10	-0.02	0.22	0.35	0.09	1.00

SE of correlation	ADHD	AN	ANX	ASD	BIP	DYX	MDD	OCD	SCZ	TS
ADHD										
AN	0.04									
ANX	0.04	0.05								
ASD	0.06	0.06	0.06							
BIP	0.03	0.04	0.04	0.05						
DYX	0.03	0.04	0.04	0.04	0.03					
MDD	0.04	0.04	0.05	0.04	0.04	0.03				
OCD	0.07	0.08	0.08	0.09	0.06	0.06	0.06			
SCZ	0.03	0.04	0.04	0.04	0.03	0.03	0.03	0.06		
TS	0.05	0.07	0.07	0.07	0.05	0.05	0.05	0.10	0.04	

P-value	ADHD	AN	ANX	ASD	BIP	DYX	MDD	OCD	SCZ	TS
ADHD	na									
AN	3.05E-01	na								
ANX	4.02E-26	2.63E-10	na							
ASD	8.87E-13	1.23E-01	3.86E-05	na						
BIP	4.10E-12	9.17E-07	7.35E-22	8.73E-06	na					
DYX	2.26E-31	2.16E-01	1.35E-06	8.20E-02	3.54E-04	na				
MDD	5.87E-47	8.67E-12	6.10E-78	3.56E-18	9.98E-36	1.85E-07	na			
OCD	1.05E-01	8.46E-08	1.35E-05	1.30E-01	1.06E-07	3.27E-02	2.13E-06	na		
SCZ	7.77E-11	1.00E-09	5.80E-21	1.80E-09	9.14E-95	4.74E-03	2.06E-23	1.76E-06	na	
TS	1.66E-04	8.18E-02	1.60E-04	2.21E-02	4.27E-02	7.13E-01	3.78E-06	6.50E-04	3.09E-02	na

Bonferroni corrections:
55 tests have been made.

Original pval:	Corrected pval:	
0.05	9.09E-04	*
0.01	1.82E-04	**
0.001	1.82E-05	***

P-value significance	ADHD	AN	ANX	ASD	BIP	DYX	MDD	OCD	SCZ	TS
ADHD	na									
AN	n.s.	na								
ANX	***	***	na							
ASD	***	n.s.	**	na						
BIP	***	***	***	***	na					
DYX	***	n.s.	***	n.s.	*	na				
MDD	***	***	***	***	***	***	na			
OCD	n.s.	***	***	n.s.	***	n.s.	***	na		
SCZ	***	***	***	***	***	n.s.	***	***	na	
TS	**	n.s.	**	n.s.	n.s.	n.s.	***	*	n.s.	na
