# Supplementary material for: Genetic neurodevelopmental clustering and dyslexia
Source: Mol Psychiatry. 2024 Jul 15;30(1):140–50. doi: 10.1038/s41380-024-02649-8 (PMC11649571; doi:10.1038/s41380-024-02649-8)
Supplement: Supplementary file 3 — Supplementary Table 3 [file 41380_2024_2649_MOESM3_ESM.pdf]

Supplementary Table 3. Genetic correlations between the attention and learning difficulties latent factor and 1468 other traits using the CTG-VL database. Phenotypes where rg, se, z and p values returned NA had been removed.

<table>
<tr><th>p2</th><th>p1</th><th>rg</th><th>se</th><th>z</th><th>p</th><th>h2_obs</th><th>h2_obs_se</th><th>h2_int</th><th>h2_int_se</th><th>gcov_int</th><th>gcov_int_se</th><th>phenotype</th><th>plotted</th><th>category</th><th>category2</th><th>renamed</th><th>N</th><th>NCases</th><th>NControls</th><th>Cohort</th><th>URL</th><th>Notes</th></tr>
<tr><td>100001_raw.txt</td><td>1710174270056F5forCTG.txt.gz</td><td>0.01367</td><td>0.06238</td><td>0.2192</td><td>0.8265</td><td>0.06527</td><td>0.01237</td><td>1.004</td><td>0.009109</td><td>0.0006313</td><td>0.008115</td><td>Food weight</td><td>FALSE</td><td></td><td></td><td></td><td>51453</td><td></td><td></td><td>UK Biobank</td><td>https://docs.google.com/spreadsheets/d/1kvPoupSzsSFBNSztMzl04xMoSC3Kcx3CrjVf4yBmESU/edit?ts=5b5f17db#gid=227859291</td><td>PHESANT Transformation:100001_0|| CONTINUOUS MAIN || CONTINUOUS || ||-Notes:Total food weight. Estimated intake, based on food and beverage consumption yesterday, excluding any supplements.-Variable type:continuous_raw</td></tr>
<tr><td>100002_raw.txt</td><td>1710174270056F5forCTG.txt.gz</td><td>-0.1628</td><td>0.0825</td><td>-1.973</td><td>0.04848</td><td>0.03565</td><td>0.01149</td><td>1.005</td><td>0.009679</td><td>0.0003251</td><td>0.007406</td><td>Energy</td><td>FALSE</td><td></td><td></td><td></td><td>51453</td><td></td><td></td><td>UK Biobank</td><td>https://docs.google.com/spreadsheets/d/1kvPoupSzsSFBNSztMzl04xMoSC3Kcx3CrjVf4yBmESU/edit?ts=5b5f17db#gid=227859291</td><td>PHESANT Transformation:100002_0|| CONTINUOUS MAIN || CONTINUOUS || ||-Notes:Total energy. Estimated intake, based on food and beverage consumption yesterday, excluding any supplements.-Variable type:continuous_raw</td></tr>
<tr><td>100003_raw.txt</td><td>1710174270056F5forCTG.txt.gz</td><td>-0.08155</td><td>0.09196</td><td>-0.8868</td><td>0.3752</td><td>0.02539</td><td>0.01199</td><td>1.006</td><td>0.01056</td><td>-0.00598</td><td>0.00783</td><td>Protein</td><td>FALSE</td><td></td><td></td><td></td><td>51453</td><td></td><td></td><td>UK Biobank</td><td>https://docs.google.com/spreadsheets/d/1kvPoupSzsSFBNSztMzl04xMoSC3Kcx3CrjVf4yBmESU/edit?ts=5b5f17db#gid=227859291</td><td>PHESANT Transformation:100003_0|| CONTINUOUS MAIN || CONTINUOUS || ||-Notes:Protein. Estimated intake, based on food and beverage consumption yesterday, excluding any supplements.-Variable type:continuous_raw</td></tr>
<tr><td>100004_raw.txt</td><td>1710174270056F5forCTG.txt.gz</td><td>-0.217</td><td>0.08244</td><td>-2.632</td><td>0.008497</td><td>0.0391</td><td>0.01159</td><td>0.995</td><td>0.0103</td><td>0.008714</td><td>0.007468</td><td>Fat</td><td>FALSE</td><td></td><td></td><td></td><td>51453</td><td></td><td></td><td>UK Biobank</td><td>https://docs.google.com/spreadsheets/d/1kvPoupSzsSFBNSztMzl04xMoSC3Kcx3CrjVf4yBmESU/edit?ts=5b5f17db#gid=227859291</td><td>PHESANT Transformation:100004_0|| CONTINUOUS MAIN || CONTINUOUS || ||-Notes:Total fat. Estimated intake, based on food and beverage consumption yesterday, excluding any supplements.-Variable type:continuous_raw</td></tr>
<tr><td>100005_raw.txt</td><td>1710174270056F5forCTG.txt.gz</td><td>-0.06231</td><td>0.09262</td><td>-0.6727</td><td>0.5011</td><td>0.02716</td><td>0.01114</td><td>1.01</td><td>0.008913</td><td>-0.008034</td><td>0.007848</td><td>Carbohydrate</td><td>FALSE</td><td></td><td></td><td></td><td>51453</td><td></td><td></td><td>UK Biobank</td><td>https://docs.google.com/spreadsheets/d/1kvPoupSzsSFBNSztMzl04xMoSC3Kcx3CrjVf4yBmESU/edit?ts=5b5f17db#gid=227859291</td><td>PHESANT Transformation:100005_0|| CONTINUOUS MAIN || CONTINUOUS || ||-Notes:Carbohydrate. Estimated intake, based on food and beverage consumption yesterday, excluding any supplements.-Variable type:continuous_raw</td></tr>
<tr><td>100006_raw.txt</td><td>1710174270056F5forCTG.txt.gz</td><td>-0.08105</td><td>0.08458</td><td>-0.9583</td><td>0.3379</td><td>0.0331</td><td>0.01085</td><td>0.998</td><td>0.009235</td><td>-0.001166</td><td>0.007591</td><td>Saturated fat</td><td>FALSE</td><td></td><td></td><td></td><td>51453</td><td></td><td></td><td>UK Biobank</td><td>https://docs.google.com/spreadsheets/d/1kvPoupSzsSFBNSztMzl04xMoSC3Kcx3CrjVf4yBmESU/edit?ts=5b5f17db#gid=227859291</td><td>PHESANT Transformation:100006_0|| CONTINUOUS MAIN || CONTINUOUS || ||-Notes:Saturated fat. Estimated intake, based on food and beverage consumption yesterday, excluding any supplements.-Variable type:continuous_raw</td></tr>
<tr><td>100007_raw.txt</td><td>1710174270056F5forCTG.txt.gz</td><td>-0.3837</td><td>0.1763</td><td>-2.177</td><td>0.02949</td><td>0.01666</td><td>0.01147</td><td>1.007</td><td>0.01001</td><td>0.01597</td><td>0.00751</td><td>Polyunsaturated fat</td><td>FALSE</td><td></td><td></td><td></td><td>51453</td><td></td><td></td><td>UK Biobank</td><td>https://docs.google.com/spreadsheets/d/1kvPoupSzsSFBNSztMzl04xMoSC3Kcx3CrjVf4yBmESU/edit?ts=5b5f17db#gid=227859291</td><td>PHESANT Transformation:100007_0|| CONTINUOUS MAIN || CONTINUOUS || ||-Notes:Polyunsaturated fat. Estimated intake, based on food and beverage consumption yesterday, excluding any supplements.-Variable type:continuous_raw</td></tr>
<tr><td>100008_raw.txt</td><td>1710174270056F5forCTG.txt.gz</td><td>0.06074</td><td>0.08009</td><td>0.7584</td><td>0.4482</td><td>0.03983</td><td>0.01161</td><td>1.009</td><td>0.009839</td><td>-0.0152</td><td>0.00867</td><td>Total sugars</td><td>FALSE</td><td></td><td></td><td></td><td>51453</td><td></td><td></td><td>UK Biobank</td><td>https://docs.google.com/spreadsheets/d/1kvPoupSzsSFBNSztMzl04xMoSC3Kcx3CrjVf4yBmESU/edit?ts=5b5f17db#gid=227859291</td><td>PHESANT Transformation:100008_0|| CONTINUOUS MAIN || CONTINUOUS || ||-Notes:Total sugars. Estimated intake, based on food and beverage consumption yesterday, excluding any supplements.-Variable type:continuous_raw</td></tr>
<tr><td>100009_raw.txt</td><td>1710174270056F5forCTG.txt.gz</td><td>-0.0614</td><td>0.08347</td><td>-0.7356</td><td>0.462</td><td>0.04306</td><td>0.01201</td><td>1.007</td><td>0.01006</td><td>0.0003731</td><td>0.008313</td><td>Englyst dietary fibre</td><td>FALSE</td><td></td><td></td><td></td><td>51453</td><td></td><td></td><td>UK Biobank</td><td>https://docs.google.com/spreadsheets/d/1kvPoupSzsSFBNSztMzl04xMoSC3Kcx3CrjVf4yBmESU/edit?ts=5b5f17db#gid=227859291</td><td>PHESANT Transformation:100009_0|| CONTINUOUS MAIN || CONTINUOUS || ||-Notes:Englyst dietary fibre. Estimated intake, based on food and beverage consumption yesterday, excluding any supplements.-Variable type:continuous_raw</td></tr>
<tr><td>100011_raw.txt</td><td>1710174270056F5forCTG.txt.gz</td><td>-0.2562</td><td>0.08931</td><td>-2.868</td><td>0.004128</td><td>0.03742</td><td>0.01172</td><td>1.011</td><td>0.01046</td><td>0.002753</td><td>0.007259</td><td>Iron</td><td>FALSE</td><td></td><td></td><td></td><td>51453</td><td></td><td></td><td>UK Biobank</td><td>https://docs.google.com/spreadsheets/d/1kvPoupSzsSFBNSztMzl04xMoSC3Kcx3CrjVf4yBmESU/edit?ts=5b5f17db#gid=227859291</td><td>PHESANT Transformation:100011_0|| CONTINUOUS MAIN || CONTINUOUS || ||-Notes:Iron. Estimated intake, based on food and beverage consumption yesterday, excluding any supplements.-Variable type:continuous_raw</td></tr>
<tr><td>100012_raw.txt</td><td>1710174270056F5forCTG.txt.gz</td><td>-0.08908</td><td>0.09522</td><td>-0.9355</td><td>0.3495</td><td>0.03137</td><td>0.01108</td><td>1.007</td><td>0.009566</td><td>-0.005312</td><td>0.008742</td><td>Vitamin B6</td><td>FALSE</td><td></td><td></td><td></td><td>51453</td><td></td><td></td><td>UK Biobank</td><td>https://docs.google.com/spreadsheets/d/1kvPoupSzsSFBNSztMzl04xMoSC3Kcx3CrjVf4yBmESU/edit?ts=5b5f17db#gid=227859291</td><td>PHESANT Transformation:100012_0|| CONTINUOUS MAIN || CONTINUOUS || ||-Notes:Vitamin B6. Estimated intake, based on food and beverage consumption yesterday, excluding any supplements.-Variable type:continuous_raw</td></tr>
<tr><td>100013_raw.txt</td><td>1710174270056F5forCTG.txt.gz</td><td>-0.1427</td><td>0.1658</td><td>-0.8607</td><td>0.3894</td><td>0.009897</td><td>0.01048</td><td>1</td><td>0.00877</td><td>-0.003935</td><td>0.007292</td><td>Vitamin B12</td><td>FALSE</td><td></td><td></td><td></td><td>51453</td><td></td><td></td><td>UK Biobank</td><td>https://docs.google.com/spreadsheets/d/1kvPoupSzsSFBNSztMzl04xMoSC3Kcx3CrjVf4yBmESU/edit?ts=5b5f17db#gid=227859291</td><td>PHESANT Transformation:100013_0|| CONTINUOUS MAIN || CONTINUOUS || ||-Notes:Vitamin B12. Estimated intake, based on food and beverage consumption yesterday, excluding any supplements.-Variable type:continuous_raw</td></tr>
<tr><td>100014_raw.txt</td><td>1710174270056F5forCTG.txt.gz</td><td>-0.197</td><td>0.08925</td><td>-2.207</td><td>0.02728</td><td>0.03728</td><td>0.01117</td><td>1.005</td><td>0.009588</td><td>0.00578</td><td>0.007688</td><td>Folate</td><td>FALSE</td><td></td><td></td><td></td><td>51453</td><td></td><td></td><td>UK Biobank</td><td>https://docs.google.com/spreadsheets/d/1kvPoupSzsSFBNSztMzl04xMoSC3Kcx3CrjVf4yBmESU/edit?ts=5b5f17db#gid=227859291</td><td>PHESANT Transformation:100014_0|| CONTINUOUS MAIN || CONTINUOUS || ||-Notes:Folate. Estimated intake, based on food and beverage consumption yesterday, excluding any supplements.-Variable type:continuous_raw</td></tr>
<tr><td>100015_raw.txt</td><td>1710174270056F5forCTG.txt.gz</td><td>-0.08945</td><td>0.09388</td><td>-0.9529</td><td>0.3407</td><td>0.03548</td><td>0.01004</td><td>1.004</td><td>0.009262</td><td>-0.004755</td><td>0.008667</td><td>Vitamin C</td><td>FALSE</td><td></td><td></td><td></td><td>51453</td><td></td><td></td><td>UK Biobank</td><td>https://docs.google.com/spreadsheets/d/1kvPoupSzsSFBNSztMzl04xMoSC3Kcx3CrjVf4yBmESU/edit?ts=5b5f17db#gid=227859291</td><td>PHESANT Transformation:100015_0|| CONTINUOUS MAIN || CONTINUOUS || ||-Notes:Vitamin C. Estimated intake, based on food and beverage consumption yesterday, excluding any supplements.-Variable type:continuous_raw</td></tr>
<tr><td>100016_raw.txt</td><td>1710174270056F5forCTG.txt.gz</td><td>-0.006895</td><td>0.08653</td><td>-0.07968</td><td>0.9365</td><td>0.03524</td><td>0.0121</td><td>1.007</td><td>0.01014</td><td>-0.01185</td><td>0.008254</td><td>Potassium</td><td>FALSE</td><td></td><td></td><td></td><td>51453</td><td></td><td></td><td>UK Biobank</td><td>https://docs.google.com/spreadsheets/d/1kvPoupSzsSFBNSztMzl04xMoSC3Kcx3CrjVf4yBmESU/edit?ts=5b5f17db#gid=227859291</td><td>PHESANT Transformation:100016_0|| CONTINUOUS MAIN || CONTINUOUS || ||-Notes:Potassium. Estimated intake, based on food and beverage consumption yesterday, excluding any supplements.-Variable type:continuous_raw</td></tr>
<tr><td>100017_raw.txt</td><td>1710174270056F5forCTG.txt.gz</td><td>-0.129</td><td>0.08416</td><td>-1.533</td><td>0.1253</td><td>0.038</td><td>0.01188</td><td>1.01</td><td>0.009722</td><td>-0.005435</td><td>0.007483</td><td>Magnesium</td><td>FALSE</td><td></td><td></td><td></td><td>51453</td><td></td><td></td><td>UK Biobank</td><td>https://docs.google.com/spreadsheets/d/1kvPoupSzsSFBNSztMzl04xMoSC3Kcx3CrjVf4yBmESU/edit?ts=5b5f17db#gid=227859291</td><td>PHESANT Transformation:100017_0|| CONTINUOUS MAIN || CONTINUOUS || ||-Notes:Magnesium. Estimated intake, based on food and beverage consumption yesterday, excluding any supplements.-Variable type:continuous_raw</td></tr>
<tr><td>100018_raw.txt</td><td>1710174270056F5forCTG.txt.gz</td><td>-0.269</td><td>0.1292</td><td>-2.081</td><td>0.03743</td><td>0.02246</td><td>0.01163</td><td>1.011</td><td>0.009793</td><td>0.006438</td><td>0.00804</td><td>Retinol</td><td>FALSE</td><td></td><td></td><td></td><td>49890</td><td></td><td></td><td>UK Biobank</td><td>https://docs.google.com/spreadsheets/d/1kvPoupSzsSFBNSztMzl04xMoSC3Kcx3CrjVf4yBmESU/edit?ts=5b5f17db#gid=227859291</td><td>PHESANT Transformation:100018_0|| CONTINUOUS MAIN || CONTINUOUS || ||-Notes:Retinol. Estimated intake, based on food and beverage consumption yesterday, excluding any supplements.-Variable type:continuous_raw</td></tr>
<tr><td>100021_raw.txt</td><td>1710174270056F5forCTG.txt.gz</td><td>-0.1394</td><td>0.09735</td><td>-1.432</td><td>0.152</td><td>0.02528</td><td>0.01099</td><td>0.988</td><td>0.008739</td><td>0.002306</td><td>0.007655</td><td>Vitamin D</td><td>FALSE</td><td></td><td></td><td></td><td>51453</td><td></td><td></td><td>UK Biobank</td><td>https://docs.google.com/spreadsheets/d/1kvPoupSzsSFBNSztMzl04xMoSC3Kcx3CrjVf4yBmESU/edit?ts=5b5f17db#gid=227859291</td><td>PHESANT Transformation:100021_0|| CONTINUOUS MAIN || CONTINUOUS || ||-Notes:Vitamin D. Estimated intake, based on food and beverage consumption yesterday, excluding any supplements.-Variable type:continuous_raw</td></tr>
<tr><td>100023_raw.txt</td><td>1710174270056F5forCTG.txt.gz</td><td>-0.2798</td><td>0.1036</td><td>-2.702</td><td>0.006902</td><td>0.02737</td><td>0.0112</td><td>1.004</td><td>0.009932</td><td>0.008854</td><td>0.007404</td><td>Starch</td><td>FALSE</td><td></td><td></td><td></td><td>51453</td><td></td><td></td><td>UK Biobank</td><td>https://docs.google.com/spreadsheets/d/1kvPoupSzsSFBNSztMzl04xMoSC3Kcx3CrjVf4yBmESU/edit?ts=5b5f17db#gid=227859291</td><td>PHESANT Transformation:100023_0|| CONTINUOUS MAIN || CONTINUOUS || ||-Notes:Starch. Estimated intake, based on food and beverage consumption yesterday, excluding any supplements.-Variable type:continuous_raw</td></tr>
<tr><td>100024_raw.txt</td><td>1710174270056F5forCTG.txt.gz</td><td>-0.00211</td><td>0.06841</td><td>-0.03084</td><td>0.9754</td><td>0.03842</td><td>0.01228</td><td>0.986</td><td>0.009584</td><td>-0.01165</td><td>0.007044</td><td>Calcium</td><td>FALSE</td><td></td><td></td><td></td><td>51453</td><td></td><td></td><td>UK Biobank</td><td>https://docs.google.com/spreadsheets/d/1kvPoupSzsSFBNSztMzl04xMoSC3Kcx3CrjVf4yBmESU/edit?ts=5b5f17db#gid=227859291</td><td>PHESANT Transformation:100024_0|| CONTINUOUS MAIN || CONTINUOUS || ||-Notes:Calcium. Estimated intake, based on food and beverage consumption yesterday, excluding any supplements.-Variable type:continuous_raw</td></tr>
<tr><td>100025_raw.txt</td><td>1710174270056F5forCTG.txt.gz</td><td>-0.1602</td><td>0.1045</td><td>-1.534</td><td>0.1251</td><td>0.02356</td><td>0.01128</td><td>1</td><td>0.009621</td><td>-0.001386</td><td>0.007451</td><td>Vitamin E</td><td>FALSE</td><td></td><td></td><td></td><td>51453</td><td></td><td></td><td>UK Biobank</td><td>https://docs.google.com/spreadsheets/d/1kvPoupSzsSFBNSztMzl04xMoSC3Kcx3CrjVf4yBmESU/edit?ts=5b5f17db#gid=227859291</td><td>PHESANT Transformation:100025_0|| CONTINUOUS MAIN || CONTINUOUS || ||-Notes:Vitamin E. Estimated intake, based on food and beverage consumption yesterday, excluding any supplements.-Variable type:continuous_raw</td></tr>
<tr><td>102_raw.txt</td><td>1710174270056F5forCTG.txt.gz</td><td>0.05505</td><td>0.02692</td><td>2.045</td><td>0.04082</td><td>0.1424</td><td>0.0114</td><td>1.08</td><td>0.05648</td><td>0.009351</td><td>0.01161</td><td>Pulse rate, automated reading</td><td>FALSE</td><td></td><td></td><td></td><td>340162</td><td></td><td></td><td>UK Biobank</td><td>https://docs.google.com/spreadsheets/d/1kvPoupSzsSFBNSztMzl04xMoSC3Kcx3CrjVf4yBmESU/edit?ts=5b5f17db#gid=227859291</td><td>PHESANT Transformation:102_0|| INTEGER || CONTINUOUS || ||-Notes:This is the pulse rate measured during the automated blood pressure readings.-Variable type:continuous_raw</td></tr>
<tr><td>12336_raw.txt</td><td>1710174270056F5forCTG.txt.gz</td><td>0.1855</td><td>0.09826</td><td>1.888</td><td>0.05904</td><td>0.1752</td><td>0.05449</td><td>0.998</td><td>0.01052</td><td>-0.006959</td><td>0.008683</td><td>Ventricular rate</td><td>FALSE</td><td></td><td></td><td></td><td>10817</td><td></td><td></td><td>UK Biobank</td><td>https://docs.google.com/spreadsheets/d/1kvPoupSzsSFBNSztMzl04xMoSC3Kcx3CrjVf4yBmESU/edit?ts=5b5f17db#gid=227859291</td><td>PHESANT Transformation:12336_2|| INTEGER || CONTINUOUS || ||-Notes:Ventricular rate during ECG measurement.-Variable type:continuous_raw</td></tr>
<tr><td>12338_raw.txt</td><td>1710174270056F5forCTG.txt.gz</td><td>-0.04485</td><td>0.1063</td><td>-0.422</td><td>0.673</td><td>0.1007</td><td>0.05189</td><td>0.993</td><td>0.009327</td><td>-0.001159</td><td>0.007682</td><td>P duration</td><td>FALSE</td><td></td><td></td><td></td><td>10785</td><td></td><td></td><td>UK Biobank</td><td>https://docs.google.com/spreadsheets/d/1kvPoupSzsSFBNSztMzl04xMoSC3Kcx3CrjVf4yBmESU/edit?ts=5b5f17db#gid=227859291</td><td>PHESANT Transformation:12338_2|| INTEGER || CONTINUOUS || ||-Notes:P duration during ECG-Variable type:continuous_raw</td></tr>
<tr><td>12340_raw.txt</td><td>1710174270056F5forCTG.txt.gz</td><td>0.1257</td><td>0.07146</td><td>1.76</td><td>0.07845</td><td>0.1731</td><td>0.06552</td><td>0.991</td><td>0.01138</td><td>-0.01719</td><td>0.007628</td><td>QRS duration</td><td>FALSE</td><td></td><td></td><td></td><td>10815</td><td></td><td></td><td>UK Biobank</td><td>https://docs.google.com/spreadsheets/d/1kvPoupSzsSFBNSztMzl04xMoSC3Kcx3CrjVf4yBmESU/edit?ts=5b5f17db#gid=227859291</td><td>PHESANT Transformation:12340_2|| INTEGER || CONTINUOUS || ||-Notes:QRS duration during ECG measurement-Variable type:continuous_raw</td></tr>
<tr><td>1438_raw.txt</td><td>1710174270056F5forCTG.txt.gz</td><td>-0.07297</td><td>0.03292</td><td>-2.216</td><td>0.02667</td><td>0.04834</td><td>0.003221</td><td>1.003</td><td>0.01335</td><td>-0.005034</td><td>0.008958</td><td>Bread intake</td><td>FALSE</td><td></td><td></td><td></td><td>353030</td><td></td><td></td><td>UK Biobank</td><td>https://docs.google.com/spreadsheets/d/1kvPoupSzsSFBNSztMzl04xMoSC3Kcx3CrjVf4yBmESU/edit?ts=5b5f17db#gid=227859291</td><td>PHESANT Transformation:1438_0|| INTEGER || reassignments: -1=NA|-3=NA|-10=NA || CONTINUOUS || ||-Notes:ACE touchscreen question How many slices of bread do you eat each WEEK? The following checks were performed: If answer If answer > 250 then rejected If answer > 50 then participant asked to confirm If the participant activated the Help button they were shown the message: For other types of bread: - one bread roll = 2 slices - one pitta bread = 2 slices-Variable type:continuous_raw</td></tr>
<tr><td>1488_raw.txt</td><td>1710174270056F5forCTG.txt.gz</td><td>0.09673</td><td>0.03577</td><td>2.705</td><td>0.006838</td><td>0.05683</td><td>0.004175</td><td>0.988</td><td>0.01546</td><td>0.02037</td><td>0.009686</td><td>Tea intake</td><td>FALSE</td><td></td><td></td><td></td><td>349376</td><td></td><td></td><td>UK Biobank</td><td>https://docs.google.com/spreadsheets/d/1kvPoupSzsSFBNSztMzl04xMoSC3Kcx3CrjVf4yBmESU/edit?ts=5b5f17db#gid=227859291</td><td>PHESANT Transformation:1488_0|| INTEGER || reassignments: -1=NA|-3=NA|-10=NA || CONTINUOUS || ||-Notes:ACE touchscreen question How many cups of tea do you drink each DAY? (Include black and green tea) The following checks were performed: If answer If answer > 99 then rejected If answer > 20 then participant asked to confirm If the participant activated the Help button they were shown the message: Please provide an average considering your intake over the last year. If you are unsure, please provide an estimate or select Do not know.-Variable type:continuous_raw</td></tr>
<tr><td>155142041596720170921_crude_pain_450K_cleaned.txt</td><td>1710174270056F5forCTG.txt.gz</td><td>0.3582</td><td>0.0358</td><td>10</td><td>1.46E-23</td><td>0.04003</td><td>0.002319</td><td>1.039</td><td>0.01432</td><td>0.006485</td><td>0.009259</td><td>Back pain</td><td>FALSE</td><td>Pain</td><td></td><td></td><td>509000</td><td>145000</td><td>364000</td><td>UK Biobank + CHARGE</td><td>https://zenodo.org/record/1319332</td><td>Meta-analysis results from UK Biobank and CHARGE provided in Freidin et al (2019) Insight into the genetic architecture of back pain and its risk factors from a study of 509,000 individuals</td></tr>
<tr><td>1552975371535tag.evrsmk.tbl</td><td>1710174270056F5forCTG.txt.gz</td><td>0.112</td><td>0.05851</td><td>1.913</td><td>0.0557</td><td>0.05542</td><td>0.007844</td><td>1.024</td><td>0.009738</td><td>0.01268</td><td>0.007716</td><td>Ever smoked</td><td>FALSE</td><td></td><td></td><td></td><td>74053</td><td>37000</td><td>37000</td><td>Tobacco and Genetics Consortium</td><td>https://www.med.unc.edu/pgc/results-and-downloads</td><td>Meta-analysis results from the Tobacco and Genetics Consortium (2010) Genome-wide meta-analyses identify multiple loci associated with smoking behavior. *Note that cases and control numbers is an approximate number based on manuscript information.</td></tr>
</table>

<table>
<tr><td>1552976047281BMI_GWAS_old-ctgvl.gwas</td><td>1710174270056F5forCTG.txt.gz</td><td>0.2221</td><td>0.0279</td><td>7.958</td><td>1.74E-15</td><td>0.08656</td><td>0.004457</td><td>0.678</td><td>0.01632</td><td>-0.000311</td><td>0.009222</td><td>BMI</td><td>FALSE</td><td>Body composition</td><td></td><td></td><td>339224</td><td></td><td></td><td>GIANT Consortium</td><td>https://portals.broadinstitute.org/collaboration/giant/index.php/GIANT_consortium_data_files</td><td>Meta-analysis results from the GIANT Consortium Consortium (2015) Genetic studies of body mass index yield new insights for obesity biology.</td></tr>
<tr><td>1552976231835GIANT_HEIGHT_Wood_et_al_2014_publicrelease_HapMapCeuFreq-ctgvl.txt</td><td>1710174270056F5forCTG.txt.gz</td><td>-0.04175</td><td>0.0239</td><td>-1.747</td><td>0.08067</td><td>0.3376</td><td>0.02104</td><td>1.21</td><td>0.05436</td><td>0.0002804</td><td>0.01277</td><td>Height</td><td>FALSE</td><td></td><td></td><td></td><td>253288</td><td></td><td></td><td>GIANT Consortium</td><td>https://portals.broadinstitute.org/collaboration/giant/index.php/GIANT_consortium_data_files</td><td>Meta-analysis results from the GIANT Consortium Consortium (2014) Defining the role of common variation in the genomic and biological architecture of adult human height.</td></tr>
<tr><td>1568940179895depression.ctgvl.input.gz</td><td>1710174270056F5forCTG.txt.gz</td><td>0.2912</td><td>0.02776</td><td>10.49</td><td>9.77E-26</td><td>0.03582</td><td>0.001787</td><td>1.022</td><td>0.01712</td><td>0.03665</td><td>0.009737</td><td>Major Depresive Disorder</td><td>FALSE</td><td>Psychiatric</td><td></td><td></td><td>480359</td><td>135458</td><td>344901</td><td>Psychiatric Genomics Consortium</td><td>http://dx.doi.org/10.1038/s41588-018-0090-3</td><td></td></tr>
<tr><td>1666964087006yyVL_DepressionNN.gz</td><td>1710174270056F5forCTG.txt.gz</td><td>0.3576</td><td>0.02585</td><td>13.83</td><td>1.68E-43</td><td>0.03968</td><td>0.001811</td><td>1.019</td><td>0.01975</td><td>0.02745</td><td>0.01051</td><td>Depression</td><td>FALSE</td><td>Psychiatric</td><td></td><td></td><td>670257</td><td>88328</td><td>581929</td><td>MVP+UKB+FinnGen</td><td>https://medicine.yale.edu/lab/gelernter/stats/</td><td>Bi-ancestral depression GWAS in the Million Veteran Program and meta-analysis (Excluding 23andMe)</td></tr>
<tr><td>1683048093043BCC_33549134-GCST90013410-EFO_0004193.h.tsv.ctg.gz</td><td>1710174270056F5forCTG.txt.gz</td><td>0.02291</td><td>0.02195</td><td>1.044</td><td>0.2966</td><td>0.03523</td><td>0.005413</td><td>1.03</td><td>0.02582</td><td>-0.01134</td><td>0.006421</td><td>Basal Cell Carcinoma</td><td>FALSE</td><td></td><td></td><td></td><td>392871</td><td>17416</td><td>375455</td><td>European</td><td>https://www.ebi.ac.uk/gwas/studies/GCST90013410</td><td>GWAS Meta-analysis Basal Cell Carcinoma</td></tr>
<tr><td>1696618005400CanEUR.gz</td><td>1710174270056F5forCTG.txt.gz</td><td>0.2931</td><td>0.03397</td><td>8.63</td><td>6.15E-18</td><td>0.08197</td><td>0.005524</td><td>1.006</td><td>0.01154</td><td>0.02539</td><td>0.00893</td><td>MVP Cannabis Meta</td><td>TRUE</td><td>Lifestyle</td><td></td><td>Cannabis use disorder</td><td>161053</td><td>42281</td><td>843744</td><td>Million Veteran Program, PGC, iPSYCH and MGH</td><td>https://medicine.yale.edu/lab/gelernter/stats/</td><td>Meta analysis of Cannabis Use Disorder in European Ancestry combining data from the Million Veteran Program, Psychiatric Genomics Consortium, MGH, and iPSCYH. Effective sample size reported under N.</td></tr>
<tr><td>1706801651650eGFRcrea_GSMR.txt.gz</td><td>1710174270056F5forCTG.txt.gz</td><td>0.002631</td><td>0.02668</td><td>0.09862</td><td>0.9214</td><td>0.07559</td><td>0.006702</td><td>0.883</td><td>0.07923</td><td>-0.02686</td><td>0.01236</td><td>creatinine-based estimated glomerular filtration rate (eGFR)</td><td>FALSE</td><td></td><td></td><td></td><td>1201909</td><td>1201909</td><td>0</td><td>CKDGen Consortium</td><td>https://ckdgen.imbi.uni-freiburg.de/datasets/Stanzick_2021</td><td>https://ckdgen.imbi.uni-freiburg.de/datasets/Stanzick_2021</td></tr>
<tr><td>1706803906180Urate_GSMR.txt.gz</td><td>1710174270056F5forCTG.txt.gz</td><td>0.08601</td><td>0.02592</td><td>3.319</td><td>0.000904</td><td>0.1447</td><td>0.04996</td><td>0.879</td><td>0.1367</td><td>-0.002577</td><td>0.009233</td><td>Urate</td><td>FALSE</td><td>Metabolic</td><td></td><td></td><td>457690</td><td>457690</td><td>0</td><td>CKDGen Consortium (EUR)</td><td>https://ckdgen.imbi.uni-freiburg.de/datasets/Tin_2019</td><td>https://ckdgen.imbi.uni-freiburg.de/datasets/Tin_2019</td></tr>
<tr><td>1706804136455eGFRcys_GSMR.txt.gz</td><td>1710174270056F5forCTG.txt.gz</td><td>-0.1254</td><td>0.02842</td><td>-4.414</td><td>1.02E-05</td><td>0.1281</td><td>0.02715</td><td>0.747</td><td>0.06354</td><td>-0.02397</td><td>0.009999</td><td>cystatin-based estimated glomerular filtration rate (eGFR</td><td>FALSE</td><td>Other (physical health)</td><td></td><td></td><td>460826</td><td>460826</td><td>0</td><td>CKDGen Consortium</td><td>https://ckdgen.imbi.uni-freiburg.de/datasets/Stanzick_2021</td><td>https://ckdgen.imbi.uni-freiburg.de/datasets/Stanzick_2021</td></tr>
<tr><td>1706805637264Gout_GSMR.txt.gz</td><td>1710174270056F5forCTG.txt.gz</td><td>0.08117</td><td>0.03717</td><td>2.184</td><td>0.029</td><td>0.01876</td><td>0.006683</td><td>0.984</td><td>0.05855</td><td>-0.001117</td><td>0.008976</td><td>Gout</td><td>FALSE</td><td></td><td></td><td></td><td>457690</td><td>457690</td><td>0</td><td>CKDGen Consortium (Transethnic)</td><td>https://ckdgen.imbi.uni-freiburg.de/datasets/Tin_2019</td><td>https://ckdgen.imbi.uni-freiburg.de/datasets/Tin_2019</td></tr>
<tr><td>1706805677763CKD_GSMR.txt.gz</td><td>1710174270056F5forCTG.txt.gz</td><td>-0.02197</td><td>0.04595</td><td>-0.4781</td><td>0.6326</td><td>0.01626</td><td>0.00249</td><td>1.053</td><td>0.01673</td><td>0.02834</td><td>0.008264</td><td>Chronic kidney disease (CKD)</td><td>FALSE</td><td></td><td></td><td></td><td>480698</td><td>41395</td><td>439303</td><td>CKDGen Consortium (EUR)</td><td>https://ckdgen.imbi.uni-freiburg.de/datasets/Wuttke_2019</td><td>Chronic kidney disease (CKD) European Meta-analysis</td></tr>
<tr><td>1706805749545UACR_GSMR.txt.gz</td><td>1710174270056F5forCTG.txt.gz</td><td>-0.05174</td><td>0.03167</td><td>-1.633</td><td>0.1024</td><td>0.04299</td><td>0.00271</td><td>0.964</td><td>0.0188</td><td>0.001763</td><td>0.009143</td><td>urinary albumin-to-creatinine ratio (UACR)</td><td>FALSE</td><td></td><td></td><td></td><td>547361</td><td>547361</td><td>0</td><td>CKDGen Consortium (EUR)</td><td>https://ckdgen.imbi.uni-freiburg.de/datasets/Teumer_2019</td><td>urinary albumin-to-creatinine ratio (UACR) meta-analysis</td></tr>
<tr><td>1706805815981microalbuminuria_GSMR.txt.gz</td><td>1710174270056F5forCTG.txt.gz</td><td>-0.01852</td><td>0.03763</td><td>-0.4921</td><td>0.6227</td><td>0.03165</td><td>0.002705</td><td>0.966</td><td>0.01333</td><td>0.01483</td><td>0.008338</td><td>Microalbuminuria</td><td>FALSE</td><td></td><td></td><td></td><td>347283</td><td>347283</td><td>0</td><td>CKDGen Consortium (Transethnic)</td><td>https://ckdgen.imbi.uni-freiburg.de/datasets/Teumer_2019</td><td>Microalbuminuria meta-analysis</td></tr>
<tr><td>1717.gwas.imputed_v3.both_sexes.tsv</td><td>1710174270056F5forCTG.txt.gz</td><td>-0.0248</td><td>0.02768</td><td>-0.8959</td><td>0.3703</td><td>0.1286</td><td>0.04056</td><td>0.936</td><td>0.1273</td><td>-0.001806</td><td>0.01091</td><td>Skin colour</td><td>FALSE</td><td></td><td></td><td></td><td>356530</td><td></td><td></td><td>UK Biobank</td><td>https://docs.google.com/spreadsheets/d/1kvPoupSzsSFBNSztMzl04xMoSC3Kcx3CrjVf4yBmESU/edit?ts=5b5f17db#gid=227859291</td><td>PHESANT Transformation:1717_0| | CAT-SINGLE | | Inc(>=10): 2(257373) | | Inc(>=10): 1(29331) | | Inc(>=10): 3(63017) | | Inc(>=10): 4(5341) | | Inc(>=10): 5(1447) | | Inc(>=10): 6(21) | | ordered | | CAT-ORD | | order: 1|2|3|4|5|6 | | num categories: 6 | |-Notes:ACE touchscreen question What best describes the colour of your skin without tanning? If the participant activated the Help button they were shown the message: If you are unsure, please provide an estimate or select Do not know.-Variable type:ordinal-Phenotype ID:1717</td></tr>
<tr><td>1727.gwas.imputed_v3.both_sexes.tsv</td><td>1710174270056F5forCTG.txt.gz</td><td>0.1079</td><td>0.03776</td><td>2.857</td><td>0.00428</td><td>0.1291</td><td>0.04553</td><td>0.96</td><td>0.1592</td><td>-0.009565</td><td>0.01045</td><td>Ease of skin tanning</td><td>FALSE</td><td></td><td></td><td></td><td>353697</td><td></td><td></td><td>UK Biobank</td><td>https://docs.google.com/spreadsheets/d/1kvPoupSzsSFBNSztMzl04xMoSC3Kcx3CrjVf4yBmESU/edit?ts=5b5f17db#gid=227859291</td><td>PHESANT Transformation:1727_0| | CAT-SINGLE | | Inc(>=10): 3(76520) | | Inc(>=10): 4(63175) | | Inc(>=10): 2(142944) | | Inc(>=10): 1(71058) | | ordered | | CAT-ORD | | order: 1|2|3|4 | | num categories: 4 | |-Notes:ACE touchscreen question What would happen to your skin if it was repeatedly exposed to bright sunlight without any protection?-Variable type:ordinal-Phenotype ID:1727</td></tr>
<tr><td>1737.gwas.imputed_v3.both_sexes.tsv</td><td>1710174270056F5forCTG.txt.gz</td><td>-0.0907</td><td>0.02815</td><td>-3.221</td><td>0.001276</td><td>0.08556</td><td>0.01847</td><td>0.962</td><td>0.06621</td><td>0.01051</td><td>0.009592</td><td>Childhood sunburn occasions</td><td>FALSE</td><td></td><td></td><td></td><td>269734</td><td></td><td></td><td>UK Biobank</td><td>https://docs.google.com/spreadsheets/d/1kvPoupSzsSFBNSztMzl04xMoSC3Kcx3CrjVf4yBmESU/edit?ts=5b5f17db#gid=227859291</td><td>PHESANT Transformation:1737_0| | INTEGER | | reassignments: -1=NA|-3=NA | | CONTINUOUS | | >20% IN ONE CATEGORY | | Split into three bins: 0: [0,0], 1: (0,2), 2: >=2 | |cat N: 139410, 40935, 89389 | | CAT-ORD | | order: 0|1|2 | | num categories: 3 | |-Notes:ACE touchscreen question Before the age of 15, how many times did you suffer sunburn that was painful for at least 2 days or caused blistering? The following checks were performed: If answer If answer > 999 then rejected If answer > 20 then participant asked to confirm If the participant activated the Help button they were shown the message: If you are unsure, please provide an estimate or select Do not know.-Variable type:ordinal-Phenotype ID:1737</td></tr>
<tr><td>1807_raw.txt</td><td>1710174270056F5forCTG.txt.gz</td><td>-0.2209</td><td>0.05214</td><td>-4.236</td><td>2.27E-05</td><td>0.02658</td><td>0.00314</td><td>1.01</td><td>0.01158</td><td>-0.01341</td><td>0.009034</td><td>Father's age at death</td><td>FALSE</td><td>Other</td><td></td><td></td><td>266231</td><td></td><td></td><td>UK Biobank</td><td>https://docs.google.com/spreadsheets/d/1kvPoupSzsSFBNSztMzl04xMoSC3Kcx3CrjVf4yBmESU/edit?ts=5b5f17db#gid=227859291</td><td>PHESANT Transformation:1807_0| | INTEGER | | reassignments: -1=NA|-3=NA | | CONTINUOUS | | | |-Notes:ACE touchscreen question What was his age when he died? The following checks were performed: If answer If answer > 122 then rejected If answer If answer > 105 then participant asked to confirm ~F1807~ was collected from participants who indicated their father has died, as defined by their answers to ~F1797~ or, if they are adopted, their adopted father has died, as defined by their answers to ~F1797~-Variable type:continuous_raw</td></tr>
<tr><td>189_raw.txt</td><td>1710174270056F5forCTG.txt.gz</td><td>0.3554</td><td>0.03572</td><td>9.95</td><td>2.53E-23</td><td>0.03313</td><td>0.002416</td><td>1.03</td><td>0.01207</td><td>0.01644</td><td>0.00856</td><td>Townsend deprivation index at recruitment</td><td>TRUE</td><td>Wellbeing</td><td></td><td>Townsend deprivation index</td><td>360763</td><td></td><td></td><td>UK Biobank</td><td>https://docs.google.com/spreadsheets/d/1kvPoupSzsSFBNSztMzl04xMoSC3Kcx3CrjVf4yBmESU/edit?ts=5b5f17db#gid=227859291</td><td>PHESANT Transformation:189_0| | CONTINUOUS MAIN | | CONTINUOUS | | | |-Notes:Townsend deprivation index calculated immediately prior to participant joining UK Biobank. Based on the preceding national census output areas. Each participant is assigned a score corresponding to the output area in which their postcode is located.-Variable type:continuous_raw</td></tr>
<tr><td>20001_1062.gwas.imputed_v3.both_sexes.txt</td><td>1710174270056F5forCTG.txt.gz</td><td>-0.09504</td><td>0.1416</td><td>-0.6712</td><td>0.5021</td><td>0.001716</td><td>0.001478</td><td>0.996</td><td>0.009944</td><td>1.72E-05</td><td>0.008325</td><td>Cancer code, self-reported: squamous cell carcinoma</td><td>FALSE</td><td></td><td></td><td></td><td>361141</td><td>449</td><td>360692</td><td>UK Biobank</td><td>http://www.nealelab.is/uk-biobank/</td><td>20001_0 | | CAT-MUL-BINARY-VAR 1062 | | Indicator name x134_0_0 | | Remove indicator var NAs: 53 | | Remove indicator var <0: 0 | | Removed 0 examples != 1062 but with missing value (<0) | | sample 360692/449(361141) | |</td></tr>
<tr><td>20007_raw.txt</td><td>1710174270056F5forCTG.txt.gz</td><td>-0.2874</td><td>0.1223</td><td>-2.351</td><td>0.01872</td><td>0.03355</td><td>0.01855</td><td>0.996</td><td>0.009475</td><td>0.006231</td><td>0.007726</td><td>Interpolated Age of participant when cancer first diagnosed</td><td>FALSE</td><td></td><td></td><td></td><td>30871</td><td></td><td></td><td>UK Biobank</td><td>https://docs.google.com/spreadsheets/d/1kvPoupSzsSFBNSztMzl04xMoSC3Kcx3CrjVf4yBmESU/edit?ts=5b5f17db#gid=227859291</td><td>PHESANT Transformation:20007_0| | CONTINUOUS MAIN | | reassignments: -1=NA|-3=NA | | CONTINUOUS | | | |-Notes:This is the interpolated time when the participant indicated the corresponding cancer was first diagnosed by a doctor, given as their estimated age. If the participant gave a calendar year, then the best-fit time is their age at the mid-point of that year. For example if the year was given as 1970, and the participant was born on 1 April 1950, then their age on 1st July 1970 is 20.25 then the value presented is 1970.5 If the participant gave their age then the value presented is the fractional year corresponding to the mid-point of that age. For example, if the participant said they were 30 years old then the value is 30.5 Interpolated values before the date of birth were truncated forwards to that time. Interpolated values after the time of data acquisition were truncated back to that time.-Variable type:continuous_raw</td></tr>
<tr><td>20015_raw.txt</td><td>1710174270056F5forCTG.txt.gz</td><td>-0.03599</td><td>0.02245</td><td>-1.603</td><td>0.1089</td><td>0.3369</td><td>0.02444</td><td>1.136</td><td>0.08487</td><td>0.006744</td><td>0.01255</td><td>Sitting height</td><td>FALSE</td><td></td><td></td><td></td><td>360066</td><td></td><td></td><td>UK Biobank</td><td>https://docs.google.com/spreadsheets/d/1kvPoupSzsSFBNSztMzl04xMoSC3Kcx3CrjVf4yBmESU/edit?ts=5b5f17db#gid=227859291</td><td>PHESANT Transformation:20015_0| | CONTINUOUS MAIN | | CONTINUOUS | | | |-Notes:Distance from rump to crown when sitting, calculated as the difference between ~F51~ and ~F3077~.-Variable type:continuous_raw</td></tr>
</table>

<table>
<tr><td>20016_raw.txt</td><td>1710174270056F5forCTG.txt.gz</td><td>-0.5469</td><td>0.02683</td><td>-20.38</td><td>2.31E-92</td><td>0.2266</td><td>0.011</td><td>1.026</td><td>0.01448</td><td>-0.008748</td><td>0.01022</td><td>Fluid intelligence score</td><td>TRUE</td><td>Cognitive</td><td>Cognitive</td><td>Fluid intelligence score</td><td>117131</td><td></td><td></td><td>UK Biobank</td><td>https://docs.google.com/spreadsheets/d/1kvPoupSzsSFBNSztMzl04xMoSC3Kcx3CrjVf4yBmESU/edit?ts=5b5f17db#gid=227859291</td><td>PHESANT Transformation:20016_0| | INTEGER | | CONTINUOUS | | | |-Notes:This is a simple unweighted sum of the number of correct answers given to the 13 fluid intelligence questions. Participants who did not answer all of the questions within the allotted 2 minute limit are scored as zero for each of the unattempted questions.-Variable type:continuous_raw</td></tr>
<tr><td>20019_raw.txt</td><td>1710174270056F5forCTG.txt.gz</td><td>0.2278</td><td>0.06938</td><td>3.284</td><td>0.001025</td><td>0.02329</td><td>0.004935</td><td>1.009</td><td>0.009786</td><td>-0.002214</td><td>0.007755</td><td>Speech-reception-threshold (SRT) estimate (left)</td><td>FALSE</td><td></td><td></td><td></td><td>114811</td><td></td><td></td><td>UK Biobank</td><td>https://docs.google.com/spreadsheets/d/1kvPoupSzsSFBNSztMzl04xMoSC3Kcx3CrjVf4yBmESU/edit?ts=5b5f17db#gid=227859291</td><td>PHESANT Transformation:20019_0| | CONTINUOUS MAIN | | CONTINUOUS | | | |-Notes:SRT is the Speech Reception Threshold, defined here as the signal-to-noise ratio at which half of the presented speech can be understood correctly. This was estimated as the value of the last round of the signal-to-noise ratio measurement for participants who completed all 15 rounds of the test (left-ear).-Variable type:continuous_raw</td></tr>
<tr><td>20021_raw.txt</td><td>1710174270056F5forCTG.txt.gz</td><td>0.2805</td><td>0.09322</td><td>3.009</td><td>0.002625</td><td>0.01743</td><td>0.005338</td><td>1.004</td><td>0.01018</td><td>-0.003341</td><td>0.008234</td><td>Speech-reception-threshold (SRT) estimate (right)</td><td>FALSE</td><td></td><td></td><td></td><td>114720</td><td></td><td></td><td>UK Biobank</td><td>https://docs.google.com/spreadsheets/d/1kvPoupSzsSFBNSztMzl04xMoSC3Kcx3CrjVf4yBmESU/edit?ts=5b5f17db#gid=227859291</td><td>PHESANT Transformation:20021_0| | CONTINUOUS MAIN | | CONTINUOUS | | | |-Notes:SRT is the Speech Reception Threshold, defined here as the signal-to-noise ratio at which half of the presented speech can be understood correctly. This was estimated as the value of the last round of the signal-to-noise ratio measurement for participants who completed all 15 rounds of the test (right-ear).-Variable type:continuous_raw</td></tr>
<tr><td>20022_raw.txt</td><td>1710174270056F5forCTG.txt.gz</td><td>-0.05094</td><td>0.03009</td><td>-1.693</td><td>0.09039</td><td>0.09418</td><td>0.006876</td><td>1.054</td><td>0.01985</td><td>-0.01084</td><td>0.008266</td><td>Birth weight</td><td>FALSE</td><td></td><td></td><td></td><td>205475</td><td></td><td></td><td>UK Biobank</td><td>https://docs.google.com/spreadsheets/d/1kvPoupSzsSFBNSztMzl04xMoSC3Kcx3CrjVf4yBmESU/edit?ts=5b5f17db#gid=227859291</td><td>PHESANT Transformation:20022_0| | CONTINUOUS MAIN | | CONTINUOUS | | | |-Notes:Participants were asked to enter their own birth-weight. The weight could be entered either in Kg or in Imperial pounds+ounces. Values entered as Imperial units are held in ~F121~ and ~F122~, and their values have been converted into Kg and incorporated into this field.-Variable type:continuous_raw</td></tr>
<tr><td>20023_raw.txt</td><td>1710174270056F5forCTG.txt.gz</td><td>-0.05733</td><td>0.03094</td><td>-1.853</td><td>0.06386</td><td>0.06272</td><td>0.003055</td><td>1.027</td><td>0.01517</td><td>-0.001388</td><td>0.009808</td><td>Mean time to correctly identify matches</td><td>FALSE</td><td></td><td></td><td></td><td>358695</td><td></td><td></td><td>UK Biobank</td><td>https://docs.google.com/spreadsheets/d/1kvPoupSzsSFBNSztMzl04xMoSC3Kcx3CrjVf4yBmESU/edit?ts=5b5f17db#gid=227859291</td><td>PHESANT Transformation:20023_0| | INTEGER | | CONTINUOUS | | | |-Notes:This field is the mean duration to first press of snap-button summed over rounds in which both cards matched. It gives a crude measure of the raw processing+reaction speed of a participant. The following data points were excluded when forming the average: Rounds 0-4 were regarded as training; Times under 50ms must be due to anticipation rather than reaction; Times over 2000ms were ignored as the cards had disappeared by then. Values were rounded to the nearest whole number.-Variable type:continuous_raw</td></tr>
<tr><td>20127_raw.txt</td><td>1710174270056F5forCTG.txt.gz</td><td>0.1271</td><td>0.03172</td><td>4.007</td><td>6.14E-05</td><td>0.1229</td><td>0.006123</td><td>0.983</td><td>0.02022</td><td>-0.01241</td><td>0.01014</td><td>Neuroticism score</td><td>FALSE</td><td></td><td></td><td></td><td>293006</td><td></td><td></td><td>UK Biobank</td><td>https://docs.google.com/spreadsheets/d/1kvPoupSzsSFBNSztMzl04xMoSC3Kcx3CrjVf4yBmESU/edit?ts=5b5f17db#gid=227859291</td><td>PHESANT Transformation:20127_0| | INTEGER | | CONTINUOUS | | | |-Notes:This is an externally derived summary score of neuroticism, based on 12 neurotic behaviour domains as reported from fields 1920, 1930, 1940, 1950, 1960, 1970, 1980, 1990, 2000, 2010, 2020 and 2030 from the touchscreen questionnaire at baseline. Participants were assessed for twelve domains of neurotic behaviours via the touchscreen questionnaire. Questions included: Does your mood often go up and down? Do you ever feel 'just miserable' for no reason? Are you an irritable person? Are your feelings easily hurt? Do you often feel 'fed-up'? Would you call yourself a nervous person? Are you a worrier? Would you call yourself tense or 'highly strung'? Do you worry too long after an embarrassing experience? Do you suffer from 'nerves'? Do you often feel lonely? Are you often troubled by feelings of guilt? Participants could answer Yes, No, Do not know or Prefer not to answer. This field summarises the number of Yes answers across these twelve questions into a single integer score for each participant. This derived data field has come from Professor Jill Pell from the Institute of Health & Wellbeing, University of Glasgow. Methods on how these fields were derived can be found in the Additional Resources tab.-Variable type:continuous_raw</td></tr>
<tr><td>20150_raw.txt</td><td>1710174270056F5forCTG.txt.gz</td><td>-0.1294</td><td>0.02671</td><td>-4.845</td><td>1.27E-06</td><td>0.2157</td><td>0.01054</td><td>1.057</td><td>0.02761</td><td>-0.01876</td><td>0.01118</td><td>Forced expiratory volume in 1-second (FEV1), Best measure</td><td>FALSE</td><td>Pulmonary</td><td></td><td></td><td>272338</td><td></td><td></td><td>UK Biobank</td><td>https://docs.google.com/spreadsheets/d/1kvPoupSzsSFBNSztMzl04xMoSC3Kcx3CrjVf4yBmESU/edit?ts=5b5f17db#gid=227859291</td><td>PHESANT Transformation:20150_0| | CONTINUOUS MAIN | | CONTINUOUS | | | |-Notes:Highest measure from the array of values for Forced Expiratory Volume in 1-second (FEV1) ~F3063~, which was acceptable according to corresponding acceptability field (~F3061~). A blow was deemed acceptable if recorded as 0 (no problems) or 32 (0x20 - USER_ACCEPTED). This derived data field has come from Professor Martin Tobin at the University of Leicester.-Variable type:continuous_raw</td></tr>
<tr><td>20151_raw.txt</td><td>1710174270056F5forCTG.txt.gz</td><td>-0.1363</td><td>0.02551</td><td>-5.344</td><td>9.11E-08</td><td>0.2373</td><td>0.01218</td><td>1.076</td><td>0.03264</td><td>-0.02008</td><td>0.01144</td><td>Forced vital capacity (FVC), Best measure</td><td>FALSE</td><td>Pulmonary</td><td></td><td></td><td>272338</td><td></td><td></td><td>UK Biobank</td><td>https://docs.google.com/spreadsheets/d/1kvPoupSzsSFBNSztMzl04xMoSC3Kcx3CrjVf4yBmESU/edit?ts=5b5f17db#gid=227859291</td><td>PHESANT Transformation:20151_0| | CONTINUOUS MAIN | | CONTINUOUS | | | |-Notes:Highest measure from the array of values for Forced Vital Capacity (FVC) (~F3062~), which was acceptable according to corresponding acceptability field (~F3061~). A blow was deemed acceptable if recorded as 0 (no problems) or 32 (0x20 - USER_ACCEPTED). This derived data field has come from Professor Martin Tobin at the University of Leicester.-Variable type:continuous_raw</td></tr>
<tr><td>20153_raw.txt</td><td>1710174270056F5forCTG.txt.gz</td><td>-0.08264</td><td>0.02623</td><td>-3.151</td><td>0.001627</td><td>0.4559</td><td>0.0325</td><td>1.065</td><td>0.0389</td><td>0.004979</td><td>0.0112</td><td>Forced expiratory volume in 1-second (FEV1), predicted</td><td>FALSE</td><td></td><td></td><td></td><td>117241</td><td></td><td></td><td>UK Biobank</td><td>https://docs.google.com/spreadsheets/d/1kvPoupSzsSFBNSztMzl04xMoSC3Kcx3CrjVf4yBmESU/edit?ts=5b5f17db#gid=227859291</td><td>PHESANT Transformation:20153_0| | CONTINUOUS MAIN | | CONTINUOUS | | | |-Notes:Predicted Forced Expiratory Volume in 1-second (FEV1), was calculated using a subset of healthy never smokers from UK Biobank. Healthy never smokers, were selected as individuals with Ever Smoking Status=0 and with Reproducible measure using the European Respiratory Society/American Thoracic Society (ERS/ATS) Criteria=YES (1), with the following exclusions: ~F2316~: Individuals who indicated that they had experienced wheeze: Yes (1), Do not know (-1) or Prefer not to answer (-3). ~F6152~: Individuals who reported emphysema/chronic bronchitis (6) or asthma (8) or Prefer not to answer (-3). ~F20002~: Individuals who reported any of the following: asthma (1111), COPD (1112), emphysema/chronic bronchitis (1113), bronchiectasis (1114), interstitial lung disease (1115), asbestosis (1120), pulmonary fibrosis (1121), fibrosing/unspecified alveolitis (1122), respiratory failure (1124), pleurisy (1125), spontaneous/recurrent pneumothorax (1126), other respiratory problems (1117). Healthy never smokers were grouped into 58 age-sex bands (29 age bands per sex; ages 39, 40 and 41 were grouped into one band and ages 69, 70 and 72 were grouped into one band with ages 42 to 68 each forming a separate band) and the following linear regression model was fitted in each age-sex band: FEV1 = Beta0 + Beta1 * Standing Height Predicted FEV1 was then calculated for all samples who were never smokers (Ever Smoking Status=0) or heavy smokers (Ever Smoking Status=1 AND Pack years as proportion of life span exposed to smoking >= 0.42) and with Reproducible measure using the European Respiratory Society/American Thoracic Society (ERS/ATS) Criteria=YES, using the estimates of Beta0 and Beta1 , from the appropriate age-sex band: Predicted FEV1= (pred. Beta0) + (pred. Beta1)^ * Standing Height Five individuals were outliers in terms of their FEV1 and were not coded. This derived data field has come from Professor Martin Tobin at the University of Leicester.-Variable type:continuous_raw</td></tr>
<tr><td>20154_raw.txt</td><td>1710174270056F5forCTG.txt.gz</td><td>-0.103</td><td>0.03217</td><td>-3.201</td><td>0.001371</td><td>0.1999</td><td>0.01052</td><td>1.012</td><td>0.01558</td><td>-0.02034</td><td>0.00919</td><td>Forced expiratory volume in 1-second (FEV1), predicted percentage</td><td>FALSE</td><td></td><td></td><td></td><td>117241</td><td></td><td></td><td>UK Biobank</td><td>https://docs.google.com/spreadsheets/d/1kvPoupSzsSFBNSztMzl04xMoSC3Kcx3CrjVf4yBmESU/edit?ts=5b5f17db#gid=227859291</td><td>PHESANT Transformation:20154_0| | CONTINUOUS MAIN | | CONTINUOUS | | | |-Notes:This value was only calculated for samples with non-missing predicted Forced Expiratory Volume in 1-second, using derived variables Force Expiratory Volume in 1-second (FEV1) and predicted Forced Expiratory Volume in 1-second (pred.FEV1). It is defined as the Force Expiratory Volume in 1-second divided by the Percent predicted Forced Expiratory Volume in 1-second multiplied by 100: FEV1 /pred.FEV1 * 100 This derived data field has come from Professor Martin Tobin at the University of Leicester.-Variable type:continuous_raw</td></tr>
<tr><td>20420_raw.txt</td><td>1710174270056F5forCTG.txt.gz</td><td>0.3103</td><td>0.1253</td><td>2.476</td><td>0.01328</td><td>0.04389</td><td>0.02239</td><td>0.991</td><td>0.009093</td><td>0.002677</td><td>0.008171</td><td>Longest period spent worried or anxious</td><td>FALSE</td><td></td><td></td><td></td><td>24738</td><td></td><td></td><td>UK Biobank</td><td>https://docs.google.com/spreadsheets/d/1kvPoupSzsSFBNSztMzl04xMoSC3Kcx3CrjVf4yBmESU/edit?ts=5b5f17db#gid=227859291</td><td>PHESANT Transformation:20420_0| | INTEGER | | reassignments: -999=NA | | CONTINUOUS | | | |-Notes:Question asked: What is the longest period of time that this kind of worrying has ever continued? Question was asked when ~F20421~ was Yes.-Variable type:continuous_raw</td></tr>
<tr><td>20433_raw.txt</td><td>1710174270056F5forCTG.txt.gz</td><td>-0.04212</td><td>0.07479</td><td>-0.5632</td><td>0.5733</td><td>0.03671</td><td>0.009504</td><td>1.004</td><td>0.009753</td><td>-0.007281</td><td>0.007597</td><td>Age at first episode of depression</td><td>FALSE</td><td></td><td></td><td></td><td>61033</td><td></td><td></td><td>UK Biobank</td><td>https://docs.google.com/spreadsheets/d/1kvPoupSzsSFBNSztMzl04xMoSC3Kcx3CrjVf4yBmESU/edit?ts=5b5f17db#gid=227859291</td><td>PHESANT Transformation:20433_0| | INTEGER | | reassignments: -121=NA|-818=NA | | CONTINUOUS | | | |-Notes:Question asked: About how old were you the FIRST time you had a period of two weeks like this? (Whether or not you received any help for it.) Question was asked when ~F20446~ was Yes or ~F20441~ was Yes.-Variable type:continuous_raw</td></tr>
<tr><td>20434_raw.txt</td><td>1710174270056F5forCTG.txt.gz</td><td>0.2655</td><td>0.07924</td><td>3.351</td><td>0.000805</td><td>0.03664</td><td>0.009167</td><td>0.995</td><td>0.01019</td><td>0.001228</td><td>0.00782</td><td>Age at last episode of depression</td><td>FALSE</td><td></td><td></td><td></td><td>59260</td><td></td><td></td><td>UK Biobank</td><td>https://docs.google.com/spreadsheets/d/1kvPoupSzsSFBNSztMzl04xMoSC3Kcx3CrjVf4yBmESU/edit?ts=5b5f17db#gid=227859291</td><td>PHESANT Transformation:20434_0| | INTEGER | | reassignments: -121=NA|-818=NA | | CONTINUOUS | | | |-Notes:Question asked: About how old were you the LAST time you had a period of two weeks like this? (Whether or not you received any help for it.) Question was asked when ~F20446~ was Yes or ~F20441~ was Yes.-Variable type:continuous_raw</td></tr>
<tr><td>20455_raw.txt</td><td>1710174270056F5forCTG.txt.gz</td><td>0.152</td><td>0.1062</td><td>1.432</td><td>0.1523</td><td>0.05033</td><td>0.0233</td><td>0.987</td><td>0.009605</td><td>0.01179</td><td>0.007713</td><td>Age when last took cannabis</td><td>FALSE</td><td></td><td></td><td></td><td>25698</td><td></td><td></td><td>UK Biobank</td><td>https://docs.google.com/spreadsheets/d/1kvPoupSzsSFBNSztMzl04xMoSC3Kcx3CrjVf4yBmESU/edit?ts=5b5f17db#gid=227859291</td><td>PHESANT Transformation:20455_0| | INTEGER | | CONTINUOUS | | | |-Notes:Question asked: About how old were you when you last had cannabis? Question was asked unless the answer to ~F20453~ was No.-Variable type:continuous_raw</td></tr>
<tr><td>21001_raw.txt</td><td>1710174270056F5forCTG.txt.gz</td><td>0.2866</td><td>0.02396</td><td>11.96</td><td>5.79E-33</td><td>0.233</td><td>0.009459</td><td>1.091</td><td>0.03397</td><td>0.02837</td><td>0.01267</td><td>Body mass index (BMI)</td><td>TRUE</td><td>Body composition</td><td></td><td>Body mass index</td><td>359983</td><td></td><td></td><td>UK Biobank</td><td>https://docs.google.com/spreadsheets/d/1kvPoupSzsSFBNSztMzl04xMoSC3Kcx3CrjVf4yBmESU/edit?ts=5b5f17db#gid=227859291</td><td>PHESANT Transformation:21001_0| | CONTINUOUS MAIN | | CONTINUOUS | | | |-Notes:BMI value here is constructed from height and weight measured during the initial Assessment Centre visit. Value is not present if either of these readings were omitted.-Variable type:continuous_raw</td></tr>
<tr><td>21002_raw.txt</td><td>1710174270056F5forCTG.txt.gz</td><td>0.2126</td><td>0.02376</td><td>8.948</td><td>3.61E-19</td><td>0.2513</td><td>0.01078</td><td>1.094</td><td>0.03723</td><td>0.03379</td><td>0.01193</td><td>Weight</td><td>FALSE</td><td>Body composition</td><td></td><td></td><td>360116</td><td></td><td></td><td>UK Biobank</td><td>https://docs.google.com/spreadsheets/d/1kvPoupSzsSFBNSztMzl04xMoSC3Kcx3CrjVf4yBmESU/edit?ts=5b5f17db#gid=227859291</td><td>PHESANT Transformation:21002_0| | CONTINUOUS MAIN | | CONTINUOUS | | | |-Notes:Weight was measured by a variety of means during the initial Assessment Centre visit. This field amalgamates these values into a single item.-Variable type:continuous_raw</td></tr>
</table>

<table>
<tr><th></th><th></th><th></th><th></th><th></th><th></th><th></th><th></th><th></th><th></th><th></th><th></th><th></th><th></th><th></th><th></th><th></th><th></th><th></th><th></th><th></th><th></th><th></th></tr>
<tr><td>21021_raw.txt</td><td>1710174270056F5forCTG.txt.gz</td><td>0.1269</td><td>0.06858</td><td>1.85</td><td>0.06426</td><td>0.02252</td><td>0.005428</td><td>1.006</td><td>0.01062</td><td>0.01271</td><td>0.007307</td><td>Pulse wave Arterial Stiffness index</td><td>FALSE</td><td></td><td></td><td></td><td>118469</td><td></td><td></td><td>UK Biobank</td><td>https://docs.google.com/spreadsheets/d/1kvPoupSzsSFBNSztMzl04xMoSC3Kcx3CrjVf4yBmESU/edit?ts=5b5f17db#gid=227859291</td><td>PHESANT Transformation:21021_0| | CONTINUOUS MAIN | | CONTINUOUS | | | |-Notes:The time between peaks of the waveform (the peak-to-peak time) is divided into the persons height to obtain the Stiffness Index. This value was calculated outside the Assessment Centre visit as the participant height was not known until after the stiffness device had been used.-Variable type:continuous_raw</td></tr>
<tr><td>2139_raw.txt</td><td>1710174270056F5forCTG.txt.gz</td><td>-0.3987</td><td>0.02566</td><td>-15.53</td><td>2.03E-54</td><td>0.12</td><td>0.005631</td><td>1.031</td><td>0.02216</td><td>-0.0171</td><td>0.01151</td><td>Age first had sexual intercourse</td><td>FALSE</td><td>Reproductive</td><td></td><td></td><td>317694</td><td></td><td></td><td>UK Biobank</td><td>https://docs.google.com/spreadsheets/d/1kvPoupSzsSFBNSztMzl04xMoSC3Kcx3CrjVf4yBmESU/edit?ts=5b5f17db#gid=227859291</td><td>PHESANT Transformation:2139_0| | INTEGER | | reassignments: -1=NA|-2=NA|-3=NA | | CONTINUOUS | | | |-Notes:ACE touchscreen question What was your age when you first had sexual intercourse? (Sexual intercourse includes vaginal, oral or anal intercourse) The following checks were performed: If answer If answer > Participants age then rejected If answer If the participant activated the Help button they were shown the message: Sexual intercourse includes vaginal, oral or anal intercourse. If you are unsure, please provide an estimate or select Do not know.-Variable type:continuous_raw</td></tr>
<tr><td>22146_raw.txt</td><td>1710174270056F5forCTG.txt.gz</td><td>0.1419</td><td>0.06957</td><td>2.04</td><td>0.04131</td><td>0.1106</td><td>0.02583</td><td>1</td><td>0.009306</td><td>-0.007181</td><td>0.0082</td><td>Age hayfever or allergic rhinitis diagnosed by doctor</td><td>FALSE</td><td></td><td></td><td></td><td>20904</td><td></td><td></td><td>UK Biobank</td><td>https://docs.google.com/spreadsheets/d/1kvPoupSzsSFBNSztMzl04xMoSC3Kcx3CrjVf4yBmESU/edit?ts=5b5f17db#gid=227859291</td><td>PHESANT Transformation:22146_0| | INTEGER | | CONTINUOUS | | | |-Notes:User was asked the age at which doctor diagnosed hayfever or allergic rhinitis-Variable type:continuous_raw</td></tr>
<tr><td>22147_raw.txt</td><td>1710174270056F5forCTG.txt.gz</td><td>0.2404</td><td>0.08269</td><td>2.908</td><td>0.00364</td><td>0.1737</td><td>0.05811</td><td>0.998</td><td>0.01049</td><td>-0.007738</td><td>0.007863</td><td>Age asthma diagnosed by doctor</td><td>FALSE</td><td></td><td></td><td></td><td>11717</td><td></td><td></td><td>UK Biobank</td><td>https://docs.google.com/spreadsheets/d/1kvPoupSzsSFBNSztMzl04xMoSC3Kcx3CrjVf4yBmESU/edit?ts=5b5f17db#gid=227859291</td><td>PHESANT Transformation:22147_0| | INTEGER | | CONTINUOUS | | | |-Notes:User was asked the age at which doctor diagnosed asthma-Variable type:continuous_raw</td></tr>
<tr><td>2217_raw.txt</td><td>1710174270056F5forCTG.txt.gz</td><td>0.1444</td><td>0.02832</td><td>5.098</td><td>3.42E-07</td><td>0.09452</td><td>0.00549</td><td>1.048</td><td>0.01941</td><td>-0.001776</td><td>0.01017</td><td>Age started wearing glasses or contact lenses</td><td>TRUE</td><td>Ophthalmic</td><td>Physical health</td><td>Age started wearing glasses or contact lenses</td><td>310992</td><td></td><td></td><td>UK Biobank</td><td>https://docs.google.com/spreadsheets/d/1kvPoupSzsSFBNSztMzl04xMoSC3Kcx3CrjVf4yBmESU/edit?ts=5b5f17db#gid=227859291</td><td>PHESANT Transformation:2217_0| | INTEGER | | reassignments: -1=NA|-3=NA | | CONTINUOUS | | | |-Notes:ACE touchscreen question What age did you first start to wear glasses or contact lenses? The following checks were performed: If answer If answer > Participants age then rejected If answer If the participant activated the Help button they were shown the message: If you are unsure, please provide an estimate or select Do not know. ~F2217~ was collected from participants who indicated they wear glasses or contact lenses to correct their vision, as defined by their answers to ~F2207~-Variable type:continuous_raw</td></tr>
<tr><td>22501_raw.txt</td><td>1710174270056F5forCTG.txt.gz</td><td>-0.4489</td><td>0.03949</td><td>-11.37</td><td>6.24E-30</td><td>0.1318</td><td>0.008785</td><td>1.055</td><td>0.01149</td><td>-0.007526</td><td>0.009118</td><td>Year ended full time education</td><td>TRUE</td><td>Education</td><td>Education</td><td>Year ended full time education</td><td>91777</td><td></td><td></td><td>UK Biobank</td><td>https://docs.google.com/spreadsheets/d/1kvPoupSzsSFBNSztMzl04xMoSC3Kcx3CrjVf4yBmESU/edit?ts=5b5f17db#gid=227859291</td><td>PHESANT Transformation:22501_0| | INTEGER | | reassignments: -1=NA|-2=NA|-3=NA | | CONTINUOUS | | | |-Notes:Year ended full time education-Variable type:continuous_raw</td></tr>
<tr><td>22503_raw.txt</td><td>1710174270056F5forCTG.txt.gz</td><td>0.1514</td><td>0.1667</td><td>0.9086</td><td>0.3636</td><td>0.04698</td><td>0.04036</td><td>0.999</td><td>0.008489</td><td>0.001128</td><td>0.007933</td><td>Years of cough on most days</td><td>FALSE</td><td></td><td></td><td></td><td>12593</td><td></td><td></td><td>UK Biobank</td><td>https://docs.google.com/spreadsheets/d/1kvPoupSzsSFBNSztMzl04xMoSC3Kcx3CrjVf4yBmESU/edit?ts=5b5f17db#gid=227859291</td><td>PHESANT Transformation:22503_0| | INTEGER | | CONTINUOUS | | | |-Notes:Participants who indicated they coughed on most days were asked: For how many years have you had this cough?-Variable type:continuous_raw</td></tr>
<tr><td>22507_raw.txt</td><td>1710174270056F5forCTG.txt.gz</td><td>0.09477</td><td>0.1039</td><td>0.912</td><td>0.3617</td><td>0.03189</td><td>0.01659</td><td>1.01</td><td>0.009184</td><td>0.009244</td><td>0.007768</td><td>Age of stopping smoking</td><td>FALSE</td><td></td><td></td><td></td><td>33335</td><td></td><td></td><td>UK Biobank</td><td>https://docs.google.com/spreadsheets/d/1kvPoupSzsSFBNSztMzl04xMoSC3Kcx3CrjVf4yBmESU/edit?ts=5b5f17db#gid=227859291</td><td>PHESANT Transformation:22507_0| | INTEGER | | CONTINUOUS | | | |-Notes:Participants who had stopped smoking where asked: At what age did you give up?-Variable type:continuous_raw</td></tr>
<tr><td>22644_raw.txt</td><td>1710174270056F5forCTG.txt.gz</td><td>-0.1178</td><td>0.1668</td><td>-0.7062</td><td>0.48</td><td>0.02787</td><td>0.02609</td><td>0.999</td><td>0.009255</td><td>0.009278</td><td>0.008263</td><td>Consecutive night shifts during mixed shift periods</td><td>FALSE</td><td></td><td></td><td></td><td>19344</td><td></td><td></td><td>UK Biobank</td><td>https://docs.google.com/spreadsheets/d/1kvPoupSzsSFBNSztMzl04xMoSC3Kcx3CrjVf4yBmESU/edit?ts=5b5f17db#gid=227859291</td><td>PHESANT Transformation:22644_0| | INTEGER | | reassignments: -1=NA | | CONTINUOUS | | | |-Notes:Participants who worked a mix of day and night shifts were asked: Please tell us about the most common night shift pattern you had during this time: How many night shifts in a row did you usually work before a change of shift or rest day?-Variable type:continuous_raw</td></tr>
<tr><td>2277.gwas.imputed_v3.both_sexes.tsv</td><td>1710174270056F5forCTG.txt.gz</td><td>-0.3952</td><td>0.05319</td><td>-7.431</td><td>1.08E-13</td><td>0.0179</td><td>0.002144</td><td>1.02</td><td>0.01155</td><td>0.01824</td><td>0.009088</td><td>Frequency of solarium/sunlamp use</td><td>FALSE</td><td>Lifestyle</td><td></td><td></td><td>340643</td><td></td><td></td><td>UK Biobank</td><td>https://docs.google.com/spreadsheets/d/1kvPoupSzsSFBNSztMzl04xMoSC3Kcx3CrjVf4yBmESU/edit?ts=5b5f17db#gid=227859291</td><td>PHESANT Transformation:2277_0| | INTEGER | | reassignments: -1=NA|-3=NA|-10=NA | | CONTINUOUS | | >20% IN ONE CATEGORY | | Split into three bins: 0: [0,0], 1: (0,6), 2: >=6 | | cat N: 324579, 6805, 9259 | | CAT-ORD | | order: 0|1|2 | | num categories: 3 | |-Notes:ACE touchscreen question How many times a year would you use a solarium or sunlamp? The following checks were performed: If answer > 9999 then rejected If answer > 400 then participant asked to confirm If the participant activated the Help button they were shown the message: If you are unsure, please provide an estimate or select Do not know.-Variable type:ordinal-Phenotype ID:2277</td></tr>
<tr><td>23098_raw.txt</td><td>1710174270056F5forCTG.txt.gz</td><td>0.2111</td><td>0.0239</td><td>8.835</td><td>9.99E-19</td><td>0.2511</td><td>0.01077</td><td>1.094</td><td>0.03704</td><td>0.03403</td><td>0.01204</td><td>Weight</td><td>FALSE</td><td>Body composition</td><td></td><td></td><td>354838</td><td></td><td></td><td>UK Biobank</td><td>https://docs.google.com/spreadsheets/d/1kvPoupSzsSFBNSztMzl04xMoSC3Kcx3CrjVf4yBmESU/edit?ts=5b5f17db#gid=227859291</td><td>PHESANT Transformation:23098_0| | CONTINUOUS MAIN | | CONTINUOUS | | | |-Notes:Weight of person taken during impedance measurement. 0.1 increments. Range 0 - 200Kg.-Variable type:continuous_raw</td></tr>
<tr><td>23099_raw.txt</td><td>1710174270056F5forCTG.txt.gz</td><td>0.2857</td><td>0.02255</td><td>12.67</td><td>8.83E-37</td><td>0.2204</td><td>0.008787</td><td>1.095</td><td>0.03179</td><td>0.01579</td><td>0.01232</td><td>Body fat percentage</td><td>FALSE</td><td>Body composition</td><td></td><td></td><td>354628</td><td></td><td></td><td>UK Biobank</td><td>https://docs.google.com/spreadsheets/d/1kvPoupSzsSFBNSztMzl04xMoSC3Kcx3CrjVf4yBmESU/edit?ts=5b5f17db#gid=227859291</td><td>PHESANT Transformation:23099_0| | CONTINUOUS MAIN | | CONTINUOUS | | | |-Notes:Body composition estimation by impedance measurement. Body fat percentage Body fat percentage. Range 1% - 75% in 0.1% increments.-Variable type:continuous_raw</td></tr>
<tr><td>23100_raw.txt</td><td>1710174270056F5forCTG.txt.gz</td><td>0.2698</td><td>0.02309</td><td>11.68</td><td>1.56E-31</td><td>0.2231</td><td>0.00905</td><td>1.088</td><td>0.0323</td><td>0.02655</td><td>0.01195</td><td>Whole body fat mass</td><td>FALSE</td><td>Body composition</td><td></td><td></td><td>354244</td><td></td><td></td><td>UK Biobank</td><td>https://docs.google.com/spreadsheets/d/1kvPoupSzsSFBNSztMzl04xMoSC3Kcx3CrjVf4yBmESU/edit?ts=5b5f17db#gid=227859291</td><td>PHESANT Transformation:23100_0| | CONTINUOUS MAIN | | CONTINUOUS | | | |-Notes:Body composition estimation by impedance measurement. Fat mass Total fat mass in Kg, in 0.1Kg increments.-Variable type:continuous_raw</td></tr>
<tr><td>23101_raw.txt</td><td>1710174270056F5forCTG.txt.gz</td><td>0.0919</td><td>0.02454</td><td>3.745</td><td>0.00018</td><td>0.2895</td><td>0.01547</td><td>1.119</td><td>0.0525</td><td>0.03625</td><td>0.01267</td><td>Whole body fat-free mass</td><td>FALSE</td><td></td><td></td><td></td><td>354808</td><td></td><td></td><td>UK Biobank</td><td>https://docs.google.com/spreadsheets/d/1kvPoupSzsSFBNSztMzl04xMoSC3Kcx3CrjVf4yBmESU/edit?ts=5b5f17db#gid=227859291</td><td>PHESANT Transformation:23101_0| | CONTINUOUS MAIN | | CONTINUOUS | | | |-Notes:Body composition estimation by impedance measurement. Fat free mass Fat free mass in Kg. Increment in 0.1 Kg-Variable type:continuous_raw</td></tr>
<tr><td>23102_raw.txt</td><td>1710174270056F5forCTG.txt.gz</td><td>0.0937</td><td>0.02453</td><td>3.819</td><td>0.000134</td><td>0.2886</td><td>0.01537</td><td>1.12</td><td>0.05222</td><td>0.03612</td><td>0.01266</td><td>Whole body water mass</td><td>FALSE</td><td></td><td></td><td></td><td>354834</td><td></td><td></td><td>UK Biobank</td><td>https://docs.google.com/spreadsheets/d/1kvPoupSzsSFBNSztMzl04xMoSC3Kcx3CrjVf4yBmESU/edit?ts=5b5f17db#gid=227859291</td><td>PHESANT Transformation:23102_0| | CONTINUOUS MAIN | | CONTINUOUS | | | |-Notes:Body composition estimation by impedance measurement. Body water mass Units in Kg. Increments in 0.1 Kg.-Variable type:continuous_raw</td></tr>
<tr><td>23104_raw.txt</td><td>1710174270056F5forCTG.txt.gz</td><td>0.2845</td><td>0.02414</td><td>11.79</td><td>4.58E-32</td><td>0.2328</td><td>0.009421</td><td>1.092</td><td>0.0334</td><td>0.02936</td><td>0.01277</td><td>Body mass index (BMI)</td><td>TRUE</td><td>Body composition</td><td></td><td>Body mass index</td><td>354831</td><td></td><td></td><td>UK Biobank</td><td>https://docs.google.com/spreadsheets/d/1kvPoupSzsSFBNSztMzl04xMoSC3Kcx3CrjVf4yBmESU/edit?ts=5b5f17db#gid=227859291</td><td>PHESANT Transformation:23104_0| | CONTINUOUS MAIN | | CONTINUOUS | | | |-Notes:Body composition estimation by impedance measurement. Body Mass Index. Increments of 0.1 .-Variable type:continuous_raw</td></tr>
<tr><td>23105_raw.txt</td><td>1710174270056F5forCTG.txt.gz</td><td>0.1256</td><td>0.0246</td><td>5.106</td><td>3.28E-07</td><td>0.2763</td><td>0.01397</td><td>1.108</td><td>0.04757</td><td>0.03614</td><td>0.01238</td><td>Basal metabolic rate</td><td>TRUE</td><td>Metabolic</td><td></td><td>Basal metabolic rate</td><td>354825</td><td></td><td></td><td>UK Biobank</td><td>https://docs.google.com/spreadsheets/d/1kvPoupSzsSFBNSztMzl04xMoSC3Kcx3CrjVf4yBmESU/edit?ts=5b5f17db#gid=227859291</td><td>PHESANT Transformation:23105_0| | CONTINUOUS MAIN | | CONTINUOUS | | | |-Notes:Body composition estimation by impedance measurement. Basal metabolic rate Return value in Kilo- Joules.-Variable type:continuous_raw</td></tr>
<tr><td>23106_raw.txt</td><td>1710174270056F5forCTG.txt.gz</td><td>-0.1185</td><td>0.02618</td><td>-4.526</td><td>6.01E-06</td><td>0.2483</td><td>0.01058</td><td>1.107</td><td>0.03454</td><td>-0.03174</td><td>0.01309</td><td>Impedance of whole body</td><td>FALSE</td><td>Body composition</td><td></td><td></td><td>354795</td><td></td><td></td><td>UK Biobank</td><td>https://docs.google.com/spreadsheets/d/1kvPoupSzsSFBNSztMzl04xMoSC3Kcx3CrjVf4yBmESU/edit?ts=5b5f17db#gid=227859291</td><td>PHESANT Transformation:23106_0| | CONTINUOUS MAIN | | CONTINUOUS | | | |-Notes:Body composition estimation by impedance measurement. Impedance of whole body. is Range 150 - 1200ohms in 1ohm increments.-Variable type:continuous_raw</td></tr>
<tr><td>23107_raw.txt</td><td>1710174270056F5forCTG.txt.gz</td><td>-0.06196</td><td>0.02688</td><td>-2.305</td><td>0.02114</td><td>0.2343</td><td>0.01017</td><td>1.078</td><td>0.03205</td><td>-0.03388</td><td>0.01326</td><td>Impedance of leg (right)</td><td>FALSE</td><td></td><td></td><td></td><td>354817</td><td></td><td></td><td>UK Biobank</td><td>https://docs.google.com/spreadsheets/d/1kvPoupSzsSFBNSztMzl04xMoSC3Kcx3CrjVf4yBmESU/edit?ts=5b5f17db#gid=227859291</td><td>PHESANT Transformation:23107_0| | CONTINUOUS MAIN | | CONTINUOUS | | | |-Notes:Body composition estimation by impedance measurement. Impedance of right leg, oh Measured impedance of right leg. Range 150 - 1200 (ohms) in 1 (ohms) increments.-Variable type:continuous_raw</td></tr>
<tr><td>23108_raw.txt</td><td>1710174270056F5forCTG.txt.gz</td><td>-0.06335</td><td>0.02658</td><td>-2.383</td><td>0.01718</td><td>0.2347</td><td>0.01025</td><td>1.084</td><td>0.03239</td><td>-0.03295</td><td>0.01314</td><td>Impedance of leg (left)</td><td>FALSE</td><td></td><td></td><td></td><td>354811</td><td></td><td></td><td>UK Biobank</td><td>https://docs.google.com/spreadsheets/d/1kvPoupSzsSFBNSztMzl04xMoSC3Kcx3CrjVf4yBmESU/edit?ts=5b5f17db#gid=227859291</td><td>PHESANT Transformation:23108_0| | CONTINUOUS MAIN | | CONTINUOUS | | | |-Notes:Body composition estimation by impedance measurement. Impedance of left leg, ohm Measured impedance of left leg. Range 150 - 1200 (ohms) in 1 (ohms) increments.-Variable type:continuous_raw</td></tr>
<tr><td>23109_raw.txt</td><td>1710174270056F5forCTG.txt.gz</td><td>-0.1553</td><td>0.02558</td><td>-6.069</td><td>1.29E-09</td><td>0.214</td><td>0.009169</td><td>1.104</td><td>0.03308</td><td>-0.02202</td><td>0.0122</td><td>Impedance of arm (right)</td><td>FALSE</td><td>Body composition</td><td></td><td></td><td>354792</td><td></td><td></td><td>UK Biobank</td><td>https://docs.google.com/spreadsheets/d/1kvPoupSzsSFBNSztMzl04xMoSC3Kcx3CrjVf4yBmESU/edit?ts=5b5f17db#gid=227859291</td><td>PHESANT Transformation:23109_0| | CONTINUOUS MAIN | | CONTINUOUS | | | |-Notes:Body composition estimation by impedance measurement. Impedance of right arm, oh Measured impedance of right arm. Range 150 - 1200 (ohms) in 1 (ohms) increments.-Variable type:continuous_raw</td></tr>
<tr><td>23110_raw.txt</td><td>1710174270056F5forCTG.txt.gz</td><td>-0.1623</td><td>0.0258</td><td>-6.291</td><td>3.16E-10</td><td>0.2171</td><td>0.009746</td><td>1.121</td><td>0.03459</td><td>-0.02647</td><td>0.0123</td><td>Impedance of arm (left)</td><td>FALSE</td><td>Body composition</td><td></td><td></td><td>354807</td><td></td><td></td><td>UK Biobank</td><td>https://docs.google.com/spreadsheets/d/1kvPoupSzsSFBNSztMzl04xMoSC3Kcx3CrjVf4yBmESU/edit?ts=5b5f17db#gid=227859291</td><td>PHESANT Transformation:23110_0| | CONTINUOUS MAIN | | CONTINUOUS | | | |-Notes:Body composition estimation by impedance measurement. Impedance of left arm, ohm Measured impedance of left arm. Range 150 - 1200 (ohms) in 1 (ohms) increments.-Variable type:continuous_raw</td></tr>
<tr><td>23111_raw.txt</td><td>1710174270056F5forCTG.txt.gz</td><td>0.3214</td><td>0.0228</td><td>14.09</td><td>4.11E-45</td><td>0.2197</td><td>0.008146</td><td>1.108</td><td>0.03017</td><td>0.01567</td><td>0.01252</td><td>Leg fat percentage (right)</td><td>FALSE</td><td>Body composition</td><td></td><td></td><td>354811</td><td></td><td></td><td>UK Biobank</td><td>https://docs.google.com/spreadsheets/d/1kvPoupSzsSFBNSztMzl04xMoSC3Kcx3CrjVf4yBmESU/edit?ts=5b5f17db#gid=227859291</td><td>PHESANT Transformation:23111_0| | CONTINUOUS MAIN | | CONTINUOUS | | | |-Notes:Body composition estimation by impedance measurement. Right leg fat percentages Range 1-75% in 0.1% increments.-Variable type:continuous_raw</td></tr>
<tr><td>23112_raw.txt</td><td>1710174270056F5forCTG.txt.gz</td><td>0.2936</td><td>0.0232</td><td>12.65</td><td>1.07E-36</td><td>0.2054</td><td>0.008413</td><td>1.088</td><td>0.03041</td><td>0.02835</td><td>0.01186</td><td>Leg fat mass (right)</td><td>FALSE</td><td>Body composition</td><td></td><td></td><td>354807</td><td></td><td></td><td>UK Biobank</td><td>https://docs.google.com/spreadsheets/d/1kvPoupSzsSFBNSztMzl04xMoSC3Kcx3CrjVf4yBmESU/edit?ts=5b5f17db#gid=227859291</td><td>PHESANT Transformation:23112_0| | CONTINUOUS MAIN | | CONTINUOUS | | | |-Notes:Body composition estimation by impedance measurement. Right leg fat mass Right leg fat mass in Kg, 0.1 increments.-Variable type:continuous_raw</td></tr>
<tr><td>23113_raw.txt</td><td>1710174270056F5forCTG.txt.gz</td><td>0.09966</td><td>0.02563</td><td>3.889</td><td>0.000101</td><td>0.2596</td><td>0.01272</td><td>1.094</td><td>0.04327</td><td>0.03677</td><td>0.01289</td><td>Leg fat-free mass (right)</td><td>FALSE</td><td></td><td></td><td></td><td>354798</td><td></td><td></td><td>UK Biobank</td><td>https://docs.google.com/spreadsheets/d/1kvPoupSzsSFBNSztMzl04xMoSC3Kcx3CrjVf4yBmESU/edit?ts=5b5f17db#gid=227859291</td><td>PHESANT Transformation:23113_0| | CONTINUOUS MAIN | | CONTINUOUS | | | |-Notes:Body composition estimation by impedance measurement. Right leg fat free mass. Right leg fat free mass in Kg, 0.1 increments.-Variable type:continuous_raw</td></tr>
<tr><td>23114_raw.txt</td><td>1710174270056F5forCTG.txt.gz</td><td>0.1003</td><td>0.02566</td><td>3.909</td><td>9.27E-05</td><td>0.2596</td><td>0.01274</td><td>1.095</td><td>0.04337</td><td>0.03611</td><td>0.01293</td><td>Leg predicted mass (right)</td><td>FALSE</td><td></td><td></td><td></td><td>354798</td><td></td><td></td><td>UK Biobank</td><td>https://docs.google.com/spreadsheets/d/1kvPoupSzsSFBNSztMzl04xMoSC3Kcx3CrjVf4yBmESU/edit?ts=5b5f17db#gid=227859291</td><td>PHESANT Transformation:23114_0| | CONTINUOUS MAIN | | CONTINUOUS | | | |-Notes:Body composition estimation by impedance measurement. Right leg predicted mass. in Kg, 0.1 increments.-Variable type:continuous_raw</td></tr>
<tr><td>23115_raw.txt</td><td>1710174270056F5forCTG.txt.gz</td><td>0.3184</td><td>0.02332</td><td>13.65</td><td>1.94E-42</td><td>0.2213</td><td>0.008202</td><td>1.104</td><td>0.03065</td><td>0.01748</td><td>0.01285</td><td>Leg fat percentage (left)</td><td>FALSE</td><td>Body composition</td><td></td><td></td><td>354791</td><td></td><td></td><td>UK Biobank</td><td>https://docs.google.com/spreadsheets/d/1kvPoupSzsSFBNSztMzl04xMoSC3Kcx3CrjVf4yBmESU/edit?ts=5b5f17db#gid=227859291</td><td>PHESANT Transformation:23115_0| | CONTINUOUS MAIN | | CONTINUOUS | | | |-Notes:Body composition estimation by impedance measurement. Left leg fat percentage Range 1-75% in 0.1% increments.-Variable type:continuous_raw</td></tr>
</table>

23116_raw.txt	1710174270056F5forCTG.txt.gz	0.2901	0.02342	12.38	3.18E-35	0.2067	0.008425	1.081	0.03061	0.02883	0.012	Leg fat mass (left)	FALSE	Body composition			354788			UK Biobank	https://docs.google.com/spreadsheets/d/1kvPoupSzsSFBNSztMzl04xMoSC3Kcx3CrjVf4yBmESU/edit?ts=5b5f17db#gid=227859291	PHESANT Transformation:23116_0| | CONTINUOUS MAIN | | CONTINUOUS | | | |-Notes:Body composition estimation by impedance measurement. Left leg fat mass Left leg free mass in Kg, 0.1 increments.-Variable type:continuous_raw
23117_raw.txt	1710174270056F5forCTG.txt.gz	0.1219	0.02519	4.837	1.32E-06	0.2564	0.01241	1.099	0.04188	0.03435	0.01252	Leg fat-free mass (left)	FALSE	Body composition			354771			UK Biobank	https://docs.google.com/spreadsheets/d/1kvPoupSzsSFBNSztMzl04xMoSC3Kcx3CrjVf4yBmESU/edit?ts=5b5f17db#gid=227859291	PHESANT Transformation:23117_0| | CONTINUOUS MAIN | | CONTINUOUS | | | |-Notes:Body composition estimation by impedance measurement. Left leg fat free mass. Left leg fat free mass in Kg, 0.1 increments.-Variable type:continuous_raw
23118_raw.txt	1710174270056F5forCTG.txt.gz	0.1214	0.02513	4.829	1.37E-06	0.2566	0.01238	1.097	0.04176	0.03427	0.01251	Leg predicted mass (left)	FALSE	Body composition			354766			UK Biobank	https://docs.google.com/spreadsheets/d/1kvPoupSzsSFBNSztMzl04xMoSC3Kcx3CrjVf4yBmESU/edit?ts=5b5f17db#gid=227859291	PHESANT Transformation:23118_0| | CONTINUOUS MAIN | | CONTINUOUS | | | |-Notes:Body composition estimation by impedance measurement. Left leg predicted mass. in Kg, 0.1 increments.-Variable type:continuous_raw
23119_raw.txt	1710174270056F5forCTG.txt.gz	0.2718	0.02331	11.66	2.00E-31	0.2072	0.008822	1.096	0.03186	0.02351	0.01233	Arm fat percentage (right)	FALSE	Body composition			354760			UK Biobank	https://docs.google.com/spreadsheets/d/1kvPoupSzsSFBNSztMzl04xMoSC3Kcx3CrjVf4yBmESU/edit?ts=5b5f17db#gid=227859291	PHESANT Transformation:23119_0| | CONTINUOUS MAIN | | CONTINUOUS | | | |-Notes:Body composition estimation by impedance measurement. Right arm fat percentage Range 1-75% in 0.1% increments.-Variable type:continuous_raw
23120_raw.txt	1710174270056F5forCTG.txt.gz	0.2665	0.02381	11.19	4.32E-29	0.1981	0.008554	1.07	0.03005	0.02944	0.01185	Arm fat mass (right)	FALSE	Body composition			354736			UK Biobank	https://docs.google.com/spreadsheets/d/1kvPoupSzsSFBNSztMzl04xMoSC3Kcx3CrjVf4yBmESU/edit?ts=5b5f17db#gid=227859291	PHESANT Transformation:23120_0| | CONTINUOUS MAIN | | CONTINUOUS | | | |-Notes:Body composition estimation by impedance measurement. Right arm fat mass. Right arm fat mass in Kg, 0.1 increments.-Variable type:continuous_raw
23121_raw.txt	1710174270056F5forCTG.txt.gz	0.1313	0.02449	5.359	8.35E-08	0.2576	0.01309	1.101	0.04569	0.03285	0.01221	Arm fat-free mass (right)	FALSE	Body composition			354732			UK Biobank	https://docs.google.com/spreadsheets/d/1kvPoupSzsSFBNSztMzl04xMoSC3Kcx3CrjVf4yBmESU/edit?ts=5b5f17db#gid=227859291	PHESANT Transformation:23121_0| | CONTINUOUS MAIN | | CONTINUOUS | | | |-Notes:Body composition estimation by impedance measurement. Right arm fat free mass Right arm fat free mass in Kg, 0.1 increments.-Variable type:continuous_raw
23122_raw.txt	1710174270056F5forCTG.txt.gz	0.1331	0.02454	5.426	5.77E-08	0.2572	0.01309	1.102	0.04585	0.03156	0.01224	Arm predicted mass (right)	FALSE	Body composition			354726			UK Biobank	https://docs.google.com/spreadsheets/d/1kvPoupSzsSFBNSztMzl04xMoSC3Kcx3CrjVf4yBmESU/edit?ts=5b5f17db#gid=227859291	PHESANT Transformation:23122_0| | CONTINUOUS MAIN | | CONTINUOUS | | | |-Notes:Body composition estimation by impedance measurement. Right Arm predicted mass in Kg, 0.1 increments.-Variable type:continuous_raw
23123_raw.txt	1710174270056F5forCTG.txt.gz	0.2672	0.02328	11.48	1.72E-30	0.2123	0.00894	1.091	0.03198	0.02106	0.01242	Arm fat percentage (left)	FALSE	Body composition			354707			UK Biobank	https://docs.google.com/spreadsheets/d/1kvPoupSzsSFBNSztMzl04xMoSC3Kcx3CrjVf4yBmESU/edit?ts=5b5f17db#gid=227859291	PHESANT Transformation:23123_0| | CONTINUOUS MAIN | | CONTINUOUS | | | |-Notes:Body composition estimation by impedance measurement. Left arm fat percentage Range 1-75% in 0.1% increments.-Variable type:continuous_raw
23124_raw.txt	1710174270056F5forCTG.txt.gz	0.2679	0.02375	11.28	1.65E-29	0.1954	0.008361	1.066	0.0294	0.02683	0.01175	Arm fat mass (left)	FALSE	Body composition			354673			UK Biobank	https://docs.google.com/spreadsheets/d/1kvPoupSzsSFBNSztMzl04xMoSC3Kcx3CrjVf4yBmESU/edit?ts=5b5f17db#gid=227859291	PHESANT Transformation:23124_0| | CONTINUOUS MAIN | | CONTINUOUS | | | |-Notes:Body composition estimation by impedance measurement. Left arm fat mass. Left arm fat mass in Kg, 0.1 increments.-Variable type:continuous_raw
23125_raw.txt	1710174270056F5forCTG.txt.gz	0.1464	0.02459	5.954	2.62E-09	0.2539	0.01294	1.106	0.04514	0.03413	0.01206	Arm fat-free mass (left)	FALSE	Body composition			354668			UK Biobank	https://docs.google.com/spreadsheets/d/1kvPoupSzsSFBNSztMzl04xMoSC3Kcx3CrjVf4yBmESU/edit?ts=5b5f17db#gid=227859291	PHESANT Transformation:23125_0| | CONTINUOUS MAIN | | CONTINUOUS | | | |-Notes:Body composition estimation by impedance measurement. Left arm fat free mass Left arm fat free mass in Kg, 0.1 increments.-Variable type:continuous_raw
23126_raw.txt	1710174270056F5forCTG.txt.gz	0.1464	0.02478	5.91	3.43E-09	0.2542	0.01301	1.109	0.04559	0.03325	0.0122	Arm predicted mass (left)	FALSE	Body composition			354653			UK Biobank	https://docs.google.com/spreadsheets/d/1kvPoupSzsSFBNSztMzl04xMoSC3Kcx3CrjVf4yBmESU/edit?ts=5b5f17db#gid=227859291	PHESANT Transformation:23126_0| | CONTINUOUS MAIN | | CONTINUOUS | | | |-Notes:Body composition estimation by impedance measurement. Left arm predicted mass. in Kg, 0.1 increments.-Variable type:continuous_raw
23127_raw.txt	1710174270056F5forCTG.txt.gz	0.2606	0.02247	11.6	4.20E-31	0.2071	0.008576	1.093	0.03049	0.01471	0.01191	Trunk fat percentage	FALSE	Body composition			354619			UK Biobank	https://docs.google.com/spreadsheets/d/1kvPoupSzsSFBNSztMzl04xMoSC3Kcx3CrjVf4yBmESU/edit?ts=5b5f17db#gid=227859291	PHESANT Transformation:23127_0| | CONTINUOUS MAIN | | CONTINUOUS | | | |-Notes:Body composition estimation by impedance measurement. Trunk fat percentages Range 1-75% in 0.1% increments.-Variable type:continuous_raw
23128_raw.txt	1710174270056F5forCTG.txt.gz	0.2518	0.02291	10.99	4.38E-28	0.2261	0.009112	1.092	0.03256	0.02347	0.01186	Trunk fat mass	FALSE	Body composition			354597			UK Biobank	https://docs.google.com/spreadsheets/d/1kvPoupSzsSFBNSztMzl04xMoSC3Kcx3CrjVf4yBmESU/edit?ts=5b5f17db#gid=227859291	PHESANT Transformation:23128_0| | CONTINUOUS MAIN | | CONTINUOUS | | | |-Notes:Body composition estimation by impedance measurement. Trunk fat mass Trunk fat mass in Kg, 0.1 increments.-Variable type:continuous_raw
23129_raw.txt	1710174270056F5forCTG.txt.gz	0.06432	0.02402	2.678	0.007415	0.2914	0.01669	1.127	0.05678	0.0342	0.01237	Trunk fat-free mass	FALSE				354530			UK Biobank	https://docs.google.com/spreadsheets/d/1kvPoupSzsSFBNSztMzl04xMoSC3Kcx3CrjVf4yBmESU/edit?ts=5b5f17db#gid=227859291	PHESANT Transformation:23129_0| | CONTINUOUS MAIN | | CONTINUOUS | | | |-Notes:Body composition estimation by impedance measurement. Trunk fat free mass. Trunk fat free mass in Kg, 0.1 increments.-Variable type:continuous_raw
23130_raw.txt	1710174270056F5forCTG.txt.gz	0.06562	0.02402	2.732	0.006291	0.2901	0.01661	1.124	0.05651	0.03371	0.01231	Trunk predicted mass	FALSE				354494			UK Biobank	https://docs.google.com/spreadsheets/d/1kvPoupSzsSFBNSztMzl04xMoSC3Kcx3CrjVf4yBmESU/edit?ts=5b5f17db#gid=227859291	PHESANT Transformation:23130_0| | CONTINUOUS MAIN | | CONTINUOUS | | | |-Notes:Body composition estimation by impedance measurement. Trunk predicted mass in Kg, 0.1 increments.-Variable type:continuous_raw
24003_raw.txt	1710174270056F5forCTG.txt.gz	-0.03162	0.05653	-0.5594	0.5759	0.01524	0.00181	1.033	0.01067	0.01128	0.008761	Nitrogen dioxide air pollution; 2010	FALSE				356078			UK Biobank	https://docs.google.com/spreadsheets/d/1kvPoupSzsSFBNSztMzl04xMoSC3Kcx3CrjVf4yBmESU/edit?ts=5b5f17db#gid=227859291	PHESANT Transformation:24003_0| | CONTINUOUS MAIN | | CONTINUOUS | | | |-Notes:Nitrogen dioxide; Land Use Regression (LUR) estimate for annual average 2010.-Variable type:continuous_raw
24004_raw.txt	1710174270056F5forCTG.txt.gz	0.02542	0.05937	0.4282	0.6685	0.01302	0.00172	1.023	0.009946	0.01519	0.008699	Nitrogen oxides air pollution; 2010	FALSE				356078			UK Biobank	https://docs.google.com/spreadsheets/d/1kvPoupSzsSFBNSztMzl04xMoSC3Kcx3CrjVf4yBmESU/edit?ts=5b5f17db#gid=227859291	PHESANT Transformation:24004_0| | CONTINUOUS MAIN | | CONTINUOUS | | | |-Notes:Nitrogen oxides; Land Use Regression (LUR) estimate for annual average 2010.-Variable type:continuous_raw
24005_raw.txt	1710174270056F5forCTG.txt.gz	-0.09538	0.1139	-0.8376	0.4023	0.003309	0.001791	1.008	0.01032	0.01661	0.008062	Particulate matter air pollution (pm10); 2010	FALSE				329886			UK Biobank	https://docs.google.com/spreadsheets/d/1kvPoupSzsSFBNSztMzl04xMoSC3Kcx3CrjVf4yBmESU/edit?ts=5b5f17db#gid=227859291	PHESANT Transformation:24005_0| | CONTINUOUS MAIN | | CONTINUOUS | | | |-Notes:PM10 (particulate matter with diameter less than or equal to 10 micrometres); Land Use Regression (LUR) estimate for annual average 2010.-Variable type:continuous_raw
24006_raw.txt	1710174270056F5forCTG.txt.gz	0.09849	0.0659	1.494	0.1351	0.01156	0.001873	1.036	0.01012	0.01621	0.008725	Particulate matter air pollution (pm2.5); 2010	FALSE				329886			UK Biobank	https://docs.google.com/spreadsheets/d/1kvPoupSzsSFBNSztMzl04xMoSC3Kcx3CrjVf4yBmESU/edit?ts=5b5f17db#gid=227859291	PHESANT Transformation:24006_0| | CONTINUOUS MAIN | | CONTINUOUS | | | |-Notes:PM10 (particulate matter with diameter less than or equal to 2.5 micrometres); Land Use Regression (LUR) estimate for annual average 2010.-Variable type:continuous_raw
24007_raw.txt	1710174270056F5forCTG.txt.gz	-0.1463	0.07487	-1.954	0.05074	0.008678	0.001742	1.041	0.009822	0.01519	0.008482	Particulate matter air pollution (pm2.5) absorbance; 2010	FALSE				329886			UK Biobank	https://docs.google.com/spreadsheets/d/1kvPoupSzsSFBNSztMzl04xMoSC3Kcx3CrjVf4yBmESU/edit?ts=5b5f17db#gid=227859291	PHESANT Transformation:24007_0| | CONTINUOUS MAIN | | CONTINUOUS | | | |-Notes:PM2.5 absorbance. This is ameasurement of the blackness of PM2.5 filters; a proxy for elemental carbon, which is the dominant light absorbing substanc. Land Use Regression (LUR) estimate for annual average 2010-Variable type:continuous_raw
24008_raw.txt	1710174270056F5forCTG.txt.gz	0.00642	0.1609	0.03991	0.9682	0.00183	0.001591	0.997	0.009022	0.01234	0.008406	Particulate matter air pollution 2.5-10um; 2010	FALSE				329886			UK Biobank	https://docs.google.com/spreadsheets/d/1kvPoupSzsSFBNSztMzl04xMoSC3Kcx3CrjVf4yBmESU/edit?ts=5b5f17db#gid=227859291	PHESANT Transformation:24008_0| | CONTINUOUS MAIN | | CONTINUOUS | | | |-Notes:PM coarse (particulate matter betweem 2.5 and 10 micrometres); Land Use Regression (LUR) estimate for annual average 2010.-Variable type:continuous_raw
24012_raw.txt	1710174270056F5forCTG.txt.gz	0.0647	0.1109	0.5834	0.5597	0.002486	0.001668	0.989	0.009759	0.008953	0.0077	Inverse distance to the nearest major road	FALSE				356078			UK Biobank	https://docs.google.com/spreadsheets/d/1kvPoupSzsSFBNSztMzl04xMoSC3Kcx3CrjVf4yBmESU/edit?ts=5b5f17db#gid=227859291	PHESANT Transformation:24012_0| | CONTINUOUS MAIN | | CONTINUOUS | | | |-Notes:Inverse distance to the nearest major road based upon a local road network. The definition of a major road for the local road network is a road with traffic intensity greater than 5000 motor vehicles per 24 hours. The local road network is taken from the Ordnance Survey Meridian 2 road network (scale 1:50000, 1 metre accuracy), 2009.-Variable type:continuous_raw
24016_raw.txt	1710174270056F5forCTG.txt.gz	-0.1194	0.05224	-2.285	0.0223	0.02028	0.002084	1.06	0.0116	0.00628	0.009474	Nitrogen dioxide air pollution; 2005	FALSE				356078			UK Biobank	https://docs.google.com/spreadsheets/d/1kvPoupSzsSFBNSztMzl04xMoSC3Kcx3CrjVf4yBmESU/edit?ts=5b5f17db#gid=227859291	PHESANT Transformation:24016_0| | CONTINUOUS MAIN | | CONTINUOUS | | | |-Notes:Nitrogen dioxide; Land Use Regression (LUR) estimate for annual average 2005.-Variable type:continuous_raw
24017_raw.txt	1710174270056F5forCTG.txt.gz	-0.1066	0.05232	-2.038	0.04152	0.01929	0.00208	1.052	0.01142	0.006769	0.009292	Nitrogen dioxide air pollution; 2006	FALSE				356078			UK Biobank	https://docs.google.com/spreadsheets/d/1kvPoupSzsSFBNSztMzl04xMoSC3Kcx3CrjVf4yBmESU/edit?ts=5b5f17db#gid=227859291	PHESANT Transformation:24017_0| | CONTINUOUS MAIN | | CONTINUOUS | | | |-Notes:Nitrogen dioxide; Land Use Regression (LUR) estimate for annual average 2006.-Variable type:continuous_raw
24018_raw.txt	1710174270056F5forCTG.txt.gz	-0.1353	0.05021	-2.695	0.007048	0.02192	0.00211	1.078	0.01176	0.004609	0.009532	Nitrogen dioxide air pollution; 2007	FALSE				356078			UK Biobank	https://docs.google.com/spreadsheets/d/1kvPoupSzsSFBNSztMzl04xMoSC3Kcx3CrjVf4yBmESU/edit?ts=5b5f17db#gid=227859291	PHESANT Transformation:24018_0| | CONTINUOUS MAIN | | CONTINUOUS | | | |-Notes:Nitrogen dioxide; Land Use Regression (LUR) estimate for annual average 2007.-Variable type:continuous_raw
24019_raw.txt	1710174270056F5forCTG.txt.gz	-0.1007	0.05235	-1.924	0.05431	0.01954	0.002364	1.15	0.01191	0.001512	0.009249	Particulate matter air pollution (pm10); 2007	FALSE				355245			UK Biobank	https://docs.google.com/spreadsheets/d/1kvPoupSzsSFBNSztMzl04xMoSC3Kcx3CrjVf4yBmESU/edit?ts=5b5f17db#gid=227859291	PHESANT Transformation:24019_0| | CONTINUOUS MAIN | | CONTINUOUS | | | |-Notes:PM10 (particulate matter with diameter less than or equal to 10 micrometres); Land Use Regression (LUR) estimate for annual average 2007.-Variable type:continuous_raw
24020_raw.txt	1710174270056F5forCTG.txt.gz	-0.03594	0.1179	-0.3048	0.7605	0.002784	0.001555	0.997	0.009187	0.01494	0.008479	Average daytime sound level of noise pollution	FALSE				356078			UK Biobank	https://docs.google.com/spreadsheets/d/1kvPoupSzsSFBNSztMzl04xMoSC3Kcx3CrjVf4yBmESU/edit?ts=5b5f17db#gid=227859291	PHESANT Transformation:24020_0| | CONTINUOUS MAIN | | CONTINUOUS | | | |-Notes:LDay (day equivalent level): Average sound level pressure LAeq over the 12-hour period 07:00 to 19:00.-Variable type:continuous_raw
24021_raw.txt	1710174270056F5forCTG.txt.gz	-0.03584	0.1179	-0.3039	0.7612	0.002785	0.001555	0.997	0.009186	0.01494	0.008479	Average evening sound level of noise pollution	FALSE				356078			UK Biobank	https://docs.google.com/spreadsheets/d/1kvPoupSzsSFBNSztMzl04xMoSC3Kcx3CrjVf4yBmESU/edit?ts=5b5f17db#gid=227859291	PHESANT Transformation:24021_0| | CONTINUOUS MAIN | | CONTINUOUS | | | |-Notes:LEve (evening equivalent level): Average sound level pressure LAeq between the hours of 19:00 to 23:00.-Variable type:continuous_raw
24022_raw.txt	1710174270056F5forCTG.txt.gz	-0.03593	0.1179	-0.3047	0.7606	0.002784	0.001555	0.997	0.009187	0.01494	0.00848	Average night-time sound level of noise pollution	FALSE				356078			UK Biobank	https://docs.google.com/spreadsheets/d/1kvPoupSzsSFBNSztMzl04xMoSC3Kcx3CrjVf4yBmESU/edit?ts=5b5f17db#gid=227859291	PHESANT Transformation:24022_0| | CONTINUOUS MAIN | | CONTINUOUS | | | |-Notes:LNight (night equivalent level): Average sound level pressure LAeq overnight 23:00 to 07:00.-Variable type:continuous_raw
24023_raw.txt	1710174270056F5forCTG.txt.gz	-0.03584	0.1178	-0.3041	0.761	0.002787	0.001555	0.997	0.009185	0.01494	0.008479	Average 16-hour sound level of noise pollution	FALSE				356078			UK Biobank	https://docs.google.com/spreadsheets/d/1kvPoupSzsSFBNSztMzl04xMoSC3Kcx3CrjVf4yBmESU/edit?ts=5b5f17db#gid=227859291	PHESANT Transformation:24023_0| | CONTINUOUS MAIN | | CONTINUOUS | | | |-Notes:LAeq,16hr (A-weighted equivalent sound level): Average sound level pressure LAeq between the hours of 07:00 to 23:00.-Variable type:continuous_raw
24024_raw.txt	1710174270056F5forCTG.txt.gz	-0.03578	0.1178	-0.3036	0.7614	0.002788	0.001555	0.997	0.009186	0.01494	0.008479	Average 24-hour sound level of noise pollution	FALSE				356078			UK Biobank	https://docs.google.com/spreadsheets/d/1kvPoupSzsSFBNSztMzl04xMoSC3Kcx3CrjVf4yBmESU/edit?ts=5b5f17db#gid=227859291	PHESANT Transformation:24024_0| | CONTINUOUS MAIN | | CONTINUOUS | | | |-Notes:LDen: (day-evening-night equivalent level): A-weighted Leq noise level measured over the 24 hour period with a 10 decibel penality added to the levels between 23:00 and 07:00.-Variable type:continuous_raw

<table>
<tr><td>2754_raw.txt</td><td>1710174270056F5forCTG.txt.gz</td><td>-0.4455</td><td>0.03284</td><td>-13.57</td><td>6.38E-42</td><td>0.1532</td><td>0.008195</td><td>1.058</td><td>0.01429</td><td>-0.01992</td><td>0.00986</td><td>Age at first live birth</td><td>TRUE</td><td>Reproductive</td><td></td><td>Age at first living birth</td><td>131987</td><td></td><td></td><td>UK Biobank</td><td>https://docs.google.com/spreadsheets/d/1kvPoupSzsSFBNSztMzl04xMoSC3Kcx3CrjVf4yBmESU/edit?ts=5b5f17db#gid=227859291</td><td>PHESANT Transformation:2754_0|| INTEGER || reassignments: -4=NA|-3=NA || CONTINUOUS || ||-Notes:ACE touchscreen question How old were you when you had your FIRST child? The following checks were performed: If answer If answer > 65 then rejected If answer > Participants age then rejected If answer If answer If answer > 48 then participant asked to confirm If answer > Age when periods stopped then participant asked to confirm ~F2754~ was collected from women who indicated they had given birth to more than one child, as defined by their answers to ~F2734~-Variable type:continuous_raw</td></tr>
<tr><td>2764_raw.txt</td><td>1710174270056F5forCTG.txt.gz</td><td>-0.388</td><td>0.04368</td><td>-8.883</td><td>6.49E-19</td><td>0.08289</td><td>0.006598</td><td>1.031</td><td>0.01277</td><td>-0.008904</td><td>0.009351</td><td>Age at last live birth</td><td>FALSE</td><td>Reproductive</td><td></td><td></td><td>131806</td><td></td><td></td><td>UK Biobank</td><td>https://docs.google.com/spreadsheets/d/1kvPoupSzsSFBNSztMzl04xMoSC3Kcx3CrjVf4yBmESU/edit?ts=5b5f17db#gid=227859291</td><td>PHESANT Transformation:2764_0|| INTEGER || reassignments: -4=NA|-3=NA || CONTINUOUS || ||-Notes:ACE touchscreen question How old were you when you had your LAST child? The following checks were performed: If answer If answer > 65 then rejected If answer > Participants age then rejected If answer If answer If answer > 48 then participant asked to confirm If answer > Age when periods stopped then participant asked to confirm ~F2764~ was collected from women who indicated they had given birth to more than one child, as defined by their answers to ~F2734~-Variable type:continuous_raw</td></tr>
<tr><td>2794_raw.txt</td><td>1710174270056F5forCTG.txt.gz</td><td>-0.3901</td><td>0.04695</td><td>-8.31</td><td>9.61E-17</td><td>0.04069</td><td>0.004842</td><td>1.018</td><td>0.01065</td><td>-0.002183</td><td>0.008007</td><td>Age started oral contraceptive pill</td><td>FALSE</td><td>Reproductive</td><td></td><td></td><td>154112</td><td></td><td></td><td>UK Biobank</td><td>https://docs.google.com/spreadsheets/d/1kvPoupSzsSFBNSztMzl04xMoSC3Kcx3CrjVf4yBmESU/edit?ts=5b5f17db#gid=227859291</td><td>PHESANT Transformation:2794_0|| INTEGER || reassignments: -1=NA|-3=NA || CONTINUOUS || ||-Notes:ACE touchscreen question About how old were you when you first went on the contraceptive pill? The following checks were performed: If answer If answer > Participants age then rejected If answer If answer > 50 then participant asked to confirm If the participant activated the Help button they were shown the message: If you are unsure, please provide an estimate or select Do not know. ~F2794~ was collected from women who indicated that they had taken the contraceptive pill, as defined by their answers to ~F2784~-Variable type:continuous_raw</td></tr>
<tr><td>2867_raw.txt</td><td>1710174270056F5forCTG.txt.gz</td><td>-0.4698</td><td>0.06971</td><td>-6.74</td><td>1.59E-11</td><td>0.0375</td><td>0.00796</td><td>1.009</td><td>0.01107</td><td>0.001103</td><td>0.007732</td><td>Age started smoking in former smokers</td><td>TRUE</td><td>Pulmonary</td><td></td><td>Age started smoking</td><td>88898</td><td></td><td></td><td>UK Biobank</td><td>https://docs.google.com/spreadsheets/d/1kvPoupSzsSFBNSztMzl04xMoSC3Kcx3CrjVf4yBmESU/edit?ts=5b5f17db#gid=227859291</td><td>PHESANT Transformation:2867_0|| INTEGER || reassignments: -1=NA|-3=NA || CONTINUOUS || ||-Notes:ACE touchscreen question How old were you when you first started smoking on most days? The following checks were performed: If answer If answer > Participants age then rejected If answer ~F2867~ was collected from participants who indicated that in the past they smoked on most or all days, as defined by their answers to ~F1249~-Variable type:continuous_raw</td></tr>
<tr><td>2966_raw.txt</td><td>1710174270056F5forCTG.txt.gz</td><td>-0.03483</td><td>0.04931</td><td>-0.7063</td><td>0.48</td><td>0.05152</td><td>0.008693</td><td>1.013</td><td>0.01033</td><td>0.00327</td><td>0.007041</td><td>Age high blood pressure diagnosed</td><td>FALSE</td><td></td><td></td><td></td><td>87024</td><td></td><td></td><td>UK Biobank</td><td>https://docs.google.com/spreadsheets/d/1kvPoupSzsSFBNSztMzl04xMoSC3Kcx3CrjVf4yBmESU/edit?ts=5b5f17db#gid=227859291</td><td>PHESANT Transformation:2966_0|| INTEGER || reassignments: -1=NA|-3=NA || CONTINUOUS || ||-Notes:ACE touchscreen question What was your age when the high blood pressure was first diagnosed? The following checks were performed: If answer If answer > Participants age then rejected If answer If answer > 69 then participant asked to confirm If the participant activated the Help button they were shown the message: If you are unsure, please provide an estimate or select Do not know. ~F2966~ was collected from participants who indicated they were told by a doctor that they have had high blood pressure, as defined by their answers to ~F6150~-Variable type:continuous_raw</td></tr>
<tr><td>2976_raw.txt</td><td>1710174270056F5forCTG.txt.gz</td><td>-0.03818</td><td>0.142</td><td>-0.2688</td><td>0.7881</td><td>0.04458</td><td>0.03575</td><td>1.009</td><td>0.009613</td><td>0.01555</td><td>0.008171</td><td>Age diabetes diagnosed</td><td>FALSE</td><td></td><td></td><td></td><td>16166</td><td></td><td></td><td>UK Biobank</td><td>https://docs.google.com/spreadsheets/d/1kvPoupSzsSFBNSztMzl04xMoSC3Kcx3CrjVf4yBmESU/edit?ts=5b5f17db#gid=227859291</td><td>PHESANT Transformation:2976_0|| INTEGER || reassignments: -1=NA|-3=NA || CONTINUOUS || ||-Notes:ACE touchscreen question What was your age when the diabetes was first diagnosed? The following checks were performed: If answer If answer > Participants age then rejected If answer If answer > 69 then participant asked to confirm If the participant activated the Help button they were shown the message: If you are unsure, please provide an estimate or select Do not know. ~F2976~ was collected from men who indicated that a doctor had told them they have diabetes, as defined by their answers to ~F2443~ and all women except those who indicated they had diabetes only during pregnancy, as defined by their answers to ~F2443~-Variable type:continuous_raw</td></tr>
<tr><td>30000_raw.txt</td><td>1710174270056F5forCTG.txt.gz</td><td>0.1276</td><td>0.03444</td><td>3.706</td><td>0.00021</td><td>0.1188</td><td>0.01296</td><td>1.138</td><td>0.04716</td><td>0.01044</td><td>0.01106</td><td>White blood cell (leukocyte) count</td><td>FALSE</td><td></td><td></td><td></td><td>350470</td><td></td><td></td><td>UK Biobank</td><td>https://docs.google.com/spreadsheets/d/1kvPoupSzsSFBNSztMzl04xMoSC3Kcx3CrjVf4yBmESU/edit?ts=5b5f17db#gid=227859291</td><td>PHESANT Transformation:30000_0|| CONTINUOUS MAIN || CONTINUOUS || ||-Notes:Result of White Blood Cell Count assay, performed on blood sample, obtained from UK Biobank assessment centre visit. White blood count is the number of leukocytes. Analyser operating range was 0 to 9x10^11 cells/Litre. Typical reference range is 3.5x10^9 to 9.6x10^9 cells/Litre.-Variable type:continuous_raw</td></tr>
<tr><td>30010_raw.txt</td><td>1710174270056F5forCTG.txt.gz</td><td>0.05434</td><td>0.02737</td><td>1.985</td><td>0.04713</td><td>0.1849</td><td>0.01819</td><td>1.257</td><td>0.07681</td><td>-0.002488</td><td>0.01402</td><td>Red blood cell (erythrocyte) count</td><td>FALSE</td><td></td><td></td><td></td><td>350475</td><td></td><td></td><td>UK Biobank</td><td>https://docs.google.com/spreadsheets/d/1kvPoupSzsSFBNSztMzl04xMoSC3Kcx3CrjVf4yBmESU/edit?ts=5b5f17db#gid=227859291</td><td>PHESANT Transformation:30010_0|| CONTINUOUS MAIN || CONTINUOUS || ||-Notes:Result of Red Blood Cell Count assay, performed on blood sample, obtained from UK Biobank assessment centre visit. Red blood cells is the number of erythrocytes. Analyser operating range was 0 to 2x10^13 cells/Litre. Typical reference range is 3.9x10^12 to 5.5x10^12 cells/Litre.-Variable type:continuous_raw</td></tr>
<tr><td>30020_raw.txt</td><td>1710174270056F5forCTG.txt.gz</td><td>0.05802</td><td>0.0334</td><td>1.737</td><td>0.0824</td><td>0.1225</td><td>0.0139</td><td>1.284</td><td>0.08115</td><td>0.0003314</td><td>0.01293</td><td>Haemoglobin concentration</td><td>FALSE</td><td></td><td></td><td></td><td>350474</td><td></td><td></td><td>UK Biobank</td><td>https://docs.google.com/spreadsheets/d/1kvPoupSzsSFBNSztMzl04xMoSC3Kcx3CrjVf4yBmESU/edit?ts=5b5f17db#gid=227859291</td><td>PHESANT Transformation:30020_0|| CONTINUOUS MAIN || CONTINUOUS || ||-Notes:Result of Haemoglobin Concentration assay, performed on blood sample, obtained from UK Biobank assessment centre visit. Analyser operating range was 0 to 99.9g/dL Typical reference range is 12.1 to 16.3g/dL-Variable type:continuous_raw</td></tr>
<tr><td>30030_raw.txt</td><td>1710174270056F5forCTG.txt.gz</td><td>0.05287</td><td>0.03355</td><td>1.576</td><td>0.1151</td><td>0.1191</td><td>0.01379</td><td>1.261</td><td>0.07722</td><td>0.002102</td><td>0.01321</td><td>Haematocrit percentage</td><td>FALSE</td><td></td><td></td><td></td><td>350475</td><td></td><td></td><td>UK Biobank</td><td>https://docs.google.com/spreadsheets/d/1kvPoupSzsSFBNSztMzl04xMoSC3Kcx3CrjVf4yBmESU/edit?ts=5b5f17db#gid=227859291</td><td>PHESANT Transformation:30030_0|| CONTINUOUS MAIN || CONTINUOUS || ||-Notes:Result of Haematocrit assay, performed on blood sample, obtained from UK Biobank assessment centre visit. Haematocrit Percentage is the relative volume of packed erythrocytes to whole blood, computed by the formula: (red blood cells x mean corpuscular volume) / 10. Analyser operating range was 0 to 99.9% Typical reference range is 35.4 to 47.2%-Variable type:continuous_raw</td></tr>
<tr><td>30040_raw.txt</td><td>1710174270056F5forCTG.txt.gz</td><td>-0.02386</td><td>0.02402</td><td>-0.9934</td><td>0.3205</td><td>0.1896</td><td>0.02111</td><td>1.246</td><td>0.1222</td><td>0.00644</td><td>0.01359</td><td>Mean corpuscular volume</td><td>FALSE</td><td></td><td></td><td></td><td>350473</td><td></td><td></td><td>UK Biobank</td><td>https://docs.google.com/spreadsheets/d/1kvPoupSzsSFBNSztMzl04xMoSC3Kcx3CrjVf4yBmESU/edit?ts=5b5f17db#gid=227859291</td><td>PHESANT Transformation:30040_0|| CONTINUOUS MAIN || CONTINUOUS || ||-Notes:Result of Mean Corpuscular Volume assay, performed on blood sample, obtained from UK Biobank assessment centre visit. Mean Corpuscular Haemoglobin Concentration (pg) is the weight of hemoglobin in the average erythrocyte, computed by the formula: MCH = (hemoglobin/red blood cells) x 10.-Variable type:continuous_raw</td></tr>
<tr><td>30050_raw.txt</td><td>1710174270056F5forCTG.txt.gz</td><td>-0.01803</td><td>0.0244</td><td>-0.7387</td><td>0.4601</td><td>0.1649</td><td>0.01998</td><td>1.166</td><td>0.1008</td><td>0.003454</td><td>0.0132</td><td>Mean corpuscular haemoglobin</td><td>FALSE</td><td></td><td></td><td></td><td>350472</td><td></td><td></td><td>UK Biobank</td><td>https://docs.google.com/spreadsheets/d/1kvPoupSzsSFBNSztMzl04xMoSC3Kcx3CrjVf4yBmESU/edit?ts=5b5f17db#gid=227859291</td><td>PHESANT Transformation:30050_0|| CONTINUOUS MAIN || CONTINUOUS || ||-Notes:Result of Mean Corpuscular Haemoglobin assay, performed on blood sample, obtained from UK Biobank assessment centre visit.-Variable type:continuous_raw</td></tr>
<tr><td>30060_raw.txt</td><td>1710174270056F5forCTG.txt.gz</td><td>0.01605</td><td>0.03587</td><td>0.4474</td><td>0.6546</td><td>0.04099</td><td>0.006195</td><td>1.032</td><td>0.0266</td><td>-0.003596</td><td>0.009467</td><td>Mean corpuscular haemoglobin concentration</td><td>FALSE</td><td></td><td></td><td></td><td>350468</td><td></td><td></td><td>UK Biobank</td><td>https://docs.google.com/spreadsheets/d/1kvPoupSzsSFBNSztMzl04xMoSC3Kcx3CrjVf4yBmESU/edit?ts=5b5f17db#gid=227859291</td><td>PHESANT Transformation:30060_0|| CONTINUOUS MAIN || CONTINUOUS || ||-Notes:Result of Mean Corpuscular Haemoglobin Concentration assay, performed on blood sample, obtained from UK Biobank assessment centre visit. Mean Corpuscular Haemoglobin Concentration (g/dL) is the average weight of haemoglobin in a measured dilution, computed by the formula: (haemoglobin/haematocrit) x 100.-Variable type:continuous_raw</td></tr>
<tr><td>30070_raw.txt</td><td>1710174270056F5forCTG.txt.gz</td><td>0.05265</td><td>0.02742</td><td>1.92</td><td>0.05486</td><td>0.1235</td><td>0.0144</td><td>1.059</td><td>0.06261</td><td>0.001029</td><td>0.01071</td><td>Red blood cell (erythrocyte) distribution width</td><td>FALSE</td><td></td><td></td><td></td><td>350473</td><td></td><td></td><td>UK Biobank</td><td>https://docs.google.com/spreadsheets/d/1kvPoupSzsSFBNSztMzl04xMoSC3Kcx3CrjVf4yBmESU/edit?ts=5b5f17db#gid=227859291</td><td>PHESANT Transformation:30070_0|| CONTINUOUS MAIN || CONTINUOUS || ||-Notes:Result of Red Distribution Width assay, performed on blood sample, obtained from UK Biobank assessment centre visit. Red blood cell Distribution Width is the size distribution spread of the erythrocyte population derived from the red blood cell histogram. It is the coefficient of variation (CV) expressed in % of the red blood cell size distribution.-Variable type:continuous_raw</td></tr>
<tr><td>30080_raw.txt</td><td>1710174270056F5forCTG.txt.gz</td><td>0.03244</td><td>0.02745</td><td>1.182</td><td>0.2373</td><td>0.2168</td><td>0.02011</td><td>1.293</td><td>0.1118</td><td>-0.000433</td><td>0.01333</td><td>Platelet count</td><td>FALSE</td><td></td><td></td><td></td><td>350474</td><td></td><td></td><td>UK Biobank</td><td>https://docs.google.com/spreadsheets/d/1kvPoupSzsSFBNSztMzl04xMoSC3Kcx3CrjVf4yBmESU/edit?ts=5b5f17db#gid=227859291</td><td>PHESANT Transformation:30080_0|| CONTINUOUS MAIN || CONTINUOUS || ||-Notes:Result of Platelet Count assay, performed on blood sample, obtained from UK Biobank assessment centre visit. Platelet count is the number of thrombocytes derived from the platelet histogram.-Variable type:continuous_raw</td></tr>
<tr><td>30090_raw.txt</td><td>1710174270056F5forCTG.txt.gz</td><td>0.04908</td><td>0.02899</td><td>1.693</td><td>0.09044</td><td>0.1749</td><td>0.01544</td><td>1.287</td><td>0.08409</td><td>0.002315</td><td>0.01293</td><td>Platelet crit</td><td>FALSE</td><td></td><td></td><td></td><td>350471</td><td></td><td></td><td>UK Biobank</td><td>https://docs.google.com/spreadsheets/d/1kvPoupSzsSFBNSztMzl04xMoSC3Kcx3CrjVf4yBmESU/edit?ts=5b5f17db#gid=227859291</td><td>PHESANT Transformation:30090_0|| CONTINUOUS MAIN || CONTINUOUS || ||-Notes:Result of Platelet Crit assay, performed on blood sample, obtained from UK Biobank assessment centre visit. Platelet Crit is a computed value that represents the platelet packed cell volume.-Variable type:continuous_raw</td></tr>
<tr><td>30100_raw.txt</td><td>1710174270056F5forCTG.txt.gz</td><td>0.004406</td><td>0.02577</td><td>0.171</td><td>0.8642</td><td>0.2602</td><td>0.03585</td><td>1.306</td><td>0.1406</td><td>0.009448</td><td>0.01308</td><td>Mean platelet (thrombocyte) volume</td><td>FALSE</td><td></td><td></td><td></td><td>350470</td><td></td><td></td><td>UK Biobank</td><td>https://docs.google.com/spreadsheets/d/1kvPoupSzsSFBNSztMzl04xMoSC3Kcx3CrjVf4yBmESU/edit?ts=5b5f17db#gid=227859291</td><td>PHESANT Transformation:30100_0|| CONTINUOUS MAIN || CONTINUOUS || ||-Notes:Result of Mean Platelet Volume assay, performed on blood sample, obtained from UK Biobank assessment centre visit. Mean Platelet Volume is the average volume of individual platelets derived from the platelet histogram.-Variable type:continuous_raw</td></tr>
<tr><td>30110_raw.txt</td><td>1710174270056F5forCTG.txt.gz</td><td>-0.003867</td><td>0.02915</td><td>-0.1327</td><td>0.8945</td><td>0.1921</td><td>0.02614</td><td>1.141</td><td>0.09696</td><td>0.004113</td><td>0.01374</td><td>Platelet distribution width</td><td>FALSE</td><td></td><td></td><td></td><td>350470</td><td></td><td></td><td>UK Biobank</td><td>https://docs.google.com/spreadsheets/d/1kvPoupSzsSFBNSztMzl04xMoSC3Kcx3CrjVf4yBmESU/edit?ts=5b5f17db#gid=227859291</td><td>PHESANT Transformation:30110_0|| CONTINUOUS MAIN || CONTINUOUS || ||-Notes:Result of Platelet Distribution Width assay, performed on blood sample, obtained from UK Biobank assessment centre visit. Platelet Distribution Width is the coefficient of variation of platelet size.-Variable type:continuous_raw</td></tr>
<tr><td>30120_raw.txt</td><td>1710174270056F5forCTG.txt.gz</td><td>0.1093</td><td>0.03498</td><td>3.124</td><td>0.001785</td><td>0.05149</td><td>0.004977</td><td>1.041</td><td>0.02239</td><td>0.0007118</td><td>0.00887</td><td>Lymphocyte count</td><td>FALSE</td><td></td><td></td><td></td><td>349856</td><td></td><td></td><td>UK Biobank</td><td>https://docs.google.com/spreadsheets/d/1kvPoupSzsSFBNSztMzl04xMoSC3Kcx3CrjVf4yBmESU/edit?ts=5b5f17db#gid=227859291</td><td>PHESANT Transformation:30120_0|| CONTINUOUS MAIN || CONTINUOUS || ||-Notes:Result of Lymphocytes Number assay, performed on blood sample, obtained from UK Biobank assessment centre visit. Lymphocyte count is the proportion of (lymphoctyes / 100) x white blood cell count-Variable type:continuous_raw</td></tr>
</table>

<table>
<tr><td>30130_raw.txt</td><td>1710174270056F5forCTG.txt.gz</td><td>0.08695</td><td>0.03227</td><td>2.695</td><td>0.007041</td><td>0.09026</td><td>0.01346</td><td>1.195</td><td>0.06805</td><td>-0.004461</td><td>0.01075</td><td>Monocyte count</td><td>FALSE</td><td></td><td></td><td></td><td>349856</td><td></td><td></td><td>UK Biobank</td><td>https://docs.google.com/spreadsheets/d/1kvPoupSzsSFBNSztMzl04xMoSC3Kcx3CrjVf4yBmESU/edit?ts=5b5f17db#gid=227859291</td><td>PHESANT Transformation:30130_0|| CONTINUOUS MAIN || CONTINUOUS || ||-Notes:Result of Monocytes Number assay, performed on blood sample, obtained from UK Biobank assessment centre visit. Monocyte count is the proportion of (monoctyes / 100) x white blood cell count.-Variable type:continuous_raw</td></tr>
<tr><td>30140_raw.txt</td><td>1710174270056F5forCTG.txt.gz</td><td>0.1139</td><td>0.03296</td><td>3.457</td><td>0.000546</td><td>0.1251</td><td>0.01342</td><td>1.18</td><td>0.05251</td><td>0.01278</td><td>0.01138</td><td>Neutrophill count</td><td>FALSE</td><td></td><td></td><td></td><td>349856</td><td></td><td></td><td>UK Biobank</td><td>https://docs.google.com/spreadsheets/d/1kvPoupSzsSFBNSztMzl04xMoSC3Kcx3CrjVf4yBmESU/edit?ts=5b5f17db#gid=227859291</td><td>PHESANT Transformation:30140_0|| CONTINUOUS MAIN || CONTINUOUS || ||-Notes:Result of Neutrophils Number assay, performed on blood sample, obtained from UK Biobank assessment centre visit. Neutrophils count is the proportion of (neutrophils / 100) x white blood cell count.-Variable type:continuous_raw</td></tr>
<tr><td>30180_raw.txt</td><td>1710174270056F5forCTG.txt.gz</td><td>0.02326</td><td>0.02621</td><td>0.8875</td><td>0.3748</td><td>0.1363</td><td>0.01325</td><td>1.136</td><td>0.04875</td><td>-0.001445</td><td>0.01056</td><td>Lymphocyte percentage</td><td>FALSE</td><td></td><td></td><td></td><td>349861</td><td></td><td></td><td>UK Biobank</td><td>https://docs.google.com/spreadsheets/d/1kvPoupSzsSFBNSztMzl04xMoSC3Kcx3CrjVf4yBmESU/edit?ts=5b5f17db#gid=227859291</td><td>PHESANT Transformation:30180_0|| CONTINUOUS MAIN || CONTINUOUS || ||-Notes:Result of Lymphocytes Percentage assay, performed on blood sample, obtained from UK Biobank assessment centre visit. Lymphocytes (Percentage) is calculated as the proportion of lymphocytes in the leukocytes.-Variable type:continuous_raw</td></tr>
<tr><td>30190_raw.txt</td><td>1710174270056F5forCTG.txt.gz</td><td>-0.004535</td><td>0.03304</td><td>-0.1373</td><td>0.8908</td><td>0.08698</td><td>0.0143</td><td>1.203</td><td>0.07347</td><td>-0.01373</td><td>0.01117</td><td>Monocyte percentage</td><td>FALSE</td><td></td><td></td><td></td><td>349861</td><td></td><td></td><td>UK Biobank</td><td>https://docs.google.com/spreadsheets/d/1kvPoupSzsSFBNSztMzl04xMoSC3Kcx3CrjVf4yBmESU/edit?ts=5b5f17db#gid=227859291</td><td>PHESANT Transformation:30190_0|| CONTINUOUS MAIN || CONTINUOUS || ||-Notes:Result of Monocytes Percentage assay, performed on blood sample, obtained from UK Biobank assessment centre visit. Monocytes (percentage) is calculated as the proportion of monocytes in the leukocytes.-Variable type:continuous_raw</td></tr>
<tr><td>30200_raw.txt</td><td>1710174270056F5forCTG.txt.gz</td><td>-0.007882</td><td>0.02768</td><td>-0.2848</td><td>0.7758</td><td>0.1258</td><td>0.01371</td><td>1.114</td><td>0.04929</td><td>0.00196</td><td>0.01057</td><td>Neutrophill percentage</td><td>FALSE</td><td></td><td></td><td></td><td>349861</td><td></td><td></td><td>UK Biobank</td><td>https://docs.google.com/spreadsheets/d/1kvPoupSzsSFBNSztMzl04xMoSC3Kcx3CrjVf4yBmESU/edit?ts=5b5f17db#gid=227859291</td><td>PHESANT Transformation:30200_0|| CONTINUOUS MAIN || CONTINUOUS || ||-Notes:Result of Neutrophils Percentage assay, performed on blood sample, obtained from UK Biobank assessment centre visit. Neutrophils (Percentage) is calculated as the proportion of neutrophils in the leukocytes.-Variable type:continuous_raw</td></tr>
<tr><td>30210_raw.txt</td><td>1710174270056F5forCTG.txt.gz</td><td>-0.04802</td><td>0.0287</td><td>-1.673</td><td>0.09438</td><td>0.1466</td><td>0.02209</td><td>1.079</td><td>0.07431</td><td>0.01499</td><td>0.01233</td><td>Eosinophill percentage</td><td>FALSE</td><td></td><td></td><td></td><td>349861</td><td></td><td></td><td>UK Biobank</td><td>https://docs.google.com/spreadsheets/d/1kvPoupSzsSFBNSztMzl04xMoSC3Kcx3CrjVf4yBmESU/edit?ts=5b5f17db#gid=227859291</td><td>PHESANT Transformation:30210_0|| CONTINUOUS MAIN || CONTINUOUS || ||-Notes:Result of Eosinophils Percentage assay, performed on blood sample, obtained from UK Biobank assessment centre visit. Eosinophils (Percentage) is calculated as the proportion of eosinophils in the leukocytes.-Variable type:continuous_raw</td></tr>
<tr><td>30220_raw.txt</td><td>1710174270056F5forCTG.txt.gz</td><td>-0.01195</td><td>0.03808</td><td>-0.3139</td><td>0.7536</td><td>0.03023</td><td>0.005148</td><td>1.035</td><td>0.02426</td><td>0.005053</td><td>0.008178</td><td>Basophill percentage</td><td>FALSE</td><td></td><td></td><td></td><td>349861</td><td></td><td></td><td>UK Biobank</td><td>https://docs.google.com/spreadsheets/d/1kvPoupSzsSFBNSztMzl04xMoSC3Kcx3CrjVf4yBmESU/edit?ts=5b5f17db#gid=227859291</td><td>PHESANT Transformation:30220_0|| CONTINUOUS MAIN || CONTINUOUS || ||-Notes:Result of Basophils Percentage assay, performed on blood sample, obtained from UK Biobank assessment centre visit. Basophils (Percentage) is calculated as the proportion of basophils in the leukocytes.-Variable type:continuous_raw</td></tr>
<tr><td>30240_raw.txt</td><td>1710174270056F5forCTG.txt.gz</td><td>0.1336</td><td>0.03735</td><td>3.576</td><td>0.000349</td><td>0.05414</td><td>0.007858</td><td>1.076</td><td>0.04429</td><td>0.0102</td><td>0.009353</td><td>Reticulocyte percentage</td><td>FALSE</td><td></td><td></td><td></td><td>344728</td><td></td><td></td><td>UK Biobank</td><td>https://docs.google.com/spreadsheets/d/1kvPoupSzsSFBNSztMzl04xMoSC3Kcx3CrjVf4yBmESU/edit?ts=5b5f17db#gid=227859291</td><td>PHESANT Transformation:30240_0|| CONTINUOUS MAIN || CONTINUOUS || ||-Notes:Result of Reticulocytes Percentage assay, performed on blood sample, obtained from UK Biobank assessment centre visit. Reticulocytes (Percentage) is the number of reticulocytes as a percentage of red blood cells.-Variable type:continuous_raw</td></tr>
<tr><td>30250_raw.txt</td><td>1710174270056F5forCTG.txt.gz</td><td>0.1342</td><td>0.03658</td><td>3.669</td><td>0.000243</td><td>0.06404</td><td>0.008797</td><td>1.087</td><td>0.04933</td><td>0.01117</td><td>0.009854</td><td>Reticulocyte count</td><td>FALSE</td><td></td><td></td><td></td><td>344729</td><td></td><td></td><td>UK Biobank</td><td>https://docs.google.com/spreadsheets/d/1kvPoupSzsSFBNSztMzl04xMoSC3Kcx3CrjVf4yBmESU/edit?ts=5b5f17db#gid=227859291</td><td>PHESANT Transformation:30250_0|| CONTINUOUS MAIN || CONTINUOUS || ||-Notes:Result of Reticulocytes Number assay, performed on blood sample, obtained from UK Biobank assessment centre visit. Reticulocytes count is computed from the reticulocyte per cent multiplied by the red blood cell count.-Variable type:continuous_raw</td></tr>
<tr><td>30260_raw.txt</td><td>1710174270056F5forCTG.txt.gz</td><td>0.02244</td><td>0.02527</td><td>0.888</td><td>0.3745</td><td>0.1563</td><td>0.01934</td><td>1.23</td><td>0.1043</td><td>-0.002126</td><td>0.01306</td><td>Mean reticulocyte volume</td><td>FALSE</td><td></td><td></td><td></td><td>344728</td><td></td><td></td><td>UK Biobank</td><td>https://docs.google.com/spreadsheets/d/1kvPoupSzsSFBNSztMzl04xMoSC3Kcx3CrjVf4yBmESU/edit?ts=5b5f17db#gid=227859291</td><td>PHESANT Transformation:30260_0|| CONTINUOUS MAIN || CONTINUOUS || ||-Notes:Result of Mean Reticulocytes Volume assay, performed on blood sample, obtained from UK Biobank assessment centre visit. Mean Reticulocytes volume is the average volume of all reticulocytes, calculated from the reticulocytes (percentage).-Variable type:continuous_raw</td></tr>
<tr><td>30270_raw.txt</td><td>1710174270056F5forCTG.txt.gz</td><td>-0.01392</td><td>0.0252</td><td>-0.5526</td><td>0.5806</td><td>0.1643</td><td>0.01918</td><td>1.251</td><td>0.09845</td><td>0.002575</td><td>0.01265</td><td>Mean sphered cell volume</td><td>FALSE</td><td></td><td></td><td></td><td>344729</td><td></td><td></td><td>UK Biobank</td><td>https://docs.google.com/spreadsheets/d/1kvPoupSzsSFBNSztMzl04xMoSC3Kcx3CrjVf4yBmESU/edit?ts=5b5f17db#gid=227859291</td><td>PHESANT Transformation:30270_0|| CONTINUOUS MAIN || CONTINUOUS || ||-Notes:Result of Mean Sphered Cells Volume assay, performed on blood sample, obtained from UK Biobank assessment centre visit.-Variable type:continuous_raw</td></tr>
<tr><td>30280_raw.txt</td><td>1710174270056F5forCTG.txt.gz</td><td>0.1626</td><td>0.03253</td><td>5</td><td>5.74E-07</td><td>0.1015</td><td>0.01566</td><td>1.258</td><td>0.1066</td><td>0.01436</td><td>0.01226</td><td>Immature reticulocyte fraction</td><td>FALSE</td><td>Ophthalmic</td><td></td><td></td><td>344728</td><td></td><td></td><td>UK Biobank</td><td>https://docs.google.com/spreadsheets/d/1kvPoupSzsSFBNSztMzl04xMoSC3Kcx3CrjVf4yBmESU/edit?ts=5b5f17db#gid=227859291</td><td>PHESANT Transformation:30280_0|| CONTINUOUS MAIN || CONTINUOUS || ||-Notes:Result of Immature Reticulocytes Fraction assay, performed on blood sample, obtained from UK Biobank assessment centre visit. Immature Reticulocytes Fraction (Percentage) is an indication of new reticulocyte synthesis and is calculated from the reticulocytes (Percentage) as the total number of reticulocyte events in the outermost light scattering region, corresponding to immature reticulocytes, relative to the total number of reticulocytes and is reported as this ratio.-Variable type:continuous_raw</td></tr>
<tr><td>30290_raw.txt</td><td>1710174270056F5forCTG.txt.gz</td><td>0.168</td><td>0.03557</td><td>4.724</td><td>2.32E-06</td><td>0.05882</td><td>0.008117</td><td>1.106</td><td>0.05116</td><td>-0.000174</td><td>0.01012</td><td>High light scatter reticulocyte percentage</td><td>FALSE</td><td>Ophthalmic</td><td></td><td></td><td>344729</td><td></td><td></td><td>UK Biobank</td><td>https://docs.google.com/spreadsheets/d/1kvPoupSzsSFBNSztMzl04xMoSC3Kcx3CrjVf4yBmESU/edit?ts=5b5f17db#gid=227859291</td><td>PHESANT Transformation:30290_0|| CONTINUOUS MAIN || CONTINUOUS || ||-Notes:Result of High Light Scatter Reticulocytes Percentage assay, performed on blood sample, obtained from UK Biobank assessment centre visit. High Light Scatter Reticulocytes (Percentage) is computed as: (HLR / Total Erythrocytes) x 100.-Variable type:continuous_raw</td></tr>
<tr><td>30300_raw.txt</td><td>1710174270056F5forCTG.txt.gz</td><td>0.1624</td><td>0.03106</td><td>5.227</td><td>1.72E-07</td><td>0.1335</td><td>0.0161</td><td>1.228</td><td>0.09472</td><td>0.01426</td><td>0.01237</td><td>High light scatter reticulocyte count</td><td>FALSE</td><td>Ophthalmic</td><td></td><td></td><td>344729</td><td></td><td></td><td>UK Biobank</td><td>https://docs.google.com/spreadsheets/d/1kvPoupSzsSFBNSztMzl04xMoSC3Kcx3CrjVf4yBmESU/edit?ts=5b5f17db#gid=227859291</td><td>PHESANT Transformation:30300_0|| CONTINUOUS MAIN || CONTINUOUS || ||-Notes:Result of High Light Scatter Reticulocytes Number assay, performed on blood sample, obtained from UK Biobank assessment centre visit. High Light Scatter Reticulocytes Count is calculated as: (HLR / Total Erythrocytes) x red blood cells-Variable type:continuous_raw</td></tr>
<tr><td>30500_raw.txt</td><td>1710174270056F5forCTG.txt.gz</td><td>0.1547</td><td>0.1132</td><td>1.367</td><td>0.1717</td><td>0.01159</td><td>0.005266</td><td>0.996</td><td>0.009349</td><td>-0.003163</td><td>0.008315</td><td>Microalbumin in urine</td><td>FALSE</td><td></td><td></td><td></td><td>108706</td><td></td><td></td><td>UK Biobank</td><td>https://docs.google.com/spreadsheets/d/1kvPoupSzsSFBNSztMzl04xMoSC3Kcx3CrjVf4yBmESU/edit?ts=5b5f17db#gid=227859291</td><td>PHESANT Transformation:30500_0|| CONTINUOUS MAIN || CONTINUOUS || ||-Notes:Measured by immunoturbidimetric analysis on a Beckman Coulter AU5400.-Variable type:continuous_raw</td></tr>
<tr><td>30520_raw.txt</td><td>1710174270056F5forCTG.txt.gz</td><td>0.01937</td><td>0.03801</td><td>0.5096</td><td>0.6104</td><td>0.03975</td><td>0.00262</td><td>1.007</td><td>0.01244</td><td>-0.00072</td><td>0.009233</td><td>Potassium in urine</td><td>FALSE</td><td></td><td></td><td></td><td>350053</td><td></td><td></td><td>UK Biobank</td><td>https://docs.google.com/spreadsheets/d/1kvPoupSzsSFBNSztMzl04xMoSC3Kcx3CrjVf4yBmESU/edit?ts=5b5f17db#gid=227859291</td><td>PHESANT Transformation:30520_0|| CONTINUOUS MAIN || CONTINUOUS || ||-Notes:Measured by ISE (ion selective electrode) analysis on a Beckman Coulter AU5400.-Variable type:continuous_raw</td></tr>
<tr><td>30530_raw.txt</td><td>1710174270056F5forCTG.txt.gz</td><td>0.1337</td><td>0.03225</td><td>4.144</td><td>3.41E-05</td><td>0.07083</td><td>0.003729</td><td>1.01</td><td>0.01616</td><td>0.008053</td><td>0.009917</td><td>Sodium in urine</td><td>FALSE</td><td></td><td></td><td></td><td>350061</td><td></td><td></td><td>UK Biobank</td><td>https://docs.google.com/spreadsheets/d/1kvPoupSzsSFBNSztMzl04xMoSC3Kcx3CrjVf4yBmESU/edit?ts=5b5f17db#gid=227859291</td><td>PHESANT Transformation:30530_0|| CONTINUOUS MAIN || CONTINUOUS || ||-Notes:Measured by ISE (ion selective electrode) analysis using Beckman Coulter AU5400.-Variable type:continuous_raw</td></tr>
<tr><td>3062_raw.txt</td><td>1710174270056F5forCTG.txt.gz</td><td>-0.1376</td><td>0.02466</td><td>-5.578</td><td>2.43E-08</td><td>0.1932</td><td>0.00954</td><td>1.06</td><td>0.02981</td><td>-0.01907</td><td>0.0107</td><td>Forced vital capacity (FVC)</td><td>FALSE</td><td>Pulmonary</td><td></td><td></td><td>329404</td><td></td><td></td><td>UK Biobank</td><td>https://docs.google.com/spreadsheets/d/1kvPoupSzsSFBNSztMzl04xMoSC3Kcx3CrjVf4yBmESU/edit?ts=5b5f17db#gid=227859291</td><td>PHESANT Transformation:3062_0|| CONTINUOUS MAIN || CONTINUOUS || ||-Notes:FVC value calculated from blow-Variable type:continuous_raw</td></tr>
<tr><td>3063_raw.txt</td><td>1710174270056F5forCTG.txt.gz</td><td>-0.1328</td><td>0.02541</td><td>-5.227</td><td>1.72E-07</td><td>0.1907</td><td>0.009099</td><td>1.064</td><td>0.02841</td><td>-0.0161</td><td>0.01087</td><td>Forced expiratory volume in 1-second (FEV1)</td><td>FALSE</td><td>Pulmonary</td><td></td><td></td><td>329404</td><td></td><td></td><td>UK Biobank</td><td>https://docs.google.com/spreadsheets/d/1kvPoupSzsSFBNSztMzl04xMoSC3Kcx3CrjVf4yBmESU/edit?ts=5b5f17db#gid=227859291</td><td>PHESANT Transformation:3063_0|| CONTINUOUS MAIN || CONTINUOUS || ||-Notes:FEV1 value calculated from blow-Variable type:continuous_raw</td></tr>
<tr><td>3064_raw.txt</td><td>1710174270056F5forCTG.txt.gz</td><td>-0.0513</td><td>0.02771</td><td>-1.851</td><td>0.0641</td><td>0.1062</td><td>0.006612</td><td>1.051</td><td>0.02365</td><td>-0.002381</td><td>0.01038</td><td>Peak expiratory flow (PEF)</td><td>FALSE</td><td></td><td></td><td></td><td>329404</td><td></td><td></td><td>UK Biobank</td><td>https://docs.google.com/spreadsheets/d/1kvPoupSzsSFBNSztMzl04xMoSC3Kcx3CrjVf4yBmESU/edit?ts=5b5f17db#gid=227859291</td><td>PHESANT Transformation:3064_0|| INTEGER || CONTINUOUS || ||-Notes:Peak expiratory flow during blow-Variable type:continuous_raw</td></tr>
<tr><td>30890_irnt.gwas.imputed_v3.both_sexes.tsv.bgz.ctg.gz</td><td>1710174270056F5forCTG.txt.gz</td><td>-0.002452</td><td>0.02827</td><td>-0.08674</td><td>0.9309</td><td>0.08736</td><td>0.02027</td><td>1.023</td><td>0.05599</td><td>-0.02227</td><td>0.01076</td><td>Vitamin D (quantile) both_sexes</td><td>FALSE</td><td></td><td></td><td></td><td>400000</td><td></td><td></td><td>UK Biobank</td><td>http://www.nealelab.is/uk-biobank/</td><td></td></tr>
<tr><td>3143_raw.txt</td><td>1710174270056F5forCTG.txt.gz</td><td>0.1031</td><td>0.02431</td><td>4.241</td><td>2.23E-05</td><td>0.2996</td><td>0.01965</td><td>1.075</td><td>0.03619</td><td>0.02498</td><td>0.01017</td><td>Ankle spacing width</td><td>FALSE</td><td>Skeletal</td><td></td><td></td><td>206589</td><td></td><td></td><td>UK Biobank</td><td>https://docs.google.com/spreadsheets/d/1kvPoupSzsSFBNSztMzl04xMoSC3Kcx3CrjVf4yBmESU/edit?ts=5b5f17db#gid=227859291</td><td>PHESANT Transformation:3143_0|| CONTINUOUS MAIN || CONTINUOUS || ||-Notes:Ankle width as indicated by the spacing between measurement transducer pads on heel-Variable type:continuous_raw</td></tr>
<tr><td>3144_raw.txt</td><td>1710174270056F5forCTG.txt.gz</td><td>0.08638</td><td>0.02536</td><td>3.406</td><td>0.000658</td><td>0.2437</td><td>0.02517</td><td>1.038</td><td>0.04974</td><td>-0.005981</td><td>0.01085</td><td>Heel Broadband ultrasound attenuation, direct entry</td><td>FALSE</td><td></td><td></td><td></td><td>206576</td><td></td><td></td><td>UK Biobank</td><td>https://docs.google.com/spreadsheets/d/1kvPoupSzsSFBNSztMzl04xMoSC3Kcx3CrjVf4yBmESU/edit?ts=5b5f17db#gid=227859291</td><td>PHESANT Transformation:3144_0|| CONTINUOUS MAIN || CONTINUOUS || ||-Notes:Broadband ultrasound attenuation through heel-Variable type:continuous_raw</td></tr>
<tr><td>3147_raw.txt</td><td>1710174270056F5forCTG.txt.gz</td><td>0.08411</td><td>0.02487</td><td>3.382</td><td>0.000719</td><td>0.2576</td><td>0.02689</td><td>1.065</td><td>0.05531</td><td>-0.01413</td><td>0.0108</td><td>Heel quantitative ultrasound index (QUI), direct entry</td><td>FALSE</td><td></td><td></td><td></td><td>206589</td><td></td><td></td><td>UK Biobank</td><td>https://docs.google.com/spreadsheets/d/1kvPoupSzsSFBNSztMzl04xMoSC3Kcx3CrjVf4yBmESU/edit?ts=5b5f17db#gid=227859291</td><td>PHESANT Transformation:3147_0|| CONTINUOUS MAIN || CONTINUOUS || ||-Notes:The QUI or 'stiffness' is based on measure of the speed of sound and broadband ultrasound attenuation. Direct entry.-Variable type:continuous_raw</td></tr>
<tr><td>3148_raw.txt</td><td>1710174270056F5forCTG.txt.gz</td><td>0.08248</td><td>0.02463</td><td>3.349</td><td>0.000811</td><td>0.2607</td><td>0.02726</td><td>1.065</td><td>0.05554</td><td>-0.01339</td><td>0.0108</td><td>Heel bone mineral density (BMD)</td><td>FALSE</td><td></td><td></td><td></td><td>206496</td><td></td><td></td><td>UK Biobank</td><td>https://docs.google.com/spreadsheets/d/1kvPoupSzsSFBNSztMzl04xMoSC3Kcx3CrjVf4yBmESU/edit?ts=5b5f17db#gid=227859291</td><td>PHESANT Transformation:3148_0|| CONTINUOUS MAIN || CONTINUOUS || ||-Notes:Estimation of bone mineral density (BMD) in the heel is based on the Quantitative Ultrasound Index through the calcaneus. Direct entry.-Variable type:continuous_raw</td></tr>
<tr><td>3160_raw.txt</td><td>1710174270056F5forCTG.txt.gz</td><td>0.5281</td><td>0.3014</td><td>1.752</td><td>0.0798</td><td>0.1077</td><td>0.1038</td><td>1.013</td><td>0.00884</td><td>-0.003165</td><td>0.007107</td><td>Weight, manual entry</td><td>FALSE</td><td></td><td></td><td></td><td>5278</td><td></td><td></td><td>UK Biobank</td><td>https://docs.google.com/spreadsheets/d/1kvPoupSzsSFBNSztMzl04xMoSC3Kcx3CrjVf4yBmESU/edit?ts=5b5f17db#gid=227859291</td><td>PHESANT Transformation:3160_0|| CONTINUOUS MAIN || CONTINUOUS || ||-Notes:Weight (manual entry)-Variable type:continuous_raw</td></tr>
</table>

<table>
<tr><td>3436_raw.txt</td><td>1710174270056F5 forCTG.txt.gz</td><td>-0.09203</td><td>0.08146</td><td>-1.13</td><td>0.2586</td><td>0.06415</td><td>0.01937</td><td>0.999</td><td>0.009702</td><td>-0.01126</td><td>0.008234</td><td>Age started smoking in current smokers</td><td>FALSE</td><td></td><td></td><td></td><td>27291</td><td></td><td></td><td>UK Biobank</td><td>https://docs.google.com/spreadsheets/d/1kvPoupSzsSFBNSztMzl04xMoSC3Kcx3CrjVf4yBmESU/edit?ts=5b5f17db#gid=227859291</td><td>PHESANT Transformation:3436_0|| INTEGER || reassignments: -1=NA|-3=NA || CONTINUOUS || ||-Notes:ACE touchscreen question How old were you when you first started smoking on most days? The following checks were performed: If answer If answer > Participants age then rejected If answer ~F3436~ was collected from participants who indicated they currently smoke tobacco on most or all days, as defined by their answers to ~F1239~-Variable type:continuous_raw</td></tr>
<tr><td>3526_raw.txt</td><td>1710174270056F5 forCTG.txt.gz</td><td>-0.334</td><td>0.06694</td><td>-4.989</td><td>6.08E-07</td><td>0.01417</td><td>0.002704</td><td>1.012</td><td>0.009884</td><td>-0.009374</td><td>0.008549</td><td>Mother's age at death</td><td>FALSE</td><td>Other</td><td></td><td></td><td>213636</td><td></td><td></td><td>UK Biobank</td><td>https://docs.google.com/spreadsheets/d/1kvPoupSzsSFBNSztMzl04xMoSC3Kcx3CrjVf4yBmESU/edit?ts=5b5f17db#gid=227859291</td><td>PHESANT Transformation:3526_0|| INTEGER || reassignments: -1=NA|-3=NA || CONTINUOUS || ||-Notes:ACE touchscreen question What was her age when she died? The following checks were performed: If answer If answer > 122 then rejected If answer If answer > 105 then participant asked to confirm ~F3526~ was collected from participants who indicated their mother has died, as defined by their answers to ~F1835~ or, if they are adopted, their adopted mother has died, as defined by their answers to ~F1835~-Variable type:continuous_raw</td></tr>
<tr><td>3536_raw.txt</td><td>1710174270056F5 forCTG.txt.gz</td><td>-0.3372</td><td>0.05366</td><td>-6.284</td><td>3.30E-10</td><td>0.08798</td><td>0.01128</td><td>0.978</td><td>0.01207</td><td>-0.001994</td><td>0.008655</td><td>Age started hormone-replacement therapy (HRT)</td><td>FALSE</td><td>Reproductive</td><td></td><td></td><td>68299</td><td></td><td></td><td>UK Biobank</td><td>https://docs.google.com/spreadsheets/d/1kvPoupSzsSFBNSztMzl04xMoSC3Kcx3CrjVf4yBmESU/edit?ts=5b5f17db#gid=227859291</td><td>PHESANT Transformation:3536_0|| INTEGER || reassignments: -1=NA|-3=NA || CONTINUOUS || ||-Notes:ACE touchscreen question How old were you when you first used HRT? The following checks were performed: If answer If answer > Participants age then rejected If answer If answer > 65 then participant asked to confirm If the participant activated the Help button they were shown the message: If you are unsure, please provide an estimate or select Do not know. ~F3536~ was collected from women who indicated that they had used HRT, as defined by their answers to ~F2814~-Variable type:continuous_raw</td></tr>
<tr><td>3581_raw.txt</td><td>1710174270056F5 forCTG.txt.gz</td><td>-0.1826</td><td>0.03931</td><td>-4.645</td><td>3.41E-06</td><td>0.123</td><td>0.01197</td><td>0.987</td><td>0.0219</td><td>-0.003894</td><td>0.008411</td><td>Age at menopause (last menstrual period)</td><td>FALSE</td><td>Reproductive</td><td></td><td></td><td>111593</td><td></td><td></td><td>UK Biobank</td><td>https://docs.google.com/spreadsheets/d/1kvPoupSzsSFBNSztMzl04xMoSC3Kcx3CrjVf4yBmESU/edit?ts=5b5f17db#gid=227859291</td><td>PHESANT Transformation:3581_0|| INTEGER || reassignments: -1=NA|-3=NA || CONTINUOUS || ||-Notes:ACE touchscreen question How old were you when your periods stopped? The following checks were performed: If answer If answer > Participants age then rejected If answer > 70 then rejected If answer If answer > 60 then participant asked to confirm If the participant activated the Help button they were shown the message: If you are unsure, please provide an estimate or select Do not know. ~F3581~ was collected from women who indicated that their periods had stopped, as defined by their answers to ~F2724~-Variable type:continuous_raw</td></tr>
<tr><td>3627_raw.txt</td><td>1710174270056F5 forCTG.txt.gz</td><td>-0.1693</td><td>0.1037</td><td>-1.632</td><td>0.1027</td><td>0.122</td><td>0.0529</td><td>0.984</td><td>0.009111</td><td>0.01015</td><td>0.007981</td><td>Age angina diagnosed</td><td>FALSE</td><td></td><td></td><td></td><td>10651</td><td></td><td></td><td>UK Biobank</td><td>https://docs.google.com/spreadsheets/d/1kvPoupSzsSFBNSztMzl04xMoSC3Kcx3CrjVf4yBmESU/edit?ts=5b5f17db#gid=227859291</td><td>PHESANT Transformation:3627_0|| INTEGER || reassignments: -1=NA|-3=NA || CONTINUOUS || ||-Notes:ACE touchscreen question What was your age when the angina was first diagnosed? The following checks were performed: If answer If answer > Participants age then rejected If answer If answer > 69 then participant asked to confirm If the participant activated the Help button they were shown the message: If you are unsure, please provide an estimate or select Do not know. ~F3627~ was collected from participants who indicated they were told by a doctor that they have had an angina, as defined by their answers to ~F6150~-Variable type:continuous_raw</td></tr>
<tr><td>3761_raw.txt</td><td>1710174270056F5 forCTG.txt.gz</td><td>0.09496</td><td>0.0471</td><td>2.016</td><td>0.04378</td><td>0.1001</td><td>0.01301</td><td>1.008</td><td>0.01245</td><td>-0.00431</td><td>0.00906</td><td>Age hay fever, rhinitis or eczema diagnosed</td><td>FALSE</td><td></td><td></td><td></td><td>72232</td><td></td><td></td><td>UK Biobank</td><td>https://docs.google.com/spreadsheets/d/1kvPoupSzsSFBNSztMzl04xMoSC3Kcx3CrjVf4yBmESU/edit?ts=5b5f17db#gid=227859291</td><td>PHESANT Transformation:3761_0|| INTEGER || reassignments: -1=NA|-3=NA || CONTINUOUS || ||-Notes:ACE touchscreen question What was your age when the hayfever, rhinitis or eczema was first diagnosed? The following checks were performed: If answer If answer > Participants age then rejected If answer If answer > 69 then participant asked to confirm If the participant activated the Help button they were shown the message: If you are unsure, please provide an estimate or select Do not know. ~F3761~ was collected from participants who indicated they were told by a doctor that they have had hayfever, allergic rhinitis or eczema, as defined by their answers to ~F6152~-Variable type:continuous_raw</td></tr>
<tr><td>3786_raw.txt</td><td>1710174270056F5 forCTG.txt.gz</td><td>0.1101</td><td>0.04885</td><td>2.254</td><td>0.02422</td><td>0.1556</td><td>0.03229</td><td>1.004</td><td>0.01363</td><td>0.003973</td><td>0.008283</td><td>Age asthma diagnosed</td><td>FALSE</td><td></td><td></td><td></td><td>36955</td><td></td><td></td><td>UK Biobank</td><td>https://docs.google.com/spreadsheets/d/1kvPoupSzsSFBNSztMzl04xMoSC3Kcx3CrjVf4yBmESU/edit?ts=5b5f17db#gid=227859291</td><td>PHESANT Transformation:3786_0|| INTEGER || reassignments: -1=NA|-3=NA || CONTINUOUS || ||-Notes:ACE touchscreen question What was your age when the asthma was first diagnosed? The following checks were performed: If answer If answer > Participants age then rejected If answer If answer > 69 then participant asked to confirm If the participant activated the Help button they were shown the message: If you are unsure, please provide an estimate or select Do not know. ~F3786~ was collected from participants who indicated they were told by a doctor that they have had asthma, as defined by their answers to ~F6152~-Variable type:continuous_raw</td></tr>
<tr><td>3872_raw.txt</td><td>1710174270056F5 forCTG.txt.gz</td><td>-0.3514</td><td>0.09935</td><td>-3.537</td><td>0.000405</td><td>0.08898</td><td>0.02345</td><td>1.008</td><td>0.009456</td><td>-0.006266</td><td>0.008386</td><td>Age of primiparous women at birth of child</td><td>FALSE</td><td></td><td></td><td></td><td>25545</td><td></td><td></td><td>UK Biobank</td><td>https://docs.google.com/spreadsheets/d/1kvPoupSzsSFBNSztMzl04xMoSC3Kcx3CrjVf4yBmESU/edit?ts=5b5f17db#gid=227859291</td><td>PHESANT Transformation:3872_0|| INTEGER || reassignments: -4=NA|-3=NA || CONTINUOUS || ||-Notes:ACE touchscreen question How old were you when you had your child? The following checks were performed: If answer If answer > Participants age then rejected If answer > 65 then rejected If answer If answer > 48 then participant asked to confirm If answer > Age when periods stopped then participant asked to confirm ~F3872~ was collected from women who indicated they had given birth to only one child, as defined by their answers to ~F2734~-Variable type:continuous_raw</td></tr>
<tr><td>3894_raw.txt</td><td>1710174270056F5 forCTG.txt.gz</td><td>-0.3217</td><td>0.1866</td><td>-1.724</td><td>0.08467</td><td>0.09754</td><td>0.07023</td><td>0.988</td><td>0.009743</td><td>0.006517</td><td>0.007332</td><td>Age heart attack diagnosed</td><td>FALSE</td><td></td><td></td><td></td><td>8024</td><td></td><td></td><td>UK Biobank</td><td>https://docs.google.com/spreadsheets/d/1kvPoupSzsSFBNSztMzl04xMoSC3Kcx3CrjVf4yBmESU/edit?ts=5b5f17db#gid=227859291</td><td>PHESANT Transformation:3894_0|| INTEGER || reassignments: -1=NA|-3=NA || CONTINUOUS || ||-Notes:ACE touchscreen question What was your age when the heart attack was first diagnosed? The following checks were performed: If answer If answer > Participants age then rejected If answer If answer > 69 then participant asked to confirm If the participant activated the Help button they were shown the message: If you are unsure, please provide an estimate or select Do not know. ~F3894~ was collected from participants who indicated they were told by a doctor that they have had a heart attack, as defined by their answers to ~F6150~-Variable type:continuous_raw</td></tr>
<tr><td>399_raw.txt</td><td>1710174270056F5 forCTG.txt.gz</td><td>0.1155</td><td>0.031</td><td>3.728</td><td>0.000193</td><td>0.0567</td><td>0.003253</td><td>1.016</td><td>0.01373</td><td>-0.01039</td><td>0.009352</td><td>Number of incorrect matches in round</td><td>FALSE</td><td></td><td></td><td></td><td>360686</td><td></td><td></td><td>UK Biobank</td><td>https://docs.google.com/spreadsheets/d/1kvPoupSzsSFBNSztMzl04xMoSC3Kcx3CrjVf4yBmESU/edit?ts=5b5f17db#gid=227859291</td><td>PHESANT Transformation:399_0|| INTEGER || CONTINUOUS || ||-Notes:A value of 0 indicates the participant made no mistakes.-Variable type:continuous_raw</td></tr>
<tr><td>400_raw.txt</td><td>1710174270056F5 forCTG.txt.gz</td><td>0.08595</td><td>0.03109</td><td>2.764</td><td>0.005702</td><td>0.05783</td><td>0.003236</td><td>1.042</td><td>0.01443</td><td>-0.01236</td><td>0.009014</td><td>Time to complete round</td><td>FALSE</td><td></td><td></td><td></td><td>354739</td><td></td><td></td><td>UK Biobank</td><td>https://docs.google.com/spreadsheets/d/1kvPoupSzsSFBNSztMzl04xMoSC3Kcx3CrjVf4yBmESU/edit?ts=5b5f17db#gid=227859291</td><td>PHESANT Transformation:400_0|| INTEGER || reassignments: 0=NA || CONTINUOUS || ||-Notes:-Variable type:continuous_raw</td></tr>
<tr><td>40007_raw.txt</td><td>1710174270056F5 forCTG.txt.gz</td><td>-0.04071</td><td>0.1288</td><td>-0.316</td><td>0.752</td><td>0.06427</td><td>0.05398</td><td>0.993</td><td>0.009122</td><td>-0.002639</td><td>0.007863</td><td>Age at death</td><td>FALSE</td><td></td><td></td><td></td><td>10483</td><td></td><td></td><td>UK Biobank</td><td>https://docs.google.com/spreadsheets/d/1kvPoupSzsSFBNSztMzl04xMoSC3Kcx3CrjVf4yBmESU/edit?ts=5b5f17db#gid=227859291</td><td>PHESANT Transformation:40007_0|| CONTINUOUS MAIN || CONTINUOUS || ||-Notes:Age calculated as interval between Date of Birth and Death.-Variable type:continuous_raw</td></tr>
<tr><td>404_raw.txt</td><td>1710174270056F5 forCTG.txt.gz</td><td>-0.06005</td><td>0.03096</td><td>-1.94</td><td>0.05241</td><td>0.0588</td><td>0.002929</td><td>1.017</td><td>0.01453</td><td>0.005159</td><td>0.009508</td><td>Duration to first press of snap-button in each round</td><td>FALSE</td><td></td><td></td><td></td><td>358500</td><td></td><td></td><td>UK Biobank</td><td>https://docs.google.com/spreadsheets/d/1kvPoupSzsSFBNSztMzl04xMoSC3Kcx3CrjVf4yBmESU/edit?ts=5b5f17db#gid=227859291</td><td>PHESANT Transformation:404_0|| INTEGER || CONTINUOUS || ||-Notes:This gives the interval between the cards being displayed and the participant pressing the snap-button to indicate a match. Note that the time is recorded regardless of whether the cards shown formed a matching pair. ~F401~ and ~F402~ need to be compared to determine if the cards were the same. Each pair was displayed for 2 seconds, followed by a 1 second gap. If the button-press occurred during a gap then it was recorded against the previous pair, giving a value in the range 2001-2999ms.-Variable type:continuous_raw</td></tr>
<tr><td>4079_raw.txt</td><td>1710174270056F5 forCTG.txt.gz</td><td>0.101</td><td>0.026</td><td>3.884</td><td>0.000103</td><td>0.1303</td><td>0.006645</td><td>1.089</td><td>0.02738</td><td>0.01159</td><td>0.01029</td><td>Diastolic blood pressure, automated reading</td><td>FALSE</td><td></td><td></td><td></td><td>340162</td><td></td><td></td><td>UK Biobank</td><td>https://docs.google.com/spreadsheets/d/1kvPoupSzsSFBNSztMzl04xMoSC3Kcx3CrjVf4yBmESU/edit?ts=5b5f17db#gid=227859291</td><td>PHESANT Transformation:4079_0|| INTEGER || CONTINUOUS || ||-Notes:Blood pressure, automated reading, diastolic. Two measures of blood pressure were taken a few moments apart. Range returned by the Omron device is is 0-255-Variable type:continuous_raw</td></tr>
<tr><td>4080_raw.txt</td><td>1710174270056F5 forCTG.txt.gz</td><td>0.07011</td><td>0.03049</td><td>2.3</td><td>0.02147</td><td>0.1379</td><td>0.007169</td><td>1.085</td><td>0.02777</td><td>-0.001754</td><td>0.01123</td><td>Systolic blood pressure, automated reading</td><td>FALSE</td><td></td><td></td><td></td><td>340159</td><td></td><td></td><td>UK Biobank</td><td>https://docs.google.com/spreadsheets/d/1kvPoupSzsSFBNSztMzl04xMoSC3Kcx3CrjVf4yBmESU/edit?ts=5b5f17db#gid=227859291</td><td>PHESANT Transformation:4080_0|| INTEGER || CONTINUOUS || ||-Notes:Blood pressure, automated reading, systolic. Two measures of blood pressure were taken a few moments apart. Range returned by the Omron device is is 0-255-Variable type:continuous_raw</td></tr>
<tr><td>4100_raw.txt</td><td>1710174270056F5 forCTG.txt.gz</td><td>0.1039</td><td>0.02754</td><td>3.773</td><td>0.000161</td><td>0.2981</td><td>0.02031</td><td>1.041</td><td>0.02239</td><td>0.02069</td><td>0.009105</td><td>Ankle spacing width (left)</td><td>FALSE</td><td></td><td></td><td></td><td>114630</td><td></td><td></td><td>UK Biobank</td><td>https://docs.google.com/spreadsheets/d/1kvPoupSzsSFBNSztMzl04xMoSC3Kcx3CrjVf4yBmESU/edit?ts=5b5f17db#gid=227859291</td><td>PHESANT Transformation:4100_0|| CONTINUOUS MAIN || CONTINUOUS || ||-Notes:Left ankle width as indicated by the spacing between measurement transducer pads on heel-Variable type:continuous_raw</td></tr>
<tr><td>4101_raw.txt</td><td>1710174270056F5 forCTG.txt.gz</td><td>0.06312</td><td>0.0299</td><td>2.111</td><td>0.03478</td><td>0.2547</td><td>0.03299</td><td>1.001</td><td>0.0329</td><td>-0.000465</td><td>0.01015</td><td>Heel broadband ultrasound attenuation (left)</td><td>FALSE</td><td></td><td></td><td></td><td>114625</td><td></td><td></td><td>UK Biobank</td><td>https://docs.google.com/spreadsheets/d/1kvPoupSzsSFBNSztMzl04xMoSC3Kcx3CrjVf4yBmESU/edit?ts=5b5f17db#gid=227859291</td><td>PHESANT Transformation:4101_0|| CONTINUOUS MAIN || CONTINUOUS || ||-Notes:Broadband ultrasound attenuation through left heel-Variable type:continuous_raw</td></tr>
<tr><td>4104_raw.txt</td><td>1710174270056F5 forCTG.txt.gz</td><td>0.05016</td><td>0.02868</td><td>1.749</td><td>0.08031</td><td>0.2687</td><td>0.03525</td><td>1.016</td><td>0.03536</td><td>-0.004691</td><td>0.01009</td><td>Heel quantitative ultrasound index (QUI), direct entry (left)</td><td>FALSE</td><td></td><td></td><td></td><td>114630</td><td></td><td></td><td>UK Biobank</td><td>https://docs.google.com/spreadsheets/d/1kvPoupSzsSFBNSztMzl04xMoSC3Kcx3CrjVf4yBmESU/edit?ts=5b5f17db#gid=227859291</td><td>PHESANT Transformation:4104_0|| CONTINUOUS MAIN || CONTINUOUS || ||-Notes:The QUI or 'stiffness' is based on measure of the speed of sound and broadband ultrasound attenuation. Direct entry.-Variable type:continuous_raw</td></tr>
<tr><td>4105_raw.txt</td><td>1710174270056F5 forCTG.txt.gz</td><td>0.05167</td><td>0.02847</td><td>1.815</td><td>0.06953</td><td>0.2728</td><td>0.03567</td><td>1.016</td><td>0.03565</td><td>-0.005106</td><td>0.01011</td><td>Heel bone mineral density (BMD) (left)</td><td>FALSE</td><td></td><td></td><td></td><td>114561</td><td></td><td></td><td>UK Biobank</td><td>https://docs.google.com/spreadsheets/d/1kvPoupSzsSFBNSztMzl04xMoSC3Kcx3CrjVf4yBmESU/edit?ts=5b5f17db#gid=227859291</td><td>PHESANT Transformation:4105_0|| CONTINUOUS MAIN || CONTINUOUS || ||-Notes:Estimation of bone mineral density (BMD) in the left heel is based on the Quantitative Ultrasound Index through the calcaneus. Direct entry.-Variable type:continuous_raw</td></tr>
</table>

<table>
<tr><td>4106_raw.txt</td><td>1710174270056F5forCTG.txt.gz</td><td>0.05016</td><td>0.02868</td><td>1.749</td><td>0.08035</td><td>0.2687</td><td>0.03525</td><td>1.016</td><td>0.03536</td><td>-0.004687</td><td>0.01009</td><td>Heel bone mineral density (BMD) T-score, automated (left)</td><td>FALSE</td><td></td><td></td><td></td><td>114630</td><td></td><td></td><td>UK Biobank</td><td>https://docs.google.com/spreadsheets/d/1kvPoupSzsSFBNSztMzl04xMoSC3Kcx3CrjVf4yBmESU/edit?ts=5b5f17db#gid=227859291</td><td>PHESANT Transformation:4106_0| | CONTINUOUS MAIN | | CONTINUOUS | | | |-Notes:This T-score is calculated from the ultrasound heel BMD measurement and is based on a person's bone density compared with what is normally expected in someone of the same sex. The units of the T-score are the number of standard deviations (SD) that the bone density is above or below the standard.-Variable type:continuous_raw</td></tr>
<tr><td>4119_raw.txt</td><td>1710174270056F5forCTG.txt.gz</td><td>0.1039</td><td>0.0273</td><td>3.807</td><td>0.000141</td><td>0.2942</td><td>0.01975</td><td>1.044</td><td>0.021</td><td>0.02017</td><td>0.008852</td><td>Ankle spacing width (right)</td><td>FALSE</td><td></td><td></td><td></td><td>114614</td><td></td><td></td><td>UK Biobank</td><td>https://docs.google.com/spreadsheets/d/1kvPoupSzsSFBNSztMzl04xMoSC3Kcx3CrjVf4yBmESU/edit?ts=5b5f17db#gid=227859291</td><td>PHESANT Transformation:4119_0| | CONTINUOUS MAIN | | CONTINUOUS | | | |-Notes:Right ankle width as indicated by the spacing between measurement transducer pads on heel-Variable type:continuous_raw</td></tr>
<tr><td>4120_raw.txt</td><td>1710174270056F5forCTG.txt.gz</td><td>0.05745</td><td>0.02882</td><td>1.993</td><td>0.04623</td><td>0.2577</td><td>0.03352</td><td>1.004</td><td>0.03364</td><td>0.00345</td><td>0.009774</td><td>Heel broadband ultrasound attenuation (right)</td><td>FALSE</td><td></td><td></td><td></td><td>114609</td><td></td><td></td><td>UK Biobank</td><td>https://docs.google.com/spreadsheets/d/1kvPoupSzsSFBNSztMzl04xMoSC3Kcx3CrjVf4yBmESU/edit?ts=5b5f17db#gid=227859291</td><td>PHESANT Transformation:4120_0| | CONTINUOUS MAIN | | CONTINUOUS | | | |-Notes:Broadband ultrasound attenuation through right heel-Variable type:continuous_raw</td></tr>
<tr><td>4123_raw.txt</td><td>1710174270056F5forCTG.txt.gz</td><td>0.05347</td><td>0.02732</td><td>1.958</td><td>0.05028</td><td>0.2805</td><td>0.03688</td><td>1.012</td><td>0.03643</td><td>-0.005085</td><td>0.009869</td><td>Heel quantitative ultrasound index (QUI), direct entry (right)</td><td>FALSE</td><td></td><td></td><td></td><td>114614</td><td></td><td></td><td>UK Biobank</td><td>https://docs.google.com/spreadsheets/d/1kvPoupSzsSFBNSztMzl04xMoSC3Kcx3CrjVf4yBmESU/edit?ts=5b5f17db#gid=227859291</td><td>PHESANT Transformation:4123_0| | CONTINUOUS MAIN | | CONTINUOUS | | | |-Notes:The QUI or 'stiffness' is based on measure of the speed of sound and broadband ultrasound attenuation. Direct entry.-Variable type:continuous_raw</td></tr>
<tr><td>4124_raw.txt</td><td>1710174270056F5forCTG.txt.gz</td><td>0.0525</td><td>0.02736</td><td>1.919</td><td>0.05501</td><td>0.2831</td><td>0.03746</td><td>1.012</td><td>0.03701</td><td>-0.004275</td><td>0.01001</td><td>Heel bone mineral density (BMD) (right)</td><td>FALSE</td><td></td><td></td><td></td><td>114552</td><td></td><td></td><td>UK Biobank</td><td>https://docs.google.com/spreadsheets/d/1kvPoupSzsSFBNSztMzl04xMoSC3Kcx3CrjVf4yBmESU/edit?ts=5b5f17db#gid=227859291</td><td>PHESANT Transformation:4124_0| | CONTINUOUS MAIN | | CONTINUOUS | | | |-Notes:Estimation of bone mineral density (BMD) in the left heel is based on the Quantitative Ultrasound Index through the calcaneus. Direct entry.-Variable type:continuous_raw</td></tr>
<tr><td>4125_raw.txt</td><td>1710174270056F5forCTG.txt.gz</td><td>0.05348</td><td>0.02732</td><td>1.958</td><td>0.05026</td><td>0.2805</td><td>0.03688</td><td>1.012</td><td>0.03643</td><td>-0.005088</td><td>0.009869</td><td>Heel bone mineral density (BMD) T-score, automated (right)</td><td>FALSE</td><td></td><td></td><td></td><td>114614</td><td></td><td></td><td>UK Biobank</td><td>https://docs.google.com/spreadsheets/d/1kvPoupSzsSFBNSztMzl04xMoSC3Kcx3CrjVf4yBmESU/edit?ts=5b5f17db#gid=227859291</td><td>PHESANT Transformation:4125_0| | CONTINUOUS MAIN | | CONTINUOUS | | | |-Notes:This T-score is calculated from the ultrasound heel BMD measurement and is based on a person's bone density compared with what is normally expected in someone of the same sex. The units of the T-score are the number of standard deviations (SD) that the bone density is above or below the standard.-Variable type:continuous_raw</td></tr>
<tr><td>4194_raw.txt</td><td>1710174270056F5forCTG.txt.gz</td><td>0.07305</td><td>0.03802</td><td>1.921</td><td>0.05468</td><td>0.1216</td><td>0.01298</td><td>1.037</td><td>0.02326</td><td>0.0001116</td><td>0.009806</td><td>Pulse rate</td><td>FALSE</td><td></td><td></td><td></td><td>118850</td><td></td><td></td><td>UK Biobank</td><td>https://docs.google.com/spreadsheets/d/1kvPoupSzsSFBNSztMzl04xMoSC3Kcx3CrjVf4yBmESU/edit?ts=5b5f17db#gid=227859291</td><td>PHESANT Transformation:4194_0| | INTEGER | | CONTINUOUS | | | |-Notes:Pulse rate during arterial stiffness measurement-Variable type:continuous_raw</td></tr>
<tr><td>4195_raw.txt</td><td>1710174270056F5forCTG.txt.gz</td><td>-0.1279</td><td>0.131</td><td>-0.9757</td><td>0.3292</td><td>0.006032</td><td>0.004817</td><td>1.015</td><td>0.00958</td><td>0.01625</td><td>0.008131</td><td>Pulse wave reflection index</td><td>FALSE</td><td></td><td></td><td></td><td>118850</td><td></td><td></td><td>UK Biobank</td><td>https://docs.google.com/spreadsheets/d/1kvPoupSzsSFBNSztMzl04xMoSC3Kcx3CrjVf4yBmESU/edit?ts=5b5f17db#gid=227859291</td><td>PHESANT Transformation:4195_0| | INTEGER | | CONTINUOUS | | | |-Notes:reflection index is a measure of reflection A digital volume pulse can be considered to be the summation of a direct and a reflected component. The direct component is due to transmission of a pressure wave from the left ventricle to the finger via the most direct route. The reflected component is formed by pressure transmitted from the heart to the lower body where it is reflected back up the aorta and thence to the finger. if 'b' is the height of direct wave peak and 'a' is the height of reflected wave peak the reflected index = (a/b) * 100%-Variable type:continuous_raw</td></tr>
<tr><td>4196_raw.txt</td><td>1710174270056F5forCTG.txt.gz</td><td>-0.2484</td><td>0.05795</td><td>-4.287</td><td>1.81E-05</td><td>0.02874</td><td>0.005384</td><td>1.002</td><td>0.01044</td><td>-0.004697</td><td>0.007444</td><td>Pulse wave peak to peak time</td><td>FALSE</td><td>Cardiac</td><td></td><td></td><td>118787</td><td></td><td></td><td>UK Biobank</td><td>https://docs.google.com/spreadsheets/d/1kvPoupSzsSFBNSztMzl04xMoSC3Kcx3CrjVf4yBmESU/edit?ts=5b5f17db#gid=227859291</td><td>PHESANT Transformation:4196_0| | INTEGER | | CONTINUOUS | | | |-Notes:peak to peak time in milliseconds A digital volume pulse can be considered to be the summation of a direct and a reflected component. The direct component is due to transmission of a pressure wave from the left ventricle to the finger via the most direct route. The reflected component is formed by pressure transmitted from the heart to the lower body where it is reflected back up the aorta and thence to the finger. peak to peak time is the difference between the peak values of direct and reflected components.-Variable type:continuous_raw</td></tr>
<tr><td>4230_raw.txt</td><td>1710174270056F5forCTG.txt.gz</td><td>0.2547</td><td>0.07681</td><td>3.316</td><td>0.000912</td><td>0.02029</td><td>0.005393</td><td>1.006</td><td>0.01083</td><td>0.0008389</td><td>0.007743</td><td>Signal-to-noise-ratio (SNR) of triplet (left)</td><td>FALSE</td><td></td><td></td><td></td><td>116790</td><td></td><td></td><td>UK Biobank</td><td>https://docs.google.com/spreadsheets/d/1kvPoupSzsSFBNSztMzl04xMoSC3Kcx3CrjVf4yBmESU/edit?ts=5b5f17db#gid=227859291</td><td>PHESANT Transformation:4230_0| | INTEGER | | CONTINUOUS | | | |-Notes:This is the signal-to-noise ratio for each round (left ear)-Variable type:continuous_raw</td></tr>
<tr><td>4241_raw.txt</td><td>1710174270056F5forCTG.txt.gz</td><td>0.2371</td><td>0.08491</td><td>2.792</td><td>0.005235</td><td>0.01795</td><td>0.00548</td><td>1.01</td><td>0.01046</td><td>-0.002409</td><td>0.008373</td><td>Signal-to-noise-ratio (SNR) of triplet (right)</td><td>FALSE</td><td></td><td></td><td></td><td>116755</td><td></td><td></td><td>UK Biobank</td><td>https://docs.google.com/spreadsheets/d/1kvPoupSzsSFBNSztMzl04xMoSC3Kcx3CrjVf4yBmESU/edit?ts=5b5f17db#gid=227859291</td><td>PHESANT Transformation:4241_0| | INTEGER | | CONTINUOUS | | | |-Notes:This is the signal-to-noise ratio for each round (right ear)-Variable type:continuous_raw</td></tr>
<tr><td>4288_raw.txt</td><td>1710174270056F5forCTG.txt.gz</td><td>0.1897</td><td>0.06484</td><td>2.926</td><td>0.003434</td><td>0.02587</td><td>0.005659</td><td>1.067</td><td>0.01075</td><td>-0.00973</td><td>0.008035</td><td>Time to answer</td><td>FALSE</td><td></td><td></td><td></td><td>119729</td><td></td><td></td><td>UK Biobank</td><td>https://docs.google.com/spreadsheets/d/1kvPoupSzsSFBNSztMzl04xMoSC3Kcx3CrjVf4yBmESU/edit?ts=5b5f17db#gid=227859291</td><td>PHESANT Transformation:4288_0| | INTEGER | | CONTINUOUS | | | |-Notes:Time between completing initial prompt and displaying answer-Variable type:continuous_raw</td></tr>
<tr><td>4290_raw.txt</td><td>1710174270056F5forCTG.txt.gz</td><td>0.3</td><td>0.04599</td><td>6.523</td><td>6.89E-11</td><td>0.05775</td><td>0.006448</td><td>1.006</td><td>0.01142</td><td>-0.005764</td><td>0.008419</td><td>Duration screen displayed</td><td>TRUE</td><td>Lifestyle</td><td></td><td>Screentime duration</td><td>119729</td><td></td><td></td><td>UK Biobank</td><td>https://docs.google.com/spreadsheets/d/1kvPoupSzsSFBNSztMzl04xMoSC3Kcx3CrjVf4yBmESU/edit?ts=5b5f17db#gid=227859291</td><td>PHESANT Transformation:4290_0| | INTEGER | | CONTINUOUS | | | |-Notes:Time for which screen was visible.-Variable type:continuous_raw</td></tr>
<tr><td>46_raw.txt</td><td>1710174270056F5forCTG.txt.gz</td><td>-0.0316</td><td>0.02827</td><td>-1.118</td><td>0.2637</td><td>0.1202</td><td>0.005998</td><td>1.037</td><td>0.02252</td><td>0.003422</td><td>0.01138</td><td>Hand grip strength (left)</td><td>FALSE</td><td></td><td></td><td></td><td>359704</td><td></td><td></td><td>UK Biobank</td><td>https://docs.google.com/spreadsheets/d/1kvPoupSzsSFBNSztMzl04xMoSC3Kcx3CrjVf4yBmESU/edit?ts=5b5f17db#gid=227859291</td><td>PHESANT Transformation:46_0| | INTEGER | | CONTINUOUS | | | |-Notes:Left grip strength-Variable type:continuous_raw</td></tr>
<tr><td>47_raw.txt</td><td>1710174270056F5forCTG.txt.gz</td><td>-0.06202</td><td>0.02829</td><td>-2.193</td><td>0.02833</td><td>0.121</td><td>0.006237</td><td>1.039</td><td>0.02331</td><td>0.00751</td><td>0.01096</td><td>Hand grip strength (right)</td><td>FALSE</td><td></td><td></td><td></td><td>359729</td><td></td><td></td><td>UK Biobank</td><td>https://docs.google.com/spreadsheets/d/1kvPoupSzsSFBNSztMzl04xMoSC3Kcx3CrjVf4yBmESU/edit?ts=5b5f17db#gid=227859291</td><td>PHESANT Transformation:47_0| | INTEGER | | CONTINUOUS | | | |-Notes:Right grip strength-Variable type:continuous_raw</td></tr>
<tr><td>48_raw.txt</td><td>1710174270056F5forCTG.txt.gz</td><td>0.2574</td><td>0.02436</td><td>10.57</td><td>4.11E-26</td><td>0.1972</td><td>0.008331</td><td>1.075</td><td>0.03225</td><td>0.03266</td><td>0.01164</td><td>Waist circumference</td><td>FALSE</td><td>Body composition</td><td></td><td></td><td>360564</td><td></td><td></td><td>UK Biobank</td><td>https://docs.google.com/spreadsheets/d/1kvPoupSzsSFBNSztMzl04xMoSC3Kcx3CrjVf4yBmESU/edit?ts=5b5f17db#gid=227859291</td><td>PHESANT Transformation:48_0| | CONTINUOUS MAIN | | CONTINUOUS | | | |-Notes:Waist circumference-Variable type:continuous_raw</td></tr>
<tr><td>49_raw.txt</td><td>1710174270056F5forCTG.txt.gz</td><td>0.1983</td><td>0.02362</td><td>8.395</td><td>4.66E-17</td><td>0.2028</td><td>0.009072</td><td>1.099</td><td>0.03113</td><td>0.03337</td><td>0.01135</td><td>Hip circumference</td><td>FALSE</td><td>Body composition</td><td></td><td></td><td>360521</td><td></td><td></td><td>UK Biobank</td><td>https://docs.google.com/spreadsheets/d/1kvPoupSzsSFBNSztMzl04xMoSC3Kcx3CrjVf4yBmESU/edit?ts=5b5f17db#gid=227859291</td><td>PHESANT Transformation:49_0| | CONTINUOUS MAIN | | CONTINUOUS | | | |-Notes:Hip circumference-Variable type:continuous_raw</td></tr>
<tr><td>50_raw.txt</td><td>1710174270056F5forCTG.txt.gz</td><td>-0.08632</td><td>0.02294</td><td>-3.763</td><td>0.000168</td><td>0.4697</td><td>0.02961</td><td>1.261</td><td>0.1088</td><td>0.01373</td><td>0.01498</td><td>Standing height</td><td>FALSE</td><td></td><td></td><td></td><td>360388</td><td></td><td></td><td>UK Biobank</td><td>https://docs.google.com/spreadsheets/d/1kvPoupSzsSFBNSztMzl04xMoSC3Kcx3CrjVf4yBmESU/edit?ts=5b5f17db#gid=227859291</td><td>PHESANT Transformation:50_0| | CONTINUOUS MAIN | | CONTINUOUS | | | |-Notes:Standing height was measured using a Seca 202 device-Variable type:continuous_raw</td></tr>
<tr><td>5084_raw.txt</td><td>1710174270056F5forCTG.txt.gz</td><td>0.1606</td><td>0.03422</td><td>4.693</td><td>2.69E-06</td><td>0.2501</td><td>0.01861</td><td>1.085</td><td>0.02021</td><td>0.005617</td><td>0.009693</td><td>Spherical power (right)</td><td>FALSE</td><td>Ophthalmic</td><td></td><td></td><td>77983</td><td></td><td></td><td>UK Biobank</td><td>https://docs.google.com/spreadsheets/d/1kvPoupSzsSFBNSztMzl04xMoSC3Kcx3CrjVf4yBmESU/edit?ts=5b5f17db#gid=227859291</td><td>PHESANT Transformation:5084_0| | CONTINUOUS MAIN | | CONTINUOUS | | | |-Notes:This is the spherical power of refractometry results for the right eye (0.00 indicates none)-Variable type:continuous_raw</td></tr>
<tr><td>5085_raw.txt</td><td>1710174270056F5forCTG.txt.gz</td><td>0.1479</td><td>0.03371</td><td>4.387</td><td>1.15E-05</td><td>0.258</td><td>0.01896</td><td>1.073</td><td>0.02</td><td>0.008399</td><td>0.009737</td><td>Spherical power (left)</td><td>FALSE</td><td>Ophthalmic</td><td></td><td></td><td>77739</td><td></td><td></td><td>UK Biobank</td><td>https://docs.google.com/spreadsheets/d/1kvPoupSzsSFBNSztMzl04xMoSC3Kcx3CrjVf4yBmESU/edit?ts=5b5f17db#gid=227859291</td><td>PHESANT Transformation:5085_0| | CONTINUOUS MAIN | | CONTINUOUS | | | |-Notes:This is the spherical power of refractometry results for the left eye (0.00 indicates none)-Variable type:continuous_raw</td></tr>
<tr><td>5086_raw.txt</td><td>1710174270056F5forCTG.txt.gz</td><td>-0.06112</td><td>0.08153</td><td>-0.7497</td><td>0.4535</td><td>0.02791</td><td>0.007896</td><td>1</td><td>0.01004</td><td>0.002411</td><td>0.007776</td><td>Cylindrical power (left)</td><td>FALSE</td><td></td><td></td><td></td><td>77739</td><td></td><td></td><td>UK Biobank</td><td>https://docs.google.com/spreadsheets/d/1kvPoupSzsSFBNSztMzl04xMoSC3Kcx3CrjVf4yBmESU/edit?ts=5b5f17db#gid=227859291</td><td>PHESANT Transformation:5086_0| | CONTINUOUS MAIN | | CONTINUOUS | | | |-Notes:This is the cylindrical power of refractometry results for the left eye (0.00 indicates none)-Variable type:continuous_raw</td></tr>
<tr><td>5087_raw.txt</td><td>1710174270056F5forCTG.txt.gz</td><td>-0.02009</td><td>0.0714</td><td>-0.2814</td><td>0.7784</td><td>0.03735</td><td>0.006993</td><td>0.995</td><td>0.009166</td><td>0.003732</td><td>0.008352</td><td>Cylindrical power (right)</td><td>FALSE</td><td></td><td></td><td></td><td>77983</td><td></td><td></td><td>UK Biobank</td><td>https://docs.google.com/spreadsheets/d/1kvPoupSzsSFBNSztMzl04xMoSC3Kcx3CrjVf4yBmESU/edit?ts=5b5f17db#gid=227859291</td><td>PHESANT Transformation:5087_0| | CONTINUOUS MAIN | | CONTINUOUS | | | |-Notes:This is the cylindrical power of refractometry results for the right eye (0.00 indicates none)-Variable type:continuous_raw</td></tr>
<tr><td>5088_raw.txt</td><td>1710174270056F5forCTG.txt.gz</td><td>0.05861</td><td>0.09052</td><td>0.6475</td><td>0.5173</td><td>0.01954</td><td>0.007348</td><td>1.023</td><td>0.009121</td><td>0.007847</td><td>0.008068</td><td>Astigmatism angle (right)</td><td>FALSE</td><td></td><td></td><td></td><td>77983</td><td></td><td></td><td>UK Biobank</td><td>https://docs.google.com/spreadsheets/d/1kvPoupSzsSFBNSztMzl04xMoSC3Kcx3CrjVf4yBmESU/edit?ts=5b5f17db#gid=227859291</td><td>PHESANT Transformation:5088_0| | INTEGER | | CONTINUOUS | | | |-Notes:This is the astigmatism axial angle of refractometry results for the right eye (blank value indicates no cylinder power)-Variable type:continuous_raw</td></tr>
<tr><td>5089_raw.txt</td><td>1710174270056F5forCTG.txt.gz</td><td>-0.008659</td><td>0.08591</td><td>-0.1008</td><td>0.9197</td><td>0.02577</td><td>0.007908</td><td>1.012</td><td>0.01025</td><td>-0.001144</td><td>0.008294</td><td>Astigmatism angle (left)</td><td>FALSE</td><td></td><td></td><td></td><td>77739</td><td></td><td></td><td>UK Biobank</td><td>https://docs.google.com/spreadsheets/d/1kvPoupSzsSFBNSztMzl04xMoSC3Kcx3CrjVf4yBmESU/edit?ts=5b5f17db#gid=227859291</td><td>PHESANT Transformation:5089_0| | INTEGER | | CONTINUOUS | | | |-Notes:This is the astigmatism axial angle of refractometry results for the left eye (blank value indicates no cylinder power)-Variable type:continuous_raw</td></tr>
<tr><td>5096_raw.txt</td><td>1710174270056F5forCTG.txt.gz</td><td>0.08514</td><td>0.03325</td><td>2.561</td><td>0.01045</td><td>0.3381</td><td>0.02556</td><td>1.075</td><td>0.02496</td><td>-0.01006</td><td>0.01005</td><td>3mm weak meridian (left)</td><td>FALSE</td><td></td><td></td><td></td><td>75398</td><td></td><td></td><td>UK Biobank</td><td>https://docs.google.com/spreadsheets/d/1kvPoupSzsSFBNSztMzl04xMoSC3Kcx3CrjVf4yBmESU/edit?ts=5b5f17db#gid=227859291</td><td>PHESANT Transformation:5096_0| | CONTINUOUS MAIN | | CONTINUOUS | | | |-Notes:This is the weak meridian of keratometry results taken at 3mm for the left eye-Variable type:continuous_raw</td></tr>
<tr><td>5097_raw.txt</td><td>1710174270056F5forCTG.txt.gz</td><td>0.1075</td><td>0.03402</td><td>3.16</td><td>0.001576</td><td>0.3591</td><td>0.02883</td><td>1.078</td><td>0.02405</td><td>-0.01655</td><td>0.01014</td><td>6mm weak meridian (left)</td><td>FALSE</td><td></td><td></td><td></td><td>65551</td><td></td><td></td><td>UK Biobank</td><td>https://docs.google.com/spreadsheets/d/1kvPoupSzsSFBNSztMzl04xMoSC3Kcx3CrjVf4yBmESU/edit?ts=5b5f17db#gid=227859291</td><td>PHESANT Transformation:5097_0| | CONTINUOUS MAIN | | CONTINUOUS | | | |-Notes:This is the weak meridian of keratometry results taken at 3mm for the left eye-Variable type:continuous_raw</td></tr>
<tr><td>5098_raw.txt</td><td>1710174270056F5forCTG.txt.gz</td><td>0.08465</td><td>0.03318</td><td>2.551</td><td>0.01074</td><td>0.3652</td><td>0.02774</td><td>1.07</td><td>0.02405</td><td>-0.008343</td><td>0.01</td><td>6mm weak meridian (right)</td><td>FALSE</td><td></td><td></td><td></td><td>66256</td><td></td><td></td><td>UK Biobank</td><td>https://docs.google.com/spreadsheets/d/1kvPoupSzsSFBNSztMzl04xMoSC3Kcx3CrjVf4yBmESU/edit?ts=5b5f17db#gid=227859291</td><td>PHESANT Transformation:5098_0| | CONTINUOUS MAIN | | CONTINUOUS | | | |-Notes:This is the weak meridian of keratometry results taken at 6mm for the right eye-Variable type:continuous_raw</td></tr>
</table>

<table>
<tr><td></td><td></td><td></td><td></td><td></td><td></td><td></td><td></td><td></td><td></td><td></td><td></td><td></td><td></td><td></td><td></td><td></td><td></td><td></td><td></td><td></td><td></td><td></td></tr>
<tr><td>5108_raw.txt</td><td>1710174270056F5forCTG.txt.gz</td><td>0.01575</td><td>0.04961</td><td>0.3174</td><td>0.751</td><td>0.08471</td><td>0.01006</td><td>1.008</td><td>0.01087</td><td>-0.006346</td><td>0.007989</td><td>3mm asymmetry angle (right)</td><td>FALSE</td><td></td><td></td><td></td><td>74038</td><td></td><td></td><td>UK Biobank</td><td>https://docs.google.com/spreadsheets/d/1kvPoupSzsSFBNSztMzl04xMoSC3Kcx3CrjVf4yBmESU/edit?ts=5b5f17db#gid=227859291</td><td>PHESANT Transformation:5108_0|| INTEGER || CONTINUOUS || ||-Notes:This is the asymmetric angle of keratometry results taken at 3mm for the right eye (blank indicates no asymmetry)-Variable type:continuous_raw</td></tr>
<tr><td>5109_raw.txt</td><td>1710174270056F5forCTG.txt.gz</td><td>-0.03035</td><td>0.05507</td><td>-0.5511</td><td>0.5815</td><td>0.1177</td><td>0.01544</td><td>1.008</td><td>0.009622</td><td>0.002001</td><td>0.008299</td><td>6mm asymmetry angle (right)</td><td>FALSE</td><td></td><td></td><td></td><td>41390</td><td></td><td></td><td>UK Biobank</td><td>https://docs.google.com/spreadsheets/d/1kvPoupSzsSFBNSztMzl04xMoSC3Kcx3CrjVf4yBmESU/edit?ts=5b5f17db#gid=227859291</td><td>PHESANT Transformation:5109_0|| INTEGER || CONTINUOUS || ||-Notes:This is the asymmetric angle of keratometry results taken at 6mm for the right eye (blank indicates no asymmetry)-Variable type:continuous_raw</td></tr>
<tr><td>5110_raw.txt</td><td>1710174270056F5forCTG.txt.gz</td><td>0.09808</td><td>0.07097</td><td>1.382</td><td>0.167</td><td>0.06763</td><td>0.01431</td><td>1.03</td><td>0.009563</td><td>-0.01236</td><td>0.008402</td><td>6mm asymmetry angle (left)</td><td>FALSE</td><td></td><td></td><td></td><td>40683</td><td></td><td></td><td>UK Biobank</td><td>https://docs.google.com/spreadsheets/d/1kvPoupSzsSFBNSztMzl04xMoSC3Kcx3CrjVf4yBmESU/edit?ts=5b5f17db#gid=227859291</td><td>PHESANT Transformation:5110_0|| INTEGER || CONTINUOUS || ||-Notes:This is the asymmetric angle of keratometry results taken at 6mm for the left eye (blank indicates no asymmetry)-Variable type:continuous_raw</td></tr>
<tr><td>5111_raw.txt</td><td>1710174270056F5forCTG.txt.gz</td><td>0.03959</td><td>0.05546</td><td>0.714</td><td>0.4752</td><td>0.06699</td><td>0.00933</td><td>1.012</td><td>0.01069</td><td>-0.005049</td><td>0.008009</td><td>3mm asymmetry angle (left)</td><td>FALSE</td><td></td><td></td><td></td><td>73818</td><td></td><td></td><td>UK Biobank</td><td>https://docs.google.com/spreadsheets/d/1kvPoupSzsSFBNSztMzl04xMoSC3Kcx3CrjVf4yBmESU/edit?ts=5b5f17db#gid=227859291</td><td>PHESANT Transformation:5111_0|| INTEGER || CONTINUOUS || ||-Notes:This is the asymmetric angle of keratometry results taken at 3mm for the left eye (blank indicates no asymmetry)-Variable type:continuous_raw</td></tr>
<tr><td>5112_raw.txt</td><td>1710174270056F5forCTG.txt.gz</td><td>-0.04939</td><td>0.05181</td><td>-0.9533</td><td>0.3404</td><td>0.08707</td><td>0.01119</td><td>1.001</td><td>0.01179</td><td>0.01374</td><td>0.008671</td><td>3mm cylindrical power angle (left)</td><td>FALSE</td><td></td><td></td><td></td><td>75389</td><td></td><td></td><td>UK Biobank</td><td>https://docs.google.com/spreadsheets/d/1kvPoupSzsSFBNSztMzl04xMoSC3Kcx3CrjVf4yBmESU/edit?ts=5b5f17db#gid=227859291</td><td>PHESANT Transformation:5112_0|| INTEGER || CONTINUOUS || ||-Notes:This is the cylindrical power angle of keratometry results taken at 3mm for the left eye (blank indicates none)-Variable type:continuous_raw</td></tr>
<tr><td>5113_raw.txt</td><td>1710174270056F5forCTG.txt.gz</td><td>0.03863</td><td>0.04903</td><td>0.788</td><td>0.4307</td><td>0.07799</td><td>0.009701</td><td>0.996</td><td>0.01024</td><td>0.001257</td><td>0.007945</td><td>6mm cylindrical power angle (left)</td><td>FALSE</td><td></td><td></td><td></td><td>65529</td><td></td><td></td><td>UK Biobank</td><td>https://docs.google.com/spreadsheets/d/1kvPoupSzsSFBNSztMzl04xMoSC3Kcx3CrjVf4yBmESU/edit?ts=5b5f17db#gid=227859291</td><td>PHESANT Transformation:5113_0|| INTEGER || CONTINUOUS || ||-Notes:This is the cylindrical power angle of keratometry results taken at 6mm for the left eye (blank indicates none)-Variable type:continuous_raw</td></tr>
<tr><td>5114_raw.txt</td><td>1710174270056F5forCTG.txt.gz</td><td>0.02481</td><td>0.05513</td><td>0.45</td><td>0.6527</td><td>0.07027</td><td>0.01035</td><td>1.003</td><td>0.00984</td><td>-0.008876</td><td>0.008194</td><td>6mm cylindrical power angle (right)</td><td>FALSE</td><td></td><td></td><td></td><td>66237</td><td></td><td></td><td>UK Biobank</td><td>https://docs.google.com/spreadsheets/d/1kvPoupSzsSFBNSztMzl04xMoSC3Kcx3CrjVf4yBmESU/edit?ts=5b5f17db#gid=227859291</td><td>PHESANT Transformation:5114_0|| INTEGER || CONTINUOUS || ||-Notes:This is the cylindrical power angle of keratometry results taken at 6mm for the right eye (blank indicates none)-Variable type:continuous_raw</td></tr>
<tr><td>5115_raw.txt</td><td>1710174270056F5forCTG.txt.gz</td><td>-0.04117</td><td>0.05117</td><td>-0.8045</td><td>0.4211</td><td>0.06854</td><td>0.008947</td><td>1.012</td><td>0.0108</td><td>0.0004464</td><td>0.00808</td><td>3mm cylindrical power angle (right)</td><td>FALSE</td><td></td><td></td><td></td><td>75401</td><td></td><td></td><td>UK Biobank</td><td>https://docs.google.com/spreadsheets/d/1kvPoupSzsSFBNSztMzl04xMoSC3Kcx3CrjVf4yBmESU/edit?ts=5b5f17db#gid=227859291</td><td>PHESANT Transformation:5115_0|| INTEGER || CONTINUOUS || ||-Notes:This is the cylindrical power angle of keratometry results taken at 3mm for the right eye (blank indicates none)-Variable type:continuous_raw</td></tr>
<tr><td>5116_raw.txt</td><td>1710174270056F5forCTG.txt.gz</td><td>-0.001026</td><td>0.05023</td><td>-0.02043</td><td>0.9837</td><td>0.08362</td><td>0.009966</td><td>0.995</td><td>0.01076</td><td>-0.008163</td><td>0.008164</td><td>3mm cylindrical power (right)</td><td>FALSE</td><td></td><td></td><td></td><td>75410</td><td></td><td></td><td>UK Biobank</td><td>https://docs.google.com/spreadsheets/d/1kvPoupSzsSFBNSztMzl04xMoSC3Kcx3CrjVf4yBmESU/edit?ts=5b5f17db#gid=227859291</td><td>PHESANT Transformation:5116_0|| CONTINUOUS MAIN || CONTINUOUS || ||-Notes:This is the cylindrical power of keratometry results taken at 3mm for the right eye (0 indicates none)-Variable type:continuous_raw</td></tr>
<tr><td>5117_raw.txt</td><td>1710174270056F5forCTG.txt.gz</td><td>0.08038</td><td>0.08872</td><td>0.906</td><td>0.365</td><td>0.02693</td><td>0.00812</td><td>1.024</td><td>0.009606</td><td>-0.006529</td><td>0.007972</td><td>6mm cylindrical power (right)</td><td>FALSE</td><td></td><td></td><td></td><td>66256</td><td></td><td></td><td>UK Biobank</td><td>https://docs.google.com/spreadsheets/d/1kvPoupSzsSFBNSztMzl04xMoSC3Kcx3CrjVf4yBmESU/edit?ts=5b5f17db#gid=227859291</td><td>PHESANT Transformation:5117_0|| CONTINUOUS MAIN || CONTINUOUS || ||-Notes:This is the cylindrical power of keratometry results taken at 6mm for the right eye (0 indicates none)-Variable type:continuous_raw</td></tr>
<tr><td>5118_raw.txt</td><td>1710174270056F5forCTG.txt.gz</td><td>0.0494</td><td>0.0725</td><td>0.6814</td><td>0.4956</td><td>0.04239</td><td>0.008596</td><td>1.018</td><td>0.009162</td><td>-0.0112</td><td>0.008035</td><td>6mm cylindrical power (left)</td><td>FALSE</td><td></td><td></td><td></td><td>65551</td><td></td><td></td><td>UK Biobank</td><td>https://docs.google.com/spreadsheets/d/1kvPoupSzsSFBNSztMzl04xMoSC3Kcx3CrjVf4yBmESU/edit?ts=5b5f17db#gid=227859291</td><td>PHESANT Transformation:5118_0|| CONTINUOUS MAIN || CONTINUOUS || ||-Notes:This is the cylindrical power of keratometry results taken at 6mm for the left eye (0 indicates none)-Variable type:continuous_raw</td></tr>
<tr><td>5119_raw.txt</td><td>1710174270056F5forCTG.txt.gz</td><td>0.07156</td><td>0.05298</td><td>1.351</td><td>0.1768</td><td>0.07113</td><td>0.008988</td><td>1.011</td><td>0.009933</td><td>-0.01418</td><td>0.007547</td><td>3mm cylindrical power (left)</td><td>FALSE</td><td></td><td></td><td></td><td>75398</td><td></td><td></td><td>UK Biobank</td><td>https://docs.google.com/spreadsheets/d/1kvPoupSzsSFBNSztMzl04xMoSC3Kcx3CrjVf4yBmESU/edit?ts=5b5f17db#gid=227859291</td><td>PHESANT Transformation:5119_0|| CONTINUOUS MAIN || CONTINUOUS || ||-Notes:This is the cylindrical power of keratometry results taken at 3mm for the left eye (0 indicates none)-Variable type:continuous_raw</td></tr>
<tr><td>5132_raw.txt</td><td>1710174270056F5forCTG.txt.gz</td><td>0.07543</td><td>0.03366</td><td>2.241</td><td>0.02505</td><td>0.3206</td><td>0.02457</td><td>1.086</td><td>0.02467</td><td>-0.003965</td><td>0.009885</td><td>3mm strong meridian (right)</td><td>FALSE</td><td></td><td></td><td></td><td>75410</td><td></td><td></td><td>UK Biobank</td><td>https://docs.google.com/spreadsheets/d/1kvPoupSzsSFBNSztMzl04xMoSC3Kcx3CrjVf4yBmESU/edit?ts=5b5f17db#gid=227859291</td><td>PHESANT Transformation:5132_0|| CONTINUOUS MAIN || CONTINUOUS || ||-Notes:This is the strong meridian angle of keratometry results taken at 3mm for the right eye-Variable type:continuous_raw</td></tr>
<tr><td>5133_raw.txt</td><td>1710174270056F5forCTG.txt.gz</td><td>0.07518</td><td>0.03358</td><td>2.239</td><td>0.02515</td><td>0.3546</td><td>0.02719</td><td>1.078</td><td>0.02394</td><td>-0.00556</td><td>0.009948</td><td>6mm strong meridian (right)</td><td>FALSE</td><td></td><td></td><td></td><td>66256</td><td></td><td></td><td>UK Biobank</td><td>https://docs.google.com/spreadsheets/d/1kvPoupSzsSFBNSztMzl04xMoSC3Kcx3CrjVf4yBmESU/edit?ts=5b5f17db#gid=227859291</td><td>PHESANT Transformation:5133_0|| CONTINUOUS MAIN || CONTINUOUS || ||-Notes:This is the strong meridian angle of keratometry results taken at 6mm for the right eye-Variable type:continuous_raw</td></tr>
<tr><td>5134_raw.txt</td><td>1710174270056F5forCTG.txt.gz</td><td>0.09819</td><td>0.03422</td><td>2.869</td><td>0.004112</td><td>0.3452</td><td>0.02774</td><td>1.088</td><td>0.02335</td><td>-0.01083</td><td>0.01004</td><td>6mm strong meridian (left)</td><td>FALSE</td><td></td><td></td><td></td><td>65551</td><td></td><td></td><td>UK Biobank</td><td>https://docs.google.com/spreadsheets/d/1kvPoupSzsSFBNSztMzl04xMoSC3Kcx3CrjVf4yBmESU/edit?ts=5b5f17db#gid=227859291</td><td>PHESANT Transformation:5134_0|| CONTINUOUS MAIN || CONTINUOUS || ||-Notes:[keratometry@6mm/left] strong meridian-Variable type:continuous_raw</td></tr>
<tr><td>5135_raw.txt</td><td>1710174270056F5forCTG.txt.gz</td><td>0.06852</td><td>0.03369</td><td>2.034</td><td>0.04197</td><td>0.3187</td><td>0.0247</td><td>1.08</td><td>0.02403</td><td>-0.003396</td><td>0.01005</td><td>3mm strong meridian (left)</td><td>FALSE</td><td></td><td></td><td></td><td>75398</td><td></td><td></td><td>UK Biobank</td><td>https://docs.google.com/spreadsheets/d/1kvPoupSzsSFBNSztMzl04xMoSC3Kcx3CrjVf4yBmESU/edit?ts=5b5f17db#gid=227859291</td><td>PHESANT Transformation:5135_0|| CONTINUOUS MAIN || CONTINUOUS || ||-Notes:This is the strong meridian angle of keratometry results taken at 3mm for the left eye-Variable type:continuous_raw</td></tr>
<tr><td>5156_raw.txt</td><td>1710174270056F5forCTG.txt.gz</td><td>-0.02916</td><td>0.09332</td><td>-0.3124</td><td>0.7547</td><td>0.0182</td><td>0.007487</td><td>0.995</td><td>0.009244</td><td>0.009907</td><td>0.0071</td><td>3mm asymmetry index (left)</td><td>FALSE</td><td></td><td></td><td></td><td>73818</td><td></td><td></td><td>UK Biobank</td><td>https://docs.google.com/spreadsheets/d/1kvPoupSzsSFBNSztMzl04xMoSC3Kcx3CrjVf4yBmESU/edit?ts=5b5f17db#gid=227859291</td><td>PHESANT Transformation:5156_0|| CONTINUOUS MAIN || CONTINUOUS || ||-Notes:This is the asymmetry index as part of the keratometry result taken at 3mm for the left eye-Variable type:continuous_raw</td></tr>
<tr><td>5157_raw.txt</td><td>1710174270056F5forCTG.txt.gz</td><td>-0.02891</td><td>0.07313</td><td>-0.3953</td><td>0.6926</td><td>0.06584</td><td>0.01498</td><td>1.032</td><td>0.009975</td><td>0.004707</td><td>0.008146</td><td>6mm asymmetry index (left)</td><td>FALSE</td><td></td><td></td><td></td><td>40683</td><td></td><td></td><td>UK Biobank</td><td>https://docs.google.com/spreadsheets/d/1kvPoupSzsSFBNSztMzl04xMoSC3Kcx3CrjVf4yBmESU/edit?ts=5b5f17db#gid=227859291</td><td>PHESANT Transformation:5157_0|| CONTINUOUS MAIN || CONTINUOUS || ||-Notes:This is the asymmetry index as part of the keratometry result taken at 6mm for the left eye-Variable type:continuous_raw</td></tr>
<tr><td>5158_raw.txt</td><td>1710174270056F5forCTG.txt.gz</td><td>-0.07093</td><td>0.06675</td><td>-1.063</td><td>0.288</td><td>0.07007</td><td>0.01602</td><td>1.011</td><td>0.01084</td><td>0.01334</td><td>0.007692</td><td>6mm asymmetry index (right)</td><td>FALSE</td><td></td><td></td><td></td><td>41390</td><td></td><td></td><td>UK Biobank</td><td>https://docs.google.com/spreadsheets/d/1kvPoupSzsSFBNSztMzl04xMoSC3Kcx3CrjVf4yBmESU/edit?ts=5b5f17db#gid=227859291</td><td>PHESANT Transformation:5158_0|| CONTINUOUS MAIN || CONTINUOUS || ||-Notes:This is the asymmetry index as part of the keratometry result taken at 6mm for the right eye-Variable type:continuous_raw</td></tr>
<tr><td>5159_raw.txt</td><td>1710174270056F5forCTG.txt.gz</td><td>0.09388</td><td>0.08862</td><td>1.059</td><td>0.2894</td><td>0.01801</td><td>0.007934</td><td>0.994</td><td>0.01007</td><td>-0.005775</td><td>0.007877</td><td>3mm asymmetry index (right)</td><td>FALSE</td><td></td><td></td><td></td><td>74038</td><td></td><td></td><td>UK Biobank</td><td>https://docs.google.com/spreadsheets/d/1kvPoupSzsSFBNSztMzl04xMoSC3Kcx3CrjVf4yBmESU/edit?ts=5b5f17db#gid=227859291</td><td>PHESANT Transformation:5159_0|| CONTINUOUS MAIN || CONTINUOUS || ||-Notes:This is the asymmetry index as part of the keratometry result taken at 3mm for the right eye-Variable type:continuous_raw</td></tr>
<tr><td>5160_raw.txt</td><td>1710174270056F5forCTG.txt.gz</td><td>0.001151</td><td>0.06542</td><td>0.01759</td><td>0.986</td><td>0.03463</td><td>0.00855</td><td>1.004</td><td>0.01034</td><td>0.005686</td><td>0.007707</td><td>3mm regularity index (right)</td><td>FALSE</td><td></td><td></td><td></td><td>74059</td><td></td><td></td><td>UK Biobank</td><td>https://docs.google.com/spreadsheets/d/1kvPoupSzsSFBNSztMzl04xMoSC3Kcx3CrjVf4yBmESU/edit?ts=5b5f17db#gid=227859291</td><td>PHESANT Transformation:5160_0|| CONTINUOUS MAIN || CONTINUOUS || ||-Notes:This is the regularity index as part of the keratometry result taken at 3mm for the right eye-Variable type:continuous_raw</td></tr>
<tr><td>5161_raw.txt</td><td>1710174270056F5forCTG.txt.gz</td><td>-0.04442</td><td>0.07971</td><td>-0.5572</td><td>0.5774</td><td>0.05247</td><td>0.01405</td><td>0.998</td><td>0.00953</td><td>0.005482</td><td>0.007997</td><td>6mm regularity index (right)</td><td>FALSE</td><td></td><td></td><td></td><td>41455</td><td></td><td></td><td>UK Biobank</td><td>https://docs.google.com/spreadsheets/d/1kvPoupSzsSFBNSztMzl04xMoSC3Kcx3CrjVf4yBmESU/edit?ts=5b5f17db#gid=227859291</td><td>PHESANT Transformation:5161_0|| CONTINUOUS MAIN || CONTINUOUS || ||-Notes:This is the regularity index as part of the keratometry result taken at 6mm for the right eye-Variable type:continuous_raw</td></tr>
<tr><td>5162_raw.txt</td><td>1710174270056F5forCTG.txt.gz</td><td>-0.1738</td><td>0.1037</td><td>-1.676</td><td>0.09383</td><td>0.02967</td><td>0.01427</td><td>1.005</td><td>0.009532</td><td>0.01675</td><td>0.007959</td><td>6mm regularity index (left)</td><td>FALSE</td><td></td><td></td><td></td><td>40700</td><td></td><td></td><td>UK Biobank</td><td>https://docs.google.com/spreadsheets/d/1kvPoupSzsSFBNSztMzl04xMoSC3Kcx3CrjVf4yBmESU/edit?ts=5b5f17db#gid=227859291</td><td>PHESANT Transformation:5162_0|| CONTINUOUS MAIN || CONTINUOUS || ||-Notes:This is the regularity index as part of the keratometry result taken at 6mm for the left eye-Variable type:continuous_raw</td></tr>
<tr><td>5163_raw.txt</td><td>1710174270056F5forCTG.txt.gz</td><td>0.009855</td><td>0.1085</td><td>0.09083</td><td>0.9276</td><td>0.01377</td><td>0.007836</td><td>1.009</td><td>0.009512</td><td>0.009392</td><td>0.007811</td><td>3mm regularity index (left)</td><td>FALSE</td><td></td><td></td><td></td><td>73829</td><td></td><td></td><td>UK Biobank</td><td>https://docs.google.com/spreadsheets/d/1kvPoupSzsSFBNSztMzl04xMoSC3Kcx3CrjVf4yBmESU/edit?ts=5b5f17db#gid=227859291</td><td>PHESANT Transformation:5163_0|| CONTINUOUS MAIN || CONTINUOUS || ||-Notes:This is the regularity index as part of the keratometry result taken at 3mm for the left eye-Variable type:continuous_raw</td></tr>
<tr><td>5201_raw.txt</td><td>1710174270056F5forCTG.txt.gz</td><td>0.3829</td><td>0.134</td><td>2.858</td><td>0.004259</td><td>0.01507</td><td>0.007856</td><td>1.014</td><td>0.009592</td><td>-0.01096</td><td>0.007942</td><td>logMAR, final (right)</td><td>FALSE</td><td></td><td></td><td></td><td>79293</td><td></td><td></td><td>UK Biobank</td><td>https://docs.google.com/spreadsheets/d/1kvPoupSzsSFBNSztMzl04xMoSC3Kcx3CrjVf4yBmESU/edit?ts=5b5f17db#gid=227859291</td><td>PHESANT Transformation:5201_0|| CONTINUOUS MAIN || CONTINUOUS || ||-Notes:Visual acuity (right), final logMAR-Variable type:continuous_raw</td></tr>
<tr><td>5208_raw.txt</td><td>1710174270056F5forCTG.txt.gz</td><td>0.1962</td><td>0.08157</td><td>2.405</td><td>0.01616</td><td>0.02373</td><td>0.007557</td><td>1.008</td><td>0.009829</td><td>-0.003465</td><td>0.007701</td><td>logMAR, final (left)</td><td>FALSE</td><td></td><td></td><td></td><td>79239</td><td></td><td></td><td>UK Biobank</td><td>https://docs.google.com/spreadsheets/d/1kvPoupSzsSFBNSztMzl04xMoSC3Kcx3CrjVf4yBmESU/edit?ts=5b5f17db#gid=227859291</td><td>PHESANT Transformation:5208_0|| CONTINUOUS MAIN || CONTINUOUS || ||-Notes:Visual acuity (left), final logMAR-Variable type:continuous_raw</td></tr>
<tr><td>5254_raw.txt</td><td>1710174270056F5forCTG.txt.gz</td><td>-0.1109</td><td>0.04767</td><td>-2.326</td><td>0.02002</td><td>0.09752</td><td>0.01087</td><td>1.032</td><td>0.01325</td><td>0.003512</td><td>0.008739</td><td>Intra-ocular pressure, corneal-compensated (right)</td><td>FALSE</td><td></td><td></td><td></td><td>76630</td><td></td><td></td><td>UK Biobank</td><td>https://docs.google.com/spreadsheets/d/1kvPoupSzsSFBNSztMzl04xMoSC3Kcx3CrjVf4yBmESU/edit?ts=5b5f17db#gid=227859291</td><td>PHESANT Transformation:5254_0|| CONTINUOUS MAIN || CONTINUOUS || ||-Notes:corneal-compensated intraocular pressure for right eye-Variable type:continuous_raw</td></tr>
<tr><td>5255_raw.txt</td><td>1710174270056F5forCTG.txt.gz</td><td>-0.05561</td><td>0.04258</td><td>-1.306</td><td>0.1916</td><td>0.1689</td><td>0.01504</td><td>1.057</td><td>0.01801</td><td>0.004303</td><td>0.009561</td><td>Intra-ocular pressure, Goldmann-correlated (right)</td><td>FALSE</td><td></td><td></td><td></td><td>76630</td><td></td><td></td><td>UK Biobank</td><td>https://docs.google.com/spreadsheets/d/1kvPoupSzsSFBNSztMzl04xMoSC3Kcx3CrjVf4yBmESU/edit?ts=5b5f17db#gid=227859291</td><td>PHESANT Transformation:5255_0|| CONTINUOUS MAIN || CONTINUOUS || ||-Notes:goldmann-correlated intraocular pressure for right eye-Variable type:continuous_raw</td></tr>
<tr><td>5256_raw.txt</td><td>1710174270056F5forCTG.txt.gz</td><td>0.06112</td><td>0.04027</td><td>1.518</td><td>0.1291</td><td>0.1219</td><td>0.01301</td><td>1.04</td><td>0.01633</td><td>0.004135</td><td>0.008739</td><td>Corneal hysteresis (right)</td><td>FALSE</td><td></td><td></td><td></td><td>76630</td><td></td><td></td><td>UK Biobank</td><td>https://docs.google.com/spreadsheets/d/1kvPoupSzsSFBNSztMzl04xMoSC3Kcx3CrjVf4yBmESU/edit?ts=5b5f17db#gid=227859291</td><td>PHESANT Transformation:5256_0|| CONTINUOUS MAIN || CONTINUOUS || ||-Notes:corneal hysteresis for right eye (a measure of viscuous damping in the cornea)-Variable type:continuous_raw</td></tr>
<tr><td>5257_raw.txt</td><td>1710174270056F5forCTG.txt.gz</td><td>0.0177</td><td>0.03932</td><td>0.4501</td><td>0.6527</td><td>0.1601</td><td>0.01692</td><td>1.057</td><td>0.02096</td><td>0.005057</td><td>0.009223</td><td>Corneal resistance factor (right)</td><td>FALSE</td><td></td><td></td><td></td><td>76630</td><td></td><td></td><td>UK Biobank</td><td>https://docs.google.com/spreadsheets/d/1kvPoupSzsSFBNSztMzl04xMoSC3Kcx3CrjVf4yBmESU/edit?ts=5b5f17db#gid=227859291</td><td>PHESANT Transformation:5257_0|| CONTINUOUS MAIN || CONTINUOUS || ||-Notes:corneal resistance factor for right eye-Variable type:continuous_raw</td></tr>
<tr><td>5262_raw.txt</td><td>1710174270056F5forCTG.txt.gz</td><td>-0.08735</td><td>0.04522</td><td>-1.932</td><td>0.05341</td><td>0.1105</td><td>0.01204</td><td>1.03</td><td>0.01349</td><td>-0.002953</td><td>0.008649</td><td>Intra-ocular pressure, corneal-compensated (left)</td><td>FALSE</td><td></td><td></td><td></td><td>76510</td><td></td><td></td><td>UK Biobank</td><td>https://docs.google.com/spreadsheets/d/1kvPoupSzsSFBNSztMzl04xMoSC3Kcx3CrjVf4yBmESU/edit?ts=5b5f17db#gid=227859291</td><td>PHESANT Transformation:5262_0|| CONTINUOUS MAIN || CONTINUOUS || ||-Notes:corneal-compensated intraocular pressure for left eye-Variable type:continuous_raw</td></tr>
</table>

<table>
<tr><th></th><th></th><th></th><th></th><th></th><th></th><th></th><th></th><th></th><th></th><th></th><th></th><th></th><th></th><th></th><th></th><th></th><th></th><th></th><th></th><th></th><th></th><th></th></tr>
<tr><td>5263_raw.txt</td><td>1710174270056F5forCTG.txt.gz</td><td>-0.03999</td><td>0.03969</td><td>-1.008</td><td>0.3136</td><td>0.1822</td><td>0.01485</td><td>1.044</td><td>0.01749</td><td>-0.002929</td><td>0.00954</td><td>Intra-ocular pressure, Goldmann-correlated (left)</td><td>FALSE</td><td></td><td></td><td></td><td>76510</td><td></td><td></td><td>UK Biobank</td><td>https://docs.google.com/spreadsheets/d/1kvPoupSzsSFBNSztMzl04xMoSC3Kcx3CrjVf4yBmESU/edit?ts=5b5f17db#gid=227859291</td><td>PHESANT Transformation:5263_0| | CONTINUOUS MAIN | | CONTINUOUS | | | |-Notes:goldmann-correlated intraocular pressure for left eye-Variable type:continuous_raw</td></tr>
<tr><td>5264_raw.txt</td><td>1710174270056F5forCTG.txt.gz</td><td>0.04338</td><td>0.0465</td><td>0.9328</td><td>0.3509</td><td>0.1028</td><td>0.01314</td><td>1.03</td><td>0.01451</td><td>0.00899</td><td>0.007728</td><td>Corneal hysteresis (left)</td><td>FALSE</td><td></td><td></td><td></td><td>76510</td><td></td><td></td><td>UK Biobank</td><td>https://docs.google.com/spreadsheets/d/1kvPoupSzsSFBNSztMzl04xMoSC3Kcx3CrjVf4yBmESU/edit?ts=5b5f17db#gid=227859291</td><td>PHESANT Transformation:5264_0| | CONTINUOUS MAIN | | CONTINUOUS | | | |-Notes:corneal hysteresis for left eye (a measure of viscuous damping in the cornea)-Variable type:continuous_raw</td></tr>
<tr><td>5265_raw.txt</td><td>1710174270056F5forCTG.txt.gz</td><td>0.01323</td><td>0.04325</td><td>0.306</td><td>0.7596</td><td>0.1444</td><td>0.01594</td><td>1.036</td><td>0.01799</td><td>0.005149</td><td>0.008709</td><td>Corneal resistance factor (left)</td><td>FALSE</td><td></td><td></td><td></td><td>76510</td><td></td><td></td><td>UK Biobank</td><td>https://docs.google.com/spreadsheets/d/1kvPoupSzsSFBNSztMzl04xMoSC3Kcx3CrjVf4yBmESU/edit?ts=5b5f17db#gid=227859291</td><td>PHESANT Transformation:5265_0| | CONTINUOUS MAIN | | CONTINUOUS | | | |-Notes:corneal resistance factor for left eye-Variable type:continuous_raw</td></tr>
<tr><td>5983_raw.txt</td><td>1710174270056F5forCTG.txt.gz</td><td>-0.02171</td><td>0.05626</td><td>-0.386</td><td>0.6995</td><td>0.08034</td><td>0.01293</td><td>1.026</td><td>0.012</td><td>-0.006597</td><td>0.008318</td><td>ECG, heart rate</td><td>FALSE</td><td></td><td></td><td></td><td>53777</td><td></td><td></td><td>UK Biobank</td><td>https://docs.google.com/spreadsheets/d/1kvPoupSzsSFBNSztMzl04xMoSC3Kcx3CrjVf4yBmESU/edit?ts=5b5f17db#gid=227859291</td><td>PHESANT Transformation:5983_0| | INTEGER | | CONTINUOUS | | | |-Notes:ECG, heart rate The Bike Test consists of many phases. A phase is generally divided into number of stages. At various points during the test, called trends, readings about heart rate, workload etc are recorded. This field contains heart rate (beats per minute) at the time of the trend entry-Variable type:continuous_raw</td></tr>
<tr><td>5984_raw.txt</td><td>1710174270056F5forCTG.txt.gz</td><td>-0.3605</td><td>0.09009</td><td>-4.002</td><td>6.29E-05</td><td>0.03644</td><td>0.01145</td><td>1.016</td><td>0.009698</td><td>-0.003102</td><td>0.00867</td><td>ECG, load</td><td>FALSE</td><td></td><td></td><td></td><td>53843</td><td></td><td></td><td>UK Biobank</td><td>https://docs.google.com/spreadsheets/d/1kvPoupSzsSFBNSztMzl04xMoSC3Kcx3CrjVf4yBmESU/edit?ts=5b5f17db#gid=227859291</td><td>PHESANT Transformation:5984_0| | INTEGER | | CONTINUOUS | | | |-Notes:ECG, load The Bike Test consists of many phases. A phase is generally divided into number of stages. At various points during the test, called trends, readings about heart rate, workload etc are recorded. This field contains load in Watts at the time of the trend entry.-Variable type:continuous_raw</td></tr>
<tr><td>5986_raw.txt</td><td>1710174270056F5forCTG.txt.gz</td><td>-0.3571</td><td>0.1097</td><td>-3.255</td><td>0.001135</td><td>0.02736</td><td>0.01103</td><td>1.011</td><td>0.009295</td><td>-0.007421</td><td>0.008392</td><td>ECG, phase time</td><td>FALSE</td><td></td><td></td><td></td><td>53998</td><td></td><td></td><td>UK Biobank</td><td>https://docs.google.com/spreadsheets/d/1kvPoupSzsSFBNSztMzl04xMoSC3Kcx3CrjVf4yBmESU/edit?ts=5b5f17db#gid=227859291</td><td>PHESANT Transformation:5986_0| | INTEGER | | CONTINUOUS | | | |-Notes:ECG, phase time The Bike Test consists of many phases. A phase is generally divided into number of stages. At various points during the test, called trends, readings about heart rate, workload etc are recorded. This field contains the time spent within the phase of the trend entry.-Variable type:continuous_raw</td></tr>
<tr><td>AAA_Bothsex_eur_inv_var_meta_GBMI_052021_nbbkgt1.txt.gz.ctgvl</td><td>1710174270056F5forCTG.txt.gz</td><td>0.07975</td><td>0.05032</td><td>1.585</td><td>0.113</td><td>0.004578</td><td>0.000665</td><td>1.013</td><td>0.01222</td><td>-0.002628</td><td>0.008589</td><td>Abdominal aortic aneurysm (AAA) (EUR Biobanks)</td><td>FALSE</td><td></td><td></td><td></td><td>1264918</td><td>8163</td><td>1256755</td><td>Global Biobank Meta-analysis Initiative Meta-analysis Initiative: powering genetic discovery across human diseases. 2022</td><td>https://www.globalbiobankmeta.org/resources</td><td>https://www.sciencedirect.com/science/article/pii/S2666979X22001410?via%3Dihub</td></tr>
<tr><td>AAA_Bothsex_inv_var_meta_GBMI_052021_nbbkgt1.txt.gz.ctgvl</td><td>1710174270056F5forCTG.txt.gz</td><td>0.07666</td><td>0.04769</td><td>1.608</td><td>0.1079</td><td>0.004547</td><td>0.000576</td><td>1.012</td><td>0.01216</td><td>0.0007421</td><td>0.008648</td><td>Abdominal aortic aneurysm (AAA) (All Biobanks)</td><td>FALSE</td><td></td><td></td><td></td><td>1455875</td><td>9453</td><td>1446422</td><td>Global Biobank Meta-analysis Initiative Meta-analysis Initiative: powering genetic discovery across human diseases. 2023</td><td>https://www.globalbiobankmeta.org/resources</td><td>https://www.sciencedirect.com/science/article/pii/S2666979X22001410?via%3Dihub</td></tr>
<tr><td>AcApp_Bothsex_eur_inv_var_meta_GBMI_052021_nbbkgt1.txt.gz.ctgvl</td><td>1710174270056F5forCTG.txt.gz</td><td>0.1548</td><td>0.06191</td><td>2.501</td><td>0.01239</td><td>0.003941</td><td>0.000695</td><td>1.026</td><td>0.01221</td><td>0.00645</td><td>0.008044</td><td>Acute appendicitis (AcApp) (EUR Biobanks)</td><td>FALSE</td><td></td><td></td><td></td><td>1010222</td><td>31714</td><td>978508</td><td>Global Biobank Meta-analysis Initiative Meta-analysis Initiative: powering genetic discovery across human diseases. 2024</td><td>https://www.globalbiobankmeta.org/resources</td><td>https://www.sciencedirect.com/science/article/pii/S2666979X22001410?via%3Dihub</td></tr>
<tr><td>AcApp_Bothsex_inv_var_meta_GBMI_052021_nbbkgt1.txt.gz.ctgvl</td><td>1710174270056F5forCTG.txt.gz</td><td>0.161</td><td>0.06234</td><td>2.582</td><td>0.009819</td><td>0.003475</td><td>0.000632</td><td>1.03</td><td>0.0121</td><td>0.006252</td><td>0.007868</td><td>Acute appendicitis (AcApp) (All Biobanks)</td><td>FALSE</td><td></td><td></td><td></td><td>1108469</td><td>32706</td><td>1075763</td><td>Global Biobank Meta-analysis Initiative Meta-analysis Initiative: powering genetic discovery across human diseases. 2025</td><td>https://www.globalbiobankmeta.org/resources</td><td>https://www.sciencedirect.com/science/article/pii/S2666979X22001410?via%3Dihub</td></tr>
<tr><td>adhd_jul2017.ctgvl</td><td>1710174270056F5forCTG.txt.gz</td><td>0.7364</td><td>0.03686</td><td>19.98</td><td>9.04E-89</td><td>0.1344</td><td>0.01196</td><td>1.026</td><td>0.01108</td><td>0.3049</td><td>0.009324</td><td>ADHD</td><td>FALSE</td><td>Psychiatric</td><td></td><td></td><td>55374</td><td>20183</td><td>35191</td><td>PGC</td><td>https://www.med.unc.edu/pgc/results-and-downloads/</td><td>GWAS made available by PGC. Please go to reference for details</td></tr>
<tr><td>Asthma_Bothsex_eur_inv_var_meta_GBMI_052021_nbbkgt1.txt.gz.ctgvl</td><td>1710174270056F5forCTG.txt.gz</td><td>0.2816</td><td>0.03367</td><td>8.363</td><td>6.14E-17</td><td>0.02562</td><td>0.002334</td><td>1.073</td><td>0.03263</td><td>0.03405</td><td>0.01007</td><td>Asthma (EUR Biobanks)</td><td>TRUE</td><td>Pulmonary</td><td>Physical health</td><td>Asthma</td><td>1376071</td><td>121940</td><td>1254131</td><td>Global Biobank Meta-analysis Initiative Meta-analysis Initiative: powering genetic discovery across human diseases. 2026</td><td>https://www.globalbiobankmeta.org/resources</td><td>https://www.sciencedirect.com/science/article/pii/S2666979X22001410?via%3Dihub</td></tr>
<tr><td>Asthma_Bothsex_inv_var_meta_GBMI_052021_nbbkgt1.txt.gz.ctgvl</td><td>1710174270056F5forCTG.txt.gz</td><td>0.2613</td><td>0.0316</td><td>8.27</td><td>1.35E-16</td><td>0.02186</td><td>0.001927</td><td>1.079</td><td>0.03458</td><td>0.03367</td><td>0.009862</td><td>Asthma (All Biobanks)</td><td>FALSE</td><td>Pulmonary</td><td></td><td></td><td>1800785</td><td>153763</td><td>1647022</td><td>Global Biobank Meta-analysis Initiative Meta-analysis Initiative: powering genetic discovery across human diseases. 2027</td><td>https://www.globalbiobankmeta.org/resources</td><td>https://www.sciencedirect.com/science/article/pii/S2666979X22001410?via%3Dihub</td></tr>
<tr><td>binary.100580.txt</td><td>1710174270056F5forCTG.txt.gz</td><td>-0.2405</td><td>0.09717</td><td>-2.475</td><td>0.01334</td><td>0.02991</td><td>0.01178</td><td>1.004</td><td>0.009856</td><td>-0.002585</td><td>0.007323</td><td>Alcohol consumed</td><td>FALSE</td><td></td><td></td><td></td><td>51427</td><td>25436</td><td>25991</td><td>UK Biobank</td><td>https://docs.google.com/spreadsheets/d/1kvPoupSzsSFBNSztMzl04xMoSC3Kcx3CrjVf4yBmESU/edit?ts=5b5f17db#gid=227859291</td><td>PHESANT Transformation:100580_0| | CAT-SINGLE | | Inc(>=10): 1(25436) | | Inc(>=10): 0(25991) | | CAT-SINGLE-BINARY | | sample 25991/25436(51427) | |-Notes:Question asked: Did you have any alcoholic drinks yesterday? For instance, beer, wine or spirits. If the participant activated the Help feature they were shown the message: Please treat liqueurs as spirits. If you had mixed drinks such as shandy, cocktails or alcopops, think about what went in them. For example, shandy is made from beer, lager or cider (please record the lemonade or ginger beer used in the shandy separately); cocktails and alcopops contain one or more spirits. Alcohol used in cooking should be ignored.-Variable type:binary</td></tr>
<tr><td>binary.100760.txt</td><td>1710174270056F5forCTG.txt.gz</td><td>-0.299</td><td>0.09669</td><td>-3.093</td><td>0.001984</td><td>0.03002</td><td>0.01129</td><td>1.018</td><td>0.01047</td><td>0.002347</td><td>0.007661</td><td>Breakfast cereal consumed</td><td>FALSE</td><td></td><td></td><td></td><td>51427</td><td>34069</td><td>17358</td><td>UK Biobank</td><td>https://docs.google.com/spreadsheets/d/1kvPoupSzsSFBNSztMzl04xMoSC3Kcx3CrjVf4yBmESU/edit?ts=5b5f17db#gid=227859291</td><td>PHESANT Transformation:100760_0| | CAT-SINGLE | | Inc(>=10): 1(34069) | | Inc(>=10): 0(17358) | | CAT-SINGLE-BINARY | | sample 17358/34069(51427) | |-Notes:Question asked: Did you eat any breakfast cereal yesterday? This could be at any time of the day. Please include hot cereals, but not cereal bars. If the participant activated the Help feature they were shown the message: If you had yogurt on your cereal (instead of milk), please record this later in the dessert section as a serving of yogurt. Two standard size shredded wheat or weetabix biscuits counts as one bowl. Please select the option that best describes your type of cereal, otherwise select the Other category.-Variable type:binary</td></tr>
</table>

binary.100890.txt	1710174270056F5forCTG.txt.gz	-0.3194	0.1133	-2.82	0.0048	0.02149	0.01058	1.01	0.009781	-0.009343	0.007519	Milk added to cereal	FALSE				51427	22510	28917	UK Biobank	https://docs.google.com/spreadsheets/d/1kvPoupSzsSFBNSztMzl04xMoSC3Kcx3CrjVf4yBmESU/edit?ts=5b5f17db#gid=227859291	PHESANT Transformation:100890_0|| CAT-SINGLE || reassignments: 111=NA || reorder 111|1 || Default related field: x20082_0_0 || default value 0 set, N= 22510 || Inc(>=10): 2(28917) || Inc(>=10): 0(22510) || CAT-SINGLE-BINARY || sample 22510/28917(51427) ||-Notes:Question asked: Did you add milk to your cereal? This question was only asked to participants who reported consuming breakfast cereals yesterday.-Variable type:binary
binary.100920_2102.txt	1710174270056F5forCTG.txt.gz	-0.2072	0.09199	-2.253	0.02428	0.02412	0.01063	0.992	0.009182	-0.000246	0.007441	Type milk consumed: semiskimmed	FALSE				51427	31930	19497	UK Biobank	https://docs.google.com/spreadsheets/d/1kvPoupSzsSFBNSztMzl04xMoSC3Kcx3CrjVf4yBmESU/edit?ts=5b5f17db#gid=227859291	PHESANT Transformation:100920_0 || CAT-SINGLE || CAT-SINGLE-BINARY-VAR: 2102 || Inc(>=10): 2102(31930) ||-Notes:Question asked: Which type of milk did you use most frequently yesterday? Remember milk in drinks, on cereal and in cooking. If the participant activated the Help feature they were shown the message: If you typically use more than one type of milk, please select the one you use the most, even if it is only used slightly more than other types. If you had 1% fat milk (e.g. Sainsbury's orange top milk) then please select Semi-skimmed milk.-Variable type:binary
binary.100920_2104.txt	1710174270056F5forCTG.txt.gz	-0.01402	0.08781	-0.1597	0.8731	0.03205	0.01048	0.989	0.009385	0.001728	0.008436	Type milk consumed: wholemilk	FALSE				51427	2934	48493	UK Biobank	https://docs.google.com/spreadsheets/d/1kvPoupSzsSFBNSztMzl04xMoSC3Kcx3CrjVf4yBmESU/edit?ts=5b5f17db#gid=227859291	PHESANT Transformation:100920_0 || CAT-SINGLE || CAT-SINGLE-BINARY-VAR: 2104 || Inc(>=10): 2104(2934) ||-Notes:Question asked: Which type of milk did you use most frequently yesterday? Remember milk in drinks, on cereal and in cooking. If the participant activated the Help feature they were shown the message: If you typically use more than one type of milk, please select the one you use the most, even if it is only used slightly more than other types. If you had 1% fat milk (e.g. Sainsbury's orange top milk) then please select Semi-skimmed milk.-Variable type:binary
binary.102080.txt	1710174270056F5forCTG.txt.gz	-0.03579	0.08042	-0.4451	0.6563	0.03842	0.01205	0.998	0.01128	-0.01144	0.008107	Yogurt/ice-cream consumers	FALSE				51427	20803	30624	UK Biobank	https://docs.google.com/spreadsheets/d/1kvPoupSzsSFBNSztMzl04xMoSC3Kcx3CrjVf4yBmESU/edit?ts=5b5f17db#gid=227859291	PHESANT Transformation:102080_0|| CAT-SINGLE || Inc(>=10): 0(30624) || Inc(>=10): 1(20803) || CAT-SINGLE-BINARY || sample 30624/20803(51427) ||-Notes:Question asked: Did you eat any yogurt or ice-cream yesterday?-Variable type:binary
binary.102250.txt	1710174270056F5forCTG.txt.gz	-0.07874	0.1121	-0.7027	0.4822	0.0208	0.01062	1.015	0.009374	-0.01118	0.007883	Sweet snack consumers	FALSE				51427	27903	23524	UK Biobank	https://docs.google.com/spreadsheets/d/1kvPoupSzsSFBNSztMzl04xMoSC3Kcx3CrjVf4yBmESU/edit?ts=5b5f17db#gid=227859291	PHESANT Transformation:102250_0|| CAT-SINGLE || Inc(>=10): 0(23524) || Inc(>=10): 1(27903) || CAT-SINGLE-BINARY || sample 23524/27903(51427) ||-Notes:Question asked: Did you eat any biscuits, chocolate or sweets yesterday? Also includes sweets, cereal bars, chocolate covered raisins, sweet popcorn and other sweet snacks. If the participant activated the Help feature they were shown the message: Select which category best describes your type of sweet snack food, otherwise select the Other category.-Variable type:binary
binary.102700.txt	1710174270056F5forCTG.txt.gz	0.0141	0.09763	0.1445	0.8851	0.02177	0.01059	1	0.009047	-0.01248	0.008075	Starchy food consumers	FALSE				51427	13706	37721	UK Biobank	https://docs.google.com/spreadsheets/d/1kvPoupSzsSFBNSztMzl04xMoSC3Kcx3CrjVf4yBmESU/edit?ts=5b5f17db#gid=227859291	PHESANT Transformation:102700_0|| CAT-SINGLE || Inc(>=10): 0(37721) || Inc(>=10): 1(13706) || CAT-SINGLE-BINARY || sample 37721/13706(51427) ||-Notes:Question asked: Did you eat any pasta, rice, sushi or couscous yesterday? This includes noodles, lasagne pasta sheets and other cooked grains such as bulgar wheat. This can accompany a meal, or be a meal in itself. It may be hot or cold.-Variable type:binary
binary.102800.txt	1710174270056F5forCTG.txt.gz	-0.04434	0.09873	-0.4491	0.6533	0.02738	0.01012	0.995	0.008492	-0.0119	0.008361	Cheese consumers	FALSE				51427	23245	28182	UK Biobank	https://docs.google.com/spreadsheets/d/1kvPoupSzsSFBNSztMzl04xMoSC3Kcx3CrjVf4yBmESU/edit?ts=5b5f17db#gid=227859291	PHESANT Transformation:102800_0|| CAT-SINGLE || Inc(>=10): 0(28182) || Inc(>=10): 1(23245) || CAT-SINGLE-BINARY || sample 28182/23245(51427) ||-Notes:Question asked: Did you eat any cheese yesterday? Cheese in sandwiches, on burgers, on jacket potato, pasta dishes. If the participant activated the Help feature they were shown the message: Please do not include cheese sauce as this will be asked later. A guide to servings: 1 serving of cheese - a chunk about the size of a small matchbox; 1 serving of grated cheese - about a handful or a large spoonful; 1 serving of spreadable cheese - the amount spread onto a typical slice of square sandwich bread.-Variable type:binary
binary.103990.txt	1710174270056F5forCTG.txt.gz	-0.2297	0.1171	-1.962	0.04973	0.02157	0.01222	1.002	0.01021	-0.004662	0.007734	Vegetable consumers	FALSE				51427	42359	9068	UK Biobank	https://docs.google.com/spreadsheets/d/1kvPoupSzsSFBNSztMzl04xMoSC3Kcx3CrjVf4yBmESU/edit?ts=5b5f17db#gid=227859291	PHESANT Transformation:103990_0|| CAT-SINGLE || Inc(>=10): 1(42359) || Inc(>=10): 0(9068) || CAT-SINGLE-BINARY || sample 9068/42359(51427) ||-Notes:Question asked: Did you eat any beans, lentils, potatoes or vegetables yesterday? Include fresh, tinned, frozen, dried; chips, salad, coleslaw, baked beans, chickpeas, veg in stews, pies etc. If the participant activated the Help feature they were shown the message: Please include all vegetables, whether they are eaten by themselves, as an accompaniment or within a particular dish/meal such as a stew or pie. A serving of vegetables would be the typical amount dished up as an accompaniment to main meal. e.g. if you had a roast dinner with meat, potatoes, carrots and peas, most people would have one serving of potatoes, one serving of carrots and one serving of peas. For small amounts of vegetables that are not big enough to be counted individually (e.g. in a stew), please guess the number of servings and record that under Vegetable pieces. A serving is about the amount that would be dished up using a serving spoon (large spoon typically used in canteens or self-service buffets). Example: a beef stew with carrot and parsnip may have enough vegetables in it to count as a half serving of carrots plus a half serving of parsnip (possibly even a whole serving if there are a lot of vegetables in the stew). If not, it may be better to record the vegetables as a half serving of Vegetable pieces. For a selection of vegetables where the precise type may be unknown (for example in a shop bought vegetable pie or a stir fry cooked by a friend) please guess the total amount and record that under Vegetable pieces. Olives should have been recorded earlier under 'Savoury Snacks'. If your vegetable is not on the list, please record it under 'Other' located at the bottom of the list.-Variable type:binary
binary.104400.txt	1710174270056F5forCTG.txt.gz	-0.2464	0.08503	-2.898	0.003752	0.03952	0.011	1.002	0.008736	0.00164	0.007859	Fruit consumers	FALSE				51427	41921	9506	UK Biobank	https://docs.google.com/spreadsheets/d/1kvPoupSzsSFBNSztMzl04xMoSC3Kcx3CrjVf4yBmESU/edit?ts=5b5f17db#gid=227859291	PHESANT Transformation:104400_0|| CAT-SINGLE || Inc(>=10): 1(41921) || Inc(>=10): 0(9506) || CAT-SINGLE-BINARY || sample 9506/41921(51427) ||-Notes:Question asked: Did you eat any fruit yesterday? If the participant activated the Help feature they were shown the message: Include fresh, frozen, dried, canned or cooked. Help section: Please include all fruit, whether eaten by itself or as part of a sweet or savoury dish (e.g. fruit pie or crumble; pork cooked with apple). Fruits recorded under cooked & dried should not be recorded individually further down the list. Small amounts of fruit that are not big enough to be counted individually, or where the precise type may be unknown (e.g. in a mixed fruit salad), please guess the total amount and record that under Mixed fruit. As canned fruit usually comes in segments or slices, please give your answer in servings rather than as whole fruit. Fruit used in a smoothie should not be recorded here. The fruit smoothie should have been recorded in the drinks section. Chocolate covered fruit (e.g. chocolate raisins) and yogurt covered fruit (e.g. yogurt coated pineapple) should not be recorded here. Instead record it under sweet snacks (several pages earlier). Dried fruit that is included with cereals such as Sultana Bran and Optivita should not be record here because it should have been recorded on the cereals page. If your fruit is not on the list, please record it under Other located at the bottom of the list.-Variable type:binary
binary.104670.txt	1710174270056F5forCTG.txt.gz	-0.02456	0.09924	-0.2475	0.8045	0.03027	0.01085	0.999	0.009695	-0.001045	0.00837	Vitamin supplement user	FALSE				51427	21119	30308	UK Biobank	https://docs.google.com/spreadsheets/d/1kvPoupSzsSFBNSztMzl04xMoSC3Kcx3CrjVf4yBmESU/edit?ts=5b5f17db#gid=227859291	PHESANT Transformation:104670_0|| CAT-SINGLE || Inc(>=10): 1(21119) || Inc(>=10): 0(30308) || CAT-SINGLE-BINARY || sample 30308/21119(51427) ||-Notes:Question asked: Did you have any vitamin or mineral supplements yesterday? e.g. Vitamin C, multivitamins, fish oil, calcium supplement. If the participant activated the Help feature they were shown the message: Select which category best describes your type of vitamin and/or mineral supplement, otherwise select the Other category.-Variable type:binary
binary.110001.txt	1710174270056F5forCTG.txt.gz	-0.3655	0.04277	-8.547	1.27E-17	0.03495	0.003249	1.022	0.01135	0.01439	0.007864	Invitation to complete online 24-hour recall dietary questionnaire, acceptance	FALSE	Other			218234	148169	70065	UK Biobank	https://docs.google.com/spreadsheets/d/1kvPoupSzsSFBNSztMzl04xMoSC3Kcx3CrjVf4yBmESU/edit?ts=5b5f17db#gid=227859291	PHESANT Transformation:110001_1|| CAT-SINGLE || Inc(>=10): 2(70065) || Inc(>=10): 0(148169) || CAT-SINGLE-BINARY || sample 148169/70065(218234) ||-Notes:Response to emailed invitation to complete online 24-hour recall dietary questionnaire. There is no data for instance=0 since this was completed as part of the initial assessment centre.-Variable type:binary
binary.1150_1.txt	1710174270056F5forCTG.txt.gz	-0.2388	0.04863	-4.91	9.09E-07	0.02246	0.00308	1.004	0.01252	0.0004868	0.00881	Usual side of head for mobile phone use: Left	FALSE	Other			303009	107802	195207	UK Biobank	https://docs.google.com/spreadsheets/d/1kvPoupSzsSFBNSztMzl04xMoSC3Kcx3CrjVf4yBmESU/edit?ts=5b5f17db#gid=227859291	PHESANT Transformation:1150_0 || CAT-SINGLE || CAT-SINGLE-BINARY-VAR: 1 || Inc(>=10): 1(107802) ||-Notes:ACE touchscreen question On what side of the head do you usually use a mobile phone? If the participant activated the Help button they were shown the message: If you are unsure, please provide an estimate or select Do not know. ~F1150~ was collected from participants who indicated they have used a mobile phone at least once per week in the past or do not know whether they have used or not, as defined by their answers to ~F1110~-Variable type:binary
binary.1150_2.txt	1710174270056F5forCTG.txt.gz	0.1313	0.04376	3	0.002698	0.02671	0.003162	1.002	0.0131	-0.005522	0.008747	Usual side of head for mobile phone use: Right	FALSE				303009	175355	127654	UK Biobank	https://docs.google.com/spreadsheets/d/1kvPoupSzsSFBNSztMzl04xMoSC3Kcx3CrjVf4yBmESU/edit?ts=5b5f17db#gid=227859291	PHESANT Transformation:1150_0 || CAT-SINGLE || CAT-SINGLE-BINARY-VAR: 2 || Inc(>=10): 2(175355) ||-Notes:ACE touchscreen question On what side of the head do you usually use a mobile phone? If the participant activated the Help button they were shown the message: If you are unsure, please provide an estimate or select Do not know. ~F1150~ was collected from participants who indicated they have used a mobile phone at least once per week in the past or do not know whether they have used or not, as defined by their answers to ~F1110~-Variable type:binary

binary.1150_3.txt	1710174270056F5 forCTG.txt.gz	0.2157	0.05358	4.026	5.67E-05	0.0146	0.002098	0.996	0.009695	0.01041	0.008372	Usual side of head for mobile phone use: Equally left and right	FALSE				303009	19852	283157	UK Biobank	https://docs.google.com/spreadsheets/d/1kvPoupSzsSFBNSztMzl04xMoSC3Kcx3CrjVf4yBmESU/edit?ts=5b5f17db#gid=227859291	PHESANT Transformation:1150_0 | | CAT-SINGLE | | CAT-SINGLE-BINARY-VAR: 3 | | Inc(>=10): 3(19852) | |-Notes:ACE touchscreen question On what side of the head do you usually use a mobile phone? If the participant activated the Help button they were shown the message: If you are unsure, please provide an estimate or select Do not know. ~F1150~ was collected from participants who indicated they have used a mobile phone at least once per week in the past or do not know whether they have used or not, as defined by their answers to ~F1110~-Variable type:binary
binary.1210.txt	1710174270056F5 forCTG.txt.gz	-0.1512	0.03294	-4.59	4.44E-06	0.05945	0.003161	1.024	0.01254	-0.01236	0.009116	Snoring	FALSE	Other (physical health)			336320	125656	210664	UK Biobank	https://docs.google.com/spreadsheets/d/1kvPoupSzsSFBNSztMzl04xMoSC3Kcx3CrjVf4yBmESU/edit?ts=5b5f17db#gid=227859291	PHESANT Transformation:1210_0| | CAT-SINGLE | | Inc(>=10): 1(125656) | | Inc(>=10): 2(210664) | | CAT-SINGLE-BINARY | | sample 125656/210664(336320) | |-Notes:ACE touchscreen question Does your partner or a close relative or friend complain about your snoring? If the participant activated the Help button they were shown the message: If you are unsure, please provide an estimate or select Do not know.-Variable type:binary
binary.1418_1.txt	1710174270056F5 forCTG.txt.gz	0.03208	0.05613	0.5715	0.5677	0.01227	0.002125	1.024	0.01194	0.01467	0.008324	Milk type used: Full cream	FALSE				360806	22902	337904	UK Biobank	https://docs.google.com/spreadsheets/d/1kvPoupSzsSFBNSztMzl04xMoSC3Kcx3CrjVf4yBmESU/edit?ts=5b5f17db#gid=227859291	PHESANT Transformation:1418_0 | | CAT-SINGLE | | CAT-SINGLE-BINARY-VAR: 1 | | Inc(>=10): 1(22902) | |-Notes:ACE touchscreen question What type of milk do you mainly use? If the participant activated the Help button they were shown the message: If you use more than one type of milk, please select the one that you drink the most. If you are unsure, select Do not know.-Variable type:binary
binary.1418_2.txt	1710174270056F5 forCTG.txt.gz	-0.2779	0.05765	-4.82	1.44E-06	0.01243	0.001752	1.008	0.01004	-0.008053	0.008121	Milk type used: Semi-skimmed	FALSE	Dietary			360806	234870	125936	UK Biobank	https://docs.google.com/spreadsheets/d/1kvPoupSzsSFBNSztMzl04xMoSC3Kcx3CrjVf4yBmESU/edit?ts=5b5f17db#gid=227859291	PHESANT Transformation:1418_0 | | CAT-SINGLE | | CAT-SINGLE-BINARY-VAR: 2 | | Inc(>=10): 2(234870) | |-Notes:ACE touchscreen question What type of milk do you mainly use? If the participant activated the Help button they were shown the message: If you use more than one type of milk, please select the one that you drink the most. If you are unsure, select Do not know.-Variable type:binary
binary.1418_3.txt	1710174270056F5 forCTG.txt.gz	0.1293	0.05153	2.509	0.01212	0.01835	0.00216	1.01	0.0118	-0.01488	0.008039	Milk type used: Skimmed	FALSE				360806	74087	286719	UK Biobank	https://docs.google.com/spreadsheets/d/1kvPoupSzsSFBNSztMzl04xMoSC3Kcx3CrjVf4yBmESU/edit?ts=5b5f17db#gid=227859291	PHESANT Transformation:1418_0 | | CAT-SINGLE | | CAT-SINGLE-BINARY-VAR: 3 | | Inc(>=10): 3(74087) | |-Notes:ACE touchscreen question What type of milk do you mainly use? If the participant activated the Help button they were shown the message: If you use more than one type of milk, please select the one that you drink the most. If you are unsure, select Do not know.-Variable type:binary
binary.1418_4.txt	1710174270056F5 forCTG.txt.gz	0.1195	0.06596	1.811	0.07007	0.008628	0.001723	1.025	0.009949	0.01251	0.008788	Milk type used: Soya	FALSE				360806	13147	347659	UK Biobank	https://docs.google.com/spreadsheets/d/1kvPoupSzsSFBNSztMzl04xMoSC3Kcx3CrjVf4yBmESU/edit?ts=5b5f17db#gid=227859291	PHESANT Transformation:1418_0 | | CAT-SINGLE | | CAT-SINGLE-BINARY-VAR: 4 | | Inc(>=10): 4(13147) | |-Notes:ACE touchscreen question What type of milk do you mainly use? If the participant activated the Help button they were shown the message: If you use more than one type of milk, please select the one that you drink the most. If you are unsure, select Do not know.-Variable type:binary
binary.1418_5.txt	1710174270056F5 forCTG.txt.gz	0.2592	0.1266	2.046	0.04071	0.002956	0.001612	1.003	0.01036	0.008721	0.00786	Milk type used: Other type of milk	FALSE				360806	4213	356593	UK Biobank	https://docs.google.com/spreadsheets/d/1kvPoupSzsSFBNSztMzl04xMoSC3Kcx3CrjVf4yBmESU/edit?ts=5b5f17db#gid=227859291	PHESANT Transformation:1418_0 | | CAT-SINGLE | | CAT-SINGLE-BINARY-VAR: 5 | | Inc(>=10): 5(4213) | |-Notes:ACE touchscreen question What type of milk do you mainly use? If the participant activated the Help button they were shown the message: If you use more than one type of milk, please select the one that you drink the most. If you are unsure, select Do not know.-Variable type:binary
binary.1418_6.txt	1710174270056F5 forCTG.txt.gz	0.1594	0.0483	3.3	0.000966	0.01299	0.001796	1.016	0.009853	0.01705	0.007216	Milk type used: Never/rarely have milk	FALSE				360806	11587	349219	UK Biobank	https://docs.google.com/spreadsheets/d/1kvPoupSzsSFBNSztMzl04xMoSC3Kcx3CrjVf4yBmESU/edit?ts=5b5f17db#gid=227859291	PHESANT Transformation:1418_0 | | CAT-SINGLE | | CAT-SINGLE-BINARY-VAR: 6 | | Inc(>=10): 6(11587) | |-Notes:ACE touchscreen question What type of milk do you mainly use? If the participant activated the Help button they were shown the message: If you use more than one type of milk, please select the one that you drink the most. If you are unsure, select Do not know.-Variable type:binary
binary.1428_0.txt	1710174270056F5 forCTG.txt.gz	0.08054	0.04563	1.765	0.07754	0.02069	0.002059	1.007	0.01145	0.00875	0.00836	Spread type: Never/rarely use spread	FALSE				360291	36579	323712	UK Biobank	https://docs.google.com/spreadsheets/d/1kvPoupSzsSFBNSztMzl04xMoSC3Kcx3CrjVf4yBmESU/edit?ts=5b5f17db#gid=227859291	PHESANT Transformation:1428_0 | | CAT-SINGLE | | CAT-SINGLE-BINARY-VAR: 0 | | Inc(>=10): 0(36579) | |-Notes:ACE touchscreen question What type of spread do you mainly use? If the participant activated the Help button they were shown the message: If you use more than one type of spread, please select the one that you use the most. If you are unsure, select Do not know.-Variable type:binary
binary.1428_1.txt	1710174270056F5 forCTG.txt.gz	0.0791	0.03859	2.05	0.04041	0.04129	0.002901	0.997	0.01577	5.30E-05	0.009466	Spread type: Butter/spreadable butter	FALSE				360291	131190	229101	UK Biobank	https://docs.google.com/spreadsheets/d/1kvPoupSzsSFBNSztMzl04xMoSC3Kcx3CrjVf4yBmESU/edit?ts=5b5f17db#gid=227859291	PHESANT Transformation:1428_0 | | CAT-SINGLE | | CAT-SINGLE-BINARY-VAR: 1 | | Inc(>=10): 1(131190) | |-Notes:ACE touchscreen question What type of spread do you mainly use? If the participant activated the Help button they were shown the message: If you use more than one type of spread, please select the one that you use the most. If you are unsure, select Do not know.-Variable type:binary
binary.1428_3.txt	1710174270056F5 forCTG.txt.gz	-0.1325	0.03959	-3.348	0.000815	0.02958	0.002377	1.001	0.0121	-0.004533	0.008744	Spread type: Other type of spread/margarine	FALSE				360291	190593	169698	UK Biobank	https://docs.google.com/spreadsheets/d/1kvPoupSzsSFBNSztMzl04xMoSC3Kcx3CrjVf4yBmESU/edit?ts=5b5f17db#gid=227859291	PHESANT Transformation:1428_0 | | CAT-SINGLE | | CAT-SINGLE-BINARY-VAR: 3 | | Inc(>=10): 3(190593) | |-Notes:ACE touchscreen question What type of spread do you mainly use? If the participant activated the Help button they were shown the message: If you use more than one type of spread, please select the one that you use the most. If you are unsure, select Do not know.-Variable type:binary
binary.1448_1.txt	1710174270056F5 forCTG.txt.gz	0.1885	0.03869	4.871	1.11E-06	0.05439	0.003072	1.042	0.01412	-0.002646	0.01107	Bread type: White	FALSE	Dietary			348424	91452	256972	UK Biobank	https://docs.google.com/spreadsheets/d/1kvPoupSzsSFBNSztMzl04xMoSC3Kcx3CrjVf4yBmESU/edit?ts=5b5f17db#gid=227859291	PHESANT Transformation:1448_0 | | CAT-SINGLE | | CAT-SINGLE-BINARY-VAR: 1 | | Inc(>=10): 1(91452) | |-Notes:ACE touchscreen question What type of bread do you mainly eat? If the participant activated the Help button they were shown the message: If you eat more than one type of bread, please select the one that you eat the most. If you are unsure, select Do not know. ~F1448~ was collected from all participants except those who indicated that they do not eat bread or less than one slice each week, as defined by their answers to ~F1438~-Variable type:binary
binary.1448_2.txt	1710174270056F5 forCTG.txt.gz	0.1802	0.0564	3.196	0.001394	0.01164	0.001771	0.995	0.009945	-0.009085	0.008053	Bread type: Brown	FALSE				348424	41518	306906	UK Biobank	https://docs.google.com/spreadsheets/d/1kvPoupSzsSFBNSztMzl04xMoSC3Kcx3CrjVf4yBmESU/edit?ts=5b5f17db#gid=227859291	PHESANT Transformation:1448_0 | | CAT-SINGLE | | CAT-SINGLE-BINARY-VAR: 2 | | Inc(>=10): 2(41518) | |-Notes:ACE touchscreen question What type of bread do you mainly eat? If the participant activated the Help button they were shown the message: If you eat more than one type of bread, please select the one that you eat the most. If you are unsure, select Do not know. ~F1448~ was collected from all participants except those who indicated that they do not eat bread or less than one slice each week, as defined by their answers to ~F1438~-Variable type:binary
binary.1448_3.txt	1710174270056F5 forCTG.txt.gz	-0.2798	0.03973	-7.042	1.90E-12	0.04483	0.002869	1.041	0.01402	0.00623	0.01056	Bread type: Wholemeal or wholegrain	FALSE	Dietary			348424	201013	147411	UK Biobank	https://docs.google.com/spreadsheets/d/1kvPoupSzsSFBNSztMzl04xMoSC3Kcx3CrjVf4yBmESU/edit?ts=5b5f17db#gid=227859291	PHESANT Transformation:1448_0 | | CAT-SINGLE | | CAT-SINGLE-BINARY-VAR: 3 | | Inc(>=10): 3(201013) | |-Notes:ACE touchscreen question What type of bread do you mainly eat? If the participant activated the Help button they were shown the message: If you eat more than one type of bread, please select the one that you eat the most. If you are unsure, select Do not know. ~F1448~ was collected from all participants except those who indicated that they do not eat bread or less than one slice each week, as defined by their answers to ~F1438~-Variable type:binary
binary.1448_4.txt	1710174270056F5 forCTG.txt.gz	0.267	0.1032	2.588	0.009659	0.004492	0.001615	0.997	0.009895	0.0053	0.008124	Bread type: Other type of bread	FALSE				348424	14441	333983	UK Biobank	https://docs.google.com/spreadsheets/d/1kvPoupSzsSFBNSztMzl04xMoSC3Kcx3CrjVf4yBmESU/edit?ts=5b5f17db#gid=227859291	PHESANT Transformation:1448_0 | | CAT-SINGLE | | CAT-SINGLE-BINARY-VAR: 4 | | Inc(>=10): 4(14441) | |-Notes:ACE touchscreen question What type of bread do you mainly eat? If the participant activated the Help button they were shown the message: If you eat more than one type of bread, please select the one that you eat the most. If you are unsure, select Do not know. ~F1448~ was collected from all participants except those who indicated that they do not eat bread or less than one slice each week, as defined by their answers to ~F1438~-Variable type:binary
binary.1468_1.txt	1710174270056F5 forCTG.txt.gz	-0.06209	0.077	-0.8064	0.42	0.006891	0.001862	1.025	0.009566	0.005353	0.00823	Cereal type: Bran cereal (e.g. All Bran, Branflakes)	FALSE				299898	50609	249289	UK Biobank	https://docs.google.com/spreadsheets/d/1kvPoupSzsSFBNSztMzl04xMoSC3Kcx3CrjVf4yBmESU/edit?ts=5b5f17db#gid=227859291	PHESANT Transformation:1468_0 | | CAT-SINGLE | | CAT-SINGLE-BINARY-VAR: 1 | | Inc(>=10): 1(50609) | |-Notes:ACE touchscreen question What type of cereal do you mainly eat? If the participant activated the Help button they were shown the message: If you eat more than one type of cereal, please select the one that you eat the most. If you are unsure, select Do not know. ~F1468~ was collected from all participants except those who indicated that they do not eat cereal or less than one bowl of cereal each week, as defined by their answers to ~F1458~-Variable type:binary
binary.1468_2.txt	1710174270056F5 forCTG.txt.gz	0.1846	0.07727	2.389	0.01687	0.008308	0.002054	1.031	0.009893	-0.00282	0.008687	Cereal type: Biscuit cereal (e.g. Weetabix)	FALSE				299898	53310	246588	UK Biobank	https://docs.google.com/spreadsheets/d/1kvPoupSzsSFBNSztMzl04xMoSC3Kcx3CrjVf4yBmESU/edit?ts=5b5f17db#gid=227859291	PHESANT Transformation:1468_0 | | CAT-SINGLE | | CAT-SINGLE-BINARY-VAR: 2 | | Inc(>=10): 2(53310) | |-Notes:ACE touchscreen question What type of cereal do you mainly eat? If the participant activated the Help button they were shown the message: If you eat more than one type of cereal, please select the one that you eat the most. If you are unsure, select Do not know. ~F1468~ was collected from all participants except those who indicated that they do not eat cereal or less than one bowl of cereal each week, as defined by their answers to ~F1458~-Variable type:binary
binary.1468_3.txt	1710174270056F5 forCTG.txt.gz	0.1592	0.05398	2.949	0.003188	0.01777	0.002383	1.015	0.01143	0.01409	0.008822	Cereal type: Oat cereal (e.g. Ready Brek, porridge)	FALSE				299898	76351	223547	UK Biobank	https://docs.google.com/spreadsheets/d/1kvPoupSzsSFBNSztMzl04xMoSC3Kcx3CrjVf4yBmESU/edit?ts=5b5f17db#gid=227859291	PHESANT Transformation:1468_0 | | CAT-SINGLE | | CAT-SINGLE-BINARY-VAR: 3 | | Inc(>=10): 3(76351) | |-Notes:ACE touchscreen question What type of cereal do you mainly eat? If the participant activated the Help button they were shown the message: If you eat more than one type of cereal, please select the one that you eat the most. If you are unsure, select Do not know. ~F1468~ was collected from all participants except those who indicated that they do not eat cereal or less than one bowl of cereal each week, as defined by their answers to ~F1458~-Variable type:binary

<table>
<tr><td>binary.1468_4.txt</td><td>1710174270056F5forCTG.txt.gz</td><td>-0.2312</td><td>0.03421</td><td>-6.758</td><td>1.40E-11</td><td>0.04753</td><td>0.002964</td><td>1.038</td><td>0.01212</td><td>-0.003482</td><td>0.008759</td><td>Cereal type: Muesli</td><td>FALSE</td><td>Dietary</td><td></td><td></td><td></td><td>299898</td><td>61523</td><td>238375</td><td>UK Biobank</td><td>https://docs.google.com/spreadsheets/d/1kvPoupSzsSFBNSztMzl04xMoSC3Kcx3CrjVf4yBmESU/edit?ts=5b5f17db#gid=227859291</td><td>PHESANT Transformation:1468_0 || CAT-SINGLE || CAT-SINGLE-BINARY-VAR: 4 || Inc(>=10): 4(61523) ||-Notes:ACE touchscreen question What type of cereal do you mainly eat? If the participant activated the Help button they were shown the message: If you eat more than one type of cereal, please select the one that you eat the most. If you are unsure, select Do not know. ~F1468~ was collected from all participants except those who indicated that they do not eat cereal or less than one bowl of cereal each week, as defined by their answers to ~F1458~-Variable type:binary</td></tr>
<tr><td>binary.1468_5.txt</td><td>1710174270056F5forCTG.txt.gz</td><td>0.08329</td><td>0.04156</td><td>2.004</td><td>0.04506</td><td>0.03601</td><td>0.00266</td><td>1.01</td><td>0.01223</td><td>-0.01307</td><td>0.008058</td><td>Cereal type: Other (e.g. Cornflakes, Frosties)</td><td>FALSE</td><td></td><td></td><td></td><td></td><td>299898</td><td>58105</td><td>241793</td><td>UK Biobank</td><td>https://docs.google.com/spreadsheets/d/1kvPoupSzsSFBNSztMzl04xMoSC3Kcx3CrjVf4yBmESU/edit?ts=5b5f17db#gid=227859291</td><td>PHESANT Transformation:1468_0 || CAT-SINGLE || CAT-SINGLE-BINARY-VAR: 5 || Inc(>=10): 5(58105) ||-Notes:ACE touchscreen question What type of cereal do you mainly eat? If the participant activated the Help button they were shown the message: If you eat more than one type of cereal, please select the one that you eat the most. If you are unsure, select Do not know. ~F1468~ was collected from all participants except those who indicated that they do not eat cereal or less than one bowl of cereal each week, as defined by their answers to ~F1458~-Variable type:binary</td></tr>
<tr><td>binary.1508_1.txt</td><td>1710174270056F5forCTG.txt.gz</td><td>0.03231</td><td>0.06033</td><td>0.5356</td><td>0.5923</td><td>0.01568</td><td>0.002476</td><td>1.009</td><td>0.01139</td><td>0.0009573</td><td>0.008379</td><td>Coffee type: Decaffeinated coffee (any type)</td><td>FALSE</td><td></td><td></td><td></td><td></td><td>283449</td><td>55310</td><td>228139</td><td>UK Biobank</td><td>https://docs.google.com/spreadsheets/d/1kvPoupSzsSFBNSztMzl04xMoSC3Kcx3CrjVf4yBmESU/edit?ts=5b5f17db#gid=227859291</td><td>PHESANT Transformation:1508_0 || CAT-SINGLE || CAT-SINGLE-BINARY-VAR: 1 || Inc(>=10): 1(55310) ||-Notes:ACE touchscreen question What type of coffee do you usually drink? If the participant activated the Help button they were shown the message: If you drink more than one type of coffee, please select the one that you drink the most. If you are unsure, select Do not know. ~F1508~ was collected from participants who indicated that they drank at least 1 cup of coffee each day or less than one cup each day, as defined by their answers to ~F1498~-Variable type:binary</td></tr>
<tr><td>binary.1508_2.txt</td><td>1710174270056F5forCTG.txt.gz</td><td>0.0772</td><td>0.04162</td><td>1.855</td><td>0.06361</td><td>0.03326</td><td>0.00312</td><td>1.022</td><td>0.01186</td><td>0.008371</td><td>0.008671</td><td>Coffee type: Instant coffee</td><td>FALSE</td><td></td><td></td><td></td><td></td><td>283449</td><td>158386</td><td>125063</td><td>UK Biobank</td><td>https://docs.google.com/spreadsheets/d/1kvPoupSzsSFBNSztMzl04xMoSC3Kcx3CrjVf4yBmESU/edit?ts=5b5f17db#gid=227859291</td><td>PHESANT Transformation:1508_0 || CAT-SINGLE || CAT-SINGLE-BINARY-VAR: 2 || Inc(>=10): 2(158386) ||-Notes:ACE touchscreen question What type of coffee do you usually drink? If the participant activated the Help button they were shown the message: If you drink more than one type of coffee, please select the one that you drink the most. If you are unsure, select Do not know. ~F1508~ was collected from participants who indicated that they drank at least 1 cup of coffee each day or less than one cup each day, as defined by their answers to ~F1498~-Variable type:binary</td></tr>
<tr><td>binary.1508_3.txt</td><td>1710174270056F5forCTG.txt.gz</td><td>-0.1036</td><td>0.03393</td><td>-3.053</td><td>0.002265</td><td>0.06444</td><td>0.003825</td><td>1.033</td><td>0.01365</td><td>-0.01066</td><td>0.008782</td><td>Coffee type: Ground coffee (include espresso, filter etc)</td><td>FALSE</td><td></td><td></td><td></td><td></td><td>283449</td><td>64962</td><td>218487</td><td>UK Biobank</td><td>https://docs.google.com/spreadsheets/d/1kvPoupSzsSFBNSztMzl04xMoSC3Kcx3CrjVf4yBmESU/edit?ts=5b5f17db#gid=227859291</td><td>PHESANT Transformation:1508_0 || CAT-SINGLE || CAT-SINGLE-BINARY-VAR: 3 || Inc(>=10): 3(64962) ||-Notes:ACE touchscreen question What type of coffee do you usually drink? If the participant activated the Help button they were shown the message: If you drink more than one type of coffee, please select the one that you drink the most. If you are unsure, select Do not know. ~F1508~ was collected from participants who indicated that they drank at least 1 cup of coffee each day or less than one cup each day, as defined by their answers to ~F1498~-Variable type:binary</td></tr>
<tr><td>binary.1508_4.txt</td><td>1710174270056F5forCTG.txt.gz</td><td>0.3453</td><td>0.1587</td><td>2.175</td><td>0.0296</td><td>0.003402</td><td>0.001938</td><td>1.007</td><td>0.008888</td><td>-0.001741</td><td>0.009387</td><td>Coffee type: Other type of coffee</td><td>FALSE</td><td></td><td></td><td></td><td></td><td>283449</td><td>4791</td><td>278658</td><td>UK Biobank</td><td>https://docs.google.com/spreadsheets/d/1kvPoupSzsSFBNSztMzl04xMoSC3Kcx3CrjVf4yBmESU/edit?ts=5b5f17db#gid=227859291</td><td>PHESANT Transformation:1508_0 || CAT-SINGLE || CAT-SINGLE-BINARY-VAR: 4 || Inc(>=10): 4(4791) ||-Notes:ACE touchscreen question What type of coffee do you usually drink? If the participant activated the Help button they were shown the message: If you drink more than one type of coffee, please select the one that you drink the most. If you are unsure, select Do not know. ~F1508~ was collected from participants who indicated that they drank at least 1 cup of coffee each day or less than one cup each day, as defined by their answers to ~F1498~-Variable type:binary</td></tr>
<tr><td>binary.1538_0.txt</td><td>1710174270056F5forCTG.txt.gz</td><td>-0.3048</td><td>0.03642</td><td>-8.368</td><td>5.85E-17</td><td>0.0358</td><td>0.003009</td><td>1.034</td><td>0.01582</td><td>-0.01106</td><td>0.008632</td><td>Major dietary changes in the last 5 years: No</td><td>FALSE</td><td>Lifestyle</td><td></td><td></td><td></td><td>360294</td><td>221368</td><td>138926</td><td>UK Biobank</td><td>https://docs.google.com/spreadsheets/d/1kvPoupSzsSFBNSztMzl04xMoSC3Kcx3CrjVf4yBmESU/edit?ts=5b5f17db#gid=227859291</td><td>PHESANT Transformation:1538_0 || CAT-SINGLE || CAT-SINGLE-BINARY-VAR: 0 || Inc(>=10): 0(221368) ||-Notes:ACE touchscreen question Have you made any major changes to your diet in the last 5 years?-Variable type:binary</td></tr>
<tr><td>binary.1538_1.txt</td><td>1710174270056F5forCTG.txt.gz</td><td>0.3713</td><td>0.04516</td><td>8.222</td><td>2.00E-16</td><td>0.02505</td><td>0.00225</td><td>1.031</td><td>0.0118</td><td>0.01745</td><td>0.008927</td><td>Major dietary changes in the last 5 years: Yes, because of illness</td><td>FALSE</td><td>Lifestyle</td><td></td><td></td><td></td><td>360294</td><td>38051</td><td>322243</td><td>UK Biobank</td><td>https://docs.google.com/spreadsheets/d/1kvPoupSzsSFBNSztMzl04xMoSC3Kcx3CrjVf4yBmESU/edit?ts=5b5f17db#gid=227859291</td><td>PHESANT Transformation:1538_0 || CAT-SINGLE || CAT-SINGLE-BINARY-VAR: 1 || Inc(>=10): 1(38051) ||-Notes:ACE touchscreen question Have you made any major changes to your diet in the last 5 years?-Variable type:binary</td></tr>
<tr><td>binary.1538_2.txt</td><td>1710174270056F5forCTG.txt.gz</td><td>0.1722</td><td>0.04899</td><td>3.515</td><td>0.00044</td><td>0.0167</td><td>0.001979</td><td>1.021</td><td>0.01097</td><td>0.000217</td><td>0.008432</td><td>Major dietary changes in the last 5 years: Yes, because of other reasons</td><td>FALSE</td><td></td><td></td><td></td><td></td><td>360294</td><td>100875</td><td>259419</td><td>UK Biobank</td><td>https://docs.google.com/spreadsheets/d/1kvPoupSzsSFBNSztMzl04xMoSC3Kcx3CrjVf4yBmESU/edit?ts=5b5f17db#gid=227859291</td><td>PHESANT Transformation:1538_0 || CAT-SINGLE || CAT-SINGLE-BINARY-VAR: 2 || Inc(>=10): 2(100875) ||-Notes:ACE touchscreen question Have you made any major changes to your diet in the last 5 years?-Variable type:binary</td></tr>
<tr><td>binary.1618.txt</td><td>1710174270056F5forCTG.txt.gz</td><td>-0.2164</td><td>0.03515</td><td>-6.157</td><td>7.43E-10</td><td>0.09168</td><td>0.005351</td><td>1.055</td><td>0.01354</td><td>-0.01005</td><td>0.009426</td><td>Alcohol usually taken with meals</td><td>FALSE</td><td>Lifestyle</td><td></td><td></td><td></td><td>184716</td><td>125164</td><td>59552</td><td>UK Biobank</td><td>https://docs.google.com/spreadsheets/d/1kvPoupSzsSFBNSztMzl04xMoSC3Kcx3CrjVf4yBmESU/edit?ts=5b5f17db#gid=227859291</td><td>PHESANT Transformation:1618_0|| CAT-SINGLE || Inc(>=10): 0(59552) || Inc(>=10): 1(125164) || CAT-SINGLE-BINARY || sample 59552/125164(184716) ||-Notes:ACE touchscreen question When you drink alcohol is it usually with meals? ~F1618~ was collected from participants who indicated they drink alcohol, as defined by their answers to ~F1558~-Variable type:binary</td></tr>
<tr><td>binary.1677.txt</td><td>1710174270056F5forCTG.txt.gz</td><td>-0.1529</td><td>0.05122</td><td>-2.985</td><td>0.00284</td><td>0.02132</td><td>0.002681</td><td>1.021</td><td>0.01093</td><td>-0.001638</td><td>0.008585</td><td>Breastfed as a baby</td><td>FALSE</td><td></td><td></td><td></td><td></td><td>273743</td><td>193838</td><td>79905</td><td>UK Biobank</td><td>https://docs.google.com/spreadsheets/d/1kvPoupSzsSFBNSztMzl04xMoSC3Kcx3CrjVf4yBmESU/edit?ts=5b5f17db#gid=227859291</td><td>PHESANT Transformation:1677_0|| CAT-SINGLE || Inc(>=10): 1(193838) || Inc(>=10): 0(79905) || CAT-SINGLE-BINARY || sample 79905/193838(273743) ||-Notes:ACE touchscreen question Were you breastfed when you were a baby?-Variable type:binary</td></tr>
<tr><td>binary.1707_1.txt</td><td>1710174270056F5forCTG.txt.gz</td><td>0.01505</td><td>0.06186</td><td>0.2434</td><td>0.8077</td><td>0.009563</td><td>0.001844</td><td>1.005</td><td>0.01025</td><td>-0.005237</td><td>0.007973</td><td>Handedness (chirality/laterality): Right-handed</td><td>FALSE</td><td></td><td></td><td></td><td></td><td>360913</td><td>320242</td><td>40671</td><td>UK Biobank</td><td>https://docs.google.com/spreadsheets/d/1kvPoupSzsSFBNSztMzl04xMoSC3Kcx3CrjVf4yBmESU/edit?ts=5b5f17db#gid=227859291</td><td>PHESANT Transformation:1707_0 || CAT-SINGLE || CAT-SINGLE-BINARY-VAR: 1 || Inc(>=10): 1(320242) ||-Notes:ACE touchscreen question Are you right or left handed?-Variable type:binary</td></tr>
<tr><td>binary.1707_2.txt</td><td>1710174270056F5forCTG.txt.gz</td><td>-0.09959</td><td>0.05863</td><td>-1.699</td><td>0.08937</td><td>0.01051</td><td>0.00187</td><td>1</td><td>0.01038</td><td>0.004467</td><td>0.007445</td><td>Handedness (chirality/laterality): Left-handed</td><td>FALSE</td><td></td><td></td><td></td><td></td><td>360913</td><td>34585</td><td>326328</td><td>UK Biobank</td><td>https://docs.google.com/spreadsheets/d/1kvPoupSzsSFBNSztMzl04xMoSC3Kcx3CrjVf4yBmESU/edit?ts=5b5f17db#gid=227859291</td><td>PHESANT Transformation:1707_0 || CAT-SINGLE || CAT-SINGLE-BINARY-VAR: 2 || Inc(>=10): 2(34585) ||-Notes:ACE touchscreen question Are you right or left handed?-Variable type:binary</td></tr>
<tr><td>binary.1707_3.txt</td><td>1710174270056F5forCTG.txt.gz</td><td>0.2877</td><td>0.08825</td><td>3.261</td><td>0.001112</td><td>0.00472</td><td>0.001623</td><td>1.004</td><td>0.009755</td><td>0.002573</td><td>0.007648</td><td>Handedness (chirality/laterality): Use both right and left hands equally</td><td>FALSE</td><td></td><td></td><td></td><td></td><td>360913</td><td>6086</td><td>354827</td><td>UK Biobank</td><td>https://docs.google.com/spreadsheets/d/1kvPoupSzsSFBNSztMzl04xMoSC3Kcx3CrjVf4yBmESU/edit?ts=5b5f17db#gid=227859291</td><td>PHESANT Transformation:1707_0 || CAT-SINGLE || CAT-SINGLE-BINARY-VAR: 3 || Inc(>=10): 3(6086) ||-Notes:ACE touchscreen question Are you right or left handed?-Variable type:binary</td></tr>
<tr><td>binary.1747_1.txt</td><td>1710174270056F5forCTG.txt.gz</td><td>0.09005</td><td>0.02764</td><td>3.258</td><td>0.00112</td><td>0.09581</td><td>0.02091</td><td>1.105</td><td>0.09972</td><td>-0.01665</td><td>0.009159</td><td>Hair colour (natural, before greying): Blonde</td><td>FALSE</td><td></td><td></td><td></td><td></td><td>360270</td><td>41178</td><td>319092</td><td>UK Biobank</td><td>https://docs.google.com/spreadsheets/d/1kvPoupSzsSFBNSztMzl04xMoSC3Kcx3CrjVf4yBmESU/edit?ts=5b5f17db#gid=227859291</td><td>PHESANT Transformation:1747_0 || CAT-SINGLE || CAT-SINGLE-BINARY-VAR: 1 || Inc(>=10): 1(41178) ||-Notes:ACE touchscreen question What best describes your natural hair colour? (If your hair colour is grey, the colour before you went grey) If the participant activated the Help button they were shown the message: If you are unsure, please select the colour closest to your natural adult hair colour or select Do not know.-Variable type:binary</td></tr>
<tr><td>binary.1747_3.txt</td><td>1710174270056F5forCTG.txt.gz</td><td>0.003065</td><td>0.04283</td><td>0.07157</td><td>0.9429</td><td>0.02717</td><td>0.007743</td><td>1.068</td><td>0.0508</td><td>-0.009616</td><td>0.008782</td><td>Hair colour (natural, before greying): Light brown</td><td>FALSE</td><td></td><td></td><td></td><td></td><td>360270</td><td>147560</td><td>212710</td><td>UK Biobank</td><td>https://docs.google.com/spreadsheets/d/1kvPoupSzsSFBNSztMzl04xMoSC3Kcx3CrjVf4yBmESU/edit?ts=5b5f17db#gid=227859291</td><td>PHESANT Transformation:1747_0 || CAT-SINGLE || CAT-SINGLE-BINARY-VAR: 3 || Inc(>=10): 3(147560) ||-Notes:ACE touchscreen question What best describes your natural hair colour? (If your hair colour is grey, the colour before you went grey) If the participant activated the Help button they were shown the message: If you are unsure, please select the colour closest to your natural adult hair colour or select Do not know.-Variable type:binary</td></tr>
<tr><td>binary.1747_4.txt</td><td>1710174270056F5forCTG.txt.gz</td><td>-0.09028</td><td>0.02587</td><td>-3.49</td><td>0.000484</td><td>0.1116</td><td>0.02823</td><td>1.001</td><td>0.1034</td><td>0.0192</td><td>0.01017</td><td>Hair colour (natural, before greying): Dark brown</td><td>FALSE</td><td></td><td></td><td></td><td></td><td>360270</td><td>134627</td><td>225643</td><td>UK Biobank</td><td>https://docs.google.com/spreadsheets/d/1kvPoupSzsSFBNSztMzl04xMoSC3Kcx3CrjVf4yBmESU/edit?ts=5b5f17db#gid=227859291</td><td>PHESANT Transformation:1747_0 || CAT-SINGLE || CAT-SINGLE-BINARY-VAR: 4 || Inc(>=10): 4(134627) ||-Notes:ACE touchscreen question What best describes your natural hair colour? (If your hair colour is grey, the colour before you went grey) If the participant activated the Help button they were shown the message: If you are unsure, please select the colour closest to your natural adult hair colour or select Do not know.-Variable type:binary</td></tr>
<tr><td>binary.1747_5.txt</td><td>1710174270056F5forCTG.txt.gz</td><td>0.02297</td><td>0.03592</td><td>0.6394</td><td>0.5225</td><td>0.03736</td><td>0.0124</td><td>1.081</td><td>0.08812</td><td>0.006647</td><td>0.009114</td><td>Hair colour (natural, before greying): Black</td><td>FALSE</td><td></td><td></td><td></td><td></td><td>360270</td><td>15809</td><td>344461</td><td>UK Biobank</td><td>https://docs.google.com/spreadsheets/d/1kvPoupSzsSFBNSztMzl04xMoSC3Kcx3CrjVf4yBmESU/edit?ts=5b5f17db#gid=227859291</td><td>PHESANT Transformation:1747_0 || CAT-SINGLE || CAT-SINGLE-BINARY-VAR: 5 || Inc(>=10): 5(15809) ||-Notes:ACE touchscreen question What best describes your natural hair colour? (If your hair colour is grey, the colour before you went grey) If the participant activated the Help button they were shown the message: If you are unsure, please select the colour closest to your natural adult hair colour or select Do not know.-Variable type:binary</td></tr>
<tr><td>binary.1747_6.txt</td><td>1710174270056F5forCTG.txt.gz</td><td>0.2939</td><td>0.1398</td><td>2.103</td><td>0.03546</td><td>0.002894</td><td>0.001714</td><td>1.014</td><td>0.008339</td><td>-0.01076</td><td>0.007292</td><td>Hair colour (natural, before greying): Other</td><td>FALSE</td><td></td><td></td><td></td><td></td><td>360270</td><td>4481</td><td>355789</td><td>UK Biobank</td><td>https://docs.google.com/spreadsheets/d/1kvPoupSzsSFBNSztMzl04xMoSC3Kcx3CrjVf4yBmESU/edit?ts=5b5f17db#gid=227859291</td><td>PHESANT Transformation:1747_0 || CAT-SINGLE || CAT-SINGLE-BINARY-VAR: 6 || Inc(>=10): 6(4481) ||-Notes:ACE touchscreen question What best describes your natural hair colour? (If your hair colour is grey, the colour before you went grey) If the participant activated the Help button they were shown the message: If you are unsure, please select the colour closest to your natural adult hair colour or select Do not know.-Variable type:binary</td></tr>
<tr><td>binary.1767.txt</td><td>1710174270056F5forCTG.txt.gz</td><td>0.3447</td><td>0.09534</td><td>3.616</td><td>0.000299</td><td>0.004652</td><td>0.001488</td><td>1.007</td><td>0.008762</td><td>0.01269</td><td>0.007135</td><td>Adopted as a child</td><td>FALSE</td><td></td><td></td><td></td><td></td><td>360450</td><td>5158</td><td>355292</td><td>UK Biobank</td><td>https://docs.google.com/spreadsheets/d/1kvPoupSzsSFBNSztMzl04xMoSC3Kcx3CrjVf4yBmESU/edit?ts=5b5f17db#gid=227859291</td><td>PHESANT Transformation:1767_0|| CAT-SINGLE || Inc(>=10): 0(355292) || Inc(>=10): 1(5158) || CAT-SINGLE-BINARY || sample 355292/5158(360450) ||-Notes:ACE touchscreen question Were you adopted as a child?-Variable type:binary</td></tr>
</table>

<table>
<tr><td>binary.1787.txt</td><td>1710174270056F5 forCTG.txt.gz</td><td>0.3199</td><td>0.03676</td><td>8.7</td><td>3.31E-18</td><td>0.0445</td><td>0.002987</td><td>1.023</td><td>0.01257</td><td>0.01513</td><td>0.009952</td><td>Maternal smoking around birth</td><td>FALSE</td><td>Other</td><td></td><td></td><td>309942</td><td>95182</td><td>214760</td><td>UK Biobank</td><td>https://docs.google.com/spreadsheets/d/1kvPoupSzsSFBNSztMzl04xMoSC3Kcx3CrjVf4yBmESU/edit?ts=5b5f17db#gid=227859291</td><td>PHESANT Transformation:1787_0|| CAT-SINGLE || Inc(>=10): 0(214760) || Inc(>=10): 1(95182) || CAT-SINGLE-BINARY || sample 214760/95182(309942) ||-Notes:ACE touchscreen question Did your mother smoke regularly around the time when you were born? ~F1787~ was collected from all participants except those who indicated they were adopted as a child, as defined by their answers to ~F1767~-Variable type:binary</td></tr>
<tr><td>binary.1797.txt</td><td>1710174270056F5 forCTG.txt.gz</td><td>-0.09728</td><td>0.05613</td><td>-1.733</td><td>0.08307</td><td>0.008932</td><td>0.001831</td><td>1.005</td><td>0.009887</td><td>-0.000769</td><td>0.007567</td><td>Father still alive</td><td>FALSE</td><td></td><td></td><td></td><td>351538</td><td>81229</td><td>270309</td><td>UK Biobank</td><td>https://docs.google.com/spreadsheets/d/1kvPoupSzsSFBNSztMzl04xMoSC3Kcx3CrjVf4yBmESU/edit?ts=5b5f17db#gid=227859291</td><td>PHESANT Transformation:1797_0|| CAT-SINGLE || Inc(>=10): 0(270309) || Inc(>=10): 1(81229) || CAT-SINGLE-BINARY || sample 270309/81229(351538) ||-Notes:ACE touchscreen question Is your father still alive? ~F1797~ was collected from all participants except those who indicated they were adopted as a child, as defined by their answers to ~F1767~-Variable type:binary</td></tr>
<tr><td>binary.1835.txt</td><td>1710174270056F5 forCTG.txt.gz</td><td>0.01777</td><td>0.0801</td><td>0.2219</td><td>0.8244</td><td>0.005752</td><td>0.001796</td><td>1.014</td><td>0.01108</td><td>-0.01115</td><td>0.008831</td><td>Mother still alive</td><td>FALSE</td><td></td><td></td><td></td><td>355029</td><td>140246</td><td>214783</td><td>UK Biobank</td><td>https://docs.google.com/spreadsheets/d/1kvPoupSzsSFBNSztMzl04xMoSC3Kcx3CrjVf4yBmESU/edit?ts=5b5f17db#gid=227859291</td><td>PHESANT Transformation:1835_0|| CAT-SINGLE || Inc(>=10): 0(214783) || Inc(>=10): 1(140246) || CAT-SINGLE-BINARY || sample 214783/140246(355029) ||-Notes:ACE touchscreen question Is your mother still alive? ~F1835~ was collected from all participants except those who indicated they were adopted as a child, as defined by their answers to ~F1767~-Variable type:binary</td></tr>
<tr><td>binary.1920.txt</td><td>1710174270056F5 forCTG.txt.gz</td><td>0.2737</td><td>0.03037</td><td>9.013</td><td>2.01E-19</td><td>0.07079</td><td>0.003683</td><td>1.007</td><td>0.0151</td><td>-0.007269</td><td>0.009396</td><td>Mood swings</td><td>TRUE</td><td>Psychiatric</td><td></td><td>Mood swings</td><td>352604</td><td>158982</td><td>193622</td><td>UK Biobank</td><td>https://docs.google.com/spreadsheets/d/1kvPoupSzsSFBNSztMzl04xMoSC3Kcx3CrjVf4yBmESU/edit?ts=5b5f17db#gid=227859291</td><td>PHESANT Transformation:1920_0|| CAT-SINGLE || Inc(>=10): 0(193622) || Inc(>=10): 1(158982) || CAT-SINGLE-BINARY || sample 193622/158982(352604) ||-Notes:ACE touchscreen question Does your mood often go up and down? If the participant activated the Help button they were shown the message: Work through these questions quickly and do not think about the exact meaning of the question-Variable type:binary</td></tr>
<tr><td>binary.1930.txt</td><td>1710174270056F5 forCTG.txt.gz</td><td>0.1924</td><td>0.03437</td><td>5.598</td><td>2.17E-08</td><td>0.06404</td><td>0.003855</td><td>0.999</td><td>0.01671</td><td>-0.003399</td><td>0.009055</td><td>Miserableness</td><td>FALSE</td><td>Psychiatric</td><td></td><td></td><td>355182</td><td>151752</td><td>203430</td><td>UK Biobank</td><td>https://docs.google.com/spreadsheets/d/1kvPoupSzsSFBNSztMzl04xMoSC3Kcx3CrjVf4yBmESU/edit?ts=5b5f17db#gid=227859291</td><td>PHESANT Transformation:1930_0|| CAT-SINGLE || Inc(>=10): 0(203430) || Inc(>=10): 1(151752) || CAT-SINGLE-BINARY || sample 203430/151752(355182) ||-Notes:ACE touchscreen question Do you ever feel 'just miserable' for no reason? If the participant activated the Help button they were shown the message: Work through these questions quickly and do not think about the exact meaning of the question-Variable type:binary</td></tr>
<tr><td>binary.1940.txt</td><td>1710174270056F5 forCTG.txt.gz</td><td>0.04957</td><td>0.03481</td><td>1.424</td><td>0.1544</td><td>0.0677</td><td>0.004274</td><td>0.986</td><td>0.01745</td><td>0.00158</td><td>0.01014</td><td>Irritability</td><td>FALSE</td><td></td><td></td><td></td><td>345231</td><td>96862</td><td>248369</td><td>UK Biobank</td><td>https://docs.google.com/spreadsheets/d/1kvPoupSzsSFBNSztMzl04xMoSC3Kcx3CrjVf4yBmESU/edit?ts=5b5f17db#gid=227859291</td><td>PHESANT Transformation:1940_0|| CAT-SINGLE || Inc(>=10): 0(248369) || Inc(>=10): 1(96862) || CAT-SINGLE-BINARY || sample 248369/96862(345231) ||-Notes:ACE touchscreen question Are you an irritable person? If the participant activated the Help button they were shown the message: Work through these questions quickly and do not think about the exact meaning of the question-Variable type:binary</td></tr>
<tr><td>binary.1950.txt</td><td>1710174270056F5 forCTG.txt.gz</td><td>0.05569</td><td>0.03425</td><td>1.626</td><td>0.104</td><td>0.06448</td><td>0.003776</td><td>0.998</td><td>0.01609</td><td>-0.01262</td><td>0.009632</td><td>Sensitivity / hurt feelings</td><td>FALSE</td><td></td><td></td><td></td><td>350821</td><td>194755</td><td>156066</td><td>UK Biobank</td><td>https://docs.google.com/spreadsheets/d/1kvPoupSzsSFBNSztMzl04xMoSC3Kcx3CrjVf4yBmESU/edit?ts=5b5f17db#gid=227859291</td><td>PHESANT Transformation:1950_0|| CAT-SINGLE || Inc(>=10): 0(156066) || Inc(>=10): 1(194755) || CAT-SINGLE-BINARY || sample 156066/194755(350821) ||-Notes:ACE touchscreen question Are your feelings easily hurt? If the participant activated the Help button they were shown the message: Work through these questions quickly and do not think about the exact meaning of the question-Variable type:binary</td></tr>
<tr><td>binary.1960.txt</td><td>1710174270056F5 forCTG.txt.gz</td><td>0.2043</td><td>0.03389</td><td>6.029</td><td>1.65E-09</td><td>0.07005</td><td>0.003628</td><td>1.014</td><td>0.0153</td><td>0.003828</td><td>0.00942</td><td>Fed-up feelings</td><td>FALSE</td><td>Psychiatric</td><td></td><td></td><td>353764</td><td>143332</td><td>210432</td><td>UK Biobank</td><td>https://docs.google.com/spreadsheets/d/1kvPoupSzsSFBNSztMzl04xMoSC3Kcx3CrjVf4yBmESU/edit?ts=5b5f17db#gid=227859291</td><td>PHESANT Transformation:1960_0|| CAT-SINGLE || Inc(>=10): 0(210432) || Inc(>=10): 1(143332) || CAT-SINGLE-BINARY || sample 210432/143332(353764) ||-Notes:ACE touchscreen question Do you often feel 'fed-up'? If the participant activated the Help button they were shown the message: Work through these questions quickly and do not think about the exact meaning of the question-Variable type:binary</td></tr>
<tr><td>binary.1970.txt</td><td>1710174270056F5 forCTG.txt.gz</td><td>-0.0277</td><td>0.0353</td><td>-0.7847</td><td>0.4327</td><td>0.06599</td><td>0.003875</td><td>1.003</td><td>0.01683</td><td>-0.02976</td><td>0.01058</td><td>Nervous feelings</td><td>FALSE</td><td></td><td></td><td></td><td>351829</td><td>83120</td><td>268709</td><td>UK Biobank</td><td>https://docs.google.com/spreadsheets/d/1kvPoupSzsSFBNSztMzl04xMoSC3Kcx3CrjVf4yBmESU/edit?ts=5b5f17db#gid=227859291</td><td>PHESANT Transformation:1970_0|| CAT-SINGLE || Inc(>=10): 0(268709) || Inc(>=10): 1(83120) || CAT-SINGLE-BINARY || sample 268709/83120(351829) ||-Notes:ACE touchscreen question Would you call yourself a nervous person? If the participant activated the Help button they were shown the message: Work through these questions quickly and do not think about the exact meaning of the question-Variable type:binary</td></tr>
<tr><td>binary.1980.txt</td><td>1710174270056F5 forCTG.txt.gz</td><td>-0.01285</td><td>0.03399</td><td>-0.378</td><td>0.7055</td><td>0.08028</td><td>0.004812</td><td>0.986</td><td>0.01828</td><td>-0.02918</td><td>0.01107</td><td>Worrier / anxious feelings</td><td>FALSE</td><td></td><td></td><td></td><td>351833</td><td>199463</td><td>152370</td><td>UK Biobank</td><td>https://docs.google.com/spreadsheets/d/1kvPoupSzsSFBNSztMzl04xMoSC3Kcx3CrjVf4yBmESU/edit?ts=5b5f17db#gid=227859291</td><td>PHESANT Transformation:1980_0|| CAT-SINGLE || Inc(>=10): 0(152370) || Inc(>=10): 1(199463) || CAT-SINGLE-BINARY || sample 152370/199463(351833) ||-Notes:ACE touchscreen question Are you a worrier? If the participant activated the Help button they were shown the message: Work through these questions quickly and do not think about the exact meaning of the question-Variable type:binary</td></tr>
<tr><td>binary.1990.txt</td><td>1710174270056F5 forCTG.txt.gz</td><td>0.1332</td><td>0.03604</td><td>3.697</td><td>0.000218</td><td>0.05791</td><td>0.003309</td><td>0.987</td><td>0.0143</td><td>-0.01159</td><td>0.00932</td><td>Tense / 'highly strung'</td><td>FALSE</td><td></td><td></td><td></td><td>350159</td><td>60513</td><td>289646</td><td>UK Biobank</td><td>https://docs.google.com/spreadsheets/d/1kvPoupSzsSFBNSztMzl04xMoSC3Kcx3CrjVf4yBmESU/edit?ts=5b5f17db#gid=227859291</td><td>PHESANT Transformation:1990_0|| CAT-SINGLE || Inc(>=10): 0(289646) || Inc(>=10): 1(60513) || CAT-SINGLE-BINARY || sample 289646/60513(350159) ||-Notes:ACE touchscreen question Would you call yourself tense or 'highly strung'? If the participant activated the Help button they were shown the message: Work through these questions quickly and do not think about the exact meaning of the question-Variable type:binary</td></tr>
<tr><td>binary.2000.txt</td><td>1710174270056F5 forCTG.txt.gz</td><td>0.01673</td><td>0.03689</td><td>0.4534</td><td>0.6502</td><td>0.06457</td><td>0.003787</td><td>1.012</td><td>0.01624</td><td>-0.01324</td><td>0.009963</td><td>Worry too long after embarrassment</td><td>FALSE</td><td></td><td></td><td></td><td>346527</td><td>165310</td><td>181217</td><td>UK Biobank</td><td>https://docs.google.com/spreadsheets/d/1kvPoupSzsSFBNSztMzl04xMoSC3Kcx3CrjVf4yBmESU/edit?ts=5b5f17db#gid=227859291</td><td>PHESANT Transformation:2000_0|| CAT-SINGLE || Inc(>=10): 0(181217) || Inc(>=10): 1(165310) || CAT-SINGLE-BINARY || sample 181217/165310(346527) ||-Notes:ACE touchscreen question Do you worry too long after an embarrassing experience? If the participant activated the Help button they were shown the message: Work through these questions quickly and do not think about the exact meaning of the question-Variable type:binary</td></tr>
<tr><td>binary.20001_1059.txt</td><td>1710174270056F5 forCTG.txt.gz</td><td>-0.106</td><td>0.07925</td><td>-1.338</td><td>0.181</td><td>0.005766</td><td>0.001753</td><td>0.993</td><td>0.009557</td><td>0.009993</td><td>0.007744</td><td>Cancer code, self-reported: malignant melanoma</td><td>FALSE</td><td></td><td></td><td></td><td>361141</td><td>2898</td><td>358243</td><td>UK Biobank</td><td>https://docs.google.com/spreadsheets/d/1kvPoupSzsSFBNSztMzl04xMoSC3Kcx3CrjVf4yBmESU/edit?ts=5b5f17db#gid=227859291</td><td>PHESANT Transformation:20001_0 || CAT-MUL-BINARY-VAR 1059 || Indicator name x134_0_0 || Remove indicator var NAs: 53 || Remove indicator var <0: 0 || Removed 0 examples != 1059 but with missing value (<0) || sample 358243/2898(361141) ||-Notes:Code for cancer. If the participant was uncertain of the type of cancer they had had, then they described it to the interviewer (a trained nurse) who attempted to place it within the coding tree. If the cancer could not be located in the coding tree then the interviewer entered a free-text description of it. These free-text descriptions were subsequently examined by a doctor and, where possible, matched to entries in the coding tree. Free-text descriptions which could not be matched with very high probability have been marked as unclassifiable.-Variable type:binary</td></tr>
<tr><td>binary.20001_1061.txt</td><td>1710174270056F5 forCTG.txt.gz</td><td>0.1033</td><td>0.05407</td><td>1.91</td><td>0.05614</td><td>0.009662</td><td>0.002134</td><td>1.002</td><td>0.0118</td><td>-0.01944</td><td>0.006997</td><td>Cancer code, self-reported: basal cell carcinoma</td><td>FALSE</td><td></td><td></td><td></td><td>361141</td><td>3441</td><td>357700</td><td>UK Biobank</td><td>https://docs.google.com/spreadsheets/d/1kvPoupSzsSFBNSztMzl04xMoSC3Kcx3CrjVf4yBmESU/edit?ts=5b5f17db#gid=227859291</td><td>PHESANT Transformation:20001_0 || CAT-MUL-BINARY-VAR 1061 || Indicator name x134_0_0 || Remove indicator var NAs: 53 || Remove indicator var <0: 0 || Removed 0 examples != 1061 but with missing value (<0) || sample 357700/3441(361141) ||-Notes:Code for cancer. If the participant was uncertain of the type of cancer they had had, then they described it to the interviewer (a trained nurse) who attempted to place it within the coding tree. If the cancer could not be located in the coding tree then the interviewer entered a free-text description of it. These free-text descriptions were subsequently examined by a doctor and, where possible, matched to entries in the coding tree. Free-text descriptions which could not be matched with very high probability have been marked as unclassifiable.-Variable type:binary</td></tr>
<tr><td>binary.20002_1065.txt</td><td>1710174270056F5 forCTG.txt.gz</td><td>0.1347</td><td>0.0302</td><td>4.46</td><td>8.19E-06</td><td>0.116</td><td>0.006207</td><td>1.092</td><td>0.02906</td><td>-0.001693</td><td>0.01111</td><td>Non-cancer illness code, self-reported: hypertension</td><td>FALSE</td><td>Cardiac</td><td></td><td></td><td>361141</td><td>93560</td><td>267581</td><td>UK Biobank</td><td>https://docs.google.com/spreadsheets/d/1kvPoupSzsSFBNSztMzl04xMoSC3Kcx3CrjVf4yBmESU/edit?ts=5b5f17db#gid=227859291</td><td>PHESANT Transformation:20002_0 || CAT-MUL-BINARY-VAR 1065 || Indicator name x135_0_0 || Remove indicator var NAs: 53 || Remove indicator var <0: 0 || Removed 0 examples != 1065 but with missing value (<0) || sample 267581/93560(361141) ||-Notes:Code for non-cancer illness. If the participant was uncertain of the type of illness they had had, then they described it to the interviewer (a trained nurse) who attempted to place it within the coding tree. If the illness could not be located in the coding tree then the interviewer entered a free-text description of it. These free-text descriptions were subsequently examined by a doctor and, where possible, matched to entries in the coding tree. Free-text descriptions which could not be matched with very high probability have been marked as unclassifiable.-Variable type:binary</td></tr>
<tr><td>binary.20002_1067.txt</td><td>1710174270056F5 forCTG.txt.gz</td><td>0.08518</td><td>0.08931</td><td>0.9537</td><td>0.3402</td><td>0.00419</td><td>0.001737</td><td>0.98</td><td>0.01074</td><td>0.006663</td><td>0.007682</td><td>Non-cancer illness code, self-reported: peripheral vascular disease</td><td>FALSE</td><td></td><td></td><td></td><td>361141</td><td>664</td><td>360477</td><td>UK Biobank</td><td>https://docs.google.com/spreadsheets/d/1kvPoupSzsSFBNSztMzl04xMoSC3Kcx3CrjVf4yBmESU/edit?ts=5b5f17db#gid=227859291</td><td>PHESANT Transformation:20002_0 || CAT-MUL-BINARY-VAR 1067 || Indicator name x135_0_0 || Remove indicator var NAs: 53 || Remove indicator var <0: 0 || Removed 0 examples != 1067 but with missing value (<0) || sample 360477/664(361141) ||-Notes:Code for non-cancer illness. If the participant was uncertain of the type of illness they had had, then they described it to the interviewer (a trained nurse) who attempted to place it within the coding tree. If the illness could not be located in the coding tree then the interviewer entered a free-text description of it. These free-text descriptions were subsequently examined by a doctor and, where possible, matched to entries in the coding tree. Free-text descriptions which could not be matched with very high probability have been marked as unclassifiable.-Variable type:binary</td></tr>
</table>

<table>
<tbody>
<tr><td>binary.20002_1075.txt</td><td>1710174270056F5forCTG.txt.gz</td><td>0.2054</td><td>0.04936</td><td>4.161</td><td>3.17E-05</td><td>0.01899</td><td>0.002143</td><td>1.014</td><td>0.01076</td><td>0.001469</td><td>0.008741</td><td>Non-cancer illness code, self-reported: heart attack/myocardial infarction</td><td>FALSE</td><td>Cardiac</td><td></td><td></td><td>361141</td><td>8239</td><td>352902</td><td>UK Biobank</td><td>https://docs.google.com/spreadsheets/d/1kvPoupSzsSFBNSztMzl04xMoSC3Kcx3CrjVf4yBmESU/edit?ts=5b5f17db#gid=227859291</td><td>PHESANT Transformation:20002_0 | | CAT-MUL-BINARY-VAR 1075 | | Indicator name x135_0_0 | | Remove indicator var NAs: 53 | | Remove indicator var <0: 0 | | Removed 0 examples != 1075 but with missing value (<0) | | sample 352902/8239(361141) | |-Notes:Code for non-cancer illness. If the participant was uncertain of the type of illness they had had, then they described it to the interviewer (a trained nurse) who attempted to place it within the coding tree. If the illness could not be located in the coding tree then the interviewer entered a free-text description of it. These free-text descriptions were subsequently examined by a doctor and, where possible, matched to entries in the coding tree. Free-text descriptions which could not be matched with very high probability have been marked as unclassifiable.-Variable type:binary</td></tr>
<tr><td>binary.20002_1081.txt</td><td>1710174270056F5forCTG.txt.gz</td><td>0.3705</td><td>0.1336</td><td>2.773</td><td>0.005549</td><td>0.003524</td><td>0.00149</td><td>1.008</td><td>0.00896</td><td>0.003077</td><td>0.008837</td><td>Non-cancer illness code, self-reported: stroke</td><td>FALSE</td><td></td><td></td><td></td><td>361141</td><td>4836</td><td>356305</td><td>UK Biobank</td><td>https://docs.google.com/spreadsheets/d/1kvPoupSzsSFBNSztMzl04xMoSC3Kcx3CrjVf4yBmESU/edit?ts=5b5f17db#gid=227859291</td><td>PHESANT Transformation:20002_0 | | CAT-MUL-BINARY-VAR 1081 | | Indicator name x135_0_0 | | Remove indicator var NAs: 53 | | Remove indicator var <0: 0 | | Removed 0 examples != 1081 but with missing value (<0) | | sample 356305/4836(361141) | |-Notes:Code for non-cancer illness. If the participant was uncertain of the type of illness they had had, then they described it to the interviewer (a trained nurse) who attempted to place it within the coding tree. If the illness could not be located in the coding tree then the interviewer entered a free-text description of it. These free-text descriptions were subsequently examined by a doctor and, where possible, matched to entries in the coding tree. Free-text descriptions which could not be matched with very high probability have been marked as unclassifiable.-Variable type:binary</td></tr>
<tr><td>binary.20002_1087.txt</td><td>1710174270056F5forCTG.txt.gz</td><td>0.08749</td><td>0.1164</td><td>0.7516</td><td>0.4523</td><td>0.002354</td><td>0.00157</td><td>0.992</td><td>0.01024</td><td>0.002166</td><td>0.008092</td><td>Non-cancer illness code, self-reported: leg claudication/ intermittent claudication</td><td>FALSE</td><td></td><td></td><td></td><td>361141</td><td>277</td><td>360864</td><td>UK Biobank</td><td>https://docs.google.com/spreadsheets/d/1kvPoupSzsSFBNSztMzl04xMoSC3Kcx3CrjVf4yBmESU/edit?ts=5b5f17db#gid=227859291</td><td>PHESANT Transformation:20002_0 | | CAT-MUL-BINARY-VAR 1087 | | Indicator name x135_0_0 | | Remove indicator var NAs: 53 | | Remove indicator var <0: 0 | | Removed 0 examples != 1087 but with missing value (<0) | | sample 360864/277(361141) | |-Notes:Code for non-cancer illness. If the participant was uncertain of the type of illness they had had, then they described it to the interviewer (a trained nurse) who attempted to place it within the coding tree. If the illness could not be located in the coding tree then the interviewer entered a free-text description of it. These free-text descriptions were subsequently examined by a doctor and, where possible, matched to entries in the coding tree. Free-text descriptions which could not be matched with very high probability have been marked as unclassifiable.-Variable type:binary</td></tr>
<tr><td>binary.20002_1093.txt</td><td>1710174270056F5forCTG.txt.gz</td><td>0.3646</td><td>0.09419</td><td>3.87</td><td>0.000109</td><td>0.005102</td><td>0.001625</td><td>1.013</td><td>0.01027</td><td>-0.01268</td><td>0.007726</td><td>Non-cancer illness code, self-reported: pulmonary embolism +/- dvt</td><td>FALSE</td><td></td><td></td><td></td><td>361141</td><td>2999</td><td>358142</td><td>UK Biobank</td><td>https://docs.google.com/spreadsheets/d/1kvPoupSzsSFBNSztMzl04xMoSC3Kcx3CrjVf4yBmESU/edit?ts=5b5f17db#gid=227859291</td><td>PHESANT Transformation:20002_0 | | CAT-MUL-BINARY-VAR 1093 | | Indicator name x135_0_0 | | Remove indicator var NAs: 53 | | Remove indicator var <0: 0 | | Removed 0 examples != 1093 but with missing value (<0) | | sample 358142/2999(361141) | |-Notes:Code for non-cancer illness. If the participant was uncertain of the type of illness they had had, then they described it to the interviewer (a trained nurse) who attempted to place it within the coding tree. If the illness could not be located in the coding tree then the interviewer entered a free-text description of it. These free-text descriptions were subsequently examined by a doctor and, where possible, matched to entries in the coding tree. Free-text descriptions which could not be matched with very high probability have been marked as unclassifiable.-Variable type:binary</td></tr>
<tr><td>binary.20002_1094.txt</td><td>1710174270056F5forCTG.txt.gz</td><td>0.2566</td><td>0.07439</td><td>3.45</td><td>0.000562</td><td>0.008652</td><td>0.001896</td><td>1.024</td><td>0.01407</td><td>-0.000582</td><td>0.007888</td><td>Non-cancer illness code, self-reported: deep venous thrombosis (dvt)</td><td>FALSE</td><td></td><td></td><td></td><td>361141</td><td>7237</td><td>353904</td><td>UK Biobank</td><td>https://docs.google.com/spreadsheets/d/1kvPoupSzsSFBNSztMzl04xMoSC3Kcx3CrjVf4yBmESU/edit?ts=5b5f17db#gid=227859291</td><td>PHESANT Transformation:20002_0 | | CAT-MUL-BINARY-VAR 1094 | | Indicator name x135_0_0 | | Remove indicator var NAs: 53 | | Remove indicator var <0: 0 | | Removed 0 examples != 1094 but with missing value (<0) | | sample 353904/7237(361141) | |-Notes:Code for non-cancer illness. If the participant was uncertain of the type of illness they had had, then they described it to the interviewer (a trained nurse) who attempted to place it within the coding tree. If the illness could not be located in the coding tree then the interviewer entered a free-text description of it. These free-text descriptions were subsequently examined by a doctor and, where possible, matched to entries in the coding tree. Free-text descriptions which could not be matched with very high probability have been marked as unclassifiable.-Variable type:binary</td></tr>
<tr><td>binary.20002_1111.txt</td><td>1710174270056F5forCTG.txt.gz</td><td>0.1353</td><td>0.03528</td><td>3.835</td><td>0.000126</td><td>0.0575</td><td>0.007474</td><td>1.02</td><td>0.02776</td><td>0.01735</td><td>0.009368</td><td>Non-cancer illness code, self-reported: asthma</td><td>FALSE</td><td></td><td></td><td></td><td>361141</td><td>41934</td><td>319207</td><td>UK Biobank</td><td>https://docs.google.com/spreadsheets/d/1kvPoupSzsSFBNSztMzl04xMoSC3Kcx3CrjVf4yBmESU/edit?ts=5b5f17db#gid=227859291</td><td>PHESANT Transformation:20002_0 | | CAT-MUL-BINARY-VAR 1111 | | Indicator name x135_0_0 | | Remove indicator var NAs: 53 | | Remove indicator var <0: 0 | | Removed 0 examples != 1111 but with missing value (<0) | | sample 319207/41934(361141) | |-Notes:Code for non-cancer illness. If the participant was uncertain of the type of illness they had had, then they described it to the interviewer (a trained nurse) who attempted to place it within the coding tree. If the illness could not be located in the coding tree then the interviewer entered a free-text description of it. These free-text descriptions were subsequently examined by a doctor and, where possible, matched to entries in the coding tree. Free-text descriptions which could not be matched with very high probability have been marked as unclassifiable.-Variable type:binary</td></tr>
<tr><td>binary.20002_1112.txt</td><td>1710174270056F5forCTG.txt.gz</td><td>0.4586</td><td>0.1194</td><td>3.84</td><td>0.000123</td><td>0.004302</td><td>0.001638</td><td>1</td><td>0.009336</td><td>-0.008559</td><td>0.007763</td><td>Non-cancer illness code, self-reported: chronic obstructive airways disease/copd</td><td>FALSE</td><td></td><td></td><td></td><td>361141</td><td>1285</td><td>359856</td><td>UK Biobank</td><td>https://docs.google.com/spreadsheets/d/1kvPoupSzsSFBNSztMzl04xMoSC3Kcx3CrjVf4yBmESU/edit?ts=5b5f17db#gid=227859291</td><td>PHESANT Transformation:20002_0 | | CAT-MUL-BINARY-VAR 1112 | | Indicator name x135_0_0 | | Remove indicator var NAs: 53 | | Remove indicator var <0: 0 | | Removed 0 examples != 1112 but with missing value (<0) | | sample 359856/1285(361141) | |-Notes:Code for non-cancer illness. If the participant was uncertain of the type of illness they had had, then they described it to the interviewer (a trained nurse) who attempted to place it within the coding tree. If the illness could not be located in the coding tree then the interviewer entered a free-text description of it. These free-text descriptions were subsequently examined by a doctor and, where possible, matched to entries in the coding tree. Free-text descriptions which could not be matched with very high probability have been marked as unclassifiable.-Variable type:binary</td></tr>
<tr><td>binary.20002_1113.txt</td><td>1710174270056F5forCTG.txt.gz</td><td>0.3479</td><td>0.05816</td><td>5.981</td><td>2.21E-09</td><td>0.01141</td><td>0.001947</td><td>0.976</td><td>0.01088</td><td>0.006514</td><td>0.007834</td><td>Non-cancer illness code, self-reported: emphysema/chronic bronchitis</td><td>FALSE</td><td>Pulmonary</td><td></td><td></td><td>361141</td><td>5031</td><td>356110</td><td>UK Biobank</td><td>https://docs.google.com/spreadsheets/d/1kvPoupSzsSFBNSztMzl04xMoSC3Kcx3CrjVf4yBmESU/edit?ts=5b5f17db#gid=227859291</td><td>PHESANT Transformation:20002_0 | | CAT-MUL-BINARY-VAR 1113 | | Indicator name x135_0_0 | | Remove indicator var NAs: 53 | | Remove indicator var <0: 0 | | Removed 0 examples != 1113 but with missing value (<0) | | sample 356110/5031(361141) | |-Notes:Code for non-cancer illness. If the participant was uncertain of the type of illness they had had, then they described it to the interviewer (a trained nurse) who attempted to place it within the coding tree. If the illness could not be located in the coding tree then the interviewer entered a free-text description of it. These free-text descriptions were subsequently examined by a doctor and, where possible, matched to entries in the coding tree. Free-text descriptions which could not be matched with very high probability have been marked as unclassifiable.-Variable type:binary</td></tr>
<tr><td>binary.20002_1123.txt</td><td>1710174270056F5forCTG.txt.gz</td><td>0.1208</td><td>0.08646</td><td>1.398</td><td>0.1622</td><td>0.004719</td><td>0.001586</td><td>0.998</td><td>0.01017</td><td>0.0154</td><td>0.008081</td><td>Non-cancer illness code, self-reported: sleep apnoea</td><td>FALSE</td><td></td><td></td><td></td><td>361141</td><td>1163</td><td>359978</td><td>UK Biobank</td><td>https://docs.google.com/spreadsheets/d/1kvPoupSzsSFBNSztMzl04xMoSC3Kcx3CrjVf4yBmESU/edit?ts=5b5f17db#gid=227859291</td><td>PHESANT Transformation:20002_0 | | CAT-MUL-BINARY-VAR 1123 | | Indicator name x135_0_0 | | Remove indicator var NAs: 53 | | Remove indicator var <0: 0 | | Removed 0 examples != 1123 but with missing value (<0) | | sample 359978/1163(361141) | |-Notes:Code for non-cancer illness. If the participant was uncertain of the type of illness they had had, then they described it to the interviewer (a trained nurse) who attempted to place it within the coding tree. If the illness could not be located in the coding tree then the interviewer entered a free-text description of it. These free-text descriptions were subsequently examined by a doctor and, where possible, matched to entries in the coding tree. Free-text descriptions which could not be matched with very high probability have been marked as unclassifiable.-Variable type:binary</td></tr>
<tr><td>binary.20002_1137.txt</td><td>1710174270056F5forCTG.txt.gz</td><td>0.06935</td><td>0.1117</td><td>0.621</td><td>0.5346</td><td>0.002912</td><td>0.001485</td><td>0.989</td><td>0.009795</td><td>-0.001946</td><td>0.009029</td><td>Non-cancer illness code, self-reported: other abdominal problem</td><td>FALSE</td><td></td><td></td><td></td><td>361141</td><td>422</td><td>360719</td><td>UK Biobank</td><td>https://docs.google.com/spreadsheets/d/1kvPoupSzsSFBNSztMzl04xMoSC3Kcx3CrjVf4yBmESU/edit?ts=5b5f17db#gid=227859291</td><td>PHESANT Transformation:20002_0 | | CAT-MUL-BINARY-VAR 1137 | | Indicator name x135_0_0 | | Remove indicator var NAs: 53 | | Remove indicator var <0: 0 | | Removed 0 examples != 1137 but with missing value (<0) | | sample 360719/422(361141) | |-Notes:Code for non-cancer illness. If the participant was uncertain of the type of illness they had had, then they described it to the interviewer (a trained nurse) who attempted to place it within the coding tree. If the illness could not be located in the coding tree then the interviewer entered a free-text description of it. These free-text descriptions were subsequently examined by a doctor and, where possible, matched to entries in the coding tree. Free-text descriptions which could not be matched with very high probability have been marked as unclassifiable.-Variable type:binary</td></tr>
</tbody>
</table>

<table>
<tr><td>binary.20002_1138.txt</td><td>1710174270056F5forCTG.txt.gz</td><td>0.2893</td><td>0.05259</td><td>5.501</td><td>3.79E-08</td><td>0.01079</td><td>0.001747</td><td>1.017</td><td>0.009961</td><td>0.00508</td><td>0.007788</td><td>Non-cancer illness code, self-reported: gastro-oesophageal reflux (gord) / gastric reflux</td><td>FALSE</td><td>Gastric</td><td></td><td></td><td>361141</td><td>15210</td><td>345931</td><td>UK Biobank</td><td>https://docs.google.com/spreadsheets/d/1kvPoupSzsSFBNSztMzl04xMoSC3Kcx3CrjVf4yBmESU/edit?ts=5b5f17db#gid=227859291</td><td>PHESANT Transformation:20002_0 | | CAT-MUL-BINARY-VAR 1138 | | Indicator name x135_0_0 | | Remove indicator var NAs: 53 | | Remove indicator var <0: 0 | | Removed 0 examples != 1138 but with missing value (<0) | | sample 345931/15210(361141) | |-Notes:Code for non-cancer illness. If the participant was uncertain of the type of illness they had had, then they described it to the interviewer (a trained nurse) who attempted to place it within the coding tree. If the illness could not be located in the coding tree then the interviewer entered a free-text description of it. These free-text descriptions were subsequently examined by a doctor and, where possible, matched to entries in the coding tree. Free-text descriptions which could not be matched with very high probability have been marked as unclassifiable.-Variable type:binary</td></tr>
<tr><td>binary.20002_1154.txt</td><td>1710174270056F5forCTG.txt.gz</td><td>0.3351</td><td>0.09593</td><td>3.493</td><td>0.000477</td><td>0.006392</td><td>0.001884</td><td>1.013</td><td>0.01056</td><td>-0.001749</td><td>0.009001</td><td>Non-cancer illness code, self-reported: irritable bowel syndrome</td><td>FALSE</td><td></td><td></td><td></td><td>361141</td><td>8537</td><td>352604</td><td>UK Biobank</td><td>https://docs.google.com/spreadsheets/d/1kvPoupSzsSFBNSztMzl04xMoSC3Kcx3CrjVf4yBmESU/edit?ts=5b5f17db#gid=227859291</td><td>PHESANT Transformation:20002_0 | | CAT-MUL-BINARY-VAR 1154 | | Indicator name x135_0_0 | | Remove indicator var NAs: 53 | | Remove indicator var <0: 0 | | Removed 0 examples != 1154 but with missing value (<0) | | sample 352604/8537(361141) | |-Notes:Code for non-cancer illness. If the participant was uncertain of the type of illness they had had, then they described it to the interviewer (a trained nurse) who attempted to place it within the coding tree. If the illness could not be located in the coding tree then the interviewer entered a free-text description of it. These free-text descriptions were subsequently examined by a doctor and, where possible, matched to entries in the coding tree. Free-text descriptions which could not be matched with very high probability have been marked as unclassifiable.-Variable type:binary</td></tr>
<tr><td>binary.20002_1162.txt</td><td>1710174270056F5forCTG.txt.gz</td><td>0.01378</td><td>0.07169</td><td>0.1922</td><td>0.8476</td><td>0.007778</td><td>0.001944</td><td>1.026</td><td>0.01619</td><td>0.0288</td><td>0.008747</td><td>Non-cancer illness code, self-reported: cholelithiasis/gall stones</td><td>FALSE</td><td></td><td></td><td></td><td>361141</td><td>5941</td><td>355200</td><td>UK Biobank</td><td>https://docs.google.com/spreadsheets/d/1kvPoupSzsSFBNSztMzl04xMoSC3Kcx3CrjVf4yBmESU/edit?ts=5b5f17db#gid=227859291</td><td>PHESANT Transformation:20002_0 | | CAT-MUL-BINARY-VAR 1162 | | Indicator name x135_0_0 | | Remove indicator var NAs: 53 | | Remove indicator var <0: 0 | | Removed 0 examples != 1162 but with missing value (<0) | | sample 355200/5941(361141) | |-Notes:Code for non-cancer illness. If the participant was uncertain of the type of illness they had had, then they described it to the interviewer (a trained nurse) who attempted to place it within the coding tree. If the illness could not be located in the coding tree then the interviewer entered a free-text description of it. These free-text descriptions were subsequently examined by a doctor and, where possible, matched to entries in the coding tree. Free-text descriptions which could not be matched with very high probability have been marked as unclassifiable.-Variable type:binary</td></tr>
<tr><td>binary.20002_1196.txt</td><td>1710174270056F5forCTG.txt.gz</td><td>0.03535</td><td>0.07543</td><td>0.4686</td><td>0.6394</td><td>0.005411</td><td>0.001506</td><td>0.973</td><td>0.009224</td><td>-0.001898</td><td>0.007626</td><td>Non-cancer illness code, self-reported: urinary tract infection/kidney infection</td><td>FALSE</td><td></td><td></td><td></td><td>361141</td><td>1698</td><td>359443</td><td>UK Biobank</td><td>https://docs.google.com/spreadsheets/d/1kvPoupSzsSFBNSztMzl04xMoSC3Kcx3CrjVf4yBmESU/edit?ts=5b5f17db#gid=227859291</td><td>PHESANT Transformation:20002_0 | | CAT-MUL-BINARY-VAR 1196 | | Indicator name x135_0_0 | | Remove indicator var NAs: 53 | | Remove indicator var <0: 0 | | Removed 0 examples != 1196 but with missing value (<0) | | sample 359443/1698(361141) | |-Notes:Code for non-cancer illness. If the participant was uncertain of the type of illness they had had, then they described it to the interviewer (a trained nurse) who attempted to place it within the coding tree. If the illness could not be located in the coding tree then the interviewer entered a free-text description of it. These free-text descriptions were subsequently examined by a doctor and, where possible, matched to entries in the coding tree. Free-text descriptions which could not be matched with very high probability have been marked as unclassifiable.-Variable type:binary</td></tr>
<tr><td>binary.20002_1197.txt</td><td>1710174270056F5forCTG.txt.gz</td><td>0.1266</td><td>0.06551</td><td>1.933</td><td>0.05325</td><td>0.008557</td><td>0.001846</td><td>1.005</td><td>0.01059</td><td>-0.002499</td><td>0.007887</td><td>Non-cancer illness code, self-reported: kidney stone/ureter stone/bladder stone</td><td>FALSE</td><td></td><td></td><td></td><td>361141</td><td>2863</td><td>358278</td><td>UK Biobank</td><td>https://docs.google.com/spreadsheets/d/1kvPoupSzsSFBNSztMzl04xMoSC3Kcx3CrjVf4yBmESU/edit?ts=5b5f17db#gid=227859291</td><td>PHESANT Transformation:20002_0 | | CAT-MUL-BINARY-VAR 1197 | | Indicator name x135_0_0 | | Remove indicator var NAs: 53 | | Remove indicator var <0: 0 | | Removed 0 examples != 1197 but with missing value (<0) | | sample 358278/2863(361141) | |-Notes:Code for non-cancer illness. If the participant was uncertain of the type of illness they had had, then they described it to the interviewer (a trained nurse) who attempted to place it within the coding tree. If the illness could not be located in the coding tree then the interviewer entered a free-text description of it. These free-text descriptions were subsequently examined by a doctor and, where possible, matched to entries in the coding tree. Free-text descriptions which could not be matched with very high probability have been marked as unclassifiable.-Variable type:binary</td></tr>
<tr><td>binary.20002_1202.txt</td><td>1710174270056F5forCTG.txt.gz</td><td>0.217</td><td>0.1629</td><td>1.332</td><td>0.1829</td><td>0.002006</td><td>0.001523</td><td>0.996</td><td>0.009298</td><td>-0.00238</td><td>0.008363</td><td>Non-cancer illness code, self-reported: urinary frequency / incontinence</td><td>FALSE</td><td></td><td></td><td></td><td>361141</td><td>1252</td><td>359889</td><td>UK Biobank</td><td>https://docs.google.com/spreadsheets/d/1kvPoupSzsSFBNSztMzl04xMoSC3Kcx3CrjVf4yBmESU/edit?ts=5b5f17db#gid=227859291</td><td>PHESANT Transformation:20002_0 | | CAT-MUL-BINARY-VAR 1202 | | Indicator name x135_0_0 | | Remove indicator var NAs: 53 | | Remove indicator var <0: 0 | | Removed 0 examples != 1202 but with missing value (<0) | | sample 359889/1252(361141) | |-Notes:Code for non-cancer illness. If the participant was uncertain of the type of illness they had had, then they described it to the interviewer (a trained nurse) who attempted to place it within the coding tree. If the illness could not be located in the coding tree then the interviewer entered a free-text description of it. These free-text descriptions were subsequently examined by a doctor and, where possible, matched to entries in the coding tree. Free-text descriptions which could not be matched with very high probability have been marked as unclassifiable.-Variable type:binary</td></tr>
<tr><td>binary.20002_1220.txt</td><td>1710174270056F5forCTG.txt.gz</td><td>0.2015</td><td>0.04192</td><td>4.806</td><td>1.54E-06</td><td>0.03412</td><td>0.002749</td><td>1.057</td><td>0.01463</td><td>0.007263</td><td>0.009538</td><td>Non-cancer illness code, self-reported: diabetes</td><td>FALSE</td><td>Metabolic</td><td></td><td></td><td>361141</td><td>14114</td><td>347027</td><td>UK Biobank</td><td>https://docs.google.com/spreadsheets/d/1kvPoupSzsSFBNSztMzl04xMoSC3Kcx3CrjVf4yBmESU/edit?ts=5b5f17db#gid=227859291</td><td>PHESANT Transformation:20002_0 | | CAT-MUL-BINARY-VAR 1220 | | Indicator name x135_0_0 | | Remove indicator var NAs: 53 | | Remove indicator var <0: 0 | | Removed 0 examples != 1220 but with missing value (<0) | | sample 347027/14114(361141) | |-Notes:Code for non-cancer illness. If the participant was uncertain of the type of illness they had had, then they described it to the interviewer (a trained nurse) who attempted to place it within the coding tree. If the illness could not be located in the coding tree then the interviewer entered a free-text description of it. These free-text descriptions were subsequently examined by a doctor and, where possible, matched to entries in the coding tree. Free-text descriptions which could not be matched with very high probability have been marked as unclassifiable.-Variable type:binary</td></tr>
<tr><td>binary.20002_1223.txt</td><td>1710174270056F5forCTG.txt.gz</td><td>0.2062</td><td>0.07741</td><td>2.664</td><td>0.007721</td><td>0.005607</td><td>0.001554</td><td>1.016</td><td>0.01079</td><td>0.007812</td><td>0.007782</td><td>Non-cancer illness code, self-reported: type 2 diabetes</td><td>FALSE</td><td></td><td></td><td></td><td>361141</td><td>2292</td><td>358849</td><td>UK Biobank</td><td>https://docs.google.com/spreadsheets/d/1kvPoupSzsSFBNSztMzl04xMoSC3Kcx3CrjVf4yBmESU/edit?ts=5b5f17db#gid=227859291</td><td>PHESANT Transformation:20002_0 | | CAT-MUL-BINARY-VAR 1223 | | Indicator name x135_0_0 | | Remove indicator var NAs: 53 | | Remove indicator var <0: 0 | | Removed 0 examples != 1223 but with missing value (<0) | | sample 358849/2292(361141) | |-Notes:Code for non-cancer illness. If the participant was uncertain of the type of illness they had had, then they described it to the interviewer (a trained nurse) who attempted to place it within the coding tree. If the illness could not be located in the coding tree then the interviewer entered a free-text description of it. These free-text descriptions were subsequently examined by a doctor and, where possible, matched to entries in the coding tree. Free-text descriptions which could not be matched with very high probability have been marked as unclassifiable.-Variable type:binary</td></tr>
<tr><td>binary.20002_1225.txt</td><td>1710174270056F5forCTG.txt.gz</td><td>-0.03602</td><td>0.07734</td><td>-0.4658</td><td>0.6414</td><td>0.005997</td><td>0.002011</td><td>1.014</td><td>0.01024</td><td>0.01005</td><td>0.008132</td><td>Non-cancer illness code, self-reported: hyperthyroidism/thyrotoxicosis</td><td>FALSE</td><td></td><td></td><td></td><td>361141</td><td>2730</td><td>358411</td><td>UK Biobank</td><td>https://docs.google.com/spreadsheets/d/1kvPoupSzsSFBNSztMzl04xMoSC3Kcx3CrjVf4yBmESU/edit?ts=5b5f17db#gid=227859291</td><td>PHESANT Transformation:20002_0 | | CAT-MUL-BINARY-VAR 1225 | | Indicator name x135_0_0 | | Remove indicator var NAs: 53 | | Remove indicator var <0: 0 | | Removed 0 examples != 1225 but with missing value (<0) | | sample 358411/2730(361141) | |-Notes:Code for non-cancer illness. If the participant was uncertain of the type of illness they had had, then they described it to the interviewer (a trained nurse) who attempted to place it within the coding tree. If the illness could not be located in the coding tree then the interviewer entered a free-text description of it. These free-text descriptions were subsequently examined by a doctor and, where possible, matched to entries in the coding tree. Free-text descriptions which could not be matched with very high probability have been marked as unclassifiable.-Variable type:binary</td></tr>
<tr><td>binary.20002_1226.txt</td><td>1710174270056F5forCTG.txt.gz</td><td>0.03867</td><td>0.04072</td><td>0.9497</td><td>0.3423</td><td>0.04745</td><td>0.006356</td><td>1.045</td><td>0.02325</td><td>0.01519</td><td>0.009586</td><td>Non-cancer illness code, self-reported: hypothyroidism/myxoedema</td><td>FALSE</td><td></td><td></td><td></td><td>361141</td><td>17574</td><td>343567</td><td>UK Biobank</td><td>https://docs.google.com/spreadsheets/d/1kvPoupSzsSFBNSztMzl04xMoSC3Kcx3CrjVf4yBmESU/edit?ts=5b5f17db#gid=227859291</td><td>PHESANT Transformation:20002_0 | | CAT-MUL-BINARY-VAR 1226 | | Indicator name x135_0_0 | | Remove indicator var NAs: 53 | | Remove indicator var <0: 0 | | Removed 0 examples != 1226 but with missing value (<0) | | sample 343567/17574(361141) | |-Notes:Code for non-cancer illness. If the participant was uncertain of the type of illness they had had, then they described it to the interviewer (a trained nurse) who attempted to place it within the coding tree. If the illness could not be located in the coding tree then the interviewer entered a free-text description of it. These free-text descriptions were subsequently examined by a doctor and, where possible, matched to entries in the coding tree. Free-text descriptions which could not be matched with very high probability have been marked as unclassifiable.-Variable type:binary</td></tr>
</table>

<table>
<tr><td>binary.20002_1261.txt</td><td>1710174270056F5forCTG.txt.gz</td><td>0.08146</td><td>0.1352</td><td>0.6027</td><td>0.5467</td><td>0.001744</td><td>0.001471</td><td>1.02</td><td>0.009362</td><td>-1.48E-05</td><td>0.0078</td><td>Non-cancer illness code, self-reported: multiple sclerosis</td><td>FALSE</td><td></td><td></td><td></td><td>361141</td><td>1326</td><td>359815</td><td>UK Biobank</td><td>https://docs.google.com/spreadsheets/d/1kvPoupSzsSFBNSztMzl04xMoSC3Kcx3CrjVf4yBmESU/edit?ts=5b5f17db#gid=227859291</td><td>PHESANT Transformation:20002_0 | | CAT-MUL-BINARY-VAR 1261 | | Indicator name x135_0_0 | | Remove indicator var NAs: 53 | | Remove indicator var <0: 0 | | Removed 0 examples != 1261 but with missing value (<0) | | sample 359815/1326(361141) | |-Notes:Code for non-cancer illness. If the participant was uncertain of the type of illness they had had, then they described it to the interviewer (a trained nurse) who attempted to place it within the coding tree. If the illness could not be located in the coding tree then the interviewer entered a free-text description of it. These free-text descriptions were subsequently examined by a doctor and, where possible, matched to entries in the coding tree. Free-text descriptions which could not be matched with very high probability have been marked as unclassifiable.-Variable type:binary</td></tr>
<tr><td>binary.20002_1266.txt</td><td>1710174270056F5forCTG.txt.gz</td><td>0.1104</td><td>0.1192</td><td>0.9267</td><td>0.3541</td><td>0.0033</td><td>0.001714</td><td>0.989</td><td>0.0109</td><td>0.007561</td><td>0.007936</td><td>Non-cancer illness code, self-reported: head injury</td><td>FALSE</td><td></td><td></td><td></td><td>361141</td><td>1230</td><td>359911</td><td>UK Biobank</td><td>https://docs.google.com/spreadsheets/d/1kvPoupSzsSFBNSztMzl04xMoSC3Kcx3CrjVf4yBmESU/edit?ts=5b5f17db#gid=227859291</td><td>PHESANT Transformation:20002_0 | | CAT-MUL-BINARY-VAR 1266 | | Indicator name x135_0_0 | | Remove indicator var NAs: 53 | | Remove indicator var <0: 0 | | Removed 0 examples != 1266 but with missing value (<0) | | sample 359911/1230(361141) | |-Notes:Code for non-cancer illness. If the participant was uncertain of the type of illness they had had, then they described it to the interviewer (a trained nurse) who attempted to place it within the coding tree. If the illness could not be located in the coding tree then the interviewer entered a free-text description of it. These free-text descriptions were subsequently examined by a doctor and, where possible, matched to entries in the coding tree. Free-text descriptions which could not be matched with very high probability have been marked as unclassifiable.-Variable type:binary</td></tr>
<tr><td>binary.20002_1277.txt</td><td>1710174270056F5forCTG.txt.gz</td><td>0.04009</td><td>0.05876</td><td>0.6823</td><td>0.4951</td><td>0.01222</td><td>0.002098</td><td>1.013</td><td>0.01178</td><td>-0.009101</td><td>0.008909</td><td>Non-cancer illness code, self-reported: glaucoma</td><td>FALSE</td><td></td><td></td><td></td><td>361141</td><td>3786</td><td>357355</td><td>UK Biobank</td><td>https://docs.google.com/spreadsheets/d/1kvPoupSzsSFBNSztMzl04xMoSC3Kcx3CrjVf4yBmESU/edit?ts=5b5f17db#gid=227859291</td><td>PHESANT Transformation:20002_0 | | CAT-MUL-BINARY-VAR 1277 | | Indicator name x135_0_0 | | Remove indicator var NAs: 53 | | Remove indicator var <0: 0 | | Removed 0 examples != 1277 but with missing value (<0) | | sample 357355/3786(361141) | |-Notes:Code for non-cancer illness. If the participant was uncertain of the type of illness they had had, then they described it to the interviewer (a trained nurse) who attempted to place it within the coding tree. If the illness could not be located in the coding tree then the interviewer entered a free-text description of it. These free-text descriptions were subsequently examined by a doctor and, where possible, matched to entries in the coding tree. Free-text descriptions which could not be matched with very high probability have been marked as unclassifiable.-Variable type:binary</td></tr>
<tr><td>binary.20002_1278.txt</td><td>1710174270056F5forCTG.txt.gz</td><td>0.1683</td><td>0.131</td><td>1.285</td><td>0.1987</td><td>0.002455</td><td>0.001614</td><td>1.01</td><td>0.009513</td><td>-0.001493</td><td>0.008599</td><td>Non-cancer illness code, self-reported: cataract</td><td>FALSE</td><td></td><td></td><td></td><td>361141</td><td>5045</td><td>356096</td><td>UK Biobank</td><td>https://docs.google.com/spreadsheets/d/1kvPoupSzsSFBNSztMzl04xMoSC3Kcx3CrjVf4yBmESU/edit?ts=5b5f17db#gid=227859291</td><td>PHESANT Transformation:20002_0 | | CAT-MUL-BINARY-VAR 1278 | | Indicator name x135_0_0 | | Remove indicator var NAs: 53 | | Remove indicator var <0: 0 | | Removed 0 examples != 1278 but with missing value (<0) | | sample 356096/5045(361141) | |-Notes:Code for non-cancer illness. If the participant was uncertain of the type of illness they had had, then they described it to the interviewer (a trained nurse) who attempted to place it within the coding tree. If the illness could not be located in the coding tree then the interviewer entered a free-text description of it. These free-text descriptions were subsequently examined by a doctor and, where possible, matched to entries in the coding tree. Free-text descriptions which could not be matched with very high probability have been marked as unclassifiable.-Variable type:binary</td></tr>
<tr><td>binary.20002_1281.txt</td><td>1710174270056F5forCTG.txt.gz</td><td>0.004258</td><td>0.1025</td><td>0.04152</td><td>0.9669</td><td>0.002974</td><td>0.001442</td><td>1.004</td><td>0.009084</td><td>-0.006596</td><td>0.007545</td><td>Non-cancer illness code, self-reported: retinal detachment</td><td>FALSE</td><td></td><td></td><td></td><td>361141</td><td>1219</td><td>359922</td><td>UK Biobank</td><td>https://docs.google.com/spreadsheets/d/1kvPoupSzsSFBNSztMzl04xMoSC3Kcx3CrjVf4yBmESU/edit?ts=5b5f17db#gid=227859291</td><td>PHESANT Transformation:20002_0 | | CAT-MUL-BINARY-VAR 1281 | | Indicator name x135_0_0 | | Remove indicator var NAs: 53 | | Remove indicator var <0: 0 | | Removed 0 examples != 1281 but with missing value (<0) | | sample 359922/1219(361141) | |-Notes:Code for non-cancer illness. If the participant was uncertain of the type of illness they had had, then they described it to the interviewer (a trained nurse) who attempted to place it within the coding tree. If the illness could not be located in the coding tree then the interviewer entered a free-text description of it. These free-text descriptions were subsequently examined by a doctor and, where possible, matched to entries in the coding tree. Free-text descriptions which could not be matched with very high probability have been marked as unclassifiable.-Variable type:binary</td></tr>
<tr><td>binary.20002_1286.txt</td><td>1710174270056F5forCTG.txt.gz</td><td>0.2677</td><td>0.04779</td><td>5.603</td><td>2.11E-08</td><td>0.01762</td><td>0.001823</td><td>1.005</td><td>0.01078</td><td>-0.005988</td><td>0.00844</td><td>Non-cancer illness code, self-reported: depression</td><td>FALSE</td><td>Psychiatric</td><td></td><td></td><td>361141</td><td>20648</td><td>340493</td><td>UK Biobank</td><td>https://docs.google.com/spreadsheets/d/1kvPoupSzsSFBNSztMzl04xMoSC3Kcx3CrjVf4yBmESU/edit?ts=5b5f17db#gid=227859291</td><td>PHESANT Transformation:20002_0 | | CAT-MUL-BINARY-VAR 1286 | | Indicator name x135_0_0 | | Remove indicator var NAs: 53 | | Remove indicator var <0: 0 | | Removed 0 examples != 1286 but with missing value (<0) | | sample 340493/20648(361141) | |-Notes:Code for non-cancer illness. If the participant was uncertain of the type of illness they had had, then they described it to the interviewer (a trained nurse) who attempted to place it within the coding tree. If the illness could not be located in the coding tree then the interviewer entered a free-text description of it. These free-text descriptions were subsequently examined by a doctor and, where possible, matched to entries in the coding tree. Free-text descriptions which could not be matched with very high probability have been marked as unclassifiable.-Variable type:binary</td></tr>
<tr><td>binary.20002_1287.txt</td><td>1710174270056F5forCTG.txt.gz</td><td>0.0101</td><td>0.07825</td><td>0.129</td><td>0.8973</td><td>0.005979</td><td>0.001445</td><td>0.999</td><td>0.008782</td><td>0.003915</td><td>0.00861</td><td>Non-cancer illness code, self-reported: anxiety/panic attacks</td><td>FALSE</td><td></td><td></td><td></td><td>361141</td><td>4961</td><td>356180</td><td>UK Biobank</td><td>https://docs.google.com/spreadsheets/d/1kvPoupSzsSFBNSztMzl04xMoSC3Kcx3CrjVf4yBmESU/edit?ts=5b5f17db#gid=227859291</td><td>PHESANT Transformation:20002_0 | | CAT-MUL-BINARY-VAR 1287 | | Indicator name x135_0_0 | | Remove indicator var NAs: 53 | | Remove indicator var <0: 0 | | Removed 0 examples != 1287 but with missing value (<0) | | sample 356180/4961(361141) | |-Notes:Code for non-cancer illness. If the participant was uncertain of the type of illness they had had, then they described it to the interviewer (a trained nurse) who attempted to place it within the coding tree. If the illness could not be located in the coding tree then the interviewer entered a free-text description of it. These free-text descriptions were subsequently examined by a doctor and, where possible, matched to entries in the coding tree. Free-text descriptions which could not be matched with very high probability have been marked as unclassifiable.-Variable type:binary</td></tr>
<tr><td>binary.20002_1291.txt</td><td>1710174270056F5forCTG.txt.gz</td><td>-0.02683</td><td>0.09056</td><td>-0.2963</td><td>0.767</td><td>0.004755</td><td>0.001601</td><td>1.004</td><td>0.009791</td><td>0.008758</td><td>0.008148</td><td>Non-cancer illness code, self-reported: mania/bipolar disorder/manic depression</td><td>FALSE</td><td></td><td></td><td></td><td>361141</td><td>1008</td><td>360133</td><td>UK Biobank</td><td>https://docs.google.com/spreadsheets/d/1kvPoupSzsSFBNSztMzl04xMoSC3Kcx3CrjVf4yBmESU/edit?ts=5b5f17db#gid=227859291</td><td>PHESANT Transformation:20002_0 | | CAT-MUL-BINARY-VAR 1291 | | Indicator name x135_0_0 | | Remove indicator var NAs: 53 | | Remove indicator var <0: 0 | | Removed 0 examples != 1291 but with missing value (<0) | | sample 360133/1008(361141) | |-Notes:Code for non-cancer illness. If the participant was uncertain of the type of illness they had had, then they described it to the interviewer (a trained nurse) who attempted to place it within the coding tree. If the illness could not be located in the coding tree then the interviewer entered a free-text description of it. These free-text descriptions were subsequently examined by a doctor and, where possible, matched to entries in the coding tree. Free-text descriptions which could not be matched with very high probability have been marked as unclassifiable.-Variable type:binary</td></tr>
<tr><td>binary.20002_1294.txt</td><td>1710174270056F5forCTG.txt.gz</td><td>0.1392</td><td>0.08291</td><td>1.678</td><td>0.09327</td><td>0.004834</td><td>0.001647</td><td>0.998</td><td>0.00951</td><td>0.01267</td><td>0.007251</td><td>Non-cancer illness code, self-reported: back problem</td><td>FALSE</td><td></td><td></td><td></td><td>361141</td><td>6339</td><td>354802</td><td>UK Biobank</td><td>https://docs.google.com/spreadsheets/d/1kvPoupSzsSFBNSztMzl04xMoSC3Kcx3CrjVf4yBmESU/edit?ts=5b5f17db#gid=227859291</td><td>PHESANT Transformation:20002_0 | | CAT-MUL-BINARY-VAR 1294 | | Indicator name x135_0_0 | | Remove indicator var NAs: 53 | | Remove indicator var <0: 0 | | Removed 0 examples != 1294 but with missing value (<0) | | sample 354802/6339(361141) | |-Notes:Code for non-cancer illness. If the participant was uncertain of the type of illness they had had, then they described it to the interviewer (a trained nurse) who attempted to place it within the coding tree. If the illness could not be located in the coding tree then the interviewer entered a free-text description of it. These free-text descriptions were subsequently examined by a doctor and, where possible, matched to entries in the coding tree. Free-text descriptions which could not be matched with very high probability have been marked as unclassifiable.-Variable type:binary</td></tr>
<tr><td>binary.20002_1309.txt</td><td>1710174270056F5forCTG.txt.gz</td><td>0.008408</td><td>0.05727</td><td>0.1468</td><td>0.8833</td><td>0.01404</td><td>0.002083</td><td>1.001</td><td>0.01137</td><td>-0.000242</td><td>0.008581</td><td>Non-cancer illness code, self-reported: osteoporosis</td><td>FALSE</td><td></td><td></td><td></td><td>361141</td><td>5736</td><td>355405</td><td>UK Biobank</td><td>https://docs.google.com/spreadsheets/d/1kvPoupSzsSFBNSztMzl04xMoSC3Kcx3CrjVf4yBmESU/edit?ts=5b5f17db#gid=227859291</td><td>PHESANT Transformation:20002_0 | | CAT-MUL-BINARY-VAR 1309 | | Indicator name x135_0_0 | | Remove indicator var NAs: 53 | | Remove indicator var <0: 0 | | Removed 0 examples != 1309 but with missing value (<0) | | sample 355405/5736(361141) | |-Notes:Code for non-cancer illness. If the participant was uncertain of the type of illness they had had, then they described it to the interviewer (a trained nurse) who attempted to place it within the coding tree. If the illness could not be located in the coding tree then the interviewer entered a free-text description of it. These free-text descriptions were subsequently examined by a doctor and, where possible, matched to entries in the coding tree. Free-text descriptions which could not be matched with very high probability have been marked as unclassifiable.-Variable type:binary</td></tr>
</table>

<table>
<tr><th></th><th></th><th></th><th></th><th></th><th></th><th></th><th></th><th></th><th></th><th></th><th></th><th></th><th></th><th></th><th></th><th></th><th></th><th></th><th></th><th></th><th></th><th></th></tr>
<tr><td>binary.20002_1311.txt</td><td>1710174270056F5forCTG.txt.gz</td><td>0.317</td><td>0.1468</td><td>2.159</td><td>0.03088</td><td>0.002605</td><td>0.001692</td><td>1.011</td><td>0.0102</td><td>0.01237</td><td>0.008207</td><td>Non-cancer illness code, self-reported: spine arthritis/spondylitis</td><td>FALSE</td><td></td><td></td><td></td><td>361141</td><td>3154</td><td>357987</td><td>UK Biobank</td><td>https://docs.google.com/spreadsheets/d/1kvPoupSzsSFBNSztMzl04xMoSC3Kcx3CrjVf4yBmESU/edit?ts=5b5f17db#gid=227859291</td><td>PHESANT Transformation:20002_0 | | CAT-MUL-BINARY-VAR 1311 | | Indicator name x135_0_0 | | Remove indicator var NAs: 53 | | Remove indicator var <0: 0 | | Removed 0 examples != 1311 but with missing value (<0) | | sample 357987/3154(361141) | | -Notes:Code for non-cancer illness. If the participant was uncertain of the type of illness they had had, then they described it to the interviewer (a trained nurse) who attempted to place it within the coding tree. If the illness could not be located in the coding tree then the interviewer entered a free-text description of it. These free-text descriptions were subsequently examined by a doctor and, where possible, matched to entries in the coding tree. Free-text descriptions which could not be matched with very high probability have been marked as unclassifiable.-Variable type:binary</td></tr>
<tr><td>binary.20002_1330.txt</td><td>1710174270056F5forCTG.txt.gz</td><td>0.06452</td><td>0.1006</td><td>0.6412</td><td>0.5214</td><td>0.002891</td><td>0.001506</td><td>0.992</td><td>0.009225</td><td>0.002339</td><td>0.007101</td><td>Non-cancer illness code, self-reported: iron deficiency anaemia</td><td>FALSE</td><td></td><td></td><td></td><td>361141</td><td>1988</td><td>359153</td><td>UK Biobank</td><td>https://docs.google.com/spreadsheets/d/1kvPoupSzsSFBNSztMzl04xMoSC3Kcx3CrjVf4yBmESU/edit?ts=5b5f17db#gid=227859291</td><td>PHESANT Transformation:20002_0 | | CAT-MUL-BINARY-VAR 1330 | | Indicator name x135_0_0 | | Remove indicator var NAs: 53 | | Remove indicator var <0: 0 | | Removed 0 examples != 1330 but with missing value (<0) | | sample 359153/1988(361141) | | -Notes:Code for non-cancer illness. If the participant was uncertain of the type of illness they had had, then they described it to the interviewer (a trained nurse) who attempted to place it within the coding tree. If the illness could not be located in the coding tree then the interviewer entered a free-text description of it. These free-text descriptions were subsequently examined by a doctor and, where possible, matched to entries in the coding tree. Free-text descriptions which could not be matched with very high probability have been marked as unclassifiable.-Variable type:binary</td></tr>
<tr><td>binary.20002_1331.txt</td><td>1710174270056F5forCTG.txt.gz</td><td>0.03907</td><td>0.1072</td><td>0.3644</td><td>0.7156</td><td>0.003165</td><td>0.001418</td><td>0.99</td><td>0.00918</td><td>-0.000149</td><td>0.007862</td><td>Non-cancer illness code, self-reported: pernicious anaemia</td><td>FALSE</td><td></td><td></td><td></td><td>361141</td><td>1109</td><td>360032</td><td>UK Biobank</td><td>https://docs.google.com/spreadsheets/d/1kvPoupSzsSFBNSztMzl04xMoSC3Kcx3CrjVf4yBmESU/edit?ts=5b5f17db#gid=227859291</td><td>PHESANT Transformation:20002_0 | | CAT-MUL-BINARY-VAR 1331 | | Indicator name x135_0_0 | | Remove indicator var NAs: 53 | | Remove indicator var <0: 0 | | Removed 0 examples != 1331 but with missing value (<0) | | sample 360032/1109(361141) | | -Notes:Code for non-cancer illness. If the participant was uncertain of the type of illness they had had, then they described it to the interviewer (a trained nurse) who attempted to place it within the coding tree. If the illness could not be located in the coding tree then the interviewer entered a free-text description of it. These free-text descriptions were subsequently examined by a doctor and, where possible, matched to entries in the coding tree. Free-text descriptions which could not be matched with very high probability have been marked as unclassifiable.-Variable type:binary</td></tr>
<tr><td>binary.20002_1371.txt</td><td>1710174270056F5forCTG.txt.gz</td><td>-0.006814</td><td>0.08823</td><td>-0.07723</td><td>0.9384</td><td>0.003976</td><td>0.001536</td><td>0.996</td><td>0.0107</td><td>3.74E-05</td><td>0.007728</td><td>Non-cancer illness code, self-reported: sarcoidosis</td><td>FALSE</td><td></td><td></td><td></td><td>361141</td><td>705</td><td>360436</td><td>UK Biobank</td><td>https://docs.google.com/spreadsheets/d/1kvPoupSzsSFBNSztMzl04xMoSC3Kcx3CrjVf4yBmESU/edit?ts=5b5f17db#gid=227859291</td><td>PHESANT Transformation:20002_0 | | CAT-MUL-BINARY-VAR 1371 | | Indicator name x135_0_0 | | Remove indicator var NAs: 53 | | Remove indicator var <0: 0 | | Removed 0 examples != 1371 but with missing value (<0) | | sample 360436/705(361141) | | -Notes:Code for non-cancer illness. If the participant was uncertain of the type of illness they had had, then they described it to the interviewer (a trained nurse) who attempted to place it within the coding tree. If the illness could not be located in the coding tree then the interviewer entered a free-text description of it. These free-text descriptions were subsequently examined by a doctor and, where possible, matched to entries in the coding tree. Free-text descriptions which could not be matched with very high probability have been marked as unclassifiable.-Variable type:binary</td></tr>
<tr><td>binary.20002_1377.txt</td><td>1710174270056F5forCTG.txt.gz</td><td>0.1275</td><td>0.09165</td><td>1.391</td><td>0.1643</td><td>0.003545</td><td>0.001412</td><td>0.988</td><td>0.009108</td><td>-0.002765</td><td>0.007277</td><td>Non-cancer illness code, self-reported: polymyalgia rheumatica</td><td>FALSE</td><td></td><td></td><td></td><td>361141</td><td>753</td><td>360388</td><td>UK Biobank</td><td>https://docs.google.com/spreadsheets/d/1kvPoupSzsSFBNSztMzl04xMoSC3Kcx3CrjVf4yBmESU/edit?ts=5b5f17db#gid=227859291</td><td>PHESANT Transformation:20002_0 | | CAT-MUL-BINARY-VAR 1377 | | Indicator name x135_0_0 | | Remove indicator var NAs: 53 | | Remove indicator var <0: 0 | | Removed 0 examples != 1377 but with missing value (<0) | | sample 360388/753(361141) | | -Notes:Code for non-cancer illness. If the participant was uncertain of the type of illness they had had, then they described it to the interviewer (a trained nurse) who attempted to place it within the coding tree. If the illness could not be located in the coding tree then the interviewer entered a free-text description of it. These free-text descriptions were subsequently examined by a doctor and, where possible, matched to entries in the coding tree. Free-text descriptions which could not be matched with very high probability have been marked as unclassifiable.-Variable type:binary</td></tr>
<tr><td>binary.20002_1387.txt</td><td>1710174270056F5forCTG.txt.gz</td><td>-0.09439</td><td>0.03997</td><td>-2.362</td><td>0.01819</td><td>0.02323</td><td>0.00284</td><td>1.006</td><td>0.01283</td><td>0.008281</td><td>0.007506</td><td>Non-cancer illness code, self-reported: hayfever/allergic rhinitis</td><td>FALSE</td><td></td><td></td><td></td><td>361141</td><td>20667</td><td>340474</td><td>UK Biobank</td><td>https://docs.google.com/spreadsheets/d/1kvPoupSzsSFBNSztMzl04xMoSC3Kcx3CrjVf4yBmESU/edit?ts=5b5f17db#gid=227859291</td><td>PHESANT Transformation:20002_0 | | CAT-MUL-BINARY-VAR 1387 | | Indicator name x135_0_0 | | Remove indicator var NAs: 53 | | Remove indicator var <0: 0 | | Removed 0 examples != 1387 but with missing value (<0) | | sample 340474/20667(361141) | | -Notes:Code for non-cancer illness. If the participant was uncertain of the type of illness they had had, then they described it to the interviewer (a trained nurse) who attempted to place it within the coding tree. If the illness could not be located in the coding tree then the interviewer entered a free-text description of it. These free-text descriptions were subsequently examined by a doctor and, where possible, matched to entries in the coding tree. Free-text descriptions which could not be matched with very high probability have been marked as unclassifiable.-Variable type:binary</td></tr>
<tr><td>binary.20002_1398.txt</td><td>1710174270056F5forCTG.txt.gz</td><td>0.2749</td><td>0.1138</td><td>2.415</td><td>0.01576</td><td>0.003404</td><td>0.001546</td><td>0.999</td><td>0.009845</td><td>-0.001256</td><td>0.007299</td><td>Non-cancer illness code, self-reported: pneumonia</td><td>FALSE</td><td></td><td></td><td></td><td>361141</td><td>5182</td><td>355959</td><td>UK Biobank</td><td>https://docs.google.com/spreadsheets/d/1kvPoupSzsSFBNSztMzl04xMoSC3Kcx3CrjVf4yBmESU/edit?ts=5b5f17db#gid=227859291</td><td>PHESANT Transformation:20002_0 | | CAT-MUL-BINARY-VAR 1398 | | Indicator name x135_0_0 | | Remove indicator var NAs: 53 | | Remove indicator var <0: 0 | | Removed 0 examples != 1398 but with missing value (<0) | | sample 355959/5182(361141) | | -Notes:Code for non-cancer illness. If the participant was uncertain of the type of illness they had had, then they described it to the interviewer (a trained nurse) who attempted to place it within the coding tree. If the illness could not be located in the coding tree then the interviewer entered a free-text description of it. These free-text descriptions were subsequently examined by a doctor and, where possible, matched to entries in the coding tree. Free-text descriptions which could not be matched with very high probability have been marked as unclassifiable.-Variable type:binary</td></tr>
<tr><td>binary.20002_1408.txt</td><td>1710174270056F5forCTG.txt.gz</td><td>0.08833</td><td>0.09185</td><td>0.9616</td><td>0.3362</td><td>0.004003</td><td>0.001549</td><td>0.983</td><td>0.009371</td><td>0.003351</td><td>0.008143</td><td>Non-cancer illness code, self-reported: alcohol dependency</td><td>FALSE</td><td></td><td></td><td></td><td>361141</td><td>561</td><td>360580</td><td>UK Biobank</td><td>https://docs.google.com/spreadsheets/d/1kvPoupSzsSFBNSztMzl04xMoSC3Kcx3CrjVf4yBmESU/edit?ts=5b5f17db#gid=227859291</td><td>PHESANT Transformation:20002_0 | | CAT-MUL-BINARY-VAR 1408 | | Indicator name x135_0_0 | | Remove indicator var NAs: 53 | | Remove indicator var <0: 0 | | Removed 0 examples != 1408 but with missing value (<0) | | sample 360580/561(361141) | | -Notes:Code for non-cancer illness. If the participant was uncertain of the type of illness they had had, then they described it to the interviewer (a trained nurse) who attempted to place it within the coding tree. If the illness could not be located in the coding tree then the interviewer entered a free-text description of it. These free-text descriptions were subsequently examined by a doctor and, where possible, matched to entries in the coding tree. Free-text descriptions which could not be matched with very high probability have been marked as unclassifiable.-Variable type:binary</td></tr>
<tr><td>binary.20002_1417.txt</td><td>1710174270056F5forCTG.txt.gz</td><td>-0.04127</td><td>0.08691</td><td>-0.4749</td><td>0.6349</td><td>0.004526</td><td>0.001905</td><td>1.01</td><td>0.01024</td><td>-0.004104</td><td>0.007867</td><td>Non-cancer illness code, self-reported: nasal polyps</td><td>FALSE</td><td></td><td></td><td></td><td>361141</td><td>1587</td><td>359554</td><td>UK Biobank</td><td>https://docs.google.com/spreadsheets/d/1kvPoupSzsSFBNSztMzl04xMoSC3Kcx3CrjVf4yBmESU/edit?ts=5b5f17db#gid=227859291</td><td>PHESANT Transformation:20002_0 | | CAT-MUL-BINARY-VAR 1417 | | Indicator name x135_0_0 | | Remove indicator var NAs: 53 | | Remove indicator var <0: 0 | | Removed 0 examples != 1417 but with missing value (<0) | | sample 359554/1587(361141) | | -Notes:Code for non-cancer illness. If the participant was uncertain of the type of illness they had had, then they described it to the interviewer (a trained nurse) who attempted to place it within the coding tree. If the illness could not be located in the coding tree then the interviewer entered a free-text description of it. These free-text descriptions were subsequently examined by a doctor and, where possible, matched to entries in the coding tree. Free-text descriptions which could not be matched with very high probability have been marked as unclassifiable.-Variable type:binary</td></tr>
<tr><td>binary.20002_1439.txt</td><td>1710174270056F5forCTG.txt.gz</td><td>0.0473</td><td>0.08949</td><td>0.5285</td><td>0.5971</td><td>0.004343</td><td>0.001653</td><td>0.98</td><td>0.01054</td><td>-0.003746</td><td>0.008988</td><td>Non-cancer illness code, self-reported: hiv/aids</td><td>FALSE</td><td></td><td></td><td></td><td>361141</td><td>285</td><td>360856</td><td>UK Biobank</td><td>https://docs.google.com/spreadsheets/d/1kvPoupSzsSFBNSztMzl04xMoSC3Kcx3CrjVf4yBmESU/edit?ts=5b5f17db#gid=227859291</td><td>PHESANT Transformation:20002_0 | | CAT-MUL-BINARY-VAR 1439 | | Indicator name x135_0_0 | | Remove indicator var NAs: 53 | | Remove indicator var <0: 0 | | Removed 0 examples != 1439 but with missing value (<0) | | sample 360856/285(361141) | | -Notes:Code for non-cancer illness. If the participant was uncertain of the type of illness they had had, then they described it to the interviewer (a trained nurse) who attempted to place it within the coding tree. If the illness could not be located in the coding tree then the interviewer entered a free-text description of it. These free-text descriptions were subsequently examined by a doctor and, where possible, matched to entries in the coding tree. Free-text descriptions which could not be matched with very high probability have been marked as unclassifiable.-Variable type:binary</td></tr>
</table>

binary.20002_1442.txt	1710174270056F5forCTG.txt.gz	0.07515	0.1133	0.6632	0.5072	0.002486	0.001288	0.995	0.007984	0.003614	0.008144	Non-cancer illness code, self-reported: helicobacter pylori	FALSE				361141	1005	360136	UK Biobank	https://docs.google.com/spreadsheets/d/1kvPoupSzsSFBNSztMzl04xMoSC3Kcx3CrjVf4yBmESU/edit?ts=5b5f17db#gid=227859291	PHESANT Transformation:20002_0 | | CAT-MUL-BINARY-VAR 1442 | | Indicator name x135_0_0 | | Remove indicator var NAs: 53 | | Remove indicator var <0: 0 | | Removed 0 examples != 1442 but with missing value (<0) | | sample 360136/1005(361141) | |-Notes:Code for non-cancer illness. If the participant was uncertain of the type of illness they had had, then they described it to the interviewer (a trained nurse) who attempted to place it within the coding tree. If the illness could not be located in the coding tree then the interviewer entered a free-text description of it. These free-text descriptions were subsequently examined by a doctor and, where possible, matched to entries in the coding tree. Free-text descriptions which could not be matched with very high probability have been marked as unclassifiable.-Variable type:binary
binary.20002_1452.txt	1710174270056F5forCTG.txt.gz	-0.1194	0.07126	-1.676	0.09376	0.00742	0.002705	1.026	0.01094	0.0152	0.008007	Non-cancer illness code, self-reported: eczema/dermatitis	FALSE				361141	9321	351820	UK Biobank	https://docs.google.com/spreadsheets/d/1kvPoupSzsSFBNSztMzl04xMoSC3Kcx3CrjVf4yBmESU/edit?ts=5b5f17db#gid=227859291	PHESANT Transformation:20002_0 | | CAT-MUL-BINARY-VAR 1452 | | Indicator name x135_0_0 | | Remove indicator var NAs: 53 | | Remove indicator var <0: 0 | | Removed 0 examples != 1452 but with missing value (<0) | | sample 351820/9321(361141) | |-Notes:Code for non-cancer illness. If the participant was uncertain of the type of illness they had had, then they described it to the interviewer (a trained nurse) who attempted to place it within the coding tree. If the illness could not be located in the coding tree then the interviewer entered a free-text description of it. These free-text descriptions were subsequently examined by a doctor and, where possible, matched to entries in the coding tree. Free-text descriptions which could not be matched with very high probability have been marked as unclassifiable.-Variable type:binary
binary.20002_1453.txt	1710174270056F5forCTG.txt.gz	0.05357	0.07073	0.7573	0.4489	0.008433	0.001999	1.006	0.01184	-0.004396	0.008344	Non-cancer illness code, self-reported: psoriasis	FALSE				361141	4192	356949	UK Biobank	https://docs.google.com/spreadsheets/d/1kvPoupSzsSFBNSztMzl04xMoSC3Kcx3CrjVf4yBmESU/edit?ts=5b5f17db#gid=227859291	PHESANT Transformation:20002_0 | | CAT-MUL-BINARY-VAR 1453 | | Indicator name x135_0_0 | | Remove indicator var NAs: 53 | | Remove indicator var <0: 0 | | Removed 0 examples != 1453 but with missing value (<0) | | sample 356949/4192(361141) | |-Notes:Code for non-cancer illness. If the participant was uncertain of the type of illness they had had, then they described it to the interviewer (a trained nurse) who attempted to place it within the coding tree. If the illness could not be located in the coding tree then the interviewer entered a free-text description of it. These free-text descriptions were subsequently examined by a doctor and, where possible, matched to entries in the coding tree. Free-text descriptions which could not be matched with very high probability have been marked as unclassifiable.-Variable type:binary
binary.20002_1456.txt	1710174270056F5forCTG.txt.gz	0.03172	0.09193	0.345	0.7301	0.004436	0.001634	1.021	0.009755	0.001609	0.007755	Non-cancer illness code, self-reported: malabsorption/coeliac disease	FALSE				361141	1587	359554	UK Biobank	https://docs.google.com/spreadsheets/d/1kvPoupSzsSFBNSztMzl04xMoSC3Kcx3CrjVf4yBmESU/edit?ts=5b5f17db#gid=227859291	PHESANT Transformation:20002_0 | | CAT-MUL-BINARY-VAR 1456 | | Indicator name x135_0_0 | | Remove indicator var NAs: 53 | | Remove indicator var <0: 0 | | Removed 0 examples != 1456 but with missing value (<0) | | sample 359554/1587(361141) | |-Notes:Code for non-cancer illness. If the participant was uncertain of the type of illness they had had, then they described it to the interviewer (a trained nurse) who attempted to place it within the coding tree. If the illness could not be located in the coding tree then the interviewer entered a free-text description of it. These free-text descriptions were subsequently examined by a doctor and, where possible, matched to entries in the coding tree. Free-text descriptions which could not be matched with very high probability have been marked as unclassifiable.-Variable type:binary
binary.20002_1458.txt	1710174270056F5forCTG.txt.gz	0.1653	0.05186	3.187	0.001438	0.0144	0.002096	0.985	0.01047	0.003265	0.008435	Non-cancer illness code, self-reported: diverticular disease/diverticulitis	FALSE				361141	4124	357017	UK Biobank	https://docs.google.com/spreadsheets/d/1kvPoupSzsSFBNSztMzl04xMoSC3Kcx3CrjVf4yBmESU/edit?ts=5b5f17db#gid=227859291	PHESANT Transformation:20002_0 | | CAT-MUL-BINARY-VAR 1458 | | Indicator name x135_0_0 | | Remove indicator var NAs: 53 | | Remove indicator var <0: 0 | | Removed 0 examples != 1458 but with missing value (<0) | | sample 357017/4124(361141) | |-Notes:Code for non-cancer illness. If the participant was uncertain of the type of illness they had had, then they described it to the interviewer (a trained nurse) who attempted to place it within the coding tree. If the illness could not be located in the coding tree then the interviewer entered a free-text description of it. These free-text descriptions were subsequently examined by a doctor and, where possible, matched to entries in the coding tree. Free-text descriptions which could not be matched with very high probability have been marked as unclassifiable.-Variable type:binary
binary.20002_1462.txt	1710174270056F5forCTG.txt.gz	0.0461	0.08387	0.5497	0.5825	0.004586	0.001735	1.007	0.01048	0.009582	0.007806	Non-cancer illness code, self-reported: crohns disease	FALSE				361141	1096	360045	UK Biobank	https://docs.google.com/spreadsheets/d/1kvPoupSzsSFBNSztMzl04xMoSC3Kcx3CrjVf4yBmESU/edit?ts=5b5f17db#gid=227859291	PHESANT Transformation:20002_0 | | CAT-MUL-BINARY-VAR 1462 | | Indicator name x135_0_0 | | Remove indicator var NAs: 53 | | Remove indicator var <0: 0 | | Removed 0 examples != 1462 but with missing value (<0) | | sample 360045/1096(361141) | |-Notes:Code for non-cancer illness. If the participant was uncertain of the type of illness they had had, then they described it to the interviewer (a trained nurse) who attempted to place it within the coding tree. If the illness could not be located in the coding tree then the interviewer entered a free-text description of it. These free-text descriptions were subsequently examined by a doctor and, where possible, matched to entries in the coding tree. Free-text descriptions which could not be matched with very high probability have been marked as unclassifiable.-Variable type:binary
binary.20002_1463.txt	1710174270056F5forCTG.txt.gz	-0.07716	0.08803	-0.8765	0.3808	0.005147	0.001801	1.017	0.01115	0.01676	0.007569	Non-cancer illness code, self-reported: ulcerative colitis	FALSE				361141	1916	359225	UK Biobank	https://docs.google.com/spreadsheets/d/1kvPoupSzsSFBNSztMzl04xMoSC3Kcx3CrjVf4yBmESU/edit?ts=5b5f17db#gid=227859291	PHESANT Transformation:20002_0 | | CAT-MUL-BINARY-VAR 1463 | | Indicator name x135_0_0 | | Remove indicator var NAs: 53 | | Remove indicator var <0: 0 | | Removed 0 examples != 1463 but with missing value (<0) | | sample 359225/1916(361141) | |-Notes:Code for non-cancer illness. If the participant was uncertain of the type of illness they had had, then they described it to the interviewer (a trained nurse) who attempted to place it within the coding tree. If the illness could not be located in the coding tree then the interviewer entered a free-text description of it. These free-text descriptions were subsequently examined by a doctor and, where possible, matched to entries in the coding tree. Free-text descriptions which could not be matched with very high probability have been marked as unclassifiable.-Variable type:binary
binary.20002_1464.txt	1710174270056F5forCTG.txt.gz	0.1886	0.09396	2.008	0.04469	0.005064	0.001483	1.002	0.00848	0.01561	0.007933	Non-cancer illness code, self-reported: rheumatoid arthritis	FALSE				361141	4017	357124	UK Biobank	https://docs.google.com/spreadsheets/d/1kvPoupSzsSFBNSztMzl04xMoSC3Kcx3CrjVf4yBmESU/edit?ts=5b5f17db#gid=227859291	PHESANT Transformation:20002_0 | | CAT-MUL-BINARY-VAR 1464 | | Indicator name x135_0_0 | | Remove indicator var NAs: 53 | | Remove indicator var <0: 0 | | Removed 0 examples != 1464 but with missing value (<0) | | sample 357124/4017(361141) | |-Notes:Code for non-cancer illness. If the participant was uncertain of the type of illness they had had, then they described it to the interviewer (a trained nurse) who attempted to place it within the coding tree. If the illness could not be located in the coding tree then the interviewer entered a free-text description of it. These free-text descriptions were subsequently examined by a doctor and, where possible, matched to entries in the coding tree. Free-text descriptions which could not be matched with very high probability have been marked as unclassifiable.-Variable type:binary
binary.20002_1465.txt	1710174270056F5forCTG.txt.gz	0.3388	0.05052	6.706	2.00E-11	0.01895	0.001853	1.021	0.01067	0.001622	0.008951	Non-cancer illness code, self-reported: osteoarthritis	TRUE	Skeletal	Physical health	Osteoarthritis	361141	30046	331095	UK Biobank	https://docs.google.com/spreadsheets/d/1kvPoupSzsSFBNSztMzl04xMoSC3Kcx3CrjVf4yBmESU/edit?ts=5b5f17db#gid=227859291	PHESANT Transformation:20002_0 | | CAT-MUL-BINARY-VAR 1465 | | Indicator name x135_0_0 | | Remove indicator var NAs: 53 | | Remove indicator var <0: 0 | | Removed 0 examples != 1465 but with missing value (<0) | | sample 331095/30046(361141) | |-Notes:Code for non-cancer illness. If the participant was uncertain of the type of illness they had had, then they described it to the interviewer (a trained nurse) who attempted to place it within the coding tree. If the illness could not be located in the coding tree then the interviewer entered a free-text description of it. These free-text descriptions were subsequently examined by a doctor and, where possible, matched to entries in the coding tree. Free-text descriptions which could not be matched with very high probability have been marked as unclassifiable.-Variable type:binary
binary.20002_1466.txt	1710174270056F5forCTG.txt.gz	0.0732	0.04229	1.731	0.08348	0.02559	0.009122	0.984	0.04139	0.001115	0.008632	Non-cancer illness code, self-reported: gout	FALSE				361141	5174	355967	UK Biobank	https://docs.google.com/spreadsheets/d/1kvPoupSzsSFBNSztMzl04xMoSC3Kcx3CrjVf4yBmESU/edit?ts=5b5f17db#gid=227859291	PHESANT Transformation:20002_0 | | CAT-MUL-BINARY-VAR 1466 | | Indicator name x135_0_0 | | Remove indicator var NAs: 53 | | Remove indicator var <0: 0 | | Removed 0 examples != 1466 but with missing value (<0) | | sample 355967/5174(361141) | |-Notes:Code for non-cancer illness. If the participant was uncertain of the type of illness they had had, then they described it to the interviewer (a trained nurse) who attempted to place it within the coding tree. If the illness could not be located in the coding tree then the interviewer entered a free-text description of it. These free-text descriptions were subsequently examined by a doctor and, where possible, matched to entries in the coding tree. Free-text descriptions which could not be matched with very high probability have been marked as unclassifiable.-Variable type:binary

<table>
<tr><th></th><th></th><th></th><th></th><th></th><th></th><th></th><th></th><th></th><th></th><th></th><th></th><th></th><th></th><th></th><th></th><th></th><th></th><th></th><th></th><th></th><th></th></tr>
<tr><td>binary.20002_1471.txt</td><td>1710174270056F5forCTG.txt.gz</td><td>-0.07529</td><td>0.09623</td><td>-0.7824</td><td>0.434</td><td>0.003963</td><td>0.002051</td><td>1.032</td><td>0.01337</td><td>0.01008</td><td>0.008046</td><td>Non-cancer illness code, self-reported: atrial fibrillation</td><td>FALSE</td><td></td><td></td><td></td><td>361141</td><td>2828</td><td>358313</td><td>UK Biobank</td><td>https://docs.google.com/spreadsheets/d/1kvPoupSzsSFBNSztMzl04xMoSC3Kcx3CrjVf4yBmESU/edit?ts=5b5f17db#gid=227859291</td><td>PHESANT Transformation:20002_0 | | CAT-MUL-BINARY-VAR 1471 | | Indicator name x135_0_0 | | Remove indicator var NAs: 53 | | Remove indicator var <0: 0 | | Removed 0 examples != 1471 but with missing value (<0) | | sample 358313/2828(361141) | |-Notes:Code for non-cancer illness. If the participant was uncertain of the type of illness they had had, then they described it to the interviewer (a trained nurse) who attempted to place it within the coding tree. If the illness could not be located in the coding tree then the interviewer entered a free-text description of it. These free-text descriptions were subsequently examined by a doctor and, where possible, matched to entries in the coding tree. Free-text descriptions which could not be matched with very high probability have been marked as unclassifiable.-Variable type:binary</td></tr>
<tr><td>binary.20002_1473.txt</td><td>1710174270056F5forCTG.txt.gz</td><td>0.1737</td><td>0.04792</td><td>3.626</td><td>0.000288</td><td>0.03679</td><td>0.009275</td><td>1.115</td><td>0.06556</td><td>-0.004104</td><td>0.009386</td><td>Non-cancer illness code, self-reported: high cholesterol</td><td>FALSE</td><td></td><td></td><td></td><td>361141</td><td>43957</td><td>317184</td><td>UK Biobank</td><td>https://docs.google.com/spreadsheets/d/1kvPoupSzsSFBNSztMzl04xMoSC3Kcx3CrjVf4yBmESU/edit?ts=5b5f17db#gid=227859291</td><td>PHESANT Transformation:20002_0 | | CAT-MUL-BINARY-VAR 1473 | | Indicator name x135_0_0 | | Remove indicator var NAs: 53 | | Remove indicator var <0: 0 | | Removed 0 examples != 1473 but with missing value (<0) | | sample 317184/43957(361141) | |-Notes:Code for non-cancer illness. If the participant was uncertain of the type of illness they had had, then they described it to the interviewer (a trained nurse) who attempted to place it within the coding tree. If the illness could not be located in the coding tree then the interviewer entered a free-text description of it. These free-text descriptions were subsequently examined by a doctor and, where possible, matched to entries in the coding tree. Free-text descriptions which could not be matched with very high probability have been marked as unclassifiable.-Variable type:binary</td></tr>
<tr><td>binary.20002_1474.txt</td><td>1710174270056F5forCTG.txt.gz</td><td>0.4021</td><td>0.07743</td><td>5.192</td><td>2.08E-07</td><td>0.008284</td><td>0.001497</td><td>1.031</td><td>0.008678</td><td>-0.006947</td><td>0.008214</td><td>Non-cancer illness code, self-reported: hiatus hernia</td><td>FALSE</td><td>Other (physical health)</td><td></td><td></td><td>361141</td><td>8340</td><td>352801</td><td>UK Biobank</td><td>https://docs.google.com/spreadsheets/d/1kvPoupSzsSFBNSztMzl04xMoSC3Kcx3CrjVf4yBmESU/edit?ts=5b5f17db#gid=227859291</td><td>PHESANT Transformation:20002_0 | | CAT-MUL-BINARY-VAR 1474 | | Indicator name x135_0_0 | | Remove indicator var NAs: 53 | | Remove indicator var <0: 0 | | Removed 0 examples != 1474 but with missing value (<0) | | sample 352801/8340(361141) | |-Notes:Code for non-cancer illness. If the participant was uncertain of the type of illness they had had, then they described it to the interviewer (a trained nurse) who attempted to place it within the coding tree. If the illness could not be located in the coding tree then the interviewer entered a free-text description of it. These free-text descriptions were subsequently examined by a doctor and, where possible, matched to entries in the coding tree. Free-text descriptions which could not be matched with very high probability have been marked as unclassifiable.-Variable type:binary</td></tr>
<tr><td>binary.20002_1476.txt</td><td>1710174270056F5forCTG.txt.gz</td><td>0.3429</td><td>0.1693</td><td>2.026</td><td>0.04274</td><td>0.002131</td><td>0.001521</td><td>1.01</td><td>0.009665</td><td>0.001659</td><td>0.007656</td><td>Non-cancer illness code, self-reported: sciatica</td><td>FALSE</td><td></td><td></td><td></td><td>361141</td><td>3400</td><td>357741</td><td>UK Biobank</td><td>https://docs.google.com/spreadsheets/d/1kvPoupSzsSFBNSztMzl04xMoSC3Kcx3CrjVf4yBmESU/edit?ts=5b5f17db#gid=227859291</td><td>PHESANT Transformation:20002_0 | | CAT-MUL-BINARY-VAR 1476 | | Indicator name x135_0_0 | | Remove indicator var NAs: 53 | | Remove indicator var <0: 0 | | Removed 0 examples != 1476 but with missing value (<0) | | sample 357741/3400(361141) | |-Notes:Code for non-cancer illness. If the participant was uncertain of the type of illness they had had, then they described it to the interviewer (a trained nurse) who attempted to place it within the coding tree. If the illness could not be located in the coding tree then the interviewer entered a free-text description of it. These free-text descriptions were subsequently examined by a doctor and, where possible, matched to entries in the coding tree. Free-text descriptions which could not be matched with very high probability have been marked as unclassifiable.-Variable type:binary</td></tr>
<tr><td>binary.20002_1478.txt</td><td>1710174270056F5forCTG.txt.gz</td><td>0.3167</td><td>0.1252</td><td>2.528</td><td>0.01146</td><td>0.002594</td><td>0.001519</td><td>1.002</td><td>0.00936</td><td>0.00397</td><td>0.008164</td><td>Non-cancer illness code, self-reported: cervical spondylosis</td><td>FALSE</td><td></td><td></td><td></td><td>361141</td><td>2510</td><td>358631</td><td>UK Biobank</td><td>https://docs.google.com/spreadsheets/d/1kvPoupSzsSFBNSztMzl04xMoSC3Kcx3CrjVf4yBmESU/edit?ts=5b5f17db#gid=227859291</td><td>PHESANT Transformation:20002_0 | | CAT-MUL-BINARY-VAR 1478 | | Indicator name x135_0_0 | | Remove indicator var NAs: 53 | | Remove indicator var <0: 0 | | Removed 0 examples != 1478 but with missing value (<0) | | sample 358631/2510(361141) | |-Notes:Code for non-cancer illness. If the participant was uncertain of the type of illness they had had, then they described it to the interviewer (a trained nurse) who attempted to place it within the coding tree. If the illness could not be located in the coding tree then the interviewer entered a free-text description of it. These free-text descriptions were subsequently examined by a doctor and, where possible, matched to entries in the coding tree. Free-text descriptions which could not be matched with very high probability have been marked as unclassifiable.-Variable type:binary</td></tr>
<tr><td>binary.20002_1482.txt</td><td>1710174270056F5forCTG.txt.gz</td><td>0.1654</td><td>0.08716</td><td>1.898</td><td>0.05774</td><td>0.00503</td><td>0.001566</td><td>0.989</td><td>0.01016</td><td>0.001305</td><td>0.007622</td><td>Non-cancer illness code, self-reported: chronic fatigue syndrome</td><td>FALSE</td><td></td><td></td><td></td><td>361141</td><td>1659</td><td>359482</td><td>UK Biobank</td><td>https://docs.google.com/spreadsheets/d/1kvPoupSzsSFBNSztMzl04xMoSC3Kcx3CrjVf4yBmESU/edit?ts=5b5f17db#gid=227859291</td><td>PHESANT Transformation:20002_0 | | CAT-MUL-BINARY-VAR 1482 | | Indicator name x135_0_0 | | Remove indicator var NAs: 53 | | Remove indicator var <0: 0 | | Removed 0 examples != 1482 but with missing value (<0) | | sample 359482/1659(361141) | |-Notes:Code for non-cancer illness. If the participant was uncertain of the type of illness they had had, then they described it to the interviewer (a trained nurse) who attempted to place it within the coding tree. If the illness could not be located in the coding tree then the interviewer entered a free-text description of it. These free-text descriptions were subsequently examined by a doctor and, where possible, matched to entries in the coding tree. Free-text descriptions which could not be matched with very high probability have been marked as unclassifiable.-Variable type:binary</td></tr>
<tr><td>binary.20002_1494.txt</td><td>1710174270056F5forCTG.txt.gz</td><td>0.09974</td><td>0.0966</td><td>1.033</td><td>0.3018</td><td>0.003173</td><td>0.001459</td><td>1.01</td><td>0.009091</td><td>-0.01353</td><td>0.007432</td><td>Non-cancer illness code, self-reported: varicose veins</td><td>FALSE</td><td></td><td></td><td></td><td>361141</td><td>1299</td><td>359842</td><td>UK Biobank</td><td>https://docs.google.com/spreadsheets/d/1kvPoupSzsSFBNSztMzl04xMoSC3Kcx3CrjVf4yBmESU/edit?ts=5b5f17db#gid=227859291</td><td>PHESANT Transformation:20002_0 | | CAT-MUL-BINARY-VAR 1494 | | Indicator name x135_0_0 | | Remove indicator var NAs: 53 | | Remove indicator var <0: 0 | | Removed 0 examples != 1494 but with missing value (<0) | | sample 359842/1299(361141) | |-Notes:Code for non-cancer illness. If the participant was uncertain of the type of illness they had had, then they described it to the interviewer (a trained nurse) who attempted to place it within the coding tree. If the illness could not be located in the coding tree then the interviewer entered a free-text description of it. These free-text descriptions were subsequently examined by a doctor and, where possible, matched to entries in the coding tree. Free-text descriptions which could not be matched with very high probability have been marked as unclassifiable.-Variable type:binary</td></tr>
<tr><td>binary.20002_99999.txt</td><td>1710174270056F5forCTG.txt.gz</td><td>0.5088</td><td>0.4018</td><td>1.266</td><td>0.2054</td><td>0.001025</td><td>0.001481</td><td>1.009</td><td>0.008886</td><td>-0.001003</td><td>0.007073</td><td>Non-cancer illness code, self-reported: unclassifiable</td><td>FALSE</td><td></td><td></td><td></td><td>361141</td><td>14597</td><td>346544</td><td>UK Biobank</td><td>https://docs.google.com/spreadsheets/d/1kvPoupSzsSFBNSztMzl04xMoSC3Kcx3CrjVf4yBmESU/edit?ts=5b5f17db#gid=227859291</td><td>PHESANT Transformation:20002_0 | | CAT-MUL-BINARY-VAR 99999 | | Indicator name x135_0_0 | | Remove indicator var NAs: 53 | | Remove indicator var <0: 0 | | Removed 0 examples != 99999 but with missing value (<0) | | sample 346544/14597(361141) | |-Notes:Code for non-cancer illness. If the participant was uncertain of the type of illness they had had, then they described it to the interviewer (a trained nurse) who attempted to place it within the coding tree. If the illness could not be located in the coding tree then the interviewer entered a free-text description of it. These free-text descriptions were subsequently examined by a doctor and, where possible, matched to entries in the coding tree. Free-text descriptions which could not be matched with very high probability have been marked as unclassifiable.-Variable type:binary</td></tr>
<tr><td>binary.20003_1140851812.txt</td><td>1710174270056F5forCTG.txt.gz</td><td>0.2426</td><td>0.108</td><td>2.246</td><td>0.02468</td><td>0.003862</td><td>0.001555</td><td>0.991</td><td>0.009027</td><td>0.001768</td><td>0.008142</td><td>Treatment/medication code: gtn 400micrograms spray</td><td>FALSE</td><td></td><td></td><td></td><td>361141</td><td>849</td><td>360292</td><td>UK Biobank</td><td>https://docs.google.com/spreadsheets/d/1kvPoupSzsSFBNSztMzl04xMoSC3Kcx3CrjVf4yBmESU/edit?ts=5b5f17db#gid=227859291</td><td>PHESANT Transformation:20003_0 | | CAT-MUL-BINARY-VAR 1140851812 | | Indicator name x137_0_0 | | Remove indicator var NAs: 53 | | Remove indicator var <0: 0 | | Removed 0 examples != 1140851812 but with missing value (<0) | | sample 360292/849(361141) | |-Notes:Code for treatment Negative codes indicate free-text entry.-Variable type:binary</td></tr>
<tr><td>binary.20003_1140860696.txt</td><td>1710174270056F5forCTG.txt.gz</td><td>0.1381</td><td>0.05691</td><td>2.426</td><td>0.01527</td><td>0.01261</td><td>0.001846</td><td>1.017</td><td>0.0111</td><td>-0.01384</td><td>0.007912</td><td>Treatment/medication code: lisinopril</td><td>FALSE</td><td></td><td></td><td></td><td>361141</td><td>9971</td><td>351170</td><td>UK Biobank</td><td>https://docs.google.com/spreadsheets/d/1kvPoupSzsSFBNSztMzl04xMoSC3Kcx3CrjVf4yBmESU/edit?ts=5b5f17db#gid=227859291</td><td>PHESANT Transformation:20003_0 | | CAT-MUL-BINARY-VAR 1140860696 | | Indicator name x137_0_0 | | Remove indicator var NAs: 53 | | Remove indicator var <0: 0 | | Removed 0 examples != 1140860696 but with missing value (<0) | | sample 351170/9971(361141) | |-Notes:Code for treatment Negative codes indicate free-text entry.-Variable type:binary</td></tr>
<tr><td>binary.20003_1140860806.txt</td><td>1710174270056F5forCTG.txt.gz</td><td>0.121</td><td>0.04993</td><td>2.423</td><td>0.01538</td><td>0.01553</td><td>0.002073</td><td>1.028</td><td>0.01089</td><td>0.01249</td><td>0.00805</td><td>Treatment/medication code: ramipril</td><td>FALSE</td><td></td><td></td><td></td><td>361141</td><td>16869</td><td>344272</td><td>UK Biobank</td><td>https://docs.google.com/spreadsheets/d/1kvPoupSzsSFBNSztMzl04xMoSC3Kcx3CrjVf4yBmESU/edit?ts=5b5f17db#gid=227859291</td><td>PHESANT Transformation:20003_0 | | CAT-MUL-BINARY-VAR 1140860806 | | Indicator name x137_0_0 | | Remove indicator var NAs: 53 | | Remove indicator var <0: 0 | | Removed 0 examples != 1140860806 but with missing value (<0) | | sample 344272/16869(361141) | |-Notes:Code for treatment Negative codes indicate free-text entry.-Variable type:binary</td></tr>
<tr><td>binary.20003_1140860954.txt</td><td>1710174270056F5forCTG.txt.gz</td><td>0.1611</td><td>0.09618</td><td>1.675</td><td>0.09394</td><td>0.003754</td><td>0.001352</td><td>0.996</td><td>0.008534</td><td>0.002673</td><td>0.008359</td><td>Treatment/medication code: isosorbide mononitrate</td><td>FALSE</td><td></td><td></td><td></td><td>361141</td><td>1189</td><td>359952</td><td>UK Biobank</td><td>https://docs.google.com/spreadsheets/d/1kvPoupSzsSFBNSztMzl04xMoSC3Kcx3CrjVf4yBmESU/edit?ts=5b5f17db#gid=227859291</td><td>PHESANT Transformation:20003_0 | | CAT-MUL-BINARY-VAR 1140860954 | | Indicator name x137_0_0 | | Remove indicator var NAs: 53 | | Remove indicator var <0: 0 | | Removed 0 examples != 1140860954 but with missing value (<0) | | sample 359952/1189(361141) | |-Notes:Code for treatment Negative codes indicate free-text entry.-Variable type:binary</td></tr>
<tr><td>binary.20003_1140861958.txt</td><td>1710174270056F5forCTG.txt.gz</td><td>0.1752</td><td>0.04708</td><td>3.721</td><td>0.000199</td><td>0.02623</td><td>0.004226</td><td>1.056</td><td>0.029</td><td>0.006367</td><td>0.008602</td><td>Treatment/medication code: simvastatin</td><td>FALSE</td><td></td><td></td><td></td><td>361141</td><td>40921</td><td>320220</td><td>UK Biobank</td><td>https://docs.google.com/spreadsheets/d/1kvPoupSzsSFBNSztMzl04xMoSC3Kcx3CrjVf4yBmESU/edit?ts=5b5f17db#gid=227859291</td><td>PHESANT Transformation:20003_0 | | CAT-MUL-BINARY-VAR 1140861958 | | Indicator name x137_0_0 | | Remove indicator var NAs: 53 | | Remove indicator var <0: 0 | | Removed 0 examples != 1140861958 but with missing value (<0) | | sample 320220/40921(361141) | |-Notes:Code for treatment Negative codes indicate free-text entry.-Variable type:binary</td></tr>
</table>

binary.20003_1140861998.txt	1710174270056F5forCTG.txt.gz	0.1328	0.05206	2.551	0.01073	0.01698	0.002788	1.014	0.01302	0.008964	0.008549	Treatment/medication code: ventolin 100micrograms inhaler	FALSE					361141	10380	350761	UK Biobank	https://docs.google.com/spreadsheets/d/1kvPoupSzsSFBNSztMzl04xMoSC3Kcx3CrjVf4yBmESU/edit?ts=5b5f17db#gid=227859291	PHESANT Transformation:20003_0 | | CAT-MUL-BINARY-VAR 1140861998 | | Indicator name x137_0_0 | | Remove indicator var NAs: 53 | | Remove indicator var <0: 0 | | Removed 0 examples != 1140861998 but with missing value (<0) | | sample 350761/10380(361141) | |-Notes:Code for treatment Negative codes indicate free-text entry.-Variable type:binary
binary.20003_1140862148.txt	1710174270056F5forCTG.txt.gz	0.2474	0.1682	1.471	0.1414	0.001944	0.001464	1.017	0.008977	-0.002791	0.008639	Treatment/medication code: serevent 25mcg inhaler	FALSE					361141	996	360145	UK Biobank	https://docs.google.com/spreadsheets/d/1kvPoupSzsSFBNSztMzl04xMoSC3Kcx3CrjVf4yBmESU/edit?ts=5b5f17db#gid=227859291	PHESANT Transformation:20003_0 | | CAT-MUL-BINARY-VAR 1140862148 | | Indicator name x137_0_0 | | Remove indicator var NAs: 53 | | Remove indicator var <0: 0 | | Removed 0 examples != 1140862148 but with missing value (<0) | | sample 360145/996(361141) | |-Notes:Code for treatment Negative codes indicate free-text entry.-Variable type:binary
binary.20003_1140862382.txt	1710174270056F5forCTG.txt.gz	-0.06689	0.08289	-0.807	0.4196	0.005185	0.001564	0.997	0.0095	0.02254	0.00729	Treatment/medication code: becotide 50 inhaler	FALSE					361141	2206	358935	UK Biobank	https://docs.google.com/spreadsheets/d/1kvPoupSzsSFBNSztMzl04xMoSC3Kcx3CrjVf4yBmESU/edit?ts=5b5f17db#gid=227859291	PHESANT Transformation:20003_0 | | CAT-MUL-BINARY-VAR 1140862382 | | Indicator name x137_0_0 | | Remove indicator var NAs: 53 | | Remove indicator var <0: 0 | | Removed 0 examples != 1140862382 but with missing value (<0) | | sample 358935/2206(361141) | |-Notes:Code for treatment Negative codes indicate free-text entry.-Variable type:binary
binary.20003_1140862476.txt	1710174270056F5forCTG.txt.gz	0.2615	0.1185	2.208	0.02727	0.003006	0.001494	0.987	0.009083	-0.00196	0.007866	Treatment/medication code: beclazone 50 inhaler	FALSE					361141	1043	360098	UK Biobank	https://docs.google.com/spreadsheets/d/1kvPoupSzsSFBNSztMzl04xMoSC3Kcx3CrjVf4yBmESU/edit?ts=5b5f17db#gid=227859291	PHESANT Transformation:20003_0 | | CAT-MUL-BINARY-VAR 1140862476 | | Indicator name x137_0_0 | | Remove indicator var NAs: 53 | | Remove indicator var <0: 0 | | Removed 0 examples != 1140862476 but with missing value (<0) | | sample 360098/1043(361141) | |-Notes:Code for treatment Negative codes indicate free-text entry.-Variable type:binary
binary.20003_1140863152.txt	1710174270056F5forCTG.txt.gz	0.2021	0.09819	2.058	0.03957	0.003343	0.001594	0.985	0.008912	2.89E-06	0.006989	Treatment/medication code: diazepam	FALSE					361141	987	360154	UK Biobank	https://docs.google.com/spreadsheets/d/1kvPoupSzsSFBNSztMzl04xMoSC3Kcx3CrjVf4yBmESU/edit?ts=5b5f17db#gid=227859291	PHESANT Transformation:20003_0 | | CAT-MUL-BINARY-VAR 1140863152 | | Indicator name x137_0_0 | | Remove indicator var NAs: 53 | | Remove indicator var <0: 0 | | Removed 0 examples != 1140863152 but with missing value (<0) | | sample 360154/987(361141) | |-Notes:Code for treatment Negative codes indicate free-text entry.-Variable type:binary
binary.20003_1140863202.txt	1710174270056F5forCTG.txt.gz	0.424	0.1587	2.672	0.007547	0.002715	0.001445	0.991	0.008925	-0.006426	0.007597	Treatment/medication code: temazepam	FALSE					361141	759	360382	UK Biobank	https://docs.google.com/spreadsheets/d/1kvPoupSzsSFBNSztMzl04xMoSC3Kcx3CrjVf4yBmESU/edit?ts=5b5f17db#gid=227859291	PHESANT Transformation:20003_0 | | CAT-MUL-BINARY-VAR 1140863202 | | Indicator name x137_0_0 | | Remove indicator var NAs: 53 | | Remove indicator var <0: 0 | | Removed 0 examples != 1140863202 but with missing value (<0) | | sample 360382/759(361141) | |-Notes:Code for treatment Negative codes indicate free-text entry.-Variable type:binary
binary.20003_1140864070.txt	1710174270056F5forCTG.txt.gz	-0.09626	0.1686	-0.5709	0.5681	0.001799	0.001714	0.994	0.01141	0.0111	0.01063	Treatment/medication code: kapake tablet	FALSE					361141	104	361037	UK Biobank	https://docs.google.com/spreadsheets/d/1kvPoupSzsSFBNSztMzl04xMoSC3Kcx3CrjVf4yBmESU/edit?ts=5b5f17db#gid=227859291	PHESANT Transformation:20003_0 | | CAT-MUL-BINARY-VAR 1140864070 | | Indicator name x137_0_0 | | Remove indicator var NAs: 53 | | Remove indicator var <0: 0 | | Removed 0 examples != 1140864070 but with missing value (<0) | | sample 361037/104(361141) | |-Notes:Code for treatment Negative codes indicate free-text entry.-Variable type:binary
binary.20003_1140864752.txt	1710174270056F5forCTG.txt.gz	0.3535	0.0581	6.085	1.17E-09	0.01066	0.001832	1.02	0.01119	0.01004	0.007535	Treatment/medication code: lansoprazole	FALSE	Gastric				361141	12832	348309	UK Biobank	https://docs.google.com/spreadsheets/d/1kvPoupSzsSFBNSztMzl04xMoSC3Kcx3CrjVf4yBmESU/edit?ts=5b5f17db#gid=227859291	PHESANT Transformation:20003_0 | | CAT-MUL-BINARY-VAR 1140864752 | | Indicator name x137_0_0 | | Remove indicator var NAs: 53 | | Remove indicator var <0: 0 | | Removed 0 examples != 1140864752 but with missing value (<0) | | sample 348309/12832(361141) | |-Notes:Code for treatment Negative codes indicate free-text entry.-Variable type:binary
binary.20003_1140864952.txt	1710174270056F5forCTG.txt.gz	-0.05803	0.09999	-0.5803	0.5617	0.003639	0.001734	0.989	0.0113	0.008715	0.008686	Treatment/medication code: lisinopril+hydrochlorothiazide 10mg/12.5mg tablet	FALSE					361141	125	361016	UK Biobank	https://docs.google.com/spreadsheets/d/1kvPoupSzsSFBNSztMzl04xMoSC3Kcx3CrjVf4yBmESU/edit?ts=5b5f17db#gid=227859291	PHESANT Transformation:20003_0 | | CAT-MUL-BINARY-VAR 1140864952 | | Indicator name x137_0_0 | | Remove indicator var NAs: 53 | | Remove indicator var <0: 0 | | Removed 0 examples != 1140864952 but with missing value (<0) | | sample 361016/125(361141) | |-Notes:Code for treatment Negative codes indicate free-text entry.-Variable type:binary
binary.20003_1140864992.txt	1710174270056F5forCTG.txt.gz	0.4822	0.07041	6.849	7.44E-12	0.008305	0.001627	1.005	0.00975	0.00626	0.007207	Treatment/medication code: tramadol	FALSE	Pain				361141	4011	357130	UK Biobank	https://docs.google.com/spreadsheets/d/1kvPoupSzsSFBNSztMzl04xMoSC3Kcx3CrjVf4yBmESU/edit?ts=5b5f17db#gid=227859291	PHESANT Transformation:20003_0 | | CAT-MUL-BINARY-VAR 1140864992 | | Indicator name x137_0_0 | | Remove indicator var NAs: 53 | | Remove indicator var <0: 0 | | Removed 0 examples != 1140864992 but with missing value (<0) | | sample 357130/4011(361141) | |-Notes:Code for treatment Negative codes indicate free-text entry.-Variable type:binary
binary.20003_1140865354.txt	1710174270056F5forCTG.txt.gz	0.3718	0.1243	2.99	0.002787	0.003619	0.001564	0.995	0.009742	-0.004088	0.007408	Treatment/medication code: gaviscon liquid	FALSE					361141	1886	359255	UK Biobank	https://docs.google.com/spreadsheets/d/1kvPoupSzsSFBNSztMzl04xMoSC3Kcx3CrjVf4yBmESU/edit?ts=5b5f17db#gid=227859291	PHESANT Transformation:20003_0 | | CAT-MUL-BINARY-VAR 1140865354 | | Indicator name x137_0_0 | | Remove indicator var NAs: 53 | | Remove indicator var <0: 0 | | Removed 0 examples != 1140865354 but with missing value (<0) | | sample 359255/1886(361141) | |-Notes:Code for treatment Negative codes indicate free-text entry.-Variable type:binary
binary.20003_1140865634.txt	1710174270056F5forCTG.txt.gz	0.377	0.05336	7.065	1.60E-12	0.01502	0.001652	1.028	0.009845	0.008057	0.008855	Treatment/medication code: omeprazole	FALSE	Gastric				361141	21119	340022	UK Biobank	https://docs.google.com/spreadsheets/d/1kvPoupSzsSFBNSztMzl04xMoSC3Kcx3CrjVf4yBmESU/edit?ts=5b5f17db#gid=227859291	PHESANT Transformation:20003_0 | | CAT-MUL-BINARY-VAR 1140865634 | | Indicator name x137_0_0 | | Remove indicator var NAs: 53 | | Remove indicator var <0: 0 | | Removed 0 examples != 1140865634 but with missing value (<0) | | sample 340022/21119(361141) | |-Notes:Code for treatment Negative codes indicate free-text entry.-Variable type:binary
binary.20003_1140866122.txt	1710174270056F5forCTG.txt.gz	0.07506	0.06161	1.218	0.2231	0.00798	0.001617	0.981	0.009288	-0.002786	0.007647	Treatment/medication code: bendrofluazide	FALSE					361141	1922	359219	UK Biobank	https://docs.google.com/spreadsheets/d/1kvPoupSzsSFBNSztMzl04xMoSC3Kcx3CrjVf4yBmESU/edit?ts=5b5f17db#gid=227859291	PHESANT Transformation:20003_0 | | CAT-MUL-BINARY-VAR 1140866122 | | Indicator name x137_0_0 | | Remove indicator var NAs: 53 | | Remove indicator var <0: 0 | | Removed 0 examples != 1140866122 but with missing value (<0) | | sample 359219/1922(361141) | |-Notes:Code for treatment Negative codes indicate free-text entry.-Variable type:binary
binary.20003_1140866738.txt	1710174270056F5forCTG.txt.gz	0.09807	0.05587	1.755	0.07922	0.01435	0.002192	1.042	0.01256	0.002753	0.008729	Treatment/medication code: atenolol	FALSE					361141	13951	347190	UK Biobank	https://docs.google.com/spreadsheets/d/1kvPoupSzsSFBNSztMzl04xMoSC3Kcx3CrjVf4yBmESU/edit?ts=5b5f17db#gid=227859291	PHESANT Transformation:20003_0 | | CAT-MUL-BINARY-VAR 1140866738 | | Indicator name x137_0_0 | | Remove indicator var NAs: 53 | | Remove indicator var <0: 0 | | Removed 0 examples != 1140866738 but with missing value (<0) | | sample 347190/13951(361141) | |-Notes:Code for treatment Negative codes indicate free-text entry.-Variable type:binary
binary.20003_1140868226.txt	1710174270056F5forCTG.txt.gz	0.253	0.04635	5.459	4.78E-08	0.02227	0.00223	1.015	0.01133	0.01287	0.00912	Treatment/medication code: aspirin	FALSE	Pain				361141	48081	313060	UK Biobank	https://docs.google.com/spreadsheets/d/1kvPoupSzsSFBNSztMzl04xMoSC3Kcx3CrjVf4yBmESU/edit?ts=5b5f17db#gid=227859291	PHESANT Transformation:20003_0 | | CAT-MUL-BINARY-VAR 1140868226 | | Indicator name x137_0_0 | | Remove indicator var NAs: 53 | | Remove indicator var <0: 0 | | Removed 0 examples != 1140868226 but with missing value (<0) | | sample 313060/48081(361141) | |-Notes:Code for treatment Negative codes indicate free-text entry.-Variable type:binary
binary.20003_1140870788.txt	1710174270056F5forCTG.txt.gz	0.2401	0.1481	1.621	0.105	0.001739	0.001298	0.994	0.008948	-0.01753	0.007138	Treatment/medication code: calcium salts	FALSE					361141	2208	358933	UK Biobank	https://docs.google.com/spreadsheets/d/1kvPoupSzsSFBNSztMzl04xMoSC3Kcx3CrjVf4yBmESU/edit?ts=5b5f17db#gid=227859291	PHESANT Transformation:20003_0 | | CAT-MUL-BINARY-VAR 1140870788 | | Indicator name x137_0_0 | | Remove indicator var NAs: 53 | | Remove indicator var <0: 0 | | Removed 0 examples != 1140870788 but with missing value (<0) | | sample 358933/2208(361141) | |-Notes:Code for treatment Negative codes indicate free-text entry.-Variable type:binary
binary.20003_1140871024.txt	1710174270056F5forCTG.txt.gz	0.1322	0.122	1.083	0.2787	0.002717	0.001476	0.994	0.008832	-0.002681	0.007799	Treatment/medication code: vitamin b compound tablet	FALSE					361141	2619	358522	UK Biobank	https://docs.google.com/spreadsheets/d/1kvPoupSzsSFBNSztMzl04xMoSC3Kcx3CrjVf4yBmESU/edit?ts=5b5f17db#gid=227859291	PHESANT Transformation:20003_0 | | CAT-MUL-BINARY-VAR 1140871024 | | Indicator name x137_0_0 | | Remove indicator var NAs: 53 | | Remove indicator var <0: 0 | | Removed 0 examples != 1140871024 but with missing value (<0) | | sample 358522/2619(361141) | |-Notes:Code for treatment Negative codes indicate free-text entry.-Variable type:binary
binary.20003_1140871050.txt	1710174270056F5forCTG.txt.gz	0.0205	0.1067	0.1921	0.8477	0.003136	0.001559	1.002	0.009048	-0.004372	0.007828	Treatment/medication code: calcichew d3 tablet	FALSE					361141	3423	357718	UK Biobank	https://docs.google.com/spreadsheets/d/1kvPoupSzsSFBNSztMzl04xMoSC3Kcx3CrjVf4yBmESU/edit?ts=5b5f17db#gid=227859291	PHESANT Transformation:20003_0 | | CAT-MUL-BINARY-VAR 1140871050 | | Indicator name x137_0_0 | | Remove indicator var NAs: 53 | | Remove indicator var <0: 0 | | Removed 0 examples != 1140871050 but with missing value (<0) | | sample 357718/3423(361141) | |-Notes:Code for treatment Negative codes indicate free-text entry.-Variable type:binary
binary.20003_1140871052.txt	1710174270056F5forCTG.txt.gz	-0.2177	0.1845	-1.18	0.2381	0.001878	0.001615	0.99	0.0107	0.008285	0.009488	Treatment/medication code: calcium carbonate+colecalciferol 1.25g/5micrograms tablet	FALSE					361141	152	360989	UK Biobank	https://docs.google.com/spreadsheets/d/1kvPoupSzsSFBNSztMzl04xMoSC3Kcx3CrjVf4yBmESU/edit?ts=5b5f17db#gid=227859291	PHESANT Transformation:20003_0 | | CAT-MUL-BINARY-VAR 1140871052 | | Indicator name x137_0_0 | | Remove indicator var NAs: 53 | | Remove indicator var <0: 0 | | Removed 0 examples != 1140871052 but with missing value (<0) | | sample 360989/152(361141) | |-Notes:Code for treatment Negative codes indicate free-text entry.-Variable type:binary
binary.20003_1140871310.txt	1710174270056F5forCTG.txt.gz	0.2577	0.05078	5.075	3.87E-07	0.01558	0.00177	1.005	0.009792	0.006613	0.00781	Treatment/medication code: ibuprofen	FALSE	Pain				361141	45005	316136	UK Biobank	https://docs.google.com/spreadsheets/d/1kvPoupSzsSFBNSztMzl04xMoSC3Kcx3CrjVf4yBmESU/edit?ts=5b5f17db#gid=227859291	PHESANT Transformation:20003_0 | | CAT-MUL-BINARY-VAR 1140871310 | | Indicator name x137_0_0 | | Remove indicator var NAs: 53 | | Remove indicator var <0: 0 | | Removed 0 examples != 1140871310 but with missing value (<0) | | sample 316136/45005(361141) | |-Notes:Code for treatment Negative codes indicate free-text entry.-Variable type:binary
binary.20003_1140872228.txt	1710174270056F5forCTG.txt.gz	0.3353	0.09438	3.553	0.000381	0.004756	0.001742	0.988	0.01018	-0.00209	0.007264	Treatment/medication code: gabapentin	FALSE					361141	1674	359467	UK Biobank	https://docs.google.com/spreadsheets/d/1kvPoupSzsSFBNSztMzl04xMoSC3Kcx3CrjVf4yBmESU/edit?ts=5b5f17db#gid=227859291	PHESANT Transformation:20003_0 | | CAT-MUL-BINARY-VAR 1140872228 | | Indicator name x137_0_0 | | Remove indicator var NAs: 53 | | Remove indicator var <0: 0 | | Removed 0 examples != 1140872228 but with missing value (<0) | | sample 359467/1674(361141) | |-Notes:Code for treatment Negative codes indicate free-text entry.-Variable type:binary
binary.20003_1140874420.txt	1710174270056F5forCTG.txt.gz	0.3503	0.08379	4.181	2.90E-05	0.00636	0.001757	1.004	0.01061	0.007794	0.007706	Treatment/medication code: quinine	FALSE	Other (physical health)				361141	2429	358712	UK Biobank	https://docs.google.com/spreadsheets/d/1kvPoupSzsSFBNSztMzl04xMoSC3Kcx3CrjVf4yBmESU/edit?ts=5b5f17db#gid=227859291	PHESANT Transformation:20003_0 | | CAT-MUL-BINARY-VAR 1140874420 | | Indicator name x137_0_0 | | Remove indicator var NAs: 53 | | Remove indicator var <0: 0 | | Removed 0 examples != 1140874420 but with missing value (<0) | | sample 358712/2429(361141) | |-Notes:Code for treatment Negative codes indicate free-text entry.-Variable type:binary
binary.20003_1140874744.txt	1710174270056F5forCTG.txt.gz	0.2531	0.07042	3.594	0.000326	0.009165	0.001796	1.028	0.01156	-0.003411	0.008201	Treatment/medication code: gliclazide	FALSE					361141	3007	358134	UK Biobank	https://docs.google.com/spreadsheets/d/1kvPoupSzsSFBNSztMzl04xMoSC3Kcx3CrjVf4yBmESU/edit?ts=5b5f17db#gid=227859291	PHESANT Transformation:20003_0 | | CAT-MUL-BINARY-VAR 1140874744 | | Indicator name x137_0_0 | | Remove indicator var NAs: 53 | | Remove indicator var <0: 0 | | Removed 0 examples != 1140874744 but with missing value (<0) | | sample 358134/3007(361141) | |-Notes:Code for treatment Negative codes indicate free-text entry.-Variable type:binary

<table>
<tr><td>binary.20003_1140874930.txt</td><td>1710174270056F5forCTG.txt.gz</td><td>-0.05551</td><td>0.09427</td><td>-0.5888</td><td>0.556</td><td>0.003753</td><td>0.001608</td><td>0.99</td><td>0.009101</td><td>0.01106</td><td>0.008171</td><td>Treatment/medication code: prednisolone</td><td>FALSE</td><td></td><td></td><td></td><td>361141</td><td>2103</td><td>359038</td><td>UK Biobank</td><td>https://docs.google.com/spreadsheets/d/1kvPoupSzsSFBNSztMzl04xMoSC3Kcx3CrjVf4yBmESU/edit?ts=5b5f17db#gid=227859291</td><td>PHESANT Transformation:20003_0 | | CAT-MUL-BINARY-VAR 1140874930 | | Indicator name x137_0_0 | | Remove indicator var NAs: 53 | | Remove indicator var <0: 0 | | Removed 0 examples != 1140874930 but with missing value (<0) | | sample 359038/2103(361141) | |-Notes:Code for treatment Negative codes indicate free-text entry.-Variable type:binary</td></tr>
<tr><td>binary.20003_1140875408.txt</td><td>1710174270056F5forCTG.txt.gz</td><td>0.1137</td><td>0.04304</td><td>2.641</td><td>0.008255</td><td>0.0228</td><td>0.007394</td><td>0.978</td><td>0.0339</td><td>-0.004441</td><td>0.007972</td><td>Treatment/medication code: allopurinol</td><td>FALSE</td><td></td><td></td><td></td><td>361141</td><td>4108</td><td>357033</td><td>UK Biobank</td><td>https://docs.google.com/spreadsheets/d/1kvPoupSzsSFBNSztMzl04xMoSC3Kcx3CrjVf4yBmESU/edit?ts=5b5f17db#gid=227859291</td><td>PHESANT Transformation:20003_0 | | CAT-MUL-BINARY-VAR 1140875408 | | Indicator name x137_0_0 | | Remove indicator var NAs: 53 | | Remove indicator var <0: 0 | | Removed 0 examples != 1140875408 but with missing value (<0) | | sample 357033/4108(361141) | |-Notes:Code for treatment Negative codes indicate free-text entry.-Variable type:binary</td></tr>
<tr><td>binary.20003_1140875486.txt</td><td>1710174270056F5forCTG.txt.gz</td><td>-0.01611</td><td>0.1021</td><td>-0.1578</td><td>0.8746</td><td>0.004205</td><td>0.001945</td><td>0.989</td><td>0.01184</td><td>0.009795</td><td>0.01038</td><td>Treatment/medication code: colchicine</td><td>FALSE</td><td></td><td></td><td></td><td>361141</td><td>190</td><td>360951</td><td>UK Biobank</td><td>https://docs.google.com/spreadsheets/d/1kvPoupSzsSFBNSztMzl04xMoSC3Kcx3CrjVf4yBmESU/edit?ts=5b5f17db#gid=227859291</td><td>PHESANT Transformation:20003_0 | | CAT-MUL-BINARY-VAR 1140875486 | | Indicator name x137_0_0 | | Remove indicator var NAs: 53 | | Remove indicator var <0: 0 | | Removed 0 examples != 1140875486 but with missing value (<0) | | sample 360951/190(361141) | |-Notes:Code for treatment Negative codes indicate free-text entry.-Variable type:binary</td></tr>
<tr><td>binary.20003_1140876076.txt</td><td>1710174270056F5forCTG.txt.gz</td><td>-0.1253</td><td>0.1071</td><td>-1.169</td><td>0.2422</td><td>0.002979</td><td>0.001571</td><td>0.995</td><td>0.009622</td><td>0.00831</td><td>0.0072</td><td>Treatment/medication code: flixonase 50micrograms aqueous nasal spray</td><td>FALSE</td><td></td><td></td><td></td><td>361141</td><td>1424</td><td>359717</td><td>UK Biobank</td><td>https://docs.google.com/spreadsheets/d/1kvPoupSzsSFBNSztMzl04xMoSC3Kcx3CrjVf4yBmESU/edit?ts=5b5f17db#gid=227859291</td><td>PHESANT Transformation:20003_0 | | CAT-MUL-BINARY-VAR 1140876076 | | Indicator name x137_0_0 | | Remove indicator var NAs: 53 | | Remove indicator var <0: 0 | | Removed 0 examples != 1140876076 but with missing value (<0) | | sample 359717/1424(361141) | |-Notes:Code for treatment Negative codes indicate free-text entry.-Variable type:binary</td></tr>
<tr><td>binary.20003_1140876136.txt</td><td>1710174270056F5forCTG.txt.gz</td><td>0.05791</td><td>0.1379</td><td>0.4199</td><td>0.6746</td><td>0.001734</td><td>0.001405</td><td>1.014</td><td>0.009271</td><td>-0.004237</td><td>0.007515</td><td>Treatment/medication code: beconase 50micrograms nasal spray</td><td>FALSE</td><td></td><td></td><td></td><td>361141</td><td>2415</td><td>358726</td><td>UK Biobank</td><td>https://docs.google.com/spreadsheets/d/1kvPoupSzsSFBNSztMzl04xMoSC3Kcx3CrjVf4yBmESU/edit?ts=5b5f17db#gid=227859291</td><td>PHESANT Transformation:20003_0 | | CAT-MUL-BINARY-VAR 1140876136 | | Indicator name x137_0_0 | | Remove indicator var NAs: 53 | | Remove indicator var <0: 0 | | Removed 0 examples != 1140876136 but with missing value (<0) | | sample 358726/2415(361141) | |-Notes:Code for treatment Negative codes indicate free-text entry.-Variable type:binary</td></tr>
<tr><td>binary.20003_1140876146.txt</td><td>1710174270056F5forCTG.txt.gz</td><td>0.1473</td><td>0.1113</td><td>1.324</td><td>0.1854</td><td>0.003872</td><td>0.001634</td><td>0.979</td><td>0.01062</td><td>-0.006022</td><td>0.009925</td><td>Treatment/medication code: rhinocort 50micrograms nasal spray</td><td>FALSE</td><td></td><td></td><td></td><td>361141</td><td>160</td><td>360981</td><td>UK Biobank</td><td>https://docs.google.com/spreadsheets/d/1kvPoupSzsSFBNSztMzl04xMoSC3Kcx3CrjVf4yBmESU/edit?ts=5b5f17db#gid=227859291</td><td>PHESANT Transformation:20003_0 | | CAT-MUL-BINARY-VAR 1140876146 | | Indicator name x137_0_0 | | Remove indicator var NAs: 53 | | Remove indicator var <0: 0 | | Removed 0 examples != 1140876146 but with missing value (<0) | | sample 360981/160(361141) | |-Notes:Code for treatment Negative codes indicate free-text entry.-Variable type:binary</td></tr>
<tr><td>binary.20003_1140876592.txt</td><td>1710174270056F5forCTG.txt.gz</td><td>-0.01914</td><td>0.1044</td><td>-0.1833</td><td>0.8546</td><td>0.002394</td><td>0.001497</td><td>1.011</td><td>0.009111</td><td>0.006272</td><td>0.007514</td><td>Treatment/medication code: multivitamin+mineral preparations</td><td>FALSE</td><td></td><td></td><td></td><td>361141</td><td>4997</td><td>356144</td><td>UK Biobank</td><td>https://docs.google.com/spreadsheets/d/1kvPoupSzsSFBNSztMzl04xMoSC3Kcx3CrjVf4yBmESU/edit?ts=5b5f17db#gid=227859291</td><td>PHESANT Transformation:20003_0 | | CAT-MUL-BINARY-VAR 1140876592 | | Indicator name x137_0_0 | | Remove indicator var NAs: 53 | | Remove indicator var <0: 0 | | Removed 0 examples != 1140876592 but with missing value (<0) | | sample 356144/4997(361141) | |-Notes:Code for treatment Negative codes indicate free-text entry.-Variable type:binary</td></tr>
<tr><td>binary.20003_1140878036.txt</td><td>1710174270056F5forCTG.txt.gz</td><td>0.07894</td><td>0.08328</td><td>0.9479</td><td>0.3432</td><td>0.004655</td><td>0.001584</td><td>0.975</td><td>0.00939</td><td>0.003596</td><td>0.008339</td><td>Treatment/medication code: diclofenac sodium+misoprostol</td><td>FALSE</td><td></td><td></td><td></td><td>361141</td><td>251</td><td>360890</td><td>UK Biobank</td><td>https://docs.google.com/spreadsheets/d/1kvPoupSzsSFBNSztMzl04xMoSC3Kcx3CrjVf4yBmESU/edit?ts=5b5f17db#gid=227859291</td><td>PHESANT Transformation:20003_0 | | CAT-MUL-BINARY-VAR 1140878036 | | Indicator name x137_0_0 | | Remove indicator var NAs: 53 | | Remove indicator var <0: 0 | | Removed 0 examples != 1140878036 but with missing value (<0) | | sample 360890/251(361141) | |-Notes:Code for treatment Negative codes indicate free-text entry.-Variable type:binary</td></tr>
<tr><td>binary.20003_1140879406.txt</td><td>1710174270056F5forCTG.txt.gz</td><td>0.4123</td><td>0.1164</td><td>3.541</td><td>0.000399</td><td>0.005589</td><td>0.001751</td><td>0.998</td><td>0.01074</td><td>0.001184</td><td>0.00823</td><td>Treatment/medication code: ranitidine</td><td>FALSE</td><td></td><td></td><td></td><td>361141</td><td>5935</td><td>355206</td><td>UK Biobank</td><td>https://docs.google.com/spreadsheets/d/1kvPoupSzsSFBNSztMzl04xMoSC3Kcx3CrjVf4yBmESU/edit?ts=5b5f17db#gid=227859291</td><td>PHESANT Transformation:20003_0 | | CAT-MUL-BINARY-VAR 1140879406 | | Indicator name x137_0_0 | | Remove indicator var NAs: 53 | | Remove indicator var <0: 0 | | Removed 0 examples != 1140879406 but with missing value (<0) | | sample 355206/5935(361141) | |-Notes:Code for treatment Negative codes indicate free-text entry.-Variable type:binary</td></tr>
<tr><td>binary.20003_1140879540.txt</td><td>1710174270056F5forCTG.txt.gz</td><td>0.4555</td><td>0.1279</td><td>3.561</td><td>0.000369</td><td>0.003604</td><td>0.001387</td><td>1.005</td><td>0.008498</td><td>-0.01617</td><td>0.008818</td><td>Treatment/medication code: fluoxetine</td><td>FALSE</td><td></td><td></td><td></td><td>361141</td><td>4140</td><td>357001</td><td>UK Biobank</td><td>https://docs.google.com/spreadsheets/d/1kvPoupSzsSFBNSztMzl04xMoSC3Kcx3CrjVf4yBmESU/edit?ts=5b5f17db#gid=227859291</td><td>PHESANT Transformation:20003_0 | | CAT-MUL-BINARY-VAR 1140879540 | | Indicator name x137_0_0 | | Remove indicator var NAs: 53 | | Remove indicator var <0: 0 | | Removed 0 examples != 1140879540 but with missing value (<0) | | sample 357001/4140(361141) | |-Notes:Code for treatment Negative codes indicate free-text entry.-Variable type:binary</td></tr>
<tr><td>binary.20003_1140879616.txt</td><td>1710174270056F5forCTG.txt.gz</td><td>0.4021</td><td>0.08738</td><td>4.601</td><td>4.20E-06</td><td>0.005814</td><td>0.001888</td><td>1.016</td><td>0.01137</td><td>0.01015</td><td>0.008177</td><td>Treatment/medication code: amitriptyline</td><td>FALSE</td><td>Pain</td><td></td><td></td><td>361141</td><td>6824</td><td>354317</td><td>UK Biobank</td><td>https://docs.google.com/spreadsheets/d/1kvPoupSzsSFBNSztMzl04xMoSC3Kcx3CrjVf4yBmESU/edit?ts=5b5f17db#gid=227859291</td><td>PHESANT Transformation:20003_0 | | CAT-MUL-BINARY-VAR 1140879616 | | Indicator name x137_0_0 | | Remove indicator var NAs: 53 | | Remove indicator var <0: 0 | | Removed 0 examples != 1140879616 but with missing value (<0) | | sample 354317/6824(361141) | |-Notes:Code for treatment Negative codes indicate free-text entry.-Variable type:binary</td></tr>
<tr><td>binary.20003_1140879760.txt</td><td>1710174270056F5forCTG.txt.gz</td><td>0.08555</td><td>0.08136</td><td>1.051</td><td>0.293</td><td>0.005321</td><td>0.001537</td><td>1.005</td><td>0.00986</td><td>0.01256</td><td>0.008289</td><td>Treatment/medication code: bisoprolol</td><td>FALSE</td><td></td><td></td><td></td><td>361141</td><td>5304</td><td>355837</td><td>UK Biobank</td><td>https://docs.google.com/spreadsheets/d/1kvPoupSzsSFBNSztMzl04xMoSC3Kcx3CrjVf4yBmESU/edit?ts=5b5f17db#gid=227859291</td><td>PHESANT Transformation:20003_0 | | CAT-MUL-BINARY-VAR 1140879760 | | Indicator name x137_0_0 | | Remove indicator var NAs: 53 | | Remove indicator var <0: 0 | | Removed 0 examples != 1140879760 but with missing value (<0) | | sample 355837/5304(361141) | |-Notes:Code for treatment Negative codes indicate free-text entry.-Variable type:binary</td></tr>
<tr><td>binary.20003_1140879778.txt</td><td>1710174270056F5forCTG.txt.gz</td><td>0.1991</td><td>0.07512</td><td>2.651</td><td>0.008037</td><td>0.00851</td><td>0.001777</td><td>1.005</td><td>0.01042</td><td>-0.01287</td><td>0.008872</td><td>Treatment/medication code: doxazosin</td><td>FALSE</td><td></td><td></td><td></td><td>361141</td><td>4043</td><td>357098</td><td>UK Biobank</td><td>https://docs.google.com/spreadsheets/d/1kvPoupSzsSFBNSztMzl04xMoSC3Kcx3CrjVf4yBmESU/edit?ts=5b5f17db#gid=227859291</td><td>PHESANT Transformation:20003_0 | | CAT-MUL-BINARY-VAR 1140879778 | | Indicator name x137_0_0 | | Remove indicator var NAs: 53 | | Remove indicator var <0: 0 | | Removed 0 examples != 1140879778 but with missing value (<0) | | sample 357098/4043(361141) | |-Notes:Code for treatment Negative codes indicate free-text entry.-Variable type:binary</td></tr>
<tr><td>binary.20003_1140879802.txt</td><td>1710174270056F5forCTG.txt.gz</td><td>0.1364</td><td>0.05029</td><td>2.711</td><td>0.0067</td><td>0.02128</td><td>0.002339</td><td>1.015</td><td>0.01089</td><td>-0.01157</td><td>0.008775</td><td>Treatment/medication code: amlodipine</td><td>FALSE</td><td></td><td></td><td></td><td>361141</td><td>14588</td><td>346553</td><td>UK Biobank</td><td>https://docs.google.com/spreadsheets/d/1kvPoupSzsSFBNSztMzl04xMoSC3Kcx3CrjVf4yBmESU/edit?ts=5b5f17db#gid=227859291</td><td>PHESANT Transformation:20003_0 | | CAT-MUL-BINARY-VAR 1140879802 | | Indicator name x137_0_0 | | Remove indicator var NAs: 53 | | Remove indicator var <0: 0 | | Removed 0 examples != 1140879802 but with missing value (<0) | | sample 346553/14588(361141) | |-Notes:Code for treatment Negative codes indicate free-text entry.-Variable type:binary</td></tr>
<tr><td>binary.20003_1140879842.txt</td><td>1710174270056F5forCTG.txt.gz</td><td>0.1423</td><td>0.07976</td><td>1.784</td><td>0.07444</td><td>0.005091</td><td>0.001509</td><td>0.991</td><td>0.008398</td><td>-0.007985</td><td>0.007564</td><td>Treatment/medication code: propranolol</td><td>FALSE</td><td></td><td></td><td></td><td>361141</td><td>2405</td><td>358736</td><td>UK Biobank</td><td>https://docs.google.com/spreadsheets/d/1kvPoupSzsSFBNSztMzl04xMoSC3Kcx3CrjVf4yBmESU/edit?ts=5b5f17db#gid=227859291</td><td>PHESANT Transformation:20003_0 | | CAT-MUL-BINARY-VAR 1140879842 | | Indicator name x137_0_0 | | Remove indicator var NAs: 53 | | Remove indicator var <0: 0 | | Removed 0 examples != 1140879842 but with missing value (<0) | | sample 358736/2405(361141) | |-Notes:Code for treatment Negative codes indicate free-text entry.-Variable type:binary</td></tr>
<tr><td>binary.20003_1140881856.txt</td><td>1710174270056F5forCTG.txt.gz</td><td>0.412</td><td>0.1382</td><td>2.98</td><td>0.002878</td><td>0.003702</td><td>0.001777</td><td>1.015</td><td>0.009729</td><td>0.0007024</td><td>0.008004</td><td>Treatment/medication code: salbutamol</td><td>FALSE</td><td></td><td></td><td></td><td>361141</td><td>4305</td><td>356836</td><td>UK Biobank</td><td>https://docs.google.com/spreadsheets/d/1kvPoupSzsSFBNSztMzl04xMoSC3Kcx3CrjVf4yBmESU/edit?ts=5b5f17db#gid=227859291</td><td>PHESANT Transformation:20003_0 | | CAT-MUL-BINARY-VAR 1140881856 | | Indicator name x137_0_0 | | Remove indicator var NAs: 53 | | Remove indicator var <0: 0 | | Removed 0 examples != 1140881856 but with missing value (<0) | | sample 356836/4305(361141) | |-Notes:Code for treatment Negative codes indicate free-text entry.-Variable type:binary</td></tr>
<tr><td>binary.20003_1140883066.txt</td><td>1710174270056F5forCTG.txt.gz</td><td>0.1727</td><td>0.0742</td><td>2.327</td><td>0.01995</td><td>0.007503</td><td>0.001968</td><td>1.011</td><td>0.0112</td><td>8.40E-05</td><td>0.00827</td><td>Treatment/medication code: insulin product</td><td>FALSE</td><td></td><td></td><td></td><td>361141</td><td>3545</td><td>357596</td><td>UK Biobank</td><td>https://docs.google.com/spreadsheets/d/1kvPoupSzsSFBNSztMzl04xMoSC3Kcx3CrjVf4yBmESU/edit?ts=5b5f17db#gid=227859291</td><td>PHESANT Transformation:20003_0 | | CAT-MUL-BINARY-VAR 1140883066 | | Indicator name x137_0_0 | | Remove indicator var NAs: 53 | | Remove indicator var <0: 0 | | Removed 0 examples != 1140883066 but with missing value (<0) | | sample 357596/3545(361141) | |-Notes:Code for treatment Negative codes indicate free-text entry.-Variable type:binary</td></tr>
<tr><td>binary.20003_1140883504.txt</td><td>1710174270056F5forCTG.txt.gz</td><td>0.02711</td><td>0.07043</td><td>0.385</td><td>0.7003</td><td>0.006096</td><td>0.001629</td><td>1.004</td><td>0.009612</td><td>0.01348</td><td>0.007715</td><td>Treatment/medication code: cetirizine</td><td>FALSE</td><td></td><td></td><td></td><td>361141</td><td>4070</td><td>357071</td><td>UK Biobank</td><td>https://docs.google.com/spreadsheets/d/1kvPoupSzsSFBNSztMzl04xMoSC3Kcx3CrjVf4yBmESU/edit?ts=5b5f17db#gid=227859291</td><td>PHESANT Transformation:20003_0 | | CAT-MUL-BINARY-VAR 1140883504 | | Indicator name x137_0_0 | | Remove indicator var NAs: 53 | | Remove indicator var <0: 0 | | Removed 0 examples != 1140883504 but with missing value (<0) | | sample 357071/4070(361141) | |-Notes:Code for treatment Negative codes indicate free-text entry.-Variable type:binary</td></tr>
<tr><td>binary.20003_1140883548.txt</td><td>1710174270056F5forCTG.txt.gz</td><td>0.1974</td><td>0.1458</td><td>1.353</td><td>0.1759</td><td>0.002112</td><td>0.001506</td><td>0.99</td><td>0.009084</td><td>0.01273</td><td>0.008303</td><td>Treatment/medication code: ipratropium</td><td>FALSE</td><td></td><td></td><td></td><td>361141</td><td>476</td><td>360665</td><td>UK Biobank</td><td>https://docs.google.com/spreadsheets/d/1kvPoupSzsSFBNSztMzl04xMoSC3Kcx3CrjVf4yBmESU/edit?ts=5b5f17db#gid=227859291</td><td>PHESANT Transformation:20003_0 | | CAT-MUL-BINARY-VAR 1140883548 | | Indicator name x137_0_0 | | Remove indicator var NAs: 53 | | Remove indicator var <0: 0 | | Removed 0 examples != 1140883548 but with missing value (<0) | | sample 360665/476(361141) | |-Notes:Code for treatment Negative codes indicate free-text entry.-Variable type:binary</td></tr>
<tr><td>binary.20003_1140883568.txt</td><td>1710174270056F5forCTG.txt.gz</td><td>0.2032</td><td>0.1131</td><td>1.797</td><td>0.07234</td><td>0.002987</td><td>0.001605</td><td>0.986</td><td>0.0092</td><td>-0.008269</td><td>0.00826</td><td>Treatment/medication code: oxybutynin</td><td>FALSE</td><td></td><td></td><td></td><td>361141</td><td>827</td><td>360314</td><td>UK Biobank</td><td>https://docs.google.com/spreadsheets/d/1kvPoupSzsSFBNSztMzl04xMoSC3Kcx3CrjVf4yBmESU/edit?ts=5b5f17db#gid=227859291</td><td>PHESANT Transformation:20003_0 | | CAT-MUL-BINARY-VAR 1140883568 | | Indicator name x137_0_0 | | Remove indicator var NAs: 53 | | Remove indicator var <0: 0 | | Removed 0 examples != 1140883568 but with missing value (<0) | | sample 360314/827(361141) | |-Notes:Code for treatment Negative codes indicate free-text entry.-Variable type:binary</td></tr>
<tr><td>binary.20003_1140884412.txt</td><td>1710174270056F5forCTG.txt.gz</td><td>0.2613</td><td>0.1222</td><td>2.139</td><td>0.03245</td><td>0.003025</td><td>0.001681</td><td>1.01</td><td>0.009371</td><td>-0.005628</td><td>0.008052</td><td>Treatment/medication code: sumatriptan</td><td>FALSE</td><td></td><td></td><td></td><td>361141</td><td>986</td><td>360155</td><td>UK Biobank</td><td>https://docs.google.com/spreadsheets/d/1kvPoupSzsSFBNSztMzl04xMoSC3Kcx3CrjVf4yBmESU/edit?ts=5b5f17db#gid=227859291</td><td>PHESANT Transformation:20003_0 | | CAT-MUL-BINARY-VAR 1140884412 | | Indicator name x137_0_0 | | Remove indicator var NAs: 53 | | Remove indicator var <0: 0 | | Removed 0 examples != 1140884412 but with missing value (<0) | | sample 360155/986(361141) | |-Notes:Code for treatment Negative codes indicate free-text entry.-Variable type:binary</td></tr>
<tr><td>binary.20003_1140884444.txt</td><td>1710174270056F5forCTG.txt.gz</td><td>0.283</td><td>0.09282</td><td>3.049</td><td>0.002297</td><td>0.004828</td><td>0.001628</td><td>0.99</td><td>0.009895</td><td>-0.000839</td><td>0.007918</td><td>Treatment/medication code: codeine</td><td>FALSE</td><td></td><td></td><td></td><td>361141</td><td>1870</td><td>359271</td><td>UK Biobank</td><td>https://docs.google.com/spreadsheets/d/1kvPoupSzsSFBNSztMzl04xMoSC3Kcx3CrjVf4yBmESU/edit?ts=5b5f17db#gid=227859291</td><td>PHESANT Transformation:20003_0 | | CAT-MUL-BINARY-VAR 1140884444 | | Indicator name x137_0_0 | | Remove indicator var NAs: 53 | | Remove indicator var <0: 0 | | Removed 0 examples != 1140884444 but with missing value (<0) | | sample 359271/1870(361141) | |-Notes:Code for treatment Negative codes indicate free-text entry.-Variable type:binary</td></tr>
<tr><td>binary.20003_1140884464.txt</td><td>1710174270056F5forCTG.txt.gz</td><td>0.4416</td><td>0.1256</td><td>3.517</td><td>0.000436</td><td>0.003517</td><td>0.001541</td><td>0.998</td><td>0.009676</td><td>0.01124</td><td>0.008143</td><td>Treatment/medication code: dihydrocodeine</td><td>FALSE</td><td></td><td></td><td></td><td>361141</td><td>1290</td><td>359851</td><td>UK Biobank</td><td>https://docs.google.com/spreadsheets/d/1kvPoupSzsSFBNSztMzl04xMoSC3Kcx3CrjVf4yBmESU/edit?ts=5b5f17db#gid=227859291</td><td>PHESANT Transformation:20003_0 | | CAT-MUL-BINARY-VAR 1140884464 | | Indicator name x137_0_0 | | Remove indicator var NAs: 53 | | Remove indicator var <0: 0 | | Removed 0 examples != 1140884464 but with missing value (<0) | | sample 359851/1290(361141) | |-Notes:Code for treatment Negative codes indicate free-text entry.-Variable type:binary</td></tr>
</table>

<table>
<tr><td>binary.20003_1140884488.txt</td><td>1710174270056F5forCTG.txt.gz</td><td>0.3518</td><td>0.07399</td><td>4.755</td><td>1.98E-06</td><td>0.008587</td><td>0.001693</td><td>0.986</td><td>0.009508</td><td>-0.0109</td><td>0.008719</td><td>Treatment/medication code: diclofenac</td><td>FALSE</td><td>Pain</td><td></td><td></td><td>361141</td><td>7545</td><td>353596</td><td>UK Biobank</td><td>https://docs.google.com/spreadsheets/d/1kvPoupSzsSFBNSztMzl04xMoSC3Kcx3CrjVf4yBmESU/edit?ts=5b5f17db#gid=227859291</td><td>PHESANT Transformation:20003_0 | | CAT-MUL-BINARY-VAR 1140884488 | | Indicator name x137_0_0 | | Remove indicator var NAs: 53 | | Remove indicator var <0: 0 | | Removed 0 examples != 1140884488 but with missing value (<0) | | sample 353596/7545(361141) | | -Notes:Code for treatment Negative codes indicate free-text entry.-Variable type:binary</td></tr>
<tr><td>binary.20003_1140884516.txt</td><td>1710174270056F5forCTG.txt.gz</td><td>0.105</td><td>0.08547</td><td>1.228</td><td>0.2194</td><td>0.007672</td><td>0.002604</td><td>1.009</td><td>0.01239</td><td>-0.006835</td><td>0.008658</td><td>Treatment/medication code: thyroxine product</td><td>FALSE</td><td></td><td></td><td></td><td>361141</td><td>4246</td><td>356895</td><td>UK Biobank</td><td>https://docs.google.com/spreadsheets/d/1kvPoupSzsSFBNSztMzl04xMoSC3Kcx3CrjVf4yBmESU/edit?ts=5b5f17db#gid=227859291</td><td>PHESANT Transformation:20003_0 | | CAT-MUL-BINARY-VAR 1140884516 | | Indicator name x137_0_0 | | Remove indicator var NAs: 53 | | Remove indicator var <0: 0 | | Removed 0 examples != 1140884516 but with missing value (<0) | | sample 356895/4246(361141) | | -Notes:Code for treatment Negative codes indicate free-text entry.-Variable type:binary</td></tr>
<tr><td>binary.20003_1140884600.txt</td><td>1710174270056F5forCTG.txt.gz</td><td>0.2139</td><td>0.04281</td><td>4.996</td><td>5.86E-07</td><td>0.0283</td><td>0.002444</td><td>1.043</td><td>0.0132</td><td>0.01158</td><td>0.008646</td><td>Treatment/medication code: metformin</td><td>FALSE</td><td>Other (physical health)</td><td></td><td></td><td>361141</td><td>8968</td><td>352173</td><td>UK Biobank</td><td>https://docs.google.com/spreadsheets/d/1kvPoupSzsSFBNSztMzl04xMoSC3Kcx3CrjVf4yBmESU/edit?ts=5b5f17db#gid=227859291</td><td>PHESANT Transformation:20003_0 | | CAT-MUL-BINARY-VAR 1140884600 | | Indicator name x137_0_0 | | Remove indicator var NAs: 53 | | Remove indicator var <0: 0 | | Removed 0 examples != 1140884600 but with missing value (<0) | | sample 352173/8968(361141) | | -Notes:Code for treatment Negative codes indicate free-text entry.-Variable type:binary</td></tr>
<tr><td>binary.20003_1140888266.txt</td><td>1710174270056F5forCTG.txt.gz</td><td>0.006842</td><td>0.09603</td><td>0.07125</td><td>0.9432</td><td>0.004034</td><td>0.00151</td><td>1.016</td><td>0.01053</td><td>0.006818</td><td>0.008179</td><td>Treatment/medication code: warfarin</td><td>FALSE</td><td></td><td></td><td></td><td>361141</td><td>3651</td><td>357490</td><td>UK Biobank</td><td>https://docs.google.com/spreadsheets/d/1kvPoupSzsSFBNSztMzl04xMoSC3Kcx3CrjVf4yBmESU/edit?ts=5b5f17db#gid=227859291</td><td>PHESANT Transformation:20003_0 | | CAT-MUL-BINARY-VAR 1140888266 | | Indicator name x137_0_0 | | Remove indicator var NAs: 53 | | Remove indicator var <0: 0 | | Removed 0 examples != 1140888266 but with missing value (<0) | | sample 357490/3651(361141) | | -Notes:Code for treatment Negative codes indicate free-text entry.-Variable type:binary</td></tr>
<tr><td>binary.20003_1140888552.txt</td><td>1710174270056F5forCTG.txt.gz</td><td>0.04656</td><td>0.08513</td><td>0.547</td><td>0.5844</td><td>0.004528</td><td>0.001645</td><td>1.015</td><td>0.009411</td><td>0.007082</td><td>0.008197</td><td>Treatment/medication code: enalapril</td><td>FALSE</td><td></td><td></td><td></td><td>361141</td><td>2803</td><td>358338</td><td>UK Biobank</td><td>https://docs.google.com/spreadsheets/d/1kvPoupSzsSFBNSztMzl04xMoSC3Kcx3CrjVf4yBmESU/edit?ts=5b5f17db#gid=227859291</td><td>PHESANT Transformation:20003_0 | | CAT-MUL-BINARY-VAR 1140888552 | | Indicator name x137_0_0 | | Remove indicator var NAs: 53 | | Remove indicator var <0: 0 | | Removed 0 examples != 1140888552 but with missing value (<0) | | sample 358338/2803(361141) | | -Notes:Code for treatment Negative codes indicate free-text entry.-Variable type:binary</td></tr>
<tr><td>binary.20003_1140888560.txt</td><td>1710174270056F5forCTG.txt.gz</td><td>0.1421</td><td>0.09563</td><td>1.486</td><td>0.1372</td><td>0.00467</td><td>0.001556</td><td>1.017</td><td>0.009273</td><td>-0.00367</td><td>0.008492</td><td>Treatment/medication code: perindopril</td><td>FALSE</td><td></td><td></td><td></td><td>361141</td><td>5157</td><td>355984</td><td>UK Biobank</td><td>https://docs.google.com/spreadsheets/d/1kvPoupSzsSFBNSztMzl04xMoSC3Kcx3CrjVf4yBmESU/edit?ts=5b5f17db#gid=227859291</td><td>PHESANT Transformation:20003_0 | | CAT-MUL-BINARY-VAR 1140888560 | | Indicator name x137_0_0 | | Remove indicator var NAs: 53 | | Remove indicator var <0: 0 | | Removed 0 examples != 1140888560 but with missing value (<0) | | sample 355984/5157(361141) | | -Notes:Code for treatment Negative codes indicate free-text entry.-Variable type:binary</td></tr>
<tr><td>binary.20003_1140888570.txt</td><td>1710174270056F5forCTG.txt.gz</td><td>0.1078</td><td>0.114</td><td>0.9453</td><td>0.3445</td><td>0.002638</td><td>0.001381</td><td>0.993</td><td>0.008761</td><td>-0.01127</td><td>0.008005</td><td>Treatment/medication code: flecainide</td><td>FALSE</td><td></td><td></td><td></td><td>361141</td><td>604</td><td>360537</td><td>UK Biobank</td><td>https://docs.google.com/spreadsheets/d/1kvPoupSzsSFBNSztMzl04xMoSC3Kcx3CrjVf4yBmESU/edit?ts=5b5f17db#gid=227859291</td><td>PHESANT Transformation:20003_0 | | CAT-MUL-BINARY-VAR 1140888570 | | Indicator name x137_0_0 | | Remove indicator var NAs: 53 | | Remove indicator var <0: 0 | | Removed 0 examples != 1140888570 but with missing value (<0) | | sample 360537/604(361141) | | -Notes:Code for treatment Negative codes indicate free-text entry.-Variable type:binary</td></tr>
<tr><td>binary.20003_1140888646.txt</td><td>1710174270056F5forCTG.txt.gz</td><td>0.08707</td><td>0.08401</td><td>1.036</td><td>0.3</td><td>0.005601</td><td>0.001817</td><td>1.001</td><td>0.01006</td><td>-0.01021</td><td>0.008361</td><td>Treatment/medication code: felodipine</td><td>FALSE</td><td></td><td></td><td></td><td>361141</td><td>3090</td><td>358051</td><td>UK Biobank</td><td>https://docs.google.com/spreadsheets/d/1kvPoupSzsSFBNSztMzl04xMoSC3Kcx3CrjVf4yBmESU/edit?ts=5b5f17db#gid=227859291</td><td>PHESANT Transformation:20003_0 | | CAT-MUL-BINARY-VAR 1140888646 | | Indicator name x137_0_0 | | Remove indicator var NAs: 53 | | Remove indicator var <0: 0 | | Removed 0 examples != 1140888646 but with missing value (<0) | | sample 358051/3090(361141) | | -Notes:Code for treatment Negative codes indicate free-text entry.-Variable type:binary</td></tr>
<tr><td>binary.20003_1140909674.txt</td><td>1710174270056F5forCTG.txt.gz</td><td>-0.1077</td><td>0.07769</td><td>-1.386</td><td>0.1657</td><td>0.005677</td><td>0.001761</td><td>1.002</td><td>0.01019</td><td>0.00723</td><td>0.007531</td><td>Treatment/medication code: cod liver oil capsule</td><td>FALSE</td><td></td><td></td><td></td><td>361141</td><td>21034</td><td>340107</td><td>UK Biobank</td><td>https://docs.google.com/spreadsheets/d/1kvPoupSzsSFBNSztMzl04xMoSC3Kcx3CrjVf4yBmESU/edit?ts=5b5f17db#gid=227859291</td><td>PHESANT Transformation:20003_0 | | CAT-MUL-BINARY-VAR 1140909674 | | Indicator name x137_0_0 | | Remove indicator var NAs: 53 | | Remove indicator var <0: 0 | | Removed 0 examples != 1140909674 but with missing value (<0) | | sample 340107/21034(361141) | | -Notes:Code for treatment Negative codes indicate free-text entry.-Variable type:binary</td></tr>
<tr><td>binary.20003_1140909708.txt</td><td>1710174270056F5forCTG.txt.gz</td><td>0.4087</td><td>0.1301</td><td>3.142</td><td>0.001679</td><td>0.003728</td><td>0.001653</td><td>1.014</td><td>0.009747</td><td>0.001759</td><td>0.008807</td><td>Treatment/medication code: furosemide</td><td>FALSE</td><td></td><td></td><td></td><td>361141</td><td>2714</td><td>358427</td><td>UK Biobank</td><td>https://docs.google.com/spreadsheets/d/1kvPoupSzsSFBNSztMzl04xMoSC3Kcx3CrjVf4yBmESU/edit?ts=5b5f17db#gid=227859291</td><td>PHESANT Transformation:20003_0 | | CAT-MUL-BINARY-VAR 1140909708 | | Indicator name x137_0_0 | | Remove indicator var NAs: 53 | | Remove indicator var <0: 0 | | Removed 0 examples != 1140909708 but with missing value (<0) | | sample 358427/2714(361141) | | -Notes:Code for treatment Negative codes indicate free-text entry.-Variable type:binary</td></tr>
<tr><td>binary.20003_1140909786.txt</td><td>1710174270056F5forCTG.txt.gz</td><td>0.1121</td><td>0.1007</td><td>1.114</td><td>0.2654</td><td>0.003936</td><td>0.001609</td><td>0.994</td><td>0.009511</td><td>0.009394</td><td>0.007851</td><td>Treatment/medication code: beclometasone</td><td>FALSE</td><td></td><td></td><td></td><td>361141</td><td>3299</td><td>357842</td><td>UK Biobank</td><td>https://docs.google.com/spreadsheets/d/1kvPoupSzsSFBNSztMzl04xMoSC3Kcx3CrjVf4yBmESU/edit?ts=5b5f17db#gid=227859291</td><td>PHESANT Transformation:20003_0 | | CAT-MUL-BINARY-VAR 1140909786 | | Indicator name x137_0_0 | | Remove indicator var NAs: 53 | | Remove indicator var <0: 0 | | Removed 0 examples != 1140909786 but with missing value (<0) | | sample 357842/3299(361141) | | -Notes:Code for treatment Negative codes indicate free-text entry.-Variable type:binary</td></tr>
<tr><td>binary.20003_1140909788.txt</td><td>1710174270056F5forCTG.txt.gz</td><td>0.09335</td><td>0.1115</td><td>0.8368</td><td>0.4027</td><td>0.002909</td><td>0.00152</td><td>0.984</td><td>0.01035</td><td>0.005994</td><td>0.008046</td><td>Treatment/medication code: sodium cromoglicate</td><td>FALSE</td><td></td><td></td><td></td><td>361141</td><td>310</td><td>360831</td><td>UK Biobank</td><td>https://docs.google.com/spreadsheets/d/1kvPoupSzsSFBNSztMzl04xMoSC3Kcx3CrjVf4yBmESU/edit?ts=5b5f17db#gid=227859291</td><td>PHESANT Transformation:20003_0 | | CAT-MUL-BINARY-VAR 1140909788 | | Indicator name x137_0_0 | | Remove indicator var NAs: 53 | | Remove indicator var <0: 0 | | Removed 0 examples != 1140909788 but with missing value (<0) | | sample 360831/310(361141) | | -Notes:Code for treatment Negative codes indicate free-text entry.-Variable type:binary</td></tr>
<tr><td>binary.20003_1140909790.txt</td><td>1710174270056F5forCTG.txt.gz</td><td>0.1929</td><td>0.1099</td><td>1.755</td><td>0.07918</td><td>0.00306</td><td>0.001415</td><td>0.994</td><td>0.009782</td><td>-0.001803</td><td>0.008903</td><td>Treatment/medication code: chlorphenamine</td><td>FALSE</td><td></td><td></td><td></td><td>361141</td><td>217</td><td>360924</td><td>UK Biobank</td><td>https://docs.google.com/spreadsheets/d/1kvPoupSzsSFBNSztMzl04xMoSC3Kcx3CrjVf4yBmESU/edit?ts=5b5f17db#gid=227859291</td><td>PHESANT Transformation:20003_0 | | CAT-MUL-BINARY-VAR 1140909790 | | Indicator name x137_0_0 | | Remove indicator var NAs: 53 | | Remove indicator var <0: 0 | | Removed 0 examples != 1140909790 but with missing value (<0) | | sample 360924/217(361141) | | -Notes:Code for treatment Negative codes indicate free-text entry.-Variable type:binary</td></tr>
<tr><td>binary.20003_1140910766.txt</td><td>1710174270056F5forCTG.txt.gz</td><td>0.3978</td><td>0.154</td><td>2.583</td><td>0.009792</td><td>0.00247</td><td>0.001377</td><td>1.014</td><td>0.009084</td><td>-0.00183</td><td>0.008717</td><td>Treatment/medication code: nicorandil</td><td>FALSE</td><td></td><td></td><td></td><td>361141</td><td>1152</td><td>359989</td><td>UK Biobank</td><td>https://docs.google.com/spreadsheets/d/1kvPoupSzsSFBNSztMzl04xMoSC3Kcx3CrjVf4yBmESU/edit?ts=5b5f17db#gid=227859291</td><td>PHESANT Transformation:20003_0 | | CAT-MUL-BINARY-VAR 1140910766 | | Indicator name x137_0_0 | | Remove indicator var NAs: 53 | | Remove indicator var <0: 0 | | Removed 0 examples != 1140910766 but with missing value (<0) | | sample 359989/1152(361141) | | -Notes:Code for treatment Negative codes indicate free-text entry.-Variable type:binary</td></tr>
<tr><td>binary.20003_1140911658.txt</td><td>1710174270056F5forCTG.txt.gz</td><td>-0.0161</td><td>0.07178</td><td>-0.2242</td><td>0.8226</td><td>0.00613</td><td>0.001642</td><td>0.98</td><td>0.009659</td><td>0.007037</td><td>0.007796</td><td>Treatment/medication code: imigran 50mg tablet</td><td>FALSE</td><td></td><td></td><td></td><td>361141</td><td>590</td><td>360551</td><td>UK Biobank</td><td>https://docs.google.com/spreadsheets/d/1kvPoupSzsSFBNSztMzl04xMoSC3Kcx3CrjVf4yBmESU/edit?ts=5b5f17db#gid=227859291</td><td>PHESANT Transformation:20003_0 | | CAT-MUL-BINARY-VAR 1140911658 | | Indicator name x137_0_0 | | Remove indicator var NAs: 53 | | Remove indicator var <0: 0 | | Removed 0 examples != 1140911658 but with missing value (<0) | | sample 360551/590(361141) | | -Notes:Code for treatment Negative codes indicate free-text entry.-Variable type:binary</td></tr>
<tr><td>binary.20003_1140911730.txt</td><td>1710174270056F5forCTG.txt.gz</td><td>-0.05828</td><td>0.1202</td><td>-0.485</td><td>0.6277</td><td>0.002714</td><td>0.001288</td><td>0.998</td><td>0.008751</td><td>0.01123</td><td>0.008717</td><td>Treatment/medication code: flax oil tablet</td><td>FALSE</td><td></td><td></td><td></td><td>361141</td><td>598</td><td>360543</td><td>UK Biobank</td><td>https://docs.google.com/spreadsheets/d/1kvPoupSzsSFBNSztMzl04xMoSC3Kcx3CrjVf4yBmESU/edit?ts=5b5f17db#gid=227859291</td><td>PHESANT Transformation:20003_0 | | CAT-MUL-BINARY-VAR 1140911730 | | Indicator name x137_0_0 | | Remove indicator var NAs: 53 | | Remove indicator var <0: 0 | | Removed 0 examples != 1140911730 but with missing value (<0) | | sample 360543/598(361141) | | -Notes:Code for treatment Negative codes indicate free-text entry.-Variable type:binary</td></tr>
<tr><td>binary.20003_1140911732.txt</td><td>1710174270056F5forCTG.txt.gz</td><td>0.04265</td><td>0.1113</td><td>0.3831</td><td>0.7016</td><td>0.00275</td><td>0.001406</td><td>0.991</td><td>0.009091</td><td>0.004398</td><td>0.007931</td><td>Treatment/medication code: garlic product</td><td>FALSE</td><td></td><td></td><td></td><td>361141</td><td>4869</td><td>356272</td><td>UK Biobank</td><td>https://docs.google.com/spreadsheets/d/1kvPoupSzsSFBNSztMzl04xMoSC3Kcx3CrjVf4yBmESU/edit?ts=5b5f17db#gid=227859291</td><td>PHESANT Transformation:20003_0 | | CAT-MUL-BINARY-VAR 1140911732 | | Indicator name x137_0_0 | | Remove indicator var NAs: 53 | | Remove indicator var <0: 0 | | Removed 0 examples != 1140911732 but with missing value (<0) | | sample 356272/4869(361141) | | -Notes:Code for treatment Negative codes indicate free-text entry.-Variable type:binary</td></tr>
<tr><td>binary.20003_1140916282.txt</td><td>1710174270056F5forCTG.txt.gz</td><td>0.1515</td><td>0.1094</td><td>1.385</td><td>0.1662</td><td>0.00349</td><td>0.001469</td><td>0.998</td><td>0.008685</td><td>0.009493</td><td>0.008315</td><td>Treatment/medication code: venlafaxine</td><td>FALSE</td><td></td><td></td><td></td><td>361141</td><td>1533</td><td>359608</td><td>UK Biobank</td><td>https://docs.google.com/spreadsheets/d/1kvPoupSzsSFBNSztMzl04xMoSC3Kcx3CrjVf4yBmESU/edit?ts=5b5f17db#gid=227859291</td><td>PHESANT Transformation:20003_0 | | CAT-MUL-BINARY-VAR 1140916282 | | Indicator name x137_0_0 | | Remove indicator var NAs: 53 | | Remove indicator var <0: 0 | | Removed 0 examples != 1140916282 but with missing value (<0) | | sample 359608/1533(361141) | | -Notes:Code for treatment Negative codes indicate free-text entry.-Variable type:binary</td></tr>
<tr><td>binary.20003_1140916356.txt</td><td>1710174270056F5forCTG.txt.gz</td><td>0.06848</td><td>0.06489</td><td>1.055</td><td>0.2913</td><td>0.009184</td><td>0.001806</td><td>0.988</td><td>0.01103</td><td>-0.000445</td><td>0.008298</td><td>Treatment/medication code: losartan</td><td>FALSE</td><td></td><td></td><td></td><td>361141</td><td>3268</td><td>357873</td><td>UK Biobank</td><td>https://docs.google.com/spreadsheets/d/1kvPoupSzsSFBNSztMzl04xMoSC3Kcx3CrjVf4yBmESU/edit?ts=5b5f17db#gid=227859291</td><td>PHESANT Transformation:20003_0 | | CAT-MUL-BINARY-VAR 1140916356 | | Indicator name x137_0_0 | | Remove indicator var NAs: 53 | | Remove indicator var <0: 0 | | Removed 0 examples != 1140916356 but with missing value (<0) | | sample 357873/3268(361141) | | -Notes:Code for treatment Negative codes indicate free-text entry.-Variable type:binary</td></tr>
<tr><td>binary.20003_1140921600.txt</td><td>1710174270056F5forCTG.txt.gz</td><td>0.2598</td><td>0.1196</td><td>2.172</td><td>0.02989</td><td>0.00367</td><td>0.001717</td><td>1.012</td><td>0.009873</td><td>0.001527</td><td>0.008956</td><td>Treatment/medication code: citalopram</td><td>FALSE</td><td></td><td></td><td></td><td>361141</td><td>6185</td><td>354956</td><td>UK Biobank</td><td>https://docs.google.com/spreadsheets/d/1kvPoupSzsSFBNSztMzl04xMoSC3Kcx3CrjVf4yBmESU/edit?ts=5b5f17db#gid=227859291</td><td>PHESANT Transformation:20003_0 | | CAT-MUL-BINARY-VAR 1140921600 | | Indicator name x137_0_0 | | Remove indicator var NAs: 53 | | Remove indicator var <0: 0 | | Removed 0 examples != 1140921600 but with missing value (<0) | | sample 354956/6185(361141) | | -Notes:Code for treatment Negative codes indicate free-text entry.-Variable type:binary</td></tr>
<tr><td>binary.20003_1140922174.txt</td><td>1710174270056F5forCTG.txt.gz</td><td>-0.0393</td><td>0.07803</td><td>-0.5037</td><td>0.6145</td><td>0.006019</td><td>0.001727</td><td>0.998</td><td>0.009904</td><td>-0.005819</td><td>0.007683</td><td>Treatment/medication code: alendronate sodium</td><td>FALSE</td><td></td><td></td><td></td><td>361141</td><td>4103</td><td>357038</td><td>UK Biobank</td><td>https://docs.google.com/spreadsheets/d/1kvPoupSzsSFBNSztMzl04xMoSC3Kcx3CrjVf4yBmESU/edit?ts=5b5f17db#gid=227859291</td><td>PHESANT Transformation:20003_0 | | CAT-MUL-BINARY-VAR 1140922174 | | Indicator name x137_0_0 | | Remove indicator var NAs: 53 | | Remove indicator var <0: 0 | | Removed 0 examples != 1140922174 but with missing value (<0) | | sample 357038/4103(361141) | | -Notes:Code for treatment Negative codes indicate free-text entry.-Variable type:binary</td></tr>
<tr><td>binary.20003_1140923346.txt</td><td>1710174270056F5forCTG.txt.gz</td><td>0.4278</td><td>0.07247</td><td>5.903</td><td>3.57E-09</td><td>0.009557</td><td>0.001635</td><td>1.015</td><td>0.01036</td><td>0.01302</td><td>0.008558</td><td>Treatment/medication code: co-codamol</td><td>FALSE</td><td>Pain</td><td></td><td></td><td>361141</td><td>8384</td><td>352757</td><td>UK Biobank</td><td>https://docs.google.com/spreadsheets/d/1kvPoupSzsSFBNSztMzl04xMoSC3Kcx3CrjVf4yBmESU/edit?ts=5b5f17db#gid=227859291</td><td>PHESANT Transformation:20003_0 | | CAT-MUL-BINARY-VAR 1140923346 | | Indicator name x137_0_0 | | Remove indicator var NAs: 53 | | Remove indicator var <0: 0 | | Removed 0 examples != 1140923346 but with missing value (<0) | | sample 352757/8384(361141) | | -Notes:Code for treatment Negative codes indicate free-text entry.-Variable type:binary</td></tr>
<tr><td>binary.20003_1140925800.txt</td><td>1710174270056F5forCTG.txt.gz</td><td>0.2432</td><td>0.1071</td><td>2.27</td><td>0.02318</td><td>0.00348</td><td>0.001537</td><td>0.989</td><td>0.00952</td><td>-0.004506</td><td>0.007907</td><td>Treatment/medication code: movicol oral powder</td><td>FALSE</td><td></td><td></td><td></td><td>361141</td><td>1131</td><td>360010</td><td>UK Biobank</td><td>https://docs.google.com/spreadsheets/d/1kvPoupSzsSFBNSztMzl04xMoSC3Kcx3CrjVf4yBmESU/edit?ts=5b5f17db#gid=227859291</td><td>PHESANT Transformation:20003_0 | | CAT-MUL-BINARY-VAR 1140925800 | | Indicator name x137_0_0 | | Remove indicator var NAs: 53 | | Remove indicator var <0: 0 | | Removed 0 examples != 1140925800 but with missing value (<0) | | sample 360010/1131(361141) | | -Notes:Code for treatment Negative codes indicate free-text entry.-Variable type:binary</td></tr>
</table>

binary.20003_1140926606.txt	1710174270056F5forCTG.txt.gz	0.1993	0.08679	2.297	0.02165	0.005144	0.00163	1.002	0.009959	0.01407	0.008231	Treatment/medication code: salbutamol 100micrograms spacehaler	FALSE				361141	2267	358874	UK Biobank	https://docs.google.com/spreadsheets/d/1kvPoupSzsSFBNSztMzl04xMoSC3Kcx3CrjVf4yBmESU/edit?ts=5b5f17db#gid=227859291	PHESANT Transformation:20003_0 | | CAT-MUL-BINARY-VAR 1140926606 | | Indicator name x137_0_0 | | Remove indicator var NAs: 53 | | Remove indicator var <0: 0 | | Removed 0 examples != 1140926606 but with missing value (<0) | | sample 358874/2267(361141) | |-Notes:Code for treatment Negative codes indicate free-text entry.-Variable type:binary
binary.20003_1140927086.txt	1710174270056F5forCTG.txt.gz	0.1676	0.1411	1.188	0.2348	0.002199	0.00151	0.999	0.009342	0.001546	0.008167	Treatment/medication code: arthrotec 50 tablet	FALSE				361141	308	360833	UK Biobank	https://docs.google.com/spreadsheets/d/1kvPoupSzsSFBNSztMzl04xMoSC3Kcx3CrjVf4yBmESU/edit?ts=5b5f17db#gid=227859291	PHESANT Transformation:20003_0 | | CAT-MUL-BINARY-VAR 1140927086 | | Indicator name x137_0_0 | | Remove indicator var NAs: 53 | | Remove indicator var <0: 0 | | Removed 0 examples != 1140927086 but with missing value (<0) | | sample 360833/308(361141) | |-Notes:Code for treatment Negative codes indicate free-text entry.-Variable type:binary
binary.20003_1140927328.txt	1710174270056F5forCTG.txt.gz	-0.1168	0.0995	-1.174	0.2404	0.003557	0.001497	0.983	0.008495	0.006868	0.007663	Treatment/medication code: terbinafine	FALSE				361141	700	360441	UK Biobank	https://docs.google.com/spreadsheets/d/1kvPoupSzsSFBNSztMzl04xMoSC3Kcx3CrjVf4yBmESU/edit?ts=5b5f17db#gid=227859291	PHESANT Transformation:20003_0 | | CAT-MUL-BINARY-VAR 1140927328 | | Indicator name x137_0_0 | | Remove indicator var NAs: 53 | | Remove indicator var <0: 0 | | Removed 0 examples != 1140927328 but with missing value (<0) | | sample 360441/700(361141) | |-Notes:Code for treatment Negative codes indicate free-text entry.-Variable type:binary
binary.20003_1140929012.txt	1710174270056F5forCTG.txt.gz	0.3022	0.1074	2.812	0.004918	0.003267	0.001333	0.983	0.008418	-0.01136	0.007949	Treatment/medication code: pantoprazole	FALSE				361141	659	360482	UK Biobank	https://docs.google.com/spreadsheets/d/1kvPoupSzsSFBNSztMzl04xMoSC3Kcx3CrjVf4yBmESU/edit?ts=5b5f17db#gid=227859291	PHESANT Transformation:20003_0 | | CAT-MUL-BINARY-VAR 1140929012 | | Indicator name x137_0_0 | | Remove indicator var NAs: 53 | | Remove indicator var <0: 0 | | Removed 0 examples != 1140929012 but with missing value (<0) | | sample 360482/659(361141) | |-Notes:Code for treatment Negative codes indicate free-text entry.-Variable type:binary
binary.20003_1141145660.txt	1710174270056F5forCTG.txt.gz	-0.03016	0.08524	-0.3538	0.7235	0.005058	0.001497	0.99	0.009753	0.006613	0.008539	Treatment/medication code: valsartan	FALSE				361141	1707	359434	UK Biobank	https://docs.google.com/spreadsheets/d/1kvPoupSzsSFBNSztMzl04xMoSC3Kcx3CrjVf4yBmESU/edit?ts=5b5f17db#gid=227859291	PHESANT Transformation:20003_0 | | CAT-MUL-BINARY-VAR 1141145660 | | Indicator name x137_0_0 | | Remove indicator var NAs: 53 | | Remove indicator var <0: 0 | | Removed 0 examples != 1141145660 but with missing value (<0) | | sample 359434/1707(361141) | |-Notes:Code for treatment Negative codes indicate free-text entry.-Variable type:binary
binary.20003_1141146188.txt	1710174270056F5forCTG.txt.gz	-0.025	0.08523	-0.2933	0.7693	0.004403	0.001561	0.996	0.009684	0.005151	0.008299	Treatment/medication code: latanoprost	FALSE				361141	725	360416	UK Biobank	https://docs.google.com/spreadsheets/d/1kvPoupSzsSFBNSztMzl04xMoSC3Kcx3CrjVf4yBmESU/edit?ts=5b5f17db#gid=227859291	PHESANT Transformation:20003_0 | | CAT-MUL-BINARY-VAR 1141146188 | | Indicator name x137_0_0 | | Remove indicator var NAs: 53 | | Remove indicator var <0: 0 | | Removed 0 examples != 1141146188 but with missing value (<0) | | sample 360416/725(361141) | |-Notes:Code for treatment Negative codes indicate free-text entry.-Variable type:binary
binary.20003_1141146198.txt	1710174270056F5forCTG.txt.gz	-0.09694	0.07068	-1.372	0.1702	0.006823	0.001804	1	0.01032	0.001572	0.008514	Treatment/medication code: xalatan 0.005% eye drops	FALSE				361141	1034	360107	UK Biobank	https://docs.google.com/spreadsheets/d/1kvPoupSzsSFBNSztMzl04xMoSC3Kcx3CrjVf4yBmESU/edit?ts=5b5f17db#gid=227859291	PHESANT Transformation:20003_0 | | CAT-MUL-BINARY-VAR 1141146198 | | Indicator name x137_0_0 | | Remove indicator var NAs: 53 | | Remove indicator var <0: 0 | | Removed 0 examples != 1141146198 but with missing value (<0) | | sample 360107/1034(361141) | |-Notes:Code for treatment Negative codes indicate free-text entry.-Variable type:binary
binary.20003_1141146234.txt	1710174270056F5forCTG.txt.gz	0.2012	0.06015	3.345	0.000823	0.01321	0.002822	1.03	0.01916	0.005309	0.008547	Treatment/medication code: atorvastatin	FALSE				361141	10805	350336	UK Biobank	https://docs.google.com/spreadsheets/d/1kvPoupSzsSFBNSztMzl04xMoSC3Kcx3CrjVf4yBmESU/edit?ts=5b5f17db#gid=227859291	PHESANT Transformation:20003_0 | | CAT-MUL-BINARY-VAR 1141146234 | | Indicator name x137_0_0 | | Remove indicator var NAs: 53 | | Remove indicator var <0: 0 | | Removed 0 examples != 1141146234 but with missing value (<0) | | sample 350336/10805(361141) | |-Notes:Code for treatment Negative codes indicate free-text entry.-Variable type:binary
binary.20003_1141156836.txt	1710174270056F5forCTG.txt.gz	0.1211	0.0684	1.77	0.07674	0.007294	0.001746	0.993	0.01007	0.003903	0.007196	Treatment/medication code: candesartan cilexetil	FALSE				361141	3875	357266	UK Biobank	https://docs.google.com/spreadsheets/d/1kvPoupSzsSFBNSztMzl04xMoSC3Kcx3CrjVf4yBmESU/edit?ts=5b5f17db#gid=227859291	PHESANT Transformation:20003_0 | | CAT-MUL-BINARY-VAR 1141156836 | | Indicator name x137_0_0 | | Remove indicator var NAs: 53 | | Remove indicator var <0: 0 | | Removed 0 examples != 1141156836 but with missing value (<0) | | sample 357266/3875(361141) | |-Notes:Code for treatment Negative codes indicate free-text entry.-Variable type:binary
binary.20003_1141157264.txt	1710174270056F5forCTG.txt.gz	0.2443	0.15	1.629	0.1033	0.002541	0.001571	1.002	0.01022	-0.002252	0.008441	Treatment/medication code: salmeterol product	FALSE				361141	224	360917	UK Biobank	https://docs.google.com/spreadsheets/d/1kvPoupSzsSFBNSztMzl04xMoSC3Kcx3CrjVf4yBmESU/edit?ts=5b5f17db#gid=227859291	PHESANT Transformation:20003_0 | | CAT-MUL-BINARY-VAR 1141157264 | | Indicator name x137_0_0 | | Remove indicator var NAs: 53 | | Remove indicator var <0: 0 | | Removed 0 examples != 1141157264 but with missing value (<0) | | sample 360917/224(361141) | |-Notes:Code for treatment Negative codes indicate free-text entry.-Variable type:binary
binary.20003_1141164828.txt	1710174270056F5forCTG.txt.gz	-0.148	0.113	-1.309	0.1905	0.003012	0.001613	1.002	0.009051	0.009515	0.007464	Treatment/medication code: adcal-d3 1.5g/10micrograms chewable tablet	FALSE				361141	2435	358706	UK Biobank	https://docs.google.com/spreadsheets/d/1kvPoupSzsSFBNSztMzl04xMoSC3Kcx3CrjVf4yBmESU/edit?ts=5b5f17db#gid=227859291	PHESANT Transformation:20003_0 | | CAT-MUL-BINARY-VAR 1141164828 | | Indicator name x137_0_0 | | Remove indicator var NAs: 53 | | Remove indicator var <0: 0 | | Removed 0 examples != 1141164828 but with missing value (<0) | | sample 358706/2435(361141) | |-Notes:Code for treatment Negative codes indicate free-text entry.-Variable type:binary
binary.20003_1141166006.txt	1710174270056F5forCTG.txt.gz	0.05716	0.1003	0.57	0.5687	0.003492	0.00164	0.993	0.01	0.001974	0.008264	Treatment/medication code: telmisartan	FALSE				361141	582	360559	UK Biobank	https://docs.google.com/spreadsheets/d/1kvPoupSzsSFBNSztMzl04xMoSC3Kcx3CrjVf4yBmESU/edit?ts=5b5f17db#gid=227859291	PHESANT Transformation:20003_0 | | CAT-MUL-BINARY-VAR 1141166006 | | Indicator name x137_0_0 | | Remove indicator var NAs: 53 | | Remove indicator var <0: 0 | | Removed 0 examples != 1141166006 but with missing value (<0) | | sample 360559/582(361141) | |-Notes:Code for treatment Negative codes indicate free-text entry.-Variable type:binary
binary.20003_1141168318.txt	1710174270056F5forCTG.txt.gz	0.3065	0.103	2.975	0.002933	0.004914	0.001757	0.996	0.009655	-0.009701	0.008223	Treatment/medication code: clopidogrel	FALSE				361141	2290	358851	UK Biobank	https://docs.google.com/spreadsheets/d/1kvPoupSzsSFBNSztMzl04xMoSC3Kcx3CrjVf4yBmESU/edit?ts=5b5f17db#gid=227859291	PHESANT Transformation:20003_0 | | CAT-MUL-BINARY-VAR 1141168318 | | Indicator name x137_0_0 | | Remove indicator var NAs: 53 | | Remove indicator var <0: 0 | | Removed 0 examples != 1141168318 but with missing value (<0) | | sample 358851/2290(361141) | |-Notes:Code for treatment Negative codes indicate free-text entry.-Variable type:binary
binary.20003_1141171646.txt	1710174270056F5forCTG.txt.gz	0.1578	0.07915	1.993	0.04621	0.005773	0.001517	0.981	0.008743	0.00314	0.007939	Treatment/medication code: pioglitazone	FALSE				361141	744	360397	UK Biobank	https://docs.google.com/spreadsheets/d/1kvPoupSzsSFBNSztMzl04xMoSC3Kcx3CrjVf4yBmESU/edit?ts=5b5f17db#gid=227859291	PHESANT Transformation:20003_0 | | CAT-MUL-BINARY-VAR 1141171646 | | Indicator name x137_0_0 | | Remove indicator var NAs: 53 | | Remove indicator var <0: 0 | | Removed 0 examples != 1141171646 but with missing value (<0) | | sample 360397/744(361141) | |-Notes:Code for treatment Negative codes indicate free-text entry.-Variable type:binary
binary.20003_1141174508.txt	1710174270056F5forCTG.txt.gz	0.2706	0.1179	2.294	0.02178	0.003463	0.001614	0.988	0.0108	-0.01121	0.009276	Treatment/medication code: reductil 10mg capsule	FALSE				361141	116	361025	UK Biobank	https://docs.google.com/spreadsheets/d/1kvPoupSzsSFBNSztMzl04xMoSC3Kcx3CrjVf4yBmESU/edit?ts=5b5f17db#gid=227859291	PHESANT Transformation:20003_0 | | CAT-MUL-BINARY-VAR 1141174508 | | Indicator name x137_0_0 | | Remove indicator var NAs: 53 | | Remove indicator var <0: 0 | | Removed 0 examples != 1141174508 but with missing value (<0) | | sample 361025/116(361141) | |-Notes:Code for treatment Negative codes indicate free-text entry.-Variable type:binary
binary.20003_1141174520.txt	1710174270056F5forCTG.txt.gz	0.335	0.1252	2.675	0.007479	0.00328	0.001683	1.013	0.009671	-0.01107	0.007666	Treatment/medication code: symbicort 100/6 turbohaler	FALSE				361141	2249	358892	UK Biobank	https://docs.google.com/spreadsheets/d/1kvPoupSzsSFBNSztMzl04xMoSC3Kcx3CrjVf4yBmESU/edit?ts=5b5f17db#gid=227859291	PHESANT Transformation:20003_0 | | CAT-MUL-BINARY-VAR 1141174520 | | Indicator name x137_0_0 | | Remove indicator var NAs: 53 | | Remove indicator var <0: 0 | | Removed 0 examples != 1141174520 but with missing value (<0) | | sample 358892/2249(361141) | |-Notes:Code for treatment Negative codes indicate free-text entry.-Variable type:binary
binary.20003_1141175684.txt	1710174270056F5forCTG.txt.gz	-0.1907	0.1054	-1.809	0.07046	0.003767	0.001557	0.983	0.009255	0.01492	0.007881	Treatment/medication code: risedronate sodium	FALSE				361141	718	360423	UK Biobank	https://docs.google.com/spreadsheets/d/1kvPoupSzsSFBNSztMzl04xMoSC3Kcx3CrjVf4yBmESU/edit?ts=5b5f17db#gid=227859291	PHESANT Transformation:20003_0 | | CAT-MUL-BINARY-VAR 1141175684 | | Indicator name x137_0_0 | | Remove indicator var NAs: 53 | | Remove indicator var <0: 0 | | Removed 0 examples != 1141175684 but with missing value (<0) | | sample 360423/718(361141) | |-Notes:Code for treatment Negative codes indicate free-text entry.-Variable type:binary
binary.20003_1141176832.txt	1710174270056F5forCTG.txt.gz	0.1426	0.05927	2.406	0.01612	0.01191	0.002074	0.989	0.01101	0.00794	0.00788	Treatment/medication code: seretide 50 evohaler	FALSE				361141	4269	356872	UK Biobank	https://docs.google.com/spreadsheets/d/1kvPoupSzsSFBNSztMzl04xMoSC3Kcx3CrjVf4yBmESU/edit?ts=5b5f17db#gid=227859291	PHESANT Transformation:20003_0 | | CAT-MUL-BINARY-VAR 1141176832 | | Indicator name x137_0_0 | | Remove indicator var NAs: 53 | | Remove indicator var <0: 0 | | Removed 0 examples != 1141176832 but with missing value (<0) | | sample 356872/4269(361141) | |-Notes:Code for treatment Negative codes indicate free-text entry.-Variable type:binary
binary.20003_1141177600.txt	1710174270056F5forCTG.txt.gz	0.1994	0.1104	1.806	0.07087	0.003436	0.001372	1	0.009616	0.005318	0.008217	Treatment/medication code: rosiglitazone	FALSE				361141	531	360610	UK Biobank	https://docs.google.com/spreadsheets/d/1kvPoupSzsSFBNSztMzl04xMoSC3Kcx3CrjVf4yBmESU/edit?ts=5b5f17db#gid=227859291	PHESANT Transformation:20003_0 | | CAT-MUL-BINARY-VAR 1141177600 | | Indicator name x137_0_0 | | Remove indicator var NAs: 53 | | Remove indicator var <0: 0 | | Removed 0 examples != 1141177600 but with missing value (<0) | | sample 360610/531(361141) | |-Notes:Code for treatment Negative codes indicate free-text entry.-Variable type:binary
binary.20003_1141182632.txt	1710174270056F5forCTG.txt.gz	0.3026	0.1021	2.963	0.003048	0.004257	0.001467	0.994	0.009183	-0.007017	0.00772	Treatment/medication code: spiriva 18micrograms inhalation capsule	FALSE				361141	539	360602	UK Biobank	https://docs.google.com/spreadsheets/d/1kvPoupSzsSFBNSztMzl04xMoSC3Kcx3CrjVf4yBmESU/edit?ts=5b5f17db#gid=227859291	PHESANT Transformation:20003_0 | | CAT-MUL-BINARY-VAR 1141182632 | | Indicator name x137_0_0 | | Remove indicator var NAs: 53 | | Remove indicator var <0: 0 | | Removed 0 examples != 1141182632 but with missing value (<0) | | sample 360602/539(361141) | |-Notes:Code for treatment Negative codes indicate free-text entry.-Variable type:binary
binary.20003_1141184726.txt	1710174270056F5forCTG.txt.gz	-0.005682	0.1206	-0.0471	0.9624	0.002859	0.001574	0.998	0.009373	0.0009984	0.008288	Treatment/medication code: xalacom 0.005%/0.5% eye drops	FALSE				361141	210	360931	UK Biobank	https://docs.google.com/spreadsheets/d/1kvPoupSzsSFBNSztMzl04xMoSC3Kcx3CrjVf4yBmESU/edit?ts=5b5f17db#gid=227859291	PHESANT Transformation:20003_0 | | CAT-MUL-BINARY-VAR 1141184726 | | Indicator name x137_0_0 | | Remove indicator var NAs: 53 | | Remove indicator var <0: 0 | | Removed 0 examples != 1141184726 but with missing value (<0) | | sample 360931/210(361141) | |-Notes:Code for treatment Negative codes indicate free-text entry.-Variable type:binary
binary.20003_1141188442.txt	1710174270056F5forCTG.txt.gz	0.05754	0.07604	0.7568	0.4492	0.006878	0.001564	1.007	0.009021	-0.005847	0.008077	Treatment/medication code: glucosamine product	FALSE				361141	24213	336928	UK Biobank	https://docs.google.com/spreadsheets/d/1kvPoupSzsSFBNSztMzl04xMoSC3Kcx3CrjVf4yBmESU/edit?ts=5b5f17db#gid=227859291	PHESANT Transformation:20003_0 | | CAT-MUL-BINARY-VAR 1141188442 | | Indicator name x137_0_0 | | Remove indicator var NAs: 53 | | Remove indicator var <0: 0 | | Removed 0 examples != 1141188442 but with missing value (<0) | | sample 336928/24213(361141) | |-Notes:Code for treatment Negative codes indicate free-text entry.-Variable type:binary
binary.20003_1141191044.txt	1710174270056F5forCTG.txt.gz	0.01249	0.04221	0.2958	0.7673	0.03396	0.004866	1.037	0.01876	0.02595	0.008949	Treatment/medication code: levothyroxine sodium	FALSE				361141	14689	346452	UK Biobank	https://docs.google.com/spreadsheets/d/1kvPoupSzsSFBNSztMzl04xMoSC3Kcx3CrjVf4yBmESU/edit?ts=5b5f17db#gid=227859291	PHESANT Transformation:20003_0 | | CAT-MUL-BINARY-VAR 1141191044 | | Indicator name x137_0_0 | | Remove indicator var NAs: 53 | | Remove indicator var <0: 0 | | Removed 0 examples != 1141191044 but with missing value (<0) | | sample 346452/14689(361141) | |-Notes:Code for treatment Negative codes indicate free-text entry.-Variable type:binary

<table>
<tr><th></th><th></th><th></th><th></th><th></th><th></th><th></th><th></th><th></th><th></th><th></th><th></th><th></th><th></th><th></th><th></th><th></th><th></th><th></th><th></th><th></th><th></th><th></th></tr>
<tr><td>binary.20003_1141192410.txt</td><td>1710174270056F5forCTG.txt.gz</td><td>0.1492</td><td>0.09977</td><td>1.495</td><td>0.1349</td><td>0.003181</td><td>0.001567</td><td>1.011</td><td>0.009798</td><td>0.004739</td><td>0.008391</td><td>Treatment/medication code: rosuvastatin</td><td>FALSE</td><td></td><td></td><td></td><td>361141</td><td>2227</td><td>358914</td><td>UK Biobank</td><td>https://docs.google.com/spreadsheets/d/1kvPoupSzsSFBNSztMzl04xMoSC3Kcx3CrjVf4yBmESU/edit?ts=5b5f17db#gid=227859291</td><td>PHESANT Transformation:20003_0 || CAT-MUL-BINARY-VAR 1141192410 || Indicator name x137_0_0 || Remove indicator var NAs: 53 || Remove indicator var <0: 0 || Removed 0 examples != 1141192410 but with missing value (<0) || sample 358914/2227(361141) ||-Notes:Code for treatment Negative codes indicate free-text entry.-Variable type:binary</td></tr>
<tr><td>binary.20003_1141192736.txt</td><td>1710174270056F5forCTG.txt.gz</td><td>0.2545</td><td>0.08776</td><td>2.9</td><td>0.003734</td><td>0.005902</td><td>0.001658</td><td>1.002</td><td>0.01014</td><td>-0.008541</td><td>0.008108</td><td>Treatment/medication code: ezetimibe</td><td>FALSE</td><td></td><td></td><td></td><td>361141</td><td>2132</td><td>359009</td><td>UK Biobank</td><td>https://docs.google.com/spreadsheets/d/1kvPoupSzsSFBNSztMzl04xMoSC3Kcx3CrjVf4yBmESU/edit?ts=5b5f17db#gid=227859291</td><td>PHESANT Transformation:20003_0 || CAT-MUL-BINARY-VAR 1141192736 || Indicator name x137_0_0 || Remove indicator var NAs: 53 || Remove indicator var <0: 0 || Removed 0 examples != 1141192736 but with missing value (<0) || sample 359009/2132(361141) ||-Notes:Code for treatment Negative codes indicate free-text entry.-Variable type:binary</td></tr>
<tr><td>binary.20003_1141194794.txt</td><td>1710174270056F5forCTG.txt.gz</td><td>0.1122</td><td>0.04386</td><td>2.559</td><td>0.0105</td><td>0.02818</td><td>0.002829</td><td>1.038</td><td>0.01488</td><td>-0.002613</td><td>0.008943</td><td>Treatment/medication code: bendroflumethiazide</td><td>FALSE</td><td></td><td></td><td></td><td>361141</td><td>20196</td><td>340945</td><td>UK Biobank</td><td>https://docs.google.com/spreadsheets/d/1kvPoupSzsSFBNSztMzl04xMoSC3Kcx3CrjVf4yBmESU/edit?ts=5b5f17db#gid=227859291</td><td>PHESANT Transformation:20003_0 || CAT-MUL-BINARY-VAR 1141194794 || Indicator name x137_0_0 || Remove indicator var NAs: 53 || Remove indicator var <0: 0 || Removed 0 examples != 1141194794 but with missing value (<0) || sample 340945/20196(361141) ||-Notes:Code for treatment Negative codes indicate free-text entry.-Variable type:binary</td></tr>
<tr><td>binary.20003_1187.txt</td><td>1710174270056F5forCTG.txt.gz</td><td>0.02934</td><td>0.09834</td><td>0.2984</td><td>0.7654</td><td>0.003341</td><td>0.001477</td><td>0.994</td><td>0.008426</td><td>-0.004395</td><td>0.007989</td><td>Treatment/medication code: chondroitin product</td><td>FALSE</td><td></td><td></td><td></td><td>361141</td><td>4663</td><td>356478</td><td>UK Biobank</td><td>https://docs.google.com/spreadsheets/d/1kvPoupSzsSFBNSztMzl04xMoSC3Kcx3CrjVf4yBmESU/edit?ts=5b5f17db#gid=227859291</td><td>PHESANT Transformation:20003_0 || CAT-MUL-BINARY-VAR 1187 || Indicator name x137_0_0 || Remove indicator var NAs: 53 || Remove indicator var <0: 0 || Removed 0 examples != 1187 but with missing value (<0) || sample 356478/4663(361141) ||-Notes:Code for treatment Negative codes indicate free-text entry.-Variable type:binary</td></tr>
<tr><td>binary.20003_1193.txt</td><td>1710174270056F5forCTG.txt.gz</td><td>0.2544</td><td>0.1297</td><td>1.962</td><td>0.0498</td><td>0.00284</td><td>0.001466</td><td>1.015</td><td>0.009293</td><td>-0.009845</td><td>0.007811</td><td>Treatment/medication code: omega-3/fish oil supplement</td><td>FALSE</td><td></td><td></td><td></td><td>361141</td><td>13689</td><td>347452</td><td>UK Biobank</td><td>https://docs.google.com/spreadsheets/d/1kvPoupSzsSFBNSztMzl04xMoSC3Kcx3CrjVf4yBmESU/edit?ts=5b5f17db#gid=227859291</td><td>PHESANT Transformation:20003_0 || CAT-MUL-BINARY-VAR 1193 || Indicator name x137_0_0 || Remove indicator var NAs: 53 || Remove indicator var <0: 0 || Removed 0 examples != 1193 but with missing value (<0) || sample 347452/13689(361141) ||-Notes:Code for treatment Negative codes indicate free-text entry.-Variable type:binary</td></tr>
<tr><td>binary.20003_2038459814.txt</td><td>1710174270056F5forCTG.txt.gz</td><td>0.08039</td><td>0.1191</td><td>0.6751</td><td>0.4996</td><td>0.002383</td><td>0.001513</td><td>1.005</td><td>0.0101</td><td>0.0003008</td><td>0.00751</td><td>Treatment/medication code: digoxin</td><td>FALSE</td><td></td><td></td><td></td><td>361141</td><td>921</td><td>360220</td><td>UK Biobank</td><td>https://docs.google.com/spreadsheets/d/1kvPoupSzsSFBNSztMzl04xMoSC3Kcx3CrjVf4yBmESU/edit?ts=5b5f17db#gid=227859291</td><td>PHESANT Transformation:20003_0 || CAT-MUL-BINARY-VAR 2038459814 || Indicator name x137_0_0 || Remove indicator var NAs: 53 || Remove indicator var <0: 0 || Removed 0 examples != 2038459814 but with missing value (<0) || sample 360220/921(361141) ||-Notes:Code for treatment Negative codes indicate free-text entry.-Variable type:binary</td></tr>
<tr><td>binary.20003_2038460150.txt</td><td>1710174270056F5forCTG.txt.gz</td><td>0.3148</td><td>0.03731</td><td>8.438</td><td>3.24E-17</td><td>0.02975</td><td>0.002243</td><td>1.033</td><td>0.01091</td><td>0.01045</td><td>0.008121</td><td>Treatment/medication code: paracetamol</td><td>FALSE</td><td>Pain</td><td></td><td></td><td>361141</td><td>66347</td><td>294794</td><td>UK Biobank</td><td>https://docs.google.com/spreadsheets/d/1kvPoupSzsSFBNSztMzl04xMoSC3Kcx3CrjVf4yBmESU/edit?ts=5b5f17db#gid=227859291</td><td>PHESANT Transformation:20003_0 || CAT-MUL-BINARY-VAR 2038460150 || Indicator name x137_0_0 || Remove indicator var NAs: 53 || Remove indicator var <0: 0 || Removed 0 examples != 2038460150 but with missing value (<0) || sample 294794/66347(361141) ||-Notes:Code for treatment Negative codes indicate free-text entry.-Variable type:binary</td></tr>
<tr><td>binary.20003_99999.txt</td><td>1710174270056F5forCTG.txt.gz</td><td>0.3674</td><td>0.2022</td><td>1.817</td><td>0.06924</td><td>0.001879</td><td>0.001588</td><td>1.01</td><td>0.009154</td><td>0.007581</td><td>0.008132</td><td>Treatment/medication code: Free-text entry, unable to be coded</td><td>FALSE</td><td></td><td></td><td></td><td>361141</td><td>12249</td><td>348892</td><td>UK Biobank</td><td>https://docs.google.com/spreadsheets/d/1kvPoupSzsSFBNSztMzl04xMoSC3Kcx3CrjVf4yBmESU/edit?ts=5b5f17db#gid=227859291</td><td>PHESANT Transformation:20003_0 || CAT-MUL-BINARY-VAR 99999 || Indicator name x137_0_0 || Remove indicator var NAs: 53 || Remove indicator var <0: 0 || Removed 0 examples != 99999 but with missing value (<0) || sample 348892/12249(361141) ||-Notes:Code for treatment Negative codes indicate free-text entry.-Variable type:binary</td></tr>
<tr><td>binary.20084_472.txt</td><td>1710174270056F5forCTG.txt.gz</td><td>0.08346</td><td>0.1247</td><td>0.6693</td><td>0.5033</td><td>0.01788</td><td>0.01324</td><td>1.01</td><td>0.01055</td><td>-0.003312</td><td>0.008264</td><td>Vitamin and/or mineral supplement use: Fish oil</td><td>FALSE</td><td></td><td></td><td></td><td>51427</td><td>12462</td><td>38965</td><td>UK Biobank</td><td>https://docs.google.com/spreadsheets/d/1kvPoupSzsSFBNSztMzl04xMoSC3Kcx3CrjVf4yBmESU/edit?ts=5b5f17db#gid=227859291</td><td>PHESANT Transformation:20084_0 || CAT-MUL-BINARY-VAR 472 || Indicator name x20082_0_0 || Remove indicator var NAs: 309767 || Remove indicator var <0: 0 || Removed 0 examples != 472 but with missing value (<0) || sample 38965/12462(51427) ||-Notes:This field indicates the vitamin and/or mineral supplements that the participant indicated they consumed yesterday. The data is an amalgamation of 21 individual Yes/No answers which were asked if the participant indicated they consumed any supplements.-Variable type:binary</td></tr>
<tr><td>binary.20084_473.txt</td><td>1710174270056F5forCTG.txt.gz</td><td>0.02077</td><td>0.1312</td><td>0.1583</td><td>0.8742</td><td>0.01798</td><td>0.01049</td><td>0.996</td><td>0.009322</td><td>-0.0089</td><td>0.008495</td><td>Vitamin and/or mineral supplement use: Glucosamine/chondroitin</td><td>FALSE</td><td></td><td></td><td></td><td>51427</td><td>7922</td><td>43505</td><td>UK Biobank</td><td>https://docs.google.com/spreadsheets/d/1kvPoupSzsSFBNSztMzl04xMoSC3Kcx3CrjVf4yBmESU/edit?ts=5b5f17db#gid=227859291</td><td>PHESANT Transformation:20084_0 || CAT-MUL-BINARY-VAR 473 || Indicator name x20082_0_0 || Remove indicator var NAs: 309767 || Remove indicator var <0: 0 || Removed 0 examples != 473 but with missing value (<0) || sample 43505/7922(51427) ||-Notes:This field indicates the vitamin and/or mineral supplements that the participant indicated they consumed yesterday. The data is an amalgamation of 21 individual Yes/No answers which were asked if the participant indicated they consumed any supplements.-Variable type:binary</td></tr>
<tr><td>binary.20084_478.txt</td><td>1710174270056F5forCTG.txt.gz</td><td>-0.06601</td><td>0.1181</td><td>-0.5589</td><td>0.5762</td><td>0.02018</td><td>0.01153</td><td>0.994</td><td>0.009866</td><td>0.009915</td><td>0.007962</td><td>Vitamin and/or mineral supplement use: Vitamin C</td><td>FALSE</td><td></td><td></td><td></td><td>51427</td><td>3291</td><td>48136</td><td>UK Biobank</td><td>https://docs.google.com/spreadsheets/d/1kvPoupSzsSFBNSztMzl04xMoSC3Kcx3CrjVf4yBmESU/edit?ts=5b5f17db#gid=227859291</td><td>PHESANT Transformation:20084_0 || CAT-MUL-BINARY-VAR 478 || Indicator name x20082_0_0 || Remove indicator var NAs: 309767 || Remove indicator var <0: 0 || Removed 0 examples != 478 but with missing value (<0) || sample 48136/3291(51427) ||-Notes:This field indicates the vitamin and/or mineral supplements that the participant indicated they consumed yesterday. The data is an amalgamation of 21 individual Yes/No answers which were asked if the participant indicated they consumed any supplements.-Variable type:binary</td></tr>
<tr><td>binary.20084_480.txt</td><td>1710174270056F5forCTG.txt.gz</td><td>-0.1194</td><td>0.09335</td><td>-1.279</td><td>0.2008</td><td>0.02719</td><td>0.01049</td><td>0.985</td><td>0.008704</td><td>0.01234</td><td>0.007859</td><td>Vitamin and/or mineral supplement use: Vitamin E</td><td>FALSE</td><td></td><td></td><td></td><td>51427</td><td>961</td><td>50466</td><td>UK Biobank</td><td>https://docs.google.com/spreadsheets/d/1kvPoupSzsSFBNSztMzl04xMoSC3Kcx3CrjVf4yBmESU/edit?ts=5b5f17db#gid=227859291</td><td>PHESANT Transformation:20084_0 || CAT-MUL-BINARY-VAR 480 || Indicator name x20082_0_0 || Remove indicator var NAs: 309767 || Remove indicator var <0: 0 || Removed 0 examples != 480 but with missing value (<0) || sample 50466/961(51427) ||-Notes:This field indicates the vitamin and/or mineral supplements that the participant indicated they consumed yesterday. The data is an amalgamation of 21 individual Yes/No answers which were asked if the participant indicated they consumed any supplements.-Variable type:binary</td></tr>
<tr><td>binary.20086_10.txt</td><td>1710174270056F5forCTG.txt.gz</td><td>0.279</td><td>0.09042</td><td>3.086</td><td>0.002032</td><td>0.03339</td><td>0.01152</td><td>0.997</td><td>0.009917</td><td>-0.003897</td><td>0.007636</td><td>Type of special diet followed: Low calorie</td><td>FALSE</td><td></td><td></td><td></td><td>51427</td><td>6077</td><td>45350</td><td>UK Biobank</td><td>https://docs.google.com/spreadsheets/d/1kvPoupSzsSFBNSztMzl04xMoSC3Kcx3CrjVf4yBmESU/edit?ts=5b5f17db#gid=227859291</td><td>PHESANT Transformation:20086_0 || CAT-MUL-BINARY-VAR 10 || Indicator name x20082_0_0 || Remove indicator var NAs: 309767 || Remove indicator var <0: 0 || Removed 0 examples != 10 but with missing value (<0) || sample 45350/6077(51427) ||-Notes:This field indicates whether the participant routinely follows a special diet. The data is an amalgamation of 4 individual Yes/No answers. Some of the self-entered data regarding special diets is inconsistent with the actual food intake reported on the same occasion by participants. For instance, around a tenth of people indicating they followed a vegan diet also answered that they had eaten meat the prevous day.-Variable type:binary</td></tr>
<tr><td>binary.20086_13.txt</td><td>1710174270056F5forCTG.txt.gz</td><td>0.2886</td><td>0.1686</td><td>1.712</td><td>0.08697</td><td>0.01247</td><td>0.009817</td><td>1.001</td><td>0.00876</td><td>-0.004449</td><td>0.00733</td><td>Type of special diet followed: Other</td><td>FALSE</td><td></td><td></td><td></td><td>51427</td><td>1755</td><td>49672</td><td>UK Biobank</td><td>https://docs.google.com/spreadsheets/d/1kvPoupSzsSFBNSztMzl04xMoSC3Kcx3CrjVf4yBmESU/edit?ts=5b5f17db#gid=227859291</td><td>PHESANT Transformation:20086_0 || CAT-MUL-BINARY-VAR 13 || Indicator name x20082_0_0 || Remove indicator var NAs: 309767 || Remove indicator var <0: 0 || Removed 0 examples != 13 but with missing value (<0) || sample 49672/1755(51427) ||-Notes:This field indicates whether the participant routinely follows a special diet. The data is an amalgamation of 4 individual Yes/No answers. Some of the self-entered data regarding special diets is inconsistent with the actual food intake reported on the same occasion by participants. For instance, around a tenth of people indicating they followed a vegan diet also answered that they had eaten meat the prevous day.-Variable type:binary</td></tr>
<tr><td>binary.20090_353.txt</td><td>1710174270056F5forCTG.txt.gz</td><td>0.07734</td><td>0.1304</td><td>0.5932</td><td>0.553</td><td>0.01656</td><td>0.01116</td><td>1.023</td><td>0.009415</td><td>-0.01141</td><td>0.00812</td><td>Type of fat/oil used in cooking: Olive oil</td><td>FALSE</td><td></td><td></td><td></td><td>51427</td><td>18019</td><td>33408</td><td>UK Biobank</td><td>https://docs.google.com/spreadsheets/d/1kvPoupSzsSFBNSztMzl04xMoSC3Kcx3CrjVf4yBmESU/edit?ts=5b5f17db#gid=227859291</td><td>PHESANT Transformation:20090_0 || CAT-MUL-BINARY-VAR 353 || Indicator name x20082_0_0 || Remove indicator var NAs: 309767 || Remove indicator var <0: 0 || Removed 0 examples != 353 but with missing value (<0) || sample 33408/18019(51427) ||-Notes:This field indicates the type(s) of fats or oils the participant used when cooking yesterday. The data is an amalgamation of 16 individual Yes/No answers and 29 corresponding tick boxes.-Variable type:binary</td></tr>
<tr><td>binary.20090_371.txt</td><td>1710174270056F5forCTG.txt.gz</td><td>0.2084</td><td>0.1223</td><td>1.704</td><td>0.08836</td><td>0.02372</td><td>0.01127</td><td>0.99</td><td>0.008996</td><td>-0.01203</td><td>0.007416</td><td>Type of fat/oil used in cooking: Polyunsaturated margarine</td><td>FALSE</td><td></td><td></td><td></td><td>51427</td><td>4194</td><td>47233</td><td>UK Biobank</td><td>https://docs.google.com/spreadsheets/d/1kvPoupSzsSFBNSztMzl04xMoSC3Kcx3CrjVf4yBmESU/edit?ts=5b5f17db#gid=227859291</td><td>PHESANT Transformation:20090_0 || CAT-MUL-BINARY-VAR 371 || Indicator name x20082_0_0 || Remove indicator var NAs: 309767 || Remove indicator var <0: 0 || Removed 0 examples != 371 but with missing value (<0) || sample 47233/4194(51427) ||-Notes:This field indicates the type(s) of fats or oils the participant used when cooking yesterday. The data is an amalgamation of 16 individual Yes/No answers and 29 corresponding tick boxes.-Variable type:binary</td></tr>
<tr><td>binary.20096_1.txt</td><td>1710174270056F5forCTG.txt.gz</td><td>-0.06515</td><td>0.09406</td><td>-0.6927</td><td>0.4885</td><td>0.02659</td><td>0.01191</td><td>0.979</td><td>0.009919</td><td>-0.001098</td><td>0.007706</td><td>Size of red wine glass drunk: small (125ml)</td><td>FALSE</td><td></td><td></td><td></td><td>51427</td><td>3078</td><td>48349</td><td>UK Biobank</td><td>https://docs.google.com/spreadsheets/d/1kvPoupSzsSFBNSztMzl04xMoSC3Kcx3CrjVf4yBmESU/edit?ts=5b5f17db#gid=227859291</td><td>PHESANT Transformation:20096_0 || CAT-MUL-BINARY-VAR 1 || Indicator name x20082_0_0 || Remove indicator var NAs: 309767 || Remove indicator var <0: 0 || Removed 0 examples != 1 but with missing value (<0) || sample 48349/3078(51427) ||-Notes:This field indicates the size(s) of red wine glass the participant consumed yesterday. The data is an amalgamation of 3 individual tick boxes which were asked if the participant indicated they consumed red wine.-Variable type:binary</td></tr>
<tr><td>binary.2010.txt</td><td>1710174270056F5forCTG.txt.gz</td><td>0.0317</td><td>0.04024</td><td>0.7878</td><td>0.4308</td><td>0.04779</td><td>0.003207</td><td>0.972</td><td>0.01408</td><td>-0.01592</td><td>0.009908</td><td>Suffer from 'nerves'</td><td>FALSE</td><td></td><td></td><td></td><td>348082</td><td>73938</td><td>274144</td><td>UK Biobank</td><td>https://docs.google.com/spreadsheets/d/1kvPoupSzsSFBNSztMzl04xMoSC3Kcx3CrjVf4yBmESU/edit?ts=5b5f17db#gid=227859291</td><td>PHESANT Transformation:2010_0|| CAT-SINGLE || Inc(>=10): 0(274144) || Inc(>=10): 1(73938) || CAT-SINGLE-BINARY || sample 274144/73938(348082) ||-Notes:ACE touchscreen question Do you suffer from 'nerves'? If the participant activated the Help button they were shown the message: Work through these questions quickly and do not think about the exact meaning of the question-Variable type:binary</td></tr>
</table>

<table>
<tr><td>binary.20107_1.txt</td><td>1710174270056F5forCTG.txt.gz</td><td>0.08258</td><td>0.04239</td><td>1.948</td><td>0.05139</td><td>0.0277</td><td>0.003127</td><td>1.007</td><td>0.0129</td><td>-0.001597</td><td>0.008607</td><td>Illnesses of father: Heart disease</td><td>FALSE</td><td></td><td></td><td></td><td>318570</td><td>104110</td><td>214460</td><td>UK Biobank</td><td>https://docs.google.com/spreadsheets/d/1kvPoupSzsSFBNSztMzl04xMoSC3Kcx3CrjVf4yBmESU/edit?ts=5b5f17db#gid=227859291</td><td>PHESANT Transformation:20107_0 || CAT-MUL-BINARY-VAR 1 || NO_NAN Remove NA participants 9656 || Removed 32968 examples != 1 but with missing value (<0) || sample 214460/104110(318570) ||-Notes:This field contains the combined results of 2 ACE touchscreen questions, both asked Has/did your father ever suffer from? (You can select more than one answer) for different sets of illnesses. For convenience, the illnesses were arbitrarily divided into two sets: Group 1 : Heart disease, Stroke, High blood pressure, Chronic bronchitis/emphysema, Alzheimer's disease/dementia, Diabetes. Group 2 : Parkinson's disease, Severe Depression, Lung cancer, Bowel cancer, Prostate cancer. If any of codes -11, -13 or -17 were selected then no additional Group 1 choices were allowed. If any of codes -21, -23 or -27 were selected then no additional Group 2 choices were allowed. If the participant activated the Help button they were shown the message: Answer this question for blood relations only. If you are not sure if your father suffered from any of the listed illnesses please select Do not know. If you know your father suffered from certain listed illnesses but are unsure about others, only select the ones you are sure about. The information was collected from participants who indicated they were not adopted as a child, as defined by their answers to ~F1767~ and who indicated whether their natural father was still alive or had died, as defined by their answers to ~F1797~. Note that although Breast cancer is present in ~C1010~, participants were not presented with this option when selecting Father illnesses.-Variable type:binary</td></tr>
<tr><td>binary.20107_100.txt</td><td>1710174270056F5forCTG.txt.gz</td><td>-0.1791</td><td>0.05965</td><td>-3.002</td><td>0.002682</td><td>0.01374</td><td>0.002145</td><td>1.026</td><td>0.01088</td><td>0.007435</td><td>0.008423</td><td>Illnesses of father: None of the above (group 1)</td><td>FALSE</td><td></td><td></td><td></td><td>314797</td><td>116736</td><td>198061</td><td>UK Biobank</td><td>https://docs.google.com/spreadsheets/d/1kvPoupSzsSFBNSztMzl04xMoSC3Kcx3CrjVf4yBmESU/edit?ts=5b5f17db#gid=227859291</td><td>PHESANT Transformation:20107_0 || CAT-MUL-BINARY-VAR 100 || NO_NAN Remove NA participants 9656 || Removed 36741 examples != 100 but with missing value (<0) || sample 198061/116736(314797) ||-Notes:This field contains the combined results of 2 ACE touchscreen questions, both asked Has/did your father ever suffer from? (You can select more than one answer) for different sets of illnesses. For convenience, the illnesses were arbitrarily divided into two sets: Group 1 : Heart disease, Stroke, High blood pressure, Chronic bronchitis/emphysema, Alzheimer's disease/dementia, Diabetes. Group 2 : Parkinson's disease, Severe Depression, Lung cancer, Bowel cancer, Prostate cancer. If any of codes -11, -13 or -17 were selected then no additional Group 1 choices were allowed. If any of codes -21, -23 or -27 were selected then no additional Group 2 choices were allowed. If the participant activated the Help button they were shown the message: Answer this question for blood relations only. If you are not sure if your father suffered from any of the listed illnesses please select Do not know. If you know your father suffered from certain listed illnesses but are unsure about others, only select the ones you are sure about. The information was collected from participants who indicated they were not adopted as a child, as defined by their answers to ~F1767~ and who indicated whether their natural father was still alive or had died, as defined by their answers to ~F1797~. Note that although Breast cancer is present in ~C1010~, participants were not presented with this option when selecting Father illnesses.-Variable type:binary</td></tr>
<tr><td>binary.20107_101.txt</td><td>1710174270056F5forCTG.txt.gz</td><td>-0.1652</td><td>0.06016</td><td>-2.746</td><td>0.00603</td><td>0.01091</td><td>0.00188</td><td>0.992</td><td>0.01017</td><td>0.00202</td><td>0.008099</td><td>Illnesses of father: None of the above (group 2)</td><td>FALSE</td><td></td><td></td><td></td><td>318545</td><td>237674</td><td>80871</td><td>UK Biobank</td><td>https://docs.google.com/spreadsheets/d/1kvPoupSzsSFBNSztMzl04xMoSC3Kcx3CrjVf4yBmESU/edit?ts=5b5f17db#gid=227859291</td><td>PHESANT Transformation:20107_0 || CAT-MUL-BINARY-VAR 101 || NO_NAN Remove NA participants 9656 || Removed 32993 examples != 101 but with missing value (<0) || sample 80871/237674(318545) || SKIP_val:-21 < 0 || SKIP_val:-23 < 0 ||-Notes:This field contains the combined results of 2 ACE touchscreen questions, both asked Has/did your father ever suffer from? (You can select more than one answer) for different sets of illnesses. For convenience, the illnesses were arbitrarily divided into two sets: Group 1 : Heart disease, Stroke, High blood pressure, Chronic bronchitis/emphysema, Alzheimer's disease/dementia, Diabetes. Group 2 : Parkinson's disease, Severe Depression, Lung cancer, Bowel cancer, Prostate cancer. If any of codes -11, -13 or -17 were selected then no additional Group 1 choices were allowed. If any of codes -21, -23 or -27 were selected then no additional Group 2 choices were allowed. If the participant activated the Help button they were shown the message: Answer this question for blood relations only. If you are not sure if your father suffered from any of the listed illnesses please select Do not know. If you know your father suffered from certain listed illnesses but are unsure about others, only select the ones you are sure about. The information was collected from participants who indicated they were not adopted as a child, as defined by their answers to ~F1767~ and who indicated whether their natural father was still alive or had died, as defined by their answers to ~F1797~. Note that although Breast cancer is present in ~C1010~, participants were not presented with this option when selecting Father illnesses.-Variable type:binary</td></tr>
<tr><td>binary.20107_11.txt</td><td>1710174270056F5forCTG.txt.gz</td><td>-0.02435</td><td>0.102</td><td>-0.2387</td><td>0.8114</td><td>0.003669</td><td>0.001705</td><td>0.994</td><td>0.01041</td><td>-0.00037</td><td>0.008575</td><td>Illnesses of father: Parkinson's disease</td><td>FALSE</td><td></td><td></td><td></td><td>312104</td><td>8043</td><td>304061</td><td>UK Biobank</td><td>https://docs.google.com/spreadsheets/d/1kvPoupSzsSFBNSztMzl04xMoSC3Kcx3CrjVf4yBmESU/edit?ts=5b5f17db#gid=227859291</td><td>PHESANT Transformation:20107_0 || CAT-MUL-BINARY-VAR 11 || NO_NAN Remove NA participants 9656 || Removed 39434 examples != 11 but with missing value (<0) || sample 304061/8043(312104) ||-Notes:This field contains the combined results of 2 ACE touchscreen questions, both asked Has/did your father ever suffer from? (You can select more than one answer) for different sets of illnesses. For convenience, the illnesses were arbitrarily divided into two sets: Group 1 : Heart disease, Stroke, High blood pressure, Chronic bronchitis/emphysema, Alzheimer's disease/dementia, Diabetes. Group 2 : Parkinson's disease, Severe Depression, Lung cancer, Bowel cancer, Prostate cancer. If any of codes -11, -13 or -17 were selected then no additional Group 1 choices were allowed. If any of codes -21, -23 or -27 were selected then no additional Group 2 choices were allowed. If the participant activated the Help button they were shown the message: Answer this question for blood relations only. If you are not sure if your father suffered from any of the listed illnesses please select Do not know. If you know your father suffered from certain listed illnesses but are unsure about others, only select the ones you are sure about. The information was collected from participants who indicated they were not adopted as a child, as defined by their answers to ~F1767~ and who indicated whether their natural father was still alive or had died, as defined by their answers to ~F1797~. Note that although Breast cancer is present in ~C1010~, participants were not presented with this option when selecting Father illnesses.-Variable type:binary</td></tr>
<tr><td>binary.20107_12.txt</td><td>1710174270056F5forCTG.txt.gz</td><td>0.008508</td><td>0.06541</td><td>0.1301</td><td>0.8965</td><td>0.007776</td><td>0.001898</td><td>0.993</td><td>0.009446</td><td>-0.001546</td><td>0.007494</td><td>Illnesses of father: Severe depression</td><td>FALSE</td><td></td><td></td><td></td><td>312437</td><td>11921</td><td>300516</td><td>UK Biobank</td><td>https://docs.google.com/spreadsheets/d/1kvPoupSzsSFBNSztMzl04xMoSC3Kcx3CrjVf4yBmESU/edit?ts=5b5f17db#gid=227859291</td><td>PHESANT Transformation:20107_0 || CAT-MUL-BINARY-VAR 12 || NO_NAN Remove NA participants 9656 || Removed 39101 examples != 12 but with missing value (<0) || sample 300516/11921(312437) ||-Notes:This field contains the combined results of 2 ACE touchscreen questions, both asked Has/did your father ever suffer from? (You can select more than one answer) for different sets of illnesses. For convenience, the illnesses were arbitrarily divided into two sets: Group 1 : Heart disease, Stroke, High blood pressure, Chronic bronchitis/emphysema, Alzheimer's disease/dementia, Diabetes. Group 2 : Parkinson's disease, Severe Depression, Lung cancer, Bowel cancer, Prostate cancer. If any of codes -11, -13 or -17 were selected then no additional Group 1 choices were allowed. If any of codes -21, -23 or -27 were selected then no additional Group 2 choices were allowed. If the participant activated the Help button they were shown the message: Answer this question for blood relations only. If you are not sure if your father suffered from any of the listed illnesses please select Do not know. If you know your father suffered from certain listed illnesses but are unsure about others, only select the ones you are sure about. The information was collected from participants who indicated they were not adopted as a child, as defined by their answers to ~F1767~ and who indicated whether their natural father was still alive or had died, as defined by their answers to ~F1797~. Note that although Breast cancer is present in ~C1010~, participants were not presented with this option when selecting Father illnesses.-Variable type:binary</td></tr>
</table>

<table>
<tr><th></th><th></th><th></th><th></th><th></th><th></th><th></th><th></th><th></th><th></th><th></th><th></th><th></th><th></th><th></th><th></th><th></th><th></th><th></th><th></th><th></th><th></th><th></th></tr>
<tr><td>binary.20107_13.txt</td><td>1710174270056F5forCTG.txt.gz</td><td>0.01334</td><td>0.0748</td><td>0.1784</td><td>0.8584</td><td>0.007298</td><td>0.002091</td><td>1.012</td><td>0.01288</td><td>-0.001086</td><td>0.007937</td><td>Illnesses of father: Prostate cancer</td><td>FALSE</td><td></td><td></td><td></td><td>312544</td><td>24207</td><td>288337</td><td>UK Biobank</td><td>https://docs.google.com/spreadsheets/d/1kvPoupSzsSFBNSztMzl04xMoSC3Kcx3CrjVf4yBmESU/edit?ts=5b5f17db#gid=227859291</td><td>PHESANT Transformation:20107_0 || CAT-MUL-BINARY-VAR 13 || NO_NAN Remove NA participants 9656 || Removed 38994 examples != 13 but with missing value (<0) || sample 288337/24207(312544) || SKIP_val:-11 < 0 ||-Notes:This field contains the combined results of 2 ACE touchscreen questions, both asked Has/did your father ever suffer from? (You can select more than one answer) for different sets of illnesses. For convenience, the illnesses were arbitrarily divided into two sets: Group 1 : Heart disease, Stroke, High blood pressure, Chronic bronchitis/emphysema, Alzheimer's disease/dementia, Diabetes. Group 2 : Parkinson's disease, Severe Depression, Lung cancer, Bowel cancer, Prostate cancer. If any of codes -11, -13 or -17 were selected then no additional Group 1 choices were allowed. If any of codes -21, -23 or -27 were selected then no additional Group 2 choices were allowed. If the participant activated the Help button they were shown the message: Answer this question for blood relations only. If you are not sure if your father suffered from any of the listed illnesses please select Do not know. If you know your father suffered from certain listed illnesses but are unsure about others, only select the ones you are sure about. The information was collected from participants who indicated they were not adopted as a child, as defined by their answers to ~F1767~ and who indicated whether their natural father was still alive or had died, as defined by their answers to ~F1797~. Note that although Breast cancer is present in ~C1010~, participants were not presented with this option when selecting Father illnesses.-Variable type:binary</td></tr>
<tr><td>binary.20107_2.txt</td><td>1710174270056F5forCTG.txt.gz</td><td>0.0007068</td><td>0.09897</td><td>0.007141</td><td>0.9943</td><td>0.003916</td><td>0.00172</td><td>1.009</td><td>0.01009</td><td>0.0005385</td><td>0.008045</td><td>Illnesses of father: Stroke</td><td>FALSE</td><td></td><td></td><td></td><td>314801</td><td>49173</td><td>265628</td><td>UK Biobank</td><td>https://docs.google.com/spreadsheets/d/1kvPoupSzsSFBNSztMzl04xMoSC3Kcx3CrjVf4yBmESU/edit?ts=5b5f17db#gid=227859291</td><td>PHESANT Transformation:20107_0 || CAT-MUL-BINARY-VAR 2 || NO_NAN Remove NA participants 9656 || Removed 36737 examples != 2 but with missing value (<0) || sample 265628/49173(314801) ||-Notes:This field contains the combined results of 2 ACE touchscreen questions, both asked Has/did your father ever suffer from? (You can select more than one answer) for different sets of illnesses. For convenience, the illnesses were arbitrarily divided into two sets: Group 1 : Heart disease, Stroke, High blood pressure, Chronic bronchitis/emphysema, Alzheimer's disease/dementia, Diabetes. Group 2 : Parkinson's disease, Severe Depression, Lung cancer, Bowel cancer, Prostate cancer. If any of codes -11, -13 or -17 were selected then no additional Group 1 choices were allowed. If any of codes -21, -23 or -27 were selected then no additional Group 2 choices were allowed. If the participant activated the Help button they were shown the message: Answer this question for blood relations only. If you are not sure if your father suffered from any of the listed illnesses please select Do not know. If you know your father suffered from certain listed illnesses but are unsure about others, only select the ones you are sure about. The information was collected from participants who indicated they were not adopted as a child, as defined by their answers to ~F1767~ and who indicated whether their natural father was still alive or had died, as defined by their answers to ~F1797~. Note that although Breast cancer is present in ~C1010~, participants were not presented with this option when selecting Father illnesses.-Variable type:binary</td></tr>
<tr><td>binary.20107_3.txt</td><td>1710174270056F5forCTG.txt.gz</td><td>0.2569</td><td>0.05155</td><td>4.983</td><td>6.25E-07</td><td>0.01567</td><td>0.002159</td><td>0.982</td><td>0.009898</td><td>-0.01032</td><td>0.007727</td><td>Illnesses of father: Lung cancer</td><td>FALSE</td><td>Other</td><td></td><td></td><td>314072</td><td>29206</td><td>284866</td><td>UK Biobank</td><td>https://docs.google.com/spreadsheets/d/1kvPoupSzsSFBNSztMzl04xMoSC3Kcx3CrjVf4yBmESU/edit?ts=5b5f17db#gid=227859291</td><td>PHESANT Transformation:20107_0 || CAT-MUL-BINARY-VAR 3 || NO_NAN Remove NA participants 9656 || Removed 37466 examples != 3 but with missing value (<0) || sample 284866/29206(314072) ||-Notes:This field contains the combined results of 2 ACE touchscreen questions, both asked Has/did your father ever suffer from? (You can select more than one answer) for different sets of illnesses. For convenience, the illnesses were arbitrarily divided into two sets: Group 1 : Heart disease, Stroke, High blood pressure, Chronic bronchitis/emphysema, Alzheimer's disease/dementia, Diabetes. Group 2 : Parkinson's disease, Severe Depression, Lung cancer, Bowel cancer, Prostate cancer. If any of codes -11, -13 or -17 were selected then no additional Group 1 choices were allowed. If any of codes -21, -23 or -27 were selected then no additional Group 2 choices were allowed. If the participant activated the Help button they were shown the message: Answer this question for blood relations only. If you are not sure if your father suffered from any of the listed illnesses please select Do not know. If you know your father suffered from certain listed illnesses but are unsure about others, only select the ones you are sure about. The information was collected from participants who indicated they were not adopted as a child, as defined by their answers to ~F1767~ and who indicated whether their natural father was still alive or had died, as defined by their answers to ~F1797~. Note that although Breast cancer is present in ~C1010~, participants were not presented with this option when selecting Father illnesses.-Variable type:binary</td></tr>
<tr><td>binary.20107_6.txt</td><td>1710174270056F5forCTG.txt.gz</td><td>0.2911</td><td>0.06338</td><td>4.594</td><td>4.35E-06</td><td>0.01377</td><td>0.00215</td><td>1.011</td><td>0.01028</td><td>0.005561</td><td>0.008518</td><td>Illnesses of father: Chronic bronchitis/emphysema</td><td>FALSE</td><td>Pulmonary</td><td></td><td></td><td>314680</td><td>36126</td><td>278554</td><td>UK Biobank</td><td>https://docs.google.com/spreadsheets/d/1kvPoupSzsSFBNSztMzl04xMoSC3Kcx3CrjVf4yBmESU/edit?ts=5b5f17db#gid=227859291</td><td>PHESANT Transformation:20107_0 || CAT-MUL-BINARY-VAR 6 || NO_NAN Remove NA participants 9656 || Removed 36858 examples != 6 but with missing value (<0) || sample 278554/36126(314680) ||-Notes:This field contains the combined results of 2 ACE touchscreen questions, both asked Has/did your father ever suffer from? (You can select more than one answer) for different sets of illnesses. For convenience, the illnesses were arbitrarily divided into two sets: Group 1 : Heart disease, Stroke, High blood pressure, Chronic bronchitis/emphysema, Alzheimer's disease/dementia, Diabetes. Group 2 : Parkinson's disease, Severe Depression, Lung cancer, Bowel cancer, Prostate cancer. If any of codes -11, -13 or -17 were selected then no additional Group 1 choices were allowed. If any of codes -21, -23 or -27 were selected then no additional Group 2 choices were allowed. If the participant activated the Help button they were shown the message: Answer this question for blood relations only. If you are not sure if your father suffered from any of the listed illnesses please select Do not know. If you know your father suffered from certain listed illnesses but are unsure about others, only select the ones you are sure about. The information was collected from participants who indicated they were not adopted as a child, as defined by their answers to ~F1767~ and who indicated whether their natural father was still alive or had died, as defined by their answers to ~F1797~. Note that although Breast cancer is present in ~C1010~, participants were not presented with this option when selecting Father illnesses.-Variable type:binary</td></tr>
<tr><td>binary.20107_8.txt</td><td>1710174270056F5forCTG.txt.gz</td><td>0.1783</td><td>0.05223</td><td>3.415</td><td>0.000639</td><td>0.01685</td><td>0.002184</td><td>1.023</td><td>0.01141</td><td>-0.0177</td><td>0.00865</td><td>Illnesses of father: High blood pressure</td><td>FALSE</td><td></td><td></td><td></td><td>315021</td><td>71136</td><td>243885</td><td>UK Biobank</td><td>https://docs.google.com/spreadsheets/d/1kvPoupSzsSFBNSztMzl04xMoSC3Kcx3CrjVf4yBmESU/edit?ts=5b5f17db#gid=227859291</td><td>PHESANT Transformation:20107_0 || CAT-MUL-BINARY-VAR 8 || NO_NAN Remove NA participants 9656 || Removed 36517 examples != 8 but with missing value (<0) || sample 243885/71136(315021) ||-Notes:This field contains the combined results of 2 ACE touchscreen questions, both asked Has/did your father ever suffer from? (You can select more than one answer) for different sets of illnesses. For convenience, the illnesses were arbitrarily divided into two sets: Group 1 : Heart disease, Stroke, High blood pressure, Chronic bronchitis/emphysema, Alzheimer's disease/dementia, Diabetes. Group 2 : Parkinson's disease, Severe Depression, Lung cancer, Bowel cancer, Prostate cancer. If any of codes -11, -13 or -17 were selected then no additional Group 1 choices were allowed. If any of codes -21, -23 or -27 were selected then no additional Group 2 choices were allowed. If the participant activated the Help button they were shown the message: Answer this question for blood relations only. If you are not sure if your father suffered from any of the listed illnesses please select Do not know. If you know your father suffered from certain listed illnesses but are unsure about others, only select the ones you are sure about. The information was collected from participants who indicated they were not adopted as a child, as defined by their answers to ~F1767~ and who indicated whether their natural father was still alive or had died, as defined by their answers to ~F1797~. Note that although Breast cancer is present in ~C1010~, participants were not presented with this option when selecting Father illnesses.-Variable type:binary</td></tr>
</table>

<table>
<tr><td>binary.20107_9.txt</td><td>1710174270056F5 forCTG.txt.gz</td><td>0.2547</td><td>0.05333</td><td>4.776</td><td>1.79E-06</td><td>0.01799</td><td>0.002644</td><td>1.025</td><td>0.01162</td><td>-0.01332</td><td>0.008551</td><td>Illnesses of father: Diabetes</td><td>FALSE</td><td>Metabolic</td><td></td><td></td><td>313294</td><td>30010</td><td>283284</td><td>UK Biobank</td><td>https://docs.google.com/spreadsheets/d/1kvPoupSzsSFBNSztMzl04xMoSC3Kcx3CrjVf4yBmESU/edit?ts=5b5f17db#gid=227859291</td><td>PHESANT Transformation:20107_0 || CAT-MUL-BINARY-VAR 9 || NO_NAN Remove NA participants 9656 || Removed 38244 examples != 9 but with missing value (<0) || sample 283284/30010(313294) ||-Notes:This field contains the combined results of 2 ACE touchscreen questions, both asked Has/did your father ever suffer from? (You can select more than one answer) for different sets of illnesses. For convenience, the illnesses were arbitrarily divided into two sets: Group 1 : Heart disease, Stroke, High blood pressure, Chronic bronchitis/emphysema, Alzheimer's disease/dementia, Diabetes. Group 2 : Parkinson's disease, Severe Depression, Lung cancer, Bowel cancer, Prostate cancer. If any of codes -11, -13 or -17 were selected then no additional Group 1 choices were allowed. If any of codes -21, -23 or -27 were selected then no additional Group 2 choices were allowed. If the participant activated the Help button they were shown the message: Answer this question for blood relations only. If you are not sure if your father suffered from any of the listed illnesses please select Do not know. If you know your father suffered from certain listed illnesses but are unsure about others, only select the ones you are sure about. The information was collected from participants who indicated they were not adopted as a child, as defined by their answers to ~F1767~ and who indicated whether their natural father was still alive or had died, as defined by their answers to ~F1797~. Note that although Breast cancer is present in ~C1010~, participants were not presented with this option when selecting Father illnesses.-Variable type:binary</td></tr>
<tr><td>binary.20110_1.txt</td><td>1710174270056F5 forCTG.txt.gz</td><td>0.2441</td><td>0.06047</td><td>4.036</td><td>5.43E-05</td><td>0.01368</td><td>0.002234</td><td>1.021</td><td>0.01108</td><td>0.003238</td><td>0.008389</td><td>Illnesses of mother: Heart disease</td><td>FALSE</td><td></td><td></td><td></td><td>332966</td><td>66343</td><td>266623</td><td>UK Biobank</td><td>https://docs.google.com/spreadsheets/d/1kvPoupSzsSFBNSztMzl04xMoSC3Kcx3CrjVf4yBmESU/edit?ts=5b5f17db#gid=227859291</td><td>PHESANT Transformation:20110_0 || CAT-MUL-BINARY-VAR 1 || NO_NAN Remove NA participants 6165 || Removed 22063 examples != 1 but with missing value (<0) || sample 266623/66343(332966) ||-Notes:This field contains the combined results of 2 ACE touchscreen questions, both asked Has/did your mother ever suffer from? (You can select more than one answer) for different sets of illnesses. For convenience, the illnesses were arbitrarily divided into two sets: Group 1 : Heart disease, Stroke, High blood pressure, Chronic bronchitis/emphysema, Alzheimer's disease/dementia, Diabetes. Group 2 : Parkinson's disease, Severe Depression, Lung cancer, Bowel cancer, Breast cancer. If any of codes -11, -13 or -17 were selected then no additional Group 1 choices were allowed. If any of codes -21, -23 or -27 were selected then no additional Group 2 choices were allowed. If the participant activated the Help button they were shown the message: Answer this question for blood relations only. If you are not sure if your mother suffered from any of the listed illnesses please select Do not know. If you know your mother suffered from certain listed illnesses but are unsure about others, only select the ones you are sure about. The information was collected from participants who indicated they were not adopted as a child, as defined by their answers to ~F1767~ and who indicated whether their natural mother was still alive or had died, as defined by their answers to ~F1835~. Note that although Prostate cancer is present in ~C1010~, participants were not presented with this option when selecting Mother illnesses.-Variable type:binary</td></tr>
<tr><td>binary.20110_100.txt</td><td>1710174270056F5 forCTG.txt.gz</td><td>-0.1685</td><td>0.05487</td><td>-3.07</td><td>0.002142</td><td>0.01606</td><td>0.002125</td><td>1.025</td><td>0.01213</td><td>0.002959</td><td>0.008401</td><td>Illnesses of mother: None of the above (group 1)</td><td>FALSE</td><td></td><td></td><td></td><td>332611</td><td>138291</td><td>194320</td><td>UK Biobank</td><td>https://docs.google.com/spreadsheets/d/1kvPoupSzsSFBNSztMzl04xMoSC3Kcx3CrjVf4yBmESU/edit?ts=5b5f17db#gid=227859291</td><td>PHESANT Transformation:20110_0 || CAT-MUL-BINARY-VAR 100 || NO_NAN Remove NA participants 6165 || Removed 22418 examples != 100 but with missing value (<0) || sample 194320/138291(332611) ||-Notes:This field contains the combined results of 2 ACE touchscreen questions, both asked Has/did your mother ever suffer from? (You can select more than one answer) for different sets of illnesses. For convenience, the illnesses were arbitrarily divided into two sets: Group 1 : Heart disease, Stroke, High blood pressure, Chronic bronchitis/emphysema, Alzheimer's disease/dementia, Diabetes. Group 2 : Parkinson's disease, Severe Depression, Lung cancer, Bowel cancer, Breast cancer. If any of codes -11, -13 or -17 were selected then no additional Group 1 choices were allowed. If any of codes -21, -23 or -27 were selected then no additional Group 2 choices were allowed. If the participant activated the Help button they were shown the message: Answer this question for blood relations only. If you are not sure if your mother suffered from any of the listed illnesses please select Do not know. If you know your mother suffered from certain listed illnesses but are unsure about others, only select the ones you are sure about. The information was collected from participants who indicated they were not adopted as a child, as defined by their answers to ~F1767~ and who indicated whether their natural mother was still alive or had died, as defined by their answers to ~F1835~. Note that although Prostate cancer is present in ~C1010~, participants were not presented with this option when selecting Mother illnesses.-Variable type:binary</td></tr>
<tr><td>binary.20110_101.txt</td><td>1710174270056F5 forCTG.txt.gz</td><td>-0.1516</td><td>0.06177</td><td>-2.455</td><td>0.0141</td><td>0.007935</td><td>0.001736</td><td>1.002</td><td>0.009922</td><td>-0.005784</td><td>0.007334</td><td>Illnesses of mother: None of the above (group 2)</td><td>FALSE</td><td></td><td></td><td></td><td>334401</td><td>257717</td><td>76684</td><td>UK Biobank</td><td>https://docs.google.com/spreadsheets/d/1kvPoupSzsSFBNSztMzl04xMoSC3Kcx3CrjVf4yBmESU/edit?ts=5b5f17db#gid=227859291</td><td>PHESANT Transformation:20110_0 || CAT-MUL-BINARY-VAR 101 || NO_NAN Remove NA participants 6165 || Removed 20628 examples != 101 but with missing value (<0) || sample 76684/257717(334401) || SKIP_val:-21 < 0 || SKIP_val:-23 < 0 ||-Notes:This field contains the combined results of 2 ACE touchscreen questions, both asked Has/did your mother ever suffer from? (You can select more than one answer) for different sets of illnesses. For convenience, the illnesses were arbitrarily divided into two sets: Group 1 : Heart disease, Stroke, High blood pressure, Chronic bronchitis/emphysema, Alzheimer's disease/dementia, Diabetes. Group 2 : Parkinson's disease, Severe Depression, Lung cancer, Bowel cancer, Breast cancer. If any of codes -11, -13 or -17 were selected then no additional Group 1 choices were allowed. If any of codes -21, -23 or -27 were selected then no additional Group 2 choices were allowed. If the participant activated the Help button they were shown the message: Answer this question for blood relations only. If you are not sure if your mother suffered from any of the listed illnesses please select Do not know. If you know your mother suffered from certain listed illnesses but are unsure about others, only select the ones you are sure about. The information was collected from participants who indicated they were not adopted as a child, as defined by their answers to ~F1767~ and who indicated whether their natural mother was still alive or had died, as defined by their answers to ~F1835~. Note that although Prostate cancer is present in ~C1010~, participants were not presented with this option when selecting Mother illnesses.-Variable type:binary</td></tr>
<tr><td>binary.20110_12.txt</td><td>1710174270056F5 forCTG.txt.gz</td><td>0.1576</td><td>0.0539</td><td>2.925</td><td>0.003448</td><td>0.009315</td><td>0.001886</td><td>1.008</td><td>0.01005</td><td>0.0001989</td><td>0.007305</td><td>Illnesses of mother: Severe depression</td><td>FALSE</td><td></td><td></td><td></td><td>330653</td><td>22004</td><td>308649</td><td>UK Biobank</td><td>https://docs.google.com/spreadsheets/d/1kvPoupSzsSFBNSztMzl04xMoSC3Kcx3CrjVf4yBmESU/edit?ts=5b5f17db#gid=227859291</td><td>PHESANT Transformation:20110_0 || CAT-MUL-BINARY-VAR 12 || NO_NAN Remove NA participants 6165 || Removed 24376 examples != 12 but with missing value (<0) || sample 308649/22004(330653) ||-Notes:This field contains the combined results of 2 ACE touchscreen questions, both asked Has/did your mother ever suffer from? (You can select more than one answer) for different sets of illnesses. For convenience, the illnesses were arbitrarily divided into two sets: Group 1 : Heart disease, Stroke, High blood pressure, Chronic bronchitis/emphysema, Alzheimer's disease/dementia, Diabetes. Group 2 : Parkinson's disease, Severe Depression, Lung cancer, Bowel cancer, Breast cancer. If any of codes -11, -13 or -17 were selected then no additional Group 1 choices were allowed. If any of codes -21, -23 or -27 were selected then no additional Group 2 choices were allowed. If the participant activated the Help button they were shown the message: Answer this question for blood relations only. If you are not sure if your mother suffered from any of the listed illnesses please select Do not know. If you know your mother suffered from certain listed illnesses but are unsure about others, only select the ones you are sure about. The information was collected from participants who indicated they were not adopted as a child, as defined by their answers to ~F1767~ and who indicated whether their natural mother was still alive or had died, as defined by their answers to ~F1835~. Note that although Prostate cancer is present in ~C1010~, participants were not presented with this option when selecting Mother illnesses.-Variable type:binary</td></tr>
</table>

<table>
<tr><th></th><th></th><th></th><th></th><th></th><th></th><th></th><th></th><th></th><th></th><th></th><th></th><th></th><th></th><th></th><th></th><th></th><th></th><th></th><th></th><th></th><th></th><th></th></tr>
<tr><td>binary.20110_2.txt</td><td>1710174270056F5forCTG.txt.gz</td><td>0.07758</td><td>0.08414</td><td>0.9221</td><td>0.3565</td><td>0.006326</td><td>0.001968</td><td>1.009</td><td>0.009867</td><td>-0.008206</td><td>0.008624</td><td>Illnesses of mother: Stroke</td><td>FALSE</td><td></td><td></td><td></td><td>331973</td><td>47485</td><td>284488</td><td>UK Biobank</td><td>https://docs.google.com/spreadsheets/d/1kvPoupSzsSFBNSztMzl04xMoSC3Kcx3CrjVf4yBmESU/edit?ts=5b5f17db#gid=227859291</td><td>PHESANT Transformation:20110_0 | | CAT-MUL-BINARY-VAR 2 | | NO_NAN Remove NA participants 6165 | | Removed 23056 examples != 2 but with missing value (<0) | | sample 284488/47485(331973) | |-Notes:This field contains the combined results of 2 ACE touchscreen questions, both asked Has/did your mother ever suffer from? (You can select more than one answer) for different sets of illnesses. For convenience, the illnesses were arbitrarily divided into two sets: Group 1 : Heart disease, Stroke, High blood pressure, Chronic bronchitis/emphysema, Alzheimer's disease/dementia, Diabetes. Group 2 : Parkinson's disease, Severe Depression, Lung cancer, Bowel cancer, Breast cancer. If any of codes -11, -13 or -17 were selected then no additional Group 1 choices were allowed. If any of codes -21, -23 or -27 were selected then no additional Group 2 choices were allowed. If the participant activated the Help button they were shown the message: Answer this question for blood relations only. If you are not sure if your mother suffered from any of the listed illnesses please select Do not know. If you know your mother suffered from certain listed illnesses but are unsure about others, only select the ones you are sure about. The information was collected from participants who indicated they were not adopted as a child, as defined by their answers to ~F1767~ and who indicated whether their natural mother was still alive or had died, as defined by their answers to ~F1835~. Note that although Prostate cancer is present in ~C1010~, participants were not presented with this option when selecting Mother illnesses.-Variable type:binary</td></tr>
<tr><td>binary.20110_3.txt</td><td>1710174270056F5forCTG.txt.gz</td><td>0.3152</td><td>0.1126</td><td>2.799</td><td>0.005127</td><td>0.004035</td><td>0.001691</td><td>1.001</td><td>0.009222</td><td>0.009961</td><td>0.007676</td><td>Illnesses of mother: Lung cancer</td><td>FALSE</td><td></td><td></td><td></td><td>330689</td><td>13689</td><td>317000</td><td>UK Biobank</td><td>https://docs.google.com/spreadsheets/d/1kvPoupSzsSFBNSztMzl04xMoSC3Kcx3CrjVf4yBmESU/edit?ts=5b5f17db#gid=227859291</td><td>PHESANT Transformation:20110_0 | | CAT-MUL-BINARY-VAR 3 | | NO_NAN Remove NA participants 6165 | | Removed 24340 examples != 3 but with missing value (<0) | | sample 317000/13689(330689) | | SKIP_val:-11 < 0 | |-Notes:This field contains the combined results of 2 ACE touchscreen questions, both asked Has/did your mother ever suffer from? (You can select more than one answer) for different sets of illnesses. For convenience, the illnesses were arbitrarily divided into two sets: Group 1 : Heart disease, Stroke, High blood pressure, Chronic bronchitis/emphysema, Alzheimer's disease/dementia, Diabetes. Group 2 : Parkinson's disease, Severe Depression, Lung cancer, Bowel cancer, Breast cancer. If any of codes -11, -13 or -17 were selected then no additional Group 1 choices were allowed. If any of codes -21, -23 or -27 were selected then no additional Group 2 choices were allowed. If the participant activated the Help button they were shown the message: Answer this question for blood relations only. If you are not sure if your mother suffered from any of the listed illnesses please select Do not know. If you know your mother suffered from certain listed illnesses but are unsure about others, only select the ones you are sure about. The information was collected from participants who indicated they were not adopted as a child, as defined by their answers to ~F1767~ and who indicated whether their natural mother was still alive or had died, as defined by their answers to ~F1835~. Note that although Prostate cancer is present in ~C1010~, participants were not presented with this option when selecting Mother illnesses.-Variable type:binary</td></tr>
<tr><td>binary.20110_4.txt</td><td>1710174270056F5forCTG.txt.gz</td><td>0.1081</td><td>0.1123</td><td>0.9626</td><td>0.3357</td><td>0.004244</td><td>0.001764</td><td>1.009</td><td>0.009457</td><td>0.004836</td><td>0.00874</td><td>Illnesses of mother: Bowel cancer</td><td>FALSE</td><td></td><td></td><td></td><td>330592</td><td>17264</td><td>313328</td><td>UK Biobank</td><td>https://docs.google.com/spreadsheets/d/1kvPoupSzsSFBNSztMzl04xMoSC3Kcx3CrjVf4yBmESU/edit?ts=5b5f17db#gid=227859291</td><td>PHESANT Transformation:20110_0 | | CAT-MUL-BINARY-VAR 4 | | NO_NAN Remove NA participants 6165 | | Removed 24437 examples != 4 but with missing value (<0) | | sample 313328/17264(330592) | |-Notes:This field contains the combined results of 2 ACE touchscreen questions, both asked Has/did your mother ever suffer from? (You can select more than one answer) for different sets of illnesses. For convenience, the illnesses were arbitrarily divided into two sets: Group 1 : Heart disease, Stroke, High blood pressure, Chronic bronchitis/emphysema, Alzheimer's disease/dementia, Diabetes. Group 2 : Parkinson's disease, Severe Depression, Lung cancer, Bowel cancer, Breast cancer. If any of codes -11, -13 or -17 were selected then no additional Group 1 choices were allowed. If any of codes -21, -23 or -27 were selected then no additional Group 2 choices were allowed. If the participant activated the Help button they were shown the message: Answer this question for blood relations only. If you are not sure if your mother suffered from any of the listed illnesses please select Do not know. If you know your mother suffered from certain listed illnesses but are unsure about others, only select the ones you are sure about. The information was collected from participants who indicated they were not adopted as a child, as defined by their answers to ~F1767~ and who indicated whether their natural mother was still alive or had died, as defined by their answers to ~F1835~. Note that although Prostate cancer is present in ~C1010~, participants were not presented with this option when selecting Mother illnesses.-Variable type:binary</td></tr>
<tr><td>binary.20110_5.txt</td><td>1710174270056F5forCTG.txt.gz</td><td>-0.01988</td><td>0.0593</td><td>-0.3353</td><td>0.7374</td><td>0.009426</td><td>0.002162</td><td>1</td><td>0.01257</td><td>-0.006854</td><td>0.006993</td><td>Illnesses of mother: Breast cancer</td><td>FALSE</td><td></td><td></td><td></td><td>330828</td><td>27622</td><td>303206</td><td>UK Biobank</td><td>https://docs.google.com/spreadsheets/d/1kvPoupSzsSFBNSztMzl04xMoSC3Kcx3CrjVf4yBmESU/edit?ts=5b5f17db#gid=227859291</td><td>PHESANT Transformation:20110_0 | | CAT-MUL-BINARY-VAR 5 | | NO_NAN Remove NA participants 6165 | | Removed 24201 examples != 5 but with missing value (<0) | | sample 303206/27622(330828) | |-Notes:This field contains the combined results of 2 ACE touchscreen questions, both asked Has/did your mother ever suffer from? (You can select more than one answer) for different sets of illnesses. For convenience, the illnesses were arbitrarily divided into two sets: Group 1 : Heart disease, Stroke, High blood pressure, Chronic bronchitis/emphysema, Alzheimer's disease/dementia, Diabetes. Group 2 : Parkinson's disease, Severe Depression, Lung cancer, Bowel cancer, Breast cancer. If any of codes -11, -13 or -17 were selected then no additional Group 1 choices were allowed. If any of codes -21, -23 or -27 were selected then no additional Group 2 choices were allowed. If the participant activated the Help button they were shown the message: Answer this question for blood relations only. If you are not sure if your mother suffered from any of the listed illnesses please select Do not know. If you know your mother suffered from certain listed illnesses but are unsure about others, only select the ones you are sure about. The information was collected from participants who indicated they were not adopted as a child, as defined by their answers to ~F1767~ and who indicated whether their natural mother was still alive or had died, as defined by their answers to ~F1835~. Note that although Prostate cancer is present in ~C1010~, participants were not presented with this option when selecting Mother illnesses.-Variable type:binary</td></tr>
<tr><td>binary.20110_6.txt</td><td>1710174270056F5forCTG.txt.gz</td><td>0.4004</td><td>0.07309</td><td>5.478</td><td>4.31E-08</td><td>0.01003</td><td>0.002038</td><td>1.013</td><td>0.009597</td><td>-0.000732</td><td>0.007863</td><td>Illnesses of mother: Chronic bronchitis/emphysema</td><td>FALSE</td><td>Pulmonary</td><td></td><td></td><td>331008</td><td>19637</td><td>311371</td><td>UK Biobank</td><td>https://docs.google.com/spreadsheets/d/1kvPoupSzsSFBNSztMzl04xMoSC3Kcx3CrjVf4yBmESU/edit?ts=5b5f17db#gid=227859291</td><td>PHESANT Transformation:20110_0 | | CAT-MUL-BINARY-VAR 6 | | NO_NAN Remove NA participants 6165 | | Removed 24021 examples != 6 but with missing value (<0) | | sample 311371/19637(331008) | | SKIP_val:-13 < 0 | |-Notes:This field contains the combined results of 2 ACE touchscreen questions, both asked Has/did your mother ever suffer from? (You can select more than one answer) for different sets of illnesses. For convenience, the illnesses were arbitrarily divided into two sets: Group 1 : Heart disease, Stroke, High blood pressure, Chronic bronchitis/emphysema, Alzheimer's disease/dementia, Diabetes. Group 2 : Parkinson's disease, Severe Depression, Lung cancer, Bowel cancer, Breast cancer. If any of codes -11, -13 or -17 were selected then no additional Group 1 choices were allowed. If any of codes -21, -23 or -27 were selected then no additional Group 2 choices were allowed. If the participant activated the Help button they were shown the message: Answer this question for blood relations only. If you are not sure if your mother suffered from any of the listed illnesses please select Do not know. If you know your mother suffered from certain listed illnesses but are unsure about others, only select the ones you are sure about. The information was collected from participants who indicated they were not adopted as a child, as defined by their answers to ~F1767~ and who indicated whether their natural mother was still alive or had died, as defined by their answers to ~F1835~. Note that although Prostate cancer is present in ~C1010~, participants were not presented with this option when selecting Mother illnesses.-Variable type:binary</td></tr>
</table>

<table>
<tr><td></td><td></td><td></td><td></td><td></td><td></td><td></td><td></td><td></td><td></td><td></td><td></td><td></td><td></td><td></td><td></td><td></td><td></td><td></td><td></td><td></td><td></td><td></td></tr>
<tr><td>binary.20110_8.txt</td><td>1710174270056F5forCTG.txt.gz</td><td>0.1037</td><td>0.04189</td><td>2.475</td><td>0.01332</td><td>0.02784</td><td>0.00253</td><td>1.017</td><td>0.01293</td><td>-0.01251</td><td>0.008916</td><td>Illnesses of mother: High blood pressure</td><td>FALSE</td><td></td><td></td><td></td><td>333048</td><td>101688</td><td>231360</td><td>UK Biobank</td><td>https://docs.google.com/spreadsheets/d/1kvPoupSzsSFBNSztMzl04xMoSC3Kcx3CrjVf4yBmESU/edit?ts=5b5f17db#gid=227859291</td><td>PHESANT Transformation:20110_0 || CAT-MUL-BINARY-VAR 8 || NO_NAN Remove NA participants 6165 || Removed 21981 examples != 8 but with missing value (<0) || sample 231360/101688(333048) ||-Notes:This field contains the combined results of 2 ACE touchscreen questions, both asked Has/did your mother ever suffer from? (You can select more than one answer) for different sets of illnesses. For convenience, the illnesses were arbitrarily divided into two sets: Group 1 : Heart disease, Stroke, High blood pressure, Chronic bronchitis/emphysema, Alzheimer's disease/dementia, Diabetes. Group 2 : Parkinson's disease, Severe Depression, Lung cancer, Bowel cancer, Breast cancer. If any of codes -11, -13 or -17 were selected then no additional Group 1 choices were allowed. If any of codes -21, -23 or -27 were selected then no additional Group 2 choices were allowed. If the participant activated the Help button they were shown the message: Answer this question for blood relations only. If you are not sure if your mother suffered from any of the listed illnesses please select Do not know. If you know your mother suffered from certain listed illnesses but are unsure about others, only select the ones you are sure about. The information was collected from participants who indicated they were not adopted as a child, as defined by their answers to ~F1767~ and who indicated whether their natural mother was still alive or had died, as defined by their answers to ~F1835~. Note that although Prostate cancer is present in ~C1010~, participants were not presented with this option when selecting Mother illnesses.-Variable type:binary</td></tr>
<tr><td>binary.20110_9.txt</td><td>1710174270056F5forCTG.txt.gz</td><td>0.1455</td><td>0.04427</td><td>3.286</td><td>0.001015</td><td>0.01904</td><td>0.002167</td><td>1.02</td><td>0.01269</td><td>0.01061</td><td>0.008259</td><td>Illnesses of mother: Diabetes</td><td>FALSE</td><td></td><td></td><td></td><td>331142</td><td>30772</td><td>300370</td><td>UK Biobank</td><td>https://docs.google.com/spreadsheets/d/1kvPoupSzsSFBNSztMzl04xMoSC3Kcx3CrjVf4yBmESU/edit?ts=5b5f17db#gid=227859291</td><td>PHESANT Transformation:20110_0 || CAT-MUL-BINARY-VAR 9 || NO_NAN Remove NA participants 6165 || Removed 23887 examples != 9 but with missing value (<0) || sample 300370/30772(331142) ||-Notes:This field contains the combined results of 2 ACE touchscreen questions, both asked Has/did your mother ever suffer from? (You can select more than one answer) for different sets of illnesses. For convenience, the illnesses were arbitrarily divided into two sets: Group 1 : Heart disease, Stroke, High blood pressure, Chronic bronchitis/emphysema, Alzheimer's disease/dementia, Diabetes. Group 2 : Parkinson's disease, Severe Depression, Lung cancer, Bowel cancer, Breast cancer. If any of codes -11, -13 or -17 were selected then no additional Group 1 choices were allowed. If any of codes -21, -23 or -27 were selected then no additional Group 2 choices were allowed. If the participant activated the Help button they were shown the message: Answer this question for blood relations only. If you are not sure if your mother suffered from any of the listed illnesses please select Do not know. If you know your mother suffered from certain listed illnesses but are unsure about others, only select the ones you are sure about. The information was collected from participants who indicated they were not adopted as a child, as defined by their answers to ~F1767~ and who indicated whether their natural mother was still alive or had died, as defined by their answers to ~F1835~. Note that although Prostate cancer is present in ~C1010~, participants were not presented with this option when selecting Mother illnesses.-Variable type:binary</td></tr>
<tr><td>binary.20111_1.txt</td><td>1710174270056F5forCTG.txt.gz</td><td>0.3406</td><td>0.05996</td><td>5.681</td><td>1.34E-08</td><td>0.01537</td><td>0.002583</td><td>1.022</td><td>0.01068</td><td>0.003538</td><td>0.008689</td><td>Illnesses of siblings: Heart disease</td><td>FALSE</td><td>Cardiac</td><td></td><td></td><td>280784</td><td>28696</td><td>252088</td><td>UK Biobank</td><td>https://docs.google.com/spreadsheets/d/1kvPoupSzsSFBNSztMzl04xMoSC3Kcx3CrjVf4yBmESU/edit?ts=5b5f17db#gid=227859291</td><td>PHESANT Transformation:20111_0 || CAT-MUL-BINARY-VAR 1 || NO_NAN Remove NA participants 52692 || Removed 27718 examples != 1 but with missing value (<0) || sample 252088/28696(280784) ||-Notes:This field contains the combined results of 2 ACE touchscreen questions, both asked Have any of your brothers or sisters suffered from any of the following diseases? (You can select more than one answer) for different sets of illnesses. For convenience, the illnesses were arbitrarily divided into two sets: Group 1 : Heart disease, Stroke, High blood pressure, Chronic bronchitis/emphysema, Alzheimer's disease/dementia, Diabetes. Group 2 : Parkinson's disease, Severe Depression, Lung cancer, Bowel cancer, Prostate cancer, Breast cancer. If any of codes -11, -13 or -17 were selected then no additional Group 1 choices were allowed. If any of codes -21, -23 or -27 were selected then no additional Group 2 choices were allowed. If the participant activated the Help button they were shown the message: Answer this question for blood relations only. Include any sisters or brothers who have died. If you are not sure if your sisters or brothers suffered from any of the listed illnesses please select Do not know. If more than one sister or brother has suffered from any of the listed illnesses, you only need to select the illness once. The information was collected from participants who indicated they were not adopted as a child, as defined by their answers to ~F1767~ and that they had at least one natural brother or sister, as defined by their answers to ~F1873~ and ~F1883~.-Variable type:binary</td></tr>
<tr><td>binary.20111_100.txt</td><td>1710174270056F5forCTG.txt.gz</td><td>-0.3459</td><td>0.0437</td><td>-7.915</td><td>2.48E-15</td><td>0.03555</td><td>0.002859</td><td>1.019</td><td>0.01191</td><td>-0.000586</td><td>0.008977</td><td>Illnesses of siblings: None of the above (group 1)</td><td>FALSE</td><td>Other</td><td></td><td></td><td>281979</td><td>190969</td><td>91010</td><td>UK Biobank</td><td>https://docs.google.com/spreadsheets/d/1kvPoupSzsSFBNSztMzl04xMoSC3Kcx3CrjVf4yBmESU/edit?ts=5b5f17db#gid=227859291</td><td>PHESANT Transformation:20111_0 || CAT-MUL-BINARY-VAR 100 || NO_NAN Remove NA participants 52692 || Removed 26523 examples != 100 but with missing value (<0) || sample 91010/190969(281979) ||-Notes:This field contains the combined results of 2 ACE touchscreen questions, both asked Have any of your brothers or sisters suffered from any of the following diseases? (You can select more than one answer) for different sets of illnesses. For convenience, the illnesses were arbitrarily divided into two sets: Group 1 : Heart disease, Stroke, High blood pressure, Chronic bronchitis/emphysema, Alzheimer's disease/dementia, Diabetes. Group 2 : Parkinson's disease, Severe Depression, Lung cancer, Bowel cancer, Prostate cancer, Breast cancer. If any of codes -11, -13 or -17 were selected then no additional Group 1 choices were allowed. If any of codes -21, -23 or -27 were selected then no additional Group 2 choices were allowed. If the participant activated the Help button they were shown the message: Answer this question for blood relations only. Include any sisters or brothers who have died. If you are not sure if your sisters or brothers suffered from any of the listed illnesses please select Do not know. If more than one sister or brother has suffered from any of the listed illnesses, you only need to select the illness once. The information was collected from participants who indicated they were not adopted as a child, as defined by their answers to ~F1767~ and that they had at least one natural brother or sister, as defined by their answers to ~F1873~ and ~F1883~.-Variable type:binary</td></tr>
<tr><td>binary.20111_101.txt</td><td>1710174270056F5forCTG.txt.gz</td><td>-0.3727</td><td>0.06746</td><td>-5.525</td><td>3.29E-08</td><td>0.01028</td><td>0.002053</td><td>0.992</td><td>0.009879</td><td>0.004578</td><td>0.00795</td><td>Illnesses of siblings: None of the above (group 2)</td><td>FALSE</td><td>Other</td><td></td><td></td><td>284896</td><td>240056</td><td>44840</td><td>UK Biobank</td><td>https://docs.google.com/spreadsheets/d/1kvPoupSzsSFBNSztMzl04xMoSC3Kcx3CrjVf4yBmESU/edit?ts=5b5f17db#gid=227859291</td><td>PHESANT Transformation:20111_0 || CAT-MUL-BINARY-VAR 101 || NO_NAN Remove NA participants 52692 || Removed 23607 examples != 101 but with missing value (<0) || sample 44840/240056(284896) || SKIP_val:-21 < 0 || SKIP_val:-23 < 0 ||-Notes:This field contains the combined results of 2 ACE touchscreen questions, both asked Have any of your brothers or sisters suffered from any of the following diseases? (You can select more than one answer) for different sets of illnesses. For convenience, the illnesses were arbitrarily divided into two sets: Group 1 : Heart disease, Stroke, High blood pressure, Chronic bronchitis/emphysema, Alzheimer's disease/dementia, Diabetes. Group 2 : Parkinson's disease, Severe Depression, Lung cancer, Bowel cancer, Prostate cancer, Breast cancer. If any of codes -11, -13 or -17 were selected then no additional Group 1 choices were allowed. If any of codes -21, -23 or -27 were selected then no additional Group 2 choices were allowed. If the participant activated the Help button they were shown the message: Answer this question for blood relations only. Include any sisters or brothers who have died. If you are not sure if your sisters or brothers suffered from any of the listed illnesses please select Do not know. If more than one sister or brother has suffered from any of the listed illnesses, you only need to select the illness once. The information was collected from participants who indicated they were not adopted as a child, as defined by their answers to ~F1767~ and that they had at least one natural brother or sister, as defined by their answers to ~F1873~ and ~F1883~.-Variable type:binary</td></tr>
</table>

<table>
<tr><td>binary.20111_12.txt</td><td>1710174270056F5forCTG.txt.gz</td><td>0.3229</td><td>0.05328</td><td>6.059</td><td>1.37E-09</td><td>0.01653</td><td>0.002027</td><td>0.994</td><td>0.009419</td><td>-0.01815</td><td>0.008136</td><td>Illnesses of siblings: Severe depression</td><td>FALSE</td><td>Psychiatric</td><td></td><td></td><td>279858</td><td>20107</td><td>259751</td><td>UK Biobank</td><td>https://docs.google.com/spreadsheets/d/1kvPoupSzsSFBNSztMzl04xMoSC3Kcx3CrjVf4yBmESU/edit?ts=5b5f17db#gid=227859291</td><td>PHESANT Transformation:20111_0 | | CAT-MUL-BINARY-VAR 12 | | NO_NAN Remove NA participants 52692 | | Removed 28645 examples != 12 but with missing value (<0) | | sample 259751/20107(279858) | |-Notes:This field contains the combined results of 2 ACE touchscreen questions, both asked Have any of your brothers or sisters suffered from any of the following diseases? (You can select more than one answer) for different sets of illnesses. For convenience, the illnesses were arbitrarily divided into two sets: Group 1 : Heart disease, Stroke, High blood pressure, Chronic bronchitis/emphysema, Alzheimer's disease/dementia, Diabetes. Group 2 : Parkinson's disease, Severe Depression, Lung cancer, Bowel cancer, Prostate cancer, Breast cancer. If any of codes -11, -13 or -17 were selected then no additional Group 1 choices were allowed. If any of codes -21, -23 or -27 were selected then no additional Group 2 choices were allowed. If the participant activated the Help button they were shown the message: Answer this question for blood relations only. Include any sisters or brothers who have died. If you are not sure if your sisters or brothers suffered from any of the listed illnesses please select Do not know. If more than one sister or brother has suffered from any of the listed illnesses, you only need to select the illness once. The information was collected from participants who indicated they were not adopted as a child, as defined by their answers to ~F1767~ and that they had at least one natural brother or sister, as defined by their answers to ~F1873~ and ~F1883~.-Variable type:binary</td></tr>
<tr><td>binary.20111_13.txt</td><td>1710174270056F5forCTG.txt.gz</td><td>0.02176</td><td>0.1108</td><td>0.1964</td><td>0.8443</td><td>0.003031</td><td>0.001812</td><td>0.992</td><td>0.009418</td><td>0.01167</td><td>0.007409</td><td>Illnesses of siblings: Prostate cancer</td><td>FALSE</td><td></td><td></td><td></td><td>279171</td><td>4518</td><td>274653</td><td>UK Biobank</td><td>https://docs.google.com/spreadsheets/d/1kvPoupSzsSFBNSztMzl04xMoSC3Kcx3CrjVf4yBmESU/edit?ts=5b5f17db#gid=227859291</td><td>PHESANT Transformation:20111_0 | | CAT-MUL-BINARY-VAR 13 | | NO_NAN Remove NA participants 52692 | | Removed 29333 examples != 13 but with missing value (<0) | | sample 274653/4518(279171) | |-Notes:This field contains the combined results of 2 ACE touchscreen questions, both asked Have any of your brothers or sisters suffered from any of the following diseases? (You can select more than one answer) for different sets of illnesses. For convenience, the illnesses were arbitrarily divided into two sets: Group 1 : Heart disease, Stroke, High blood pressure, Chronic bronchitis/emphysema, Alzheimer's disease/dementia, Diabetes. Group 2 : Parkinson's disease, Severe Depression, Lung cancer, Bowel cancer, Prostate cancer, Breast cancer. If any of codes -11, -13 or -17 were selected then no additional Group 1 choices were allowed. If any of codes -21, -23 or -27 were selected then no additional Group 2 choices were allowed. If the participant activated the Help button they were shown the message: Answer this question for blood relations only. Include any sisters or brothers who have died. If you are not sure if your sisters or brothers suffered from any of the listed illnesses please select Do not know. If more than one sister or brother has suffered from any of the listed illnesses, you only need to select the illness once. The information was collected from participants who indicated they were not adopted as a child, as defined by their answers to ~F1767~ and that they had at least one natural brother or sister, as defined by their answers to ~F1873~ and ~F1883~.-Variable type:binary</td></tr>
<tr><td>binary.20111_2.txt</td><td>1710174270056F5forCTG.txt.gz</td><td>0.3555</td><td>0.1525</td><td>2.331</td><td>0.01975</td><td>0.00303</td><td>0.001956</td><td>1.004</td><td>0.009357</td><td>0.001335</td><td>0.007118</td><td>Illnesses of siblings: Stroke</td><td>FALSE</td><td></td><td></td><td></td><td>279577</td><td>9197</td><td>270380</td><td>UK Biobank</td><td>https://docs.google.com/spreadsheets/d/1kvPoupSzsSFBNSztMzl04xMoSC3Kcx3CrjVf4yBmESU/edit?ts=5b5f17db#gid=227859291</td><td>PHESANT Transformation:20111_0 | | CAT-MUL-BINARY-VAR 2 | | NO_NAN Remove NA participants 52692 | | Removed 28925 examples != 2 but with missing value (<0) | | sample 270380/9197(279577) | |-Notes:This field contains the combined results of 2 ACE touchscreen questions, both asked Have any of your brothers or sisters suffered from any of the following diseases? (You can select more than one answer) for different sets of illnesses. For convenience, the illnesses were arbitrarily divided into two sets: Group 1 : Heart disease, Stroke, High blood pressure, Chronic bronchitis/emphysema, Alzheimer's disease/dementia, Diabetes. Group 2 : Parkinson's disease, Severe Depression, Lung cancer, Bowel cancer, Prostate cancer, Breast cancer. If any of codes -11, -13 or -17 were selected then no additional Group 1 choices were allowed. If any of codes -21, -23 or -27 were selected then no additional Group 2 choices were allowed. If the participant activated the Help button they were shown the message: Answer this question for blood relations only. Include any sisters or brothers who have died. If you are not sure if your sisters or brothers suffered from any of the listed illnesses please select Do not know. If more than one sister or brother has suffered from any of the listed illnesses, you only need to select the illness once. The information was collected from participants who indicated they were not adopted as a child, as defined by their answers to ~F1767~ and that they had at least one natural brother or sister, as defined by their answers to ~F1873~ and ~F1883~.-Variable type:binary</td></tr>
<tr><td>binary.20111_3.txt</td><td>1710174270056F5forCTG.txt.gz</td><td>0.3528</td><td>0.1237</td><td>2.851</td><td>0.004355</td><td>0.004306</td><td>0.002338</td><td>1.001</td><td>0.009984</td><td>-0.00389</td><td>0.007912</td><td>Illnesses of siblings: Lung cancer</td><td>FALSE</td><td></td><td></td><td></td><td>279301</td><td>6189</td><td>273112</td><td>UK Biobank</td><td>https://docs.google.com/spreadsheets/d/1kvPoupSzsSFBNSztMzl04xMoSC3Kcx3CrjVf4yBmESU/edit?ts=5b5f17db#gid=227859291</td><td>PHESANT Transformation:20111_0 | | CAT-MUL-BINARY-VAR 3 | | NO_NAN Remove NA participants 52692 | | Removed 29201 examples != 3 but with missing value (<0) | | sample 273112/6189(279301) | |-Notes:This field contains the combined results of 2 ACE touchscreen questions, both asked Have any of your brothers or sisters suffered from any of the following diseases? (You can select more than one answer) for different sets of illnesses. For convenience, the illnesses were arbitrarily divided into two sets: Group 1 : Heart disease, Stroke, High blood pressure, Chronic bronchitis/emphysema, Alzheimer's disease/dementia, Diabetes. Group 2 : Parkinson's disease, Severe Depression, Lung cancer, Bowel cancer, Prostate cancer, Breast cancer. If any of codes -11, -13 or -17 were selected then no additional Group 1 choices were allowed. If any of codes -21, -23 or -27 were selected then no additional Group 2 choices were allowed. If the participant activated the Help button they were shown the message: Answer this question for blood relations only. Include any sisters or brothers who have died. If you are not sure if your sisters or brothers suffered from any of the listed illnesses please select Do not know. If more than one sister or brother has suffered from any of the listed illnesses, you only need to select the illness once. The information was collected from participants who indicated they were not adopted as a child, as defined by their answers to ~F1767~ and that they had at least one natural brother or sister, as defined by their answers to ~F1873~ and ~F1883~.-Variable type:binary</td></tr>
<tr><td>binary.20111_4.txt</td><td>1710174270056F5forCTG.txt.gz</td><td>0.1279</td><td>0.1034</td><td>1.236</td><td>0.2164</td><td>0.004156</td><td>0.001879</td><td>1</td><td>0.00884</td><td>0.001797</td><td>0.007787</td><td>Illnesses of siblings: Bowel cancer</td><td>FALSE</td><td></td><td></td><td></td><td>279242</td><td>6727</td><td>272515</td><td>UK Biobank</td><td>https://docs.google.com/spreadsheets/d/1kvPoupSzsSFBNSztMzl04xMoSC3Kcx3CrjVf4yBmESU/edit?ts=5b5f17db#gid=227859291</td><td>PHESANT Transformation:20111_0 | | CAT-MUL-BINARY-VAR 4 | | NO_NAN Remove NA participants 52692 | | Removed 29260 examples != 4 but with missing value (<0) | | sample 272515/6727(279242) | | SKIP_val:-13 < 0 | |-Notes:This field contains the combined results of 2 ACE touchscreen questions, both asked Have any of your brothers or sisters suffered from any of the following diseases? (You can select more than one answer) for different sets of illnesses. For convenience, the illnesses were arbitrarily divided into two sets: Group 1 : Heart disease, Stroke, High blood pressure, Chronic bronchitis/emphysema, Alzheimer's disease/dementia, Diabetes. Group 2 : Parkinson's disease, Severe Depression, Lung cancer, Bowel cancer, Prostate cancer, Breast cancer. If any of codes -11, -13 or -17 were selected then no additional Group 1 choices were allowed. If any of codes -21, -23 or -27 were selected then no additional Group 2 choices were allowed. If the participant activated the Help button they were shown the message: Answer this question for blood relations only. Include any sisters or brothers who have died. If you are not sure if your sisters or brothers suffered from any of the listed illnesses please select Do not know. If more than one sister or brother has suffered from any of the listed illnesses, you only need to select the illness once. The information was collected from participants who indicated they were not adopted as a child, as defined by their answers to ~F1767~ and that they had at least one natural brother or sister, as defined by their answers to ~F1873~ and ~F1883~.-Variable type:binary</td></tr>
</table>

<table>
<tr><th></th><th></th><th></th><th></th><th></th><th></th><th></th><th></th><th></th><th></th><th></th><th></th><th></th><th></th><th></th><th></th><th></th><th></th><th></th><th></th><th></th><th></th><th></th></tr>
<tr><td>binary.20111_5.txt</td><td>1710174270056F5forCTG.txt.gz</td><td>0.1035</td><td>0.09697</td><td>1.067</td><td>0.2859</td><td>0.004716</td><td>0.002097</td><td>1.006</td><td>0.00927</td><td>0.002932</td><td>0.008119</td><td>Illnesses of siblings: Breast cancer</td><td>FALSE</td><td></td><td></td><td></td><td>279478</td><td>12445</td><td>267033</td><td>UK Biobank</td><td>https://docs.google.com/spreadsheets/d/1kvPoupSzsSFBNSztMzl04xMoSC3Kcx3CrjVf4yBmESU/edit?ts=5b5f17db#gid=227859291</td><td>PHESANT Transformation:20111_0 | | CAT-MUL-BINARY-VAR 5 | | NO_NAN Remove NA participants 52692 | | Removed 29024 examples != 5 but with missing value (<0) | | sample 267033/12445(279478) | |-Notes:This field contains the combined results of 2 ACE touchscreen questions, both asked Have any of your brothers or sisters suffered from any of the following diseases? (You can select more than one answer) for different sets of illnesses. For convenience, the illnesses were arbitrarily divided into two sets: Group 1 : Heart disease, Stroke, High blood pressure, Chronic bronchitis/emphysema, Alzheimer's disease/dementia, Diabetes. Group 2 : Parkinson's disease, Severe Depression, Lung cancer, Bowel cancer, Prostate cancer, Breast cancer. If any of codes -11, -13 or -17 were selected then no additional Group 1 choices were allowed. If any of codes -21, -23 or -27 were selected then no additional Group 2 choices were allowed. If the participant activated the Help button they were shown the message: Answer this question for blood relations only. Include any sisters or brothers who have died. If you are not sure if your sisters or brothers suffered from any of the listed illnesses please select Do not know. If more than one sister or brother has suffered from any of the listed illnesses, you only need to select the illness once. The information was collected from participants who indicated they were not adopted as a child, as defined by their answers to ~F1767~ and that they had at least one natural brother or sister, as defined by their answers to ~F1873~ and ~F1883~.-Variable type:binary</td></tr>
<tr><td>binary.20111_6.txt</td><td>1710174270056F5forCTG.txt.gz</td><td>0.589</td><td>0.1825</td><td>3.227</td><td>0.00125</td><td>0.003825</td><td>0.002129</td><td>1.018</td><td>0.01025</td><td>0.004457</td><td>0.007703</td><td>Illnesses of siblings: Chronic bronchitis/emphysema</td><td>FALSE</td><td></td><td></td><td></td><td>279496</td><td>7933</td><td>271563</td><td>UK Biobank</td><td>https://docs.google.com/spreadsheets/d/1kvPoupSzsSFBNSztMzl04xMoSC3Kcx3CrjVf4yBmESU/edit?ts=5b5f17db#gid=227859291</td><td>PHESANT Transformation:20111_0 | | CAT-MUL-BINARY-VAR 6 | | NO_NAN Remove NA participants 52692 | | Removed 29007 examples != 6 but with missing value (<0) | | sample 271563/7933(279496) | | SKIP_val:-11 < 0 | |-Notes:This field contains the combined results of 2 ACE touchscreen questions, both asked Have any of your brothers or sisters suffered from any of the following diseases? (You can select more than one answer) for different sets of illnesses. For convenience, the illnesses were arbitrarily divided into two sets: Group 1 : Heart disease, Stroke, High blood pressure, Chronic bronchitis/emphysema, Alzheimer's disease/dementia, Diabetes. Group 2 : Parkinson's disease, Severe Depression, Lung cancer, Bowel cancer, Prostate cancer, Breast cancer. If any of codes -11, -13 or -17 were selected then no additional Group 1 choices were allowed. If any of codes -21, -23 or -27 were selected then no additional Group 2 choices were allowed. If the participant activated the Help button they were shown the message: Answer this question for blood relations only. Include any sisters or brothers who have died. If you are not sure if your sisters or brothers suffered from any of the listed illnesses please select Do not know. If more than one sister or brother has suffered from any of the listed illnesses, you only need to select the illness once. The information was collected from participants who indicated they were not adopted as a child, as defined by their answers to ~F1767~ and that they had at least one natural brother or sister, as defined by their answers to ~F1873~ and ~F1883~.-Variable type:binary</td></tr>
<tr><td>binary.20111_8.txt</td><td>1710174270056F5forCTG.txt.gz</td><td>0.2342</td><td>0.04219</td><td>5.552</td><td>2.83E-08</td><td>0.03579</td><td>0.002988</td><td>1.009</td><td>0.01222</td><td>0.0005825</td><td>0.008193</td><td>Illnesses of siblings: High blood pressure</td><td>FALSE</td><td>Cardiac</td><td></td><td></td><td>281619</td><td>58495</td><td>223124</td><td>UK Biobank</td><td>https://docs.google.com/spreadsheets/d/1kvPoupSzsSFBNSztMzl04xMoSC3Kcx3CrjVf4yBmESU/edit?ts=5b5f17db#gid=227859291</td><td>PHESANT Transformation:20111_0 | | CAT-MUL-BINARY-VAR 8 | | NO_NAN Remove NA participants 52692 | | Removed 26883 examples != 8 but with missing value (<0) | | sample 223124/58495(281619) | |-Notes:This field contains the combined results of 2 ACE touchscreen questions, both asked Have any of your brothers or sisters suffered from any of the following diseases? (You can select more than one answer) for different sets of illnesses. For convenience, the illnesses were arbitrarily divided into two sets: Group 1 : Heart disease, Stroke, High blood pressure, Chronic bronchitis/emphysema, Alzheimer's disease/dementia, Diabetes. Group 2 : Parkinson's disease, Severe Depression, Lung cancer, Bowel cancer, Prostate cancer, Breast cancer. If any of codes -11, -13 or -17 were selected then no additional Group 1 choices were allowed. If any of codes -21, -23 or -27 were selected then no additional Group 2 choices were allowed. If the participant activated the Help button they were shown the message: Answer this question for blood relations only. Include any sisters or brothers who have died. If you are not sure if your sisters or brothers suffered from any of the listed illnesses please select Do not know. If more than one sister or brother has suffered from any of the listed illnesses, you only need to select the illness once. The information was collected from participants who indicated they were not adopted as a child, as defined by their answers to ~F1767~ and that they had at least one natural brother or sister, as defined by their answers to ~F1873~ and ~F1883~.-Variable type:binary</td></tr>
<tr><td>binary.20111_9.txt</td><td>1710174270056F5forCTG.txt.gz</td><td>0.3754</td><td>0.05078</td><td>7.392</td><td>1.45E-13</td><td>0.0197</td><td>0.002519</td><td>1.009</td><td>0.0104</td><td>-0.01803</td><td>0.008223</td><td>Illnesses of siblings: Diabetes</td><td>FALSE</td><td>Metabolic</td><td></td><td></td><td>280211</td><td>23446</td><td>256765</td><td>UK Biobank</td><td>https://docs.google.com/spreadsheets/d/1kvPoupSzsSFBNSztMzl04xMoSC3Kcx3CrjVf4yBmESU/edit?ts=5b5f17db#gid=227859291</td><td>PHESANT Transformation:20111_0 | | CAT-MUL-BINARY-VAR 9 | | NO_NAN Remove NA participants 52692 | | Removed 28292 examples != 9 but with missing value (<0) | | sample 256765/23446(280211) | |-Notes:This field contains the combined results of 2 ACE touchscreen questions, both asked Have any of your brothers or sisters suffered from any of the following diseases? (You can select more than one answer) for different sets of illnesses. For convenience, the illnesses were arbitrarily divided into two sets: Group 1 : Heart disease, Stroke, High blood pressure, Chronic bronchitis/emphysema, Alzheimer's disease/dementia, Diabetes. Group 2 : Parkinson's disease, Severe Depression, Lung cancer, Bowel cancer, Prostate cancer, Breast cancer. If any of codes -11, -13 or -17 were selected then no additional Group 1 choices were allowed. If any of codes -21, -23 or -27 were selected then no additional Group 2 choices were allowed. If the participant activated the Help button they were shown the message: Answer this question for blood relations only. Include any sisters or brothers who have died. If you are not sure if your sisters or brothers suffered from any of the listed illnesses please select Do not know. If more than one sister or brother has suffered from any of the listed illnesses, you only need to select the illness once. The information was collected from participants who indicated they were not adopted as a child, as defined by their answers to ~F1767~ and that they had at least one natural brother or sister, as defined by their answers to ~F1873~ and ~F1883~.-Variable type:binary</td></tr>
<tr><td>binary.20114_101.txt</td><td>1710174270056F5forCTG.txt.gz</td><td>0.08823</td><td>0.08889</td><td>0.9926</td><td>0.3209</td><td>0.7701</td><td>0.2839</td><td>0.98</td><td>0.00893</td><td>-0.008166</td><td>0.008066</td><td>Illnesses of adopted siblings: None of the above (group 2)</td><td>FALSE</td><td></td><td></td><td></td><td>1869</td><td>1577</td><td>292</td><td>UK Biobank</td><td>https://docs.google.com/spreadsheets/d/1kvPoupSzsSFBNSztMzl04xMoSC3Kcx3CrjVf4yBmESU/edit?ts=5b5f17db#gid=227859291</td><td>PHESANT Transformation:20114_0 | | CAT-MUL-BINARY-VAR 101 | | NO_NAN Remove NA participants 358593 | | Removed 733 examples != 101 but with missing value (<0) | | sample 292/1577(1869) | | SKIP_val:-23 < 0 | |-Notes:This field contains the combined results of 2 ACE touchscreen questions, both asked Have any of your ADOPTED brothers or sisters suffered from any of the following diseases? (You can select more than one answer) for different sets of illnesses. For convenience, the illnesses were arbitrarily divided into two sets: Group 1 : Heart disease, Stroke, High blood pressure, Chronic bronchitis/emphysema, Alzheimer's disease/dementia, Diabetes. Group 2 : Parkinson's disease, Severe Depression, Lung cancer, Bowel cancer, Prostate cancer, Breast cancer. If any of codes -11, -13 or -17 were selected then no additional Group 1 choices were allowed. If any of codes -21, -23 or -27 were selected then no additional Group 2 choices were allowed. If the participant activated the Help button they were shown the message: Answer this question for adopted brothers and sisters only. Include any adopted sisters or brothers who have died. If you are not sure if your adopted sisters or brothers suffered from any of the listed illnesses please select Do not know. If more than one adopted sister or brother has suffered from any of the listed illnesses, you only need to select the illness once. The information was collected from participants who indicated they were adopted as a child, as defined by their answers to ~F1767~ and that they had at least one adopted brother or sister, as defined by their answers to ~F3972~ and ~F3982~.-Variable type:binary</td></tr>
<tr><td>binary.20116_0.txt</td><td>1710174270056F5forCTG.txt.gz</td><td>-0.2085</td><td>0.02791</td><td>-7.47</td><td>8.03E-14</td><td>0.08944</td><td>0.004301</td><td>1.047</td><td>0.01837</td><td>-0.008707</td><td>0.01003</td><td>Smoking status: Never</td><td>FALSE</td><td>Lifestyle</td><td></td><td></td><td>359706</td><td>195068</td><td>164638</td><td>UK Biobank</td><td>https://docs.google.com/spreadsheets/d/1kvPoupSzsSFBNSztMzl04xMoSC3Kcx3CrjVf4yBmESU/edit?ts=5b5f17db#gid=227859291</td><td>PHESANT Transformation:20116_0 | | CAT-SINGLE | | CAT-SINGLE-BINARY-VAR: 0 | | Inc(>=10): 0(195068) | |-Notes:This field summarises the current/past smoking status of the participant.-Variable type:binary</td></tr>
<tr><td>binary.20116_1.txt</td><td>1710174270056F5forCTG.txt.gz</td><td>0.1082</td><td>0.03416</td><td>3.168</td><td>0.001532</td><td>0.04924</td><td>0.003142</td><td>1.027</td><td>0.01475</td><td>0.005159</td><td>0.009478</td><td>Smoking status: Previous</td><td>FALSE</td><td></td><td></td><td></td><td>359706</td><td>127550</td><td>232156</td><td>UK Biobank</td><td>https://docs.google.com/spreadsheets/d/1kvPoupSzsSFBNSztMzl04xMoSC3Kcx3CrjVf4yBmESU/edit?ts=5b5f17db#gid=227859291</td><td>PHESANT Transformation:20116_0 | | CAT-SINGLE | | CAT-SINGLE-BINARY-VAR: 1 | | Inc(>=10): 1(127550) | |-Notes:This field summarises the current/past smoking status of the participant.-Variable type:binary</td></tr>
<tr><td>binary.20116_2.txt</td><td>1710174270056F5forCTG.txt.gz</td><td>0.2896</td><td>0.03127</td><td>9.261</td><td>2.03E-20</td><td>0.04759</td><td>0.002866</td><td>1.017</td><td>0.01389</td><td>0.007306</td><td>0.008495</td><td>Smoking status: Current</td><td>TRUE</td><td>Lifestyle</td><td></td><td>Current smoker</td><td>359706</td><td>37088</td><td>322618</td><td>UK Biobank</td><td>https://docs.google.com/spreadsheets/d/1kvPoupSzsSFBNSztMzl04xMoSC3Kcx3CrjVf4yBmESU/edit?ts=5b5f17db#gid=227859291</td><td>PHESANT Transformation:20116_0 | | CAT-SINGLE | | CAT-SINGLE-BINARY-VAR: 2 | | Inc(>=10): 2(37088) | |-Notes:This field summarises the current/past smoking status of the participant.-Variable type:binary</td></tr>
<tr><td>binary.20117_0.txt</td><td>1710174270056F5forCTG.txt.gz</td><td>0.1948</td><td>0.05324</td><td>3.66</td><td>0.000253</td><td>0.01451</td><td>0.001986</td><td>1.017</td><td>0.01179</td><td>0.003499</td><td>0.007754</td><td>Alcohol drinker status: Never</td><td>FALSE</td><td></td><td></td><td></td><td>360726</td><td>11243</td><td>349483</td><td>UK Biobank</td><td>https://docs.google.com/spreadsheets/d/1kvPoupSzsSFBNSztMzl04xMoSC3Kcx3CrjVf4yBmESU/edit?ts=5b5f17db#gid=227859291</td><td>PHESANT Transformation:20117_0 | | CAT-SINGLE | | CAT-SINGLE-BINARY-VAR: 0 | | Inc(>=10): 0(11243) | |-Notes:-Variable type:binary</td></tr>
</table>

<table>
<tr><td>binary.20117_1.txt</td><td>1710174270056F5forCTG.txt.gz</td><td>0.3295</td><td>0.05311</td><td>6.205</td><td>5.47E-10</td><td>0.0118</td><td>0.001716</td><td>1.009</td><td>0.01056</td><td>0.01806</td><td>0.008085</td><td>Alcohol drinker status: Previous</td><td>FALSE</td><td>Lifestyle</td><td></td><td></td><td>360726</td><td>12564</td><td>348162</td><td>UK Biobank</td><td>https://docs.google.com/spreadsheets/d/1kvPoupSzsSFBNSztMzl04xMoSC3Kcx3CrjVf4yBmESU/edit?ts=5b5f17db#gid=227859291</td><td>PHESANT Transformation:20117_0 | | CAT-SINGLE | | CAT-SINGLE-BINARY-VAR: 1 | | Inc(>=10): 1(12564) | | -Notes:-Variable type:binary</td></tr>
<tr><td>binary.20117_2.txt</td><td>1710174270056F5forCTG.txt.gz</td><td>-0.3187</td><td>0.04828</td><td>-6.6</td><td>4.12E-11</td><td>0.01829</td><td>0.002127</td><td>1.034</td><td>0.0119</td><td>-0.01561</td><td>0.008515</td><td>Alcohol drinker status: Current</td><td>TRUE</td><td>Lifestyle</td><td></td><td>Current alcohol drinker</td><td>360726</td><td>336919</td><td>23807</td><td>UK Biobank</td><td>https://docs.google.com/spreadsheets/d/1kvPoupSzsSFBNSztMzl04xMoSC3Kcx3CrjVf4yBmESU/edit?ts=5b5f17db#gid=227859291</td><td>PHESANT Transformation:20117_0 | | CAT-SINGLE | | CAT-SINGLE-BINARY-VAR: 2 | | Inc(>=10): 2(336919) | | -Notes:-Variable type:binary</td></tr>
<tr><td>binary.20118_11.txt</td><td>1710174270056F5forCTG.txt.gz</td><td>-0.3221</td><td>0.07848</td><td>-4.105</td><td>4.05E-05</td><td>0.008656</td><td>0.002308</td><td>1.257</td><td>0.01292</td><td>0.006557</td><td>0.009001</td><td>Home area population density - urban or rural: Scotland - Large Urban Area</td><td>FALSE</td><td></td><td></td><td></td><td>357712</td><td>20931</td><td>336781</td><td>UK Biobank</td><td>https://docs.google.com/spreadsheets/d/1kvPoupSzsSFBNSztMzl04xMoSC3Kcx3CrjVf4yBmESU/edit?ts=5b5f17db#gid=227859291</td><td>PHESANT Transformation:20118_0 | | CAT-SINGLE | | CAT-SINGLE-BINARY-VAR: 11 | | Inc(>=10): 11(20931) | | -Notes:The classification is derived by combining each participants home postcode with data generated from the 2001 census from the Office of National Statistics, using the Geoconvert tool from Census Dissemination Unit .-Variable type:binary</td></tr>
<tr><td>binary.20118_12.txt</td><td>1710174270056F5forCTG.txt.gz</td><td>0.0923</td><td>0.09127</td><td>1.011</td><td>0.3119</td><td>0.004881</td><td>0.001689</td><td>1.05</td><td>0.008727</td><td>0.008083</td><td>0.008733</td><td>Home area population density - urban or rural: Scotland - Other Urban Area</td><td>FALSE</td><td></td><td></td><td></td><td>357712</td><td>3998</td><td>353714</td><td>UK Biobank</td><td>https://docs.google.com/spreadsheets/d/1kvPoupSzsSFBNSztMzl04xMoSC3Kcx3CrjVf4yBmESU/edit?ts=5b5f17db#gid=227859291</td><td>PHESANT Transformation:20118_0 | | CAT-SINGLE | | CAT-SINGLE-BINARY-VAR: 12 | | Inc(>=10): 12(3998) | | -Notes:The classification is derived by combining each participants home postcode with data generated from the 2001 census from the Office of National Statistics, using the Geoconvert tool from Census Dissemination Unit .-Variable type:binary</td></tr>
<tr><td>binary.20118_5.txt</td><td>1710174270056F5forCTG.txt.gz</td><td>0.2504</td><td>0.09952</td><td>2.516</td><td>0.01187</td><td>0.005112</td><td>0.001755</td><td>1.128</td><td>0.009816</td><td>-0.001794</td><td>0.007914</td><td>Home area population density - urban or rural: England/Wales - Urban - less sparse</td><td>FALSE</td><td></td><td></td><td></td><td>357712</td><td>279000</td><td>78712</td><td>UK Biobank</td><td>https://docs.google.com/spreadsheets/d/1kvPoupSzsSFBNSztMzl04xMoSC3Kcx3CrjVf4yBmESU/edit?ts=5b5f17db#gid=227859291</td><td>PHESANT Transformation:20118_0 | | CAT-SINGLE | | CAT-SINGLE-BINARY-VAR: 5 | | Inc(>=10): 5(279000) | | -Notes:The classification is derived by combining each participants home postcode with data generated from the 2001 census from the Office of National Statistics, using the Geoconvert tool from Census Dissemination Unit .-Variable type:binary</td></tr>
<tr><td>binary.20118_8.txt</td><td>1710174270056F5forCTG.txt.gz</td><td>-0.04409</td><td>0.08644</td><td>-0.51</td><td>0.61</td><td>0.003717</td><td>0.001447</td><td>0.995</td><td>0.009485</td><td>0.001663</td><td>0.006999</td><td>Home area population density - urban or rural: England/Wales - Hamlet and Isolated Dwelling - less sparse</td><td>FALSE</td><td></td><td></td><td></td><td>357712</td><td>7954</td><td>349758</td><td>UK Biobank</td><td>https://docs.google.com/spreadsheets/d/1kvPoupSzsSFBNSztMzl04xMoSC3Kcx3CrjVf4yBmESU/edit?ts=5b5f17db#gid=227859291</td><td>PHESANT Transformation:20118_0 | | CAT-SINGLE | | CAT-SINGLE-BINARY-VAR: 8 | | Inc(>=10): 8(7954) | | -Notes:The classification is derived by combining each participants home postcode with data generated from the 2001 census from the Office of National Statistics, using the Geoconvert tool from Census Dissemination Unit .-Variable type:binary</td></tr>
<tr><td>binary.20126_0.txt</td><td>1710174270056F5forCTG.txt.gz</td><td>-0.2874</td><td>0.05212</td><td>-5.513</td><td>3.52E-08</td><td>0.06472</td><td>0.007331</td><td>0.998</td><td>0.0103</td><td>-0.005156</td><td>0.007877</td><td>Bipolar and major depression status: No Bipolar or Depression</td><td>FALSE</td><td>Psychiatric</td><td></td><td></td><td>86895</td><td>62825</td><td>24070</td><td>UK Biobank</td><td>https://docs.google.com/spreadsheets/d/1kvPoupSzsSFBNSztMzl04xMoSC3Kcx3CrjVf4yBmESU/edit?ts=5b5f17db#gid=227859291</td><td>PHESANT Transformation:20126_0 | | CAT-SINGLE | | CAT-SINGLE-BINARY-VAR: 0 | | Inc(>=10): 0(62825) | | -Notes:Bipolar and major depression status. This derived data field has come from Professor Jill Pell from the Institute of Health & Wellbeing, University of Glasgow. Methods on how these fields were derived can be found in the Additional Resources tab.-Variable type:binary</td></tr>
<tr><td>binary.20126_1.txt</td><td>1710174270056F5forCTG.txt.gz</td><td>0.1936</td><td>0.0973</td><td>1.989</td><td>0.04665</td><td>0.01507</td><td>0.006723</td><td>0.999</td><td>0.009946</td><td>-0.005118</td><td>0.008132</td><td>Bipolar and major depression status: Bipolar I Disorder</td><td>FALSE</td><td></td><td></td><td></td><td>86895</td><td>556</td><td>86339</td><td>UK Biobank</td><td>https://docs.google.com/spreadsheets/d/1kvPoupSzsSFBNSztMzl04xMoSC3Kcx3CrjVf4yBmESU/edit?ts=5b5f17db#gid=227859291</td><td>PHESANT Transformation:20126_0 | | CAT-SINGLE | | CAT-SINGLE-BINARY-VAR: 1 | | Inc(>=10): 1(556) | | -Notes:Bipolar and major depression status. This derived data field has come from Professor Jill Pell from the Institute of Health & Wellbeing, University of Glasgow. Methods on how these fields were derived can be found in the Additional Resources tab.-Variable type:binary</td></tr>
<tr><td>binary.20126_3.txt</td><td>1710174270056F5forCTG.txt.gz</td><td>0.2308</td><td>0.08175</td><td>2.823</td><td>0.004759</td><td>0.02558</td><td>0.007632</td><td>1.004</td><td>0.01028</td><td>0.01328</td><td>0.008127</td><td>Bipolar and major depression status: Probable Recurrent major depression (severe)</td><td>FALSE</td><td></td><td></td><td></td><td>86895</td><td>6304</td><td>80591</td><td>UK Biobank</td><td>https://docs.google.com/spreadsheets/d/1kvPoupSzsSFBNSztMzl04xMoSC3Kcx3CrjVf4yBmESU/edit?ts=5b5f17db#gid=227859291</td><td>PHESANT Transformation:20126_0 | | CAT-SINGLE | | CAT-SINGLE-BINARY-VAR: 3 | | Inc(>=10): 3(6304) | | -Notes:Bipolar and major depression status. This derived data field has come from Professor Jill Pell from the Institute of Health & Wellbeing, University of Glasgow. Methods on how these fields were derived can be found in the Additional Resources tab.-Variable type:binary</td></tr>
<tr><td>binary.20126_4.txt</td><td>1710174270056F5forCTG.txt.gz</td><td>0.2525</td><td>0.07637</td><td>3.306</td><td>0.000945</td><td>0.02727</td><td>0.006438</td><td>0.998</td><td>0.009281</td><td>0.004076</td><td>0.007776</td><td>Bipolar and major depression status: Probable Recurrent major depression (moderate)</td><td>FALSE</td><td></td><td></td><td></td><td>86895</td><td>10902</td><td>75993</td><td>UK Biobank</td><td>https://docs.google.com/spreadsheets/d/1kvPoupSzsSFBNSztMzl04xMoSC3Kcx3CrjVf4yBmESU/edit?ts=5b5f17db#gid=227859291</td><td>PHESANT Transformation:20126_0 | | CAT-SINGLE | | CAT-SINGLE-BINARY-VAR: 4 | | Inc(>=10): 4(10902) | | -Notes:Bipolar and major depression status. This derived data field has come from Professor Jill Pell from the Institute of Health & Wellbeing, University of Glasgow. Methods on how these fields were derived can be found in the Additional Resources tab.-Variable type:binary</td></tr>
<tr><td>binary.20152.txt</td><td>1710174270056F5forCTG.txt.gz</td><td>-0.01023</td><td>0.05821</td><td>-0.1757</td><td>0.8606</td><td>0.01399</td><td>0.00223</td><td>1</td><td>0.009215</td><td>-0.000668</td><td>0.007875</td><td>Reproduciblity of spirometry measurement using ERS/ATS criteria</td><td>FALSE</td><td></td><td></td><td></td><td>272338</td><td>212530</td><td>59808</td><td>UK Biobank</td><td>https://docs.google.com/spreadsheets/d/1kvPoupSzsSFBNSztMzl04xMoSC3Kcx3CrjVf4yBmESU/edit?ts=5b5f17db#gid=227859291</td><td>PHESANT Transformation:20152_0| | CAT-SINGLE | | reassignments: 9=NA | | Inc(>=10): 1(212530) | | Inc(>=10): 0(59808) | | CAT-SINGLE-BINARY | | sample 59808/212530(272338) | | -Notes:Indicates whether an individual's spirometry meets European Respiratory Society/American Thoracic Society (ERS/ATS) Criteria, that is their highest acceptable measure was reproducible (the highest acceptable measure and any other measure were within 150 millilitres for both for Forced Expiratory Volume in 1-second (FEV1) and Forced Vital Capacity (FVC), although not necessarily the same blow. Note, if FVC <=1 litre the criteria is within 100 millilitres). This derived data field has come from Professor Martin Tobin at the University of Leicester.-Variable type:binary</td></tr>
<tr><td>binary.20160.txt</td><td>1710174270056F5forCTG.txt.gz</td><td>0.1108</td><td>0.02993</td><td>3.703</td><td>0.000213</td><td>0.07061</td><td>0.003823</td><td>1.025</td><td>0.01721</td><td>0.01007</td><td>0.009464</td><td>Ever smoked</td><td>FALSE</td><td></td><td></td><td></td><td>359751</td><td>218191</td><td>141560</td><td>UK Biobank</td><td>https://docs.google.com/spreadsheets/d/1kvPoupSzsSFBNSztMzl04xMoSC3Kcx3CrjVf4yBmESU/edit?ts=5b5f17db#gid=227859291</td><td>PHESANT Transformation:20160_0| | CAT-SINGLE | | reassignments: 9=NA | | Inc(>=10): 1(218191) | | Inc(>=10): 0(141560) | | CAT-SINGLE-BINARY | | sample 141560/218191(359751) | | -Notes:Derived using variables Current tobacco smoking (~F1239~) and Past tobacco smoking (~F1249~). Individual classed as Eversmoker if Current tobacco smoking= most days (1) or occasionally (2) OR Past tobacco smoking= most days (1) or occasionally (2) or tried once or twice (3). Individual were classed as Neversmoker if Current tobacco smoking= no (0) AND Past tobacco smoking= never (4). Individuals who answered to either question Do not know (-1) Prefer not to answer (-3) and None of the above (-7) were not coded. This derived data field has come from Professor Martin Tobin at the University of Leicester.-Variable type:binary</td></tr>
<tr><td>binary.2020.txt</td><td>1710174270056F5forCTG.txt.gz</td><td>0.3582</td><td>0.03785</td><td>9.464</td><td>2.97E-21</td><td>0.03468</td><td>0.002542</td><td>1.028</td><td>0.01243</td><td>-0.004795</td><td>0.008308</td><td>Loneliness, isolation</td><td>TRUE</td><td>Psychiatric</td><td></td><td>Loneliness, isolation</td><td>355583</td><td>63508</td><td>292075</td><td>UK Biobank</td><td>https://docs.google.com/spreadsheets/d/1kvPoupSzsSFBNSztMzl04xMoSC3Kcx3CrjVf4yBmESU/edit?ts=5b5f17db#gid=227859291</td><td>PHESANT Transformation:2020_0| | CAT-SINGLE | | Inc(>=10): 0(292075) | | Inc(>=10): 1(63508) | | CAT-SINGLE-BINARY | | sample 292075/63508(355583) | | -Notes:ACE touchscreen question Do you often feel lonely? If the participant activated the Help button they were shown the message: Work through these questions quickly and do not think about the exact meaning of the question-Variable type:binary</td></tr>
<tr><td>binary.2030.txt</td><td>1710174270056F5forCTG.txt.gz</td><td>0.1409</td><td>0.035</td><td>4.024</td><td>5.72E-05</td><td>0.05255</td><td>0.002828</td><td>0.996</td><td>0.01275</td><td>-0.003444</td><td>0.00942</td><td>Guilty feelings</td><td>FALSE</td><td></td><td></td><td></td><td>351907</td><td>100128</td><td>251779</td><td>UK Biobank</td><td>https://docs.google.com/spreadsheets/d/1kvPoupSzsSFBNSztMzl04xMoSC3Kcx3CrjVf4yBmESU/edit?ts=5b5f17db#gid=227859291</td><td>PHESANT Transformation:2030_0| | CAT-SINGLE | | Inc(>=10): 0(251779) | | Inc(>=10): 1(100128) | | CAT-SINGLE-BINARY | | sample 251779/100128(351907) | | -Notes:ACE touchscreen question Are you often troubled by feelings of guilt? If the participant activated the Help button they were shown the message: Work through these questions quickly and do not think about the exact meaning of the question-Variable type:binary</td></tr>
<tr><td>binary.2040.txt</td><td>1710174270056F5forCTG.txt.gz</td><td>0.4031</td><td>0.03296</td><td>12.23</td><td>2.22E-34</td><td>0.052</td><td>0.003222</td><td>1.015</td><td>0.01447</td><td>0.004268</td><td>0.0101</td><td>Risk taking</td><td>TRUE</td><td>Psychiatric</td><td></td><td>Risk taking</td><td>348549</td><td>90500</td><td>258049</td><td>UK Biobank</td><td>https://docs.google.com/spreadsheets/d/1kvPoupSzsSFBNSztMzl04xMoSC3Kcx3CrjVf4yBmESU/edit?ts=5b5f17db#gid=227859291</td><td>PHESANT Transformation:2040_0| | CAT-SINGLE | | Inc(>=10): 0(258049) | | Inc(>=10): 1(90500) | | CAT-SINGLE-BINARY | | sample 258049/90500(348549) | | -Notes:ACE touchscreen question Would you describe yourself as someone who takes risks? If the participant activated the Help button they were shown the message: Work through these questions quickly and do not think about the exact meaning of the question-Variable type:binary</td></tr>
<tr><td>binary.20401.txt</td><td>1710174270056F5forCTG.txt.gz</td><td>0.1662</td><td>0.08441</td><td>1.969</td><td>0.0489</td><td>0.01811</td><td>0.005445</td><td>1.003</td><td>0.01027</td><td>-0.002106</td><td>0.008123</td><td>Ever addicted to any substance or behaviour</td><td>FALSE</td><td></td><td></td><td></td><td>116746</td><td>7023</td><td>109723</td><td>UK Biobank</td><td>https://docs.google.com/spreadsheets/d/1kvPoupSzsSFBNSztMzl04xMoSC3Kcx3CrjVf4yBmESU/edit?ts=5b5f17db#gid=227859291</td><td>PHESANT Transformation:20401_0| | CAT-SINGLE | | Inc(>=10): 0(109723) | | Inc(>=10): 1(7023) | | CAT-SINGLE-BINARY | | sample 109723/7023(116746) | | -Notes:Question asked: Have you been addicted to or dependent on one or more things, including substances (not cigarettes/coffee) or behaviours (such as gambling)?-Variable type:binary</td></tr>
<tr><td>binary.20405_0.txt</td><td>1710174270056F5forCTG.txt.gz</td><td>-0.02996</td><td>0.06863</td><td>-0.4365</td><td>0.6624</td><td>0.02969</td><td>0.005084</td><td>1.003</td><td>0.009709</td><td>-0.008026</td><td>0.007986</td><td>Ever had known person concerned about, or recommend reduction of, alcohol consumption: No</td><td>FALSE</td><td></td><td></td><td></td><td>117880</td><td>107474</td><td>10406</td><td>UK Biobank</td><td>https://docs.google.com/spreadsheets/d/1kvPoupSzsSFBNSztMzl04xMoSC3Kcx3CrjVf4yBmESU/edit?ts=5b5f17db#gid=227859291</td><td>PHESANT Transformation:20405_0 | | CAT-SINGLE | | CAT-SINGLE-BINARY-VAR: 0 | | Inc(>=10): 0(107474) | | -Notes:Question asked: Has a relative or friend or a doctor or another health worker been concerned about your drinking or suggested you cut down?-Variable type:binary</td></tr>
</table>

<table>
<tr><td>binary.20405_1.txt</td><td>1710174270056F5 forCTG.txt.gz</td><td>0.04647</td><td>0.08297</td><td>0.5601</td><td>0.5754</td><td>0.0151</td><td>0.004594</td><td>1.002</td><td>0.009638</td><td>0.007391</td><td>0.007235</td><td>Ever had known person concerned about, or recommend reduction of, alcohol consumption: Yes, but not in the last year</td><td>FALSE</td><td></td><td></td><td></td><td>117880</td><td>5482</td><td>112398</td><td>UK Biobank</td><td>https://docs.google.com/spreadsheets/d/1kvPoupSzsSFBNSztMzl04xMoSC3Kcx3CrjVf4yBmESU/edit?ts=5b5f17db#gid=227859291</td><td>PHESANT Transformation:20405_0 | | CAT-SINGLE | | CAT-SINGLE-BINARY-VAR: 1 | | Inc(>=10): 1(5482) | | -Notes:Question asked: Has a relative or friend or a doctor or another health worker been concerned about your drinking or suggested you cut down?-Variable type:binary</td></tr>
<tr><td>binary.20405_2.txt</td><td>1710174270056F5 forCTG.txt.gz</td><td>0.01057</td><td>0.08072</td><td>0.131</td><td>0.8958</td><td>0.01946</td><td>0.00514</td><td>1.001</td><td>0.01013</td><td>0.003363</td><td>0.008701</td><td>Ever had known person concerned about, or recommend reduction of, alcohol consumption: Yes, during the last year</td><td>FALSE</td><td></td><td></td><td></td><td>117880</td><td>4924</td><td>112956</td><td>UK Biobank</td><td>https://docs.google.com/spreadsheets/d/1kvPoupSzsSFBNSztMzl04xMoSC3Kcx3CrjVf4yBmESU/edit?ts=5b5f17db#gid=227859291</td><td>PHESANT Transformation:20405_0 | | CAT-SINGLE | | CAT-SINGLE-BINARY-VAR: 2 | | Inc(>=10): 2(4924) | | -Notes:Question asked: Has a relative or friend or a doctor or another health worker been concerned about your drinking or suggested you cut down?-Variable type:binary</td></tr>
<tr><td>binary.20406.txt</td><td>1710174270056F5 forCTG.txt.gz</td><td>-0.05134</td><td>0.09491</td><td>-0.541</td><td>0.5885</td><td>0.226</td><td>0.09016</td><td>0.996</td><td>0.009876</td><td>0.006683</td><td>0.007947</td><td>Ever addicted to alcohol</td><td>FALSE</td><td></td><td></td><td></td><td>6514</td><td>2778</td><td>3736</td><td>UK Biobank</td><td>https://docs.google.com/spreadsheets/d/1kvPoupSzsSFBNSztMzl04xMoSC3Kcx3CrjVf4yBmESU/edit?ts=5b5f17db#gid=227859291</td><td>PHESANT Transformation:20406_0| | CAT-SINGLE | | Inc(>=10): 1(2778) | | Inc(>=10): 0(3736) | | CAT-SINGLE-BINARY | | sample 3736/2778(6514) | | -Notes:Question asked: Have you been addicted to alcohol? Question was asked when ~F20401~ was Yes.-Variable type:binary</td></tr>
<tr><td>binary.20411_0.txt</td><td>1710174270056F5 forCTG.txt.gz</td><td>-0.024</td><td>0.1119</td><td>-0.2144</td><td>0.8302</td><td>0.01012</td><td>0.005097</td><td>1.01</td><td>0.01036</td><td>-0.0195</td><td>0.008548</td><td>Ever been injured or injured someone else through drinking alcohol: No</td><td>FALSE</td><td></td><td></td><td></td><td>118002</td><td>112909</td><td>5093</td><td>UK Biobank</td><td>https://docs.google.com/spreadsheets/d/1kvPoupSzsSFBNSztMzl04xMoSC3Kcx3CrjVf4yBmESU/edit?ts=5b5f17db#gid=227859291</td><td>PHESANT Transformation:20411_0 | | CAT-SINGLE | | CAT-SINGLE-BINARY-VAR: 0 | | Inc(>=10): 0(112909) | | -Notes:Question asked: Have you or someone else been injured as a result of your drinking?-Variable type:binary</td></tr>
<tr><td>binary.20417.txt</td><td>1710174270056F5 forCTG.txt.gz</td><td>0.3965</td><td>0.07845</td><td>5.055</td><td>4.31E-07</td><td>0.06431</td><td>0.01638</td><td>0.999</td><td>0.009453</td><td>0.004751</td><td>0.006983</td><td>Tense, sore, or aching muscles during worst period of anxiety</td><td>FALSE</td><td>Pain</td><td></td><td></td><td>33301</td><td>12996</td><td>20305</td><td>UK Biobank</td><td>https://docs.google.com/spreadsheets/d/1kvPoupSzsSFBNSztMzl04xMoSC3Kcx3CrjVf4yBmESU/edit?ts=5b5f17db#gid=227859291</td><td>PHESANT Transformation:20417_0| | CAT-SINGLE | | Inc(>=10): 1(12996) | | Inc(>=10): 0(20305) | | CAT-SINGLE-BINARY | | sample 20305/12996(33301) | | -Notes:Question asked: When you were worried or anxious, were you also: Having tense, sore, or aching muscles? Question was asked when ~F20425~ was Yes or ~F20420~ was at least 6 months.-Variable type:binary</td></tr>
<tr><td>binary.20419.txt</td><td>1710174270056F5 forCTG.txt.gz</td><td>0.347</td><td>0.1381</td><td>2.514</td><td>0.01195</td><td>0.02735</td><td>0.01539</td><td>1</td><td>0.008983</td><td>-0.002082</td><td>0.00837</td><td>Difficulty concentrating during worst period of anxiety</td><td>FALSE</td><td></td><td></td><td></td><td>34879</td><td>26400</td><td>8479</td><td>UK Biobank</td><td>https://docs.google.com/spreadsheets/d/1kvPoupSzsSFBNSztMzl04xMoSC3Kcx3CrjVf4yBmESU/edit?ts=5b5f17db#gid=227859291</td><td>PHESANT Transformation:20419_0| | CAT-SINGLE | | Inc(>=10): 1(26400) | | Inc(>=10): 0(8479) | | CAT-SINGLE-BINARY | | sample 8479/26400(34879) | | -Notes:Question asked: When you were worried or anxious, were you also: Having difficulty keeping your mind on what you were doing? Question was asked when ~F20425~ was Yes or ~F20420~ was at least 6 months.-Variable type:binary</td></tr>
<tr><td>binary.20421.txt</td><td>1710174270056F5 forCTG.txt.gz</td><td>0.2435</td><td>0.05028</td><td>4.842</td><td>1.28E-06</td><td>0.05637</td><td>0.006062</td><td>0.992</td><td>0.01044</td><td>-0.002871</td><td>0.008365</td><td>Ever felt worried, tense, or anxious for most of a month or longer</td><td>FALSE</td><td>Psychiatric</td><td></td><td></td><td>110315</td><td>29351</td><td>80964</td><td>UK Biobank</td><td>https://docs.google.com/spreadsheets/d/1kvPoupSzsSFBNSztMzl04xMoSC3Kcx3CrjVf4yBmESU/edit?ts=5b5f17db#gid=227859291</td><td>PHESANT Transformation:20421_0| | CAT-SINGLE | | Inc(>=10): 1(29351) | | Inc(>=10): 0(80964) | | CAT-SINGLE-BINARY | | sample 80964/29351(110315) | | -Notes:Question asked: Have you ever had a period lasting one month or longer when most of the time you felt worried, tense, or anxious?-Variable type:binary</td></tr>
<tr><td>binary.20422.txt</td><td>1710174270056F5 forCTG.txt.gz</td><td>0.4333</td><td>0.1254</td><td>3.455</td><td>0.000551</td><td>0.03906</td><td>0.01675</td><td>1.005</td><td>0.00976</td><td>-0.007459</td><td>0.007607</td><td>More irritable than usual during worst period of anxiety</td><td>FALSE</td><td></td><td></td><td></td><td>33425</td><td>24014</td><td>9411</td><td>UK Biobank</td><td>https://docs.google.com/spreadsheets/d/1kvPoupSzsSFBNSztMzl04xMoSC3Kcx3CrjVf4yBmESU/edit?ts=5b5f17db#gid=227859291</td><td>PHESANT Transformation:20422_0| | CAT-SINGLE | | Inc(>=10): 1(24014) | | Inc(>=10): 0(9411) | | CAT-SINGLE-BINARY | | sample 9411/24014(33425) | | -Notes:Question asked: When you were worried or anxious, were you also: More irritable than usual? Question was asked when ~F20425~ was Yes or ~F20420~ was at least 6 months.-Variable type:binary</td></tr>
<tr><td>binary.20425.txt</td><td>1710174270056F5 forCTG.txt.gz</td><td>0.1105</td><td>0.04997</td><td>2.212</td><td>0.02696</td><td>0.06554</td><td>0.007126</td><td>0.994</td><td>0.01107</td><td>-0.001573</td><td>0.008546</td><td>Ever worried more than most people would in similar situation</td><td>FALSE</td><td></td><td></td><td></td><td>98990</td><td>25623</td><td>73367</td><td>UK Biobank</td><td>https://docs.google.com/spreadsheets/d/1kvPoupSzsSFBNSztMzl04xMoSC3Kcx3CrjVf4yBmESU/edit?ts=5b5f17db#gid=227859291</td><td>PHESANT Transformation:20425_0| | CAT-SINGLE | | Inc(>=10): 0(73367) | | Inc(>=10): 1(25623) | | CAT-SINGLE-BINARY | | sample 73367/25623(98990) | | -Notes:Question asked: People differ a lot in how much they worry about things. Did you ever have a time when you worried a lot more than most people would in your situation?-Variable type:binary</td></tr>
<tr><td>binary.20426.txt</td><td>1710174270056F5 forCTG.txt.gz</td><td>0.3838</td><td>0.1341</td><td>2.861</td><td>0.004224</td><td>0.03319</td><td>0.01756</td><td>1</td><td>0.009342</td><td>0.002534</td><td>0.00805</td><td>Restless during period of worst anxiety</td><td>FALSE</td><td></td><td></td><td></td><td>33864</td><td>20328</td><td>13536</td><td>UK Biobank</td><td>https://docs.google.com/spreadsheets/d/1kvPoupSzsSFBNSztMzl04xMoSC3Kcx3CrjVf4yBmESU/edit?ts=5b5f17db#gid=227859291</td><td>PHESANT Transformation:20426_0| | CAT-SINGLE | | Inc(>=10): 1(20328) | | Inc(>=10): 0(13536) | | CAT-SINGLE-BINARY | | sample 13536/20328(33864) | | -Notes:Question asked: When you were worried or anxious, were you also: Restless? Question was asked when ~F20425~ was Yes or ~F20420~ was at least 6 months.-Variable type:binary</td></tr>
<tr><td>binary.20427.txt</td><td>1710174270056F5 forCTG.txt.gz</td><td>0.1823</td><td>0.08012</td><td>2.275</td><td>0.02288</td><td>0.06069</td><td>0.01642</td><td>0.982</td><td>0.008761</td><td>-0.005365</td><td>0.008524</td><td>Frequent trouble falling or staying asleep during worst period of anxiety</td><td>FALSE</td><td></td><td></td><td></td><td>35584</td><td>29129</td><td>6455</td><td>UK Biobank</td><td>https://docs.google.com/spreadsheets/d/1kvPoupSzsSFBNSztMzl04xMoSC3Kcx3CrjVf4yBmESU/edit?ts=5b5f17db#gid=227859291</td><td>PHESANT Transformation:20427_0| | CAT-SINGLE | | Inc(>=10): 1(29129) | | Inc(>=10): 0(6455) | | CAT-SINGLE-BINARY | | sample 6455/29129(35584) | | -Notes:Question asked: When you were worried or anxious, were you also: Often having trouble falling or staying asleep? Question was asked when ~F20425~ was Yes or ~F20420~ was at least 6 months.-Variable type:binary</td></tr>
<tr><td>binary.20428.txt</td><td>1710174270056F5 forCTG.txt.gz</td><td>0.05886</td><td>0.08266</td><td>0.7121</td><td>0.4764</td><td>0.05397</td><td>0.01459</td><td>0.984</td><td>0.008577</td><td>0.01814</td><td>0.007647</td><td>Professional informed about anxiety</td><td>FALSE</td><td></td><td></td><td></td><td>36366</td><td>22238</td><td>14128</td><td>UK Biobank</td><td>https://docs.google.com/spreadsheets/d/1kvPoupSzsSFBNSztMzl04xMoSC3Kcx3CrjVf4yBmESU/edit?ts=5b5f17db#gid=227859291</td><td>PHESANT Transformation:20428_0| | CAT-SINGLE | | Inc(>=10): 1(22238) | | Inc(>=10): 0(14128) | | CAT-SINGLE-BINARY | | sample 14128/22238(36366) | | -Notes:Question asked: Did you ever tell a professional about these problems (medical doctor, psychologist, social worker, counsellor, nurse, clergy, or other helping professional)? Question was asked when ~F20425~ was Yes or ~F20420~ was at least 6 months.-Variable type:binary</td></tr>
<tr><td>binary.20429.txt</td><td>1710174270056F5 forCTG.txt.gz</td><td>0.2894</td><td>0.09403</td><td>3.078</td><td>0.002083</td><td>0.05502</td><td>0.01456</td><td>0.997</td><td>0.007977</td><td>-0.002324</td><td>0.008453</td><td>Easily tired during worst period of anxiety</td><td>FALSE</td><td></td><td></td><td></td><td>34121</td><td>24544</td><td>9577</td><td>UK Biobank</td><td>https://docs.google.com/spreadsheets/d/1kvPoupSzsSFBNSztMzl04xMoSC3Kcx3CrjVf4yBmESU/edit?ts=5b5f17db#gid=227859291</td><td>PHESANT Transformation:20429_0| | CAT-SINGLE | | Inc(>=10): 1(24544) | | Inc(>=10): 0(9577) | | CAT-SINGLE-BINARY | | sample 9577/24544(34121) | | -Notes:Question asked: When you were worried or anxious, were you also: Easily tired? uestion was asked when ~F20425~ was Yes or ~F20420~ was at least 6 months.-Variable type:binary</td></tr>
<tr><td>binary.20435.txt</td><td>1710174270056F5 forCTG.txt.gz</td><td>0.3639</td><td>0.1023</td><td>3.557</td><td>0.000375</td><td>0.02998</td><td>0.009741</td><td>1.009</td><td>0.009698</td><td>-0.002286</td><td>0.008345</td><td>Difficulty concentrating during worst depression</td><td>FALSE</td><td></td><td></td><td></td><td>58750</td><td>46278</td><td>12472</td><td>UK Biobank</td><td>https://docs.google.com/spreadsheets/d/1kvPoupSzsSFBNSztMzl04xMoSC3Kcx3CrjVf4yBmESU/edit?ts=5b5f17db#gid=227859291</td><td>PHESANT Transformation:20435_0| | CAT-SINGLE | | Inc(>=10): 1(46278) | | Inc(>=10): 0(12472) | | CAT-SINGLE-BINARY | | sample 12472/46278(58750) | | -Notes:Question asked: Did you have a lot more trouble concentrating than usual? Question was asked when ~F20446~ was Yes or ~F20441~ was Yes.-Variable type:binary</td></tr>
<tr><td>binary.20437.txt</td><td>1710174270056F5 forCTG.txt.gz</td><td>0.5241</td><td>0.2424</td><td>2.162</td><td>0.03061</td><td>0.01141</td><td>0.008795</td><td>1.015</td><td>0.009717</td><td>-0.006105</td><td>0.007729</td><td>Thoughts of death during worst depression</td><td>FALSE</td><td></td><td></td><td></td><td>62648</td><td>32630</td><td>30018</td><td>UK Biobank</td><td>https://docs.google.com/spreadsheets/d/1kvPoupSzsSFBNSztMzl04xMoSC3Kcx3CrjVf4yBmESU/edit?ts=5b5f17db#gid=227859291</td><td>PHESANT Transformation:20437_0| | CAT-SINGLE | | Inc(>=10): 1(32630) | | Inc(>=10): 0(30018) | | CAT-SINGLE-BINARY | | sample 30018/32630(62648) | | -Notes:Question asked: Did you think a lot about death - either your own, someone else's or death in general? Question was asked when ~F20446~ was Yes or ~F20441~ was Yes.-Variable type:binary</td></tr>
<tr><td>binary.20441.txt</td><td>1710174270056F5 forCTG.txt.gz</td><td>0.3446</td><td>0.04557</td><td>7.562</td><td>3.98E-14</td><td>0.06384</td><td>0.005719</td><td>0.999</td><td>0.01092</td><td>-0.002459</td><td>0.00778</td><td>Ever had prolonged loss of interest in normal activities</td><td>FALSE</td><td>Psychiatric</td><td></td><td></td><td>117727</td><td>46330</td><td>71397</td><td>UK Biobank</td><td>https://docs.google.com/spreadsheets/d/1kvPoupSzsSFBNSztMzl04xMoSC3Kcx3CrjVf4yBmESU/edit?ts=5b5f17db#gid=227859291</td><td>PHESANT Transformation:20441_0| | CAT-SINGLE | | Inc(>=10): 1(46330) | | Inc(>=10): 0(71397) | | CAT-SINGLE-BINARY | | sample 71397/46330(117727) | | -Notes:Question asked: Have you ever had a time in your life lasting two weeks or more when you lost interest in most things like hobbies, work, or activities that usually give you pleasure?-Variable type:binary</td></tr>
<tr><td>binary.20446.txt</td><td>1710174270056F5 forCTG.txt.gz</td><td>0.3004</td><td>0.05371</td><td>5.592</td><td>2.24E-08</td><td>0.05612</td><td>0.005577</td><td>0.999</td><td>0.009486</td><td>-0.00092</td><td>0.008731</td><td>Ever had prolonged feelings of sadness or depression</td><td>FALSE</td><td>Psychiatric</td><td></td><td></td><td>117763</td><td>64374</td><td>53389</td><td>UK Biobank</td><td>https://docs.google.com/spreadsheets/d/1kvPoupSzsSFBNSztMzl04xMoSC3Kcx3CrjVf4yBmESU/edit?ts=5b5f17db#gid=227859291</td><td>PHESANT Transformation:20446_0| | CAT-SINGLE | | Inc(>=10): 1(64374) | | Inc(>=10): 0(53389) | | CAT-SINGLE-BINARY | | sample 53389/64374(117763) | | -Notes:Question asked: Have you ever had a time in your life when you felt sad, blue, or depressed for two weeks or more in a row?-Variable type:binary</td></tr>
<tr><td>binary.20447.txt</td><td>1710174270056F5 forCTG.txt.gz</td><td>0.2929</td><td>0.09743</td><td>3.006</td><td>0.002646</td><td>0.02126</td><td>0.008935</td><td>1.007</td><td>0.009746</td><td>-0.01383</td><td>0.006454</td><td>Depression possibly related to stressful or traumatic event</td><td>FALSE</td><td></td><td></td><td></td><td>66334</td><td>48490</td><td>17844</td><td>UK Biobank</td><td>https://docs.google.com/spreadsheets/d/1kvPoupSzsSFBNSztMzl04xMoSC3Kcx3CrjVf4yBmESU/edit?ts=5b5f17db#gid=227859291</td><td>PHESANT Transformation:20447_0| | CAT-SINGLE | | Inc(>=10): 1(48490) | | Inc(>=10): 0(17844) | | CAT-SINGLE-BINARY | | sample 17844/48490(66334) | | -Notes:Question asked: Did this worst period start within two months of the death of someone close to you or after a stressful or traumatic event in your life? Question was asked when ~F20446~ was Yes or ~F20441~ was Yes.-Variable type:binary</td></tr>
<tr><td>binary.20448.txt</td><td>1710174270056F5 forCTG.txt.gz</td><td>0.24</td><td>0.06599</td><td>3.637</td><td>0.000276</td><td>0.03983</td><td>0.009642</td><td>1.003</td><td>0.009932</td><td>0.003883</td><td>0.008052</td><td>Professional informed about depression</td><td>FALSE</td><td></td><td></td><td></td><td>66302</td><td>42728</td><td>23574</td><td>UK Biobank</td><td>https://docs.google.com/spreadsheets/d/1kvPoupSzsSFBNSztMzl04xMoSC3Kcx3CrjVf4yBmESU/edit?ts=5b5f17db#gid=227859291</td><td>PHESANT Transformation:20448_0| | CAT-SINGLE | | Inc(>=10): 1(42728) | | Inc(>=10): 0(23574) | | CAT-SINGLE-BINARY | | sample 23574/42728(66302) | | -Notes:Question asked: Did you ever tell a professional about these problems (medical doctor, psychologist, social worker, counsellor, nurse, clergy, or other helping professional)? Question was asked when ~F20446~ was Yes or ~F20441~ was Yes.-Variable type:binary</td></tr>
<tr><td>binary.20449.txt</td><td>1710174270056F5 forCTG.txt.gz</td><td>0.1543</td><td>0.09237</td><td>1.671</td><td>0.09475</td><td>0.03151</td><td>0.01063</td><td>1.001</td><td>0.01036</td><td>0.003928</td><td>0.008637</td><td>Feelings of tiredness during worst episode of depression</td><td>FALSE</td><td></td><td></td><td></td><td>59774</td><td>48952</td><td>10822</td><td>UK Biobank</td><td>https://docs.google.com/spreadsheets/d/1kvPoupSzsSFBNSztMzl04xMoSC3Kcx3CrjVf4yBmESU/edit?ts=5b5f17db#gid=227859291</td><td>PHESANT Transformation:20449_0| | CAT-SINGLE | | Inc(>=10): 1(48952) | | Inc(>=10): 0(10822) | | CAT-SINGLE-BINARY | | sample 10822/48952(59774) | | -Notes:Question asked: Did you feel more tired out or low on energy than is usual for you? Question was asked when ~F20446~ was Yes or ~F20441~ was Yes.-Variable type:binary</td></tr>
<tr><td>binary.20450.txt</td><td>1710174270056F5 forCTG.txt.gz</td><td>0.1262</td><td>0.06439</td><td>1.96</td><td>0.04997</td><td>0.04622</td><td>0.009261</td><td>1.005</td><td>0.009097</td><td>0.005192</td><td>0.007765</td><td>Feelings of worthlessness during worst period of depression</td><td>FALSE</td><td></td><td></td><td></td><td>62128</td><td>31593</td><td>30535</td><td>UK Biobank</td><td>https://docs.google.com/spreadsheets/d/1kvPoupSzsSFBNSztMzl04xMoSC3Kcx3CrjVf4yBmESU/edit?ts=5b5f17db#gid=227859291</td><td>PHESANT Transformation:20450_0| | CAT-SINGLE | | Inc(>=10): 0(30535) | | Inc(>=10): 1(31593) | | CAT-SINGLE-BINARY | | sample 30535/31593(62128) | | -Notes:Question asked: People sometimes feel down on themselves, no good, worthless. Did you feel this way? Question was asked when ~F20446~ was Yes or ~F20441~ was Yes.-Variable type:binary</td></tr>
</table>

<table>
<tr><td>binary.20463.txt</td><td>1710174270056F5forCTG.txt.gz</td><td>0.1833</td><td>0.1066</td><td>1.719</td><td>0.0856</td><td>0.01072</td><td>0.004767</td><td>0.992</td><td>0.009443</td><td>0.01028</td><td>0.007976</td><td>Ever heard an un-real voice</td><td>FALSE</td><td></td><td></td><td></td><td>117503</td><td>2009</td><td>115494</td><td>UK Biobank</td><td>https://docs.google.com/spreadsheets/d/1kvPoupSzsSFBNSztMzl04xMoSC3Kcx3CrjVf4yBmESU/edit?ts=5b5f17db#gid=227859291</td><td>PHESANT Transformation:20463_0|| CAT-SINGLE || Inc(>=10): 0(115494) || Inc(>=10): 1(2009) || CAT-SINGLE-BINARY || sample 115494/2009(117503) ||-Notes:Question asked: Did you ever hear things that other people said did not exist, like strange voices coming from inside your head talking to you or about you, or voices coming out of the air when there was no one around?-Variable type:binary</td></tr>
<tr><td>binary.20471.txt</td><td>1710174270056F5forCTG.txt.gz</td><td>0.2543</td><td>0.09768</td><td>2.603</td><td>0.009228</td><td>0.01439</td><td>0.004491</td><td>0.999</td><td>0.009031</td><td>0.01714</td><td>0.008366</td><td>Ever seen an un-real vision</td><td>FALSE</td><td></td><td></td><td></td><td>116787</td><td>3768</td><td>113019</td><td>UK Biobank</td><td>https://docs.google.com/spreadsheets/d/1kvPoupSzsSFBNSztMzl04xMoSC3Kcx3CrjVf4yBmESU/edit?ts=5b5f17db#gid=227859291</td><td>PHESANT Transformation:20471_0|| CAT-SINGLE || Inc(>=10): 0(113019) || Inc(>=10): 1(3768) || CAT-SINGLE-BINARY || sample 113019/3768(116787) ||-Notes:Question asked: Did you ever see something that wasn't really there that other people could not see?-Variable type:binary</td></tr>
<tr><td>binary.20480.txt</td><td>1710174270056F5forCTG.txt.gz</td><td>0.308</td><td>0.06792</td><td>4.535</td><td>5.77E-06</td><td>0.02297</td><td>0.005184</td><td>1.003</td><td>0.009781</td><td>0.01453</td><td>0.008175</td><td>Ever self-harmed</td><td>TRUE</td><td>Psychiatric</td><td></td><td>Ever self-harmed</td><td>117733</td><td>5099</td><td>112634</td><td>UK Biobank</td><td>https://docs.google.com/spreadsheets/d/1kvPoupSzsSFBNSztMzl04xMoSC3Kcx3CrjVf4yBmESU/edit?ts=5b5f17db#gid=227859291</td><td>PHESANT Transformation:20480_0|| CAT-SINGLE || Inc(>=10): 0(112634) || Inc(>=10): 1(5099) || CAT-SINGLE-BINARY || sample 112634/5099(117733) ||-Notes:Question asked: Have you deliberately harmed yourself, whether or not you meant to end your life?-Variable type:binary</td></tr>
<tr><td>binary.20493.txt</td><td>1710174270056F5forCTG.txt.gz</td><td>0.01312</td><td>0.07412</td><td>0.177</td><td>0.8595</td><td>0.07856</td><td>0.0202</td><td>0.984</td><td>0.009017</td><td>0.01168</td><td>0.006933</td><td>Severity of problems due to mania or irritability</td><td>FALSE</td><td></td><td></td><td></td><td>26655</td><td>7988</td><td>18667</td><td>UK Biobank</td><td>https://docs.google.com/spreadsheets/d/1kvPoupSzsSFBNSztMzl04xMoSC3Kcx3CrjVf4yBmESU/edit?ts=5b5f17db#gid=227859291</td><td>PHESANT Transformation:20493_0|| CAT-SINGLE || Inc(>=10): 0(18667) || Inc(>=10): 1(7988) || CAT-SINGLE-BINARY || sample 18667/7988(26655) ||-Notes:Question asked: How much of a problem have these high or irritable periods caused you? Question was asked when ~F20501~ was Yes or ~F20502~ was Yes.-Variable type:binary</td></tr>
<tr><td>binary.20499.txt</td><td>1710174270056F5forCTG.txt.gz</td><td>0.2027</td><td>0.03878</td><td>5.226</td><td>1.73E-07</td><td>0.0691</td><td>0.006597</td><td>1.001</td><td>0.01157</td><td>0.006885</td><td>0.007668</td><td>Ever sought or received professional help for mental distress</td><td>TRUE</td><td>Psychiatric</td><td></td><td>Ever sought or received professional help for mental distress</td><td>117677</td><td>46020</td><td>71657</td><td>UK Biobank</td><td>https://docs.google.com/spreadsheets/d/1kvPoupSzsSFBNSztMzl04xMoSC3Kcx3CrjVf4yBmESU/edit?ts=5b5f17db#gid=227859291</td><td>PHESANT Transformation:20499_0|| CAT-SINGLE || Inc(>=10): 0(71657) || Inc(>=10): 1(46020) || CAT-SINGLE-BINARY || sample 71657/46020(117677) ||-Notes:Question asked: In your life, did you seek or receive help from a professional (medical doctor, psychologist, social worker, counsellor, nurse, clergy, or other helping professional) for mental distress, psychological problems or unusual experiences?-Variable type:binary</td></tr>
<tr><td>binary.20500.txt</td><td>1710174270056F5forCTG.txt.gz</td><td>0.2951</td><td>0.0422</td><td>6.993</td><td>2.69E-12</td><td>0.06281</td><td>0.006014</td><td>1.008</td><td>0.01108</td><td>0.001765</td><td>0.00804</td><td>Ever suffered mental distress preventing usual activities</td><td>FALSE</td><td>Psychiatric</td><td></td><td></td><td>116527</td><td>38681</td><td>77846</td><td>UK Biobank</td><td>https://docs.google.com/spreadsheets/d/1kvPoupSzsSFBNSztMzl04xMoSC3Kcx3CrjVf4yBmESU/edit?ts=5b5f17db#gid=227859291</td><td>PHESANT Transformation:20500_0|| CAT-SINGLE || Inc(>=10): 0(77846) || Inc(>=10): 1(38681) || CAT-SINGLE-BINARY || sample 77846/38681(116527) ||-Notes:Question asked: In your life, have you suffered from a period of mental distress that prevented you from doing your usual activities?-Variable type:binary</td></tr>
<tr><td>binary.20501.txt</td><td>1710174270056F5forCTG.txt.gz</td><td>0.343</td><td>0.08554</td><td>4.01</td><td>6.07E-05</td><td>0.02091</td><td>0.005537</td><td>0.991</td><td>0.0103</td><td>0.008947</td><td>0.008279</td><td>Ever had period of mania / excitability</td><td>FALSE</td><td></td><td></td><td></td><td>115338</td><td>4816</td><td>110522</td><td>UK Biobank</td><td>https://docs.google.com/spreadsheets/d/1kvPoupSzsSFBNSztMzl04xMoSC3Kcx3CrjVf4yBmESU/edit?ts=5b5f17db#gid=227859291</td><td>PHESANT Transformation:20501_0|| CAT-SINGLE || Inc(>=10): 0(110522) || Inc(>=10): 1(4816) || CAT-SINGLE-BINARY || sample 110522/4816(115338) ||-Notes:Question asked: Have you ever had a period of time when you were feeling so good, high, excited, or hyper that other people thought you were not your normal self or you were so hyper that you got into trouble?-Variable type:binary</td></tr>
<tr><td>binary.20502.txt</td><td>1710174270056F5forCTG.txt.gz</td><td>0.3354</td><td>0.04846</td><td>6.922</td><td>4.45E-12</td><td>0.05818</td><td>0.006685</td><td>1</td><td>0.01186</td><td>0.006951</td><td>0.007937</td><td>Ever had period extreme irritability</td><td>FALSE</td><td>Psychiatric</td><td></td><td></td><td>114422</td><td>29747</td><td>84675</td><td>UK Biobank</td><td>https://docs.google.com/spreadsheets/d/1kvPoupSzsSFBNSztMzl04xMoSC3Kcx3CrjVf4yBmESU/edit?ts=5b5f17db#gid=227859291</td><td>PHESANT Transformation:20502_0|| CAT-SINGLE || Inc(>=10): 1(29747) || Inc(>=10): 0(84675) || CAT-SINGLE-BINARY || sample 84675/29747(114422) ||-Notes:Question asked: Have you ever had a period of time when you were so irritable that you found yourself shouting at people or starting fights or arguments?-Variable type:binary</td></tr>
<tr><td>binary.20526.txt</td><td>1710174270056F5forCTG.txt.gz</td><td>0.4353</td><td>0.09806</td><td>4.439</td><td>9.02E-06</td><td>0.01624</td><td>0.004863</td><td>1.01</td><td>0.00893</td><td>0.006206</td><td>0.007699</td><td>Been in serious accident believed to be life-threatening</td><td>FALSE</td><td>Wellbeing</td><td></td><td></td><td>117922</td><td>11325</td><td>106597</td><td>UK Biobank</td><td>https://docs.google.com/spreadsheets/d/1kvPoupSzsSFBNSztMzl04xMoSC3Kcx3CrjVf4yBmESU/edit?ts=5b5f17db#gid=227859291</td><td>PHESANT Transformation:20526_0|| CAT-SINGLE || reassignments: 2=1 || Inc(>=10): 0(106597) || Inc(>=10): 1(11325) || CAT-SINGLE-BINARY || sample 106597/11325(117922) ||-Notes:Question asked: In your life, have you...? Been in a serious accident that you believed to be life-threatening at the time-Variable type:binary</td></tr>
<tr><td>binary.20527.txt</td><td>1710174270056F5forCTG.txt.gz</td><td>0.6162</td><td>0.2282</td><td>2.701</td><td>0.006923</td><td>0.007055</td><td>0.004706</td><td>1.013</td><td>0.009677</td><td>0.01199</td><td>0.007814</td><td>Been involved in combat or exposed to war-zone</td><td>FALSE</td><td></td><td></td><td></td><td>117954</td><td>4010</td><td>113944</td><td>UK Biobank</td><td>https://docs.google.com/spreadsheets/d/1kvPoupSzsSFBNSztMzl04xMoSC3Kcx3CrjVf4yBmESU/edit?ts=5b5f17db#gid=227859291</td><td>PHESANT Transformation:20527_0|| CAT-SINGLE || reassignments: 2=1 || Inc(>=10): 0(113944) || Inc(>=10): 1(4010) || CAT-SINGLE-BINARY || sample 113944/4010(117954) ||-Notes:Question asked: In your life, have you...? Been involved in combat or exposed to a war-zone (either in the military or as a civilian)-Variable type:binary</td></tr>
<tr><td>binary.20528.txt</td><td>1710174270056F5forCTG.txt.gz</td><td>0.2961</td><td>0.1074</td><td>2.755</td><td>0.00586</td><td>0.01134</td><td>0.004902</td><td>1.011</td><td>0.008576</td><td>0.006926</td><td>0.007487</td><td>Diagnosed with life-threatening illness</td><td>FALSE</td><td></td><td></td><td></td><td>117617</td><td>19291</td><td>98326</td><td>UK Biobank</td><td>https://docs.google.com/spreadsheets/d/1kvPoupSzsSFBNSztMzl04xMoSC3Kcx3CrjVf4yBmESU/edit?ts=5b5f17db#gid=227859291</td><td>PHESANT Transformation:20528_0|| CAT-SINGLE || reassignments: 2=1 || Inc(>=10): 0(98326) || Inc(>=10): 1(19291) || CAT-SINGLE-BINARY || sample 98326/19291(117617) ||-Notes:Question asked: In your life, have you...? Been diagnosed with a life-threatening illness-Variable type:binary</td></tr>
<tr><td>binary.20529.txt</td><td>1710174270056F5forCTG.txt.gz</td><td>0.1268</td><td>0.06064</td><td>2.091</td><td>0.03654</td><td>0.02777</td><td>0.005357</td><td>0.998</td><td>0.01029</td><td>0.01812</td><td>0.008344</td><td>Victim of physically violent crime</td><td>FALSE</td><td></td><td></td><td></td><td>117846</td><td>21926</td><td>95920</td><td>UK Biobank</td><td>https://docs.google.com/spreadsheets/d/1kvPoupSzsSFBNSztMzl04xMoSC3Kcx3CrjVf4yBmESU/edit?ts=5b5f17db#gid=227859291</td><td>PHESANT Transformation:20529_0|| CAT-SINGLE || reassignments: 2=1 || Inc(>=10): 1(21926) || Inc(>=10): 0(95920) || CAT-SINGLE-BINARY || sample 95920/21926(117846) ||-Notes:Question asked: In your life, have you...? Been attacked, mugged, robbed, or been the victim of a physically violent crime-Variable type:binary</td></tr>
<tr><td>binary.20530.txt</td><td>1710174270056F5forCTG.txt.gz</td><td>0.3592</td><td>0.06956</td><td>5.164</td><td>2.41E-07</td><td>0.0283</td><td>0.005345</td><td>0.996</td><td>0.01033</td><td>0.00924</td><td>0.008715</td><td>Witnessed sudden violent death</td><td>FALSE</td><td>Wellbeing</td><td></td><td></td><td>117862</td><td>15959</td><td>101903</td><td>UK Biobank</td><td>https://docs.google.com/spreadsheets/d/1kvPoupSzsSFBNSztMzl04xMoSC3Kcx3CrjVf4yBmESU/edit?ts=5b5f17db#gid=227859291</td><td>PHESANT Transformation:20530_0|| CAT-SINGLE || reassignments: 2=1 || Inc(>=10): 0(101903) || Inc(>=10): 1(15959) || CAT-SINGLE-BINARY || sample 101903/15959(117862) ||-Notes:Question asked: In your life, have you...? Witnessed a sudden violent death (eg. murder, suicide, aftermath of an accident)-Variable type:binary</td></tr>
<tr><td>binary.20531.txt</td><td>1710174270056F5forCTG.txt.gz</td><td>0.3008</td><td>0.05746</td><td>5.234</td><td>1.66E-07</td><td>0.03624</td><td>0.00564</td><td>1.001</td><td>0.01017</td><td>0.0126</td><td>0.008532</td><td>Victim of sexual assault</td><td>FALSE</td><td>Wellbeing</td><td></td><td></td><td>116671</td><td>17230</td><td>99441</td><td>UK Biobank</td><td>https://docs.google.com/spreadsheets/d/1kvPoupSzsSFBNSztMzl04xMoSC3Kcx3CrjVf4yBmESU/edit?ts=5b5f17db#gid=227859291</td><td>PHESANT Transformation:20531_0|| CAT-SINGLE || reassignments: 2=1 || Inc(>=10): 1(17230) || Inc(>=10): 0(99441) || CAT-SINGLE-BINARY || sample 99441/17230(116671) ||-Notes:Question asked: In your life, have you...? Been a victim of a sexual assault, whether by a stranger or someone you knew-Variable type:binary</td></tr>
<tr><td>binary.20532.txt</td><td>1710174270056F5forCTG.txt.gz</td><td>0.5439</td><td>0.1726</td><td>3.15</td><td>0.00163</td><td>0.02041</td><td>0.0111</td><td>1.006</td><td>0.01001</td><td>-0.01365</td><td>0.008337</td><td>Did your sleep change?</td><td>FALSE</td><td></td><td></td><td></td><td>57086</td><td>45540</td><td>11546</td><td>UK Biobank</td><td>https://docs.google.com/spreadsheets/d/1kvPoupSzsSFBNSztMzl04xMoSC3Kcx3CrjVf4yBmESU/edit?ts=5b5f17db#gid=227859291</td><td>PHESANT Transformation:20532_0|| CAT-SINGLE || Inc(>=10): 1(45540) || Inc(>=10): 0(11546) || CAT-SINGLE-BINARY || sample 11546/45540(57086) ||-Notes:Question asked: Did your sleep change? Question was asked when ~F20446~ was Yes or ~F20441~ was Yes.-Variable type:binary</td></tr>
<tr><td>binary.20533.txt</td><td>1710174270056F5forCTG.txt.gz</td><td>0.1295</td><td>0.07669</td><td>1.689</td><td>0.09119</td><td>0.04436</td><td>0.01092</td><td>0.992</td><td>0.008814</td><td>0.005219</td><td>0.007792</td><td>Trouble falling asleep</td><td>FALSE</td><td></td><td></td><td></td><td>45540</td><td>34491</td><td>11049</td><td>UK Biobank</td><td>https://docs.google.com/spreadsheets/d/1kvPoupSzsSFBNSztMzl04xMoSC3Kcx3CrjVf4yBmESU/edit?ts=5b5f17db#gid=227859291</td><td>PHESANT Transformation:20533_0|| CAT-SINGLE || Inc(>=10): 1(34491) || Inc(>=10): 0(11049) || CAT-SINGLE-BINARY || sample 11049/34491(45540) ||-Notes:Question asked: Was that: [re sleep change] Trouble falling asleep Question was asked when ~F20532~ was Yes.-Variable type:binary</td></tr>
<tr><td>binary.20534.txt</td><td>1710174270056F5forCTG.txt.gz</td><td>0.2726</td><td>0.09765</td><td>2.792</td><td>0.005238</td><td>0.04122</td><td>0.01218</td><td>0.998</td><td>0.009318</td><td>0.004865</td><td>0.00838</td><td>Sleeping too much</td><td>FALSE</td><td></td><td></td><td></td><td>45540</td><td>9354</td><td>36186</td><td>UK Biobank</td><td>https://docs.google.com/spreadsheets/d/1kvPoupSzsSFBNSztMzl04xMoSC3Kcx3CrjVf4yBmESU/edit?ts=5b5f17db#gid=227859291</td><td>PHESANT Transformation:20534_0|| CAT-SINGLE || Inc(>=10): 0(36186) || Inc(>=10): 1(9354) || CAT-SINGLE-BINARY || sample 36186/9354(45540) ||-Notes:Question asked: Was that: [re sleep change] Sleeping too much Question was asked when ~F20532~ was Yes.-Variable type:binary</td></tr>
<tr><td>binary.20535.txt</td><td>1710174270056F5forCTG.txt.gz</td><td>0.04806</td><td>0.08837</td><td>0.5439</td><td>0.5865</td><td>0.03881</td><td>0.0127</td><td>1.008</td><td>0.009484</td><td>0.005869</td><td>0.007926</td><td>Waking too early</td><td>FALSE</td><td></td><td></td><td></td><td>45540</td><td>34528</td><td>11012</td><td>UK Biobank</td><td>https://docs.google.com/spreadsheets/d/1kvPoupSzsSFBNSztMzl04xMoSC3Kcx3CrjVf4yBmESU/edit?ts=5b5f17db#gid=227859291</td><td>PHESANT Transformation:20535_0|| CAT-SINGLE || Inc(>=10): 1(34528) || Inc(>=10): 0(11012) || CAT-SINGLE-BINARY || sample 11012/34528(45540) ||-Notes:Question asked: Was that: [re sleep change] Waking too early Question was asked when ~F20532~ was Yes.-Variable type:binary</td></tr>
<tr><td>binary.20536_0.txt</td><td>1710174270056F5forCTG.txt.gz</td><td>-0.529</td><td>0.06525</td><td>-8.108</td><td>5.17E-16</td><td>0.06386</td><td>0.01112</td><td>0.98</td><td>0.01022</td><td>0.01261</td><td>0.00737</td><td>Weight change during worst episode of depression: Stayed about the same or was on a diet</td><td>FALSE</td><td>Psychiatric</td><td></td><td></td><td>56571</td><td>22981</td><td>33590</td><td>UK Biobank</td><td>https://docs.google.com/spreadsheets/d/1kvPoupSzsSFBNSztMzl04xMoSC3Kcx3CrjVf4yBmESU/edit?ts=5b5f17db#gid=227859291</td><td>PHESANT Transformation:20536_0 || CAT-SINGLE || CAT-SINGLE-BINARY-VAR: 0 || Inc(>=10): 0(22981) ||-Notes:Question asked: Did you gain or lose weight without trying, or did you stay about the same weight? Question was asked when ~F20446~ was Yes or ~F20441~ was Yes.-Variable type:binary</td></tr>
<tr><td>binary.20536_1.txt</td><td>1710174270056F5forCTG.txt.gz</td><td>0.4462</td><td>0.06079</td><td>7.339</td><td>2.15E-13</td><td>0.05933</td><td>0.01034</td><td>0.992</td><td>0.009781</td><td>-0.0004</td><td>0.007416</td><td>Weight change during worst episode of depression: Gained weight</td><td>FALSE</td><td>Psychiatric</td><td></td><td></td><td>56571</td><td>9708</td><td>46863</td><td>UK Biobank</td><td>https://docs.google.com/spreadsheets/d/1kvPoupSzsSFBNSztMzl04xMoSC3Kcx3CrjVf4yBmESU/edit?ts=5b5f17db#gid=227859291</td><td>PHESANT Transformation:20536_0 || CAT-SINGLE || CAT-SINGLE-BINARY-VAR: 1 || Inc(>=10): 1(9708) ||-Notes:Question asked: Did you gain or lose weight without trying, or did you stay about the same weight? Question was asked when ~F20446~ was Yes or ~F20441~ was Yes.-Variable type:binary</td></tr>
<tr><td>binary.20536_2.txt</td><td>1710174270056F5forCTG.txt.gz</td><td>0.1412</td><td>0.08347</td><td>1.692</td><td>0.09074</td><td>0.03359</td><td>0.01068</td><td>1.001</td><td>0.009667</td><td>-0.009948</td><td>0.007669</td><td>Weight change during worst episode of depression: Lost weight</td><td>FALSE</td><td></td><td></td><td></td><td>56571</td><td>20313</td><td>36258</td><td>UK Biobank</td><td>https://docs.google.com/spreadsheets/d/1kvPoupSzsSFBNSztMzl04xMoSC3Kcx3CrjVf4yBmESU/edit?ts=5b5f17db#gid=227859291</td><td>PHESANT Transformation:20536_0 || CAT-SINGLE || CAT-SINGLE-BINARY-VAR: 2 || Inc(>=10): 2(20313) ||-Notes:Question asked: Did you gain or lose weight without trying, or did you stay about the same weight? Question was asked when ~F20446~ was Yes or ~F20441~ was Yes.-Variable type:binary</td></tr>
<tr><td>binary.20541.txt</td><td>1710174270056F5forCTG.txt.gz</td><td>-0.07836</td><td>0.08895</td><td>-0.8809</td><td>0.3784</td><td>0.03854</td><td>0.01528</td><td>0.991</td><td>0.009221</td><td>0.005485</td><td>0.007196</td><td>Difficulty stopping worrying during worst period of anxiety</td><td>FALSE</td><td></td><td></td><td></td><td>35523</td><td>33466</td><td>2057</td><td>UK Biobank</td><td>https://docs.google.com/spreadsheets/d/1kvPoupSzsSFBNSztMzl04xMoSC3Kcx3CrjVf4yBmESU/edit?ts=5b5f17db#gid=227859291</td><td>PHESANT Transformation:20541_0|| CAT-SINGLE || Inc(>=10): 1(33466) || Inc(>=10): 0(2057) || CAT-SINGLE-BINARY || sample 2057/33466(35523) ||-Notes:Question asked: Please think of the period in your life when you have felt worried, tense, anxious, or more worried than most people would in your situation. This could be in the past, or it could be continuing now. Did you find it difficult to stop worrying? Question was asked when ~F20425~ was Yes or ~F20420~ was at least 6 months.-Variable type:binary</td></tr>
<tr><td>binary.20543.txt</td><td>1710174270056F5forCTG.txt.gz</td><td>0.1281</td><td>0.08805</td><td>1.455</td><td>0.1457</td><td>0.04763</td><td>0.0145</td><td>0.976</td><td>0.008475</td><td>0.008819</td><td>0.007768</td><td>Number of things worried about during worst period of anxiety</td><td>FALSE</td><td></td><td></td><td></td><td>35558</td><td>15643</td><td>19915</td><td>UK Biobank</td><td>https://docs.google.com/spreadsheets/d/1kvPoupSzsSFBNSztMzl04xMoSC3Kcx3CrjVf4yBmESU/edit?ts=5b5f17db#gid=227859291</td><td>PHESANT Transformation:20543_0|| CAT-SINGLE || Inc(>=10): 1(15643) || Inc(>=10): 2(19915) || CAT-SINGLE-BINARY || sample 15643/19915(35558) ||-Notes:Question asked: Please think of the period in your life when you have felt worried, tense, anxious, or more worried than most people would in your situation. This could be in the past, or it could be continuing now. Did you usually worry about one particular thing, such as your job security or the failing health of a loved one, or more than one thing? Question was asked when ~F20425~ was Yes or ~F20420~ was at least 6 months.-Variable type:binary</td></tr>
</table>

<table>
<tr><td>binary.20544_1.txt</td><td>1710174270056F5 forCTG.txt.gz</td><td>0.1058</td><td>0.1096</td><td>0.9651</td><td>0.3345</td><td>0.009605</td><td>0.004414</td><td>1.003</td><td>0.009617</td><td>0.007993</td><td>0.008119</td><td>Mental health problems ever diagnosed by a professional: Social anxiety or social phobia</td><td>FALSE</td><td></td><td></td><td></td><td>117716</td><td>1474</td><td>116242</td><td>UK Biobank</td><td>https://docs.google.com/spreadsheets/d/1kvPoupSzsSFBNSztMzl04xMoSC3Kcx3CrjVf4yBmESU/edit?ts=5b5f17db#gid=227859291</td><td>PHESANT Transformation:20544_0 | | CAT-MUL-BINARY-VAR 1 | | Indicator name x20400_0_0 | | Remove indicator var NAs: 243105 | | Removed 373 examples != 1 but with missing value (<0) | | sample 116242/1474(117716) | |-Notes:Question asked: Have you been diagnosed with one or more of the following mental health problems by a professional, even if you don't have it currently? (tick all that apply): Participant was offered a set of options which have been combined here into a single answer-Variable type:binary</td></tr>
<tr><td>binary.20544_11.txt</td><td>1710174270056F5 forCTG.txt.gz</td><td>0.3262</td><td>0.04554</td><td>7.163</td><td>7.87E-13</td><td>0.05169</td><td>0.005967</td><td>0.996</td><td>0.01072</td><td>0.004933</td><td>0.00761</td><td>Mental health problems ever diagnosed by a professional: Depression</td><td>FALSE</td><td>Psychiatric</td><td></td><td></td><td>117782</td><td>25087</td><td>92695</td><td>UK Biobank</td><td>https://docs.google.com/spreadsheets/d/1kvPoupSzsSFBNSztMzl04xMoSC3Kcx3CrjVf4yBmESU/edit?ts=5b5f17db#gid=227859291</td><td>PHESANT Transformation:20544_0 | | CAT-MUL-BINARY-VAR 11 | | Indicator name x20400_0_0 | | Remove indicator var NAs: 243105 | | Removed 307 examples != 11 but with missing value (<0) | | sample 92695/25087(117782) | |-Notes:Question asked: Have you been diagnosed with one or more of the following mental health problems by a professional, even if you don't have it currently? (tick all that apply): Participant was offered a set of options which have been combined here into a single answer-Variable type:binary</td></tr>
<tr><td>binary.20544_15.txt</td><td>1710174270056F5 forCTG.txt.gz</td><td>0.1143</td><td>0.05797</td><td>1.972</td><td>0.04866</td><td>0.04066</td><td>0.005236</td><td>0.985</td><td>0.009946</td><td>-0.001542</td><td>0.008426</td><td>Mental health problems ever diagnosed by a professional: Anxiety, nerves or generalized anxiety disorder</td><td>FALSE</td><td></td><td></td><td></td><td>117751</td><td>16730</td><td>101021</td><td>UK Biobank</td><td>https://docs.google.com/spreadsheets/d/1kvPoupSzsSFBNSztMzl04xMoSC3Kcx3CrjVf4yBmESU/edit?ts=5b5f17db#gid=227859291</td><td>PHESANT Transformation:20544_0 | | CAT-MUL-BINARY-VAR 15 | | Indicator name x20400_0_0 | | Remove indicator var NAs: 243105 | | Removed 338 examples != 15 but with missing value (<0) | | sample 101021/16730(117751) | |-Notes:Question asked: Have you been diagnosed with one or more of the following mental health problems by a professional, even if you don't have it currently? (tick all that apply): Participant was offered a set of options which have been combined here into a single answer-Variable type:binary</td></tr>
<tr><td>binary.20544_6.txt</td><td>1710174270056F5 forCTG.txt.gz</td><td>0.3213</td><td>0.07607</td><td>4.224</td><td>2.40E-05</td><td>0.02212</td><td>0.00487</td><td>1.003</td><td>0.01</td><td>0.002994</td><td>0.008252</td><td>Mental health problems ever diagnosed by a professional: Panic attacks</td><td>FALSE</td><td>Psychiatric</td><td></td><td></td><td>117722</td><td>6518</td><td>111204</td><td>UK Biobank</td><td>https://docs.google.com/spreadsheets/d/1kvPoupSzsSFBNSztMzl04xMoSC3Kcx3CrjVf4yBmESU/edit?ts=5b5f17db#gid=227859291</td><td>PHESANT Transformation:20544_0 | | CAT-MUL-BINARY-VAR 6 | | Indicator name x20400_0_0 | | Remove indicator var NAs: 243105 | | Removed 367 examples != 6 but with missing value (<0) | | sample 111204/6518(117722) | | SKIP_val:-818 < 0 | |-Notes:Question asked: Have you been diagnosed with one or more of the following mental health problems by a professional, even if you don't have it currently? (tick all that apply): Participant was offered a set of options which have been combined here into a single answer-Variable type:binary</td></tr>
<tr><td>binary.20544_7.txt</td><td>1710174270056F5 forCTG.txt.gz</td><td>0.08028</td><td>0.133</td><td>0.6035</td><td>0.5462</td><td>0.006332</td><td>0.004289</td><td>1.007</td><td>0.00903</td><td>0.001095</td><td>0.007519</td><td>Mental health problems ever diagnosed by a professional: Obsessive compulsive disorder (OCD)</td><td>FALSE</td><td></td><td></td><td></td><td>117711</td><td>733</td><td>116978</td><td>UK Biobank</td><td>https://docs.google.com/spreadsheets/d/1kvPoupSzsSFBNSztMzl04xMoSC3Kcx3CrjVf4yBmESU/edit?ts=5b5f17db#gid=227859291</td><td>PHESANT Transformation:20544_0 | | CAT-MUL-BINARY-VAR 7 | | Indicator name x20400_0_0 | | Remove indicator var NAs: 243105 | | Removed 378 examples != 7 but with missing value (<0) | | sample 116978/733(117711) | |-Notes:Question asked: Have you been diagnosed with one or more of the following mental health problems by a professional, even if you don't have it currently? (tick all that apply): Participant was offered a set of options which have been combined here into a single answer-Variable type:binary</td></tr>
<tr><td>binary.20546_1.txt</td><td>1710174270056F5 forCTG.txt.gz</td><td>0.08792</td><td>0.1167</td><td>0.7533</td><td>0.4512</td><td>0.00794</td><td>0.004449</td><td>0.996</td><td>0.009689</td><td>0.01276</td><td>0.007727</td><td>Substances taken for depression: Unprescribed medication (more than once)</td><td>FALSE</td><td></td><td></td><td></td><td>117763</td><td>4111</td><td>113652</td><td>UK Biobank</td><td>https://docs.google.com/spreadsheets/d/1kvPoupSzsSFBNSztMzl04xMoSC3Kcx3CrjVf4yBmESU/edit?ts=5b5f17db#gid=227859291</td><td>PHESANT Transformation:20546_0 | | CAT-MUL-BINARY-VAR 1 | | Indicator name x20446_0_0 | | Remove indicator var NAs: 243128 | | Remove indicator var <0: 303 | | Removed 0 examples != 1 but with missing value (<0) | | sample 113652/4111(117763) | |-Notes:Question asked: Did you ever try the following for these problems? (tick all that apply) Participant was offered a set of options which have been combined here into a single answer Question was asked when ~F20446~ was Yes or ~F20441~ was Yes.-Variable type:binary</td></tr>
<tr><td>binary.20546_3.txt</td><td>1710174270056F5 forCTG.txt.gz</td><td>0.2787</td><td>0.04908</td><td>5.679</td><td>1.35E-08</td><td>0.05247</td><td>0.005971</td><td>1.001</td><td>0.01043</td><td>0.01162</td><td>0.008393</td><td>Substances taken for depression: Medication prescribed to you (for at least two weeks)</td><td>FALSE</td><td>Psychiatric</td><td></td><td></td><td>117763</td><td>28351</td><td>89412</td><td>UK Biobank</td><td>https://docs.google.com/spreadsheets/d/1kvPoupSzsSFBNSztMzl04xMoSC3Kcx3CrjVf4yBmESU/edit?ts=5b5f17db#gid=227859291</td><td>PHESANT Transformation:20546_0 | | CAT-MUL-BINARY-VAR 3 | | Indicator name x20446_0_0 | | Remove indicator var NAs: 243128 | | Remove indicator var <0: 303 | | Removed 0 examples != 3 but with missing value (<0) | | sample 89412/28351(117763) | |-Notes:Question asked: Did you ever try the following for these problems? (tick all that apply) Participant was offered a set of options which have been combined here into a single answer Question was asked when ~F20446~ was Yes or ~F20441~ was Yes.-Variable type:binary</td></tr>
<tr><td>binary.20546_4.txt</td><td>1710174270056F5 forCTG.txt.gz</td><td>0.1888</td><td>0.0683</td><td>2.764</td><td>0.005706</td><td>0.02511</td><td>0.005385</td><td>1.001</td><td>0.009855</td><td>0.009898</td><td>0.007781</td><td>Substances taken for depression: Drugs or alcohol (more than once)</td><td>FALSE</td><td></td><td></td><td></td><td>117763</td><td>8627</td><td>109136</td><td>UK Biobank</td><td>https://docs.google.com/spreadsheets/d/1kvPoupSzsSFBNSztMzl04xMoSC3Kcx3CrjVf4yBmESU/edit?ts=5b5f17db#gid=227859291</td><td>PHESANT Transformation:20546_0 | | CAT-MUL-BINARY-VAR 4 | | Indicator name x20446_0_0 | | Remove indicator var NAs: 243128 | | Remove indicator var <0: 303 | | Removed 0 examples != 4 but with missing value (<0) | | sample 109136/8627(117763) | |-Notes:Question asked: Did you ever try the following for these problems? (tick all that apply) Participant was offered a set of options which have been combined here into a single answer Question was asked when ~F20446~ was Yes or ~F20441~ was Yes.-Variable type:binary</td></tr>
<tr><td>binary.20547_1.txt</td><td>1710174270056F5 forCTG.txt.gz</td><td>0.2563</td><td>0.05001</td><td>5.125</td><td>2.98E-07</td><td>0.05057</td><td>0.005894</td><td>1.001</td><td>0.00982</td><td>-0.000714</td><td>0.007981</td><td>Activities undertaken to treat depression: Talking therapies, such as psychotherapy, counselling, group therapy or CBT</td><td>FALSE</td><td>Psychiatric</td><td></td><td></td><td>117763</td><td>25960</td><td>91803</td><td>UK Biobank</td><td>https://docs.google.com/spreadsheets/d/1kvPoupSzsSFBNSztMzl04xMoSC3Kcx3CrjVf4yBmESU/edit?ts=5b5f17db#gid=227859291</td><td>PHESANT Transformation:20547_0 | | CAT-MUL-BINARY-VAR 1 | | Indicator name x20446_0_0 | | Remove indicator var NAs: 243128 | | Remove indicator var <0: 303 | | Removed 0 examples != 1 but with missing value (<0) | | sample 91803/25960(117763) | |-Notes:Question asked: Did you ever try talking therapies for these problems, or other structured activities you regard as therapeutic? Include only those you attended more than once. Participant was offered a set of options which have been combined here into a single answer Question was asked when ~F20446~ was Yes or ~F20441~ was Yes.-Variable type:binary</td></tr>
<tr><td>binary.20547_3.txt</td><td>1710174270056F5 forCTG.txt.gz</td><td>0.2036</td><td>0.05996</td><td>3.395</td><td>0.000686</td><td>0.02896</td><td>0.005593</td><td>0.993</td><td>0.01036</td><td>0.008028</td><td>0.007783</td><td>Activities undertaken to treat depression: Other therapeutic activities such as mindfulness, yoga or art classes</td><td>FALSE</td><td></td><td></td><td></td><td>117763</td><td>9199</td><td>108564</td><td>UK Biobank</td><td>https://docs.google.com/spreadsheets/d/1kvPoupSzsSFBNSztMzl04xMoSC3Kcx3CrjVf4yBmESU/edit?ts=5b5f17db#gid=227859291</td><td>PHESANT Transformation:20547_0 | | CAT-MUL-BINARY-VAR 3 | | Indicator name x20446_0_0 | | Remove indicator var NAs: 243128 | | Remove indicator var <0: 303 | | Removed 0 examples != 3 but with missing value (<0) | | sample 108564/9199(117763) | |-Notes:Question asked: Did you ever try talking therapies for these problems, or other structured activities you regard as therapeutic? Include only those you attended more than once. Participant was offered a set of options which have been combined here into a single answer Question was asked when ~F20446~ was Yes or ~F20441~ was Yes.-Variable type:binary</td></tr>
<tr><td>binary.20548_1.txt</td><td>1710174270056F5 forCTG.txt.gz</td><td>0.331</td><td>0.0717</td><td>4.616</td><td>3.90E-06</td><td>0.02459</td><td>0.004932</td><td>1.001</td><td>0.00862</td><td>0.00393</td><td>0.007971</td><td>Manifestations of mania or irritability: I was more talkative than usual</td><td>FALSE</td><td>Psychiatric</td><td></td><td></td><td>114422</td><td>5465</td><td>108957</td><td>UK Biobank</td><td>https://docs.google.com/spreadsheets/d/1kvPoupSzsSFBNSztMzl04xMoSC3Kcx3CrjVf4yBmESU/edit?ts=5b5f17db#gid=227859291</td><td>PHESANT Transformation:20548_0 | | CAT-MUL-BINARY-VAR 1 | | Indicator name x20502_0_0 | | Remove indicator var NAs: 243128 | | Remove indicator var <0: 3644 | | Removed 0 examples != 1 but with missing value (<0) | | sample 108957/5465(114422) | |-Notes:Question asked: Please try to remember a period when you were in a high or irritable state and select all of the following that apply: Participant was offered a set of options which have been combined here into a single answer Question was asked when ~F20501~ was Yes or ~F20502~ was Yes.-Variable type:binary</td></tr>
<tr><td>binary.20548_2.txt</td><td>1710174270056F5 forCTG.txt.gz</td><td>0.3736</td><td>0.05985</td><td>6.241</td><td>4.35E-10</td><td>0.03316</td><td>0.005664</td><td>0.996</td><td>0.009933</td><td>-0.004434</td><td>0.007885</td><td>Manifestations of mania or irritability: I was more restless than usual</td><td>FALSE</td><td>Psychiatric</td><td></td><td></td><td>114422</td><td>12876</td><td>101546</td><td>UK Biobank</td><td>https://docs.google.com/spreadsheets/d/1kvPoupSzsSFBNSztMzl04xMoSC3Kcx3CrjVf4yBmESU/edit?ts=5b5f17db#gid=227859291</td><td>PHESANT Transformation:20548_0 | | CAT-MUL-BINARY-VAR 2 | | Indicator name x20502_0_0 | | Remove indicator var NAs: 243128 | | Remove indicator var <0: 3644 | | Removed 0 examples != 2 but with missing value (<0) | | sample 101546/12876(114422) | |-Notes:Question asked: Please try to remember a period when you were in a high or irritable state and select all of the following that apply: Participant was offered a set of options which have been combined here into a single answer Question was asked when ~F20501~ was Yes or ~F20502~ was Yes.-Variable type:binary</td></tr>
<tr><td>binary.20548_3.txt</td><td>1710174270056F5 forCTG.txt.gz</td><td>0.3172</td><td>0.07377</td><td>4.3</td><td>1.71E-05</td><td>0.0292</td><td>0.00592</td><td>1.003</td><td>0.01195</td><td>0.003756</td><td>0.009005</td><td>Manifestations of mania or irritability: My thoughts were racing</td><td>TRUE</td><td>Psychiatric</td><td></td><td>Racing thoughts</td><td>114422</td><td>10580</td><td>103842</td><td>UK Biobank</td><td>https://docs.google.com/spreadsheets/d/1kvPoupSzsSFBNSztMzl04xMoSC3Kcx3CrjVf4yBmESU/edit?ts=5b5f17db#gid=227859291</td><td>PHESANT Transformation:20548_0 | | CAT-MUL-BINARY-VAR 3 | | Indicator name x20502_0_0 | | Remove indicator var NAs: 243128 | | Remove indicator var <0: 3644 | | Removed 0 examples != 3 but with missing value (<0) | | sample 103842/10580(114422) | |-Notes:Question asked: Please try to remember a period when you were in a high or irritable state and select all of the following that apply: Participant was offered a set of options which have been combined here into a single answer Question was asked when ~F20501~ was Yes or ~F20502~ was Yes.-Variable type:binary</td></tr>
<tr><td>binary.20548_5.txt</td><td>1710174270056F5 forCTG.txt.gz</td><td>0.5062</td><td>0.1028</td><td>4.923</td><td>8.54E-07</td><td>0.01891</td><td>0.005038</td><td>0.993</td><td>0.01006</td><td>-0.00841</td><td>0.008144</td><td>Manifestations of mania or irritability: I needed less sleep than usual</td><td>FALSE</td><td>Psychiatric</td><td></td><td></td><td>114422</td><td>3777</td><td>110645</td><td>UK Biobank</td><td>https://docs.google.com/spreadsheets/d/1kvPoupSzsSFBNSztMzl04xMoSC3Kcx3CrjVf4yBmESU/edit?ts=5b5f17db#gid=227859291</td><td>PHESANT Transformation:20548_0 | | CAT-MUL-BINARY-VAR 5 | | Indicator name x20502_0_0 | | Remove indicator var NAs: 243128 | | Remove indicator var <0: 3644 | | Removed 0 examples != 5 but with missing value (<0) | | sample 110645/3777(114422) | |-Notes:Question asked: Please try to remember a period when you were in a high or irritable state and select all of the following that apply: Participant was offered a set of options which have been combined here into a single answer Question was asked when ~F20501~ was Yes or ~F20502~ was Yes.-Variable type:binary</td></tr>
<tr><td>binary.20548_6.txt</td><td>1710174270056F5 forCTG.txt.gz</td><td>0.5658</td><td>0.1504</td><td>3.762</td><td>0.000168</td><td>0.01117</td><td>0.005195</td><td>1.015</td><td>0.01029</td><td>-0.005072</td><td>0.008166</td><td>Manifestations of mania or irritability: I was more creative or had more ideas than usual</td><td>FALSE</td><td></td><td></td><td></td><td>114422</td><td>3090</td><td>111332</td><td>UK Biobank</td><td>https://docs.google.com/spreadsheets/d/1kvPoupSzsSFBNSztMzl04xMoSC3Kcx3CrjVf4yBmESU/edit?ts=5b5f17db#gid=227859291</td><td>PHESANT Transformation:20548_0 | | CAT-MUL-BINARY-VAR 6 | | Indicator name x20502_0_0 | | Remove indicator var NAs: 243128 | | Remove indicator var <0: 3644 | | Removed 0 examples != 6 but with missing value (<0) | | sample 111332/3090(114422) | |-Notes:Question asked: Please try to remember a period when you were in a high or irritable state and select all of the following that apply: Participant was offered a set of options which have been combined here into a single answer Question was asked when ~F20501~ was Yes or ~F20502~ was Yes.-Variable type:binary</td></tr>
</table>

binary.20548_7.txt	1710174270056F5 forCTG.txt.gz	0.4737	0.08914	5.314	1.07E-07	0.02122	0.005162	0.995	0.009312	-0.004852	0.00795	Manifestations of mania or irritability: I was easily distracted	TRUE	Psychiatric		Easily distracted	114422	9162	105260	UK Biobank	https://docs.google.com/spreadsheets/d/1kvPoupSzsSFBNSztMzl04xMoSC3Kcx3CrjVf4yBmESU/edit?ts=5b5f17db#gid=227859291	PHESANT Transformation:20548_0 | | CAT-MUL-BINARY-VAR 7 | | Indicator name x20502_0_0 | | Remove indicator var NAs: 243128 | | Remove indicator var <0: 3644 | | Removed 0 examples != 7 but with missing value (<0) | | sample 105260/9162(114422) | |-Notes:Question asked: Please try to remember a period when you were in a high or irritable state and select all of the following that apply: Participant was offered a set of options which have been combined here into a single answer Question was asked when ~F20501~ was Yes or ~F20502~ was Yes.-Variable type:binary
binary.20548_9.txt	1710174270056F5 forCTG.txt.gz	0.3785	0.09317	4.063	4.85E-05	0.0197	0.004957	0.998	0.00895	0.007146	0.008518	Manifestations of mania or irritability: I was more active than usual	FALSE				114422	5688	108734	UK Biobank	https://docs.google.com/spreadsheets/d/1kvPoupSzsSFBNSztMzl04xMoSC3Kcx3CrjVf4yBmESU/edit?ts=5b5f17db#gid=227859291	PHESANT Transformation:20548_0 | | CAT-MUL-BINARY-VAR 9 | | Indicator name x20502_0_0 | | Remove indicator var NAs: 243128 | | Remove indicator var <0: 3644 | | Removed 0 examples != 9 but with missing value (<0) | | sample 108734/5688(114422) | |-Notes:Question asked: Please try to remember a period when you were in a high or irritable state and select all of the following that apply: Participant was offered a set of options which have been combined here into a single answer Question was asked when ~F20501~ was Yes or ~F20502~ was Yes.-Variable type:binary
binary.20549_3.txt	1710174270056F5 forCTG.txt.gz	0.1714	0.05595	3.062	0.002195	0.04987	0.006062	0.99	0.009284	0.01096	0.008465	Substances taken for anxiety: Medication prescribed to you (for at least two weeks)	FALSE				98990	12942	86048	UK Biobank	https://docs.google.com/spreadsheets/d/1kvPoupSzsSFBNSztMzl04xMoSC3Kcx3CrjVf4yBmESU/edit?ts=5b5f17db#gid=227859291	PHESANT Transformation:20549_0 | | CAT-MUL-BINARY-VAR 3 | | Indicator name x20425_0_0 | | Remove indicator var NAs: 243128 | | Remove indicator var <0: 19076 | | Removed 0 examples != 3 but with missing value (<0) | | sample 86048/12942(98990) | |-Notes:Question asked: Did you ever use the following for the worry or the problems it caused? (tick all that apply): Participant was offered a set of options which have been combined here into a single answer Question was asked when ~F20425~ was Yes or ~F20420~ was at least 6 months.-Variable type:binary
binary.20549_4.txt	1710174270056F5 forCTG.txt.gz	0.197	0.08331	2.364	0.01806	0.01973	0.005232	1.007	0.009433	0.006503	0.008305	Substances taken for anxiety: Drugs or alcohol (more than once)	FALSE				98990	5402	93588	UK Biobank	https://docs.google.com/spreadsheets/d/1kvPoupSzsSFBNSztMzl04xMoSC3Kcx3CrjVf4yBmESU/edit?ts=5b5f17db#gid=227859291	PHESANT Transformation:20549_0 | | CAT-MUL-BINARY-VAR 4 | | Indicator name x20425_0_0 | | Remove indicator var NAs: 243128 | | Remove indicator var <0: 19076 | | Removed 0 examples != 4 but with missing value (<0) | | sample 93588/5402(98990) | |-Notes:Question asked: Did you ever use the following for the worry or the problems it caused? (tick all that apply): Participant was offered a set of options which have been combined here into a single answer Question was asked when ~F20425~ was Yes or ~F20420~ was at least 6 months.-Variable type:binary
binary.20550_1.txt	1710174270056F5 forCTG.txt.gz	0.139	0.05731	2.425	0.01533	0.04452	0.005985	0.982	0.009773	0.007123	0.007673	Activities undertaken to treat anxiety: Talking therapies, such as psychotherapy, counselling, group therapy or CBT	FALSE				98990	12858	86132	UK Biobank	https://docs.google.com/spreadsheets/d/1kvPoupSzsSFBNSztMzl04xMoSC3Kcx3CrjVf4yBmESU/edit?ts=5b5f17db#gid=227859291	PHESANT Transformation:20550_0 | | CAT-MUL-BINARY-VAR 1 | | Indicator name x20425_0_0 | | Remove indicator var NAs: 243128 | | Remove indicator var <0: 19076 | | Removed 0 examples != 1 but with missing value (<0) | | sample 86132/12858(98990) | |-Notes:Question asked: Did you ever try talking therapies for these problems, or other structured activities you regard as therapeutic? Include only those you attended more than once. Participant was offered a set of options which have been combined here into a single answer Question was asked when ~F20425~ was Yes or ~F20420~ was at least 6 months.-Variable type:binary
binary.20550_3.txt	1710174270056F5 forCTG.txt.gz	0.1465	0.07531	1.946	0.05165	0.02727	0.005696	0.996	0.008855	0.006348	0.007856	Activities undertaken to treat anxiety: Other therapeutic activities such as mindfulness, yoga or art classes	FALSE				98990	5735	93255	UK Biobank	https://docs.google.com/spreadsheets/d/1kvPoupSzsSFBNSztMzl04xMoSC3Kcx3CrjVf4yBmESU/edit?ts=5b5f17db#gid=227859291	PHESANT Transformation:20550_0 | | CAT-MUL-BINARY-VAR 3 | | Indicator name x20425_0_0 | | Remove indicator var NAs: 243128 | | Remove indicator var <0: 19076 | | Removed 0 examples != 3 but with missing value (<0) | | sample 93255/5735(98990) | |-Notes:Question asked: Did you ever try talking therapies for these problems, or other structured activities you regard as therapeutic? Include only those you attended more than once. Participant was offered a set of options which have been combined here into a single answer Question was asked when ~F20425~ was Yes or ~F20420~ was at least 6 months.-Variable type:binary
binary.20553_4.txt	1710174270056F5 forCTG.txt.gz	0.3437	0.1047	3.283	0.001028	0.01163	0.004315	1.02	0.009881	0.01365	0.008003	Methods of self-harm used: Ingesting a medication in excess of the normal dose	FALSE				117733	2937	114796	UK Biobank	https://docs.google.com/spreadsheets/d/1kvPoupSzsSFBNSztMzl04xMoSC3Kcx3CrjVf4yBmESU/edit?ts=5b5f17db#gid=227859291	PHESANT Transformation:20553_0 | | CAT-MUL-BINARY-VAR 4 | | Indicator name x20480_0_0 | | Remove indicator var NAs: 243128 | | Remove indicator var <0: 333 | | Removed 0 examples != 4 but with missing value (<0) | | sample 114796/2937(117733) | |-Notes:Question asked: Have you done any of the following to harm or endanger yourself? (tick all that apply): Participant was offered a set of options which have been combined here into a single answer Question was asked when ~F20480~ was Yes.-Variable type:binary
binary.20554_1.txt	1710174270056F5 forCTG.txt.gz	0.2183	0.09423	2.316	0.02054	0.0119	0.004661	1.01	0.009329	0.01276	0.008224	Actions taken following self-harm: See anyone from psychiatric or mental health services, including liaison services	FALSE				117733	1693	116040	UK Biobank	https://docs.google.com/spreadsheets/d/1kvPoupSzsSFBNSztMzl04xMoSC3Kcx3CrjVf4yBmESU/edit?ts=5b5f17db#gid=227859291	PHESANT Transformation:20554_0 | | CAT-MUL-BINARY-VAR 1 | | Indicator name x20480_0_0 | | Remove indicator var NAs: 243128 | | Remove indicator var <0: 333 | | Removed 0 examples != 1 but with missing value (<0) | | sample 116040/1693(117733) | |-Notes:Question asked: Following any time when you took an overdose or deliberately tried to harm yourself did you (tick all that apply): Participant was offered a set of options which have been combined here into a single answer Question was asked when ~F20480~ was Yes.-Variable type:binary
binary.2090.txt	1710174270056F5 forCTG.txt.gz	0.2526	0.02934	8.611	7.22E-18	0.06041	0.003381	1.015	0.0144	0.002021	0.009417	Seen doctor (GP) for nerves, anxiety, tension or depression	FALSE	Psychiatric			358693	123528	235165	UK Biobank	https://docs.google.com/spreadsheets/d/1kvPoupSzsSFBNSztMzl04xMoSC3Kcx3CrjVf4yBmESU/edit?ts=5b5f17db#gid=227859291	PHESANT Transformation:2090_0| | CAT-SINGLE | | Inc(>=10): 0(235165) | | Inc(>=10): 1(123528) | | CAT-SINGLE-BINARY | | sample 235165/123528(358693) | |-Notes:ACE touchscreen question Have you ever seen a general practitioner (GP) for nerves, anxiety, tension or depression?-Variable type:binary
binary.2100.txt	1710174270056F5 forCTG.txt.gz	0.2972	0.03665	8.11	5.07E-16	0.03092	0.002486	1.002	0.01253	0.00773	0.00907	Seen a psychiatrist for nerves, anxiety, tension or depression	FALSE	Psychiatric			359535	41233	318302	UK Biobank	https://docs.google.com/spreadsheets/d/1kvPoupSzsSFBNSztMzl04xMoSC3Kcx3CrjVf4yBmESU/edit?ts=5b5f17db#gid=227859291	PHESANT Transformation:2100_0| | CAT-SINGLE | | Inc(>=10): 0(318302) | | Inc(>=10): 1(41233) | | CAT-SINGLE-BINARY | | sample 318302/41233(359535) | |-Notes:ACE touchscreen question Have you ever seen a psychiatrist for nerves, anxiety, tension or depression?-Variable type:binary
binary.2129.txt	1710174270056F5 forCTG.txt.gz	0.04566	0.04281	1.066	0.2862	0.0233	0.002296	1.011	0.01197	0.01019	0.007989	Answered sexual history questions	FALSE				361015	331404	29611	UK Biobank	https://docs.google.com/spreadsheets/d/1kvPoupSzsSFBNSztMzl04xMoSC3Kcx3CrjVf4yBmESU/edit?ts=5b5f17db#gid=227859291	PHESANT Transformation:2129_0| | CAT-SINGLE | | Inc(>=10): 1(331404) | | Inc(>=10): 2(29611) | | CAT-SINGLE-BINARY | | sample 331404/29611(361015) | |-Notes:ACE touchscreen question The next section contains questions about your sexual history. If you feel that a question is too sensitive, you can skip the question or skip the entire section if you prefer.-Variable type:binary
binary.2159.txt	1710174270056F5 forCTG.txt.gz	0.129	0.058	2.224	0.02618	0.01124	0.002005	1.01	0.0102	-0.000422	0.007834	Ever had same-sex intercourse	FALSE				326849	11109	315740	UK Biobank	https://docs.google.com/spreadsheets/d/1kvPoupSzsSFBNSztMzl04xMoSC3Kcx3CrjVf4yBmESU/edit?ts=5b5f17db#gid=227859291	PHESANT Transformation:2159_0| | CAT-SINGLE | | Inc(>=10): 0(315740) | | Inc(>=10): 1(11109) | | CAT-SINGLE-BINARY | | sample 315740/11109(326849) | |-Notes:ACE touchscreen question Have you ever had sexual intercourse with someone of the same sex? If the participant activated the Help button they were shown the message: Sexual intercourse includes vaginal, oral or anal intercourse. ~F2159~ was collected from all participants except those who indicated they never had had sexual intercourse, as defined by their answers to ~F2139~-Variable type:binary
binary.2188.txt	1710174270056F5 forCTG.txt.gz	0.4082	0.0341	11.97	5.06E-33	0.0507	0.0028	1.026	0.01275	0.02166	0.008986	Long-standing illness, disability or infirmity	TRUE	Wellbeing		Long-standing illness, disability or infirmity	352798	114798	238000	UK Biobank	https://docs.google.com/spreadsheets/d/1kvPoupSzsSFBNSztMzl04xMoSC3Kcx3CrjVf4yBmESU/edit?ts=5b5f17db#gid=227859291	PHESANT Transformation:2188_0| | CAT-SINGLE | | Inc(>=10): 0(238000) | | Inc(>=10): 1(114798) | | CAT-SINGLE-BINARY | | sample 238000/114798(352798) | |-Notes:ACE touchscreen question Do you have any long-standing illness, disability or infirmity?-Variable type:binary
binary.2207.txt	1710174270056F5 forCTG.txt.gz	0.02158	0.04514	0.478	0.6326	0.0188	0.001806	0.987	0.01013	-0.000694	0.007629	Wears glasses or contact lenses	FALSE				360677	321768	38909	UK Biobank	https://docs.google.com/spreadsheets/d/1kvPoupSzsSFBNSztMzl04xMoSC3Kcx3CrjVf4yBmESU/edit?ts=5b5f17db#gid=227859291	PHESANT Transformation:2207_0| | CAT-SINGLE | | Inc(>=10): 1(321768) | | Inc(>=10): 0(38909) | | CAT-SINGLE-BINARY | | sample 38909/321768(360677) | |-Notes:ACE touchscreen question Do you wear glasses or contact lenses to correct your vision?-Variable type:binary
binary.22126.txt	1710174270056F5 forCTG.txt.gz	0.051	0.04476	1.14	0.2545	0.08215	0.01135	0.999	0.01297	0.0004528	0.008392	Doctor diagnosed hayfever or allergic rhinitis	FALSE				91787	20904	70883	UK Biobank	https://docs.google.com/spreadsheets/d/1kvPoupSzsSFBNSztMzl04xMoSC3Kcx3CrjVf4yBmESU/edit?ts=5b5f17db#gid=227859291	PHESANT Transformation:22126_0| | CAT-SINGLE | | reassignments: 9=NA | | Inc(>=10): 0(70883) | | Inc(>=10): 1(20904) | | CAT-SINGLE-BINARY | | sample 70883/20904(91787) | |-Notes:User asked Has a doctor ever told you that you have had any of the conditions below? hayfever or allergic rhinitis was one of the options listed.-Variable type:binary
binary.22127.txt	1710174270056F5 forCTG.txt.gz	0.05975	0.0494	1.21	0.2265	0.07432	0.01199	0.985	0.01351	0.02322	0.008238	Doctor diagnosed asthma	FALSE				91787	11717	80070	UK Biobank	https://docs.google.com/spreadsheets/d/1kvPoupSzsSFBNSztMzl04xMoSC3Kcx3CrjVf4yBmESU/edit?ts=5b5f17db#gid=227859291	PHESANT Transformation:22127_0| | CAT-SINGLE | | reassignments: 9=NA | | Inc(>=10): 0(80070) | | Inc(>=10): 1(11717) | | CAT-SINGLE-BINARY | | sample 80070/11717(91787) | |-Notes:User asked Has a doctor ever told you that you have had any of the conditions below? asthma was one of the options listed.-Variable type:binary
binary.22133.txt	1710174270056F5 forCTG.txt.gz	0.102	0.08814	1.157	0.2472	0.01635	0.006354	0.986	0.009854	-0.01181	0.007895	Doctor diagnosed sarcoidosis	FALSE				91787	395	91392	UK Biobank	https://docs.google.com/spreadsheets/d/1kvPoupSzsSFBNSztMzl04xMoSC3Kcx3CrjVf4yBmESU/edit?ts=5b5f17db#gid=227859291	PHESANT Transformation:22133_0| | CAT-SINGLE | | reassignments: 9=NA | | Inc(>=10): 0(91392) | | Inc(>=10): 1(395) | | CAT-SINGLE-BINARY | | sample 91392/395(91787) | |-Notes:User asked Has a doctor ever told you that you have had any of the conditions below? sarcoidosis was one of the options listed.-Variable type:binary
binary.2227.txt	1710174270056F5 forCTG.txt.gz	0.4115	0.08362	4.921	8.62E-07	0.008488	0.001903	1.026	0.0108	-0.008767	0.00856	Other eye problems	FALSE	Ophthalmic			360134	51720	308414	UK Biobank	https://docs.google.com/spreadsheets/d/1kvPoupSzsSFBNSztMzl04xMoSC3Kcx3CrjVf4yBmESU/edit?ts=5b5f17db#gid=227859291	PHESANT Transformation:2227_0| | CAT-SINGLE | | Inc(>=10): 1(51720) | | Inc(>=10): 0(308414) | | CAT-SINGLE-BINARY | | sample 308414/51720(360134) | |-Notes:ACE touchscreen question Do you have any other problems with your eyes or eyesight?-Variable type:binary
binary.2247_0.txt	1710174270056F5 forCTG.txt.gz	-0.2531	0.03477	-7.279	3.36E-13	0.04134	0.002887	1.014	0.01281	-0.00758	0.008812	Hearing difficulty/problems: No	FALSE	Auditory			346635	255838	90797	UK Biobank	https://docs.google.com/spreadsheets/d/1kvPoupSzsSFBNSztMzl04xMoSC3Kcx3CrjVf4yBmESU/edit?ts=5b5f17db#gid=227859291	PHESANT Transformation:2247_0 | | CAT-SINGLE | | CAT-SINGLE-BINARY-VAR: 0 | | Inc(>=10): 0(255838) | |-Notes:ACE touchscreen question Do you have any difficulty with your hearing? The option to identify oneself as completely deaf was added in 2009 alongside the introduction of the speech-in-noise test.-Variable type:binary

binary.2247_1.txt	1710174270056F5forCTG.txt.gz	0.2532	0.03476	7.283	3.26E-13	0.04137	0.002863	1.014	0.01273	0.007377	0.008806	Hearing difficulty/problems: Yes	TRUE	Auditory	Physical health	Hearing difficulty	346635	90710	255925	UK Biobank	https://docs.google.com/spreadsheets/d/1kvPoupSzsSFBNSztMzl04xMoSC3Kcx3CrjVf4yBmESU/edit?ts=5b5f17db#gid=227859291	PHESANT Transformation:2247_0 | | CAT-SINGLE | | CAT-SINGLE-BINARY-VAR: 1 | | Inc(>=10): 1(90710) | |-Notes:ACE touchscreen question Do you have any difficulty with your hearing? The option to identify oneself as completely deaf was added in 2009 alongside the introduction of the speech-in-noise test.-Variable type:binary
binary.22502.txt	1710174270056F5forCTG.txt.gz	0.2639	0.06394	4.128	3.66E-05	0.04561	0.006946	0.998	0.009681	0.004005	0.008842	Cough on most days	FALSE				91787	12593	79194	UK Biobank	https://docs.google.com/spreadsheets/d/1kvPoupSzsSFBNSztMzl04xMoSC3Kcx3CrjVf4yBmESU/edit?ts=5b5f17db#gid=227859291	PHESANT Transformation:22502_0| | CAT-SINGLE | | reassignments: 9=NA | | Inc(>=10): 1(12593) | | Inc(>=10): 0(79194) | | CAT-SINGLE-BINARY | | sample 79194/12593(91787) | |-Notes:Participant asked Do you cough on most days?-Variable type:binary
binary.22504.txt	1710174270056F5forCTG.txt.gz	0.3991	0.09663	4.13	3.62E-05	0.02406	0.00658	1.015	0.01112	0.0008903	0.00812	Bring up phlegm/sputum/mucus on most days	FALSE				91787	7808	83979	UK Biobank	https://docs.google.com/spreadsheets/d/1kvPoupSzsSFBNSztMzl04xMoSC3Kcx3CrjVf4yBmESU/edit?ts=5b5f17db#gid=227859291	PHESANT Transformation:22504_0| | CAT-SINGLE | | reassignments: 9=NA | | Inc(>=10): 1(7808) | | Inc(>=10): 0(83979) | | CAT-SINGLE-BINARY | | sample 83979/7808(91787) | |-Notes:Participant asked Do you bring up phlegm/sputum/mucus on most days?-Variable type:binary
binary.22506_112.txt	1710174270056F5forCTG.txt.gz	-0.04653	0.1245	-0.3737	0.7086	0.01053	0.005202	0.995	0.008712	0.008759	0.007976	Tobacco smoking: Occasionally	FALSE				91353	1374	89979	UK Biobank	https://docs.google.com/spreadsheets/d/1kvPoupSzsSFBNSztMzl04xMoSC3Kcx3CrjVf4yBmESU/edit?ts=5b5f17db#gid=227859291	PHESANT Transformation:22506_0 | | CAT-SINGLE | | CAT-SINGLE-BINARY-VAR: 112 | | Inc(>=10): 112(1374) | |-Notes:Participants asked Do you smoke tobacco now?-Variable type:binary
binary.22506_113.txt	1710174270056F5forCTG.txt.gz	0.1969	0.04905	4.015	5.95E-05	0.06783	0.009033	1.019	0.01166	-0.001134	0.007986	Tobacco smoking: Ex-smoker	FALSE				91353	33335	58018	UK Biobank	https://docs.google.com/spreadsheets/d/1kvPoupSzsSFBNSztMzl04xMoSC3Kcx3CrjVf4yBmESU/edit?ts=5b5f17db#gid=227859291	PHESANT Transformation:22506_0 | | CAT-SINGLE | | CAT-SINGLE-BINARY-VAR: 113 | | Inc(>=10): 113(33335) | |-Notes:Participants asked Do you smoke tobacco now?-Variable type:binary
binary.22506_114.txt	1710174270056F5forCTG.txt.gz	-0.1885	0.04425	-4.261	2.03E-05	0.08052	0.009736	1.027	0.01259	-0.001856	0.008315	Tobacco smoking: Never smoked	FALSE	Lifestyle			91353	54520	36833	UK Biobank	https://docs.google.com/spreadsheets/d/1kvPoupSzsSFBNSztMzl04xMoSC3Kcx3CrjVf4yBmESU/edit?ts=5b5f17db#gid=227859291	PHESANT Transformation:22506_0 | | CAT-SINGLE | | CAT-SINGLE-BINARY-VAR: 114 | | Inc(>=10): 114(54520) | |-Notes:Participants asked Do you smoke tobacco now?-Variable type:binary
binary.2257.txt	1710174270056F5forCTG.txt.gz	0.2239	0.03171	7.06	1.66E-12	0.05102	0.003004	1.025	0.01351	0.006789	0.008143	Hearing difficulty/problems with background noise	FALSE	Auditory			353983	134141	219842	UK Biobank	https://docs.google.com/spreadsheets/d/1kvPoupSzsSFBNSztMzl04xMoSC3Kcx3CrjVf4yBmESU/edit?ts=5b5f17db#gid=227859291	PHESANT Transformation:2257_0| | CAT-SINGLE | | Inc(>=10): 0(219842) | | Inc(>=10): 1(134141) | | CAT-SINGLE-BINARY | | sample 219842/134141(353983) | |-Notes:ACE touchscreen question Do you find it difficult to follow a conversation if there is background noise (such as TV, radio, children playing)? ~F2257~ was collected from all participants except those who indicated they were completely deaf, as defined by their answers to ~F2247~-Variable type:binary
binary.22601_11853100.txt	1710174270056F5forCTG.txt.gz	0.09483	0.1134	0.8365	0.4029	0.012	0.00594	0.994	0.009126	-0.000515	0.008166	Job coding: residential manager, day care manager, nursing home manager, retirement home manager, convalescent home manager	FALSE				89866	318	89548	UK Biobank	https://docs.google.com/spreadsheets/d/1kvPoupSzsSFBNSztMzl04xMoSC3Kcx3CrjVf4yBmESU/edit?ts=5b5f17db#gid=227859291	PHESANT Transformation:22601_0 | | CAT-MUL-BINARY-VAR 11853100 | | Indicator name x22599_0_0 | | Remove indicator var NAs: 271328 | | Remove indicator var <0: 0 | | Removed 0 examples != 11853100 but with missing value (<0) | | sample 89548/318(89866) | |-Notes:Participants were asked to select a job group by navigating down a three-level tree. This field records the code they selected at the final level of the tree.-Variable type:binary
binary.22601_12233383.txt	1710174270056F5forCTG.txt.gz	0.1782	0.1022	1.743	0.08139	0.01336	0.006725	0.983	0.01014	-2.71E-05	0.007656	Job coding: restaurant or catering manager, restaurateur; canteen manager; takeaway food shop manager	FALSE				89866	560	89306	UK Biobank	https://docs.google.com/spreadsheets/d/1kvPoupSzsSFBNSztMzl04xMoSC3Kcx3CrjVf4yBmESU/edit?ts=5b5f17db#gid=227859291	PHESANT Transformation:22601_0 | | CAT-MUL-BINARY-VAR 12233383 | | Indicator name x22599_0_0 | | Remove indicator var NAs: 271328 | | Remove indicator var <0: 0 | | Removed 0 examples != 12233383 but with missing value (<0) | | sample 89306/560(89866) | |-Notes:Participants were asked to select a job group by navigating down a three-level tree. This field records the code they selected at the final level of the tree.-Variable type:binary
binary.22601_21113020.txt	1710174270056F5forCTG.txt.gz	-0.1659	0.09882	-1.679	0.09317	0.01264	0.006354	0.997	0.009803	0.003953	0.007781	Job coding: chemist (analytic, developmental, industrial, research)	FALSE				89866	1061	88805	UK Biobank	https://docs.google.com/spreadsheets/d/1kvPoupSzsSFBNSztMzl04xMoSC3Kcx3CrjVf4yBmESU/edit?ts=5b5f17db#gid=227859291	PHESANT Transformation:22601_0 | | CAT-MUL-BINARY-VAR 21113020 | | Indicator name x22599_0_0 | | Remove indicator var NAs: 271328 | | Remove indicator var <0: 0 | | Removed 0 examples != 21113020 but with missing value (<0) | | sample 88805/1061(89866) | |-Notes:Participants were asked to select a job group by navigating down a three-level tree. This field records the code they selected at the final level of the tree.-Variable type:binary
binary.22601_21123021.txt	1710174270056F5forCTG.txt.gz	-0.09148	0.1106	-0.8272	0.4081	0.01181	0.006067	0.989	0.008817	-0.00013	0.007603	Job coding: biologist, botanist, entomologist, zoologist, ecologist, microbiologist/pathologist/virologist/geneticist (not medically qualified)	FALSE				89866	646	89220	UK Biobank	https://docs.google.com/spreadsheets/d/1kvPoupSzsSFBNSztMzl04xMoSC3Kcx3CrjVf4yBmESU/edit?ts=5b5f17db#gid=227859291	PHESANT Transformation:22601_0 | | CAT-MUL-BINARY-VAR 21123021 | | Indicator name x22599_0_0 | | Remove indicator var NAs: 271328 | | Remove indicator var <0: 0 | | Removed 0 examples != 21123021 but with missing value (<0) | | sample 89220/646(89866) | |-Notes:Participants were asked to select a job group by navigating down a three-level tree. This field records the code they selected at the final level of the tree.-Variable type:binary
binary.22601_21133023.txt	1710174270056F5forCTG.txt.gz	-0.0922	0.1147	-0.8036	0.4216	0.01148	0.006163	0.993	0.008583	-0.00704	0.008228	Job coding: physicist, astronomer, geologist, geophysicist, meteorologist, oceanographer, seismologist	FALSE				89866	585	89281	UK Biobank	https://docs.google.com/spreadsheets/d/1kvPoupSzsSFBNSztMzl04xMoSC3Kcx3CrjVf4yBmESU/edit?ts=5b5f17db#gid=227859291	PHESANT Transformation:22601_0 | | CAT-MUL-BINARY-VAR 21133023 | | Indicator name x22599_0_0 | | Remove indicator var NAs: 271328 | | Remove indicator var <0: 0 | | Removed 0 examples != 21133023 but with missing value (<0) | | sample 89281/585(89866) | |-Notes:Participants were asked to select a job group by navigating down a three-level tree. This field records the code they selected at the final level of the tree.-Variable type:binary
binary.22601_21133024.txt	1710174270056F5forCTG.txt.gz	0.0673	0.1524	0.4415	0.6588	0.007651	0.006178	1	0.01185	-0.00664	0.009518	Job coding: mathematician	FALSE				89866	144	89722	UK Biobank	https://docs.google.com/spreadsheets/d/1kvPoupSzsSFBNSztMzl04xMoSC3Kcx3CrjVf4yBmESU/edit?ts=5b5f17db#gid=227859291	PHESANT Transformation:22601_0 | | CAT-MUL-BINARY-VAR 21133024 | | Indicator name x22599_0_0 | | Remove indicator var NAs: 271328 | | Remove indicator var <0: 0 | | Removed 0 examples != 21133024 but with missing value (<0) | | sample 89722/144(89866) | |-Notes:Participants were asked to select a job group by navigating down a three-level tree. This field records the code they selected at the final level of the tree.-Variable type:binary
binary.22601_21212592.txt	1710174270056F5forCTG.txt.gz	-0.01379	0.09417	-0.1465	0.8836	0.01535	0.00648	0.984	0.009909	-0.01285	0.008052	Job coding: civil engineer	FALSE				89866	483	89383	UK Biobank	https://docs.google.com/spreadsheets/d/1kvPoupSzsSFBNSztMzl04xMoSC3Kcx3CrjVf4yBmESU/edit?ts=5b5f17db#gid=227859291	PHESANT Transformation:22601_0 | | CAT-MUL-BINARY-VAR 21212592 | | Indicator name x22599_0_0 | | Remove indicator var NAs: 271328 | | Remove indicator var <0: 0 | | Removed 0 examples != 21212592 but with missing value (<0) | | sample 89383/483(89866) | |-Notes:Participants were asked to select a job group by navigating down a three-level tree. This field records the code they selected at the final level of the tree.-Variable type:binary
binary.22601_21323059.txt	1710174270056F5forCTG.txt.gz	-0.2336	0.1478	-1.58	0.1141	0.009717	0.006767	1.024	0.01009	-0.00973	0.008017	Job coding: software professional, analyst-programmer, computer programmer, software analyst or engineer, systems designer or programmer	FALSE				89866	3445	86421	UK Biobank	https://docs.google.com/spreadsheets/d/1kvPoupSzsSFBNSztMzl04xMoSC3Kcx3CrjVf4yBmESU/edit?ts=5b5f17db#gid=227859291	PHESANT Transformation:22601_0 | | CAT-MUL-BINARY-VAR 21323059 | | Indicator name x22599_0_0 | | Remove indicator var NAs: 271328 | | Remove indicator var <0: 0 | | Removed 0 examples != 21323059 but with missing value (<0) | | sample 86421/3445(89866) | |-Notes:Participants were asked to select a job group by navigating down a three-level tree. This field records the code they selected at the final level of the tree.-Variable type:binary
binary.22601_22113066.txt	1710174270056F5forCTG.txt.gz	-0.1825	0.08138	-2.242	0.02496	0.02322	0.006044	1.001	0.008434	-0.01135	0.008374	Job coding: medical doctor, general practitioner, hospital consultant	FALSE				89866	1467	88399	UK Biobank	https://docs.google.com/spreadsheets/d/1kvPoupSzsSFBNSztMzl04xMoSC3Kcx3CrjVf4yBmESU/edit?ts=5b5f17db#gid=227859291	PHESANT Transformation:22601_0 | | CAT-MUL-BINARY-VAR 22113066 | | Indicator name x22599_0_0 | | Remove indicator var NAs: 271328 | | Remove indicator var <0: 0 | | Removed 0 examples != 22113066 but with missing value (<0) | | sample 88399/1467(89866) | |-Notes:Participants were asked to select a job group by navigating down a three-level tree. This field records the code they selected at the final level of the tree.-Variable type:binary

<table>
<tr><td>binary.22601_23113399.txt</td><td>1710174270056F5forCTG.txt.gz</td><td>-0.0976</td><td>0.06933</td><td>-1.408</td><td>0.1592</td><td>0.02801</td><td>0.006609</td><td>0.976</td><td>0.009184</td><td>-0.003811</td><td>0.007804</td><td>Job coding: higher education teaching professional, university lecturer/professor (including college/university head/vice chancellor)</td><td>FALSE</td><td></td><td></td><td></td><td>89866</td><td>2631</td><td>87235</td><td>UK Biobank</td><td>https://docs.google.com/spreadsheets/d/1kvPoupSzsSFBNSztMzl04xMoSC3Kcx3CrjVf4yBmESU/edit?ts=5b5f17db#gid=227859291</td><td>PHESANT Transformation:22601_0 | | CAT-MUL-BINARY-VAR 23113399 | | Indicator name x22599_0_0 | | Remove indicator var NAs: 271328 | | Remove indicator var <0: 0 | | Removed 0 examples != 23113399 but with missing value (<0) | | sample 87235/2631(89866) | | -Notes:Participants were asked to select a job group by navigating down a three-level tree. This field records the code they selected at the final level of the tree.-Variable type:binary</td></tr>
<tr><td>binary.22601_23143401.txt</td><td>1710174270056F5forCTG.txt.gz</td><td>-0.2088</td><td>0.08322</td><td>-2.509</td><td>0.01211</td><td>0.02197</td><td>0.005807</td><td>1.002</td><td>0.009353</td><td>-0.01516</td><td>0.007812</td><td>Job coding: secondary school teacher or teaching professional (including head teacher)</td><td>FALSE</td><td></td><td></td><td></td><td>89866</td><td>7556</td><td>82310</td><td>UK Biobank</td><td>https://docs.google.com/spreadsheets/d/1kvPoupSzsSFBNSztMzl04xMoSC3Kcx3CrjVf4yBmESU/edit?ts=5b5f17db#gid=227859291</td><td>PHESANT Transformation:22601_0 | | CAT-MUL-BINARY-VAR 23143401 | | Indicator name x22599_0_0 | | Remove indicator var NAs: 271328 | | Remove indicator var <0: 0 | | Removed 0 examples != 23143401 but with missing value (<0) | | sample 82310/7556(89866) | | -Notes:Participants were asked to select a job group by navigating down a three-level tree. This field records the code they selected at the final level of the tree.-Variable type:binary</td></tr>
<tr><td>binary.22601_23153402.txt</td><td>1710174270056F5forCTG.txt.gz</td><td>-0.2116</td><td>0.08715</td><td>-2.428</td><td>0.01518</td><td>0.02256</td><td>0.00644</td><td>0.984</td><td>0.009788</td><td>-0.000131</td><td>0.008854</td><td>Job coding: primary /junior school teacher or teaching professional, nursery school teacher (including head teacher)</td><td>FALSE</td><td></td><td></td><td></td><td>89866</td><td>5555</td><td>84311</td><td>UK Biobank</td><td>https://docs.google.com/spreadsheets/d/1kvPoupSzsSFBNSztMzl04xMoSC3Kcx3CrjVf4yBmESU/edit?ts=5b5f17db#gid=227859291</td><td>PHESANT Transformation:22601_0 | | CAT-MUL-BINARY-VAR 23153402 | | Indicator name x22599_0_0 | | Remove indicator var NAs: 271328 | | Remove indicator var <0: 0 | | Removed 0 examples != 23153402 but with missing value (<0) | | sample 84311/5555(89866) | | -Notes:Participants were asked to select a job group by navigating down a three-level tree. This field records the code they selected at the final level of the tree.-Variable type:binary</td></tr>
<tr><td>binary.22601_23213026.txt</td><td>1710174270056F5forCTG.txt.gz</td><td>-0.04948</td><td>0.0962</td><td>-0.5144</td><td>0.607</td><td>0.01123</td><td>0.006071</td><td>1.002</td><td>0.009084</td><td>-0.000588</td><td>0.006764</td><td>Job coding: scientific researcher, scientific officer, medical research associate, experimental officer</td><td>FALSE</td><td></td><td></td><td></td><td>89866</td><td>1165</td><td>88701</td><td>UK Biobank</td><td>https://docs.google.com/spreadsheets/d/1kvPoupSzsSFBNSztMzl04xMoSC3Kcx3CrjVf4yBmESU/edit?ts=5b5f17db#gid=227859291</td><td>PHESANT Transformation:22601_0 | | CAT-MUL-BINARY-VAR 23213026 | | Indicator name x22599_0_0 | | Remove indicator var NAs: 271328 | | Remove indicator var <0: 0 | | Removed 0 examples != 23213026 but with missing value (<0) | | sample 88701/1165(89866) | | -Notes:Participants were asked to select a job group by navigating down a three-level tree. This field records the code they selected at the final level of the tree.-Variable type:binary</td></tr>
<tr><td>binary.22601_23293164.txt</td><td>1710174270056F5forCTG.txt.gz</td><td>-0.1116</td><td>0.08613</td><td>-1.296</td><td>0.195</td><td>0.02249</td><td>0.006311</td><td>0.964</td><td>0.01038</td><td>0.001738</td><td>0.008696</td><td>Job coding: researcher in broadcasting, journalism, photography, printing and publishing</td><td>FALSE</td><td></td><td></td><td></td><td>89866</td><td>162</td><td>89704</td><td>UK Biobank</td><td>https://docs.google.com/spreadsheets/d/1kvPoupSzsSFBNSztMzl04xMoSC3Kcx3CrjVf4yBmESU/edit?ts=5b5f17db#gid=227859291</td><td>PHESANT Transformation:22601_0 | | CAT-MUL-BINARY-VAR 23293164 | | Indicator name x22599_0_0 | | Remove indicator var NAs: 271328 | | Remove indicator var <0: 0 | | Removed 0 examples != 23293164 but with missing value (<0) | | sample 89704/162(89866) | | -Notes:Participants were asked to select a job group by navigating down a three-level tree. This field records the code they selected at the final level of the tree.-Variable type:binary</td></tr>
<tr><td>binary.22601_24323289.txt</td><td>1710174270056F5forCTG.txt.gz</td><td>-0.06065</td><td>0.09059</td><td>-0.6695</td><td>0.5032</td><td>0.01547</td><td>0.006655</td><td>0.984</td><td>0.01084</td><td>0.001421</td><td>0.008025</td><td>Job coding: town planner, development officer</td><td>FALSE</td><td></td><td></td><td></td><td>89866</td><td>217</td><td>89649</td><td>UK Biobank</td><td>https://docs.google.com/spreadsheets/d/1kvPoupSzsSFBNSztMzl04xMoSC3Kcx3CrjVf4yBmESU/edit?ts=5b5f17db#gid=227859291</td><td>PHESANT Transformation:22601_0 | | CAT-MUL-BINARY-VAR 24323289 | | Indicator name x22599_0_0 | | Remove indicator var NAs: 271328 | | Remove indicator var <0: 0 | | Removed 0 examples != 24323289 but with missing value (<0) | | sample 89649/217(89866) | | -Notes:Participants were asked to select a job group by navigating down a three-level tree. This field records the code they selected at the final level of the tree.-Variable type:binary</td></tr>
<tr><td>binary.22601_24413202.txt</td><td>1710174270056F5forCTG.txt.gz</td><td>-0.2401</td><td>0.1404</td><td>-1.711</td><td>0.08716</td><td>0.008391</td><td>0.00607</td><td>1.008</td><td>0.009851</td><td>0.004144</td><td>0.007123</td><td>Job coding: civil service senior manager outside the senior civil service (former grades 6 and 7)</td><td>FALSE</td><td></td><td></td><td></td><td>89866</td><td>1324</td><td>88542</td><td>UK Biobank</td><td>https://docs.google.com/spreadsheets/d/1kvPoupSzsSFBNSztMzl04xMoSC3Kcx3CrjVf4yBmESU/edit?ts=5b5f17db#gid=227859291</td><td>PHESANT Transformation:22601_0 | | CAT-MUL-BINARY-VAR 24413202 | | Indicator name x22599_0_0 | | Remove indicator var NAs: 271328 | | Remove indicator var <0: 0 | | Removed 0 examples != 24413202 but with missing value (<0) | | sample 88542/1324(89866) | | -Notes:Participants were asked to select a job group by navigating down a three-level tree. This field records the code they selected at the final level of the tree.-Variable type:binary</td></tr>
<tr><td>binary.22601_32113072.txt</td><td>1710174270056F5forCTG.txt.gz</td><td>0.3192</td><td>0.1425</td><td>2.24</td><td>0.02509</td><td>0.01108</td><td>0.005608</td><td>1.006</td><td>0.008847</td><td>-0.004706</td><td>0.007797</td><td>Job coding: nurse (of any kind, at any level)</td><td>FALSE</td><td></td><td></td><td></td><td>89866</td><td>4860</td><td>85006</td><td>UK Biobank</td><td>https://docs.google.com/spreadsheets/d/1kvPoupSzsSFBNSztMzl04xMoSC3Kcx3CrjVf4yBmESU/edit?ts=5b5f17db#gid=227859291</td><td>PHESANT Transformation:22601_0 | | CAT-MUL-BINARY-VAR 32113072 | | Indicator name x22599_0_0 | | Remove indicator var NAs: 271328 | | Remove indicator var <0: 0 | | Removed 0 examples != 32113072 but with missing value (<0) | | sample 85006/4860(89866) | | -Notes:Participants were asked to select a job group by navigating down a three-level tree. This field records the code they selected at the final level of the tree.-Variable type:binary</td></tr>
<tr><td>binary.22601_33113434.txt</td><td>1710174270056F5forCTG.txt.gz</td><td>0.5011</td><td>0.2217</td><td>2.26</td><td>0.0238</td><td>0.007393</td><td>0.006054</td><td>1.013</td><td>0.009347</td><td>0.01106</td><td>0.007523</td><td>Job coding: non-commissioned officers or other rank of armed forces</td><td>FALSE</td><td></td><td></td><td></td><td>89866</td><td>2003</td><td>87863</td><td>UK Biobank</td><td>https://docs.google.com/spreadsheets/d/1kvPoupSzsSFBNSztMzl04xMoSC3Kcx3CrjVf4yBmESU/edit?ts=5b5f17db#gid=227859291</td><td>PHESANT Transformation:22601_0 | | CAT-MUL-BINARY-VAR 33113434 | | Indicator name x22599_0_0 | | Remove indicator var NAs: 271328 | | Remove indicator var <0: 0 | | Removed 0 examples != 33113434 but with missing value (<0) | | sample 87863/2003(89866) | | -Notes:Participants were asked to select a job group by navigating down a three-level tree. This field records the code they selected at the final level of the tree.-Variable type:binary</td></tr>
<tr><td>binary.22601_34123157.txt</td><td>1710174270056F5forCTG.txt.gz</td><td>0.001408</td><td>0.08665</td><td>0.01625</td><td>0.987</td><td>0.01765</td><td>0.006118</td><td>0.977</td><td>0.009942</td><td>-0.004352</td><td>0.008419</td><td>Job coding: author, writer, biographer, book editor, novelist, dramatist, playwright, poet</td><td>FALSE</td><td></td><td></td><td></td><td>89866</td><td>277</td><td>89589</td><td>UK Biobank</td><td>https://docs.google.com/spreadsheets/d/1kvPoupSzsSFBNSztMzl04xMoSC3Kcx3CrjVf4yBmESU/edit?ts=5b5f17db#gid=227859291</td><td>PHESANT Transformation:22601_0 | | CAT-MUL-BINARY-VAR 34123157 | | Indicator name x22599_0_0 | | Remove indicator var NAs: 271328 | | Remove indicator var <0: 0 | | Removed 0 examples != 34123157 but with missing value (<0) | | sample 89589/277(89866) | | -Notes:Participants were asked to select a job group by navigating down a three-level tree. This field records the code they selected at the final level of the tree.-Variable type:binary</td></tr>
<tr><td>binary.22601_34313159.txt</td><td>1710174270056F5forCTG.txt.gz</td><td>-0.002158</td><td>0.1024</td><td>-0.02107</td><td>0.9832</td><td>0.01351</td><td>0.005569</td><td>0.994</td><td>0.008919</td><td>-0.01131</td><td>0.007843</td><td>Job coding: journalist, reporter, newspaper correspondent, sports writer, newspaper or magazine editor</td><td>FALSE</td><td></td><td></td><td></td><td>89866</td><td>496</td><td>89370</td><td>UK Biobank</td><td>https://docs.google.com/spreadsheets/d/1kvPoupSzsSFBNSztMzl04xMoSC3Kcx3CrjVf4yBmESU/edit?ts=5b5f17db#gid=227859291</td><td>PHESANT Transformation:22601_0 | | CAT-MUL-BINARY-VAR 34313159 | | Indicator name x22599_0_0 | | Remove indicator var NAs: 271328 | | Remove indicator var <0: 0 | | Removed 0 examples != 34313159 but with missing value (<0) | | sample 89370/496(89866) | | -Notes:Participants were asked to select a job group by navigating down a three-level tree. This field records the code they selected at the final level of the tree.-Variable type:binary</td></tr>
<tr><td>binary.22601_35132648.txt</td><td>1710174270056F5forCTG.txt.gz</td><td>-0.05209</td><td>0.2083</td><td>-0.2501</td><td>0.8025</td><td>0.003336</td><td>0.005848</td><td>0.997</td><td>0.01062</td><td>0.01014</td><td>0.008743</td><td>Job coding: ship's engineer, ship's purser</td><td>FALSE</td><td></td><td></td><td></td><td>89866</td><td>155</td><td>89711</td><td>UK Biobank</td><td>https://docs.google.com/spreadsheets/d/1kvPoupSzsSFBNSztMzl04xMoSC3Kcx3CrjVf4yBmESU/edit?ts=5b5f17db#gid=227859291</td><td>PHESANT Transformation:22601_0 | | CAT-MUL-BINARY-VAR 35132648 | | Indicator name x22599_0_0 | | Remove indicator var NAs: 271328 | | Remove indicator var <0: 0 | | Removed 0 examples != 35132648 but with missing value (<0) | | sample 89711/155(89866) | | -Notes:Participants were asked to select a job group by navigating down a three-level tree. This field records the code they selected at the final level of the tree.-Variable type:binary</td></tr>
<tr><td>binary.22601_35393271.txt</td><td>1710174270056F5forCTG.txt.gz</td><td>-0.09892</td><td>0.08859</td><td>-1.117</td><td>0.2642</td><td>0.01454</td><td>0.006754</td><td>0.993</td><td>0.009803</td><td>-0.003763</td><td>0.007913</td><td>Job coding: management information officer, conference/events co-ordinator/organiser, exhibition officer, work study engineer/officer/analyst, contract adviser/agent, election agent, business system analyst</td><td>FALSE</td><td></td><td></td><td></td><td>89866</td><td>1857</td><td>88009</td><td>UK Biobank</td><td>https://docs.google.com/spreadsheets/d/1kvPoupSzsSFBNSztMzl04xMoSC3Kcx3CrjVf4yBmESU/edit?ts=5b5f17db#gid=227859291</td><td>PHESANT Transformation:22601_0 | | CAT-MUL-BINARY-VAR 35393271 | | Indicator name x22599_0_0 | | Remove indicator var NAs: 271328 | | Remove indicator var <0: 0 | | Removed 0 examples != 35393271 but with missing value (<0) | | sample 88009/1857(89866) | | -Notes:Participants were asked to select a job group by navigating down a three-level tree. This field records the code they selected at the final level of the tree.-Variable type:binary</td></tr>
</table>

<table>
<tr><td>binary.22601_41143216.txt</td><td>1710174270056F5forCTG.txt.gz</td><td>0.01285</td><td>0.0913</td><td>0.1408</td><td>0.888</td><td>0.0165</td><td>0.006344</td><td>0.984</td><td>0.009805</td><td>-0.02258</td><td>0.008207</td><td>Job coding: officer of ngo, trade union organiser/official, charity administrator, secretary of research or charity or political or professional or trade association or trade union</td><td>FALSE</td><td></td><td></td><td></td><td>89866</td><td>1557</td><td>88309</td><td>UK Biobank</td><td>https://docs.google.com/spreadsheets/d/1kvPoupSzsSFBNSztMzl04xMoSC3Kcx3CrjVf4yBmESU/edit?ts=5b5f17db#gid=227859291</td><td>PHESANT Transformation:22601_0 | | CAT-MUL-BINARY-VAR 41143216 | | Indicator name x22599_0_0 | | Remove indicator var NAs: 271328 | | Remove indicator var <0: 0 | | Removed 0 examples != 41143216 but with missing value (<0) | | sample 88309/1557(89866) | |-Notes:Participants were asked to select a job group by navigating down a three-level tree. This field records the code they selected at the final level of the tree.-Variable type:binary</td></tr>
<tr><td>binary.22601_41503307.txt</td><td>1710174270056F5forCTG.txt.gz</td><td>-0.1371</td><td>0.1171</td><td>-1.171</td><td>0.2416</td><td>0.0122</td><td>0.006221</td><td>0.996</td><td>0.01016</td><td>0.0002329</td><td>0.008436</td><td>Job coding: general office assistant/clerk, clerical officer, clerk-typist, office supervisor, press/newspaper corrector/reader</td><td>FALSE</td><td></td><td></td><td></td><td>89866</td><td>3957</td><td>85909</td><td>UK Biobank</td><td>https://docs.google.com/spreadsheets/d/1kvPoupSzsSFBNSztMzl04xMoSC3Kcx3CrjVf4yBmESU/edit?ts=5b5f17db#gid=227859291</td><td>PHESANT Transformation:22601_0 | | CAT-MUL-BINARY-VAR 41503307 | | Indicator name x22599_0_0 | | Remove indicator var NAs: 271328 | | Remove indicator var <0: 0 | | Removed 0 examples != 41503307 but with missing value (<0) | | sample 85909/3957(89866) | |-Notes:Participants were asked to select a job group by navigating down a three-level tree. This field records the code they selected at the final level of the tree.-Variable type:binary</td></tr>
<tr><td>binary.22601_42153302.txt</td><td>1710174270056F5forCTG.txt.gz</td><td>-0.146</td><td>0.08199</td><td>-1.781</td><td>0.0749</td><td>0.02202</td><td>0.0061</td><td>0.981</td><td>0.009306</td><td>0.001421</td><td>0.008288</td><td>Job coding: all other personal assistants and secretaries</td><td>FALSE</td><td></td><td></td><td></td><td>89866</td><td>5344</td><td>84522</td><td>UK Biobank</td><td>https://docs.google.com/spreadsheets/d/1kvPoupSzsSFBNSztMzl04xMoSC3Kcx3CrjVf4yBmESU/edit?ts=5b5f17db#gid=227859291</td><td>PHESANT Transformation:22601_0 | | CAT-MUL-BINARY-VAR 42153302 | | Indicator name x22599_0_0 | | Remove indicator var NAs: 271328 | | Remove indicator var <0: 0 | | Removed 0 examples != 42153302 but with missing value (<0) | | sample 84522/5344(89866) | |-Notes:Participants were asked to select a job group by navigating down a three-level tree. This field records the code they selected at the final level of the tree.-Variable type:binary</td></tr>
<tr><td>binary.22601_42163095.txt</td><td>1710174270056F5forCTG.txt.gz</td><td>-0.09682</td><td>0.1142</td><td>-0.8475</td><td>0.3967</td><td>0.01131</td><td>0.006392</td><td>0.993</td><td>0.009661</td><td>0.01066</td><td>0.008575</td><td>Job coding: receptionist, doctor's/dental receptionist</td><td>FALSE</td><td></td><td></td><td></td><td>89866</td><td>312</td><td>89554</td><td>UK Biobank</td><td>https://docs.google.com/spreadsheets/d/1kvPoupSzsSFBNSztMzl04xMoSC3Kcx3CrjVf4yBmESU/edit?ts=5b5f17db#gid=227859291</td><td>PHESANT Transformation:22601_0 | | CAT-MUL-BINARY-VAR 42163095 | | Indicator name x22599_0_0 | | Remove indicator var NAs: 271328 | | Remove indicator var <0: 0 | | Removed 0 examples != 42163095 but with missing value (<0) | | sample 89554/312(89866) | |-Notes:Participants were asked to select a job group by navigating down a three-level tree. This field records the code they selected at the final level of the tree.-Variable type:binary</td></tr>
<tr><td>binary.22601_61143103.txt</td><td>1710174270056F5forCTG.txt.gz</td><td>0.261</td><td>0.104</td><td>2.509</td><td>0.01211</td><td>0.01702</td><td>0.006584</td><td>0.996</td><td>0.008962</td><td>-0.01624</td><td>0.007949</td><td>Job coding: houseparent, child careworker, residential warden, sheltered accommodation warden, foster parent</td><td>FALSE</td><td></td><td></td><td></td><td>89866</td><td>633</td><td>89233</td><td>UK Biobank</td><td>https://docs.google.com/spreadsheets/d/1kvPoupSzsSFBNSztMzl04xMoSC3Kcx3CrjVf4yBmESU/edit?ts=5b5f17db#gid=227859291</td><td>PHESANT Transformation:22601_0 | | CAT-MUL-BINARY-VAR 61143103 | | Indicator name x22599_0_0 | | Remove indicator var NAs: 271328 | | Remove indicator var <0: 0 | | Removed 0 examples != 61143103 but with missing value (<0) | | sample 89233/633(89866) | |-Notes:Participants were asked to select a job group by navigating down a three-level tree. This field records the code they selected at the final level of the tree.-Variable type:binary</td></tr>
<tr><td>binary.22601_81112700.txt</td><td>1710174270056F5forCTG.txt.gz</td><td>0.06476</td><td>0.09655</td><td>0.6707</td><td>0.5024</td><td>0.01562</td><td>0.00627</td><td>0.986</td><td>0.01029</td><td>0.01597</td><td>0.008632</td><td>Job coding: machine operator, processor, foreman</td><td>FALSE</td><td></td><td></td><td></td><td>89866</td><td>243</td><td>89623</td><td>UK Biobank</td><td>https://docs.google.com/spreadsheets/d/1kvPoupSzsSFBNSztMzl04xMoSC3Kcx3CrjVf4yBmESU/edit?ts=5b5f17db#gid=227859291</td><td>PHESANT Transformation:22601_0 | | CAT-MUL-BINARY-VAR 81112700 | | Indicator name x22599_0_0 | | Remove indicator var NAs: 271328 | | Remove indicator var <0: 0 | | Removed 0 examples != 81112700 but with missing value (<0) | | sample 89623/243(89866) | |-Notes:Participants were asked to select a job group by navigating down a three-level tree. This field records the code they selected at the final level of the tree.-Variable type:binary</td></tr>
<tr><td>binary.22601_81322815.txt</td><td>1710174270056F5forCTG.txt.gz</td><td>0.1383</td><td>0.1294</td><td>1.068</td><td>0.2855</td><td>0.008154</td><td>0.005463</td><td>0.989</td><td>0.009194</td><td>0.002469</td><td>0.008881</td><td>Job coding: assembler, machine operator, foreman</td><td>FALSE</td><td></td><td></td><td></td><td>89866</td><td>216</td><td>89650</td><td>UK Biobank</td><td>https://docs.google.com/spreadsheets/d/1kvPoupSzsSFBNSztMzl04xMoSC3Kcx3CrjVf4yBmESU/edit?ts=5b5f17db#gid=227859291</td><td>PHESANT Transformation:22601_0 | | CAT-MUL-BINARY-VAR 81322815 | | Indicator name x22599_0_0 | | Remove indicator var NAs: 271328 | | Remove indicator var <0: 0 | | Removed 0 examples != 81322815 but with missing value (<0) | | sample 89650/216(89866) | |-Notes:Participants were asked to select a job group by navigating down a three-level tree. This field records the code they selected at the final level of the tree.-Variable type:binary</td></tr>
<tr><td>binary.22601_82112603.txt</td><td>1710174270056F5forCTG.txt.gz</td><td>0.3333</td><td>0.1262</td><td>2.641</td><td>0.008268</td><td>0.01225</td><td>0.005801</td><td>0.991</td><td>0.009446</td><td>-0.008877</td><td>0.00777</td><td>Job coding: heavy goods vehicle (hgv) driver, lorry or truck driver, tanker driver, haulage driver</td><td>FALSE</td><td></td><td></td><td></td><td>89866</td><td>671</td><td>89195</td><td>UK Biobank</td><td>https://docs.google.com/spreadsheets/d/1kvPoupSzsSFBNSztMzl04xMoSC3Kcx3CrjVf4yBmESU/edit?ts=5b5f17db#gid=227859291</td><td>PHESANT Transformation:22601_0 | | CAT-MUL-BINARY-VAR 82112603 | | Indicator name x22599_0_0 | | Remove indicator var NAs: 271328 | | Remove indicator var <0: 0 | | Removed 0 examples != 82112603 but with missing value (<0) | | sample 89195/671(89866) | |-Notes:Participants were asked to select a job group by navigating down a three-level tree. This field records the code they selected at the final level of the tree.-Variable type:binary</td></tr>
<tr><td>binary.22601_82152612.txt</td><td>1710174270056F5forCTG.txt.gz</td><td>0.02607</td><td>0.1077</td><td>0.242</td><td>0.8088</td><td>0.01527</td><td>0.006318</td><td>0.978</td><td>0.01102</td><td>-0.00055</td><td>0.009977</td><td>Job coding: driving instructor, hgv instructor</td><td>FALSE</td><td></td><td></td><td></td><td>89866</td><td>124</td><td>89742</td><td>UK Biobank</td><td>https://docs.google.com/spreadsheets/d/1kvPoupSzsSFBNSztMzl04xMoSC3Kcx3CrjVf4yBmESU/edit?ts=5b5f17db#gid=227859291</td><td>PHESANT Transformation:22601_0 | | CAT-MUL-BINARY-VAR 82152612 | | Indicator name x22599_0_0 | | Remove indicator var NAs: 271328 | | Remove indicator var <0: 0 | | Removed 0 examples != 82152612 but with missing value (<0) | | sample 89742/124(89866) | |-Notes:Participants were asked to select a job group by navigating down a three-level tree. This field records the code they selected at the final level of the tree.-Variable type:binary</td></tr>
<tr><td>binary.22601_92193312.txt</td><td>1710174270056F5forCTG.txt.gz</td><td>0.3244</td><td>0.1568</td><td>2.069</td><td>0.0385</td><td>0.009247</td><td>0.006338</td><td>1.005</td><td>0.009394</td><td>0.003073</td><td>0.008116</td><td>Job coding: other general office clerical tasks including: office junior, office worker, photocopy/print room operator, office machinist</td><td>FALSE</td><td></td><td></td><td></td><td>89866</td><td>3270</td><td>86596</td><td>UK Biobank</td><td>https://docs.google.com/spreadsheets/d/1kvPoupSzsSFBNSztMzl04xMoSC3Kcx3CrjVf4yBmESU/edit?ts=5b5f17db#gid=227859291</td><td>PHESANT Transformation:22601_0 | | CAT-MUL-BINARY-VAR 92193312 | | Indicator name x22599_0_0 | | Remove indicator var NAs: 271328 | | Remove indicator var <0: 0 | | Removed 0 examples != 92193312 but with missing value (<0) | | sample 86596/3270(89866) | |-Notes:Participants were asked to select a job group by navigating down a three-level tree. This field records the code they selected at the final level of the tree.-Variable type:binary</td></tr>
<tr><td>binary.22604_4.txt</td><td>1710174270056F5forCTG.txt.gz</td><td>0.1518</td><td>0.08377</td><td>1.812</td><td>0.06995</td><td>0.02723</td><td>0.01009</td><td>1.001</td><td>0.0121</td><td>0.006488</td><td>0.007646</td><td>Work hours - lumped category: Over 40 hours</td><td>FALSE</td><td></td><td></td><td></td><td>75177</td><td>37201</td><td>37976</td><td>UK Biobank</td><td>https://docs.google.com/spreadsheets/d/1kvPoupSzsSFBNSztMzl04xMoSC3Kcx3CrjVf4yBmESU/edit?ts=5b5f17db#gid=227859291</td><td>PHESANT Transformation:22604_0 | | CAT-MUL-BINARY-VAR 4 | | NO_NAN Remove NA participants 286017 | | Removed 0 examples != 4 but with missing value (<0) | | sample 37976/37201(75177) | |-Notes:Participants were asked On average, how many hours a week did you work?. They were able to select a lumped category or enter a precise number of hours. This field captures the results for participants who selected a lumped category.-Variable type:binary</td></tr>
<tr><td>binary.22606_0.txt</td><td>1710174270056F5forCTG.txt.gz</td><td>-0.2192</td><td>0.06568</td><td>-3.338</td><td>0.000845</td><td>0.0287</td><td>0.00627</td><td>0.997</td><td>0.009239</td><td>-0.01147</td><td>0.007271</td><td>Workplace very noisy: Rarely/never</td><td>FALSE</td><td></td><td></td><td></td><td>90897</td><td>69515</td><td>21382</td><td>UK Biobank</td><td>https://docs.google.com/spreadsheets/d/1kvPoupSzsSFBNSztMzl04xMoSC3Kcx3CrjVf4yBmESU/edit?ts=5b5f17db#gid=227859291</td><td>PHESANT Transformation:22606_0 | | CAT-MUL-BINARY-VAR 0 | | NO_NAN Remove NA participants 270211 | | Removed 86 examples != 0 but with missing value (<0) | | sample 21382/69515(90897) | |-Notes:Participant asked Thinking about the place where you worked: Was it very noisy? Participants were allowed to skip answering this question.-Variable type:binary</td></tr>
<tr><td>binary.22606_1.txt</td><td>1710174270056F5forCTG.txt.gz</td><td>0.4429</td><td>0.08896</td><td>4.978</td><td>6.42E-07</td><td>0.02353</td><td>0.006228</td><td>1.012</td><td>0.008974</td><td>0.0004123</td><td>0.00777</td><td>Workplace very noisy: Sometimes</td><td>FALSE</td><td>Occupation</td><td></td><td></td><td>90777</td><td>51689</td><td>39088</td><td>UK Biobank</td><td>https://docs.google.com/spreadsheets/d/1kvPoupSzsSFBNSztMzl04xMoSC3Kcx3CrjVf4yBmESU/edit?ts=5b5f17db#gid=227859291</td><td>PHESANT Transformation:22606_0 | | CAT-MUL-BINARY-VAR 1 | | NO_NAN Remove NA participants 270211 | | Removed 206 examples != 1 but with missing value (<0) | | sample 39088/51689(90777) | |-Notes:Participant asked Thinking about the place where you worked: Was it very noisy? Participants were allowed to skip answering this question.-Variable type:binary</td></tr>
<tr><td>binary.22606_2.txt</td><td>1710174270056F5forCTG.txt.gz</td><td>0.4603</td><td>0.06002</td><td>7.669</td><td>1.73E-14</td><td>0.04338</td><td>0.006917</td><td>1.008</td><td>0.009633</td><td>0.01878</td><td>0.007946</td><td>Workplace very noisy: Often</td><td>FALSE</td><td>Occupation</td><td></td><td></td><td>90653</td><td>17469</td><td>73184</td><td>UK Biobank</td><td>https://docs.google.com/spreadsheets/d/1kvPoupSzsSFBNSztMzl04xMoSC3Kcx3CrjVf4yBmESU/edit?ts=5b5f17db#gid=227859291</td><td>PHESANT Transformation:22606_0 | | CAT-MUL-BINARY-VAR 2 | | NO_NAN Remove NA participants 270211 | | Removed 331 examples != 2 but with missing value (<0) | | sample 73184/17469(90653) | | SKIP_val:-121 < 0 | |-Notes:Participant asked Thinking about the place where you worked: Was it very noisy? Participants were allowed to skip answering this question.-Variable type:binary</td></tr>
<tr><td>binary.22607_0.txt</td><td>1710174270056F5forCTG.txt.gz</td><td>-0.2158</td><td>0.06323</td><td>-3.413</td><td>0.000641</td><td>0.03581</td><td>0.006304</td><td>1.001</td><td>0.008872</td><td>-0.01412</td><td>0.007898</td><td>Workplace very cold: Rarely/never</td><td>FALSE</td><td></td><td></td><td></td><td>90696</td><td>77053</td><td>13643</td><td>UK Biobank</td><td>https://docs.google.com/spreadsheets/d/1kvPoupSzsSFBNSztMzl04xMoSC3Kcx3CrjVf4yBmESU/edit?ts=5b5f17db#gid=227859291</td><td>PHESANT Transformation:22607_0 | | CAT-MUL-BINARY-VAR 0 | | NO_NAN Remove NA participants 270311 | | Removed 187 examples != 0 but with missing value (<0) | | sample 13643/77053(90696) | |-Notes:Participant asked Thinking about the place where you worked: Was it very cold? Participants were allowed to skip answering this question.-Variable type:binary</td></tr>
<tr><td>binary.22607_1.txt</td><td>1710174270056F5forCTG.txt.gz</td><td>0.3851</td><td>0.0559</td><td>6.889</td><td>5.61E-12</td><td>0.04764</td><td>0.00709</td><td>1.003</td><td>0.01089</td><td>0.01638</td><td>0.008291</td><td>Workplace very cold: Sometimes</td><td>FALSE</td><td>Occupation</td><td></td><td></td><td>90500</td><td>42823</td><td>47677</td><td>UK Biobank</td><td>https://docs.google.com/spreadsheets/d/1kvPoupSzsSFBNSztMzl04xMoSC3Kcx3CrjVf4yBmESU/edit?ts=5b5f17db#gid=227859291</td><td>PHESANT Transformation:22607_0 | | CAT-MUL-BINARY-VAR 1 | | NO_NAN Remove NA participants 270311 | | Removed 383 examples != 1 but with missing value (<0) | | sample 47677/42823(90500) | |-Notes:Participant asked Thinking about the place where you worked: Was it very cold? Participants were allowed to skip answering this question.-Variable type:binary</td></tr>
<tr><td>binary.22607_2.txt</td><td>1710174270056F5forCTG.txt.gz</td><td>0.4372</td><td>0.07607</td><td>5.747</td><td>9.09E-09</td><td>0.02842</td><td>0.006658</td><td>1.009</td><td>0.01037</td><td>0.01575</td><td>0.007736</td><td>Workplace very cold: Often</td><td>FALSE</td><td>Occupation</td><td></td><td></td><td>90188</td><td>7881</td><td>82307</td><td>UK Biobank</td><td>https://docs.google.com/spreadsheets/d/1kvPoupSzsSFBNSztMzl04xMoSC3Kcx3CrjVf4yBmESU/edit?ts=5b5f17db#gid=227859291</td><td>PHESANT Transformation:22607_0 | | CAT-MUL-BINARY-VAR 2 | | NO_NAN Remove NA participants 270311 | | Removed 696 examples != 2 but with missing value (<0) | | sample 82307/7881(90188) | | SKIP_val:-121 < 0 | |-Notes:Participant asked Thinking about the place where you worked: Was it very cold? Participants were allowed to skip answering this question.-Variable type:binary</td></tr>
</table>

binary.22608_0.txt	1710174270056F5 forCTG.txt.gz	-0.228	0.06675	-3.416	0.000635	0.02852	0.006759	1.016	0.009387	-0.005699	0.008041	Workplace very hot: Rarely/never	FALSE				90644	68705	21939	UK Biobank	https://docs.google.com/spreadsheets/d/1kvPoupSzsSFBNSztMzl04xMoSC3Kcx3CrjVf4yBmESU/edit?ts=5b5f17db#gid=227859291	PHESANT Transformation:22608_0 | | CAT-MUL-BINARY-VAR 0 | | NO_NAN Remove NA participants 270293 | | Removed 257 examples != 0 but with missing value (<0) | | sample 21939/68705(90644) | |-Notes:Participant asked Thinking about the place where you worked: Was it very hot? Participants were allowed to skip answering this question.-Variable type:binary
binary.22608_1.txt	1710174270056F5 forCTG.txt.gz	0.3786	0.06581	5.752	8.81E-09	0.03264	0.007913	1.012	0.01097	0.003638	0.007897	Workplace very hot: Sometimes	FALSE	Occupation			90544	52209	38335	UK Biobank	https://docs.google.com/spreadsheets/d/1kvPoupSzsSFBNSztMzl04xMoSC3Kcx3CrjVf4yBmESU/edit?ts=5b5f17db#gid=227859291	PHESANT Transformation:22608_0 | | CAT-MUL-BINARY-VAR 1 | | NO_NAN Remove NA participants 270293 | | Removed 357 examples != 1 but with missing value (<0) | | sample 38335/52209(90544) | |-Notes:Participant asked Thinking about the place where you worked: Was it very hot? Participants were allowed to skip answering this question.-Variable type:binary
binary.22608_2.txt	1710174270056F5 forCTG.txt.gz	0.352	0.0746	4.718	2.38E-06	0.02901	0.006532	0.995	0.009777	0.01549	0.008047	Workplace very hot: Often	FALSE	Occupation			90165	10169	79996	UK Biobank	https://docs.google.com/spreadsheets/d/1kvPoupSzsSFBNSztMzl04xMoSC3Kcx3CrjVf4yBmESU/edit?ts=5b5f17db#gid=227859291	PHESANT Transformation:22608_0 | | CAT-MUL-BINARY-VAR 2 | | NO_NAN Remove NA participants 270293 | | Removed 737 examples != 2 but with missing value (<0) | | sample 79996/10169(90165) | | SKIP_val:-121 < 0 | |-Notes:Participant asked Thinking about the place where you worked: Was it very hot? Participants were allowed to skip answering this question.-Variable type:binary
binary.22609_1.txt	1710174270056F5 forCTG.txt.gz	0.6088	0.1013	6.011	1.85E-09	0.02137	0.005999	1.011	0.009049	0.01128	0.007578	Workplace very dusty: Sometimes	FALSE	Occupation			89965	31586	58379	UK Biobank	https://docs.google.com/spreadsheets/d/1kvPoupSzsSFBNSztMzl04xMoSC3Kcx3CrjVf4yBmESU/edit?ts=5b5f17db#gid=227859291	PHESANT Transformation:22609_0 | | CAT-MUL-BINARY-VAR 1 | | NO_NAN Remove NA participants 270347 | | Removed 882 examples != 1 but with missing value (<0) | | sample 58379/31586(89965) | | SKIP_val:-121 < 0 | |-Notes:Participant asked Thinking about the place where you worked: Was it very dusty? Participants were allowed to skip answering this question.-Variable type:binary
binary.22609_2.txt	1710174270056F5 forCTG.txt.gz	0.5389	0.07413	7.269	3.62E-13	0.03211	0.006417	1.015	0.009535	0.01007	0.008339	Workplace very dusty: Often	TRUE	Occupation	Occupation	Dusty workplace	89631	9561	80070	UK Biobank	https://docs.google.com/spreadsheets/d/1kvPoupSzsSFBNSztMzl04xMoSC3Kcx3CrjVf4yBmESU/edit?ts=5b5f17db#gid=227859291	PHESANT Transformation:22609_0 | | CAT-MUL-BINARY-VAR 2 | | NO_NAN Remove NA participants 270347 | | Removed 1217 examples != 2 but with missing value (<0) | | sample 80070/9561(89631) | |-Notes:Participant asked Thinking about the place where you worked: Was it very dusty? Participants were allowed to skip answering this question.-Variable type:binary
binary.22610_0.txt	1710174270056F5 forCTG.txt.gz	-0.2873	0.08558	-3.357	0.000789	0.01879	0.006139	0.996	0.009274	-0.005931	0.007522	Workplace full of chemical or other fumes: Rarely/never	FALSE				90344	85358	4986	UK Biobank	https://docs.google.com/spreadsheets/d/1kvPoupSzsSFBNSztMzl04xMoSC3Kcx3CrjVf4yBmESU/edit?ts=5b5f17db#gid=227859291	PHESANT Transformation:22610_0 | | CAT-MUL-BINARY-VAR 0 | | NO_NAN Remove NA participants 270338 | | Removed 512 examples != 0 but with missing value (<0) | | sample 4986/85358(90344) | |-Notes:Participant asked Thinking about the place where you worked: Was it full of chemical or other fumes? Participants were allowed to skip answering this question.-Variable type:binary
binary.22610_1.txt	1710174270056F5 forCTG.txt.gz	0.5889	0.1023	5.758	8.51E-09	0.02236	0.006164	1.02	0.009068	0.01779	0.007456	Workplace full of chemical or other fumes: Sometimes	FALSE	Occupation			89060	21709	67351	UK Biobank	https://docs.google.com/spreadsheets/d/1kvPoupSzsSFBNSztMzl04xMoSC3Kcx3CrjVf4yBmESU/edit?ts=5b5f17db#gid=227859291	PHESANT Transformation:22610_0 | | CAT-MUL-BINARY-VAR 1 | | NO_NAN Remove NA participants 270338 | | Removed 1797 examples != 1 but with missing value (<0) | | sample 67351/21709(89060) | |-Notes:Participant asked Thinking about the place where you worked: Was it full of chemical or other fumes? Participants were allowed to skip answering this question.-Variable type:binary
binary.22610_2.txt	1710174270056F5 forCTG.txt.gz	0.5803	0.1474	3.937	8.24E-05	0.01389	0.006207	1.002	0.009521	0.01843	0.007718	Workplace full of chemical or other fumes: Often	FALSE				88735	5872	82863	UK Biobank	https://docs.google.com/spreadsheets/d/1kvPoupSzsSFBNSztMzl04xMoSC3Kcx3CrjVf4yBmESU/edit?ts=5b5f17db#gid=227859291	PHESANT Transformation:22610_0 | | CAT-MUL-BINARY-VAR 2 | | NO_NAN Remove NA participants 270338 | | Removed 2122 examples != 2 but with missing value (<0) | | sample 82863/5872(88735) | | SKIP_val:-121 < 0 | |-Notes:Participant asked Thinking about the place where you worked: Was it full of chemical or other fumes? Participants were allowed to skip answering this question.-Variable type:binary
binary.22611_0.txt	1710174270056F5 forCTG.txt.gz	-0.003034	0.09844	-0.03082	0.9754	0.01355	0.006068	0.998	0.009139	-0.01345	0.007339	Workplace had a lot of cigarette smoke from other people smoking: Rarely/never	FALSE				90716	77294	13422	UK Biobank	https://docs.google.com/spreadsheets/d/1kvPoupSzsSFBNSztMzl04xMoSC3Kcx3CrjVf4yBmESU/edit?ts=5b5f17db#gid=227859291	PHESANT Transformation:22611_0 | | CAT-MUL-BINARY-VAR 0 | | NO_NAN Remove NA participants 270296 | | Removed 182 examples != 0 but with missing value (<0) | | sample 13422/77294(90716) | |-Notes:Participant asked Thinking about the place where you worked: Was there a lot of cigarette smoke from other people smoking? Participants were allowed to skip answering this question.-Variable type:binary
binary.22611_1.txt	1710174270056F5 forCTG.txt.gz	0.1348	0.08404	1.604	0.1087	0.01854	0.005756	1	0.009159	0.01532	0.007814	Workplace had a lot of cigarette smoke from other people smoking: Sometimes	FALSE				90168	45053	45115	UK Biobank	https://docs.google.com/spreadsheets/d/1kvPoupSzsSFBNSztMzl04xMoSC3Kcx3CrjVf4yBmESU/edit?ts=5b5f17db#gid=227859291	PHESANT Transformation:22611_0 | | CAT-MUL-BINARY-VAR 1 | | NO_NAN Remove NA participants 270296 | | Removed 730 examples != 1 but with missing value (<0) | | sample 45115/45053(90168) | |-Notes:Participant asked Thinking about the place where you worked: Was there a lot of cigarette smoke from other people smoking? Participants were allowed to skip answering this question.-Variable type:binary
binary.22611_2.txt	1710174270056F5 forCTG.txt.gz	0.3375	0.08055	4.19	2.79E-05	0.02489	0.006654	1.01	0.009805	0.01827	0.008094	Workplace had a lot of cigarette smoke from other people smoking: Often	FALSE	Occupation			89803	14941	74862	UK Biobank	https://docs.google.com/spreadsheets/d/1kvPoupSzsSFBNSztMzl04xMoSC3Kcx3CrjVf4yBmESU/edit?ts=5b5f17db#gid=227859291	PHESANT Transformation:22611_0 | | CAT-MUL-BINARY-VAR 2 | | NO_NAN Remove NA participants 270296 | | Removed 1096 examples != 2 but with missing value (<0) | | sample 74862/14941(89803) | | SKIP_val:-121 < 0 | |-Notes:Participant asked Thinking about the place where you worked: Was there a lot of cigarette smoke from other people smoking? Participants were allowed to skip answering this question.-Variable type:binary
binary.22612_0.txt	1710174270056F5 forCTG.txt.gz	-0.505	0.1796	-2.812	0.004924	0.01029	0.006015	0.993	0.008463	0.0007777	0.008049	Worked with materials containing asbestos: Rarely/never	FALSE				85668	83506	2162	UK Biobank	https://docs.google.com/spreadsheets/d/1kvPoupSzsSFBNSztMzl04xMoSC3Kcx3CrjVf4yBmESU/edit?ts=5b5f17db#gid=227859291	PHESANT Transformation:22612_0 | | CAT-MUL-BINARY-VAR 0 | | NO_NAN Remove NA participants 270383 | | Removed 5143 examples != 0 but with missing value (<0) | | sample 2162/83506(85668) | | SKIP_val:-121 < 0 | |-Notes:Participant asked Thinking about the place where you worked: Did you work with materials containing asbestos? Participants were allowed to skip answering this question.-Variable type:binary
binary.22612_1.txt	1710174270056F5 forCTG.txt.gz	0.5624	0.1004	5.6	2.15E-08	0.02514	0.008325	1.001	0.009864	0.006086	0.008024	Worked with materials containing asbestos: Sometimes	FALSE	Occupation			77361	8397	68964	UK Biobank	https://docs.google.com/spreadsheets/d/1kvPoupSzsSFBNSztMzl04xMoSC3Kcx3CrjVf4yBmESU/edit?ts=5b5f17db#gid=227859291	PHESANT Transformation:22612_0 | | CAT-MUL-BINARY-VAR 1 | | NO_NAN Remove NA participants 270383 | | Removed 13450 examples != 1 but with missing value (<0) | | sample 68964/8397(77361) | |-Notes:Participant asked Thinking about the place where you worked: Did you work with materials containing asbestos? Participants were allowed to skip answering this question.-Variable type:binary
binary.22612_2.txt	1710174270056F5 forCTG.txt.gz	0.1036	0.07937	1.305	0.1918	0.02368	0.007409	0.983	0.009658	0.02189	0.007378	Worked with materials containing asbestos: Often	FALSE				76457	1262	75195	UK Biobank	https://docs.google.com/spreadsheets/d/1kvPoupSzsSFBNSztMzl04xMoSC3Kcx3CrjVf4yBmESU/edit?ts=5b5f17db#gid=227859291	PHESANT Transformation:22612_0 | | CAT-MUL-BINARY-VAR 2 | | NO_NAN Remove NA participants 270383 | | Removed 14356 examples != 2 but with missing value (<0) | | sample 75195/1262(76457) | |-Notes:Participant asked Thinking about the place where you worked: Did you work with materials containing asbestos? Participants were allowed to skip answering this question.-Variable type:binary
binary.22613_0.txt	1710174270056F5 forCTG.txt.gz	-0.4453	0.1313	-3.392	0.000694	0.0168	0.006431	0.999	0.009828	0.004056	0.008467	Worked with paints, thinners or glues: Rarely/never	FALSE				90389	86796	3593	UK Biobank	https://docs.google.com/spreadsheets/d/1kvPoupSzsSFBNSztMzl04xMoSC3Kcx3CrjVf4yBmESU/edit?ts=5b5f17db#gid=227859291	PHESANT Transformation:22613_0 | | CAT-MUL-BINARY-VAR 0 | | NO_NAN Remove NA participants 270332 | | Removed 473 examples != 0 but with missing value (<0) | | sample 3593/86796(90389) | |-Notes:Participant asked Thinking about the place where you worked: Did you work with paints, thinners or glues? Participants were allowed to skip answering this question.-Variable type:binary
binary.22613_1.txt	1710174270056F5 forCTG.txt.gz	0.588	0.08224	7.15	8.67E-13	0.03071	0.006454	1.001	0.009093	0.008445	0.007771	Worked with paints, thinners or glues: Sometimes	FALSE	Occupation			89003	14095	74908	UK Biobank	https://docs.google.com/spreadsheets/d/1kvPoupSzsSFBNSztMzl04xMoSC3Kcx3CrjVf4yBmESU/edit?ts=5b5f17db#gid=227859291	PHESANT Transformation:22613_0 | | CAT-MUL-BINARY-VAR 1 | | NO_NAN Remove NA participants 270332 | | Removed 1859 examples != 1 but with missing value (<0) | | sample 74908/14095(89003) | | SKIP_val:-121 < 0 | |-Notes:Participant asked Thinking about the place where you worked: Did you work with paints, thinners or glues? Participants were allowed to skip answering this question.-Variable type:binary
binary.22613_2.txt	1710174270056F5 forCTG.txt.gz	0.6856	0.3117	2.199	0.02786	0.007071	0.006067	1.014	0.009447	0.00132	0.008009	Worked with paints, thinners or glues: Often	FALSE				88718	3723	84995	UK Biobank	https://docs.google.com/spreadsheets/d/1kvPoupSzsSFBNSztMzl04xMoSC3Kcx3CrjVf4yBmESU/edit?ts=5b5f17db#gid=227859291	PHESANT Transformation:22613_0 | | CAT-MUL-BINARY-VAR 2 | | NO_NAN Remove NA participants 270332 | | Removed 2145 examples != 2 but with missing value (<0) | | sample 84995/3723(88718) | |-Notes:Participant asked Thinking about the place where you worked: Did you work with paints, thinners or glues? Participants were allowed to skip answering this question.-Variable type:binary
binary.22615_1.txt	1710174270056F5 forCTG.txt.gz	0.4315	0.06811	6.336	2.36E-10	0.03006	0.007296	0.998	0.0103	0.008905	0.008028	Workplace had a lot of diesel exhaust: Sometimes	FALSE	Occupation			89310	13038	76272	UK Biobank	https://docs.google.com/spreadsheets/d/1kvPoupSzsSFBNSztMzl04xMoSC3Kcx3CrjVf4yBmESU/edit?ts=5b5f17db#gid=227859291	PHESANT Transformation:22615_0 | | CAT-MUL-BINARY-VAR 1 | | NO_NAN Remove NA participants 270410 | | Removed 1474 examples != 1 but with missing value (<0) | | sample 76272/13038(89310) | | SKIP_val:-121 < 0 | |-Notes:Participant asked Thinking about the place where you worked: Was there a lot of diesel exhaust? Participants were allowed to skip answering this question.-Variable type:binary
binary.22615_2.txt	1710174270056F5 forCTG.txt.gz	0.5859	0.1536	3.814	0.000137	0.01583	0.006651	1.006	0.009983	0.005731	0.007594	Workplace had a lot of diesel exhaust: Often	FALSE				89104	3483	85621	UK Biobank	https://docs.google.com/spreadsheets/d/1kvPoupSzsSFBNSztMzl04xMoSC3Kcx3CrjVf4yBmESU/edit?ts=5b5f17db#gid=227859291	PHESANT Transformation:22615_0 | | CAT-MUL-BINARY-VAR 2 | | NO_NAN Remove NA participants 270410 | | Removed 1680 examples != 2 but with missing value (<0) | | sample 85621/3483(89104) | |-Notes:Participant asked Thinking about the place where you worked: Was there a lot of diesel exhaust? Participants were allowed to skip answering this question.-Variable type:binary
binary.22616_0.txt	1710174270056F5 forCTG.txt.gz	-0.051	0.0869	-0.5869	0.5573	0.01738	0.00607	0.996	0.009308	-0.02169	0.007866	Breathing problems during period of job: No	FALSE				91149	89591	1558	UK Biobank	https://docs.google.com/spreadsheets/d/1kvPoupSzsSFBNSztMzl04xMoSC3Kcx3CrjVf4yBmESU/edit?ts=5b5f17db#gid=227859291	PHESANT Transformation:22616_0 | | CAT-MUL-BINARY-VAR 0 | | NO_NAN Remove NA participants 270045 | | Removed 0 examples != 0 but with missing value (<0) | | sample 1558/89591(91149) | |-Notes:Particpant asked: When you had this job, did you have problems with your breathing?-Variable type:binary
binary.22616_1.txt	1710174270056F5 forCTG.txt.gz	0.3623	0.08221	4.407	1.05E-05	0.02839	0.006032	1.012	0.00867	0.01413	0.008457	Breathing problems during period of job: Yes	TRUE	Occupation	Physical health	Breathing problems while working	91149	6697	84452	UK Biobank	https://docs.google.com/spreadsheets/d/1kvPoupSzsSFBNSztMzl04xMoSC3Kcx3CrjVf4yBmESU/edit?ts=5b5f17db#gid=227859291	PHESANT Transformation:22616_0 | | CAT-MUL-BINARY-VAR 1 | | NO_NAN Remove NA participants 270045 | | Removed 0 examples != 1 but with missing value (<0) | | sample 84452/6697(91149) | |-Notes:Particpant asked: When you had this job, did you have problems with your breathing?-Variable type:binary

<table>
<tr><td>binary.22617_1222.txt</td><td>1710174270056F5forCTG.txt.gz</td><td>0.07063</td><td>0.09364</td><td>0.7542</td><td>0.4507</td><td>0.01813</td><td>0.006388</td><td>0.988</td><td>0.01153</td><td>-0.00243</td><td>0.009648</td><td>Job SOC coding: Conference and exhibition managers</td><td>FALSE</td><td></td><td></td><td></td><td>91149</td><td>104</td><td>91045</td><td>UK Biobank</td><td>https://docs.google.com/spreadsheets/d/1kvPoupSzsSFBNSztMzl04xMoSC3Kcx3CrjVf4yBmESU/edit?ts=5b5f17db#gid=227859291</td><td>PHESANT Transformation:22617_0 | | CAT-MUL-BINARY-VAR 1222 | | NO_NAN Remove NA participants 270045 | | Removed 0 examples != 1222 but with missing value (<0) | | sample 91045/104(91149) | |-Notes:Each self-coded job group was mapped to a 4-digit SOC2003 coding. In some cases several self-coded job groups map to the same SOC2003 coding as the input interface was designed to help untrained users classify their job according to the criteria/paths that would seem most natural to them. It was not thought practical to ask participants to code their job to the more detailed levels of the SOC2003 coding.-Variable type:binary</td></tr>
<tr><td>binary.22617_2111.txt</td><td>1710174270056F5forCTG.txt.gz</td><td>-0.1463</td><td>0.08907</td><td>-1.643</td><td>0.1004</td><td>0.015</td><td>0.006166</td><td>0.995</td><td>0.009703</td><td>0.003613</td><td>0.007827</td><td>Job SOC coding: Chemists</td><td>FALSE</td><td></td><td></td><td></td><td>91149</td><td>1080</td><td>90069</td><td>UK Biobank</td><td>https://docs.google.com/spreadsheets/d/1kvPoupSzsSFBNSztMzl04xMoSC3Kcx3CrjVf4yBmESU/edit?ts=5b5f17db#gid=227859291</td><td>PHESANT Transformation:22617_0 | | CAT-MUL-BINARY-VAR 2111 | | NO_NAN Remove NA participants 270045 | | Removed 0 examples != 2111 but with missing value (<0) | | sample 90069/1080(91149) | |-Notes:Each self-coded job group was mapped to a 4-digit SOC2003 coding. In some cases several self-coded job groups map to the same SOC2003 coding as the input interface was designed to help untrained users classify their job according to the criteria/paths that would seem most natural to them. It was not thought practical to ask participants to code their job to the more detailed levels of the SOC2003 coding.-Variable type:binary</td></tr>
<tr><td>binary.22617_2112.txt</td><td>1710174270056F5forCTG.txt.gz</td><td>0.007672</td><td>0.09383</td><td>0.08177</td><td>0.9348</td><td>0.01594</td><td>0.00673</td><td>0.99</td><td>0.0105</td><td>-0.004368</td><td>0.007964</td><td>Job SOC coding: Biological scientists and biochemists</td><td>FALSE</td><td></td><td></td><td></td><td>91149</td><td>1067</td><td>90082</td><td>UK Biobank</td><td>https://docs.google.com/spreadsheets/d/1kvPoupSzsSFBNSztMzl04xMoSC3Kcx3CrjVf4yBmESU/edit?ts=5b5f17db#gid=227859291</td><td>PHESANT Transformation:22617_0 | | CAT-MUL-BINARY-VAR 2112 | | NO_NAN Remove NA participants 270045 | | Removed 0 examples != 2112 but with missing value (<0) | | sample 90082/1067(91149) | |-Notes:Each self-coded job group was mapped to a 4-digit SOC2003 coding. In some cases several self-coded job groups map to the same SOC2003 coding as the input interface was designed to help untrained users classify their job according to the criteria/paths that would seem most natural to them. It was not thought practical to ask participants to code their job to the more detailed levels of the SOC2003 coding.-Variable type:binary</td></tr>
<tr><td>binary.22617_2113.txt</td><td>1710174270056F5forCTG.txt.gz</td><td>-0.04446</td><td>0.09392</td><td>-0.4734</td><td>0.6359</td><td>0.01734</td><td>0.006256</td><td>0.989</td><td>0.008914</td><td>-0.009422</td><td>0.007944</td><td>Job SOC coding: Physicists, geologists and meteorologists</td><td>FALSE</td><td></td><td></td><td></td><td>91149</td><td>733</td><td>90416</td><td>UK Biobank</td><td>https://docs.google.com/spreadsheets/d/1kvPoupSzsSFBNSztMzl04xMoSC3Kcx3CrjVf4yBmESU/edit?ts=5b5f17db#gid=227859291</td><td>PHESANT Transformation:22617_0 | | CAT-MUL-BINARY-VAR 2113 | | NO_NAN Remove NA participants 270045 | | Removed 0 examples != 2113 but with missing value (<0) | | sample 90416/733(91149) | |-Notes:Each self-coded job group was mapped to a 4-digit SOC2003 coding. In some cases several self-coded job groups map to the same SOC2003 coding as the input interface was designed to help untrained users classify their job according to the criteria/paths that would seem most natural to them. It was not thought practical to ask participants to code their job to the more detailed levels of the SOC2003 coding.-Variable type:binary</td></tr>
<tr><td>binary.22617_2132.txt</td><td>1710174270056F5forCTG.txt.gz</td><td>-0.224</td><td>0.1399</td><td>-1.601</td><td>0.1094</td><td>0.01018</td><td>0.006637</td><td>1.022</td><td>0.0101</td><td>-0.007917</td><td>0.007966</td><td>Job SOC coding: Software professionals</td><td>FALSE</td><td></td><td></td><td></td><td>91149</td><td>3519</td><td>87630</td><td>UK Biobank</td><td>https://docs.google.com/spreadsheets/d/1kvPoupSzsSFBNSztMzl04xMoSC3Kcx3CrjVf4yBmESU/edit?ts=5b5f17db#gid=227859291</td><td>PHESANT Transformation:22617_0 | | CAT-MUL-BINARY-VAR 2132 | | NO_NAN Remove NA participants 270045 | | Removed 0 examples != 2132 but with missing value (<0) | | sample 87630/3519(91149) | |-Notes:Each self-coded job group was mapped to a 4-digit SOC2003 coding. In some cases several self-coded job groups map to the same SOC2003 coding as the input interface was designed to help untrained users classify their job according to the criteria/paths that would seem most natural to them. It was not thought practical to ask participants to code their job to the more detailed levels of the SOC2003 coding.-Variable type:binary</td></tr>
<tr><td>binary.22617_2211.txt</td><td>1710174270056F5forCTG.txt.gz</td><td>-0.1942</td><td>0.08231</td><td>-2.359</td><td>0.01832</td><td>0.0224</td><td>0.005869</td><td>1.007</td><td>0.008465</td><td>-0.01106</td><td>0.008386</td><td>Job SOC coding: Medical practitioners</td><td>FALSE</td><td></td><td></td><td></td><td>91149</td><td>1500</td><td>89649</td><td>UK Biobank</td><td>https://docs.google.com/spreadsheets/d/1kvPoupSzsSFBNSztMzl04xMoSC3Kcx3CrjVf4yBmESU/edit?ts=5b5f17db#gid=227859291</td><td>PHESANT Transformation:22617_0 | | CAT-MUL-BINARY-VAR 2211 | | NO_NAN Remove NA participants 270045 | | Removed 0 examples != 2211 but with missing value (<0) | | sample 89649/1500(91149) | |-Notes:Each self-coded job group was mapped to a 4-digit SOC2003 coding. In some cases several self-coded job groups map to the same SOC2003 coding as the input interface was designed to help untrained users classify their job according to the criteria/paths that would seem most natural to them. It was not thought practical to ask participants to code their job to the more detailed levels of the SOC2003 coding.-Variable type:binary</td></tr>
<tr><td>binary.22617_2311.txt</td><td>1710174270056F5forCTG.txt.gz</td><td>-0.1216</td><td>0.07559</td><td>-1.608</td><td>0.1078</td><td>0.02401</td><td>0.006177</td><td>0.993</td><td>0.008973</td><td>-0.002284</td><td>0.00796</td><td>Job SOC coding: Higher education teaching professionals</td><td>FALSE</td><td></td><td></td><td></td><td>91149</td><td>3100</td><td>88049</td><td>UK Biobank</td><td>https://docs.google.com/spreadsheets/d/1kvPoupSzsSFBNSztMzl04xMoSC3Kcx3CrjVf4yBmESU/edit?ts=5b5f17db#gid=227859291</td><td>PHESANT Transformation:22617_0 | | CAT-MUL-BINARY-VAR 2311 | | NO_NAN Remove NA participants 270045 | | Removed 0 examples != 2311 but with missing value (<0) | | sample 88049/3100(91149) | |-Notes:Each self-coded job group was mapped to a 4-digit SOC2003 coding. In some cases several self-coded job groups map to the same SOC2003 coding as the input interface was designed to help untrained users classify their job according to the criteria/paths that would seem most natural to them. It was not thought practical to ask participants to code their job to the more detailed levels of the SOC2003 coding.-Variable type:binary</td></tr>
<tr><td>binary.22617_2314.txt</td><td>1710174270056F5forCTG.txt.gz</td><td>-0.197</td><td>0.07862</td><td>-2.506</td><td>0.01222</td><td>0.02398</td><td>0.005684</td><td>1</td><td>0.0093</td><td>-0.01576</td><td>0.007927</td><td>Job SOC coding: Secondary education teaching professionals</td><td>FALSE</td><td></td><td></td><td></td><td>91149</td><td>7682</td><td>83467</td><td>UK Biobank</td><td>https://docs.google.com/spreadsheets/d/1kvPoupSzsSFBNSztMzl04xMoSC3Kcx3CrjVf4yBmESU/edit?ts=5b5f17db#gid=227859291</td><td>PHESANT Transformation:22617_0 | | CAT-MUL-BINARY-VAR 2314 | | NO_NAN Remove NA participants 270045 | | Removed 0 examples != 2314 but with missing value (<0) | | sample 83467/7682(91149) | |-Notes:Each self-coded job group was mapped to a 4-digit SOC2003 coding. In some cases several self-coded job groups map to the same SOC2003 coding as the input interface was designed to help untrained users classify their job according to the criteria/paths that would seem most natural to them. It was not thought practical to ask participants to code their job to the more detailed levels of the SOC2003 coding.-Variable type:binary</td></tr>
<tr><td>binary.22617_2315.txt</td><td>1710174270056F5forCTG.txt.gz</td><td>-0.205</td><td>0.08844</td><td>-2.318</td><td>0.02045</td><td>0.0217</td><td>0.006294</td><td>0.986</td><td>0.009906</td><td>-0.000649</td><td>0.008968</td><td>Job SOC coding: Primary and nursery education teaching professionals</td><td>FALSE</td><td></td><td></td><td></td><td>91149</td><td>5625</td><td>85524</td><td>UK Biobank</td><td>https://docs.google.com/spreadsheets/d/1kvPoupSzsSFBNSztMzl04xMoSC3Kcx3CrjVf4yBmESU/edit?ts=5b5f17db#gid=227859291</td><td>PHESANT Transformation:22617_0 | | CAT-MUL-BINARY-VAR 2315 | | NO_NAN Remove NA participants 270045 | | Removed 0 examples != 2315 but with missing value (<0) | | sample 85524/5625(91149) | |-Notes:Each self-coded job group was mapped to a 4-digit SOC2003 coding. In some cases several self-coded job groups map to the same SOC2003 coding as the input interface was designed to help untrained users classify their job according to the criteria/paths that would seem most natural to them. It was not thought practical to ask participants to code their job to the more detailed levels of the SOC2003 coding.-Variable type:binary</td></tr>
<tr><td>binary.22617_2321.txt</td><td>1710174270056F5forCTG.txt.gz</td><td>-0.03703</td><td>0.08735</td><td>-0.424</td><td>0.6716</td><td>0.01343</td><td>0.00596</td><td>1</td><td>0.009178</td><td>-0.000651</td><td>0.006836</td><td>Job SOC coding: Scientific researchers</td><td>FALSE</td><td></td><td></td><td></td><td>91149</td><td>1190</td><td>89959</td><td>UK Biobank</td><td>https://docs.google.com/spreadsheets/d/1kvPoupSzsSFBNSztMzl04xMoSC3Kcx3CrjVf4yBmESU/edit?ts=5b5f17db#gid=227859291</td><td>PHESANT Transformation:22617_0 | | CAT-MUL-BINARY-VAR 2321 | | NO_NAN Remove NA participants 270045 | | Removed 0 examples != 2321 but with missing value (<0) | | sample 89959/1190(91149) | |-Notes:Each self-coded job group was mapped to a 4-digit SOC2003 coding. In some cases several self-coded job groups map to the same SOC2003 coding as the input interface was designed to help untrained users classify their job according to the criteria/paths that would seem most natural to them. It was not thought practical to ask participants to code their job to the more detailed levels of the SOC2003 coding.-Variable type:binary</td></tr>
<tr><td>binary.22617_2329.txt</td><td>1710174270056F5forCTG.txt.gz</td><td>-0.08392</td><td>0.1167</td><td>-0.7189</td><td>0.4722</td><td>0.01051</td><td>0.005886</td><td>1.002</td><td>0.009636</td><td>-0.005102</td><td>0.008</td><td>Job SOC coding: Researchers n.e.c.</td><td>FALSE</td><td></td><td></td><td></td><td>91149</td><td>1028</td><td>90121</td><td>UK Biobank</td><td>https://docs.google.com/spreadsheets/d/1kvPoupSzsSFBNSztMzl04xMoSC3Kcx3CrjVf4yBmESU/edit?ts=5b5f17db#gid=227859291</td><td>PHESANT Transformation:22617_0 | | CAT-MUL-BINARY-VAR 2329 | | NO_NAN Remove NA participants 270045 | | Removed 0 examples != 2329 but with missing value (<0) | | sample 90121/1028(91149) | |-Notes:Each self-coded job group was mapped to a 4-digit SOC2003 coding. In some cases several self-coded job groups map to the same SOC2003 coding as the input interface was designed to help untrained users classify their job according to the criteria/paths that would seem most natural to them. It was not thought practical to ask participants to code their job to the more detailed levels of the SOC2003 coding.-Variable type:binary</td></tr>
<tr><td>binary.22617_2432.txt</td><td>1710174270056F5forCTG.txt.gz</td><td>-0.06277</td><td>0.09622</td><td>-0.6523</td><td>0.5142</td><td>0.01346</td><td>0.006532</td><td>0.986</td><td>0.01085</td><td>0.0006007</td><td>0.007973</td><td>Job SOC coding: Town planners</td><td>FALSE</td><td></td><td></td><td></td><td>91149</td><td>221</td><td>90928</td><td>UK Biobank</td><td>https://docs.google.com/spreadsheets/d/1kvPoupSzsSFBNSztMzl04xMoSC3Kcx3CrjVf4yBmESU/edit?ts=5b5f17db#gid=227859291</td><td>PHESANT Transformation:22617_0 | | CAT-MUL-BINARY-VAR 2432 | | NO_NAN Remove NA participants 270045 | | Removed 0 examples != 2432 but with missing value (<0) | | sample 90928/221(91149) | |-Notes:Each self-coded job group was mapped to a 4-digit SOC2003 coding. In some cases several self-coded job groups map to the same SOC2003 coding as the input interface was designed to help untrained users classify their job according to the criteria/paths that would seem most natural to them. It was not thought practical to ask participants to code their job to the more detailed levels of the SOC2003 coding.-Variable type:binary</td></tr>
<tr><td>binary.22617_2441.txt</td><td>1710174270056F5forCTG.txt.gz</td><td>-0.2779</td><td>0.1572</td><td>-1.768</td><td>0.07706</td><td>0.007604</td><td>0.005904</td><td>1.009</td><td>0.009562</td><td>0.00517</td><td>0.007115</td><td>Job SOC coding: Public service administrative professionals</td><td>FALSE</td><td></td><td></td><td></td><td>91149</td><td>1352</td><td>89797</td><td>UK Biobank</td><td>https://docs.google.com/spreadsheets/d/1kvPoupSzsSFBNSztMzl04xMoSC3Kcx3CrjVf4yBmESU/edit?ts=5b5f17db#gid=227859291</td><td>PHESANT Transformation:22617_0 | | CAT-MUL-BINARY-VAR 2441 | | NO_NAN Remove NA participants 270045 | | Removed 0 examples != 2441 but with missing value (<0) | | sample 89797/1352(91149) | |-Notes:Each self-coded job group was mapped to a 4-digit SOC2003 coding. In some cases several self-coded job groups map to the same SOC2003 coding as the input interface was designed to help untrained users classify their job according to the criteria/paths that would seem most natural to them. It was not thought practical to ask participants to code their job to the more detailed levels of the SOC2003 coding.-Variable type:binary</td></tr>
</table>

<table>
<tr><td>binary.22617_3211.txt</td><td>1710174270056F5forCTG.txt.gz</td><td>0.3336</td><td>0.1571</td><td>2.124</td><td>0.03367</td><td>0.009665</td><td>0.005682</td><td>1.009</td><td>0.009518</td><td>-0.004143</td><td>0.00783</td><td>Job SOC coding: Nurses</td><td>FALSE</td><td></td><td></td><td></td><td>91149</td><td>5062</td><td>86087</td><td>UK Biobank</td><td>https://docs.google.com/spreadsheets/d/1kvPoupSzsSFBNSztMzl04xMoSC3Kcx3CrjVf4yBmESU/edit?ts=5b5f17db#gid=227859291</td><td>PHESANT Transformation:22617_0 | | CAT-MUL-BINARY-VAR 3211 | | NO_NAN Remove NA participants 270045 | | Removed 0 examples != 3211 but with missing value (<0) | | sample 86087/5062(91149) | |-Notes:Each self-coded job group was mapped to a 4-digit SOC2003 coding. In some cases several self-coded job groups map to the same SOC2003 coding as the input interface was designed to help untrained users classify their job according to the criteria/paths that would seem most natural to them. It was not thought practical to ask participants to code their job to the more detailed levels of the SOC2003 coding.-Variable type:binary</td></tr>
<tr><td>binary.22617_3222.txt</td><td>1710174270056F5forCTG.txt.gz</td><td>-0.1589</td><td>0.1128</td><td>-1.409</td><td>0.1589</td><td>0.01146</td><td>0.006288</td><td>0.994</td><td>0.00983</td><td>0.001121</td><td>0.008269</td><td>Job SOC coding: Occupational therapists</td><td>FALSE</td><td></td><td></td><td></td><td>91149</td><td>299</td><td>90850</td><td>UK Biobank</td><td>https://docs.google.com/spreadsheets/d/1kvPoupSzsSFBNSztMzl04xMoSC3Kcx3CrjVf4yBmESU/edit?ts=5b5f17db#gid=227859291</td><td>PHESANT Transformation:22617_0 | | CAT-MUL-BINARY-VAR 3222 | | NO_NAN Remove NA participants 270045 | | Removed 0 examples != 3222 but with missing value (<0) | | sample 90850/299(91149) | |-Notes:Each self-coded job group was mapped to a 4-digit SOC2003 coding. In some cases several self-coded job groups map to the same SOC2003 coding as the input interface was designed to help untrained users classify their job according to the criteria/paths that would seem most natural to them. It was not thought practical to ask participants to code their job to the more detailed levels of the SOC2003 coding.-Variable type:binary</td></tr>
<tr><td>binary.22617_3311.txt</td><td>1710174270056F5forCTG.txt.gz</td><td>0.5389</td><td>0.2139</td><td>2.52</td><td>0.01174</td><td>0.008092</td><td>0.006104</td><td>1.012</td><td>0.009516</td><td>0.007699</td><td>0.007572</td><td>Job SOC coding: NCOs and other ranks</td><td>FALSE</td><td></td><td></td><td></td><td>91149</td><td>2058</td><td>89091</td><td>UK Biobank</td><td>https://docs.google.com/spreadsheets/d/1kvPoupSzsSFBNSztMzl04xMoSC3Kcx3CrjVf4yBmESU/edit?ts=5b5f17db#gid=227859291</td><td>PHESANT Transformation:22617_0 | | CAT-MUL-BINARY-VAR 3311 | | NO_NAN Remove NA participants 270045 | | Removed 0 examples != 3311 but with missing value (<0) | | sample 89091/2058(91149) | |-Notes:Each self-coded job group was mapped to a 4-digit SOC2003 coding. In some cases several self-coded job groups map to the same SOC2003 coding as the input interface was designed to help untrained users classify their job according to the criteria/paths that would seem most natural to them. It was not thought practical to ask participants to code their job to the more detailed levels of the SOC2003 coding.-Variable type:binary</td></tr>
<tr><td>binary.22617_3412.txt</td><td>1710174270056F5forCTG.txt.gz</td><td>-0.02896</td><td>0.08762</td><td>-0.3306</td><td>0.741</td><td>0.01696</td><td>0.006043</td><td>0.976</td><td>0.009901</td><td>-0.003628</td><td>0.008049</td><td>Job SOC coding: Authors, writers</td><td>FALSE</td><td></td><td></td><td></td><td>91149</td><td>313</td><td>90836</td><td>UK Biobank</td><td>https://docs.google.com/spreadsheets/d/1kvPoupSzsSFBNSztMzl04xMoSC3Kcx3CrjVf4yBmESU/edit?ts=5b5f17db#gid=227859291</td><td>PHESANT Transformation:22617_0 | | CAT-MUL-BINARY-VAR 3412 | | NO_NAN Remove NA participants 270045 | | Removed 0 examples != 3412 but with missing value (<0) | | sample 90836/313(91149) | |-Notes:Each self-coded job group was mapped to a 4-digit SOC2003 coding. In some cases several self-coded job groups map to the same SOC2003 coding as the input interface was designed to help untrained users classify their job according to the criteria/paths that would seem most natural to them. It was not thought practical to ask participants to code their job to the more detailed levels of the SOC2003 coding.-Variable type:binary</td></tr>
<tr><td>binary.22617_3431.txt</td><td>1710174270056F5forCTG.txt.gz</td><td>-0.004348</td><td>0.104</td><td>-0.04182</td><td>0.9666</td><td>0.01353</td><td>0.005311</td><td>0.995</td><td>0.008618</td><td>-0.01133</td><td>0.007903</td><td>Job SOC coding: Journalists, newspaper and periodical editors</td><td>FALSE</td><td></td><td></td><td></td><td>91149</td><td>507</td><td>90642</td><td>UK Biobank</td><td>https://docs.google.com/spreadsheets/d/1kvPoupSzsSFBNSztMzl04xMoSC3Kcx3CrjVf4yBmESU/edit?ts=5b5f17db#gid=227859291</td><td>PHESANT Transformation:22617_0 | | CAT-MUL-BINARY-VAR 3431 | | NO_NAN Remove NA participants 270045 | | Removed 0 examples != 3431 but with missing value (<0) | | sample 90642/507(91149) | |-Notes:Each self-coded job group was mapped to a 4-digit SOC2003 coding. In some cases several self-coded job groups map to the same SOC2003 coding as the input interface was designed to help untrained users classify their job according to the criteria/paths that would seem most natural to them. It was not thought practical to ask participants to code their job to the more detailed levels of the SOC2003 coding.-Variable type:binary</td></tr>
<tr><td>binary.22617_3513.txt</td><td>1710174270056F5forCTG.txt.gz</td><td>0.01883</td><td>0.1257</td><td>0.1497</td><td>0.881</td><td>0.009502</td><td>0.005959</td><td>0.979</td><td>0.009546</td><td>0.006479</td><td>0.008464</td><td>Job SOC coding: Ship and hovercraft officers</td><td>FALSE</td><td></td><td></td><td></td><td>91149</td><td>342</td><td>90807</td><td>UK Biobank</td><td>https://docs.google.com/spreadsheets/d/1kvPoupSzsSFBNSztMzl04xMoSC3Kcx3CrjVf4yBmESU/edit?ts=5b5f17db#gid=227859291</td><td>PHESANT Transformation:22617_0 | | CAT-MUL-BINARY-VAR 3513 | | NO_NAN Remove NA participants 270045 | | Removed 0 examples != 3513 but with missing value (<0) | | sample 90807/342(91149) | |-Notes:Each self-coded job group was mapped to a 4-digit SOC2003 coding. In some cases several self-coded job groups map to the same SOC2003 coding as the input interface was designed to help untrained users classify their job according to the criteria/paths that would seem most natural to them. It was not thought practical to ask participants to code their job to the more detailed levels of the SOC2003 coding.-Variable type:binary</td></tr>
<tr><td>binary.22617_3539.txt</td><td>1710174270056F5forCTG.txt.gz</td><td>-0.107</td><td>0.1014</td><td>-1.055</td><td>0.2913</td><td>0.01078</td><td>0.006387</td><td>0.997</td><td>0.009624</td><td>-0.001867</td><td>0.007966</td><td>Job SOC coding: Business and related associate professionals n.e.c.</td><td>FALSE</td><td></td><td></td><td></td><td>91149</td><td>1980</td><td>89169</td><td>UK Biobank</td><td>https://docs.google.com/spreadsheets/d/1kvPoupSzsSFBNSztMzl04xMoSC3Kcx3CrjVf4yBmESU/edit?ts=5b5f17db#gid=227859291</td><td>PHESANT Transformation:22617_0 | | CAT-MUL-BINARY-VAR 3539 | | NO_NAN Remove NA participants 270045 | | Removed 0 examples != 3539 but with missing value (<0) | | sample 89169/1980(91149) | |-Notes:Each self-coded job group was mapped to a 4-digit SOC2003 coding. In some cases several self-coded job groups map to the same SOC2003 coding as the input interface was designed to help untrained users classify their job according to the criteria/paths that would seem most natural to them. It was not thought practical to ask participants to code their job to the more detailed levels of the SOC2003 coding.-Variable type:binary</td></tr>
<tr><td>binary.22617_4114.txt</td><td>1710174270056F5forCTG.txt.gz</td><td>0.005788</td><td>0.08784</td><td>0.06589</td><td>0.9475</td><td>0.0171</td><td>0.00621</td><td>0.983</td><td>0.00956</td><td>-0.02181</td><td>0.008189</td><td>Job SOC coding: Officers of non-governmental organisations</td><td>FALSE</td><td></td><td></td><td></td><td>91149</td><td>1579</td><td>89570</td><td>UK Biobank</td><td>https://docs.google.com/spreadsheets/d/1kvPoupSzsSFBNSztMzl04xMoSC3Kcx3CrjVf4yBmESU/edit?ts=5b5f17db#gid=227859291</td><td>PHESANT Transformation:22617_0 | | CAT-MUL-BINARY-VAR 4114 | | NO_NAN Remove NA participants 270045 | | Removed 0 examples != 4114 but with missing value (<0) | | sample 89570/1579(91149) | |-Notes:Each self-coded job group was mapped to a 4-digit SOC2003 coding. In some cases several self-coded job groups map to the same SOC2003 coding as the input interface was designed to help untrained users classify their job according to the criteria/paths that would seem most natural to them. It was not thought practical to ask participants to code their job to the more detailed levels of the SOC2003 coding.-Variable type:binary</td></tr>
<tr><td>binary.22617_4150.txt</td><td>1710174270056F5forCTG.txt.gz</td><td>-0.1437</td><td>0.1196</td><td>-1.202</td><td>0.2295</td><td>0.01161</td><td>0.006033</td><td>0.995</td><td>0.009945</td><td>0.001412</td><td>0.00846</td><td>Job SOC coding: General office assistants/clerks</td><td>FALSE</td><td></td><td></td><td></td><td>91149</td><td>4022</td><td>87127</td><td>UK Biobank</td><td>https://docs.google.com/spreadsheets/d/1kvPoupSzsSFBNSztMzl04xMoSC3Kcx3CrjVf4yBmESU/edit?ts=5b5f17db#gid=227859291</td><td>PHESANT Transformation:22617_0 | | CAT-MUL-BINARY-VAR 4150 | | NO_NAN Remove NA participants 270045 | | Removed 0 examples != 4150 but with missing value (<0) | | sample 87127/4022(91149) | |-Notes:Each self-coded job group was mapped to a 4-digit SOC2003 coding. In some cases several self-coded job groups map to the same SOC2003 coding as the input interface was designed to help untrained users classify their job according to the criteria/paths that would seem most natural to them. It was not thought practical to ask participants to code their job to the more detailed levels of the SOC2003 coding.-Variable type:binary</td></tr>
<tr><td>binary.22617_4215.txt</td><td>1710174270056F5forCTG.txt.gz</td><td>-0.1299</td><td>0.07572</td><td>-1.715</td><td>0.08629</td><td>0.02524</td><td>0.006171</td><td>0.978</td><td>0.009418</td><td>0.0007854</td><td>0.008228</td><td>Job SOC coding: Personal assistants and other secretaries</td><td>FALSE</td><td></td><td></td><td></td><td>91149</td><td>5418</td><td>85731</td><td>UK Biobank</td><td>https://docs.google.com/spreadsheets/d/1kvPoupSzsSFBNSztMzl04xMoSC3Kcx3CrjVf4yBmESU/edit?ts=5b5f17db#gid=227859291</td><td>PHESANT Transformation:22617_0 | | CAT-MUL-BINARY-VAR 4215 | | NO_NAN Remove NA participants 270045 | | Removed 0 examples != 4215 but with missing value (<0) | | sample 85731/5418(91149) | |-Notes:Each self-coded job group was mapped to a 4-digit SOC2003 coding. In some cases several self-coded job groups map to the same SOC2003 coding as the input interface was designed to help untrained users classify their job according to the criteria/paths that would seem most natural to them. It was not thought practical to ask participants to code their job to the more detailed levels of the SOC2003 coding.-Variable type:binary</td></tr>
<tr><td>binary.22617_4216.txt</td><td>1710174270056F5forCTG.txt.gz</td><td>0.2581</td><td>0.124</td><td>2.081</td><td>0.0374</td><td>0.0109</td><td>0.006297</td><td>0.996</td><td>0.009416</td><td>-0.004237</td><td>0.008075</td><td>Job SOC coding: Receptionists</td><td>FALSE</td><td></td><td></td><td></td><td>91149</td><td>1407</td><td>89742</td><td>UK Biobank</td><td>https://docs.google.com/spreadsheets/d/1kvPoupSzsSFBNSztMzl04xMoSC3Kcx3CrjVf4yBmESU/edit?ts=5b5f17db#gid=227859291</td><td>PHESANT Transformation:22617_0 | | CAT-MUL-BINARY-VAR 4216 | | NO_NAN Remove NA participants 270045 | | Removed 0 examples != 4216 but with missing value (<0) | | sample 89742/1407(91149) | |-Notes:Each self-coded job group was mapped to a 4-digit SOC2003 coding. In some cases several self-coded job groups map to the same SOC2003 coding as the input interface was designed to help untrained users classify their job according to the criteria/paths that would seem most natural to them. It was not thought practical to ask participants to code their job to the more detailed levels of the SOC2003 coding.-Variable type:binary</td></tr>
<tr><td>binary.22617_6114.txt</td><td>1710174270056F5forCTG.txt.gz</td><td>0.2448</td><td>0.1011</td><td>2.422</td><td>0.01545</td><td>0.01696</td><td>0.006589</td><td>0.995</td><td>0.008964</td><td>-0.01538</td><td>0.007884</td><td>Job SOC coding: Houseparents and residential wardens</td><td>FALSE</td><td></td><td></td><td></td><td>91149</td><td>646</td><td>90503</td><td>UK Biobank</td><td>https://docs.google.com/spreadsheets/d/1kvPoupSzsSFBNSztMzl04xMoSC3Kcx3CrjVf4yBmESU/edit?ts=5b5f17db#gid=227859291</td><td>PHESANT Transformation:22617_0 | | CAT-MUL-BINARY-VAR 6114 | | NO_NAN Remove NA participants 270045 | | Removed 0 examples != 6114 but with missing value (<0) | | sample 90503/646(91149) | |-Notes:Each self-coded job group was mapped to a 4-digit SOC2003 coding. In some cases several self-coded job groups map to the same SOC2003 coding as the input interface was designed to help untrained users classify their job according to the criteria/paths that would seem most natural to them. It was not thought practical to ask participants to code their job to the more detailed levels of the SOC2003 coding.-Variable type:binary</td></tr>
<tr><td>binary.22617_6115.txt</td><td>1710174270056F5forCTG.txt.gz</td><td>0.4195</td><td>0.144</td><td>2.913</td><td>0.003575</td><td>0.01112</td><td>0.005475</td><td>0.986</td><td>0.008643</td><td>-0.01079</td><td>0.008247</td><td>Job SOC coding: Care assistants and home carers</td><td>FALSE</td><td></td><td></td><td></td><td>91149</td><td>1172</td><td>89977</td><td>UK Biobank</td><td>https://docs.google.com/spreadsheets/d/1kvPoupSzsSFBNSztMzl04xMoSC3Kcx3CrjVf4yBmESU/edit?ts=5b5f17db#gid=227859291</td><td>PHESANT Transformation:22617_0 | | CAT-MUL-BINARY-VAR 6115 | | NO_NAN Remove NA participants 270045 | | Removed 0 examples != 6115 but with missing value (<0) | | sample 89977/1172(91149) | |-Notes:Each self-coded job group was mapped to a 4-digit SOC2003 coding. In some cases several self-coded job groups map to the same SOC2003 coding as the input interface was designed to help untrained users classify their job according to the criteria/paths that would seem most natural to them. It was not thought practical to ask participants to code their job to the more detailed levels of the SOC2003 coding.-Variable type:binary</td></tr>
</table>

binary.22617_8111.txt	1710174270056F5forCTG.txt.gz	0.03549	0.09795	0.3623	0.7171	0.01469	0.006221	0.987	0.01036	0.01806	0.008744	Job SOC coding: Food, drink and tobacco process operatives	FALSE				91149	247	90902	UK Biobank	https://docs.google.com/spreadsheets/d/1kvPoupSzsSFBNSztMzl04xMoSC3Kcx3CrjVf4yBmESU/edit?ts=5b5f17db#gid=227859291	PHESANT Transformation:22617_0 | | CAT-MUL-BINARY-VAR 8111 | | NO_NAN Remove NA participants 270045 | | Removed 0 examples != 8111 but with missing value (<0) | | sample 90902/247(91149) | |-Notes:Each self-coded job group was mapped to a 4-digit SOC2003 coding. In some cases several self-coded job groups map to the same SOC2003 coding as the input interface was designed to help untrained users classify their job according to the criteria/paths that would seem most natural to them. It was not thought practical to ask participants to code their job to the more detailed levels of the SOC2003 coding.-Variable type:binary
binary.22617_8211.txt	1710174270056F5forCTG.txt.gz	0.3109	0.1197	2.597	0.009404	0.01267	0.005859	0.991	0.009755	-0.00637	0.007781	Job SOC coding: Heavy goods vehicle drivers	FALSE				91149	701	90448	UK Biobank	https://docs.google.com/spreadsheets/d/1kvPoupSzsSFBNSztMzl04xMoSC3Kcx3CrjVf4yBmESU/edit?ts=5b5f17db#gid=227859291	PHESANT Transformation:22617_0 | | CAT-MUL-BINARY-VAR 8211 | | NO_NAN Remove NA participants 270045 | | Removed 0 examples != 8211 but with missing value (<0) | | sample 90448/701(91149) | |-Notes:Each self-coded job group was mapped to a 4-digit SOC2003 coding. In some cases several self-coded job groups map to the same SOC2003 coding as the input interface was designed to help untrained users classify their job according to the criteria/paths that would seem most natural to them. It was not thought practical to ask participants to code their job to the more detailed levels of the SOC2003 coding.-Variable type:binary
binary.22617_8215.txt	1710174270056F5forCTG.txt.gz	0.07289	0.1032	0.706	0.4802	0.0167	0.00642	0.978	0.01097	-0.00505	0.009878	Job SOC coding: Driving instructors	FALSE				91149	128	91021	UK Biobank	https://docs.google.com/spreadsheets/d/1kvPoupSzsSFBNSztMzl04xMoSC3Kcx3CrjVf4yBmESU/edit?ts=5b5f17db#gid=227859291	PHESANT Transformation:22617_0 | | CAT-MUL-BINARY-VAR 8215 | | NO_NAN Remove NA participants 270045 | | Removed 0 examples != 8215 but with missing value (<0) | | sample 91021/128(91149) | |-Notes:Each self-coded job group was mapped to a 4-digit SOC2003 coding. In some cases several self-coded job groups map to the same SOC2003 coding as the input interface was designed to help untrained users classify their job according to the criteria/paths that would seem most natural to them. It was not thought practical to ask participants to code their job to the more detailed levels of the SOC2003 coding.-Variable type:binary
binary.22617_9121.txt	1710174270056F5forCTG.txt.gz	0.1729	0.1135	1.524	0.1275	0.0107	0.005176	0.987	0.008842	-0.001759	0.007349	Job SOC coding: Labourers in building and woodworking trades	FALSE				91149	540	90609	UK Biobank	https://docs.google.com/spreadsheets/d/1kvPoupSzsSFBNSztMzl04xMoSC3Kcx3CrjVf4yBmESU/edit?ts=5b5f17db#gid=227859291	PHESANT Transformation:22617_0 | | CAT-MUL-BINARY-VAR 9121 | | NO_NAN Remove NA participants 270045 | | Removed 0 examples != 9121 but with missing value (<0) | | sample 90609/540(91149) | |-Notes:Each self-coded job group was mapped to a 4-digit SOC2003 coding. In some cases several self-coded job groups map to the same SOC2003 coding as the input interface was designed to help untrained users classify their job according to the criteria/paths that would seem most natural to them. It was not thought practical to ask participants to code their job to the more detailed levels of the SOC2003 coding.-Variable type:binary
binary.22617_9149.txt	1710174270056F5forCTG.txt.gz	0.2663	0.2226	1.196	0.2315	0.00501	0.005651	1	0.009796	0.009248	0.008125	Job SOC coding: Other goods handling and storage occupations n.e.c.	FALSE				91149	1279	89870	UK Biobank	https://docs.google.com/spreadsheets/d/1kvPoupSzsSFBNSztMzl04xMoSC3Kcx3CrjVf4yBmESU/edit?ts=5b5f17db#gid=227859291	PHESANT Transformation:22617_0 | | CAT-MUL-BINARY-VAR 9149 | | NO_NAN Remove NA participants 270045 | | Removed 0 examples != 9149 but with missing value (<0) | | sample 89870/1279(91149) | |-Notes:Each self-coded job group was mapped to a 4-digit SOC2003 coding. In some cases several self-coded job groups map to the same SOC2003 coding as the input interface was designed to help untrained users classify their job according to the criteria/paths that would seem most natural to them. It was not thought practical to ask participants to code their job to the more detailed levels of the SOC2003 coding.-Variable type:binary
binary.22617_9219.txt	1710174270056F5forCTG.txt.gz	0.3154	0.1637	1.927	0.05398	0.008452	0.006344	1.005	0.009396	0.004536	0.008169	Job SOC coding: Elementary office occupations n.e.c.	FALSE				91149	3340	87809	UK Biobank	https://docs.google.com/spreadsheets/d/1kvPoupSzsSFBNSztMzl04xMoSC3Kcx3CrjVf4yBmESU/edit?ts=5b5f17db#gid=227859291	PHESANT Transformation:22617_0 | | CAT-MUL-BINARY-VAR 9219 | | NO_NAN Remove NA participants 270045 | | Removed 0 examples != 9219 but with missing value (<0) | | sample 87809/3340(91149) | |-Notes:Each self-coded job group was mapped to a 4-digit SOC2003 coding. In some cases several self-coded job groups map to the same SOC2003 coding as the input interface was designed to help untrained users classify their job according to the criteria/paths that would seem most natural to them. It was not thought practical to ask participants to code their job to the more detailed levels of the SOC2003 coding.-Variable type:binary
binary.22618_0.txt	1710174270056F5forCTG.txt.gz	0.3097	0.1111	2.789	0.005292	0.01748	0.006232	1.009	0.009048	0.01155	0.008289	Breathing problems improved/stopped away from workplace or on holiday: No	FALSE				91149	4382	86767	UK Biobank	https://docs.google.com/spreadsheets/d/1kvPoupSzsSFBNSztMzl04xMoSC3Kcx3CrjVf4yBmESU/edit?ts=5b5f17db#gid=227859291	PHESANT Transformation:22618_0 | | CAT-MUL-BINARY-VAR 0 | | Indicator name x22616_0_0 | | Remove indicator var NAs: 270045 | | Remove indicator var <0: 0 | | Removed 0 examples != 0 but with missing value (<0) | | sample 86767/4382(91149) | |-Notes:Participants who indicated they had breathing problems during a job were asked: Did these problems improve or stop when you were away from the workplace or on holiday? Question only asked if participant has value Yes in ~F22616~-Variable type:binary
binary.22618_1.txt	1710174270056F5forCTG.txt.gz	0.4192	0.1294	3.24	0.001194	0.01335	0.005853	1.004	0.009207	0.01054	0.007829	Breathing problems improved/stopped away from workplace or on holiday: Yes	FALSE				91149	2688	88461	UK Biobank	https://docs.google.com/spreadsheets/d/1kvPoupSzsSFBNSztMzl04xMoSC3Kcx3CrjVf4yBmESU/edit?ts=5b5f17db#gid=227859291	PHESANT Transformation:22618_0 | | CAT-MUL-BINARY-VAR 1 | | Indicator name x22616_0_0 | | Remove indicator var NAs: 270045 | | Remove indicator var <0: 0 | | Removed 0 examples != 1 but with missing value (<0) | | sample 88461/2688(91149) | |-Notes:Participants who indicated they had breathing problems during a job were asked: Did these problems improve or stop when you were away from the workplace or on holiday? Question only asked if participant has value Yes in ~F22616~-Variable type:binary
binary.22619_0.txt	1710174270056F5forCTG.txt.gz	0.3021	0.0776	3.894	9.87E-05	0.03026	0.006222	1.006	0.009066	0.01854	0.008333	Breathing problems responsible for leaving job: No	FALSE				91149	5412	85737	UK Biobank	https://docs.google.com/spreadsheets/d/1kvPoupSzsSFBNSztMzl04xMoSC3Kcx3CrjVf4yBmESU/edit?ts=5b5f17db#gid=227859291	PHESANT Transformation:22619_0 | | CAT-MUL-BINARY-VAR 0 | | Indicator name x22616_0_0 | | Remove indicator var NAs: 270045 | | Remove indicator var <0: 0 | | Removed 0 examples != 0 but with missing value (<0) | | sample 85737/5412(91149) | |-Notes:Participants who indicated they had breathing problems during a job were asked: Did you leave this job because of breathing problems? Question only asked if participant has value Yes in ~F22616~-Variable type:binary
binary.22620_1.txt	1710174270056F5forCTG.txt.gz	0.5909	0.08411	7.025	2.15E-12	0.02802	0.006056	1.007	0.009488	0.0003679	0.008157	Job involved shift work: Yes	TRUE	Occupation	Occupation	Job involves shift work	91149	25698	65451	UK Biobank	https://docs.google.com/spreadsheets/d/1kvPoupSzsSFBNSztMzl04xMoSC3Kcx3CrjVf4yBmESU/edit?ts=5b5f17db#gid=227859291	PHESANT Transformation:22620_0 | | CAT-MUL-BINARY-VAR 1 | | NO_NAN Remove NA participants 270045 | | Removed 0 examples != 1 but with missing value (<0) | | sample 65451/25698(91149) | |-Notes:Participants were asked: Did you ever work shifts (day and/or night shifts) for this job? Day-shifts were defined as work in nomal daytime hours or morning, afternoon or evening work. Night-shifts were defined as work for at least 3 hours between midnight and 5am.-Variable type:binary
binary.22650_9.txt	1710174270056F5forCTG.txt.gz	-0.02952	0.104	-0.2839	0.7765	0.05372	0.02188	0.991	0.009679	-0.003088	0.008402	Night shifts worked: This type of shift pattern was not worked during job	FALSE				25697	24780	917	UK Biobank	https://docs.google.com/spreadsheets/d/1kvPoupSzsSFBNSztMzl04xMoSC3Kcx3CrjVf4yBmESU/edit?ts=5b5f17db#gid=227859291	PHESANT Transformation:22650_0 | | CAT-MUL-BINARY-VAR 9 | | NO_NAN Remove NA participants 335497 | | Removed 0 examples != 9 but with missing value (<0) | | sample 917/24780(25697) | |-Notes:Participants were asked: Which shift pattern(s) did you follow for this job? Participants who indicated they worked night shifts were then asked: Did you work night shifts for the whole of this job? Night-shifts were defined as work for at least 3 hours between midnight and 5am.-Variable type:binary
binary.22660_103.txt	1710174270056F5forCTG.txt.gz	0.02748	0.09409	0.2921	0.7702	0.0194	0.007563	1.006	0.008684	0.001473	0.007887	Gap coding: Full-time or part-time education	FALSE				68899	12489	56410	UK Biobank	https://docs.google.com/spreadsheets/d/1kvPoupSzsSFBNSztMzl04xMoSC3Kcx3CrjVf4yBmESU/edit?ts=5b5f17db#gid=227859291	PHESANT Transformation:22660_0 | | CAT-MUL-BINARY-VAR 103 | | NO_NAN Remove NA participants 288425 | | Removed 3874 examples != 103 but with missing value (<0) | | sample 56410/12489(68899) | |-Notes:Participants were asked what they did in any periods when they were not working in paid employment for at least 20 hours each week. These periods were called gaps.-Variable type:binary
binary.22660_105.txt	1710174270056F5forCTG.txt.gz	0.06454	0.1138	0.5671	0.5706	0.0167	0.007924	1.003	0.009345	-0.005692	0.008445	Gap coding: Looking after the home and/or family	FALSE				69370	24577	44793	UK Biobank	https://docs.google.com/spreadsheets/d/1kvPoupSzsSFBNSztMzl04xMoSC3Kcx3CrjVf4yBmESU/edit?ts=5b5f17db#gid=227859291	PHESANT Transformation:22660_0 | | CAT-MUL-BINARY-VAR 105 | | NO_NAN Remove NA participants 288425 | | Removed 3401 examples != 105 but with missing value (<0) | | sample 44793/24577(69370) | | SKIP_val:-717 < 0 | |-Notes:Participants were asked what they did in any periods when they were not working in paid employment for at least 20 hours each week. These periods were called gaps.-Variable type:binary
binary.22660_106.txt	1710174270056F5forCTG.txt.gz	0.2998	0.1149	2.61	0.00906	0.02025	0.008809	0.996	0.009362	0.01121	0.007601	Gap coding: Unable to work due to sickness or disability	FALSE				67892	2028	65864	UK Biobank	https://docs.google.com/spreadsheets/d/1kvPoupSzsSFBNSztMzl04xMoSC3Kcx3CrjVf4yBmESU/edit?ts=5b5f17db#gid=227859291	PHESANT Transformation:22660_0 | | CAT-MUL-BINARY-VAR 106 | | NO_NAN Remove NA participants 288425 | | Removed 4880 examples != 106 but with missing value (<0) | | sample 65864/2028(67892) | |-Notes:Participants were asked what they did in any periods when they were not working in paid employment for at least 20 hours each week. These periods were called gaps.-Variable type:binary
binary.2316.txt	1710174270056F5forCTG.txt.gz	0.3683	0.03444	10.69	1.08E-26	0.06015	0.003934	1.016	0.01613	0.02006	0.01007	Wheeze or whistling in the chest in last year	FALSE	Pulmonary			354523	73828	280695	UK Biobank	https://docs.google.com/spreadsheets/d/1kvPoupSzsSFBNSztMzl04xMoSC3Kcx3CrjVf4yBmESU/edit?ts=5b5f17db#gid=227859291	PHESANT Transformation:2316_0| | CAT-SINGLE | | Inc(>=10): 0(280695) | | Inc(>=10): 1(73828) | | CAT-SINGLE-BINARY | | sample 280695/73828(354523) | |-Notes:ACE touchscreen question In the last year have you ever had wheeze or whistling in the chest?-Variable type:binary
binary.2335.txt	1710174270056F5forCTG.txt.gz	0.3728	0.03888	9.59	8.85E-22	0.03341	0.002399	1.007	0.01189	0.01578	0.009087	Chest pain or discomfort	FALSE	Pain			357507	56233	301274	UK Biobank	https://docs.google.com/spreadsheets/d/1kvPoupSzsSFBNSztMzl04xMoSC3Kcx3CrjVf4yBmESU/edit?ts=5b5f17db#gid=227859291	PHESANT Transformation:2335_0| | CAT-SINGLE | | Inc(>=10): 0(301274) | | Inc(>=10): 1(56233) | | CAT-SINGLE-BINARY | | sample 301274/56233(357507) | |-Notes:ACE touchscreen question Do you ever have any pain or discomfort in your chest?-Variable type:binary

binary.2345.txt	1710174270056F5 forCTG.txt.gz	0.1498	0.0529	2.832	0.00463	0.01342	0.001872	1.015	0.01047	0.0004401	0.007496	Ever had bowel cancer screening	FALSE				355353	114475	240878	UK Biobank	https://docs.google.com/spreadsheets/d/1kvPoupSzsSFBNSztMzl04xMoSC3Kcx3CrjVf4yBmESU/edit?ts=5b5f17db#gid=227859291	PHESANT Transformation:2345_0 | | CAT-SINGLE | | Inc(>=10): 1(114475) | | Inc(>=10): 0(240878) | | CAT-SINGLE-BINARY | | sample 240878/114475(355353) | |-Notes:ACE touchscreen question Have you ever had a screening test for bowel (colorectal) cancer? (Please include tests for blood in the stool/faeces or a colonoscopy or a sigmoidoscopy) If the participant activated the Help button they were shown the message: Screening tests for bowel or colorectal cancer include: - FOBT (faecal occult blood test) - this is when you are given a set of cards and asked to smear a part of your stool on three separate occasions onto the cards and then return the cards to be tested for blood. - Sigmoidoscopy - a tube is used to examine the lower bowel. This is usually done in a doctor's office without pain relief. - Colonoscopy - a long tube is used the examine the whole large bowel; you would usually have to drink a large amount of special liquid to prepare the bowel, and you would be given a sedative medication for the procedure.-Variable type:binary
binary.2365.txt	1710174270056F5 forCTG.txt.gz	-0.07728	0.04971	-1.555	0.1201	0.03245	0.004103	0.996	0.01094	-0.005535	0.00809	Ever had prostate specific antigen (PSA) test	FALSE				158108	49459	108649	UK Biobank	https://docs.google.com/spreadsheets/d/1kvPoupSzsSFBNSztMzl04xMoSC3Kcx3CrjVf4yBmESU/edit?ts=5b5f17db#gid=227859291	PHESANT Transformation:2365_0 | | CAT-SINGLE | | Inc(>=10): 0(108649) | | Inc(>=10): 1(49459) | | CAT-SINGLE-BINARY | | sample 108649/49459(158108) | |-Notes:ACE touchscreen question Have you ever had a blood test for prostate cancer (prostate specific antigen or PSA test)? If the participant activated the Help button they were shown the message: If you are unsure, select Do not know.-Variable type:binary
binary.2395_1.txt	1710174270056F5 forCTG.txt.gz	0.002344	0.02994	0.07831	0.9376	0.1987	0.03514	1.118	0.06635	0.01778	0.009837	Hair/balding pattern: Pattern 1	FALSE				165649	53076	112573	UK Biobank	https://docs.google.com/spreadsheets/d/1kvPoupSzsSFBNSztMzl04xMoSC3Kcx3CrjVf4yBmESU/edit?ts=5b5f17db#gid=227859291	PHESANT Transformation:2395_0 | | CAT-SINGLE | | CAT-SINGLE-BINARY-VAR: 1 | | Inc(>=10): 1(53076) | |-Notes:ACE touchscreen question Which of the following best describes your hair/balding pattern? If the participant activated the Help button they were shown the message: If you are unsure, please provide an estimate or select Do not know. [Illustrations presented in this question have been adapted from Giles et al. 2002 Cancer Epidemiol Biomarkers Prev]-Variable type:binary
binary.2395_2.txt	1710174270056F5 forCTG.txt.gz	0.09222	0.041	2.25	0.02447	0.05492	0.007997	1.036	0.02045	-0.01954	0.008201	Hair/balding pattern: Pattern 2	FALSE				165649	38044	127605	UK Biobank	https://docs.google.com/spreadsheets/d/1kvPoupSzsSFBNSztMzl04xMoSC3Kcx3CrjVf4yBmESU/edit?ts=5b5f17db#gid=227859291	PHESANT Transformation:2395_0 | | CAT-SINGLE | | CAT-SINGLE-BINARY-VAR: 2 | | Inc(>=10): 2(38044) | |-Notes:ACE touchscreen question Which of the following best describes your hair/balding pattern? If the participant activated the Help button they were shown the message: If you are unsure, please provide an estimate or select Do not know. [Illustrations presented in this question have been adapted from Giles et al. 2002 Cancer Epidemiol Biomarkers Prev]-Variable type:binary
binary.2395_3.txt	1710174270056F5 forCTG.txt.gz	-0.123	0.04688	-2.623	0.008719	0.06521	0.02116	0.999	0.04176	0.009435	0.008415	Hair/balding pattern: Pattern 3	FALSE				165649	44304	121345	UK Biobank	https://docs.google.com/spreadsheets/d/1kvPoupSzsSFBNSztMzl04xMoSC3Kcx3CrjVf4yBmESU/edit?ts=5b5f17db#gid=227859291	PHESANT Transformation:2395_0 | | CAT-SINGLE | | CAT-SINGLE-BINARY-VAR: 3 | | Inc(>=10): 3(44304) | |-Notes:ACE touchscreen question Which of the following best describes your hair/balding pattern? If the participant activated the Help button they were shown the message: If you are unsure, please provide an estimate or select Do not know. [Illustrations presented in this question have been adapted from Giles et al. 2002 Cancer Epidemiol Biomarkers Prev]-Variable type:binary
binary.2395_4.txt	1710174270056F5 forCTG.txt.gz	0.0233	0.0282	0.8265	0.4085	0.1748	0.02657	1.111	0.05156	-0.009828	0.009444	Hair/balding pattern: Pattern 4	FALSE				165649	30225	135424	UK Biobank	https://docs.google.com/spreadsheets/d/1kvPoupSzsSFBNSztMzl04xMoSC3Kcx3CrjVf4yBmESU/edit?ts=5b5f17db#gid=227859291	PHESANT Transformation:2395_0 | | CAT-SINGLE | | CAT-SINGLE-BINARY-VAR: 4 | | Inc(>=10): 4(30225) | |-Notes:ACE touchscreen question Which of the following best describes your hair/balding pattern? If the participant activated the Help button they were shown the message: If you are unsure, please provide an estimate or select Do not know. [Illustrations presented in this question have been adapted from Giles et al. 2002 Cancer Epidemiol Biomarkers Prev]-Variable type:binary
binary.2415.txt	1710174270056F5 forCTG.txt.gz	0.1534	0.05764	2.662	0.007776	0.02308	0.004012	1.015	0.01026	0.01258	0.007615	Had major operations	FALSE				165737	106506	59231	UK Biobank	https://docs.google.com/spreadsheets/d/1kvPoupSzsSFBNSztMzl04xMoSC3Kcx3CrjVf4yBmESU/edit?ts=5b5f17db#gid=227859291	PHESANT Transformation:2415_0 | | CAT-SINGLE | | Inc(>=10): 1(106506) | | Inc(>=10): 0(59231) | | CAT-SINGLE-BINARY | | sample 59231/106506(165737) | |-Notes:ACE touchscreen question Have you had any major operations? (For example, operations that required an overnight stay in hospital) If the participant activated the Help button they were shown the message: If you are unsure if you have had a 'major' operation select Do not know and you will be asked about this by an interviewer later during this visit. ~F2415~ is male question equivalent of female question ~F2844~-Variable type:binary
binary.2443.txt	1710174270056F5 forCTG.txt.gz	0.2153	0.0395	5.45	5.04E-08	0.04042	0.003055	1.071	0.0156	0.009962	0.009807	Diabetes diagnosed by doctor	FALSE	Metabolic			360192	17275	342917	UK Biobank	https://docs.google.com/spreadsheets/d/1kvPoupSzsSFBNSztMzl04xMoSC3Kcx3CrjVf4yBmESU/edit?ts=5b5f17db#gid=227859291	PHESANT Transformation:2443_0 | | CAT-SINGLE | | Inc(>=10): 0(342917) | | Inc(>=10): 1(17275) | | CAT-SINGLE-BINARY | | sample 342917/17275(360192) | |-Notes:ACE touchscreen question Has a doctor ever told you that you have diabetes? If the participant activated the Help button they were shown the message: If you are unsure if you have been told you had diabetes, select Do not know and you will be asked about this by an interviewer later during this visit.-Variable type:binary
binary.2453.txt	1710174270056F5 forCTG.txt.gz	0.1409	0.06874	2.05	0.04033	0.007075	0.00173	1.011	0.01024	0.000221	0.007401	Cancer diagnosed by doctor	FALSE				359981	28509	331472	UK Biobank	https://docs.google.com/spreadsheets/d/1kvPoupSzsSFBNSztMzl04xMoSC3Kcx3CrjVf4yBmESU/edit?ts=5b5f17db#gid=227859291	PHESANT Transformation:2453_0 | | CAT-SINGLE | | Inc(>=10): 1(28509) | | Inc(>=10): 0(331472) | | CAT-SINGLE-BINARY | | sample 331472/28509(359981) | |-Notes:ACE touchscreen question Has a doctor ever told you that you have had cancer? If the participant activated the Help button they were shown the message: If you are unsure if you have been told you had cancer, select Do not know and you will be asked about this by an interviewer later during this visit.-Variable type:binary
binary.2463.txt	1710174270056F5 forCTG.txt.gz	0.1537	0.04742	3.241	0.00119	0.0185	0.002435	1.007	0.0114	0.02848	0.008326	Fractured/broken bones in last 5 years	FALSE				359241	34780	324461	UK Biobank	https://docs.google.com/spreadsheets/d/1kvPoupSzsSFBNSztMzl04xMoSC3Kcx3CrjVf4yBmESU/edit?ts=5b5f17db#gid=227859291	PHESANT Transformation:2463_0 | | CAT-SINGLE | | Inc(>=10): 0(324461) | | Inc(>=10): 1(34780) | | CAT-SINGLE-BINARY | | sample 324461/34780(359241) | |-Notes:ACE touchscreen question Have you fractured/broken any bones in the last 5 years?-Variable type:binary
binary.2473.txt	1710174270056F5 forCTG.txt.gz	0.2786	0.04173	6.676	2.46E-11	0.02577	0.002058	1.011	0.01109	0.01646	0.008725	Other serious medical condition/disability diagnosed by doctor	FALSE	Wellbeing			354588	72965	281623	UK Biobank	https://docs.google.com/spreadsheets/d/1kvPoupSzsSFBNSztMzl04xMoSC3Kcx3CrjVf4yBmESU/edit?ts=5b5f17db#gid=227859291	PHESANT Transformation:2473_0 | | CAT-SINGLE | | Inc(>=10): 0(281623) | | Inc(>=10): 1(72965) | | CAT-SINGLE-BINARY | | sample 281623/72965(354588) | |-Notes:ACE touchscreen question Has a doctor ever told you that you have had any other serious medical conditions or disabilities? If the participant activated the Help button they were shown the message: If you are not sure if you have a serious illness or you are not sure that you have had one of the conditions listed previously, enter Do not know and you will be asked about this by an interviewer later in this visit.-Variable type:binary
binary.2492.txt	1710174270056F5 forCTG.txt.gz	0.3353	0.03515	9.539	1.45E-21	0.0446	0.002645	1.02	0.01246	0.02117	0.008978	Taking other prescription medications	FALSE	Other (physical health)			360027	168993	191034	UK Biobank	https://docs.google.com/spreadsheets/d/1kvPoupSzsSFBNSztMzl04xMoSC3Kcx3CrjVf4yBmESU/edit?ts=5b5f17db#gid=227859291	PHESANT Transformation:2492_0 | | CAT-SINGLE | | Inc(>=10): 1(168993) | | Inc(>=10): 0(191034) | | CAT-SINGLE-BINARY | | sample 191034/168993(360027) | |-Notes:ACE touchscreen question Do you regularly take any other PRESCRIPTION medications? (Do not forget medications such as puffers or patches)-Variable type:binary
binary.2644.txt	1710174270056F5 forCTG.txt.gz	0.2585	0.05185	4.987	6.14E-07	0.0673	0.008124	1.017	0.01003	-0.000731	0.008382	Light smokers, at least 100 smokes in lifetime	FALSE	Lifestyle			97021	44253	52768	UK Biobank	https://docs.google.com/spreadsheets/d/1kvPoupSzsSFBNSztMzl04xMoSC3Kcx3CrjVf4yBmESU/edit?ts=5b5f17db#gid=227859291	PHESANT Transformation:2644_0 | | CAT-SINGLE | | Inc(>=10): 1(44253) | | Inc(>=10): 0(52768) | | CAT-SINGLE-BINARY | | sample 52768/44253(97021) | |-Notes:ACE touchscreen question In your lifetime, have you smoked a total of at least 100 times? ~F2644~ was collected from participants who indicated they smoked occasionally or Just tried once or twice, as defined by their answers to ~F1249~-Variable type:binary
binary.2654_2.txt	1710174270056F5 forCTG.txt.gz	0.07041	0.09189	0.7663	0.4435	0.008165	0.003457	1.027	0.01109	-0.009051	0.007696	Non-butter spread type details: Flora Pro-Active or Benecol	FALSE				190094	29048	161046	UK Biobank	https://docs.google.com/spreadsheets/d/1kvPoupSzsSFBNSztMzl04xMoSC3Kcx3CrjVf4yBmESU/edit?ts=5b5f17db#gid=227859291	PHESANT Transformation:2654_0 | | CAT-SINGLE | | CAT-SINGLE-BINARY-VAR: 2 | | Inc(>=10): 2(29048) | |-Notes:ACE touchscreen question What type of spread do you mainly use? If the participant activated the Help button they were shown the message: If you use more than one type of spread, please select the one that you use the most. If you are unsure, select Do not know. ~F2654~ was collected from participants who indicated they mainly use another type of spread/margarine rather than butter/spreadable butter or do not know what they use, as defined by their answers to ~F1428~-Variable type:binary
binary.2654_4.txt	1710174270056F5 forCTG.txt.gz	0.3266	0.06247	5.228	1.72E-07	0.02452	0.003461	1.023	0.01062	0.01419	0.008256	Non-butter spread type details: Soft (tub) margarine	FALSE	Dietary			190094	23625	166469	UK Biobank	https://docs.google.com/spreadsheets/d/1kvPoupSzsSFBNSztMzl04xMoSC3Kcx3CrjVf4yBmESU/edit?ts=5b5f17db#gid=227859291	PHESANT Transformation:2654_0 | | CAT-SINGLE | | CAT-SINGLE-BINARY-VAR: 4 | | Inc(>=10): 4(23625) | |-Notes:ACE touchscreen question What type of spread do you mainly use? If the participant activated the Help button they were shown the message: If you use more than one type of spread, please select the one that you use the most. If you are unsure, select Do not know. ~F2654~ was collected from participants who indicated they mainly use another type of spread/margarine rather than butter/spreadable butter or do not know what they use, as defined by their answers to ~F1428~-Variable type:binary
binary.2654_6.txt	1710174270056F5 forCTG.txt.gz	-0.1605	0.07152	-2.244	0.02486	0.01499	0.003171	1.002	0.01021	0.002447	0.007873	Non-butter spread type details: Olive oil based spread (eg: Bertolli)	FALSE				190094	47030	143064	UK Biobank	https://docs.google.com/spreadsheets/d/1kvPoupSzsSFBNSztMzl04xMoSC3Kcx3CrjVf4yBmESU/edit?ts=5b5f17db#gid=227859291	PHESANT Transformation:2654_0 | | CAT-SINGLE | | CAT-SINGLE-BINARY-VAR: 6 | | Inc(>=10): 6(47030) | |-Notes:ACE touchscreen question What type of spread do you mainly use? If the participant activated the Help button they were shown the message: If you use more than one type of spread, please select the one that you use the most. If you are unsure, select Do not know. ~F2654~ was collected from participants who indicated they mainly use another type of spread/margarine rather than butter/spreadable butter or do not know what they use, as defined by their answers to ~F1428~-Variable type:binary

binary.2654_7.txt	1710174270056F5forCTG.txt.gz	-0.488	0.154	-3.17	0.001524	0.00646	0.003056	1.002	0.009055	0.0002836	0.007188	Non-butter spread type details: Polyunsaturated/sunflower oil based spread (eg: Flora)	FALSE				190094	64325	125769	UK Biobank	https://docs.google.com/spreadsheets/d/1kvPoupSzsSFBNSztMzl04xMoSC3Kcx3CrjVf4yBmESU/edit?ts=5b5f17db#gid=227859291	PHESANT Transformation:2654_0 | | CAT-SINGLE | | CAT-SINGLE-BINARY-VAR: 7 | | Inc(>=10): 7(64325) | |-Notes:ACE touchscreen question What type of spread do you mainly use? If the participant activated the Help button they were shown the message: If you use more than one type of spread, please select the one that you use the most. If you are unsure, select Do not know. ~F2654~ was collected from participants who indicated they mainly use another type of spread/margarine rather than butter/spreadable butter or do not know what they use, as defined by their answers to ~F1428~-Variable type:binary
binary.2654_8.txt	1710174270056F5forCTG.txt.gz	0.1932	0.0889	2.173	0.02976	0.01073	0.003237	0.997	0.009632	-0.003266	0.008433	Non-butter spread type details: Other low or reduced fat spread	FALSE				190094	19035	171059	UK Biobank	https://docs.google.com/spreadsheets/d/1kvPoupSzsSFBNSztMzl04xMoSC3Kcx3CrjVf4yBmESU/edit?ts=5b5f17db#gid=227859291	PHESANT Transformation:2654_0 | | CAT-SINGLE | | CAT-SINGLE-BINARY-VAR: 8 | | Inc(>=10): 8(19035) | |-Notes:ACE touchscreen question What type of spread do you mainly use? If the participant activated the Help button they were shown the message: If you use more than one type of spread, please select the one that you use the most. If you are unsure, select Do not know. ~F2654~ was collected from participants who indicated they mainly use another type of spread/margarine rather than butter/spreadable butter or do not know what they use, as defined by their answers to ~F1428~-Variable type:binary
binary.2664_1.txt	1710174270056F5forCTG.txt.gz	0.4955	0.1293	3.833	0.000127	0.009411	0.00422	1.011	0.01015	-0.001414	0.007669	Reason for reducing amount of alcohol drunk: Illness or ill health	FALSE				134033	9512	124521	UK Biobank	https://docs.google.com/spreadsheets/d/1kvPoupSzsSFBNSztMzl04xMoSC3Kcx3CrjVf4yBmESU/edit?ts=5b5f17db#gid=227859291	PHESANT Transformation:2664_0 | | CAT-SINGLE | | CAT-SINGLE-BINARY-VAR: 1 | | Inc(>=10): 1(9512) | |-Notes:ACE touchscreen question Why did you reduce the amount you drank? ~F2664~ was collected from participants who indicated they drink alcohol, as defined by their answers to ~F1558~ and they drink less nowadays than 10 years ago, as defined by their answers to ~F1558~-Variable type:binary
binary.2664_3.txt	1710174270056F5forCTG.txt.gz	-0.1931	0.04863	-3.97	7.18E-05	0.04521	0.004804	0.999	0.01033	-0.01195	0.007862	Reason for reducing amount of alcohol drunk: Health precaution	FALSE				134033	43868	90165	UK Biobank	https://docs.google.com/spreadsheets/d/1kvPoupSzsSFBNSztMzl04xMoSC3Kcx3CrjVf4yBmESU/edit?ts=5b5f17db#gid=227859291	PHESANT Transformation:2664_0 | | CAT-SINGLE | | CAT-SINGLE-BINARY-VAR: 3 | | Inc(>=10): 3(43868) | |-Notes:ACE touchscreen question Why did you reduce the amount you drank? ~F2664~ was collected from participants who indicated they drink alcohol, as defined by their answers to ~F1558~ and they drink less nowadays than 10 years ago, as defined by their answers to ~F1558~-Variable type:binary
binary.2664_4.txt	1710174270056F5forCTG.txt.gz	0.3557	0.1332	2.67	0.007577	0.008268	0.004292	1.004	0.009573	0.002009	0.00806	Reason for reducing amount of alcohol drunk: Financial reasons	FALSE				134033	7054	126979	UK Biobank	https://docs.google.com/spreadsheets/d/1kvPoupSzsSFBNSztMzl04xMoSC3Kcx3CrjVf4yBmESU/edit?ts=5b5f17db#gid=227859291	PHESANT Transformation:2664_0 | | CAT-SINGLE | | CAT-SINGLE-BINARY-VAR: 4 | | Inc(>=10): 4(7054) | |-Notes:ACE touchscreen question Why did you reduce the amount you drank? ~F2664~ was collected from participants who indicated they drink alcohol, as defined by their answers to ~F1558~ and they drink less nowadays than 10 years ago, as defined by their answers to ~F1558~-Variable type:binary
binary.2664_5.txt	1710174270056F5forCTG.txt.gz	-0.02904	0.05067	-0.5731	0.5666	0.04272	0.004848	1.004	0.009603	0.01001	0.008608	Reason for reducing amount of alcohol drunk: Other reason	FALSE				134033	70609	63424	UK Biobank	https://docs.google.com/spreadsheets/d/1kvPoupSzsSFBNSztMzl04xMoSC3Kcx3CrjVf4yBmESU/edit?ts=5b5f17db#gid=227859291	PHESANT Transformation:2664_0 | | CAT-SINGLE | | CAT-SINGLE-BINARY-VAR: 5 | | Inc(>=10): 5(70609) | |-Notes:ACE touchscreen question Why did you reduce the amount you drank? ~F2664~ was collected from participants who indicated they drink alcohol, as defined by their answers to ~F1558~ and they drink less nowadays than 10 years ago, as defined by their answers to ~F1558~-Variable type:binary
binary.2724.txt	1710174270056F5forCTG.txt.gz	0.1532	0.04831	3.172	0.001514	0.04089	0.006837	0.997	0.01659	-0.004766	0.007491	Had menopause	FALSE				163184	119355	43829	UK Biobank	https://docs.google.com/spreadsheets/d/1kvPoupSzsSFBNSztMzl04xMoSC3Kcx3CrjVf4yBmESU/edit?ts=5b5f17db#gid=227859291	PHESANT Transformation:2724_0| | CAT-SINGLE | | reassignments: 2=-1|3=-2 | | Inc(>=10): 1(119355) | | Inc(>=10): 0(43829) | | CAT-SINGLE-BINARY | | sample 43829/119355(163184) | |-Notes:ACE touchscreen question Have you had your menopause (periods stopped)?-Variable type:binary
binary.2814.txt	1710174270056F5forCTG.txt.gz	0.4494	0.04043	11.12	1.04E-28	0.0474	0.003872	0.995	0.01122	-0.01023	0.008992	Ever used hormone-replacement therapy (HRT)	TRUE	Reproductive		Ever used hormone-replacement therapy	193606	76179	117427	UK Biobank	https://docs.google.com/spreadsheets/d/1kvPoupSzsSFBNSztMzl04xMoSC3Kcx3CrjVf4yBmESU/edit?ts=5b5f17db#gid=227859291	PHESANT Transformation:2814_0| | CAT-SINGLE | | Inc(>=10): 1(76179) | | Inc(>=10): 0(117427) | | CAT-SINGLE-BINARY | | sample 117427/76179(193606) | |-Notes:ACE touchscreen question Have you ever used hormone replacement therapy (HRT)?-Variable type:binary
binary.2834.txt	1710174270056F5forCTG.txt.gz	0.3173	0.05452	5.82	5.90E-09	0.02168	0.00348	1.01	0.01055	0.003725	0.007926	Bilateral oophorectomy (both ovaries removed)	FALSE	Reproductive			191515	15629	175886	UK Biobank	https://docs.google.com/spreadsheets/d/1kvPoupSzsSFBNSztMzl04xMoSC3Kcx3CrjVf4yBmESU/edit?ts=5b5f17db#gid=227859291	PHESANT Transformation:2834_0| | CAT-SINGLE | | Inc(>=10): 0(175886) | | Inc(>=10): 1(15629) | | CAT-SINGLE-BINARY | | sample 175886/15629(191515) | |-Notes:ACE touchscreen question Have you had BOTH ovaries removed? If the participant activated the Help button they were shown the message: Only enter Yes if you have had both ovaries removed. If you have only had one ovary removed you will be able to let the interviewer know later in the visit. If you are unsure of whether both ovaries have been removed, select Do not know.-Variable type:binary
binary.2844.txt	1710174270056F5forCTG.txt.gz	0.3301	0.05717	5.774	7.76E-09	0.0258	0.003582	1.028	0.01025	0.009037	0.008886	Had other major operations	FALSE	Other (physical health)			192470	129435	63035	UK Biobank	https://docs.google.com/spreadsheets/d/1kvPoupSzsSFBNSztMzl04xMoSC3Kcx3CrjVf4yBmESU/edit?ts=5b5f17db#gid=227859291	PHESANT Transformation:2844_0| | CAT-SINGLE | | Inc(>=10): 1(129435) | | Inc(>=10): 0(63035) | | CAT-SINGLE-BINARY | | sample 63035/129435(192470) | |-Notes:ACE touchscreen question Have you had any other major operations? (for example, operations that required an overnight stay in hospital) If the participant activated the Help button they were shown the message: If you are unsure if you have had a 'major' operation select Do not know and you will be asked about this by an interviewer later during this visit. The other refers to operations other than hysterectomy and bilateral oophorectomy.-Variable type:binary
binary.2877_1.txt	1710174270056F5forCTG.txt.gz	-0.07486	0.09078	-0.8246	0.4096	0.01869	0.006134	1.003	0.009415	-0.002745	0.008245	Type of tobacco previously smoked: Manufactured cigarettes	FALSE				89224	79113	10111	UK Biobank	https://docs.google.com/spreadsheets/d/1kvPoupSzsSFBNSztMzl04xMoSC3Kcx3CrjVf4yBmESU/edit?ts=5b5f17db#gid=227859291	PHESANT Transformation:2877_0 | | CAT-SINGLE | | CAT-SINGLE-BINARY-VAR: 1 | | Inc(>=10): 1(79113) | |-Notes:ACE touchscreen question What type of tobacco did you usually smoke? If the participant activated the Help button they were shown the message: If you smoked both hand-rolled and manufactured cigarettes select the one that you smoked more of. ~F2877~ was collected from participants who indicated that in the past they smoked on most or all days, as defined by their answers to ~F1249~-Variable type:binary
binary.2877_3.txt	1710174270056F5forCTG.txt.gz	-0.3741	0.2954	-1.267	0.2053	0.004332	0.005483	1.01	0.008361	0.001792	0.007523	Type of tobacco previously smoked: Cigars or pipes	FALSE				89224	4133	85091	UK Biobank	https://docs.google.com/spreadsheets/d/1kvPoupSzsSFBNSztMzl04xMoSC3Kcx3CrjVf4yBmESU/edit?ts=5b5f17db#gid=227859291	PHESANT Transformation:2877_0 | | CAT-SINGLE | | CAT-SINGLE-BINARY-VAR: 3 | | Inc(>=10): 3(4133) | |-Notes:ACE touchscreen question What type of tobacco did you usually smoke? If the participant activated the Help button they were shown the message: If you smoked both hand-rolled and manufactured cigarettes select the one that you smoked more of. ~F2877~ was collected from participants who indicated that in the past they smoked on most or all days, as defined by their answers to ~F1249~-Variable type:binary
binary.2907.txt	1710174270056F5forCTG.txt.gz	0.3865	0.08099	4.772	1.83E-06	0.02374	0.005901	0.991	0.009137	-0.002352	0.007668	Ever stopped smoking for 6+ months	FALSE	Lifestyle			87937	37903	50034	UK Biobank	https://docs.google.com/spreadsheets/d/1kvPoupSzsSFBNSztMzl04xMoSC3Kcx3CrjVf4yBmESU/edit?ts=5b5f17db#gid=227859291	PHESANT Transformation:2907_0| | CAT-SINGLE | | Inc(>=10): 1(37903) | | Inc(>=10): 0(50034) | | CAT-SINGLE-BINARY | | sample 50034/37903(87937) | |-Notes:ACE touchscreen question In the time that you smoked, did you ever stop for more than 6 months? ~F2907~ was collected from participants who indicated that in the past they smoked tobacco on most or all days, as defined by their answers to ~F1249~-Variable type:binary
binary.2986.txt	1710174270056F5forCTG.txt.gz	-0.1677	0.07804	-2.149	0.03163	0.135	0.04631	1.004	0.01102	0.003068	0.007699	Started insulin within one year diagnosis of diabetes	FALSE				16415	1999	14416	UK Biobank	https://docs.google.com/spreadsheets/d/1kvPoupSzsSFBNSztMzl04xMoSC3Kcx3CrjVf4yBmESU/edit?ts=5b5f17db#gid=227859291	PHESANT Transformation:2986_0| | CAT-SINGLE | | Inc(>=10): 0(14416) | | Inc(>=10): 1(1999) | | CAT-SINGLE-BINARY | | sample 14416/1999(16415) | |-Notes:ACE touchscreen question Did you start insulin within one year of your diagnosis of diabetes? ~F2986~ was collected from men who indicated that a doctor had told them they have diabetes, as defined by their answers to ~F2443~ and all women except those who indicated they had diabetes only during pregnancy, as defined by their answers to ~F2443~-Variable type:binary
binary.3005.txt	1710174270056F5forCTG.txt.gz	0.04899	0.115	0.4261	0.67	0.03246	0.01634	0.996	0.009433	-0.004234	0.007973	Fracture resulting from simple fall	FALSE				34310	20384	13926	UK Biobank	https://docs.google.com/spreadsheets/d/1kvPoupSzsSFBNSztMzl04xMoSC3Kcx3CrjVf4yBmESU/edit?ts=5b5f17db#gid=227859291	PHESANT Transformation:3005_0| | CAT-SINGLE | | Inc(>=10): 1(20384) | | Inc(>=10): 0(13926) | | CAT-SINGLE-BINARY | | sample 13926/20384(34310) | |-Notes:ACE touchscreen question Did the fracture result from a simple fall (i.e. from standing height)? If the participant activated the Help button they were shown the message: A simple fall is any fall from standing height or lower. For example if you trip and fall over, this is a simple fall. Falls from a stool or chair that you are sitting on are also counted as simple. Falls from anything higher ie: down a flight of stairs, from a ladder, from standing on a stool or chair are NOT simple falls. If you have had more than one fracture in the last 5 years, select YES if any one of the fractures resulted from a simple fall. ~F3005~ was collected from participants who indicated they have had fractured/broken bones in the last 5 years, as defined by their answers to ~F2463~-Variable type:binary
binary.3090.txt	1710174270056F5forCTG.txt.gz	0.25	0.1173	2.131	0.03308	0.003285	0.001827	1.011	0.009428	0.006556	0.007258	Used an inhaler for chest within last hour	FALSE				331625	2424	329201	UK Biobank	https://docs.google.com/spreadsheets/d/1kvPoupSzsSFBNSztMzl04xMoSC3Kcx3CrjVf4yBmESU/edit?ts=5b5f17db#gid=227859291	PHESANT Transformation:3090_0| | CAT-SINGLE | | reassignments: 9=NA | | Inc(>=10): 0(329201) | | Inc(>=10): 1(2424) | | CAT-SINGLE-BINARY | | sample 329201/2424(331625) | |-Notes:Participants were asked whether they had used a chest inhaler within the hour prior to doing the spirometry test-Variable type:binary
binary.3393.txt	1710174270056F5forCTG.txt.gz	0.1638	0.06941	2.36	0.01827	0.02015	0.003691	0.992	0.0129	0.0076	0.009986	Hearing aid user	FALSE				219358	10942	208416	UK Biobank	https://docs.google.com/spreadsheets/d/1kvPoupSzsSFBNSztMzl04xMoSC3Kcx3CrjVf4yBmESU/edit?ts=5b5f17db#gid=227859291	PHESANT Transformation:3393_0| | CAT-SINGLE | | Inc(>=10): 0(208416) | | Inc(>=10): 1(10942) | | CAT-SINGLE-BINARY | | sample 208416/10942(219358) | |-Notes:ACE touchscreen question Do you use a hearing aid most of the time? Initially this information was collected from all participants answering Yes to either ~F2247~ (~F10793~ in the pilot) or ~F2257~. When the speech-in-noise hearing test was introduced (2009), program logic was altered so that the information was collected from all participants except those who indicated they were completely deaf, as defined by their answers to ~F2247~.-Variable type:binary

binary.3404.txt	1710174270056F5forCTG.txt.gz	0.5663	0.1091	5.191	2.09E-07	0.02227	0.006356	1.005	0.009169	-0.001953	0.00827	Neck/shoulder pain for 3+ months	FALSE	Pain			81276	56156	25120	UK Biobank	https://docs.google.com/spreadsheets/d/1kvPoupSzsSFBNSztMzl04xMoSC3Kcx3CrjVf4yBmESU/edit?ts=5b5f17db#gid=227859291	PHESANT Transformation:3404_0| | CAT-SINGLE | | Inc(>=10): 1(56156) | | Inc(>=10): 0(25120) | | CAT-SINGLE-BINARY | | sample 25120/56156(81276) | |-Notes:ACE touchscreen question Have you had neck or shoulder pains for more than 3 months? ~F3404~ was collected from participants who indicated that in the last month they experienced neck or shoulder pain that interfered with their usual activities, as defined by their answers to ~F6159~-Variable type:binary
binary.3571.txt	1710174270056F5forCTG.txt.gz	0.4869	0.06735	7.229	4.85E-13	0.03976	0.006967	0.986	0.01064	-0.01243	0.008935	Back pain for 3+ months	FALSE	Pain			90555	62055	28500	UK Biobank	https://docs.google.com/spreadsheets/d/1kvPoupSzsSFBNSztMzl04xMoSC3Kcx3CrjVf4yBmESU/edit?ts=5b5f17db#gid=227859291	PHESANT Transformation:3571_0| | CAT-SINGLE | | Inc(>=10): 1(62055) | | Inc(>=10): 0(28500) | | CAT-SINGLE-BINARY | | sample 28500/62055(90555) | |-Notes:ACE touchscreen question Have you had back pains for more than 3 months? ~F3571~ was collected from participants who indicated that in the last month they experienced back pain that interfered with their usual activities, as defined by their answers to ~F6159~-Variable type:binary
binary.3591.txt	1710174270056F5forCTG.txt.gz	0.3079	0.05775	5.331	9.75E-08	0.0244	0.00382	0.997	0.0105	0.01211	0.007951	Ever had hysterectomy (womb removed)	FALSE	Reproductive			171413	13973	157440	UK Biobank	https://docs.google.com/spreadsheets/d/1kvPoupSzsSFBNSztMzl04xMoSC3Kcx3CrjVf4yBmESU/edit?ts=5b5f17db#gid=227859291	PHESANT Transformation:3591_0| | CAT-SINGLE | | Inc(>=10): 0(157440) | | Inc(>=10): 1(13973) | | CAT-SINGLE-BINARY | | sample 157440/13973(171413) | |-Notes:ACE touchscreen question Have you had a hysterectomy (womb removed)? ~F3591~ was collected from all women except those who indicated they have had a hysterectomy (womb removed), as defined by their answers to ~F2724~-Variable type:binary
binary.3606.txt	1710174270056F5forCTG.txt.gz	0.4169	0.05822	7.16	8.04E-13	0.07554	0.01061	1	0.0104	-0.004193	0.008447	Chest pain or discomfort walking normally	FALSE	Pain			55737	9617	46120	UK Biobank	https://docs.google.com/spreadsheets/d/1kvPoupSzsSFBNSztMzl04xMoSC3Kcx3CrjVf4yBmESU/edit?ts=5b5f17db#gid=227859291	PHESANT Transformation:3606_0| | CAT-SINGLE | | Inc(>=10): 0(46120) | | Inc(>=10): 1(9617) | | CAT-SINGLE-BINARY | | sample 46120/9617(55737) | |-Notes:ACE touchscreen question Do you get this pain or discomfort when you walk at an ordinary pace on the level? ~F3606~ was collected from participants who indicated that they get pain or discomfort in their chest, as defined by their answers to ~F2335~-Variable type:binary
binary.3731.txt	1710174270056F5forCTG.txt.gz	0.07892	0.06381	1.237	0.2162	0.1236	0.02322	0.99	0.00922	0.009471	0.00728	Former alcohol drinker	FALSE				23745	12564	11181	UK Biobank	https://docs.google.com/spreadsheets/d/1kvPoupSzsSFBNSztMzl04xMoSC3Kcx3CrjVf4yBmESU/edit?ts=5b5f17db#gid=227859291	PHESANT Transformation:3731_0| | CAT-SINGLE | | Inc(>=10): 1(12564) | | Inc(>=10): 0(11181) | | CAT-SINGLE-BINARY | | sample 11181/12564(23745) | |-Notes:ACE touchscreen question Did you previously drink alcohol? ~F3731~ was collected from participants who indicated they never drink alcohol, as defined by their answers to ~F1558~-Variable type:binary
binary.3741.txt	1710174270056F5forCTG.txt.gz	0.6274	0.3123	2.009	0.04453	0.02021	0.01786	1.013	0.008896	-0.001953	0.007914	Stomach/abdominal pain for 3+ months	FALSE				29747	16580	13167	UK Biobank	https://docs.google.com/spreadsheets/d/1kvPoupSzsSFBNSztMzl04xMoSC3Kcx3CrjVf4yBmESU/edit?ts=5b5f17db#gid=227859291	PHESANT Transformation:3741_0| | CAT-SINGLE | | Inc(>=10): 1(16580) | | Inc(>=10): 0(13167) | | CAT-SINGLE-BINARY | | sample 13167/16580(29747) | |-Notes:ACE touchscreen question Have you had stomach or abdominal pains for more than 3 months? ~F3741~ was collected from participants who indicated that in the last month they experienced stomach or abdominal pain that interfered with their usual activities, as defined by their answers to ~F6159~-Variable type:binary
binary.3751.txt	1710174270056F5forCTG.txt.gz	0.2111	0.08784	2.403	0.01626	0.03535	0.01236	1.014	0.009271	0.01259	0.008122	Chest pain or discomfort when walking uphill or hurrying	FALSE				44867	11938	32929	UK Biobank	https://docs.google.com/spreadsheets/d/1kvPoupSzsSFBNSztMzl04xMoSC3Kcx3CrjVf4yBmESU/edit?ts=5b5f17db#gid=227859291	PHESANT Transformation:3751_0| | CAT-SINGLE | | Inc(>=10): 1(11938) | | Inc(>=10): 0(32929) | | CAT-SINGLE-BINARY | | sample 32929/11938(44867) | |-Notes:ACE touchscreen question Do you get this pain or discomfort when you walk uphill or hurry? ~F3751~ was collected from participants who indicated that they get pain or discomfort in their chest, as defined by their answers to ~F2335~ but they do not get this pain when they walk at an ordinary pace on the level, as defined by their answers to ~F2335~-Variable type:binary
binary.3773.txt	1710174270056F5forCTG.txt.gz	0.4391	0.1036	4.24	2.24E-05	0.02688	0.007428	0.995	0.009285	-0.008313	0.008148	Knee pain for 3+ months	FALSE	Pain			76000	59814	16186	UK Biobank	https://docs.google.com/spreadsheets/d/1kvPoupSzsSFBNSztMzl04xMoSC3Kcx3CrjVf4yBmESU/edit?ts=5b5f17db#gid=227859291	PHESANT Transformation:3773_0| | CAT-SINGLE | | Inc(>=10): 1(59814) | | Inc(>=10): 0(16186) | | CAT-SINGLE-BINARY | | sample 16186/59814(76000) | |-Notes:ACE touchscreen question Have you had knee pains for more than 3 months? ~F3773~ was collected from participants who indicated that in the last month they experienced knee pain that interfered with their usual activities, as defined by their answers to ~F6159~-Variable type:binary
binary.3799.txt	1710174270056F5forCTG.txt.gz	0.1591	0.05836	2.726	0.006407	0.05479	0.008827	1.007	0.009713	0.002906	0.007783	Headaches for 3+ months	FALSE				70181	32075	38106	UK Biobank	https://docs.google.com/spreadsheets/d/1kvPoupSzsSFBNSztMzl04xMoSC3Kcx3CrjVf4yBmESU/edit?ts=5b5f17db#gid=227859291	PHESANT Transformation:3799_0| | CAT-SINGLE | | Inc(>=10): 1(32075) | | Inc(>=10): 0(38106) | | CAT-SINGLE-BINARY | | sample 38106/32075(70181) | |-Notes:ACE touchscreen question Have you had headaches for more than 3 months? ~F3799~ was collected from participants who indicated that in the last month they experienced headache that interfered with their usual activities, as defined by their answers to ~F6159~-Variable type:binary
binary.40001_C349.txt	1710174270056F5forCTG.txt.gz	-0.03162	0.1114	-0.2837	0.7766	0.09726	0.05858	0.996	0.008854	0.0143	0.007583	Underlying (primary) cause of death: ICD10: C34.9 Bronchus or lung, unspecified	FALSE				9092	1100	7992	UK Biobank	https://docs.google.com/spreadsheets/d/1kvPoupSzsSFBNSztMzl04xMoSC3Kcx3CrjVf4yBmESU/edit?ts=5b5f17db#gid=227859291	PHESANT Transformation:40001_0 | | CAT-SINGLE | | CAT-SINGLE-BINARY-VAR: C349 | | Inc(>=10): C349(1100) | |-Notes:Underlying/primary cause of death reported for participant. Note that this may not match the text value in ~F40010~ due to transcription errors at source. Acquired from central registry.-Variable type:binary
binary.40001_C459.txt	1710174270056F5forCTG.txt.gz	0.02667	0.1326	0.2011	0.8407	0.1007	0.06562	0.997	0.01148	-0.001612	0.009708	Underlying (primary) cause of death: ICD10: C45.9 Mesothelioma, unspecified	FALSE				9092	109	8983	UK Biobank	https://docs.google.com/spreadsheets/d/1kvPoupSzsSFBNSztMzl04xMoSC3Kcx3CrjVf4yBmESU/edit?ts=5b5f17db#gid=227859291	PHESANT Transformation:40001_0 | | CAT-SINGLE | | CAT-SINGLE-BINARY-VAR: C459 | | Inc(>=10): C459(109) | |-Notes:Underlying/primary cause of death reported for participant. Note that this may not match the text value in ~F40010~ due to transcription errors at source. Acquired from central registry.-Variable type:binary
binary.40001_J841.txt	1710174270056F5forCTG.txt.gz	-0.197	0.1541	-1.278	0.2011	0.08339	0.06094	0.996	0.0101	0.02474	0.009695	Underlying (primary) cause of death: ICD10: J84.1 Other interstitial pulmonary diseases with fibrosis	FALSE				9092	120	8972	UK Biobank	https://docs.google.com/spreadsheets/d/1kvPoupSzsSFBNSztMzl04xMoSC3Kcx3CrjVf4yBmESU/edit?ts=5b5f17db#gid=227859291	PHESANT Transformation:40001_0 | | CAT-SINGLE | | CAT-SINGLE-BINARY-VAR: J841 | | Inc(>=10): J841(120) | |-Notes:Underlying/primary cause of death reported for participant. Note that this may not match the text value in ~F40010~ due to transcription errors at source. Acquired from central registry.-Variable type:binary
binary.41215_0.txt	1710174270056F5forCTG.txt.gz	0.3597	0.08931	4.028	5.63E-05	0.005707	0.001694	1.018	0.01013	0.01387	0.00788	Detention categories: Informal, not formally detained	FALSE				361194	28228	332966	UK Biobank	https://docs.google.com/spreadsheets/d/1kvPoupSzsSFBNSztMzl04xMoSC3Kcx3CrjVf4yBmESU/edit?ts=5b5f17db#gid=227859291	PHESANT Transformation:41215_0 | | CAT-MUL-BINARY-VAR 0 | | ALL | | Removed 0 examples != 0 but with missing value (<0) | | sample 332966/28228(361194) | |-Notes:This field is a summary of the distinct detention category codes a participant has had recorded across all their episodes in hospital. Detention category identifies the legislation under which the person was detained. In order to conduct analyses using the source information, researchers must select the corresponding Spell and Episode (~F41128~) data-field.-Variable type:binary
binary.41231_1.txt	1710174270056F5forCTG.txt.gz	0.5073	0.05496	9.23	2.70E-20	0.01601	0.002099	1.117	0.01098	0.004877	0.008224	Hospital episode type: General episode	FALSE	Other (physical health)			361194	246807	114387	UK Biobank	https://docs.google.com/spreadsheets/d/1kvPoupSzsSFBNSztMzl04xMoSC3Kcx3CrjVf4yBmESU/edit?ts=5b5f17db#gid=227859291	PHESANT Transformation:41231_0 | | CAT-MUL-BINARY-VAR 1 | | ALL | | Removed 0 examples != 1 but with missing value (<0) | | sample 114387/246807(361194) | |-Notes:This field is a summary of the distinct episode type codes a participant has had recorded across all their episodes in hospital. Hospital episode type identifies whether the period of care in hospital was a general episode or a psychiatric episode, for example. In order to conduct analyses using the source information, researchers must select the corresponding Spell and Episode (~F41132~) data.-Variable type:binary
binary.41248_1000.txt	1710174270056F5forCTG.txt.gz	0.4676	0.04604	10.16	3.10E-24	0.02055	0.002147	1.053	0.0119	0.014	0.008183	Destinations on discharge from hospital (recoded): Usual Place of residence	FALSE	Other			361058	232634	128424	UK Biobank	https://docs.google.com/spreadsheets/d/1kvPoupSzsSFBNSztMzl04xMoSC3Kcx3CrjVf4yBmESU/edit?ts=5b5f17db#gid=227859291	PHESANT Transformation:41248_0 | | CAT-MUL-BINARY-VAR 1000 | | ALL | | Removed 136 examples != 1000 but with missing value (<0) | | sample 128424/232634(361058) | |-Notes:This field is a summary of the distinct destination on discharge codes a participant has had recorded across all their episodes in hospital. Destination on discharge identifies whether a patient was due to return to their usual place of residence on leaving hospital, or whether they were due to go elsewhere. In order to conduct analyses using the source information, researchers must select the corresponding Spell and Episode (~F41103~) data. Note that the original information was obtained from a variety of external sources, some of which used overlapping and contradictory coding schemes. This field has been re-coded to remove such data conflicts.-Variable type:binary
binary.41248_1001.txt	1710174270056F5forCTG.txt.gz	0.05812	0.08098	0.7177	0.4729	0.005554	0.001787	1.117	0.01132	0.008722	0.008321	Destinations on discharge from hospital (recoded): Usual Place of residence: Living with relatives	FALSE				359439	10386	349053	UK Biobank	https://docs.google.com/spreadsheets/d/1kvPoupSzsSFBNSztMzl04xMoSC3Kcx3CrjVf4yBmESU/edit?ts=5b5f17db#gid=227859291	PHESANT Transformation:41248_0 | | CAT-MUL-BINARY-VAR 1001 | | ALL | | Removed 1755 examples != 1001 but with missing value (<0) | | sample 349053/10386(359439) | |-Notes:This field is a summary of the distinct destination on discharge codes a participant has had recorded across all their episodes in hospital. Destination on discharge identifies whether a patient was due to return to their usual place of residence on leaving hospital, or whether they were due to go elsewhere. In order to conduct analyses using the source information, researchers must select the corresponding Spell and Episode (~F41103~) data. Note that the original information was obtained from a variety of external sources, some of which used overlapping and contradictory coding schemes. This field has been re-coded to remove such data conflicts.-Variable type:binary

<table>
<tr><td></td><td></td><td></td><td></td><td></td><td></td><td></td><td></td><td></td><td></td><td></td><td></td><td></td><td></td><td></td><td></td><td></td><td></td><td></td><td></td><td></td><td></td><td></td></tr>
<tr><td>binary.41248_5000.txt</td><td>1710174270056F5forCTG.txt.gz</td><td>0.06131</td><td>0.1075</td><td>0.5701</td><td>0.5686</td><td>0.003449</td><td>0.001482</td><td>0.996</td><td>0.009961</td><td>0.001013</td><td>0.008781</td><td>Destinations on discharge from hospital (recoded): Transfer to other NHS provider</td><td>FALSE</td><td></td><td></td><td></td><td>359437</td><td>438</td><td>358999</td><td>UK Biobank</td><td>https://docs.google.com/spreadsheets/d/1kvPoupSzsSFBNSztMzl04xMoSC3Kcx3CrjVf4yBmESU/edit?ts=5b5f17db#gid=227859291</td><td>PHESANT Transformation:41248_0 || CAT-MUL-BINARY-VAR 5000 || ALL || Removed 1757 examples != 5000 but with missing value (<0) || sample 358999/438(359437) ||-Notes:This field is a summary of the distinct destination on discharge codes a participant has had recorded across all their episodes in hospital. Destination on discharge identifies whether a patient was due to return to their usual place of residence on leaving hospital, or whether they were due to go elsewhere. In order to conduct analyses using the source information, researchers must select the corresponding Spell and Episode (~F41103~) data. Note that the original information was obtained from a variety of external sources, some of which used overlapping and contradictory coding schemes. This field has been re-coded to remove such data conflicts.-Variable type:binary</td></tr>
<tr><td>binary.41248_5001.txt</td><td>1710174270056F5forCTG.txt.gz</td><td>0.2029</td><td>0.1019</td><td>1.991</td><td>0.04647</td><td>0.003249</td><td>0.001482</td><td>0.992</td><td>0.009098</td><td>0.01322</td><td>0.007161</td><td>Destinations on discharge from hospital (recoded): Transfer to other NHS provider: General ward, young physically disabled, A&E</td><td>FALSE</td><td></td><td></td><td></td><td>359670</td><td>5240</td><td>354430</td><td>UK Biobank</td><td>https://docs.google.com/spreadsheets/d/1kvPoupSzsSFBNSztMzl04xMoSC3Kcx3CrjVf4yBmESU/edit?ts=5b5f17db#gid=227859291</td><td>PHESANT Transformation:41248_0 || CAT-MUL-BINARY-VAR 5001 || ALL || Removed 1524 examples != 5001 but with missing value (<0) || sample 354430/5240(359670) ||-Notes:This field is a summary of the distinct destination on discharge codes a participant has had recorded across all their episodes in hospital. Destination on discharge identifies whether a patient was due to return to their usual place of residence on leaving hospital, or whether they were due to go elsewhere. In order to conduct analyses using the source information, researchers must select the corresponding Spell and Episode (~F41103~) data. Note that the original information was obtained from a variety of external sources, some of which used overlapping and contradictory coding schemes. This field has been re-coded to remove such data conflicts.-Variable type:binary</td></tr>
<tr><td>binary.4291.txt</td><td>1710174270056F5forCTG.txt.gz</td><td>0.1749</td><td>0.04249</td><td>4.115</td><td>3.87E-05</td><td>0.06135</td><td>0.006048</td><td>1.005</td><td>0.0116</td><td>-0.01033</td><td>0.008585</td><td>Number of attempts</td><td>FALSE</td><td></td><td></td><td></td><td>119729</td><td>95738</td><td>23991</td><td>UK Biobank</td><td>https://docs.google.com/spreadsheets/d/1kvPoupSzsSFBNSztMzl04xMoSC3Kcx3CrjVf4yBmESU/edit?ts=5b5f17db#gid=227859291</td><td>PHESANT Transformation:4291_0|| INTEGER || CONTINUOUS || >20% IN ONE CATEGORY || Split into three bins: 0: <1, 1: [1,1], 2: >1 ||cat N: 18, 95720, 23991 || Combine first two bins and treat as binary || sample 95738/23991(119729) ||-Notes:-Variable type:binary</td></tr>
<tr><td>binary.4294_0.txt</td><td>1710174270056F5forCTG.txt.gz</td><td>0.2397</td><td>0.08156</td><td>2.939</td><td>0.003289</td><td>0.01948</td><td>0.005083</td><td>1.003</td><td>0.009614</td><td>0.00818</td><td>0.008364</td><td>Final attempt correct: no</td><td>FALSE</td><td></td><td></td><td></td><td>119729</td><td>4581</td><td>115148</td><td>UK Biobank</td><td>https://docs.google.com/spreadsheets/d/1kvPoupSzsSFBNSztMzl04xMoSC3Kcx3CrjVf4yBmESU/edit?ts=5b5f17db#gid=227859291</td><td>PHESANT Transformation:4294_0 || CAT-SINGLE || CAT-SINGLE-BINARY-VAR: 0 || Inc(>=10): 0(4581) ||-Notes:True if final answer given was correct.-Variable type:binary</td></tr>
<tr><td>binary.4294_1.txt</td><td>1710174270056F5forCTG.txt.gz</td><td>-0.2526</td><td>0.08116</td><td>-3.113</td><td>0.001853</td><td>0.0188</td><td>0.004877</td><td>1.006</td><td>0.0094</td><td>-0.007853</td><td>0.008143</td><td>Final attempt correct: yes</td><td>FALSE</td><td></td><td></td><td></td><td>119729</td><td>115007</td><td>4722</td><td>UK Biobank</td><td>https://docs.google.com/spreadsheets/d/1kvPoupSzsSFBNSztMzl04xMoSC3Kcx3CrjVf4yBmESU/edit?ts=5b5f17db#gid=227859291</td><td>PHESANT Transformation:4294_0 || CAT-SINGLE || CAT-SINGLE-BINARY-VAR: 1 || Inc(>=10): 1(115007) ||-Notes:True if final answer given was correct.-Variable type:binary</td></tr>
<tr><td>binary.4501.txt</td><td>1710174270056F5forCTG.txt.gz</td><td>0.1126</td><td>0.08179</td><td>1.376</td><td>0.1688</td><td>0.01885</td><td>0.004969</td><td>0.992</td><td>0.009078</td><td>0.005454</td><td>0.008177</td><td>Non-accidental death in close genetic family</td><td>FALSE</td><td></td><td></td><td></td><td>113705</td><td>31044</td><td>82661</td><td>UK Biobank</td><td>https://docs.google.com/spreadsheets/d/1kvPoupSzsSFBNSztMzl04xMoSC3Kcx3CrjVf4yBmESU/edit?ts=5b5f17db#gid=227859291</td><td>PHESANT Transformation:4501_0|| CAT-SINGLE || Inc(>=10): 1(31044) || Inc(>=10): 0(82661) || CAT-SINGLE-BINARY || sample 82661/31044(113705) ||-Notes:ACE touchscreen question Have any of your mother, father, brothers or sisters died suddenly from a non-accidental cause? (Do not include half-, step- or adopted brothers and sisters)-Variable type:binary</td></tr>
<tr><td>binary.4598.txt</td><td>1710174270056F5forCTG.txt.gz</td><td>0.2902</td><td>0.04687</td><td>6.192</td><td>5.94E-10</td><td>0.06263</td><td>0.005577</td><td>0.991</td><td>0.01052</td><td>-0.01003</td><td>0.008169</td><td>Ever depressed for a whole week</td><td>FALSE</td><td>Psychiatric</td><td></td><td></td><td>117705</td><td>63119</td><td>54586</td><td>UK Biobank</td><td>https://docs.google.com/spreadsheets/d/1kvPoupSzsSFBNSztMzl04xMoSC3Kcx3CrjVf4yBmESU/edit?ts=5b5f17db#gid=227859291</td><td>PHESANT Transformation:4598_0|| CAT-SINGLE || Inc(>=10): 0(54586) || Inc(>=10): 1(63119) || CAT-SINGLE-BINARY || sample 54586/63119(117705) ||-Notes:ACE touchscreen question Looking back over your life, have you ever had a time when you were feeling depressed or down for at least a whole week?-Variable type:binary</td></tr>
<tr><td>binary.4631.txt</td><td>1710174270056F5forCTG.txt.gz</td><td>0.3065</td><td>0.04972</td><td>6.164</td><td>7.09E-10</td><td>0.05915</td><td>0.006595</td><td>0.993</td><td>0.01096</td><td>-0.004799</td><td>0.007922</td><td>Ever unenthusiastic/disinterested for a whole week</td><td>FALSE</td><td>Psychiatric</td><td></td><td></td><td>115145</td><td>42374</td><td>72771</td><td>UK Biobank</td><td>https://docs.google.com/spreadsheets/d/1kvPoupSzsSFBNSztMzl04xMoSC3Kcx3CrjVf4yBmESU/edit?ts=5b5f17db#gid=227859291</td><td>PHESANT Transformation:4631_0|| CAT-SINGLE || Inc(>=10): 0(72771) || Inc(>=10): 1(42374) || CAT-SINGLE-BINARY || sample 72771/42374(115145) ||-Notes:ACE touchscreen question Have you ever had a time when you were uninterested in things or unable to enjoy the things you used to for at least a whole week?-Variable type:binary</td></tr>
<tr><td>binary.4642.txt</td><td>1710174270056F5forCTG.txt.gz</td><td>0.5282</td><td>0.09476</td><td>5.575</td><td>2.48E-08</td><td>0.01938</td><td>0.00553</td><td>0.996</td><td>0.01015</td><td>0.004189</td><td>0.007765</td><td>Ever manic/hyper for 2 days</td><td>TRUE</td><td>Psychiatric</td><td></td><td>Ever manic or hyper for 2 days</td><td>117155</td><td>6569</td><td>110586</td><td>UK Biobank</td><td>https://docs.google.com/spreadsheets/d/1kvPoupSzsSFBNSztMzl04xMoSC3Kcx3CrjVf4yBmESU/edit?ts=5b5f17db#gid=227859291</td><td>PHESANT Transformation:4642_0|| CAT-SINGLE || Inc(>=10): 0(110586) || Inc(>=10): 1(6569) || CAT-SINGLE-BINARY || sample 110586/6569(117155) ||-Notes:ACE touchscreen question Have you ever had a period of time lasting at least two days when you were feeling so good, high, excited or hyper that other people thought you were not your normal self or you were so hyper that you got into trouble?-Variable type:binary</td></tr>
<tr><td>binary.4653.txt</td><td>1710174270056F5forCTG.txt.gz</td><td>0.3593</td><td>0.04956</td><td>7.25</td><td>4.16E-13</td><td>0.05109</td><td>0.005598</td><td>0.987</td><td>0.01069</td><td>0.01382</td><td>0.007755</td><td>Ever highly irritable/argumentative for 2 days</td><td>FALSE</td><td>Psychiatric</td><td></td><td></td><td>117359</td><td>20930</td><td>96429</td><td>UK Biobank</td><td>https://docs.google.com/spreadsheets/d/1kvPoupSzsSFBNSztMzl04xMoSC3Kcx3CrjVf4yBmESU/edit?ts=5b5f17db#gid=227859291</td><td>PHESANT Transformation:4653_0|| CAT-SINGLE || Inc(>=10): 0(96429) || Inc(>=10): 1(20930) || CAT-SINGLE-BINARY || sample 96429/20930(117359) ||-Notes:ACE touchscreen question Have you ever had a period of time lasting at least two days when you were so irritable that you found yourself shouting at people or starting fights or arguments?-Variable type:binary</td></tr>
<tr><td>binary.4717.txt</td><td>1710174270056F5forCTG.txt.gz</td><td>0.4652</td><td>0.04836</td><td>9.619</td><td>6.62E-22</td><td>0.0499</td><td>0.005662</td><td>1.004</td><td>0.01057</td><td>0.00107</td><td>0.007913</td><td>Shortness of breath walking on level ground</td><td>TRUE</td><td>Pulmonary</td><td>Physical health</td><td>Shortness of breath while walking</td><td>118006</td><td>12418</td><td>105588</td><td>UK Biobank</td><td>https://docs.google.com/spreadsheets/d/1kvPoupSzsSFBNSztMzl04xMoSC3Kcx3CrjVf4yBmESU/edit?ts=5b5f17db#gid=227859291</td><td>PHESANT Transformation:4717_0|| CAT-SINGLE || Inc(>=10): 0(105588) || Inc(>=10): 1(12418) || CAT-SINGLE-BINARY || sample 105588/12418(118006) ||-Notes:ACE touchscreen question Do you get short of breath walking with people of your own age on level ground?-Variable type:binary</td></tr>
<tr><td>binary.4728.txt</td><td>1710174270056F5forCTG.txt.gz</td><td>0.5724</td><td>0.05085</td><td>11.26</td><td>2.15E-29</td><td>0.05103</td><td>0.005444</td><td>1.012</td><td>0.01058</td><td>-0.01977</td><td>0.008363</td><td>Leg pain on walking</td><td>FALSE</td><td>Pain</td><td></td><td></td><td>118905</td><td>25997</td><td>92908</td><td>UK Biobank</td><td>https://docs.google.com/spreadsheets/d/1kvPoupSzsSFBNSztMzl04xMoSC3Kcx3CrjVf4yBmESU/edit?ts=5b5f17db#gid=227859291</td><td>PHESANT Transformation:4728_0|| CAT-SINGLE || Inc(>=10): 0(92908) || Inc(>=10): 1(25997) || CAT-SINGLE-BINARY || sample 92908/25997(118905) ||-Notes:ACE touchscreen question Do you get a pain in either leg on walking?-Variable type:binary</td></tr>
<tr><td>binary.4803_0.txt</td><td>1710174270056F5forCTG.txt.gz</td><td>-0.3284</td><td>0.04946</td><td>-6.641</td><td>3.12E-11</td><td>0.05963</td><td>0.005564</td><td>0.999</td><td>0.01031</td><td>-0.003779</td><td>0.008371</td><td>Tinnitus: No, never</td><td>FALSE</td><td>Auditory</td><td></td><td></td><td>117882</td><td>83615</td><td>34267</td><td>UK Biobank</td><td>https://docs.google.com/spreadsheets/d/1kvPoupSzsSFBNSztMzl04xMoSC3Kcx3CrjVf4yBmESU/edit?ts=5b5f17db#gid=227859291</td><td>PHESANT Transformation:4803_0 || CAT-SINGLE || CAT-SINGLE-BINARY-VAR: 0 || Inc(>=10): 0(83615) ||-Notes:ACE touchscreen question Do you get or have you had noises (such as ringing or buzzing) in your head or in one or both ears that lasts for more than five minutes at a time? ~F4803~ was collected from all participants except those who indicated they were completely deaf, as defined by their answers to ~F2247~-Variable type:binary</td></tr>
<tr><td>binary.4803_11.txt</td><td>1710174270056F5forCTG.txt.gz</td><td>0.1865</td><td>0.05986</td><td>3.115</td><td>0.001839</td><td>0.03079</td><td>0.005268</td><td>1.004</td><td>0.009828</td><td>0.0079</td><td>0.008032</td><td>Tinnitus: Yes, now most or all of the time</td><td>FALSE</td><td></td><td></td><td></td><td>117882</td><td>7739</td><td>110143</td><td>UK Biobank</td><td>https://docs.google.com/spreadsheets/d/1kvPoupSzsSFBNSztMzl04xMoSC3Kcx3CrjVf4yBmESU/edit?ts=5b5f17db#gid=227859291</td><td>PHESANT Transformation:4803_0 || CAT-SINGLE || CAT-SINGLE-BINARY-VAR: 11 || Inc(>=10): 11(7739) ||-Notes:ACE touchscreen question Do you get or have you had noises (such as ringing or buzzing) in your head or in one or both ears that lasts for more than five minutes at a time? ~F4803~ was collected from all participants except those who indicated they were completely deaf, as defined by their answers to ~F2247~-Variable type:binary</td></tr>
<tr><td>binary.4803_13.txt</td><td>1710174270056F5forCTG.txt.gz</td><td>0.3804</td><td>0.1341</td><td>2.837</td><td>0.004547</td><td>0.009933</td><td>0.004539</td><td>0.996</td><td>0.008961</td><td>-0.002056</td><td>0.006765</td><td>Tinnitus: Yes, now some of the time</td><td>FALSE</td><td></td><td></td><td></td><td>117882</td><td>10489</td><td>107393</td><td>UK Biobank</td><td>https://docs.google.com/spreadsheets/d/1kvPoupSzsSFBNSztMzl04xMoSC3Kcx3CrjVf4yBmESU/edit?ts=5b5f17db#gid=227859291</td><td>PHESANT Transformation:4803_0 || CAT-SINGLE || CAT-SINGLE-BINARY-VAR: 13 || Inc(>=10): 13(10489) ||-Notes:ACE touchscreen question Do you get or have you had noises (such as ringing or buzzing) in your head or in one or both ears that lasts for more than five minutes at a time? ~F4803~ was collected from all participants except those who indicated they were completely deaf, as defined by their answers to ~F2247~-Variable type:binary</td></tr>
<tr><td>binary.4803_14.txt</td><td>1710174270056F5forCTG.txt.gz</td><td>0.3607</td><td>0.1026</td><td>3.515</td><td>0.000439</td><td>0.01595</td><td>0.004441</td><td>0.994</td><td>0.009597</td><td>-0.002161</td><td>0.007668</td><td>Tinnitus: Yes, but not now, but have in the past</td><td>FALSE</td><td></td><td></td><td></td><td>117882</td><td>13080</td><td>104802</td><td>UK Biobank</td><td>https://docs.google.com/spreadsheets/d/1kvPoupSzsSFBNSztMzl04xMoSC3Kcx3CrjVf4yBmESU/edit?ts=5b5f17db#gid=227859291</td><td>PHESANT Transformation:4803_0 || CAT-SINGLE || CAT-SINGLE-BINARY-VAR: 14 || Inc(>=10): 14(13080) ||-Notes:ACE touchscreen question Do you get or have you had noises (such as ringing or buzzing) in your head or in one or both ears that lasts for more than five minutes at a time? ~F4803~ was collected from all participants except those who indicated they were completely deaf, as defined by their answers to ~F2247~-Variable type:binary</td></tr>
<tr><td>binary.4935.txt</td><td>1710174270056F5forCTG.txt.gz</td><td>-0.7147</td><td>0.184</td><td>-3.884</td><td>0.000103</td><td>0.01058</td><td>0.004761</td><td>1.007</td><td>0.009832</td><td>0.007148</td><td>0.008319</td><td>FI1 : numeric addition test</td><td>FALSE</td><td></td><td></td><td></td><td>116723</td><td>111965</td><td>4758</td><td>UK Biobank</td><td>https://docs.google.com/spreadsheets/d/1kvPoupSzsSFBNSztMzl04xMoSC3Kcx3CrjVf4yBmESU/edit?ts=5b5f17db#gid=227859291</td><td>PHESANT Transformation:4935_0|| CAT-SINGLE || reassignments: 13=0|14=0|15=1|16=0|17=0|-1=0 || Inc(>=10): 1(111965) || Inc(>=10): 0(4758) || CAT-SINGLE-BINARY || sample 4758/111965(116723) ||-Notes:ACE touchscreen question Add the following numbers together: 1 2 3 4 5 - is the answer?. Part 1 of Fluid Intelligence test. Asked to choose from 13, 14, 15, 16 or 17.-Variable type:binary</td></tr>
<tr><td>binary.4957.txt</td><td>1710174270056F5forCTG.txt.gz</td><td>-0.4706</td><td>0.04334</td><td>-10.86</td><td>1.80E-27</td><td>0.0736</td><td>0.006583</td><td>1.02</td><td>0.0109</td><td>0.006539</td><td>0.009109</td><td>FI3 : word interpolation</td><td>TRUE</td><td>Cognitive</td><td>Cognitive</td><td>Word interpolation test</td><td>116815</td><td>98753</td><td>18062</td><td>UK Biobank</td><td>https://docs.google.com/spreadsheets/d/1kvPoupSzsSFBNSztMzl04xMoSC3Kcx3CrjVf4yBmESU/edit?ts=5b5f17db#gid=227859291</td><td>PHESANT Transformation:4957_0|| CAT-SINGLE || reassignments: 1=0|2=0|3=0|4=1|5=0|-1=0 || Inc(>=10): 1(98753) || Inc(>=10): 0(18062) || CAT-SINGLE-BINARY || sample 18062/98753(116815) ||-Notes:ACE touchscreen question Bud is to Flower as Child is to?. Part 3 of Fluid Intelligence test. Offered choice from Grow, Develop, Improve, Adult and Old.-Variable type:binary</td></tr>
<tr><td>binary.4968.txt</td><td>1710174270056F5forCTG.txt.gz</td><td>-0.5805</td><td>0.04776</td><td>-12.16</td><td>5.32E-34</td><td>0.06108</td><td>0.006497</td><td>1.015</td><td>0.01155</td><td>-0.009292</td><td>0.009183</td><td>FI4 : positional arithmetic</td><td>TRUE</td><td>Cognitive</td><td>Cognitive</td><td>Positional arithmetic test</td><td>115690</td><td>98344</td><td>17346</td><td>UK Biobank</td><td>https://docs.google.com/spreadsheets/d/1kvPoupSzsSFBNSztMzl04xMoSC3Kcx3CrjVf4yBmESU/edit?ts=5b5f17db#gid=227859291</td><td>PHESANT Transformation:4968_0|| CAT-SINGLE || reassignments: 5=0|6=1|7=0|8=0|-1=0 || Inc(>=10): 1(98344) || Inc(>=10): 0(17346) || CAT-SINGLE-BINARY || sample 17346/98344(115690) ||-Notes:ACE touchscreen question 11 12 13 14 15 16 17 18 Divide the sixth number to the right of twelve by three. Is the answer?. Part 4 of Fluid Intelligence test. Offered choice from 5, 6, 7, or 8.-Variable type:binary</td></tr>
<tr><td>binary.4979.txt</td><td>1710174270056F5forCTG.txt.gz</td><td>-0.3269</td><td>0.04961</td><td>-6.59</td><td>4.40E-11</td><td>0.04518</td><td>0.006248</td><td>1</td><td>0.01051</td><td>-0.006909</td><td>0.007988</td><td>FI5 : family relationship calculation</td><td>FALSE</td><td>Cognitive</td><td></td><td></td><td>113356</td><td>55922</td><td>57434</td><td>UK Biobank</td><td>https://docs.google.com/spreadsheets/d/1kvPoupSzsSFBNSztMzl04xMoSC3Kcx3CrjVf4yBmESU/edit?ts=5b5f17db#gid=227859291</td><td>PHESANT Transformation:4979_0|| CAT-SINGLE || reassignments: 1=0|2=0|3=0|4=1|5=0|-1=0 || Inc(>=10): 1(55922) || Inc(>=10): 0(57434) || CAT-SINGLE-BINARY || sample 57434/55922(113356) ||-Notes:ACE touchscreen question If Truda's mother's brother is Tim's sister's father, what relation is Truda to Tim?. Part 5 of Fluid Intelligence test. Offered choice from Aunt, Sister, Niece, Cousin, No-relation.-Variable type:binary</td></tr>
</table>

<table>
<tr><td>binary.4990.txt</td><td>1710174270056F5forCTG.txt.gz</td><td>-0.5031</td><td>0.03837</td><td>-13.11</td><td>2.79E-39</td><td>0.08572</td><td>0.006974</td><td>1.012</td><td>0.01094</td><td>0.002306</td><td>0.008609</td><td>FI6 : conditional arithmetic</td><td>FALSE</td><td>Cognitive</td><td>Cognitive</td><td>Conditional arithmetic test</td><td>102181</td><td>69944</td><td>32237</td><td>UK Biobank</td><td>https://docs.google.com/spreadsheets/d/1kvPoupSzsSFBNSztMzl04xMoSC3Kcx3CrjVf4yBmESU/edit?ts=5b5f17db#gid=227859291</td><td>PHESANT Transformation:4990_0| | CAT-SINGLE | | reassignments: 68=0|69=1|70=0|71=0|72=0|-1=0 | | Inc(>=10): 0(32237) | | Inc(>=10): 1(69944) | | CAT-SINGLE-BINARY | | sample 32237/69944(102181) | |-Notes:ACE touchscreen question If sixty is more than half of seventy-five, multiply twenty-three by three. If not subtract 15 from eighty-five. Is the answer?. Part 6 of Fluid Intelligence test. Offered choice from 68, 69, 70, 71, 72.-Variable type:binary</td></tr>
<tr><td>binary.5001.txt</td><td>1710174270056F5forCTG.txt.gz</td><td>-0.5674</td><td>0.06074</td><td>-9.341</td><td>9.51E-21</td><td>0.05198</td><td>0.007904</td><td>1.009</td><td>0.01049</td><td>0.005024</td><td>0.007593</td><td>FI7 : synonym</td><td>TRUE</td><td>Cognitive</td><td>Cognitive</td><td>Synonym naming test</td><td>84560</td><td>74026</td><td>10534</td><td>UK Biobank</td><td>https://docs.google.com/spreadsheets/d/1kvPoupSzsSFBNSztMzl04xMoSC3Kcx3CrjVf4yBmESU/edit?ts=5b5f17db#gid=227859291</td><td>PHESANT Transformation:5001_0| | CAT-SINGLE | | reassignments: 1=0|2=0|3=1|4=0|5=0|-1=0 | | Inc(>=10): 1(74026) | | Inc(>=10): 0(10534) | | CAT-SINGLE-BINARY | | sample 10534/74026(84560) | |-Notes:ACE touchscreen question Stop means the same as?. Part 7 of Fluid Intelligence test. Offered choice from Pause, Close, Cease, Break or Rest.-Variable type:binary</td></tr>
<tr><td>binary.5012.txt</td><td>1710174270056F5forCTG.txt.gz</td><td>-0.5072</td><td>0.04134</td><td>-12.27</td><td>1.32E-34</td><td>0.09657</td><td>0.009027</td><td>1.013</td><td>0.01003</td><td>-0.003947</td><td>0.008139</td><td>FI8 : chained arithmetic</td><td>FALSE</td><td>Cognitive</td><td></td><td></td><td>75692</td><td>49086</td><td>26606</td><td>UK Biobank</td><td>https://docs.google.com/spreadsheets/d/1kvPoupSzsSFBNSztMzl04xMoSC3Kcx3CrjVf4yBmESU/edit?ts=5b5f17db#gid=227859291</td><td>PHESANT Transformation:5012_0| | CAT-SINGLE | | reassignments: 25=0|26=1|27=0|28=0|29=0|-1=0 | | Inc(>=10): 1(49086) | | Inc(>=10): 0(26606) | | CAT-SINGLE-BINARY | | sample 26606/49086(75692) | |-Notes:ACE touchscreen question If David is twenty-one and Owen is nineteen and Daniel is nine years younger than David, what is half their combined age?. Part 8 of Fluid Intelligence test. Offered choice from 25, 26, 27, 28 or 29.-Variable type:binary</td></tr>
<tr><td>binary.5181.txt</td><td>1710174270056F5forCTG.txt.gz</td><td>-0.001513</td><td>0.09337</td><td>-0.0162</td><td>0.9871</td><td>0.02036</td><td>0.006616</td><td>0.991</td><td>0.009145</td><td>0.008196</td><td>0.008244</td><td>Ever had eye surgery</td><td>FALSE</td><td></td><td></td><td></td><td>79912</td><td>5825</td><td>74087</td><td>UK Biobank</td><td>https://docs.google.com/spreadsheets/d/1kvPoupSzsSFBNSztMzl04xMoSC3Kcx3CrjVf4yBmESU/edit?ts=5b5f17db#gid=227859291</td><td>PHESANT Transformation:5181_0| | CAT-SINGLE | | reassignments: 2=1|3=NA | | Inc(>=10): 0(74087) | | Inc(>=10): 1(5825) | | CAT-SINGLE-BINARY | | sample 74087/5825(79912) | |-Notes:Participant asked Ever had eye surgery?-Variable type:binary</td></tr>
<tr><td>binary.5463.txt</td><td>1710174270056F5forCTG.txt.gz</td><td>0.293</td><td>0.1205</td><td>2.431</td><td>0.01506</td><td>0.04418</td><td>0.02196</td><td>1.004</td><td>0.01026</td><td>0.008036</td><td>0.008141</td><td>Leg pain in calf/calves</td><td>FALSE</td><td></td><td></td><td></td><td>25567</td><td>8462</td><td>17105</td><td>UK Biobank</td><td>https://docs.google.com/spreadsheets/d/1kvPoupSzsSFBNSztMzl04xMoSC3Kcx3CrjVf4yBmESU/edit?ts=5b5f17db#gid=227859291</td><td>PHESANT Transformation:5463_0| | CAT-SINGLE | | Inc(>=10): 1(8462) | | Inc(>=10): 0(17105) | | CAT-SINGLE-BINARY | | sample 17105/8462(25567) | |-Notes:ACE touchscreen question Do you get this pain in your calf (calves)? ~F5463~ was collected from participants who indicated that they get a pain in either leg on walking, as defined by their answers to ~F4728~-Variable type:binary</td></tr>
<tr><td>binary.5474.txt</td><td>1710174270056F5forCTG.txt.gz</td><td>0.2136</td><td>0.09126</td><td>2.341</td><td>0.01925</td><td>0.06543</td><td>0.02103</td><td>0.992</td><td>0.009447</td><td>0.008145</td><td>0.007385</td><td>Leg pain when walking uphill or hurrying</td><td>FALSE</td><td></td><td></td><td></td><td>24997</td><td>17250</td><td>7747</td><td>UK Biobank</td><td>https://docs.google.com/spreadsheets/d/1kvPoupSzsSFBNSztMzl04xMoSC3Kcx3CrjVf4yBmESU/edit?ts=5b5f17db#gid=227859291</td><td>PHESANT Transformation:5474_0| | CAT-SINGLE | | Inc(>=10): 0(7747) | | Inc(>=10): 1(17250) | | CAT-SINGLE-BINARY | | sample 7747/17250(24997) | |-Notes:ACE touchscreen question Do you get pain when you walk uphill or hurry? ~F5474~ was collected from participants who indicated that they get a pain in either leg on walking, as defined by their answers to ~F4728~-Variable type:binary</td></tr>
<tr><td>binary.5556.txt</td><td>1710174270056F5forCTG.txt.gz</td><td>-0.5679</td><td>0.05567</td><td>-10.2</td><td>1.97E-24</td><td>0.1525</td><td>0.01859</td><td>1.007</td><td>0.009617</td><td>0.006247</td><td>0.009104</td><td>FI9 : concept interpolation</td><td>TRUE</td><td>Cognitive</td><td>Cognitive</td><td>Concept interpolation test</td><td>35147</td><td>19701</td><td>15446</td><td>UK Biobank</td><td>https://docs.google.com/spreadsheets/d/1kvPoupSzsSFBNSztMzl04xMoSC3Kcx3CrjVf4yBmESU/edit?ts=5b5f17db#gid=227859291</td><td>PHESANT Transformation:5556_0| | CAT-SINGLE | | reassignments: 1=0|2=0|3=0|4=1|5=0|-1=0 | | Inc(>=10): 1(19701) | | Inc(>=10): 0(15446) | | CAT-SINGLE-BINARY | | sample 15446/19701(35147) | |-Notes:ACE touchscreen question Age is to Years as Height is to?. Part 9 of Fluid Intelligence test. Offered choice from Long, Deep, Top, Metres or Tall.-Variable type:binary</td></tr>
<tr><td>binary.5663.txt</td><td>1710174270056F5forCTG.txt.gz</td><td>0.0836</td><td>0.1143</td><td>0.7312</td><td>0.4647</td><td>0.04865</td><td>0.0256</td><td>1</td><td>0.008544</td><td>0.003521</td><td>0.007978</td><td>Length of longest manic/irritable episode</td><td>FALSE</td><td></td><td></td><td></td><td>19474</td><td>15382</td><td>4092</td><td>UK Biobank</td><td>https://docs.google.com/spreadsheets/d/1kvPoupSzsSFBNSztMzl04xMoSC3Kcx3CrjVf4yBmESU/edit?ts=5b5f17db#gid=227859291</td><td>PHESANT Transformation:5663_0| | CAT-SINGLE | | reassignments: 12=11 | | Inc(>=10): 13(4092) | | Inc(>=10): 11(15382) | | CAT-SINGLE-BINARY | | sample 15382/4092(19474) | |-Notes:ACE touchscreen question What is the longest time period that these high or irritable periods have lasted? ~F5663~ was collected from participants who indicated they have had a period of at least two days when they were more high, excited or hyper than their normal self, or so irritable they shouted or started fights or arguments, as defined by their answers to ~F4642~ and ~F4642~-Variable type:binary</td></tr>
<tr><td>binary.5699.txt</td><td>1710174270056F5forCTG.txt.gz</td><td>-0.4652</td><td>0.0605</td><td>-7.689</td><td>1.48E-14</td><td>0.1713</td><td>0.02833</td><td>0.987</td><td>0.009822</td><td>-0.000959</td><td>0.007748</td><td>FI10 : arithmetic sequence recognition</td><td>FALSE</td><td>Cognitive</td><td></td><td></td><td>20880</td><td>16721</td><td>4159</td><td>UK Biobank</td><td>https://docs.google.com/spreadsheets/d/1kvPoupSzsSFBNSztMzl04xMoSC3Kcx3CrjVf4yBmESU/edit?ts=5b5f17db#gid=227859291</td><td>PHESANT Transformation:5699_0| | CAT-SINGLE | | reassignments: 96=0|95=1|94=0|93=0|92=0 | | Inc(>=10): 1(16721) | | Inc(>=10): 0(4159) | | CAT-SINGLE-BINARY | | sample 4159/16721(20880) | |-Notes:ACE touchscreen question 150 ... 137 ... 125 ... 114 ... 104 ... What comes next?. Part 10 of Fluid Intelligence test. Offered choice from 92, 93, 94, 95 or 96.-Variable type:binary</td></tr>
<tr><td>binary.5779.txt</td><td>1710174270056F5forCTG.txt.gz</td><td>-0.4673</td><td>0.1113</td><td>-4.2</td><td>2.67E-05</td><td>0.1239</td><td>0.04495</td><td>0.992</td><td>0.009265</td><td>0.01282</td><td>0.008146</td><td>FI11 : antonym</td><td>FALSE</td><td>Cognitive</td><td></td><td></td><td>12493</td><td>8943</td><td>3550</td><td>UK Biobank</td><td>https://docs.google.com/spreadsheets/d/1kvPoupSzsSFBNSztMzl04xMoSC3Kcx3CrjVf4yBmESU/edit?ts=5b5f17db#gid=227859291</td><td>PHESANT Transformation:5779_0| | CAT-SINGLE | | reassignments: 1=0|2=0|3=0|4=0|5=1|-1=0 | | Inc(>=10): 1(8943) | | Inc(>=10): 0(3550) | | CAT-SINGLE-BINARY | | sample 3550/8943(12493) | |-Notes:ACE touchscreen question Relaxed means the opposite of?. Part 11 of Fluid Intelligence test. Offered choice from Calm, Anxious, Cool, Worried, Tense.-Variable type:binary</td></tr>
<tr><td>binary.5843_1.txt</td><td>1710174270056F5forCTG.txt.gz</td><td>0.07774</td><td>0.08729</td><td>0.8905</td><td>0.3732</td><td>0.05569</td><td>0.01652</td><td>0.985</td><td>0.00853</td><td>-0.000335</td><td>0.007775</td><td>Which eye(s) affected by myopia (short sight): Right eye</td><td>FALSE</td><td></td><td></td><td></td><td>29317</td><td>1607</td><td>27710</td><td>UK Biobank</td><td>https://docs.google.com/spreadsheets/d/1kvPoupSzsSFBNSztMzl04xMoSC3Kcx3CrjVf4yBmESU/edit?ts=5b5f17db#gid=227859291</td><td>PHESANT Transformation:5843_0 | | CAT-SINGLE | | CAT-SINGLE-BINARY-VAR: 1 | | Inc(>=10): 1(1607) | |-Notes:ACE touchscreen question Which eye(s) are affected by myopia (short sight)? ~F5843~ was collected from participants who indicated they wear glasses or contact lenses to correct their myopia, as defined by their answers to ~F6147~-Variable type:binary</td></tr>
<tr><td>binary.5843_3.txt</td><td>1710174270056F5forCTG.txt.gz</td><td>-0.1805</td><td>0.136</td><td>-1.327</td><td>0.1844</td><td>0.02778</td><td>0.01797</td><td>0.998</td><td>0.008603</td><td>0.004899</td><td>0.007404</td><td>Which eye(s) affected by myopia (short sight): Both eyes</td><td>FALSE</td><td></td><td></td><td></td><td>29317</td><td>26184</td><td>3133</td><td>UK Biobank</td><td>https://docs.google.com/spreadsheets/d/1kvPoupSzsSFBNSztMzl04xMoSC3Kcx3CrjVf4yBmESU/edit?ts=5b5f17db#gid=227859291</td><td>PHESANT Transformation:5843_0 | | CAT-SINGLE | | CAT-SINGLE-BINARY-VAR: 3 | | Inc(>=10): 3(26184) | |-Notes:ACE touchscreen question Which eye(s) are affected by myopia (short sight)? ~F5843~ was collected from participants who indicated they wear glasses or contact lenses to correct their myopia, as defined by their answers to ~F6147~-Variable type:binary</td></tr>
<tr><td>binary.5855_1.txt</td><td>1710174270056F5forCTG.txt.gz</td><td>-0.002837</td><td>0.08114</td><td>-0.03497</td><td>0.9721</td><td>0.168</td><td>0.06015</td><td>0.987</td><td>0.01024</td><td>-0.004017</td><td>0.008112</td><td>Which eye(s) affected by astigmatism: Right eye</td><td>FALSE</td><td></td><td></td><td></td><td>9749</td><td>1451</td><td>8298</td><td>UK Biobank</td><td>https://docs.google.com/spreadsheets/d/1kvPoupSzsSFBNSztMzl04xMoSC3Kcx3CrjVf4yBmESU/edit?ts=5b5f17db#gid=227859291</td><td>PHESANT Transformation:5855_0 | | CAT-SINGLE | | CAT-SINGLE-BINARY-VAR: 1 | | Inc(>=10): 1(1451) | |-Notes:ACE touchscreen question Which eye(s) are affected by astigmatism? ~F5855~ was collected from participants who indicated they wear glasses or contact lenses to correct their astigmatism, as defined by their answers to ~F6147~-Variable type:binary</td></tr>
<tr><td>binary.5855_3.txt</td><td>1710174270056F5forCTG.txt.gz</td><td>-0.03617</td><td>0.08488</td><td>-0.4262</td><td>0.67</td><td>0.1519</td><td>0.04441</td><td>0.984</td><td>0.007568</td><td>0.006188</td><td>0.00786</td><td>Which eye(s) affected by astigmatism: Both eyes</td><td>FALSE</td><td></td><td></td><td></td><td>9749</td><td>6704</td><td>3045</td><td>UK Biobank</td><td>https://docs.google.com/spreadsheets/d/1kvPoupSzsSFBNSztMzl04xMoSC3Kcx3CrjVf4yBmESU/edit?ts=5b5f17db#gid=227859291</td><td>PHESANT Transformation:5855_0 | | CAT-SINGLE | | CAT-SINGLE-BINARY-VAR: 3 | | Inc(>=10): 3(6704) | |-Notes:ACE touchscreen question Which eye(s) are affected by astigmatism? ~F5855~ was collected from participants who indicated they wear glasses or contact lenses to correct their astigmatism, as defined by their answers to ~F6147~-Variable type:binary</td></tr>
<tr><td>binary.5992.txt</td><td>1710174270056F5forCTG.txt.gz</td><td>-0.2684</td><td>0.1058</td><td>-2.535</td><td>0.01123</td><td>0.02833</td><td>0.01086</td><td>1.01</td><td>0.009021</td><td>-0.009521</td><td>0.008494</td><td>ECG, phase duration</td><td>FALSE</td><td></td><td></td><td></td><td>53998</td><td>42709</td><td>11289</td><td>UK Biobank</td><td>https://docs.google.com/spreadsheets/d/1kvPoupSzsSFBNSztMzl04xMoSC3Kcx3CrjVf4yBmESU/edit?ts=5b5f17db#gid=227859291</td><td>PHESANT Transformation:5992_0| | INTEGER | | CONTINUOUS | | >20% IN ONE CATEGORY | | Split into three bins: 0: <145, 1: [145,145], 2: >145 | | cat N: 11289, 42702, 7 | | Combine last two bins and treat as binary | | sample 11289/42709(53998) | |-Notes:ECG, phase duration The exercise test consists of many phases. This field contains the duration of a phase in seconds in an exercise test.-Variable type:binary</td></tr>
<tr><td>binary.6015.txt</td><td>1710174270056F5forCTG.txt.gz</td><td>0.2145</td><td>0.1544</td><td>1.39</td><td>0.1646</td><td>0.01527</td><td>0.01045</td><td>1.001</td><td>0.009656</td><td>0.01376</td><td>0.008303</td><td>Chest pain felt during physical activity</td><td>FALSE</td><td></td><td></td><td></td><td>54011</td><td>52067</td><td>1944</td><td>UK Biobank</td><td>https://docs.google.com/spreadsheets/d/1kvPoupSzsSFBNSztMzl04xMoSC3Kcx3CrjVf4yBmESU/edit?ts=5b5f17db#gid=227859291</td><td>PHESANT Transformation:6015_0| | CAT-SINGLE | | reassignments: 2=NA | | reorder 0|2|1 | | Inc(>=10): 1(52067) | | Inc(>=10): 3(1944) | | CAT-SINGLE-BINARY | | sample 52067/1944(54011) | |-Notes:Exercise safety question 2, asked prior to fitness test Do you feel pain in your chest when you do physical activity-Variable type:binary</td></tr>
<tr><td>binary.6020_1.txt</td><td>1710174270056F5forCTG.txt.gz</td><td>0.002239</td><td>0.1032</td><td>0.02169</td><td>0.9827</td><td>0.02087</td><td>0.01058</td><td>1.003</td><td>0.009253</td><td>-0.005157</td><td>0.007956</td><td>Completion status of test: Fully completed</td><td>FALSE</td><td></td><td></td><td></td><td>54007</td><td>50215</td><td>3792</td><td>UK Biobank</td><td>https://docs.google.com/spreadsheets/d/1kvPoupSzsSFBNSztMzl04xMoSC3Kcx3CrjVf4yBmESU/edit?ts=5b5f17db#gid=227859291</td><td>PHESANT Transformation:6020_0 | | CAT-SINGLE | | CAT-SINGLE-BINARY-VAR: 1 | | Inc(>=10): 1(50215) | |-Notes:Completion status of test 1, Fully completed / 31, Participant wanted to stop early / 32, Participant reported chest-pain and/or other discomfort / 33, Heart rate reached safety level / 34, Incomplete - other reason-Variable type:binary</td></tr>
<tr><td>binary.6020_33.txt</td><td>1710174270056F5forCTG.txt.gz</td><td>-0.06523</td><td>0.08477</td><td>-0.7695</td><td>0.4416</td><td>0.02975</td><td>0.01098</td><td>0.998</td><td>0.01006</td><td>0.006197</td><td>0.008031</td><td>Completion status of test: Heart rate reached safety level</td><td>FALSE</td><td></td><td></td><td></td><td>54007</td><td>2923</td><td>51084</td><td>UK Biobank</td><td>https://docs.google.com/spreadsheets/d/1kvPoupSzsSFBNSztMzl04xMoSC3Kcx3CrjVf4yBmESU/edit?ts=5b5f17db#gid=227859291</td><td>PHESANT Transformation:6020_0 | | CAT-SINGLE | | CAT-SINGLE-BINARY-VAR: 33 | | Inc(>=10): 33(2923) | |-Notes:Completion status of test 1, Fully completed / 31, Participant wanted to stop early / 32, Participant reported chest-pain and/or other discomfort / 33, Heart rate reached safety level / 34, Incomplete - other reason-Variable type:binary</td></tr>
<tr><td>binary.6034.txt</td><td>1710174270056F5forCTG.txt.gz</td><td>-0.1295</td><td>0.07846</td><td>-1.651</td><td>0.09874</td><td>0.04002</td><td>0.01122</td><td>1.004</td><td>0.01004</td><td>0.01285</td><td>0.007961</td><td>Target heart rate achieved</td><td>FALSE</td><td></td><td></td><td></td><td>53998</td><td>5554</td><td>48444</td><td>UK Biobank</td><td>https://docs.google.com/spreadsheets/d/1kvPoupSzsSFBNSztMzl04xMoSC3Kcx3CrjVf4yBmESU/edit?ts=5b5f17db#gid=227859291</td><td>PHESANT Transformation:6034_0| | CAT-SINGLE | | Inc(>=10): 0(48444) | | Inc(>=10): 1(5554) | | CAT-SINGLE-BINARY | | sample 48444/5554(53998) | |-Notes:This field specifies whether the target heart rate was achieved during the fitness test. Target HR formula used for biobank participants is = (220 - age) * 0.75.-Variable type:binary</td></tr>
<tr><td>binary.6138_1.txt</td><td>1710174270056F5forCTG.txt.gz</td><td>-0.3445</td><td>0.02659</td><td>-12.95</td><td>2.28E-38</td><td>0.1561</td><td>0.005745</td><td>1.122</td><td>0.02172</td><td>-0.009501</td><td>0.01186</td><td>Qualifications: College or University degree</td><td>TRUE</td><td>Education</td><td>Education</td><td>College or university qualification</td><td>357549</td><td>115981</td><td>241568</td><td>UK Biobank</td><td>https://docs.google.com/spreadsheets/d/1kvPoupSzsSFBNSztMzl04xMoSC3Kcx3CrjVf4yBmESU/edit?ts=5b5f17db#gid=227859291</td><td>PHESANT Transformation:6138_0 | | CAT-MUL-BINARY-VAR 1 | | NO_NAN Remove NA participants 510 | | Removed 3135 examples != 1 but with missing value (<0) | | sample 241568/115981(357549) | |-Notes:ACE touchscreen question Which of the following qualifications do you have? (You can select more than one) The following checks were performed: If code -7 was selected, then no additional choices were allowed. If code -3 was selected, then no additional choices were allowed. If the participant activated the Help button they were shown the message: A levels/AS levels and equivalent includes the Higher School Certificate O levels/GCSEs and equivalent includes the School Certificate.-Variable type:binary</td></tr>
<tr><td>binary.6138_100.txt</td><td>1710174270056F5forCTG.txt.gz</td><td>0.4097</td><td>0.03011</td><td>13.61</td><td>3.60E-42</td><td>0.09764</td><td>0.004283</td><td>1.074</td><td>0.01867</td><td>0.01417</td><td>0.01208</td><td>Qualifications: None of the above</td><td>TRUE</td><td>Education</td><td>Education</td><td>No qualification</td><td>357549</td><td>61093</td><td>296456</td><td>UK Biobank</td><td>https://docs.google.com/spreadsheets/d/1kvPoupSzsSFBNSztMzl04xMoSC3Kcx3CrjVf4yBmESU/edit?ts=5b5f17db#gid=227859291</td><td>PHESANT Transformation:6138_0 | | CAT-MUL-BINARY-VAR 100 | | NO_NAN Remove NA participants 510 | | Removed 3135 examples != 100 but with missing value (<0) | | sample 296456/61093(357549) | |-Notes:ACE touchscreen question Which of the following qualifications do you have? (You can select more than one) The following checks were performed: If code -7 was selected, then no additional choices were allowed. If code -3 was selected, then no additional choices were allowed. If the participant activated the Help button they were shown the message: A levels/AS levels and equivalent includes the Higher School Certificate O levels/GCSEs and equivalent includes the School Certificate.-Variable type:binary</td></tr>
</table>

<table>
<tr><td>binary.6138_3.txt</td><td>1710174270056F5forCTG.txt.gz</td><td>-0.6215</td><td>0.03458</td><td>-17.97</td><td>3.25E-72</td><td>0.04199</td><td>0.00257</td><td>1.026</td><td>0.01261</td><td>-0.01125</td><td>0.0102</td><td>Qualifications: O levels/GCSEs or equivalent</td><td>TRUE</td><td>Education</td><td>Education</td><td>GCSE qualification</td><td>357549</td><td>168672</td><td>188877</td><td>UK Biobank</td><td>https://docs.google.com/spreadsheets/d/1kvPoupSzsSFBNSztMzl04xMoSC3Kcx3CrjVf4yBmESU/edit?ts=5b5f17db#gid=227859291</td><td>PHESANT Transformation:6138_0 || CAT-MUL-BINARY-VAR 3 || NO_NAN Remove NA participants 510 || Removed 3135 examples != 3 but with missing value (<0) || sample 188877/168672(357549) ||-Notes:ACE touchscreen question Which of the following qualifications do you have? (You can select more than one) The following checks were performed: If code -7 was selected, then no additional choices were allowed. If code -3 was selected, then no additional choices were allowed. If the participant activated the Help button they were shown the message: A levels/AS levels and equivalent includes the Higher School Certificate O levels/GCSEs and equivalent includes the School Certificate.-Variable type:binary</td></tr>
<tr><td>binary.6138_4.txt</td><td>1710174270056F5forCTG.txt.gz</td><td>0.4334</td><td>0.04941</td><td>8.771</td><td>1.78E-18</td><td>0.02068</td><td>0.001939</td><td>1.009</td><td>0.01023</td><td>0.01033</td><td>0.008655</td><td>Qualifications: CSEs or equivalent</td><td>FALSE</td><td>Education</td><td></td><td></td><td>357549</td><td>46672</td><td>310877</td><td>UK Biobank</td><td>https://docs.google.com/spreadsheets/d/1kvPoupSzsSFBNSztMzl04xMoSC3Kcx3CrjVf4yBmESU/edit?ts=5b5f17db#gid=227859291</td><td>PHESANT Transformation:6138_0 || CAT-MUL-BINARY-VAR 4 || NO_NAN Remove NA participants 510 || Removed 3135 examples != 4 but with missing value (<0) || sample 310877/46672(357549) || SKIP_val:-3 < 0 ||-Notes:ACE touchscreen question Which of the following qualifications do you have? (You can select more than one) The following checks were performed: If code -7 was selected, then no additional choices were allowed. If code -3 was selected, then no additional choices were allowed. If the participant activated the Help button they were shown the message: A levels/AS levels and equivalent includes the Higher School Certificate O levels/GCSEs and equivalent includes the School Certificate.-Variable type:binary</td></tr>
<tr><td>binary.6138_5.txt</td><td>1710174270056F5forCTG.txt.gz</td><td>0.5677</td><td>0.06111</td><td>9.289</td><td>1.55E-20</td><td>0.01328</td><td>0.001769</td><td>1.006</td><td>0.01045</td><td>0.02073</td><td>0.009037</td><td>Qualifications: NVQ or HND or HNC or equivalent</td><td>TRUE</td><td>Education</td><td>Education</td><td>NVQ/HND/HNC qualification</td><td>357549</td><td>66544</td><td>291005</td><td>UK Biobank</td><td>https://docs.google.com/spreadsheets/d/1kvPoupSzsSFBNSztMzl04xMoSC3Kcx3CrjVf4yBmESU/edit?ts=5b5f17db#gid=227859291</td><td>PHESANT Transformation:6138_0 || CAT-MUL-BINARY-VAR 5 || NO_NAN Remove NA participants 510 || Removed 3135 examples != 5 but with missing value (<0) || sample 291005/66544(357549) ||-Notes:ACE touchscreen question Which of the following qualifications do you have? (You can select more than one) The following checks were performed: If code -7 was selected, then no additional choices were allowed. If code -3 was selected, then no additional choices were allowed. If the participant activated the Help button they were shown the message: A levels/AS levels and equivalent includes the Higher School Certificate O levels/GCSEs and equivalent includes the School Certificate.-Variable type:binary</td></tr>
<tr><td>binary.6138_6.txt</td><td>1710174270056F5forCTG.txt.gz</td><td>-0.3045</td><td>0.03478</td><td>-8.754</td><td>2.06E-18</td><td>0.0405</td><td>0.002356</td><td>1.029</td><td>0.0116</td><td>-0.006453</td><td>0.009487</td><td>Qualifications: Other professional qualifications eg: nursing, teaching</td><td>TRUE</td><td>Education</td><td></td><td></td><td>357549</td><td>104241</td><td>253308</td><td>UK Biobank</td><td>https://docs.google.com/spreadsheets/d/1kvPoupSzsSFBNSztMzl04xMoSC3Kcx3CrjVf4yBmESU/edit?ts=5b5f17db#gid=227859291</td><td>PHESANT Transformation:6138_0 || CAT-MUL-BINARY-VAR 6 || NO_NAN Remove NA participants 510 || Removed 3135 examples != 6 but with missing value (<0) || sample 253308/104241(357549) ||-Notes:ACE touchscreen question Which of the following qualifications do you have? (You can select more than one) The following checks were performed: If code -7 was selected, then no additional choices were allowed. If code -3 was selected, then no additional choices were allowed. If the participant activated the Help button they were shown the message: A levels/AS levels and equivalent includes the Higher School Certificate O levels/GCSEs and equivalent includes the School Certificate.-Variable type:binary</td></tr>
<tr><td>binary.6139_1.txt</td><td>1710174270056F5forCTG.txt.gz</td><td>-0.06681</td><td>0.1029</td><td>-0.6492</td><td>0.5162</td><td>0.003511</td><td>0.00147</td><td>1.005</td><td>0.009081</td><td>0.005951</td><td>0.007476</td><td>Gas or solid-fuel cooking/heating: A gas hob or gas cooker</td><td>FALSE</td><td></td><td></td><td></td><td>360066</td><td>251680</td><td>108386</td><td>UK Biobank</td><td>https://docs.google.com/spreadsheets/d/1kvPoupSzsSFBNSztMzl04xMoSC3Kcx3CrjVf4yBmESU/edit?ts=5b5f17db#gid=227859291</td><td>PHESANT Transformation:6139_0 || CAT-MUL-BINARY-VAR 1 || NO_NAN Remove NA participants 510 || Removed 618 examples != 1 but with missing value (<0) || sample 108386/251680(360066) ||-Notes:ACE touchscreen question Do you have any of the following in your home? (You can select more than one answer) The following checks were performed: If code -7 was selected, then no additional choices were allowed. If code -1 was selected, then no additional choices were allowed. If code -3 was selected, then no additional choices were allowed. If the participant activated the Help button they were shown the message: Solid fuel refers to wood or coal. Regular use is when you use this for most days of the week in the winter time.-Variable type:binary</td></tr>
<tr><td>binary.6139_100.txt</td><td>1710174270056F5forCTG.txt.gz</td><td>0.1074</td><td>0.07552</td><td>1.422</td><td>0.1549</td><td>0.005563</td><td>0.001501</td><td>0.992</td><td>0.009714</td><td>-0.002923</td><td>0.006739</td><td>Gas or solid-fuel cooking/heating: None of the above</td><td>FALSE</td><td></td><td></td><td></td><td>360066</td><td>63653</td><td>296413</td><td>UK Biobank</td><td>https://docs.google.com/spreadsheets/d/1kvPoupSzsSFBNSztMzl04xMoSC3Kcx3CrjVf4yBmESU/edit?ts=5b5f17db#gid=227859291</td><td>PHESANT Transformation:6139_0 || CAT-MUL-BINARY-VAR 100 || NO_NAN Remove NA participants 510 || Removed 618 examples != 100 but with missing value (<0) || sample 296413/63653(360066) ||-Notes:ACE touchscreen question Do you have any of the following in your home? (You can select more than one answer) The following checks were performed: If code -7 was selected, then no additional choices were allowed. If code -1 was selected, then no additional choices were allowed. If code -3 was selected, then no additional choices were allowed. If the participant activated the Help button they were shown the message: Solid fuel refers to wood or coal. Regular use is when you use this for most days of the week in the winter time.-Variable type:binary</td></tr>
<tr><td>binary.6139_2.txt</td><td>1710174270056F5forCTG.txt.gz</td><td>0.07783</td><td>0.07707</td><td>1.01</td><td>0.3125</td><td>0.00617</td><td>0.001653</td><td>1.037</td><td>0.01004</td><td>-0.00969</td><td>0.007632</td><td>Gas or solid-fuel cooking/heating: A gas fire that you use regularly in winter time</td><td>FALSE</td><td></td><td></td><td></td><td>360066</td><td>145315</td><td>214751</td><td>UK Biobank</td><td>https://docs.google.com/spreadsheets/d/1kvPoupSzsSFBNSztMzl04xMoSC3Kcx3CrjVf4yBmESU/edit?ts=5b5f17db#gid=227859291</td><td>PHESANT Transformation:6139_0 || CAT-MUL-BINARY-VAR 2 || NO_NAN Remove NA participants 510 || Removed 618 examples != 2 but with missing value (<0) || sample 214751/145315(360066) || SKIP_val:-3 < 0 || SKIP_val:-1 < 0 ||-Notes:ACE touchscreen question Do you have any of the following in your home? (You can select more than one answer) The following checks were performed: If code -7 was selected, then no additional choices were allowed. If code -1 was selected, then no additional choices were allowed. If code -3 was selected, then no additional choices were allowed. If the participant activated the Help button they were shown the message: Solid fuel refers to wood or coal. Regular use is when you use this for most days of the week in the winter time.-Variable type:binary</td></tr>
<tr><td>binary.6139_3.txt</td><td>1710174270056F5forCTG.txt.gz</td><td>-0.1017</td><td>0.05125</td><td>-1.985</td><td>0.04711</td><td>0.01756</td><td>0.002015</td><td>1.02</td><td>0.01138</td><td>-0.005736</td><td>0.008027</td><td>Gas or solid-fuel cooking/heating: An open solid fuel fire that you use regularly in winter time</td><td>FALSE</td><td></td><td></td><td></td><td>360066</td><td>32662</td><td>327404</td><td>UK Biobank</td><td>https://docs.google.com/spreadsheets/d/1kvPoupSzsSFBNSztMzl04xMoSC3Kcx3CrjVf4yBmESU/edit?ts=5b5f17db#gid=227859291</td><td>PHESANT Transformation:6139_0 || CAT-MUL-BINARY-VAR 3 || NO_NAN Remove NA participants 510 || Removed 618 examples != 3 but with missing value (<0) || sample 327404/32662(360066) ||-Notes:ACE touchscreen question Do you have any of the following in your home? (You can select more than one answer) The following checks were performed: If code -7 was selected, then no additional choices were allowed. If code -1 was selected, then no additional choices were allowed. If code -3 was selected, then no additional choices were allowed. If the participant activated the Help button they were shown the message: Solid fuel refers to wood or coal. Regular use is when you use this for most days of the week in the winter time.-Variable type:binary</td></tr>
<tr><td>binary.6141_1.txt</td><td>1710174270056F5forCTG.txt.gz</td><td>-0.1287</td><td>0.03917</td><td>-3.286</td><td>0.001014</td><td>0.0243</td><td>0.001911</td><td>1.009</td><td>0.01058</td><td>-0.004161</td><td>0.007758</td><td>How are people in household related to participant: Husband, wife or partner</td><td>FALSE</td><td></td><td></td><td></td><td>358963</td><td>265738</td><td>93225</td><td>UK Biobank</td><td>https://docs.google.com/spreadsheets/d/1kvPoupSzsSFBNSztMzl04xMoSC3Kcx3CrjVf4yBmESU/edit?ts=5b5f17db#gid=227859291</td><td>PHESANT Transformation:6141_0 || CAT-MUL-BINARY-VAR 1 || Indicator name x709_0_0 || Remove indicator var NAs: 1076 || Remove indicator var <0: 1155 || Removed 0 examples != 1 but with missing value (<0) || sample 93225/265738(358963) ||-Notes:ACE touchscreen question How are the other people who live with you related to you? (You can select more than one answer) The following checks were performed: If code -3 was selected, then no additional choices were allowed. If the participant activated the Help button they were shown the message: Please select all the options that apply. Answer this question considering all the people who you counted in the household in response to the previous question. ~F6141~ was collected from all participants except those who indicated they were living in a sheltered accommodation or in a care home, as defined by their answers to ~F670~ and living alone, as defined by their answers to ~F670~-Variable type:binary</td></tr>
<tr><td>binary.6141_2.txt</td><td>1710174270056F5forCTG.txt.gz</td><td>-0.06458</td><td>0.08558</td><td>-0.7546</td><td>0.4505</td><td>0.006751</td><td>0.001838</td><td>1.003</td><td>0.01021</td><td>-0.01816</td><td>0.00896</td><td>How are people in household related to participant: Son and/or daughter (include step-children)</td><td>FALSE</td><td></td><td></td><td></td><td>358963</td><td>121071</td><td>237892</td><td>UK Biobank</td><td>https://docs.google.com/spreadsheets/d/1kvPoupSzsSFBNSztMzl04xMoSC3Kcx3CrjVf4yBmESU/edit?ts=5b5f17db#gid=227859291</td><td>PHESANT Transformation:6141_0 || CAT-MUL-BINARY-VAR 2 || Indicator name x709_0_0 || Remove indicator var NAs: 1076 || Remove indicator var <0: 1155 || Removed 0 examples != 2 but with missing value (<0) || sample 237892/121071(358963) ||-Notes:ACE touchscreen question How are the other people who live with you related to you? (You can select more than one answer) The following checks were performed: If code -3 was selected, then no additional choices were allowed. If the participant activated the Help button they were shown the message: Please select all the options that apply. Answer this question considering all the people who you counted in the household in response to the previous question. ~F6141~ was collected from all participants except those who indicated they were living in a sheltered accommodation or in a care home, as defined by their answers to ~F670~ and living alone, as defined by their answers to ~F670~-Variable type:binary</td></tr>
<tr><td>binary.6141_4.txt</td><td>1710174270056F5forCTG.txt.gz</td><td>0.04962</td><td>0.09523</td><td>0.521</td><td>0.6023</td><td>0.003715</td><td>0.00165</td><td>0.995</td><td>0.009623</td><td>0.00557</td><td>0.008191</td><td>How are people in household related to participant: Mother and/or father</td><td>FALSE</td><td></td><td></td><td></td><td>358963</td><td>6302</td><td>352661</td><td>UK Biobank</td><td>https://docs.google.com/spreadsheets/d/1kvPoupSzsSFBNSztMzl04xMoSC3Kcx3CrjVf4yBmESU/edit?ts=5b5f17db#gid=227859291</td><td>PHESANT Transformation:6141_0 || CAT-MUL-BINARY-VAR 4 || Indicator name x709_0_0 || Remove indicator var NAs: 1076 || Remove indicator var <0: 1155 || Removed 0 examples != 4 but with missing value (<0) || sample 352661/6302(358963) ||-Notes:ACE touchscreen question How are the other people who live with you related to you? (You can select more than one answer) The following checks were performed: If code -3 was selected, then no additional choices were allowed. If the participant activated the Help button they were shown the message: Please select all the options that apply. Answer this question considering all the people who you counted in the household in response to the previous question. ~F6141~ was collected from all participants except those who indicated they were living in a sheltered accommodation or in a care home, as defined by their answers to ~F670~ and living alone, as defined by their answers to ~F670~-Variable type:binary</td></tr>
</table>

binary.6141_6.txt	1710174270056F5forCTG.txt.gz	0.6198	0.156	3.974	7.08E-05	0.003483	0.001519	1.002	0.009483	-0.000711	0.00696	How are people in household related to participant: Grandchild	FALSE				358963	2907	356056	UK Biobank	https://docs.google.com/spreadsheets/d/1kvPoupSzsSFBNSztMzl04xMoSC3Kcx3CrjVf4yBmESU/edit?ts=5b5f17db#gid=227859291	PHESANT Transformation:6141_0 | | CAT-MUL-BINARY-VAR 6 | | Indicator name x709_0_0 | | Remove indicator var NAs: 1076 | | Remove indicator var <0: 1155 | | Removed 0 examples != 6 but with missing value (<0) | | sample 356056/2907(358963) | |-Notes:ACE touchscreen question How are the other people who live with you related to you? (You can select more than one answer) The following checks were performed: If code -3 was selected, then no additional choices were allowed. If the participant activated the Help button they were shown the message: Please select all the options that apply. Answer this question considering all the people who you counted in the household in response to the previous question. ~F6141~ was collected from all participants except those who indicated they were living in a sheltered accommodation or in a care home, as defined by their answers to ~F670~ and living alone, as defined by their answers to ~F670~-Variable type:binary
binary.6142_1.txt	1710174270056F5forCTG.txt.gz	-0.1961	0.06025	-3.254	0.001138	0.01331	0.001897	1.014	0.009788	-0.002464	0.008453	Current employment status: In paid employment or self-employed	FALSE				359931	205149	154782	UK Biobank	https://docs.google.com/spreadsheets/d/1kvPoupSzsSFBNSztMzl04xMoSC3Kcx3CrjVf4yBmESU/edit?ts=5b5f17db#gid=227859291	PHESANT Transformation:6142_0 | | CAT-MUL-BINARY-VAR 1 | | NO_NAN Remove NA participants 177 | | Removed 1086 examples != 1 but with missing value (<0) | | sample 154782/205149(359931) | |-Notes:ACE touchscreen question Which of the following describes your current situation? (You can select more than one answer) The following checks were performed: If code -7 was selected, then no additional choices were allowed. If code -3 was selected, then no additional choices were allowed. If the participant activated the Help button they were shown the message: If more than one situation applies, select all that are appropriate.-Variable type:binary
binary.6142_2.txt	1710174270056F5forCTG.txt.gz	-0.2781	0.07791	-3.569	0.000358	0.008118	0.001658	1.005	0.009422	0.004799	0.008308	Current employment status: Retired	FALSE				359931	132931	227000	UK Biobank	https://docs.google.com/spreadsheets/d/1kvPoupSzsSFBNSztMzl04xMoSC3Kcx3CrjVf4yBmESU/edit?ts=5b5f17db#gid=227859291	PHESANT Transformation:6142_0 | | CAT-MUL-BINARY-VAR 2 | | NO_NAN Remove NA participants 177 | | Removed 1086 examples != 2 but with missing value (<0) | | sample 227000/132931(359931) | |-Notes:ACE touchscreen question Which of the following describes your current situation? (You can select more than one answer) The following checks were performed: If code -7 was selected, then no additional choices were allowed. If code -3 was selected, then no additional choices were allowed. If the participant activated the Help button they were shown the message: If more than one situation applies, select all that are appropriate.-Variable type:binary
binary.6142_3.txt	1710174270056F5forCTG.txt.gz	-0.1048	0.08176	-1.282	0.1998	0.004758	0.00143	1.005	0.008601	-0.01055	0.007841	Current employment status: Looking after home and/or family	FALSE				359931	18014	341917	UK Biobank	https://docs.google.com/spreadsheets/d/1kvPoupSzsSFBNSztMzl04xMoSC3Kcx3CrjVf4yBmESU/edit?ts=5b5f17db#gid=227859291	PHESANT Transformation:6142_0 | | CAT-MUL-BINARY-VAR 3 | | NO_NAN Remove NA participants 177 | | Removed 1086 examples != 3 but with missing value (<0) | | sample 341917/18014(359931) | | SKIP_val:-3 < 0 | |-Notes:ACE touchscreen question Which of the following describes your current situation? (You can select more than one answer) The following checks were performed: If code -7 was selected, then no additional choices were allowed. If code -3 was selected, then no additional choices were allowed. If the participant activated the Help button they were shown the message: If more than one situation applies, select all that are appropriate.-Variable type:binary
binary.6142_4.txt	1710174270056F5forCTG.txt.gz	0.5517	0.04763	11.58	5.04E-31	0.02196	0.001996	1.016	0.01023	-0.005149	0.00869	Current employment status: Unable to work because of sickness or disability	TRUE	Occupation	Occupation	Unable to work because of sickness or disability	359931	13756	346175	UK Biobank	https://docs.google.com/spreadsheets/d/1kvPoupSzsSFBNSztMzl04xMoSC3Kcx3CrjVf4yBmESU/edit?ts=5b5f17db#gid=227859291	PHESANT Transformation:6142_0 | | CAT-MUL-BINARY-VAR 4 | | NO_NAN Remove NA participants 177 | | Removed 1086 examples != 4 but with missing value (<0) | | sample 346175/13756(359931) | |-Notes:ACE touchscreen question Which of the following describes your current situation? (You can select more than one answer) The following checks were performed: If code -7 was selected, then no additional choices were allowed. If code -3 was selected, then no additional choices were allowed. If the participant activated the Help button they were shown the message: If more than one situation applies, select all that are appropriate.-Variable type:binary
binary.6142_5.txt	1710174270056F5forCTG.txt.gz	0.1857	0.09444	1.966	0.04925	0.004418	0.001547	0.997	0.008944	0.002271	0.007752	Current employment status: Unemployed	FALSE				359931	5694	354237	UK Biobank	https://docs.google.com/spreadsheets/d/1kvPoupSzsSFBNSztMzl04xMoSC3Kcx3CrjVf4yBmESU/edit?ts=5b5f17db#gid=227859291	PHESANT Transformation:6142_0 | | CAT-MUL-BINARY-VAR 5 | | NO_NAN Remove NA participants 177 | | Removed 1086 examples != 5 but with missing value (<0) | | sample 354237/5694(359931) | |-Notes:ACE touchscreen question Which of the following describes your current situation? (You can select more than one answer) The following checks were performed: If code -7 was selected, then no additional choices were allowed. If code -3 was selected, then no additional choices were allowed. If the participant activated the Help button they were shown the message: If more than one situation applies, select all that are appropriate.-Variable type:binary
binary.6142_6.txt	1710174270056F5forCTG.txt.gz	-0.2988	0.0815	-3.667	0.000246	0.007025	0.001576	1.015	0.009772	-0.0028	0.008128	Current employment status: Doing unpaid or voluntary work	FALSE				359931	13308	346623	UK Biobank	https://docs.google.com/spreadsheets/d/1kvPoupSzsSFBNSztMzl04xMoSC3Kcx3CrjVf4yBmESU/edit?ts=5b5f17db#gid=227859291	PHESANT Transformation:6142_0 | | CAT-MUL-BINARY-VAR 6 | | NO_NAN Remove NA participants 177 | | Removed 1086 examples != 6 but with missing value (<0) | | sample 346623/13308(359931) | |-Notes:ACE touchscreen question Which of the following describes your current situation? (You can select more than one answer) The following checks were performed: If code -7 was selected, then no additional choices were allowed. If code -3 was selected, then no additional choices were allowed. If the participant activated the Help button they were shown the message: If more than one situation applies, select all that are appropriate.-Variable type:binary
binary.6143_1.txt	1710174270056F5forCTG.txt.gz	0.242	0.0549	4.408	1.04E-05	0.02398	0.003138	1.022	0.01001	0.001121	0.008129	Transport type for commuting to job workplace: Car/motor vehicle	FALSE	Lifestyle			190832	152481	38351	UK Biobank	https://docs.google.com/spreadsheets/d/1kvPoupSzsSFBNSztMzl04xMoSC3Kcx3CrjVf4yBmESU/edit?ts=5b5f17db#gid=227859291	PHESANT Transformation:6143_0 | | CAT-MUL-BINARY-VAR 1 | | NO_NAN Remove NA participants 170220 | | Removed 142 examples != 1 but with missing value (<0) | | sample 38351/152481(190832) | |-Notes:ACE touchscreen question What types of transport do you use to get to and from work? (You can select more than one answer) The following checks were performed: If code -7 was selected, then no additional choices were allowed. If code -3 was selected, then no additional choices were allowed. If the participant activated the Help button they were shown the message: If you have more than one 'current job' then answer this question for your MAIN job only. If you use more than one form of transport then select all that apply ~F6143~ was collected from participants who indicated they were in paid employment or self-employed, as defined by their answers to ~F6142~ except those who indicated they always work from home, as defined by their answers to ~F6142~-Variable type:binary
binary.6143_2.txt	1710174270056F5forCTG.txt.gz	-0.266	0.06322	-4.207	2.58E-05	0.01737	0.00338	0.996	0.01031	0.005462	0.007281	Transport type for commuting to job workplace: Walk	FALSE	Lifestyle			190832	30287	160545	UK Biobank	https://docs.google.com/spreadsheets/d/1kvPoupSzsSFBNSztMzl04xMoSC3Kcx3CrjVf4yBmESU/edit?ts=5b5f17db#gid=227859291	PHESANT Transformation:6143_0 | | CAT-MUL-BINARY-VAR 2 | | NO_NAN Remove NA participants 170220 | | Removed 142 examples != 2 but with missing value (<0) | | sample 160545/30287(190832) | |-Notes:ACE touchscreen question What types of transport do you use to get to and from work? (You can select more than one answer) The following checks were performed: If code -7 was selected, then no additional choices were allowed. If code -3 was selected, then no additional choices were allowed. If the participant activated the Help button they were shown the message: If you have more than one 'current job' then answer this question for your MAIN job only. If you use more than one form of transport then select all that apply ~F6143~ was collected from participants who indicated they were in paid employment or self-employed, as defined by their answers to ~F6142~ except those who indicated they always work from home, as defined by their answers to ~F6142~-Variable type:binary
binary.6143_3.txt	1710174270056F5forCTG.txt.gz	-0.3277	0.04958	-6.61	3.84E-11	0.03001	0.003601	1.011	0.01035	0.002959	0.008617	Transport type for commuting to job workplace: Public transport	FALSE	Lifestyle			190832	39655	151177	UK Biobank	https://docs.google.com/spreadsheets/d/1kvPoupSzsSFBNSztMzl04xMoSC3Kcx3CrjVf4yBmESU/edit?ts=5b5f17db#gid=227859291	PHESANT Transformation:6143_0 | | CAT-MUL-BINARY-VAR 3 | | NO_NAN Remove NA participants 170220 | | Removed 142 examples != 3 but with missing value (<0) | | sample 151177/39655(190832) | |-Notes:ACE touchscreen question What types of transport do you use to get to and from work? (You can select more than one answer) The following checks were performed: If code -7 was selected, then no additional choices were allowed. If code -3 was selected, then no additional choices were allowed. If the participant activated the Help button they were shown the message: If you have more than one 'current job' then answer this question for your MAIN job only. If you use more than one form of transport then select all that apply ~F6143~ was collected from participants who indicated they were in paid employment or self-employed, as defined by their answers to ~F6142~ except those who indicated they always work from home, as defined by their answers to ~F6142~-Variable type:binary
binary.6143_4.txt	1710174270056F5forCTG.txt.gz	-0.1769	0.05307	-3.334	0.000856	0.02846	0.003501	1.022	0.01089	0.003043	0.008809	Transport type for commuting to job workplace: Cycle	FALSE				190832	14885	175947	UK Biobank	https://docs.google.com/spreadsheets/d/1kvPoupSzsSFBNSztMzl04xMoSC3Kcx3CrjVf4yBmESU/edit?ts=5b5f17db#gid=227859291	PHESANT Transformation:6143_0 | | CAT-MUL-BINARY-VAR 4 | | NO_NAN Remove NA participants 170220 | | Removed 142 examples != 4 but with missing value (<0) | | sample 175947/14885(190832) | |-Notes:ACE touchscreen question What types of transport do you use to get to and from work? (You can select more than one answer) The following checks were performed: If code -7 was selected, then no additional choices were allowed. If code -3 was selected, then no additional choices were allowed. If the participant activated the Help button they were shown the message: If you have more than one 'current job' then answer this question for your MAIN job only. If you use more than one form of transport then select all that apply ~F6143~ was collected from participants who indicated they were in paid employment or self-employed, as defined by their answers to ~F6142~ except those who indicated they always work from home, as defined by their answers to ~F6142~-Variable type:binary

<table>
<tr><th></th><th></th><th></th><th></th><th></th><th></th><th></th><th></th><th></th><th></th><th></th><th></th><th></th><th></th><th></th><th></th><th></th><th></th><th></th><th></th><th></th><th></th><th></th><th></th></tr>
<tr><td>binary.6144_1.txt</td><td>1710174270056F5forCTG.txt.gz</td><td>0.2556</td><td>0.06906</td><td>3.701</td><td>0.000214</td><td>0.007561</td><td>0.001595</td><td>0.999</td><td>0.009042</td><td>0.009526</td><td>0.00811</td><td>Never eat eggs, dairy, wheat, sugar: Eggs or foods containing eggs</td><td>FALSE</td><td></td><td></td><td></td><td>359777</td><td>9315</td><td>350462</td><td>UK Biobank</td><td>https://docs.google.com/spreadsheets/d/1kvPoupSzsSFBNSztMzl04xMoSC3Kcx3CrjVf4yBmESU/edit?ts=5b5f17db#gid=227859291</td><td>PHESANT Transformation:6144_0 | | CAT-MUL-BINARY-VAR 1 | | NO_NAN Remove NA participants 1417 | | Removed 0 examples != 1 but with missing value (<0) | | sample 350462/9315(359777) | |-Notes:ACE touchscreen question Which of the following do you NEVER eat? (You can select more than one answer) The following checks were performed: If code 5 was selected, then no additional choices were allowed. If code -3 was selected, then no additional choices were allowed.-Variable type:binary</td></tr>
<tr><td>binary.6144_2.txt</td><td>1710174270056F5forCTG.txt.gz</td><td>0.449</td><td>0.09702</td><td>4.628</td><td>3.70E-06</td><td>0.004437</td><td>0.001552</td><td>1.008</td><td>0.009472</td><td>0.01562</td><td>0.007697</td><td>Never eat eggs, dairy, wheat, sugar: Dairy products</td><td>FALSE</td><td>Dietary</td><td></td><td></td><td>359777</td><td>7923</td><td>351854</td><td>UK Biobank</td><td>https://docs.google.com/spreadsheets/d/1kvPoupSzsSFBNSztMzl04xMoSC3Kcx3CrjVf4yBmESU/edit?ts=5b5f17db#gid=227859291</td><td>PHESANT Transformation:6144_0 | | CAT-MUL-BINARY-VAR 2 | | NO_NAN Remove NA participants 1417 | | Removed 0 examples != 2 but with missing value (<0) | | sample 351854/7923(359777) | |-Notes:ACE touchscreen question Which of the following do you NEVER eat? (You can select more than one answer) The following checks were performed: If code 5 was selected, then no additional choices were allowed. If code -3 was selected, then no additional choices were allowed.-Variable type:binary</td></tr>
<tr><td>binary.6144_3.txt</td><td>1710174270056F5forCTG.txt.gz</td><td>0.5135</td><td>0.2055</td><td>2.498</td><td>0.01249</td><td>0.002306</td><td>0.001634</td><td>1.017</td><td>0.009878</td><td>0.01361</td><td>0.008392</td><td>Never eat eggs, dairy, wheat, sugar: Wheat products</td><td>FALSE</td><td></td><td></td><td></td><td>359777</td><td>9573</td><td>350204</td><td>UK Biobank</td><td>https://docs.google.com/spreadsheets/d/1kvPoupSzsSFBNSztMzl04xMoSC3Kcx3CrjVf4yBmESU/edit?ts=5b5f17db#gid=227859291</td><td>PHESANT Transformation:6144_0 | | CAT-MUL-BINARY-VAR 3 | | NO_NAN Remove NA participants 1417 | | Removed 0 examples != 3 but with missing value (<0) | | sample 350204/9573(359777) | |-Notes:ACE touchscreen question Which of the following do you NEVER eat? (You can select more than one answer) The following checks were performed: If code 5 was selected, then no additional choices were allowed. If code -3 was selected, then no additional choices were allowed.-Variable type:binary</td></tr>
<tr><td>binary.6144_4.txt</td><td>1710174270056F5forCTG.txt.gz</td><td>0.3032</td><td>0.03552</td><td>8.536</td><td>1.40E-17</td><td>0.04626</td><td>0.002665</td><td>1.007</td><td>0.01337</td><td>0.007268</td><td>0.009816</td><td>Never eat eggs, dairy, wheat, sugar: Sugar or foods/drinks containing sugar</td><td>FALSE</td><td>Dietary</td><td></td><td></td><td>359777</td><td>67292</td><td>292485</td><td>UK Biobank</td><td>https://docs.google.com/spreadsheets/d/1kvPoupSzsSFBNSztMzl04xMoSC3Kcx3CrjVf4yBmESU/edit?ts=5b5f17db#gid=227859291</td><td>PHESANT Transformation:6144_0 | | CAT-MUL-BINARY-VAR 4 | | NO_NAN Remove NA participants 1417 | | Removed 0 examples != 4 but with missing value (<0) | | sample 292485/67292(359777) | |-Notes:ACE touchscreen question Which of the following do you NEVER eat? (You can select more than one answer) The following checks were performed: If code 5 was selected, then no additional choices were allowed. If code -3 was selected, then no additional choices were allowed.-Variable type:binary</td></tr>
<tr><td>binary.6144_5.txt</td><td>1710174270056F5forCTG.txt.gz</td><td>-0.3452</td><td>0.03264</td><td>-10.57</td><td>3.95E-26</td><td>0.04675</td><td>0.002822</td><td>1.004</td><td>0.01409</td><td>-0.009414</td><td>0.009599</td><td>Never eat eggs, dairy, wheat, sugar: I eat all of the above</td><td>FALSE</td><td>Dietary</td><td></td><td></td><td>359777</td><td>278162</td><td>81615</td><td>UK Biobank</td><td>https://docs.google.com/spreadsheets/d/1kvPoupSzsSFBNSztMzl04xMoSC3Kcx3CrjVf4yBmESU/edit?ts=5b5f17db#gid=227859291</td><td>PHESANT Transformation:6144_0 | | CAT-MUL-BINARY-VAR 5 | | NO_NAN Remove NA participants 1417 | | Removed 0 examples != 5 but with missing value (<0) | | sample 81615/278162(359777) | |-Notes:ACE touchscreen question Which of the following do you NEVER eat? (You can select more than one answer) The following checks were performed: If code 5 was selected, then no additional choices were allowed. If code -3 was selected, then no additional choices were allowed.-Variable type:binary</td></tr>
<tr><td>binary.6145_1.txt</td><td>1710174270056F5forCTG.txt.gz</td><td>0.3675</td><td>0.05867</td><td>6.264</td><td>3.76E-10</td><td>0.01424</td><td>0.001867</td><td>1.015</td><td>0.009943</td><td>0.01176</td><td>0.008523</td><td>Illness, injury, bereavement, stress in last 2 years: Serious illness, injury or assault to yourself</td><td>FALSE</td><td>Wellbeing</td><td></td><td></td><td>358836</td><td>33241</td><td>325595</td><td>UK Biobank</td><td>https://docs.google.com/spreadsheets/d/1kvPoupSzsSFBNSztMzl04xMoSC3Kcx3CrjVf4yBmESU/edit?ts=5b5f17db#gid=227859291</td><td>PHESANT Transformation:6145_0 | | CAT-MUL-BINARY-VAR 1 | | NO_NAN Remove NA participants 512 | | Removed 1846 examples != 1 but with missing value (<0) | | sample 325595/33241(358836) | |-Notes:ACE touchscreen question In the last 2 years have you experienced any of the following? (You can select more than one answer) The following checks were performed: If code -7 was selected, then no additional choices were allowed. If code -3 was selected, then no additional choices were allowed.-Variable type:binary</td></tr>
<tr><td>binary.6145_100.txt</td><td>1710174270056F5forCTG.txt.gz</td><td>-0.4588</td><td>0.05187</td><td>-8.846</td><td>9.04E-19</td><td>0.01957</td><td>0.001851</td><td>1.026</td><td>0.00974</td><td>0.003202</td><td>0.008513</td><td>Illness, injury, bereavement, stress in last 2 years: None of the above</td><td>FALSE</td><td>Wellbeing</td><td></td><td></td><td>358836</td><td>201851</td><td>156985</td><td>UK Biobank</td><td>https://docs.google.com/spreadsheets/d/1kvPoupSzsSFBNSztMzl04xMoSC3Kcx3CrjVf4yBmESU/edit?ts=5b5f17db#gid=227859291</td><td>PHESANT Transformation:6145_0 | | CAT-MUL-BINARY-VAR 100 | | NO_NAN Remove NA participants 512 | | Removed 1846 examples != 100 but with missing value (<0) | | sample 156985/201851(358836) | |-Notes:ACE touchscreen question In the last 2 years have you experienced any of the following? (You can select more than one answer) The following checks were performed: If code -7 was selected, then no additional choices were allowed. If code -3 was selected, then no additional choices were allowed.-Variable type:binary</td></tr>
<tr><td>binary.6145_2.txt</td><td>1710174270056F5forCTG.txt.gz</td><td>0.0009655</td><td>0.06739</td><td>0.01433</td><td>0.9886</td><td>0.01038</td><td>0.001984</td><td>1.012</td><td>0.01105</td><td>-0.006291</td><td>0.008774</td><td>Illness, injury, bereavement, stress in last 2 years: Serious illness, injury or assault of a close relative</td><td>FALSE</td><td></td><td></td><td></td><td>358836</td><td>41459</td><td>317377</td><td>UK Biobank</td><td>https://docs.google.com/spreadsheets/d/1kvPoupSzsSFBNSztMzl04xMoSC3Kcx3CrjVf4yBmESU/edit?ts=5b5f17db#gid=227859291</td><td>PHESANT Transformation:6145_0 | | CAT-MUL-BINARY-VAR 2 | | NO_NAN Remove NA participants 512 | | Removed 1846 examples != 2 but with missing value (<0) | | sample 317377/41459(358836) | |-Notes:ACE touchscreen question In the last 2 years have you experienced any of the following? (You can select more than one answer) The following checks were performed: If code -7 was selected, then no additional choices were allowed. If code -3 was selected, then no additional choices were allowed.-Variable type:binary</td></tr>
<tr><td>binary.6145_3.txt</td><td>1710174270056F5forCTG.txt.gz</td><td>0.3887</td><td>0.1104</td><td>3.522</td><td>0.000428</td><td>0.00452</td><td>0.001601</td><td>1.017</td><td>0.009428</td><td>0.007546</td><td>0.008341</td><td>Illness, injury, bereavement, stress in last 2 years: Death of a close relative</td><td>FALSE</td><td></td><td></td><td></td><td>358836</td><td>75305</td><td>283531</td><td>UK Biobank</td><td>https://docs.google.com/spreadsheets/d/1kvPoupSzsSFBNSztMzl04xMoSC3Kcx3CrjVf4yBmESU/edit?ts=5b5f17db#gid=227859291</td><td>PHESANT Transformation:6145_0 | | CAT-MUL-BINARY-VAR 3 | | NO_NAN Remove NA participants 512 | | Removed 1846 examples != 3 but with missing value (<0) | | sample 283531/75305(358836) | |-Notes:ACE touchscreen question In the last 2 years have you experienced any of the following? (You can select more than one answer) The following checks were performed: If code -7 was selected, then no additional choices were allowed. If code -3 was selected, then no additional choices were allowed.-Variable type:binary</td></tr>
<tr><td>binary.6145_5.txt</td><td>1710174270056F5forCTG.txt.gz</td><td>0.4567</td><td>0.1544</td><td>2.959</td><td>0.003091</td><td>0.003153</td><td>0.001604</td><td>1.008</td><td>0.009922</td><td>-0.00634</td><td>0.00772</td><td>Illness, injury, bereavement, stress in last 2 years: Marital separation/divorce</td><td>FALSE</td><td></td><td></td><td></td><td>358836</td><td>10816</td><td>348020</td><td>UK Biobank</td><td>https://docs.google.com/spreadsheets/d/1kvPoupSzsSFBNSztMzl04xMoSC3Kcx3CrjVf4yBmESU/edit?ts=5b5f17db#gid=227859291</td><td>PHESANT Transformation:6145_0 | | CAT-MUL-BINARY-VAR 5 | | NO_NAN Remove NA participants 512 | | Removed 1846 examples != 5 but with missing value (<0) | | sample 348020/10816(358836) | |-Notes:ACE touchscreen question In the last 2 years have you experienced any of the following? (You can select more than one answer) The following checks were performed: If code -7 was selected, then no additional choices were allowed. If code -3 was selected, then no additional choices were allowed.-Variable type:binary</td></tr>
<tr><td>binary.6145_6.txt</td><td>1710174270056F5forCTG.txt.gz</td><td>0.4326</td><td>0.0384</td><td>11.27</td><td>1.92E-29</td><td>0.03132</td><td>0.002413</td><td>1.016</td><td>0.01159</td><td>-0.001198</td><td>0.008675</td><td>Illness, injury, bereavement, stress in last 2 years: Financial difficulties</td><td>TRUE</td><td>Wellbeing</td><td></td><td>Financial difficulties in the last 2 years</td><td>358836</td><td>40670</td><td>318166</td><td>UK Biobank</td><td>https://docs.google.com/spreadsheets/d/1kvPoupSzsSFBNSztMzl04xMoSC3Kcx3CrjVf4yBmESU/edit?ts=5b5f17db#gid=227859291</td><td>PHESANT Transformation:6145_0 | | CAT-MUL-BINARY-VAR 6 | | NO_NAN Remove NA participants 512 | | Removed 1846 examples != 6 but with missing value (<0) | | sample 318166/40670(358836) | |-Notes:ACE touchscreen question In the last 2 years have you experienced any of the following? (You can select more than one answer) The following checks were performed: If code -7 was selected, then no additional choices were allowed. If code -3 was selected, then no additional choices were allowed.-Variable type:binary</td></tr>
<tr><td>binary.6146_100.txt</td><td>1710174270056F5forCTG.txt.gz</td><td>-0.4789</td><td>0.03801</td><td>-12.6</td><td>2.10E-36</td><td>0.03007</td><td>0.002258</td><td>1.016</td><td>0.01232</td><td>-0.01744</td><td>0.008912</td><td>Attendance/disability/mobility allowance: None of the above</td><td>FALSE</td><td>Wellbeing</td><td></td><td></td><td>358597</td><td>337879</td><td>20718</td><td>UK Biobank</td><td>https://docs.google.com/spreadsheets/d/1kvPoupSzsSFBNSztMzl04xMoSC3Kcx3CrjVf4yBmESU/edit?ts=5b5f17db#gid=227859291</td><td>PHESANT Transformation:6146_0 | | CAT-MUL-BINARY-VAR 100 | | NO_NAN Remove NA participants 179 | | Removed 2418 examples != 100 but with missing value (<0) | | sample 20718/337879(358597) | |-Notes:ACE touchscreen question Do you receive any of the following? (You can select more than one answer) The following checks were performed: If code -7 was selected, then no additional choices were allowed. If code -1 was selected, then no additional choices were allowed. If code -3 was selected, then no additional choices were allowed. If the participant activated the Help button they were shown the message: Only select a response if you personally receive the benefit. Do not include if your spouse or someone in your household receives one of these benefits.-Variable type:binary</td></tr>
<tr><td>binary.6146_2.txt</td><td>1710174270056F5forCTG.txt.gz</td><td>0.4843</td><td>0.0396</td><td>12.23</td><td>2.10E-34</td><td>0.02494</td><td>0.001963</td><td>1.005</td><td>0.01073</td><td>0.01443</td><td>0.008349</td><td>Attendance/disability/mobility allowance: Disability living allowance</td><td>TRUE</td><td>Wellbeing</td><td></td><td>Receive disability living allowance</td><td>358597</td><td>15697</td><td>342900</td><td>UK Biobank</td><td>https://docs.google.com/spreadsheets/d/1kvPoupSzsSFBNSztMzl04xMoSC3Kcx3CrjVf4yBmESU/edit?ts=5b5f17db#gid=227859291</td><td>PHESANT Transformation:6146_0 | | CAT-MUL-BINARY-VAR 2 | | NO_NAN Remove NA participants 179 | | Removed 2418 examples != 2 but with missing value (<0) | | sample 342900/15697(358597) | |-Notes:ACE touchscreen question Do you receive any of the following? (You can select more than one answer) The following checks were performed: If code -7 was selected, then no additional choices were allowed. If code -1 was selected, then no additional choices were allowed. If code -3 was selected, then no additional choices were allowed. If the participant activated the Help button they were shown the message: Only select a response if you personally receive the benefit. Do not include if your spouse or someone in your household receives one of these benefits.-Variable type:binary</td></tr>
<tr><td>binary.6146_3.txt</td><td>1710174270056F5forCTG.txt.gz</td><td>0.4605</td><td>0.04654</td><td>9.894</td><td>4.41E-23</td><td>0.02059</td><td>0.001966</td><td>1.022</td><td>0.01108</td><td>0.01946</td><td>0.008538</td><td>Attendance/disability/mobility allowance: Blue badge</td><td>FALSE</td><td>Wellbeing</td><td></td><td></td><td>358597</td><td>12733</td><td>345864</td><td>UK Biobank</td><td>https://docs.google.com/spreadsheets/d/1kvPoupSzsSFBNSztMzl04xMoSC3Kcx3CrjVf4yBmESU/edit?ts=5b5f17db#gid=227859291</td><td>PHESANT Transformation:6146_0 | | CAT-MUL-BINARY-VAR 3 | | NO_NAN Remove NA participants 179 | | Removed 2418 examples != 3 but with missing value (<0) | | sample 345864/12733(358597) | | SKIP_val:-1 < 0 | | SKIP_val:-3 < 0 | |-Notes:ACE touchscreen question Do you receive any of the following? (You can select more than one answer) The following checks were performed: If code -7 was selected, then no additional choices were allowed. If code -1 was selected, then no additional choices were allowed. If code -3 was selected, then no additional choices were allowed. If the participant activated the Help button they were shown the message: Only select a response if you personally receive the benefit. Do not include if your spouse or someone in your household receives one of these benefits.-Variable type:binary</td></tr>
<tr><td>binary.6147_1.txt</td><td>1710174270056F5forCTG.txt.gz</td><td>-0.2036</td><td>0.04565</td><td>-4.46</td><td>8.18E-06</td><td>0.02368</td><td>0.002452</td><td>1.092</td><td>0.01359</td><td>-0.006877</td><td>0.00899</td><td>Reason for glasses/contact lenses: For short-sightedness, i.e. only or mainly for distance viewing such as driving, cinema etc (called 'myopia')</td><td>FALSE</td><td>Ophthalmic</td><td></td><td></td><td>360677</td><td>29318</td><td>331359</td><td>UK Biobank</td><td>https://docs.google.com/spreadsheets/d/1kvPoupSzsSFBNSztMzl04xMoSC3Kcx3CrjVf4yBmESU/edit?ts=5b5f17db#gid=227859291</td><td>PHESANT Transformation:6147_0 | | CAT-MUL-BINARY-VAR 1 | | Indicator name x2207_0_0 | | Remove indicator var NAs: 179 | | Remove indicator var <0: 338 | | Removed 0 examples != 1 but with missing value (<0) | | sample 331359/29318(360677) | |-Notes:ACE touchscreen question Why were you prescribed glasses/contacts? (You can select more than one answer) The following checks were performed: If code -1 was selected, then no additional choices were allowed. If code -3 was selected, then no additional choices were allowed. ~F6147~ was collected from participants who indicated they wear glasses or contact lenses to correct their vision, as defined by their answers to ~F2207~-Variable type:binary</td></tr>
</table>

<table>
<tr><td>binary.6147_2.txt</td><td>1710174270056F5 forCTG.txt.gz</td><td>0.03326</td><td>0.07168</td><td>0.464</td><td>0.6426</td><td>0.00756</td><td>0.001611</td><td>1.049</td><td>0.0104</td><td>0.01455</td><td>0.007937</td><td>Reason for glasses/contact lenses: For long-sightedness, i.e. for distance and near, but particularly for near tasks like reading (called 'hypermetropia')</td><td>FALSE</td><td></td><td></td><td></td><td>360677</td><td>15091</td><td>345586</td><td>UK Biobank</td><td>https://docs.google.com/spreadsheets/d/1kvPoupSzsSFBNSztMzl04xMoSC3Kcx3CrjVf4yBmESU/edit?ts=5b5f17db#gid=227859291</td><td>PHESANT Transformation:6147_0 || CAT-MUL-BINARY-VAR 2 || Indicator name x2207_0_0 || Remove indicator var NAs: 179 || Remove indicator var <0: 338 || Removed 0 examples != 2 but with missing value (<0) || sample 345586/15091(360677) ||-Notes:ACE touchscreen question Why were you prescribed glasses/contacts? (You can select more than one answer) The following checks were performed: If code -1 was selected, then no additional choices were allowed. If code -3 was selected, then no additional choices were allowed. ~F6147~ was collected from participants who indicated they wear glasses or contact lenses to correct their vision, as defined by their answers to ~F2207~-Variable type:binary</td></tr>
<tr><td>binary.6147_3.txt</td><td>1710174270056F5 forCTG.txt.gz</td><td>0.01466</td><td>0.09668</td><td>0.1516</td><td>0.8795</td><td>0.003957</td><td>0.001682</td><td>1.087</td><td>0.01045</td><td>-0.002368</td><td>0.008018</td><td>Reason for glasses/contact lenses: For just reading/near work as you are getting older (called 'presbyopia')</td><td>FALSE</td><td></td><td></td><td></td><td>360677</td><td>31496</td><td>329181</td><td>UK Biobank</td><td>https://docs.google.com/spreadsheets/d/1kvPoupSzsSFBNSztMzl04xMoSC3Kcx3CrjVf4yBmESU/edit?ts=5b5f17db#gid=227859291</td><td>PHESANT Transformation:6147_0 || CAT-MUL-BINARY-VAR 3 || Indicator name x2207_0_0 || Remove indicator var NAs: 179 || Remove indicator var <0: 338 || Removed 0 examples != 3 but with missing value (<0) || sample 329181/31496(360677) ||-Notes:ACE touchscreen question Why were you prescribed glasses/contacts? (You can select more than one answer) The following checks were performed: If code -1 was selected, then no additional choices were allowed. If code -3 was selected, then no additional choices were allowed. ~F6147~ was collected from participants who indicated they wear glasses or contact lenses to correct their vision, as defined by their answers to ~F2207~-Variable type:binary</td></tr>
<tr><td>binary.6147_4.txt</td><td>1710174270056F5 forCTG.txt.gz</td><td>-0.2014</td><td>0.0648</td><td>-3.107</td><td>0.001887</td><td>0.008785</td><td>0.001802</td><td>1.015</td><td>0.01043</td><td>0.0005052</td><td>0.008286</td><td>Reason for glasses/contact lenses: For 'astigmatism'</td><td>FALSE</td><td></td><td></td><td></td><td>360677</td><td>9752</td><td>350925</td><td>UK Biobank</td><td>https://docs.google.com/spreadsheets/d/1kvPoupSzsSFBNSztMzl04xMoSC3Kcx3CrjVf4yBmESU/edit?ts=5b5f17db#gid=227859291</td><td>PHESANT Transformation:6147_0 || CAT-MUL-BINARY-VAR 4 || Indicator name x2207_0_0 || Remove indicator var NAs: 179 || Remove indicator var <0: 338 || Removed 0 examples != 4 but with missing value (<0) || sample 350925/9752(360677) ||-Notes:ACE touchscreen question Why were you prescribed glasses/contacts? (You can select more than one answer) The following checks were performed: If code -1 was selected, then no additional choices were allowed. If code -3 was selected, then no additional choices were allowed. ~F6147~ was collected from participants who indicated they wear glasses or contact lenses to correct their vision, as defined by their answers to ~F2207~-Variable type:binary</td></tr>
<tr><td>binary.6147_5.txt</td><td>1710174270056F5 forCTG.txt.gz</td><td>-0.05178</td><td>0.09563</td><td>-0.5414</td><td>0.5882</td><td>0.003556</td><td>0.001462</td><td>1.005</td><td>0.01027</td><td>0.01758</td><td>0.007232</td><td>Reason for glasses/contact lenses: For a 'squint' or 'turn' in an eye since childhood (called 'strabismus')</td><td>FALSE</td><td></td><td></td><td></td><td>360677</td><td>1566</td><td>359111</td><td>UK Biobank</td><td>https://docs.google.com/spreadsheets/d/1kvPoupSzsSFBNSztMzl04xMoSC3Kcx3CrjVf4yBmESU/edit?ts=5b5f17db#gid=227859291</td><td>PHESANT Transformation:6147_0 || CAT-MUL-BINARY-VAR 5 || Indicator name x2207_0_0 || Remove indicator var NAs: 179 || Remove indicator var <0: 338 || Removed 0 examples != 5 but with missing value (<0) || sample 359111/1566(360677) ||-Notes:ACE touchscreen question Why were you prescribed glasses/contacts? (You can select more than one answer) The following checks were performed: If code -1 was selected, then no additional choices were allowed. If code -3 was selected, then no additional choices were allowed. ~F6147~ was collected from participants who indicated they wear glasses or contact lenses to correct their vision, as defined by their answers to ~F2207~-Variable type:binary</td></tr>
<tr><td>binary.6147_6.txt</td><td>1710174270056F5 forCTG.txt.gz</td><td>-0.007223</td><td>0.1176</td><td>-0.0614</td><td>0.951</td><td>0.002858</td><td>0.001525</td><td>1.012</td><td>0.008637</td><td>7.33E-06</td><td>0.00856</td><td>Reason for glasses/contact lenses: For a 'lazy' eye or an eye with poor vision since childhood (called 'amblyopia')</td><td>FALSE</td><td></td><td></td><td></td><td>360677</td><td>2964</td><td>357713</td><td>UK Biobank</td><td>https://docs.google.com/spreadsheets/d/1kvPoupSzsSFBNSztMzl04xMoSC3Kcx3CrjVf4yBmESU/edit?ts=5b5f17db#gid=227859291</td><td>PHESANT Transformation:6147_0 || CAT-MUL-BINARY-VAR 6 || Indicator name x2207_0_0 || Remove indicator var NAs: 179 || Remove indicator var <0: 338 || Removed 0 examples != 6 but with missing value (<0) || sample 357713/2964(360677) ||-Notes:ACE touchscreen question Why were you prescribed glasses/contacts? (You can select more than one answer) The following checks were performed: If code -1 was selected, then no additional choices were allowed. If code -3 was selected, then no additional choices were allowed. ~F6147~ was collected from participants who indicated they wear glasses or contact lenses to correct their vision, as defined by their answers to ~F2207~-Variable type:binary</td></tr>
<tr><td>binary.6148_1.txt</td><td>1710174270056F5 forCTG.txt.gz</td><td>0.2949</td><td>0.08629</td><td>3.418</td><td>0.000632</td><td>0.01681</td><td>0.005064</td><td>1.012</td><td>0.009339</td><td>-0.003954</td><td>0.007819</td><td>Eye problems/disorders: Diabetes related eye disease</td><td>FALSE</td><td></td><td></td><td></td><td>117890</td><td>2249</td><td>115641</td><td>UK Biobank</td><td>https://docs.google.com/spreadsheets/d/1kvPoupSzsSFBNSztMzl04xMoSC3Kcx3CrjVf4yBmESU/edit?ts=5b5f17db#gid=227859291</td><td>PHESANT Transformation:6148_0 || CAT-MUL-BINARY-VAR 1 || NO_NAN Remove NA participants 240412 || Removed 2892 examples != 1 but with missing value (<0) || sample 115641/2249(117890) ||-Notes:ACE touchscreen question Has a doctor told you that you have any of the following problems with your eyes? (You can select more than one answer) The following checks were performed: If code -7 was selected, then no additional choices were allowed. If code -3 was selected, then no additional choices were allowed. If code -1 was selected, then no additional choices were allowed. If the participant activated the Help button they were shown the message: If you are not sure if you have had any of the listed eye problems enter Do not know.-Variable type:binary</td></tr>
<tr><td>binary.6148_100.txt</td><td>1710174270056F5 forCTG.txt.gz</td><td>-0.1754</td><td>0.07154</td><td>-2.451</td><td>0.01423</td><td>0.02419</td><td>0.005649</td><td>1.022</td><td>0.009741</td><td>-0.007298</td><td>0.008171</td><td>Eye problems/disorders: None of the above</td><td>FALSE</td><td></td><td></td><td></td><td>117890</td><td>91941</td><td>25949</td><td>UK Biobank</td><td>https://docs.google.com/spreadsheets/d/1kvPoupSzsSFBNSztMzl04xMoSC3Kcx3CrjVf4yBmESU/edit?ts=5b5f17db#gid=227859291</td><td>PHESANT Transformation:6148_0 || CAT-MUL-BINARY-VAR 100 || NO_NAN Remove NA participants 240412 || Removed 2892 examples != 100 but with missing value (<0) || sample 25949/91941(117890) ||-Notes:ACE touchscreen question Has a doctor told you that you have any of the following problems with your eyes? (You can select more than one answer) The following checks were performed: If code -7 was selected, then no additional choices were allowed. If code -3 was selected, then no additional choices were allowed. If code -1 was selected, then no additional choices were allowed. If the participant activated the Help button they were shown the message: If you are not sure if you have had any of the listed eye problems enter Do not know.-Variable type:binary</td></tr>
<tr><td>binary.6148_2.txt</td><td>1710174270056F5 forCTG.txt.gz</td><td>0.008863</td><td>0.05538</td><td>0.16</td><td>0.8728</td><td>0.0412</td><td>0.006923</td><td>1.014</td><td>0.0125</td><td>0.0004357</td><td>0.009074</td><td>Eye problems/disorders: Glaucoma</td><td>FALSE</td><td></td><td></td><td></td><td>117890</td><td>5092</td><td>112798</td><td>UK Biobank</td><td>https://docs.google.com/spreadsheets/d/1kvPoupSzsSFBNSztMzl04xMoSC3Kcx3CrjVf4yBmESU/edit?ts=5b5f17db#gid=227859291</td><td>PHESANT Transformation:6148_0 || CAT-MUL-BINARY-VAR 2 || NO_NAN Remove NA participants 240412 || Removed 2892 examples != 2 but with missing value (<0) || sample 112798/5092(117890) ||-Notes:ACE touchscreen question Has a doctor told you that you have any of the following problems with your eyes? (You can select more than one answer) The following checks were performed: If code -7 was selected, then no additional choices were allowed. If code -3 was selected, then no additional choices were allowed. If code -1 was selected, then no additional choices were allowed. If the participant activated the Help button they were shown the message: If you are not sure if you have had any of the listed eye problems enter Do not know.-Variable type:binary</td></tr>
<tr><td>binary.6148_4.txt</td><td>1710174270056F5 forCTG.txt.gz</td><td>0.1038</td><td>0.08039</td><td>1.291</td><td>0.1968</td><td>0.01651</td><td>0.004863</td><td>1.023</td><td>0.009838</td><td>-0.000404</td><td>0.007599</td><td>Eye problems/disorders: Cataract</td><td>FALSE</td><td></td><td></td><td></td><td>117890</td><td>11194</td><td>106696</td><td>UK Biobank</td><td>https://docs.google.com/spreadsheets/d/1kvPoupSzsSFBNSztMzl04xMoSC3Kcx3CrjVf4yBmESU/edit?ts=5b5f17db#gid=227859291</td><td>PHESANT Transformation:6148_0 || CAT-MUL-BINARY-VAR 4 || NO_NAN Remove NA participants 240412 || Removed 2892 examples != 4 but with missing value (<0) || sample 106696/11194(117890) || SKIP_val:-1 < 0 ||-Notes:ACE touchscreen question Has a doctor told you that you have any of the following problems with your eyes? (You can select more than one answer) The following checks were performed: If code -7 was selected, then no additional choices were allowed. If code -3 was selected, then no additional choices were allowed. If code -1 was selected, then no additional choices were allowed. If the participant activated the Help button they were shown the message: If you are not sure if you have had any of the listed eye problems enter Do not know.-Variable type:binary</td></tr>
<tr><td>binary.6149_1.txt</td><td>1710174270056F5 forCTG.txt.gz</td><td>0.1229</td><td>0.04658</td><td>2.638</td><td>0.008329</td><td>0.02843</td><td>0.003891</td><td>1.023</td><td>0.01875</td><td>0.00605</td><td>0.009103</td><td>Mouth/teeth dental problems: Mouth ulcers</td><td>FALSE</td><td></td><td></td><td></td><td>359841</td><td>36831</td><td>323010</td><td>UK Biobank</td><td>https://docs.google.com/spreadsheets/d/1kvPoupSzsSFBNSztMzl04xMoSC3Kcx3CrjVf4yBmESU/edit?ts=5b5f17db#gid=227859291</td><td>PHESANT Transformation:6149_0 || CAT-MUL-BINARY-VAR 1 || NO_NAN Remove NA participants 512 || Removed 841 examples != 1 but with missing value (<0) || sample 323010/36831(359841) ||-Notes:ACE touchscreen question Do you have any of the following? (You can select more than one answer) The following checks were performed: If code -7 was selected, then no additional choices were allowed. If code -3 was selected, then no additional choices were allowed. If the participant activated the Help button they were shown the message: Answer this question thinking about the past year.-Variable type:binary</td></tr>
<tr><td>binary.6149_100.txt</td><td>1710174270056F5 forCTG.txt.gz</td><td>-0.3027</td><td>0.03809</td><td>-7.946</td><td>1.93E-15</td><td>0.03781</td><td>0.002543</td><td>1.018</td><td>0.01357</td><td>-0.007904</td><td>0.009285</td><td>Mouth/teeth dental problems: None of the above</td><td>FALSE</td><td>Dental</td><td></td><td></td><td>359841</td><td>218346</td><td>141495</td><td>UK Biobank</td><td>https://docs.google.com/spreadsheets/d/1kvPoupSzsSFBNSztMzl04xMoSC3Kcx3CrjVf4yBmESU/edit?ts=5b5f17db#gid=227859291</td><td>PHESANT Transformation:6149_0 || CAT-MUL-BINARY-VAR 100 || NO_NAN Remove NA participants 512 || Removed 841 examples != 100 but with missing value (<0) || sample 141495/218346(359841) ||-Notes:ACE touchscreen question Do you have any of the following? (You can select more than one answer) The following checks were performed: If code -7 was selected, then no additional choices were allowed. If code -3 was selected, then no additional choices were allowed. If the participant activated the Help button they were shown the message: Answer this question thinking about the past year.-Variable type:binary</td></tr>
<tr><td>binary.6149_2.txt</td><td>1710174270056F5 forCTG.txt.gz</td><td>0.3005</td><td>0.08323</td><td>3.61</td><td>0.000306</td><td>0.005975</td><td>0.001617</td><td>1.007</td><td>0.009569</td><td>-0.006136</td><td>0.007658</td><td>Mouth/teeth dental problems: Painful gums</td><td>FALSE</td><td></td><td></td><td></td><td>359841</td><td>10115</td><td>349726</td><td>UK Biobank</td><td>https://docs.google.com/spreadsheets/d/1kvPoupSzsSFBNSztMzl04xMoSC3Kcx3CrjVf4yBmESU/edit?ts=5b5f17db#gid=227859291</td><td>PHESANT Transformation:6149_0 || CAT-MUL-BINARY-VAR 2 || NO_NAN Remove NA participants 512 || Removed 841 examples != 2 but with missing value (<0) || sample 349726/10115(359841) ||-Notes:ACE touchscreen question Do you have any of the following? (You can select more than one answer) The following checks were performed: If code -7 was selected, then no additional choices were allowed. If code -3 was selected, then no additional choices were allowed. If the participant activated the Help button they were shown the message: Answer this question thinking about the past year.-Variable type:binary</td></tr>
</table>

<table>
<tr><td>binary.6149_3.txt</td><td>1710174270056F5 forCTG.txt.gz</td><td>0.1538</td><td>0.04865</td><td>3.162</td><td>0.001567</td><td>0.02094</td><td>0.001981</td><td>1.021</td><td>0.01175</td><td>-0.008196</td><td>0.008654</td><td>Mouth/teeth dental problems: Bleeding gums</td><td>FALSE</td><td></td><td></td><td></td><td>359841</td><td>46694</td><td>313147</td><td>UK Biobank</td><td>https://docs.google.com/spreadsheets/d/1kvPoupSzsSFBNSztMzl04xMoSC3Kcx3CrjVf4yBmESU/edit?ts=5b5f17db#gid=227859291</td><td>PHESANT Transformation:6149_0 | | CAT-MUL-BINARY-VAR 3 | | NO_NAN Remove NA participants 512 | | Removed 841 examples != 3 but with missing value (<0) | | sample 313147/46694(359841) | | -Notes:ACE touchscreen question Do you have any of the following? (You can select more than one answer) The following checks were performed: If code -7 was selected, then no additional choices were allowed. If code -3 was selected, then no additional choices were allowed. If the participant activated the Help button they were shown the message: Answer this question thinking about the past year.-Variable type:binary</td></tr>
<tr><td>binary.6149_4.txt</td><td>1710174270056F5 forCTG.txt.gz</td><td>0.3771</td><td>0.0555</td><td>6.795</td><td>1.08E-11</td><td>0.01324</td><td>0.00204</td><td>1.012</td><td>0.01097</td><td>0.002339</td><td>0.008037</td><td>Mouth/teeth dental problems: Loose teeth</td><td>FALSE</td><td>Dental</td><td></td><td></td><td>359841</td><td>14364</td><td>345477</td><td>UK Biobank</td><td>https://docs.google.com/spreadsheets/d/1kvPoupSzsSFBNSztMzl04xMoSC3Kcx3CrjVf4yBmESU/edit?ts=5b5f17db#gid=227859291</td><td>PHESANT Transformation:6149_0 | | CAT-MUL-BINARY-VAR 4 | | NO_NAN Remove NA participants 512 | | Removed 841 examples != 4 but with missing value (<0) | | sample 345477/14364(359841) | | -Notes:ACE touchscreen question Do you have any of the following? (You can select more than one answer) The following checks were performed: If code -7 was selected, then no additional choices were allowed. If code -3 was selected, then no additional choices were allowed. If the participant activated the Help button they were shown the message: Answer this question thinking about the past year.-Variable type:binary</td></tr>
<tr><td>binary.6149_5.txt</td><td>1710174270056F5 forCTG.txt.gz</td><td>0.3317</td><td>0.06764</td><td>4.904</td><td>9.40E-07</td><td>0.008015</td><td>0.001726</td><td>0.999</td><td>0.01002</td><td>0.01622</td><td>0.007224</td><td>Mouth/teeth dental problems: Toothache</td><td>FALSE</td><td>Dental</td><td></td><td></td><td>359841</td><td>14477</td><td>345364</td><td>UK Biobank</td><td>https://docs.google.com/spreadsheets/d/1kvPoupSzsSFBNSztMzl04xMoSC3Kcx3CrjVf4yBmESU/edit?ts=5b5f17db#gid=227859291</td><td>PHESANT Transformation:6149_0 | | CAT-MUL-BINARY-VAR 5 | | NO_NAN Remove NA participants 512 | | Removed 841 examples != 5 but with missing value (<0) | | sample 345364/14477(359841) | | SKIP_val:-3 < 0 | | -Notes:ACE touchscreen question Do you have any of the following? (You can select more than one answer) The following checks were performed: If code -7 was selected, then no additional choices were allowed. If code -3 was selected, then no additional choices were allowed. If the participant activated the Help button they were shown the message: Answer this question thinking about the past year.-Variable type:binary</td></tr>
<tr><td>binary.6149_6.txt</td><td>1710174270056F5 forCTG.txt.gz</td><td>0.2244</td><td>0.03541</td><td>6.337</td><td>2.35E-10</td><td>0.05131</td><td>0.003256</td><td>1.04</td><td>0.0162</td><td>0.01533</td><td>0.009137</td><td>Mouth/teeth dental problems: Dentures</td><td>FALSE</td><td>Dental</td><td></td><td></td><td>359841</td><td>60977</td><td>298864</td><td>UK Biobank</td><td>https://docs.google.com/spreadsheets/d/1kvPoupSzsSFBNSztMzl04xMoSC3Kcx3CrjVf4yBmESU/edit?ts=5b5f17db#gid=227859291</td><td>PHESANT Transformation:6149_0 | | CAT-MUL-BINARY-VAR 6 | | NO_NAN Remove NA participants 512 | | Removed 841 examples != 6 but with missing value (<0) | | sample 298864/60977(359841) | | -Notes:ACE touchscreen question Do you have any of the following? (You can select more than one answer) The following checks were performed: If code -7 was selected, then no additional choices were allowed. If code -3 was selected, then no additional choices were allowed. If the participant activated the Help button they were shown the message: Answer this question thinking about the past year.-Variable type:binary</td></tr>
<tr><td>binary.6150_1.txt</td><td>1710174270056F5 forCTG.txt.gz</td><td>0.2132</td><td>0.04987</td><td>4.274</td><td>1.92E-05</td><td>0.01887</td><td>0.00215</td><td>1.014</td><td>0.01092</td><td>0.002098</td><td>0.008699</td><td>Vascular/heart problems diagnosed by doctor: Heart attack</td><td>FALSE</td><td>Cardiac</td><td></td><td></td><td>360420</td><td>8288</td><td>352132</td><td>UK Biobank</td><td>https://docs.google.com/spreadsheets/d/1kvPoupSzsSFBNSztMzl04xMoSC3Kcx3CrjVf4yBmESU/edit?ts=5b5f17db#gid=227859291</td><td>PHESANT Transformation:6150_0 | | CAT-MUL-BINARY-VAR 1 | | NO_NAN Remove NA participants 180 | | Removed 594 examples != 1 but with missing value (<0) | | sample 352132/8288(360420) | | -Notes:ACE touchscreen question Has a doctor ever told you that you have had any of the following conditions? (You can select more than one answer) The following checks were performed: If code -7 was selected, then no additional choices were allowed. If code -3 was selected, then no additional choices were allowed. If the participant activated the Help button they were shown the message: If you do not know if you have had any of the listed conditions, enter None of the above. You can check this with an interviewer later in the visit.-Variable type:binary</td></tr>
<tr><td>binary.6150_100.txt</td><td>1710174270056F5 forCTG.txt.gz</td><td>-0.1631</td><td>0.02989</td><td>-5.457</td><td>4.85E-08</td><td>0.1156</td><td>0.006161</td><td>1.105</td><td>0.02804</td><td>-0.000822</td><td>0.01109</td><td>Vascular/heart problems diagnosed by doctor: None of the above</td><td>FALSE</td><td>Cardiac</td><td></td><td></td><td>360420</td><td>253565</td><td>106855</td><td>UK Biobank</td><td>https://docs.google.com/spreadsheets/d/1kvPoupSzsSFBNSztMzl04xMoSC3Kcx3CrjVf4yBmESU/edit?ts=5b5f17db#gid=227859291</td><td>PHESANT Transformation:6150_0 | | CAT-MUL-BINARY-VAR 100 | | NO_NAN Remove NA participants 180 | | Removed 594 examples != 100 but with missing value (<0) | | sample 106855/253565(360420) | | -Notes:ACE touchscreen question Has a doctor ever told you that you have had any of the following conditions? (You can select more than one answer) The following checks were performed: If code -7 was selected, then no additional choices were allowed. If code -3 was selected, then no additional choices were allowed. If the participant activated the Help button they were shown the message: If you do not know if you have had any of the listed conditions, enter None of the above. You can check this with an interviewer later in the visit.-Variable type:binary</td></tr>
<tr><td>binary.6150_2.txt</td><td>1710174270056F5 forCTG.txt.gz</td><td>0.2929</td><td>0.04992</td><td>5.868</td><td>4.41E-09</td><td>0.02098</td><td>0.002481</td><td>1.038</td><td>0.01198</td><td>0.01019</td><td>0.009328</td><td>Vascular/heart problems diagnosed by doctor: Angina</td><td>FALSE</td><td>Cardiac</td><td></td><td></td><td>360420</td><td>11372</td><td>349048</td><td>UK Biobank</td><td>https://docs.google.com/spreadsheets/d/1kvPoupSzsSFBNSztMzl04xMoSC3Kcx3CrjVf4yBmESU/edit?ts=5b5f17db#gid=227859291</td><td>PHESANT Transformation:6150_0 | | CAT-MUL-BINARY-VAR 2 | | NO_NAN Remove NA participants 180 | | Removed 594 examples != 2 but with missing value (<0) | | sample 349048/11372(360420) | | -Notes:ACE touchscreen question Has a doctor ever told you that you have had any of the following conditions? (You can select more than one answer) The following checks were performed: If code -7 was selected, then no additional choices were allowed. If code -3 was selected, then no additional choices were allowed. If the participant activated the Help button they were shown the message: If you do not know if you have had any of the listed conditions, enter None of the above. You can check this with an interviewer later in the visit.-Variable type:binary</td></tr>
<tr><td>binary.6150_3.txt</td><td>1710174270056F5 forCTG.txt.gz</td><td>0.3756</td><td>0.1322</td><td>2.841</td><td>0.004498</td><td>0.003413</td><td>0.001406</td><td>1.009</td><td>0.008949</td><td>0.004926</td><td>0.008397</td><td>Vascular/heart problems diagnosed by doctor: Stroke</td><td>FALSE</td><td></td><td></td><td></td><td>360420</td><td>5587</td><td>354833</td><td>UK Biobank</td><td>https://docs.google.com/spreadsheets/d/1kvPoupSzsSFBNSztMzl04xMoSC3Kcx3CrjVf4yBmESU/edit?ts=5b5f17db#gid=227859291</td><td>PHESANT Transformation:6150_0 | | CAT-MUL-BINARY-VAR 3 | | NO_NAN Remove NA participants 180 | | Removed 594 examples != 3 but with missing value (<0) | | sample 354833/5587(360420) | | SKIP_val:-3 < 0 | | -Notes:ACE touchscreen question Has a doctor ever told you that you have had any of the following conditions? (You can select more than one answer) The following checks were performed: If code -7 was selected, then no additional choices were allowed. If code -3 was selected, then no additional choices were allowed. If the participant activated the Help button they were shown the message: If you do not know if you have had any of the listed conditions, enter None of the above. You can check this with an interviewer later in the visit.-Variable type:binary</td></tr>
<tr><td>binary.6150_4.txt</td><td>1710174270056F5 forCTG.txt.gz</td><td>0.1295</td><td>0.03069</td><td>4.22</td><td>2.44E-05</td><td>0.1165</td><td>0.006344</td><td>1.103</td><td>0.02959</td><td>-0.000976</td><td>0.01118</td><td>Vascular/heart problems diagnosed by doctor: High blood pressure</td><td>FALSE</td><td>Cardiac</td><td></td><td></td><td>360420</td><td>97139</td><td>263281</td><td>UK Biobank</td><td>https://docs.google.com/spreadsheets/d/1kvPoupSzsSFBNSztMzl04xMoSC3Kcx3CrjVf4yBmESU/edit?ts=5b5f17db#gid=227859291</td><td>PHESANT Transformation:6150_0 | | CAT-MUL-BINARY-VAR 4 | | NO_NAN Remove NA participants 180 | | Removed 594 examples != 4 but with missing value (<0) | | sample 263281/97139(360420) | | -Notes:ACE touchscreen question Has a doctor ever told you that you have had any of the following conditions? (You can select more than one answer) The following checks were performed: If code -7 was selected, then no additional choices were allowed. If code -3 was selected, then no additional choices were allowed. If the participant activated the Help button they were shown the message: If you do not know if you have had any of the listed conditions, enter None of the above. You can check this with an interviewer later in the visit.-Variable type:binary</td></tr>
<tr><td>binary.6151_1.txt</td><td>1710174270056F5 forCTG.txt.gz</td><td>0.1137</td><td>0.07832</td><td>1.451</td><td>0.1467</td><td>0.00562</td><td>0.001624</td><td>0.996</td><td>0.009486</td><td>0.01105</td><td>0.007768</td><td>Fractured bone site(s): Ankle</td><td>FALSE</td><td></td><td></td><td></td><td>359241</td><td>5068</td><td>354173</td><td>UK Biobank</td><td>https://docs.google.com/spreadsheets/d/1kvPoupSzsSFBNSztMzl04xMoSC3Kcx3CrjVf4yBmESU/edit?ts=5b5f17db#gid=227859291</td><td>PHESANT Transformation:6151_0 | | CAT-MUL-BINARY-VAR 1 | | Indicator name x2463_0_0 | | Remove indicator var NAs: 181 | | Remove indicator var <0: 1772 | | Removed 0 examples != 1 but with missing value (<0) | | sample 354173/5068(359241) | | -Notes:ACE touchscreen question Which bones did you fracture/break? (You can select more than one answer) The following checks were performed: If code -1 was selected, then no additional choices were allowed. If code -3 was selected, then no additional choices were allowed. ~F6151~ was collected from participants who indicated they have had fractured/broken bones in the last 5 years, as defined by their answers to ~F2463~-Variable type:binary</td></tr>
<tr><td>binary.6151_2.txt</td><td>1710174270056F5 forCTG.txt.gz</td><td>0.1046</td><td>0.1389</td><td>0.7533</td><td>0.4513</td><td>0.001831</td><td>0.001367</td><td>1.002</td><td>0.008258</td><td>0.01845</td><td>0.008146</td><td>Fractured bone site(s): Leg</td><td>FALSE</td><td></td><td></td><td></td><td>359241</td><td>2311</td><td>356930</td><td>UK Biobank</td><td>https://docs.google.com/spreadsheets/d/1kvPoupSzsSFBNSztMzl04xMoSC3Kcx3CrjVf4yBmESU/edit?ts=5b5f17db#gid=227859291</td><td>PHESANT Transformation:6151_0 | | CAT-MUL-BINARY-VAR 2 | | Indicator name x2463_0_0 | | Remove indicator var NAs: 181 | | Remove indicator var <0: 1772 | | Removed 0 examples != 2 but with missing value (<0) | | sample 356930/2311(359241) | | -Notes:ACE touchscreen question Which bones did you fracture/break? (You can select more than one answer) The following checks were performed: If code -1 was selected, then no additional choices were allowed. If code -3 was selected, then no additional choices were allowed. ~F6151~ was collected from participants who indicated they have had fractured/broken bones in the last 5 years, as defined by their answers to ~F2463~-Variable type:binary</td></tr>
<tr><td>binary.6151_5.txt</td><td>1710174270056F5 forCTG.txt.gz</td><td>0.07397</td><td>0.08435</td><td>0.877</td><td>0.3805</td><td>0.004861</td><td>0.001828</td><td>1.019</td><td>0.01034</td><td>0.009532</td><td>0.008253</td><td>Fractured bone site(s): Wrist</td><td>FALSE</td><td></td><td></td><td></td><td>359241</td><td>7200</td><td>352041</td><td>UK Biobank</td><td>https://docs.google.com/spreadsheets/d/1kvPoupSzsSFBNSztMzl04xMoSC3Kcx3CrjVf4yBmESU/edit?ts=5b5f17db#gid=227859291</td><td>PHESANT Transformation:6151_0 | | CAT-MUL-BINARY-VAR 5 | | Indicator name x2463_0_0 | | Remove indicator var NAs: 181 | | Remove indicator var <0: 1772 | | Removed 0 examples != 5 but with missing value (<0) | | sample 352041/7200(359241) | | -Notes:ACE touchscreen question Which bones did you fracture/break? (You can select more than one answer) The following checks were performed: If code -1 was selected, then no additional choices were allowed. If code -3 was selected, then no additional choices were allowed. ~F6151~ was collected from participants who indicated they have had fractured/broken bones in the last 5 years, as defined by their answers to ~F2463~-Variable type:binary</td></tr>
</table>

binary.6151_6.txt	1710174270056F5forCTG.txt.gz	-0.02085	0.08228	-0.2534	0.8	0.005816	0.001531	0.996	0.009542	0.02439	0.008507	Fractured bone site(s): Arm	FALSE					359241	3676	355565	UK Biobank	https://docs.google.com/spreadsheets/d/1kvPoupSzsSFBNSztMzl04xMoSC3Kcx3CrjVf4yBmESU/edit?ts=5b5f17db#gid=227859291	PHESANT Transformation:6151_0 || CAT-MUL-BINARY-VAR 6 || Indicator name x2463_0_0 || Remove indicator var NAs: 181 || Remove indicator var <0: 1772 || Removed 0 examples != 6 but with missing value (<0) || sample 355565/3676(359241) ||-Notes:ACE touchscreen question Which bones did you fracture/break? (You can select more than one answer) The following checks were performed: If code -1 was selected, then no additional choices were allowed. If code -3 was selected, then no additional choices were allowed. ~F6151~ was collected from participants who indicated they have had fractured/broken bones in the last 5 years, as defined by their answers to ~F2463~-Variable type:binary
binary.6151_7.txt	1710174270056F5forCTG.txt.gz	0.2956	0.07445	3.97	7.18E-05	0.008309	0.00194	1.005	0.01039	0.0119	0.007692	Fractured bone site(s): Other bones	FALSE					359241	18787	340454	UK Biobank	https://docs.google.com/spreadsheets/d/1kvPoupSzsSFBNSztMzl04xMoSC3Kcx3CrjVf4yBmESU/edit?ts=5b5f17db#gid=227859291	PHESANT Transformation:6151_0 || CAT-MUL-BINARY-VAR 7 || Indicator name x2463_0_0 || Remove indicator var NAs: 181 || Remove indicator var <0: 1772 || Removed 0 examples != 7 but with missing value (<0) || sample 340454/18787(359241) ||-Notes:ACE touchscreen question Which bones did you fracture/break? (You can select more than one answer) The following checks were performed: If code -1 was selected, then no additional choices were allowed. If code -3 was selected, then no additional choices were allowed. ~F6151~ was collected from participants who indicated they have had fractured/broken bones in the last 5 years, as defined by their answers to ~F2463~-Variable type:binary
binary.6152_100.txt	1710174270056F5forCTG.txt.gz	-0.04895	0.03282	-1.491	0.1358	0.07591	0.008734	1.031	0.03156	-0.01945	0.009847	Blood clot, DVT, bronchitis, emphysema, asthma, rhinitis, eczema, allergy diagnosed by doctor: None of the above	FALSE					360527	244522	116005	UK Biobank	https://docs.google.com/spreadsheets/d/1kvPoupSzsSFBNSztMzl04xMoSC3Kcx3CrjVf4yBmESU/edit?ts=5b5f17db#gid=227859291	PHESANT Transformation:6152_0 || CAT-MUL-BINARY-VAR 100 || NO_NAN Remove NA participants 181 || Removed 486 examples != 100 but with missing value (<0) || sample 116005/244522(360527) ||-Notes:ACE touchscreen question Has a doctor ever told you that you have had any of the following conditions? (You can select more than one answer) The following checks were performed: If code -7 was selected, then no additional choices were allowed. If code -3 was selected, then no additional choices were allowed. If the participant activated the Help button they were shown the message: If you do not know if you have had any of the listed conditions, enter None of the above. You can check this with an interviewer later in the visit.-Variable type:binary
binary.6152_5.txt	1710174270056F5forCTG.txt.gz	0.2801	0.08027	3.49	0.000483	0.008112	0.001882	1.027	0.0144	-0.000282	0.007921	Blood clot, DVT, bronchitis, emphysema, asthma, rhinitis, eczema, allergy diagnosed by doctor: Blood clot in the leg (DVT)	FALSE					360527	7386	353141	UK Biobank	https://docs.google.com/spreadsheets/d/1kvPoupSzsSFBNSztMzl04xMoSC3Kcx3CrjVf4yBmESU/edit?ts=5b5f17db#gid=227859291	PHESANT Transformation:6152_0 || CAT-MUL-BINARY-VAR 5 || NO_NAN Remove NA participants 181 || Removed 486 examples != 5 but with missing value (<0) || sample 353141/7386(360527) || SKIP_val:-3 < 0 ||-Notes:ACE touchscreen question Has a doctor ever told you that you have had any of the following conditions? (You can select more than one answer) The following checks were performed: If code -7 was selected, then no additional choices were allowed. If code -3 was selected, then no additional choices were allowed. If the participant activated the Help button they were shown the message: If you do not know if you have had any of the listed conditions, enter None of the above. You can check this with an interviewer later in the visit.-Variable type:binary
binary.6152_6.txt	1710174270056F5forCTG.txt.gz	0.329	0.05341	6.16	7.27E-10	0.01402	0.001993	0.976	0.0108	0.01288	0.007894	Blood clot, DVT, bronchitis, emphysema, asthma, rhinitis, eczema, allergy diagnosed by doctor: Emphysema/chronic bronchitis	FALSE	Pulmonary				360527	6138	354389	UK Biobank	https://docs.google.com/spreadsheets/d/1kvPoupSzsSFBNSztMzl04xMoSC3Kcx3CrjVf4yBmESU/edit?ts=5b5f17db#gid=227859291	PHESANT Transformation:6152_0 || CAT-MUL-BINARY-VAR 6 || NO_NAN Remove NA participants 181 || Removed 486 examples != 6 but with missing value (<0) || sample 354389/6138(360527) ||-Notes:ACE touchscreen question Has a doctor ever told you that you have had any of the following conditions? (You can select more than one answer) The following checks were performed: If code -7 was selected, then no additional choices were allowed. If code -3 was selected, then no additional choices were allowed. If the participant activated the Help button they were shown the message: If you do not know if you have had any of the listed conditions, enter None of the above. You can check this with an interviewer later in the visit.-Variable type:binary
binary.6152_7.txt	1710174270056F5forCTG.txt.gz	0.3715	0.08936	4.157	3.23E-05	0.005423	0.001644	1.014	0.01032	-0.01539	0.007567	Blood clot, DVT, bronchitis, emphysema, asthma, rhinitis, eczema, allergy diagnosed by doctor: Blood clot in the lung	FALSE	Pulmonary				360527	2984	357543	UK Biobank	https://docs.google.com/spreadsheets/d/1kvPoupSzsSFBNSztMzl04xMoSC3Kcx3CrjVf4yBmESU/edit?ts=5b5f17db#gid=227859291	PHESANT Transformation:6152_0 || CAT-MUL-BINARY-VAR 7 || NO_NAN Remove NA participants 181 || Removed 486 examples != 7 but with missing value (<0) || sample 357543/2984(360527) ||-Notes:ACE touchscreen question Has a doctor ever told you that you have had any of the following conditions? (You can select more than one answer) The following checks were performed: If code -7 was selected, then no additional choices were allowed. If code -3 was selected, then no additional choices were allowed. If the participant activated the Help button they were shown the message: If you do not know if you have had any of the listed conditions, enter None of the above. You can check this with an interviewer later in the visit.-Variable type:binary
binary.6152_8.txt	1710174270056F5forCTG.txt.gz	0.1218	0.03491	3.489	0.000485	0.05701	0.007339	1.017	0.02746	0.02068	0.009352	Blood clot, DVT, bronchitis, emphysema, asthma, rhinitis, eczema, allergy diagnosed by doctor: Asthma	FALSE					360527	41633	318894	UK Biobank	https://docs.google.com/spreadsheets/d/1kvPoupSzsSFBNSztMzl04xMoSC3Kcx3CrjVf4yBmESU/edit?ts=5b5f17db#gid=227859291	PHESANT Transformation:6152_0 || CAT-MUL-BINARY-VAR 8 || NO_NAN Remove NA participants 181 || Removed 486 examples != 8 but with missing value (<0) || sample 318894/41633(360527) ||-Notes:ACE touchscreen question Has a doctor ever told you that you have had any of the following conditions? (You can select more than one answer) The following checks were performed: If code -7 was selected, then no additional choices were allowed. If code -3 was selected, then no additional choices were allowed. If the participant activated the Help button they were shown the message: If you do not know if you have had any of the listed conditions, enter None of the above. You can check this with an interviewer later in the visit.-Variable type:binary
binary.6152_9.txt	1710174270056F5forCTG.txt.gz	-0.06499	0.02984	-2.178	0.02941	0.07157	0.007152	1.037	0.02733	0.01921	0.009121	Blood clot, DVT, bronchitis, emphysema, asthma, rhinitis, eczema, allergy diagnosed by doctor: Hayfever, allergic rhinitis or eczema	FALSE					360527	83407	277120	UK Biobank	https://docs.google.com/spreadsheets/d/1kvPoupSzsSFBNSztMzl04xMoSC3Kcx3CrjVf4yBmESU/edit?ts=5b5f17db#gid=227859291	PHESANT Transformation:6152_0 || CAT-MUL-BINARY-VAR 9 || NO_NAN Remove NA participants 181 || Removed 486 examples != 9 but with missing value (<0) || sample 277120/83407(360527) ||-Notes:ACE touchscreen question Has a doctor ever told you that you have had any of the following conditions? (You can select more than one answer) The following checks were performed: If code -7 was selected, then no additional choices were allowed. If code -3 was selected, then no additional choices were allowed. If the participant activated the Help button they were shown the message: If you do not know if you have had any of the listed conditions, enter None of the above. You can check this with an interviewer later in the visit.-Variable type:binary
binary.6153_1.txt	1710174270056F5forCTG.txt.gz	0.2383	0.04943	4.822	1.42E-06	0.04811	0.008931	1.051	0.03369	0.0012	0.008417	Medication for cholesterol, blood pressure, diabetes, or take exogenous hormones: Cholesterol lowering medication	FALSE	Metabolic				193148	24247	168901	UK Biobank	https://docs.google.com/spreadsheets/d/1kvPoupSzsSFBNSztMzl04xMoSC3Kcx3CrjVf4yBmESU/edit?ts=5b5f17db#gid=227859291	PHESANT Transformation:6153_0 || CAT-MUL-BINARY-VAR 1 || NO_NAN Remove NA participants 167294 || Removed 752 examples != 1 but with missing value (<0) || sample 168901/24247(193148) ||-Notes:ACE touchscreen question Do you regularly take any of the following medications? (You can select more than one answer) The following checks were performed: If code -7 was selected, then no additional choices were allowed. If code -1 was selected, then no additional choices were allowed. If code -3 was selected, then no additional choices were allowed. If the participant activated the Help button they were shown the message: If you are not sure if you take any of the types of medications, enter Do not know. You will be asked to provide all of the medications that you take later in the visit.-Variable type:binary
binary.6153_100.txt	1710174270056F5forCTG.txt.gz	-0.2314	0.04197	-5.512	3.54E-08	0.06504	0.005409	1.035	0.01503	0.001617	0.009052	Medication for cholesterol, blood pressure, diabetes, or take exogenous hormones: None of the above	FALSE	Metabolic				193148	133338	59810	UK Biobank	https://docs.google.com/spreadsheets/d/1kvPoupSzsSFBNSztMzl04xMoSC3Kcx3CrjVf4yBmESU/edit?ts=5b5f17db#gid=227859291	PHESANT Transformation:6153_0 || CAT-MUL-BINARY-VAR 100 || NO_NAN Remove NA participants 167294 || Removed 752 examples != 100 but with missing value (<0) || sample 59810/133338(193148) ||-Notes:ACE touchscreen question Do you regularly take any of the following medications? (You can select more than one answer) The following checks were performed: If code -7 was selected, then no additional choices were allowed. If code -1 was selected, then no additional choices were allowed. If code -3 was selected, then no additional choices were allowed. If the participant activated the Help button they were shown the message: If you are not sure if you take any of the types of medications, enter Do not know. You will be asked to provide all of the medications that you take later in the visit.-Variable type:binary
binary.6153_2.txt	1710174270056F5forCTG.txt.gz	0.1091	0.03668	2.975	0.002927	0.1055	0.00779	1.059	0.01902	0.004468	0.009933	Medication for cholesterol, blood pressure, diabetes, or take exogenous hormones: Blood pressure medication	FALSE					193148	33519	159629	UK Biobank	https://docs.google.com/spreadsheets/d/1kvPoupSzsSFBNSztMzl04xMoSC3Kcx3CrjVf4yBmESU/edit?ts=5b5f17db#gid=227859291	PHESANT Transformation:6153_0 || CAT-MUL-BINARY-VAR 2 || NO_NAN Remove NA participants 167294 || Removed 752 examples != 2 but with missing value (<0) || sample 159629/33519(193148) ||-Notes:ACE touchscreen question Do you regularly take any of the following medications? (You can select more than one answer) The following checks were performed: If code -7 was selected, then no additional choices were allowed. If code -1 was selected, then no additional choices were allowed. If code -3 was selected, then no additional choices were allowed. If the participant activated the Help button they were shown the message: If you are not sure if you take any of the types of medications, enter Do not know. You will be asked to provide all of the medications that you take later in the visit.-Variable type:binary

binary.6153_3.txt	1710174270056F5 forCTG.txt.gz	0.09798	0.08229	1.191	0.2338	0.008197	0.003087	1.001	0.009722	0.001174	0.007289	Medication for cholesterol, blood pressure, diabetes, or take exogenous hormones: Insulin	FALSE				193148	1476	191672	UK Biobank	https://docs.google.com/spreadsheets/d/1kvPoupSzsSFBNSztMzl04xMoSC3Kcx3CrjVf4yBmESU/edit?ts=5b5f17db#gid=227859291	PHESANT Transformation:6153_0 | | CAT-MUL-BINARY-VAR 3 | | NO_NAN Remove NA participants 167294 | | Removed 752 examples != 3 but with missing value (<0) | | sample 191672/1476(193148) | | SKIP_val:-1 < 0 | | SKIP_val:-3 < 0 | |-Notes:ACE touchscreen question Do you regularly take any of the following medications? (You can select more than one answer) The following checks were performed: If code -7 was selected, then no additional choices were allowed. If code -1 was selected, then no additional choices were allowed. If code -3 was selected, then no additional choices were allowed. If the participant activated the Help button they were shown the message: If you are not sure if you take any of the types of medications, enter Do not know. You will be asked to provide all of the medications that you take later in the visit.-Variable type:binary
binary.6153_4.txt	1710174270056F5 forCTG.txt.gz	0.4688	0.07229	6.486	8.84E-11	0.01523	0.00318	1.003	0.01019	-0.01116	0.008765	Medication for cholesterol, blood pressure, diabetes, or take exogenous hormones: Hormone replacement therapy	FALSE	Reproductive			193148	14385	178763	UK Biobank	https://docs.google.com/spreadsheets/d/1kvPoupSzsSFBNSztMzl04xMoSC3Kcx3CrjVf4yBmESU/edit?ts=5b5f17db#gid=227859291	PHESANT Transformation:6153_0 | | CAT-MUL-BINARY-VAR 4 | | NO_NAN Remove NA participants 167294 | | Removed 752 examples != 4 but with missing value (<0) | | sample 178763/14385(193148) | |-Notes:ACE touchscreen question Do you regularly take any of the following medications? (You can select more than one answer) The following checks were performed: If code -7 was selected, then no additional choices were allowed. If code -1 was selected, then no additional choices were allowed. If code -3 was selected, then no additional choices were allowed. If the participant activated the Help button they were shown the message: If you are not sure if you take any of the types of medications, enter Do not know. You will be asked to provide all of the medications that you take later in the visit.-Variable type:binary
binary.6153_5.txt	1710174270056F5 forCTG.txt.gz	-0.2204	0.0915	-2.408	0.01603	0.00831	0.00289	0.979	0.009133	-0.001311	0.007114	Medication for cholesterol, blood pressure, diabetes, or take exogenous hormones: Oral contraceptive pill or minipill	FALSE				193148	5042	188106	UK Biobank	https://docs.google.com/spreadsheets/d/1kvPoupSzsSFBNSztMzl04xMoSC3Kcx3CrjVf4yBmESU/edit?ts=5b5f17db#gid=227859291	PHESANT Transformation:6153_0 | | CAT-MUL-BINARY-VAR 5 | | NO_NAN Remove NA participants 167294 | | Removed 752 examples != 5 but with missing value (<0) | | sample 188106/5042(193148) | |-Notes:ACE touchscreen question Do you regularly take any of the following medications? (You can select more than one answer) The following checks were performed: If code -7 was selected, then no additional choices were allowed. If code -1 was selected, then no additional choices were allowed. If code -3 was selected, then no additional choices were allowed. If the participant activated the Help button they were shown the message: If you are not sure if you take any of the types of medications, enter Do not know. You will be asked to provide all of the medications that you take later in the visit.-Variable type:binary
binary.6154_1.txt	1710174270056F5 forCTG.txt.gz	0.2566	0.04679	5.484	4.16E-08	0.02309	0.002349	1.014	0.01186	0.0118	0.009122	Medication for pain relief, constipation, heartburn: Aspirin	FALSE	Pain			357084	50204	306880	UK Biobank	https://docs.google.com/spreadsheets/d/1kvPoupSzsSFBNSztMzl04xMoSC3Kcx3CrjVf4yBmESU/edit?ts=5b5f17db#gid=227859291	PHESANT Transformation:6154_0 | | CAT-MUL-BINARY-VAR 1 | | NO_NAN Remove NA participants 514 | | Removed 3596 examples != 1 but with missing value (<0) | | sample 306880/50204(357084) | |-Notes:ACE touchscreen question Do you regularly take any of the following? (You can select more than one answer) The following checks were performed: If code -7 was selected, then no additional choices were allowed. If code -1 was selected, then no additional choices were allowed. If code -3 was selected, then no additional choices were allowed. If the participant activated the Help button they were shown the message: Some over the counter medicines are known by other names. Please enter the corresponding name if you take any of the following REGULARLY (that is, most days of the week for the last 4 weeks): Aspirin: Alka Rapid Crystals, Alka-Seltzer XS, Anadin Extra, Anadin Original, Askit powders, Aspro Clear, Codis 500, Disprin, Disprin Extra Ibuprofen: Anadin Ultra, Anadin Ibuprofen, Cuprofen Plus, Nurofen, Solpaflex, Ibuleve Paracetamol: Anadin Extra, Hedex Extra, Panadol, Paracodol, Paramol, Solpadeine, Syndol, Veganin, Feminax, Midrid, Migraleve Codeine: Codis 500, Cuprofen Plus, Nurofen Plus, Panadol Ultra, Paracodol, Paramol, Solpadeine Max, Sopadeine Plus, Solpafelx, Syndol, Veganin, Feminax, Migraleve-Variable type:binary
binary.6154_100.txt	1710174270056F5 forCTG.txt.gz	-0.3337	0.03151	-10.59	3.34E-26	0.05098	0.002954	1.046	0.01341	-0.0179	0.009153	Medication for pain relief, constipation, heartburn: None of the above	FALSE	Pain			357084	198043	159041	UK Biobank	https://docs.google.com/spreadsheets/d/1kvPoupSzsSFBNSztMzl04xMoSC3Kcx3CrjVf4yBmESU/edit?ts=5b5f17db#gid=227859291	PHESANT Transformation:6154_0 | | CAT-MUL-BINARY-VAR 100 | | NO_NAN Remove NA participants 514 | | Removed 3596 examples != 100 but with missing value (<0) | | sample 159041/198043(357084) | |-Notes:ACE touchscreen question Do you regularly take any of the following? (You can select more than one answer) The following checks were performed: If code -7 was selected, then no additional choices were allowed. If code -1 was selected, then no additional choices were allowed. If code -3 was selected, then no additional choices were allowed. If the participant activated the Help button they were shown the message: Some over the counter medicines are known by other names. Please enter the corresponding name if you take any of the following REGULARLY (that is, most days of the week for the last 4 weeks): Aspirin: Alka Rapid Crystals, Alka-Seltzer XS, Anadin Extra, Anadin Original, Askit powders, Aspro Clear, Codis 500, Disprin, Disprin Extra Ibuprofen: Anadin Ultra, Anadin Ibuprofen, Cuprofen Plus, Nurofen, Solpaflex, Ibuleve Paracetamol: Anadin Extra, Hedex Extra, Panadol, Paracodol, Paramol, Solpadeine, Syndol, Veganin, Feminax, Midrid, Migraleve Codeine: Codis 500, Cuprofen Plus, Nurofen Plus, Panadol Ultra, Paracodol, Paramol, Solpadeine Max, Sopadeine Plus, Solpafelx, Syndol, Veganin, Feminax, Migraleve-Variable type:binary
binary.6154_2.txt	1710174270056F5 forCTG.txt.gz	0.2338	0.04705	4.97	6.70E-07	0.01962	0.001903	1.007	0.01001	0.009492	0.008281	Medication for pain relief, constipation, heartburn: Ibuprofen (e.g. Nurofen)	FALSE	Pain			357084	53049	304035	UK Biobank	https://docs.google.com/spreadsheets/d/1kvPoupSzsSFBNSztMzl04xMoSC3Kcx3CrjVf4yBmESU/edit?ts=5b5f17db#gid=227859291	PHESANT Transformation:6154_0 | | CAT-MUL-BINARY-VAR 2 | | NO_NAN Remove NA participants 514 | | Removed 3596 examples != 2 but with missing value (<0) | | sample 304035/53049(357084) | |-Notes:ACE touchscreen question Do you regularly take any of the following? (You can select more than one answer) The following checks were performed: If code -7 was selected, then no additional choices were allowed. If code -1 was selected, then no additional choices were allowed. If code -3 was selected, then no additional choices were allowed. If the participant activated the Help button they were shown the message: Some over the counter medicines are known by other names. Please enter the corresponding name if you take any of the following REGULARLY (that is, most days of the week for the last 4 weeks): Aspirin: Alka Rapid Crystals, Alka-Seltzer XS, Anadin Extra, Anadin Original, Askit powders, Aspro Clear, Codis 500, Disprin, Disprin Extra Ibuprofen: Anadin Ultra, Anadin Ibuprofen, Cuprofen Plus, Nurofen, Solpaflex, Ibuleve Paracetamol: Anadin Extra, Hedex Extra, Panadol, Paracodol, Paramol, Solpadeine, Syndol, Veganin, Feminax, Midrid, Migraleve Codeine: Codis 500, Cuprofen Plus, Nurofen Plus, Panadol Ultra, Paracodol, Paramol, Solpadeine Max, Sopadeine Plus, Solpafelx, Syndol, Veganin, Feminax, Migraleve-Variable type:binary
binary.6154_3.txt	1710174270056F5 forCTG.txt.gz	0.2583	0.03559	7.258	3.93E-13	0.03928	0.002459	1.028	0.01096	0.01495	0.008564	Medication for pain relief, constipation, heartburn: Paracetamol	FALSE	Pain			357084	77936	279148	UK Biobank	https://docs.google.com/spreadsheets/d/1kvPoupSzsSFBNSztMzl04xMoSC3Kcx3CrjVf4yBmESU/edit?ts=5b5f17db#gid=227859291	PHESANT Transformation:6154_0 | | CAT-MUL-BINARY-VAR 3 | | NO_NAN Remove NA participants 514 | | Removed 3596 examples != 3 but with missing value (<0) | | sample 279148/77936(357084) | |-Notes:ACE touchscreen question Do you regularly take any of the following? (You can select more than one answer) The following checks were performed: If code -7 was selected, then no additional choices were allowed. If code -1 was selected, then no additional choices were allowed. If code -3 was selected, then no additional choices were allowed. If the participant activated the Help button they were shown the message: Some over the counter medicines are known by other names. Please enter the corresponding name if you take any of the following REGULARLY (that is, most days of the week for the last 4 weeks): Aspirin: Alka Rapid Crystals, Alka-Seltzer XS, Anadin Extra, Anadin Original, Askit powders, Aspro Clear, Codis 500, Disprin, Disprin Extra Ibuprofen: Anadin Ultra, Anadin Ibuprofen, Cuprofen Plus, Nurofen, Solpaflex, Ibuleve Paracetamol: Anadin Extra, Hedex Extra, Panadol, Paracodol, Paramol, Solpadeine, Syndol, Veganin, Feminax, Midrid, Migraleve Codeine: Codis 500, Cuprofen Plus, Nurofen Plus, Panadol Ultra, Paracodol, Paramol, Solpadeine Max, Sopadeine Plus, Solpafelx, Syndol, Veganin, Feminax, Migraleve-Variable type:binary

<table>
<tr><td>binary.6154_4.txt</td><td>1710174270056F5forCTG.txt.gz</td><td>0.3923</td><td>0.1067</td><td>3.679</td><td>0.000235</td><td>0.006267</td><td>0.001673</td><td>0.995</td><td>0.0104</td><td>0.0007527</td><td>0.008097</td><td>Medication for pain relief, constipation, heartburn: Ranitidine (e.g. Zantac)</td><td>FALSE</td><td></td><td></td><td></td><td>357084</td><td>6826</td><td>350258</td><td>UK Biobank</td><td>https://docs.google.com/spreadsheets/d/1kvPoupSzsSFBNSztMzl04xMoSC3Kcx3CrjVf4yBmESU/edit?ts=5b5f17db#gid=227859291</td><td>PHESANT Transformation:6154_0 || CAT-MUL-BINARY-VAR 4 || NO_NAN Remove NA participants 514 || Removed 3596 examples != 4 but with missing value (<0) || sample 350258/6826(357084) ||-Notes:ACE touchscreen question Do you regularly take any of the following? (You can select more than one answer) The following checks were performed: If code -7 was selected, then no additional choices were allowed. If code -1 was selected, then no additional choices were allowed. If code -3 was selected, then no additional choices were allowed. If the participant activated the Help button they were shown the message: Some over the counter medicines are known by other names. Please enter the corresponding name if you take any of the following REGULARLY (that is, most days of the week for the last 4 weeks): Aspirin: Alka Rapid Crystals, Alka-Seltzer XS, Anadin Extra, Anadin Original, Askit powders, Aspro Clear, Codis 500, Disprin, Disprin Extra Ibuprofen: Anadin Ultra, Anadin Ibuprofen, Cuprofen Plus, Nurofen, Solpaflex, Ibuleve Paracetamol: Anadin Extra, Hedex Extra, Panadol, Paracodol, Paramol, Solpadeine, Syndol, Veganin, Feminax, Midrid, Migraleve Codeine: Codis 500, Cuprofen Plus, Nurofen Plus, Panadol Ultra, Paracodol, Paramol, Solpadeine Max, Sopadeine Plus, Solpafelx, Syndol, Veganin, Feminax, Migraleve-Variable type:binary</td></tr>
<tr><td>binary.6154_5.txt</td><td>1710174270056F5forCTG.txt.gz</td><td>0.3113</td><td>0.04949</td><td>6.29</td><td>3.18E-10</td><td>0.01806</td><td>0.001712</td><td>1.025</td><td>0.009915</td><td>0.002381</td><td>0.008448</td><td>Medication for pain relief, constipation, heartburn: Omeprazole (e.g. Zanprol)</td><td>FALSE</td><td>Pain</td><td></td><td></td><td>357084</td><td>21464</td><td>335620</td><td>UK Biobank</td><td>https://docs.google.com/spreadsheets/d/1kvPoupSzsSFBNSztMzl04xMoSC3Kcx3CrjVf4yBmESU/edit?ts=5b5f17db#gid=227859291</td><td>PHESANT Transformation:6154_0 || CAT-MUL-BINARY-VAR 5 || NO_NAN Remove NA participants 514 || Removed 3596 examples != 5 but with missing value (<0) || sample 335620/21464(357084) ||-Notes:ACE touchscreen question Do you regularly take any of the following? (You can select more than one answer) The following checks were performed: If code -7 was selected, then no additional choices were allowed. If code -1 was selected, then no additional choices were allowed. If code -3 was selected, then no additional choices were allowed. If the participant activated the Help button they were shown the message: Some over the counter medicines are known by other names. Please enter the corresponding name if you take any of the following REGULARLY (that is, most days of the week for the last 4 weeks): Aspirin: Alka Rapid Crystals, Alka-Seltzer XS, Anadin Extra, Anadin Original, Askit powders, Aspro Clear, Codis 500, Disprin, Disprin Extra Ibuprofen: Anadin Ultra, Anadin Ibuprofen, Cuprofen Plus, Nurofen, Solpaflex, Ibuleve Paracetamol: Anadin Extra, Hedex Extra, Panadol, Paracodol, Paramol, Solpadeine, Syndol, Veganin, Feminax, Midrid, Migraleve Codeine: Codis 500, Cuprofen Plus, Nurofen Plus, Panadol Ultra, Paracodol, Paramol, Solpadeine Max, Sopadeine Plus, Solpafelx, Syndol, Veganin, Feminax, Migraleve-Variable type:binary</td></tr>
<tr><td>binary.6154_6.txt</td><td>1710174270056F5forCTG.txt.gz</td><td>0.3561</td><td>0.0635</td><td>5.607</td><td>2.06E-08</td><td>0.01028</td><td>0.001682</td><td>1.011</td><td>0.009042</td><td>0.001105</td><td>0.007942</td><td>Medication for pain relief, constipation, heartburn: Laxatives (e.g. Dulcolax, Senokot)</td><td>FALSE</td><td>Pain</td><td></td><td></td><td>357084</td><td>10307</td><td>346777</td><td>UK Biobank</td><td>https://docs.google.com/spreadsheets/d/1kvPoupSzsSFBNSztMzl04xMoSC3Kcx3CrjVf4yBmESU/edit?ts=5b5f17db#gid=227859291</td><td>PHESANT Transformation:6154_0 || CAT-MUL-BINARY-VAR 6 || NO_NAN Remove NA participants 514 || Removed 3596 examples != 6 but with missing value (<0) || sample 346777/10307(357084) || SKIP_val:-1 < 0 || SKIP_val:-3 < 0 ||-Notes:ACE touchscreen question Do you regularly take any of the following? (You can select more than one answer) The following checks were performed: If code -7 was selected, then no additional choices were allowed. If code -1 was selected, then no additional choices were allowed. If code -3 was selected, then no additional choices were allowed. If the participant activated the Help button they were shown the message: Some over the counter medicines are known by other names. Please enter the corresponding name if you take any of the following REGULARLY (that is, most days of the week for the last 4 weeks): Aspirin: Alka Rapid Crystals, Alka-Seltzer XS, Anadin Extra, Anadin Original, Askit powders, Aspro Clear, Codis 500, Disprin, Disprin Extra Ibuprofen: Anadin Ultra, Anadin Ibuprofen, Cuprofen Plus, Nurofen, Solpaflex, Ibuleve Paracetamol: Anadin Extra, Hedex Extra, Panadol, Paracodol, Paramol, Solpadeine, Syndol, Veganin, Feminax, Midrid, Migraleve Codeine: Codis 500, Cuprofen Plus, Nurofen Plus, Panadol Ultra, Paracodol, Paramol, Solpadeine Max, Sopadeine Plus, Solpafelx, Syndol, Veganin, Feminax, Migraleve-Variable type:binary</td></tr>
<tr><td>binary.6155_1.txt</td><td>1710174270056F5forCTG.txt.gz</td><td>0.1508</td><td>0.09849</td><td>1.531</td><td>0.1258</td><td>0.003773</td><td>0.001633</td><td>1.003</td><td>0.009655</td><td>0.01305</td><td>0.00853</td><td>Vitamin and mineral supplements: Vitamin A</td><td>FALSE</td><td></td><td></td><td></td><td>359245</td><td>6735</td><td>352510</td><td>UK Biobank</td><td>https://docs.google.com/spreadsheets/d/1kvPoupSzsSFBNSztMzl04xMoSC3Kcx3CrjVf4yBmESU/edit?ts=5b5f17db#gid=227859291</td><td>PHESANT Transformation:6155_0 || CAT-MUL-BINARY-VAR 1 || NO_NAN Remove NA participants 514 || Removed 1435 examples != 1 but with missing value (<0) || sample 352510/6735(359245) || SKIP_val:-3 < 0 ||-Notes:ACE touchscreen question Do you regularly take any of the following? (You can select more than one answer) The following checks were performed: If code -7 was selected, then no additional choices were allowed. If code -3 was selected, then no additional choices were allowed.-Variable type:binary</td></tr>
<tr><td>binary.6155_100.txt</td><td>1710174270056F5forCTG.txt.gz</td><td>-0.05308</td><td>0.04267</td><td>-1.244</td><td>0.2135</td><td>0.0294</td><td>0.002161</td><td>0.999</td><td>0.01041</td><td>-0.008158</td><td>0.008892</td><td>Vitamin and mineral supplements: None of the above</td><td>FALSE</td><td></td><td></td><td></td><td>359245</td><td>246781</td><td>112464</td><td>UK Biobank</td><td>https://docs.google.com/spreadsheets/d/1kvPoupSzsSFBNSztMzl04xMoSC3Kcx3CrjVf4yBmESU/edit?ts=5b5f17db#gid=227859291</td><td>PHESANT Transformation:6155_0 || CAT-MUL-BINARY-VAR 100 || NO_NAN Remove NA participants 514 || Removed 1435 examples != 100 but with missing value (<0) || sample 112464/246781(359245) ||-Notes:ACE touchscreen question Do you regularly take any of the following? (You can select more than one answer) The following checks were performed: If code -7 was selected, then no additional choices were allowed. If code -3 was selected, then no additional choices were allowed.-Variable type:binary</td></tr>
<tr><td>binary.6155_2.txt</td><td>1710174270056F5forCTG.txt.gz</td><td>0.2166</td><td>0.0706</td><td>3.069</td><td>0.002149</td><td>0.007569</td><td>0.001628</td><td>1.002</td><td>0.009361</td><td>0.005869</td><td>0.008226</td><td>Vitamin and mineral supplements: Vitamin B</td><td>FALSE</td><td></td><td></td><td></td><td>359245</td><td>14792</td><td>344453</td><td>UK Biobank</td><td>https://docs.google.com/spreadsheets/d/1kvPoupSzsSFBNSztMzl04xMoSC3Kcx3CrjVf4yBmESU/edit?ts=5b5f17db#gid=227859291</td><td>PHESANT Transformation:6155_0 || CAT-MUL-BINARY-VAR 2 || NO_NAN Remove NA participants 514 || Removed 1435 examples != 2 but with missing value (<0) || sample 344453/14792(359245) ||-Notes:ACE touchscreen question Do you regularly take any of the following? (You can select more than one answer) The following checks were performed: If code -7 was selected, then no additional choices were allowed. If code -3 was selected, then no additional choices were allowed.-Variable type:binary</td></tr>
<tr><td>binary.6155_3.txt</td><td>1710174270056F5forCTG.txt.gz</td><td>0.03171</td><td>0.0562</td><td>0.5642</td><td>0.5726</td><td>0.01365</td><td>0.001778</td><td>0.989</td><td>0.01018</td><td>0.002961</td><td>0.008506</td><td>Vitamin and mineral supplements: Vitamin C</td><td>FALSE</td><td></td><td></td><td></td><td>359245</td><td>30950</td><td>328295</td><td>UK Biobank</td><td>https://docs.google.com/spreadsheets/d/1kvPoupSzsSFBNSztMzl04xMoSC3Kcx3CrjVf4yBmESU/edit?ts=5b5f17db#gid=227859291</td><td>PHESANT Transformation:6155_0 || CAT-MUL-BINARY-VAR 3 || NO_NAN Remove NA participants 514 || Removed 1435 examples != 3 but with missing value (<0) || sample 328295/30950(359245) ||-Notes:ACE touchscreen question Do you regularly take any of the following? (You can select more than one answer) The following checks were performed: If code -7 was selected, then no additional choices were allowed. If code -3 was selected, then no additional choices were allowed.-Variable type:binary</td></tr>
<tr><td>binary.6155_4.txt</td><td>1710174270056F5forCTG.txt.gz</td><td>-0.01297</td><td>0.08212</td><td>-0.1579</td><td>0.8745</td><td>0.004777</td><td>0.001677</td><td>1.005</td><td>0.009647</td><td>0.002674</td><td>0.007469</td><td>Vitamin and mineral supplements: Vitamin D</td><td>FALSE</td><td></td><td></td><td></td><td>359245</td><td>13687</td><td>345558</td><td>UK Biobank</td><td>https://docs.google.com/spreadsheets/d/1kvPoupSzsSFBNSztMzl04xMoSC3Kcx3CrjVf4yBmESU/edit?ts=5b5f17db#gid=227859291</td><td>PHESANT Transformation:6155_0 || CAT-MUL-BINARY-VAR 4 || NO_NAN Remove NA participants 514 || Removed 1435 examples != 4 but with missing value (<0) || sample 345558/13687(359245) ||-Notes:ACE touchscreen question Do you regularly take any of the following? (You can select more than one answer) The following checks were performed: If code -7 was selected, then no additional choices were allowed. If code -3 was selected, then no additional choices were allowed.-Variable type:binary</td></tr>
<tr><td>binary.6155_5.txt</td><td>1710174270056F5forCTG.txt.gz</td><td>0.1704</td><td>0.07748</td><td>2.2</td><td>0.02784</td><td>0.006853</td><td>0.001733</td><td>0.996</td><td>0.01029</td><td>0.006747</td><td>0.00833</td><td>Vitamin and mineral supplements: Vitamin E</td><td>FALSE</td><td></td><td></td><td></td><td>359245</td><td>10356</td><td>348889</td><td>UK Biobank</td><td>https://docs.google.com/spreadsheets/d/1kvPoupSzsSFBNSztMzl04xMoSC3Kcx3CrjVf4yBmESU/edit?ts=5b5f17db#gid=227859291</td><td>PHESANT Transformation:6155_0 || CAT-MUL-BINARY-VAR 5 || NO_NAN Remove NA participants 514 || Removed 1435 examples != 5 but with missing value (<0) || sample 348889/10356(359245) ||-Notes:ACE touchscreen question Do you regularly take any of the following? (You can select more than one answer) The following checks were performed: If code -7 was selected, then no additional choices were allowed. If code -3 was selected, then no additional choices were allowed.-Variable type:binary</td></tr>
<tr><td>binary.6155_7.txt</td><td>1710174270056F5forCTG.txt.gz</td><td>0.03496</td><td>0.04189</td><td>0.8345</td><td>0.404</td><td>0.02489</td><td>0.002102</td><td>0.999</td><td>0.01122</td><td>0.005141</td><td>0.008237</td><td>Vitamin and mineral supplements: Multivitamins +/- minerals</td><td>FALSE</td><td></td><td></td><td></td><td>359245</td><td>77472</td><td>281773</td><td>UK Biobank</td><td>https://docs.google.com/spreadsheets/d/1kvPoupSzsSFBNSztMzl04xMoSC3Kcx3CrjVf4yBmESU/edit?ts=5b5f17db#gid=227859291</td><td>PHESANT Transformation:6155_0 || CAT-MUL-BINARY-VAR 7 || NO_NAN Remove NA participants 514 || Removed 1435 examples != 7 but with missing value (<0) || sample 281773/77472(359245) ||-Notes:ACE touchscreen question Do you regularly take any of the following? (You can select more than one answer) The following checks were performed: If code -7 was selected, then no additional choices were allowed. If code -3 was selected, then no additional choices were allowed.-Variable type:binary</td></tr>
<tr><td>binary.6156_100.txt</td><td>1710174270056F5forCTG.txt.gz</td><td>-0.45</td><td>0.1511</td><td>-2.979</td><td>0.002891</td><td>0.04113</td><td>0.02098</td><td>0.999</td><td>0.00888</td><td>0.006786</td><td>0.00794</td><td>Manic/hyper symptoms: None of the above</td><td>FALSE</td><td></td><td></td><td></td><td>24049</td><td>8672</td><td>15377</td><td>UK Biobank</td><td>https://docs.google.com/spreadsheets/d/1kvPoupSzsSFBNSztMzl04xMoSC3Kcx3CrjVf4yBmESU/edit?ts=5b5f17db#gid=227859291</td><td>PHESANT Transformation:6156_0 || CAT-MUL-BINARY-VAR 100 || NO_NAN Remove NA participants 337145 || Removed 0 examples != 100 but with missing value (<0) || sample 15377/8672(24049) ||-Notes:ACE touchscreen question Please try to remember a period when you were in a high or irritable state and select which of the following apply. The following checks were performed: If code 15 was selected, then no additional choices were allowed. If code -7 was selected, then no additional choices were allowed. ~F6156~ was collected from participants who indicated they have had a period of at least two days when they were more high, excited or hyper than their normal self, or so irritable they shouted or started fights or arguments, as defined by their answers to ~F4642~ and ~F4642~-Variable type:binary</td></tr>
</table>

binary.6156_11.txt	1710174270056F5 forCTG.txt.gz	0.09329	0.1233	0.7563	0.4495	0.03256	0.02191	0.996	0.009669	-0.01041	0.008284	Manic/hyper symptoms: I was more active than usual	FALSE				24049	6030	18019	UK Biobank	https://docs.google.com/spreadsheets/d/1kvPoupSzsSFBNSztMzl04xMoSC3Kcx3CrjVf4yBmESU/edit?ts=5b5f17db#gid=227859291	PHESANT Transformation:6156_0 | | CAT-MUL-BINARY-VAR 11 | | NO_NAN Remove NA participants 337145 | | Removed 0 examples != 11 but with missing value (<0) | | sample 18019/6030(24049) | |-Notes:ACE touchscreen question Please try to remember a period when you were in a high or irritable state and select which of the following apply. The following checks were performed: If code 15 was selected, then no additional choices were allowed. If code -7 was selected, then no additional choices were allowed. ~F6156~ was collected from participants who indicated they have had a period of at least two days when they were more high, excited or hyper than their normal self, or so irritable they shouted or started fights or arguments, as defined by their answers to ~F4642~ and ~F4642~-Variable type:binary
binary.6157_1.txt	1710174270056F5 forCTG.txt.gz	0.2221	0.09035	2.459	0.01394	0.01924	0.006474	1	0.01037	0.005422	0.007443	Why stopped smoking: Illness or ill health	FALSE				88516	10637	77879	UK Biobank	https://docs.google.com/spreadsheets/d/1kvPoupSzsSFBNSztMzl04xMoSC3Kcx3CrjVf4yBmESU/edit?ts=5b5f17db#gid=227859291	PHESANT Transformation:6157_0 | | CAT-MUL-BINARY-VAR 1 | | NO_NAN Remove NA participants 271928 | | Removed 750 examples != 1 but with missing value (<0) | | sample 77879/10637(88516) | |-Notes:ACE touchscreen question Why did you stop smoking? (You can select more than one answer) The following checks were performed: If code -7 was selected, then no additional choices were allowed. If code -1 was selected, then no additional choices were allowed. If code -3 was selected, then no additional choices were allowed. ~F6157~ was collected from participants who indicated that in the past they smoked tobacco on most or all days, as defined by their answers to ~F1249~ and that during the time they smoked they stopped for more than 6 months, as defined by their answers to ~F1249~-Variable type:binary
binary.6157_100.txt	1710174270056F5 forCTG.txt.gz	0.2554	0.05942	4.298	1.73E-05	0.04	0.006441	0.989	0.009211	0.009805	0.008149	Why stopped smoking: None of the above	FALSE	Lifestyle			88516	17748	70768	UK Biobank	https://docs.google.com/spreadsheets/d/1kvPoupSzsSFBNSztMzl04xMoSC3Kcx3CrjVf4yBmESU/edit?ts=5b5f17db#gid=227859291	PHESANT Transformation:6157_0 | | CAT-MUL-BINARY-VAR 100 | | NO_NAN Remove NA participants 271928 | | Removed 750 examples != 100 but with missing value (<0) | | sample 70768/17748(88516) | |-Notes:ACE touchscreen question Why did you stop smoking? (You can select more than one answer) The following checks were performed: If code -7 was selected, then no additional choices were allowed. If code -1 was selected, then no additional choices were allowed. If code -3 was selected, then no additional choices were allowed. ~F6157~ was collected from participants who indicated that in the past they smoked tobacco on most or all days, as defined by their answers to ~F1249~ and that during the time they smoked they stopped for more than 6 months, as defined by their answers to ~F1249~-Variable type:binary
binary.6157_2.txt	1710174270056F5 forCTG.txt.gz	0.4423	0.1697	2.607	0.009134	0.01081	0.005839	1.016	0.009155	-0.002782	0.008339	Why stopped smoking: Doctor's advice	FALSE				88516	6356	82160	UK Biobank	https://docs.google.com/spreadsheets/d/1kvPoupSzsSFBNSztMzl04xMoSC3Kcx3CrjVf4yBmESU/edit?ts=5b5f17db#gid=227859291	PHESANT Transformation:6157_0 | | CAT-MUL-BINARY-VAR 2 | | NO_NAN Remove NA participants 271928 | | Removed 750 examples != 2 but with missing value (<0) | | sample 82160/6356(88516) | | SKIP_val:-1 < 0 | | SKIP_val:-3 < 0 | |-Notes:ACE touchscreen question Why did you stop smoking? (You can select more than one answer) The following checks were performed: If code -7 was selected, then no additional choices were allowed. If code -1 was selected, then no additional choices were allowed. If code -3 was selected, then no additional choices were allowed. ~F6157~ was collected from participants who indicated that in the past they smoked tobacco on most or all days, as defined by their answers to ~F1249~ and that during the time they smoked they stopped for more than 6 months, as defined by their answers to ~F1249~-Variable type:binary
binary.6157_3.txt	1710174270056F5 forCTG.txt.gz	-0.3942	0.06125	-6.435	1.24E-10	0.04926	0.006908	1.002	0.009932	-0.01003	0.008201	Why stopped smoking: Health precaution	FALSE	Lifestyle			88516	55663	32853	UK Biobank	https://docs.google.com/spreadsheets/d/1kvPoupSzsSFBNSztMzl04xMoSC3Kcx3CrjVf4yBmESU/edit?ts=5b5f17db#gid=227859291	PHESANT Transformation:6157_0 | | CAT-MUL-BINARY-VAR 3 | | NO_NAN Remove NA participants 271928 | | Removed 750 examples != 3 but with missing value (<0) | | sample 32853/55663(88516) | |-Notes:ACE touchscreen question Why did you stop smoking? (You can select more than one answer) The following checks were performed: If code -7 was selected, then no additional choices were allowed. If code -1 was selected, then no additional choices were allowed. If code -3 was selected, then no additional choices were allowed. ~F6157~ was collected from participants who indicated that in the past they smoked tobacco on most or all days, as defined by their answers to ~F1249~ and that during the time they smoked they stopped for more than 6 months, as defined by their answers to ~F1249~-Variable type:binary
binary.6157_4.txt	1710174270056F5 forCTG.txt.gz	-0.027	0.08533	-0.3164	0.7517	0.01819	0.006375	0.992	0.01006	0.0001999	0.008475	Why stopped smoking: Financial reasons	FALSE				88516	21653	66863	UK Biobank	https://docs.google.com/spreadsheets/d/1kvPoupSzsSFBNSztMzl04xMoSC3Kcx3CrjVf4yBmESU/edit?ts=5b5f17db#gid=227859291	PHESANT Transformation:6157_0 | | CAT-MUL-BINARY-VAR 4 | | NO_NAN Remove NA participants 271928 | | Removed 750 examples != 4 but with missing value (<0) | | sample 66863/21653(88516) | |-Notes:ACE touchscreen question Why did you stop smoking? (You can select more than one answer) The following checks were performed: If code -7 was selected, then no additional choices were allowed. If code -1 was selected, then no additional choices were allowed. If code -3 was selected, then no additional choices were allowed. ~F6157~ was collected from participants who indicated that in the past they smoked tobacco on most or all days, as defined by their answers to ~F1249~ and that during the time they smoked they stopped for more than 6 months, as defined by their answers to ~F1249~-Variable type:binary
binary.6158_1.txt	1710174270056F5 forCTG.txt.gz	0.5	0.1961	2.55	0.01076	0.0687	0.04448	1.003	0.008805	-0.009473	0.00785	Why reduced smoking: Illness or ill health	FALSE				11443	1604	9839	UK Biobank	https://docs.google.com/spreadsheets/d/1kvPoupSzsSFBNSztMzl04xMoSC3Kcx3CrjVf4yBmESU/edit?ts=5b5f17db#gid=227859291	PHESANT Transformation:6158_0 | | CAT-MUL-BINARY-VAR 1 | | NO_NAN Remove NA participants 349256 | | Removed 495 examples != 1 but with missing value (<0) | | sample 9839/1604(11443) | |-Notes:ACE touchscreen question Why did you reduce your smoking? (You can select more than one answer) The following checks were performed: If code -7 was selected, then no additional choices were allowed. If code -1 was selected, then no additional choices were allowed. If code -3 was selected, then no additional choices were allowed. ~F6158~ was collected from participants who indicated they currently smoke tobacco on most or all days, as defined by their answers to ~F1239~ and those who smoke less nowadays than 10 years ago, as defined by their answers to ~F1239~-Variable type:binary
binary.6158_3.txt	1710174270056F5 forCTG.txt.gz	-0.317	0.1472	-2.153	0.03128	0.08006	0.04806	0.992	0.0094	-0.003127	0.008555	Why reduced smoking: Health precaution	FALSE				11443	5443	6000	UK Biobank	https://docs.google.com/spreadsheets/d/1kvPoupSzsSFBNSztMzl04xMoSC3Kcx3CrjVf4yBmESU/edit?ts=5b5f17db#gid=227859291	PHESANT Transformation:6158_0 | | CAT-MUL-BINARY-VAR 3 | | NO_NAN Remove NA participants 349256 | | Removed 495 examples != 3 but with missing value (<0) | | sample 6000/5443(11443) | |-Notes:ACE touchscreen question Why did you reduce your smoking? (You can select more than one answer) The following checks were performed: If code -7 was selected, then no additional choices were allowed. If code -1 was selected, then no additional choices were allowed. If code -3 was selected, then no additional choices were allowed. ~F6158~ was collected from participants who indicated they currently smoke tobacco on most or all days, as defined by their answers to ~F1239~ and those who smoke less nowadays than 10 years ago, as defined by their answers to ~F1239~-Variable type:binary
binary.6159_1.txt	1710174270056F5 forCTG.txt.gz	0.2204	0.03556	6.197	5.74E-10	0.04305	0.003398	1.009	0.01507	0.00889	0.007911	Pain type(s) experienced in last month: Headache	FALSE	Pain			360391	71672	288719	UK Biobank	https://docs.google.com/spreadsheets/d/1kvPoupSzsSFBNSztMzl04xMoSC3Kcx3CrjVf4yBmESU/edit?ts=5b5f17db#gid=227859291	PHESANT Transformation:6159_0 | | CAT-MUL-BINARY-VAR 1 | | NO_NAN Remove NA participants 180 | | Removed 623 examples != 1 but with missing value (<0) | | sample 288719/71672(360391) | |-Notes:ACE touchscreen question In the last month have you experienced any of the following that interfered with your usual activites? (You can select more than one answer) The following checks were performed: If code 8 was selected, then no additional choices were allowed. If code -7 was selected, then no additional choices were allowed. If code -3 was selected, then no additional choices were allowed.-Variable type:binary
binary.6159_100.txt	1710174270056F5 forCTG.txt.gz	-0.4475	0.03178	-14.08	4.94E-45	0.04889	0.003002	1.047	0.01338	-0.003187	0.009323	Pain type(s) experienced in last month: None of the above	FALSE	Pain			360391	145514	214877	UK Biobank	https://docs.google.com/spreadsheets/d/1kvPoupSzsSFBNSztMzl04xMoSC3Kcx3CrjVf4yBmESU/edit?ts=5b5f17db#gid=227859291	PHESANT Transformation:6159_0 | | CAT-MUL-BINARY-VAR 100 | | NO_NAN Remove NA participants 180 | | Removed 623 examples != 100 but with missing value (<0) | | sample 214877/145514(360391) | |-Notes:ACE touchscreen question In the last month have you experienced any of the following that interfered with your usual activites? (You can select more than one answer) The following checks were performed: If code 8 was selected, then no additional choices were allowed. If code -7 was selected, then no additional choices were allowed. If code -3 was selected, then no additional choices were allowed.-Variable type:binary
binary.6159_2.txt	1710174270056F5 forCTG.txt.gz	0.3635	0.07587	4.791	1.66E-06	0.007678	0.001674	0.992	0.009671	0.006621	0.008147	Pain type(s) experienced in last month: Facial pain	FALSE	Pain			360391	6599	353792	UK Biobank	https://docs.google.com/spreadsheets/d/1kvPoupSzsSFBNSztMzl04xMoSC3Kcx3CrjVf4yBmESU/edit?ts=5b5f17db#gid=227859291	PHESANT Transformation:6159_0 | | CAT-MUL-BINARY-VAR 2 | | NO_NAN Remove NA participants 180 | | Removed 623 examples != 2 but with missing value (<0) | | sample 353792/6599(360391) | |-Notes:ACE touchscreen question In the last month have you experienced any of the following that interfered with your usual activites? (You can select more than one answer) The following checks were performed: If code 8 was selected, then no additional choices were allowed. If code -7 was selected, then no additional choices were allowed. If code -3 was selected, then no additional choices were allowed.-Variable type:binary

<table>
<tr><td>binary.6159_4.txt</td><td>1710174270056F5forCTG.txt.gz</td><td>0.3562</td><td>0.03708</td><td>9.607</td><td>7.44E-22</td><td>0.03754</td><td>0.002311</td><td>1.019</td><td>0.01139</td><td>-0.000307</td><td>0.008995</td><td>Pain type(s) experienced in last month: Back pain</td><td>FALSE</td><td>Pain</td><td></td><td></td><td>360391</td><td>91349</td><td>269042</td><td>UK Biobank</td><td>https://docs.google.com/spreadsheets/d/1kvPoupSzsSFBNSztMzl04xMoSC3Kcx3CrjVf4yBmESU/edit?ts=5b5f17db#gid=227859291</td><td>PHESANT Transformation:6159_0 | | CAT-MUL-BINARY-VAR 4 | | NO_NAN Remove NA participants 180 | | Removed 623 examples != 4 but with missing value (<0) | | sample 269042/91349(360391) | | -Notes:ACE touchscreen question In the last month have you experienced any of the following that interfered with your usual activites? (You can select more than one answer) The following checks were performed: If code 8 was selected, then no additional choices were allowed. If code -7 was selected, then no additional choices were allowed. If code -3 was selected, then no additional choices were allowed.-Variable type:binary</td></tr>
<tr><td>binary.6159_5.txt</td><td>1710174270056F5forCTG.txt.gz</td><td>0.364</td><td>0.05391</td><td>6.751</td><td>1.47E-11</td><td>0.01867</td><td>0.001909</td><td>1.018</td><td>0.01068</td><td>0.007885</td><td>0.009173</td><td>Pain type(s) experienced in last month: Stomach or abdominal pain</td><td>FALSE</td><td>Pain</td><td></td><td></td><td>360391</td><td>30306</td><td>330085</td><td>UK Biobank</td><td>https://docs.google.com/spreadsheets/d/1kvPoupSzsSFBNSztMzl04xMoSC3Kcx3CrjVf4yBmESU/edit?ts=5b5f17db#gid=227859291</td><td>PHESANT Transformation:6159_0 | | CAT-MUL-BINARY-VAR 5 | | NO_NAN Remove NA participants 180 | | Removed 623 examples != 5 but with missing value (<0) | | sample 330085/30306(360391) | | -Notes:ACE touchscreen question In the last month have you experienced any of the following that interfered with your usual activites? (You can select more than one answer) The following checks were performed: If code 8 was selected, then no additional choices were allowed. If code -7 was selected, then no additional choices were allowed. If code -3 was selected, then no additional choices were allowed.-Variable type:binary</td></tr>
<tr><td>binary.6159_6.txt</td><td>1710174270056F5forCTG.txt.gz</td><td>0.4494</td><td>0.04257</td><td>10.56</td><td>4.72E-26</td><td>0.02422</td><td>0.002158</td><td>1.01</td><td>0.01108</td><td>0.007092</td><td>0.008156</td><td>Pain type(s) experienced in last month: Hip pain</td><td>FALSE</td><td>Pain</td><td></td><td></td><td>360391</td><td>40500</td><td>319891</td><td>UK Biobank</td><td>https://docs.google.com/spreadsheets/d/1kvPoupSzsSFBNSztMzl04xMoSC3Kcx3CrjVf4yBmESU/edit?ts=5b5f17db#gid=227859291</td><td>PHESANT Transformation:6159_0 | | CAT-MUL-BINARY-VAR 6 | | NO_NAN Remove NA participants 180 | | Removed 623 examples != 6 but with missing value (<0) | | sample 319891/40500(360391) | | -Notes:ACE touchscreen question In the last month have you experienced any of the following that interfered with your usual activites? (You can select more than one answer) The following checks were performed: If code 8 was selected, then no additional choices were allowed. If code -7 was selected, then no additional choices were allowed. If code -3 was selected, then no additional choices were allowed.-Variable type:binary</td></tr>
<tr><td>binary.6159_7.txt</td><td>1710174270056F5forCTG.txt.gz</td><td>0.3803</td><td>0.03707</td><td>10.26</td><td>1.08E-24</td><td>0.0386</td><td>0.002586</td><td>1.011</td><td>0.0127</td><td>0.008614</td><td>0.009098</td><td>Pain type(s) experienced in last month: Knee pain</td><td>FALSE</td><td>Pain</td><td></td><td></td><td>360391</td><td>76628</td><td>283763</td><td>UK Biobank</td><td>https://docs.google.com/spreadsheets/d/1kvPoupSzsSFBNSztMzl04xMoSC3Kcx3CrjVf4yBmESU/edit?ts=5b5f17db#gid=227859291</td><td>PHESANT Transformation:6159_0 | | CAT-MUL-BINARY-VAR 7 | | NO_NAN Remove NA participants 180 | | Removed 623 examples != 7 but with missing value (<0) | | sample 283763/76628(360391) | | -Notes:ACE touchscreen question In the last month have you experienced any of the following that interfered with your usual activites? (You can select more than one answer) The following checks were performed: If code 8 was selected, then no additional choices were allowed. If code -7 was selected, then no additional choices were allowed. If code -3 was selected, then no additional choices were allowed.-Variable type:binary</td></tr>
<tr><td>binary.6159_8.txt</td><td>1710174270056F5forCTG.txt.gz</td><td>0.5794</td><td>0.07248</td><td>7.994</td><td>1.30E-15</td><td>0.008767</td><td>0.001573</td><td>1.008</td><td>0.00899</td><td>0.01142</td><td>0.007791</td><td>Pain type(s) experienced in last month: Pain all over the body</td><td>FALSE</td><td>Pain</td><td></td><td></td><td>360391</td><td>5530</td><td>354861</td><td>UK Biobank</td><td>https://docs.google.com/spreadsheets/d/1kvPoupSzsSFBNSztMzl04xMoSC3Kcx3CrjVf4yBmESU/edit?ts=5b5f17db#gid=227859291</td><td>PHESANT Transformation:6159_0 | | CAT-MUL-BINARY-VAR 8 | | NO_NAN Remove NA participants 180 | | Removed 623 examples != 8 but with missing value (<0) | | sample 354861/5530(360391) | | SKIP_val:-3 < 0 | | -Notes:ACE touchscreen question In the last month have you experienced any of the following that interfered with your usual activites? (You can select more than one answer) The following checks were performed: If code 8 was selected, then no additional choices were allowed. If code -7 was selected, then no additional choices were allowed. If code -3 was selected, then no additional choices were allowed.-Variable type:binary</td></tr>
<tr><td>binary.6160_1.txt</td><td>1710174270056F5forCTG.txt.gz</td><td>-0.2293</td><td>0.04164</td><td>-5.506</td><td>3.66E-08</td><td>0.03591</td><td>0.002412</td><td>1.007</td><td>0.01246</td><td>-0.005817</td><td>0.009374</td><td>Leisure/social activities: Sports club or gym</td><td>TRUE</td><td>Lifestyle</td><td></td><td>Attending a sports club or gym</td><td>360063</td><td>108473</td><td>251590</td><td>UK Biobank</td><td>https://docs.google.com/spreadsheets/d/1kvPoupSzsSFBNSztMzl04xMoSC3Kcx3CrjVf4yBmESU/edit?ts=5b5f17db#gid=227859291</td><td>PHESANT Transformation:6160_0 | | CAT-MUL-BINARY-VAR 1 | | NO_NAN Remove NA participants 177 | | Removed 954 examples != 1 but with missing value (<0) | | sample 251590/108473(360063) | | -Notes:ACE touchscreen question Which of the following do you attend once a week or more often? (You can select more than one) The following checks were performed: If code -7 was selected, then no additional choices were allowed. If code -3 was selected, then no additional choices were allowed. If the participant activated the Help button they were shown the message: If this varies, please think about activities in the last year.-Variable type:binary</td></tr>
<tr><td>binary.6160_100.txt</td><td>1710174270056F5forCTG.txt.gz</td><td>0.1763</td><td>0.04098</td><td>4.302</td><td>1.69E-05</td><td>0.03574</td><td>0.00234</td><td>1.012</td><td>0.01077</td><td>0.01672</td><td>0.009774</td><td>Leisure/social activities: None of the above</td><td>FALSE</td><td>Lifestyle</td><td></td><td></td><td>360063</td><td>108704</td><td>251359</td><td>UK Biobank</td><td>https://docs.google.com/spreadsheets/d/1kvPoupSzsSFBNSztMzl04xMoSC3Kcx3CrjVf4yBmESU/edit?ts=5b5f17db#gid=227859291</td><td>PHESANT Transformation:6160_0 | | CAT-MUL-BINARY-VAR 100 | | NO_NAN Remove NA participants 177 | | Removed 954 examples != 100 but with missing value (<0) | | sample 251359/108704(360063) | | -Notes:ACE touchscreen question Which of the following do you attend once a week or more often? (You can select more than one) The following checks were performed: If code -7 was selected, then no additional choices were allowed. If code -3 was selected, then no additional choices were allowed. If the participant activated the Help button they were shown the message: If this varies, please think about activities in the last year.-Variable type:binary</td></tr>
<tr><td>binary.6160_2.txt</td><td>1710174270056F5forCTG.txt.gz</td><td>0.109</td><td>0.03934</td><td>2.772</td><td>0.005573</td><td>0.03422</td><td>0.002514</td><td>1.008</td><td>0.01243</td><td>-0.01959</td><td>0.009118</td><td>Leisure/social activities: Pub or social club</td><td>FALSE</td><td></td><td></td><td></td><td>360063</td><td>98499</td><td>261564</td><td>UK Biobank</td><td>https://docs.google.com/spreadsheets/d/1kvPoupSzsSFBNSztMzl04xMoSC3Kcx3CrjVf4yBmESU/edit?ts=5b5f17db#gid=227859291</td><td>PHESANT Transformation:6160_0 | | CAT-MUL-BINARY-VAR 2 | | NO_NAN Remove NA participants 177 | | Removed 954 examples != 2 but with missing value (<0) | | sample 261564/98499(360063) | | -Notes:ACE touchscreen question Which of the following do you attend once a week or more often? (You can select more than one) The following checks were performed: If code -7 was selected, then no additional choices were allowed. If code -3 was selected, then no additional choices were allowed. If the participant activated the Help button they were shown the message: If this varies, please think about activities in the last year.-Variable type:binary</td></tr>
<tr><td>binary.6160_3.txt</td><td>1710174270056F5forCTG.txt.gz</td><td>-0.2243</td><td>0.03453</td><td>-6.496</td><td>8.24E-11</td><td>0.04583</td><td>0.002867</td><td>1.029</td><td>0.01386</td><td>0.008494</td><td>0.009567</td><td>Leisure/social activities: Religious group</td><td>FALSE</td><td>Lifestyle</td><td></td><td></td><td>360063</td><td>52697</td><td>307366</td><td>UK Biobank</td><td>https://docs.google.com/spreadsheets/d/1kvPoupSzsSFBNSztMzl04xMoSC3Kcx3CrjVf4yBmESU/edit?ts=5b5f17db#gid=227859291</td><td>PHESANT Transformation:6160_0 | | CAT-MUL-BINARY-VAR 3 | | NO_NAN Remove NA participants 177 | | Removed 954 examples != 3 but with missing value (<0) | | sample 307366/52697(360063) | | -Notes:ACE touchscreen question Which of the following do you attend once a week or more often? (You can select more than one) The following checks were performed: If code -7 was selected, then no additional choices were allowed. If code -3 was selected, then no additional choices were allowed. If the participant activated the Help button they were shown the message: If this varies, please think about activities in the last year.-Variable type:binary</td></tr>
<tr><td>binary.6160_4.txt</td><td>1710174270056F5forCTG.txt.gz</td><td>-0.1224</td><td>0.0505</td><td>-2.423</td><td>0.01539</td><td>0.0172</td><td>0.001828</td><td>0.99</td><td>0.01019</td><td>-0.000286</td><td>0.008009</td><td>Leisure/social activities: Adult education class</td><td>FALSE</td><td></td><td></td><td></td><td>360063</td><td>25842</td><td>334221</td><td>UK Biobank</td><td>https://docs.google.com/spreadsheets/d/1kvPoupSzsSFBNSztMzl04xMoSC3Kcx3CrjVf4yBmESU/edit?ts=5b5f17db#gid=227859291</td><td>PHESANT Transformation:6160_0 | | CAT-MUL-BINARY-VAR 4 | | NO_NAN Remove NA participants 177 | | Removed 954 examples != 4 but with missing value (<0) | | sample 334221/25842(360063) | | SKIP_val:-3 < 0 | | -Notes:ACE touchscreen question Which of the following do you attend once a week or more often? (You can select more than one) The following checks were performed: If code -7 was selected, then no additional choices were allowed. If code -3 was selected, then no additional choices were allowed. If the participant activated the Help button they were shown the message: If this varies, please think about activities in the last year.-Variable type:binary</td></tr>
<tr><td>binary.6160_5.txt</td><td>1710174270056F5forCTG.txt.gz</td><td>-0.1602</td><td>0.04709</td><td>-3.402</td><td>0.00067</td><td>0.02167</td><td>0.00205</td><td>1.014</td><td>0.01155</td><td>0.006226</td><td>0.008115</td><td>Leisure/social activities: Other group activity</td><td>FALSE</td><td></td><td></td><td></td><td>360063</td><td>80178</td><td>279885</td><td>UK Biobank</td><td>https://docs.google.com/spreadsheets/d/1kvPoupSzsSFBNSztMzl04xMoSC3Kcx3CrjVf4yBmESU/edit?ts=5b5f17db#gid=227859291</td><td>PHESANT Transformation:6160_0 | | CAT-MUL-BINARY-VAR 5 | | NO_NAN Remove NA participants 177 | | Removed 954 examples != 5 but with missing value (<0) | | sample 279885/80178(360063) | | -Notes:ACE touchscreen question Which of the following do you attend once a week or more often? (You can select more than one) The following checks were performed: If code -7 was selected, then no additional choices were allowed. If code -3 was selected, then no additional choices were allowed. If the participant activated the Help button they were shown the message: If this varies, please think about activities in the last year.-Variable type:binary</td></tr>
<tr><td>binary.6162_1.txt</td><td>1710174270056F5forCTG.txt.gz</td><td>-0.1041</td><td>0.04205</td><td>-2.476</td><td>0.0133</td><td>0.02528</td><td>0.001953</td><td>1.017</td><td>0.01048</td><td>0.002087</td><td>0.008371</td><td>Types of transport used (excluding work): Car/motor vehicle</td><td>FALSE</td><td></td><td></td><td></td><td>359324</td><td>285459</td><td>73865</td><td>UK Biobank</td><td>https://docs.google.com/spreadsheets/d/1kvPoupSzsSFBNSztMzl04xMoSC3Kcx3CrjVf4yBmESU/edit?ts=5b5f17db#gid=227859291</td><td>PHESANT Transformation:6162_0 | | CAT-MUL-BINARY-VAR 1 | | NO_NAN Remove NA participants 1769 | | Removed 101 examples != 1 but with missing value (<0) | | sample 73865/285459(359324) | | -Notes:ACE touchscreen question In the last 4 weeks, which forms of transport have you used most often to get about? (Not including any journeys to and from work; you can select more than one answer) The following checks were performed: If code -7 was selected, then no additional choices were allowed. If code -3 was selected, then no additional choices were allowed. If the participant activated the Help button they were shown the message: Remember not to include journeys to and from work. ~F6162~ was collected from all participants except those who indicated they were unable to walk, as defined by their answers to ~F864~-Variable type:binary</td></tr>
<tr><td>binary.6162_100.txt</td><td>1710174270056F5forCTG.txt.gz</td><td>0.09502</td><td>0.09107</td><td>1.043</td><td>0.2968</td><td>0.00411</td><td>0.001525</td><td>0.996</td><td>0.00934</td><td>0.01013</td><td>0.008342</td><td>Types of transport used (excluding work): None of the above</td><td>FALSE</td><td></td><td></td><td></td><td>359324</td><td>795</td><td>358529</td><td>UK Biobank</td><td>https://docs.google.com/spreadsheets/d/1kvPoupSzsSFBNSztMzl04xMoSC3Kcx3CrjVf4yBmESU/edit?ts=5b5f17db#gid=227859291</td><td>PHESANT Transformation:6162_0 | | CAT-MUL-BINARY-VAR 100 | | NO_NAN Remove NA participants 1769 | | Removed 101 examples != 100 but with missing value (<0) | | sample 358529/795(359324) | | SKIP_val:-3 < 0 | | -Notes:ACE touchscreen question In the last 4 weeks, which forms of transport have you used most often to get about? (Not including any journeys to and from work; you can select more than one answer) The following checks were performed: If code -7 was selected, then no additional choices were allowed. If code -3 was selected, then no additional choices were allowed. If the participant activated the Help button they were shown the message: Remember not to include journeys to and from work. ~F6162~ was collected from all participants except those who indicated they were unable to walk, as defined by their answers to ~F864~-Variable type:binary</td></tr>
</table>

<table>
<tr><td>binary.6162_2.txt</td><td>1710174270056F5forCTG.txt.gz</td><td>-0.2593</td><td>0.03857</td><td>-6.724</td><td>1.77E-11</td><td>0.03116</td><td>0.002497</td><td>1.028</td><td>0.01195</td><td>-0.001777</td><td>0.009222</td><td>Types of transport used (excluding work): Walk</td><td>TRUE</td><td>Lifestyle</td><td></td><td>Walking as a general means of transport</td><td>359324</td><td>176934</td><td>182390</td><td>UK Biobank</td><td>https://docs.google.com/spreadsheets/d/1kvPoupSzsSFBNSztMzl04xMoSC3Kcx3CrjVf4yBmESU/edit?ts=5b5f17db#gid=227859291</td><td>PHESANT Transformation:6162_0 | | CAT-MUL-BINARY-VAR 2 | | NO_NAN Remove NA participants 1769 | | Removed 101 examples != 2 but with missing value (<0) | | sample 182390/176934(359324) | |-Notes:ACE touchscreen question In the last 4 weeks, which forms of transport have you used most often to get about? (Not including any journeys to and from work; you can select more than one answer) The following checks were performed: If code -7 was selected, then no additional choices were allowed. If code -3 was selected, then no additional choices were allowed. If the participant activated the Help button they were shown the message: Remember not to include journeys to and from work. ~F6162~ was collected from all participants except those who indicated they were unable to walk, as defined by their answers to ~F864~-Variable type:binary</td></tr>
<tr><td>binary.6162_3.txt</td><td>1710174270056F5forCTG.txt.gz</td><td>-0.2247</td><td>0.04487</td><td>-5.007</td><td>5.53E-07</td><td>0.02425</td><td>0.002032</td><td>1.006</td><td>0.0108</td><td>3.90E-06</td><td>0.009674</td><td>Types of transport used (excluding work): Public transport</td><td>FALSE</td><td>Lifestyle</td><td></td><td></td><td>359324</td><td>107727</td><td>251597</td><td>UK Biobank</td><td>https://docs.google.com/spreadsheets/d/1kvPoupSzsSFBNSztMzl04xMoSC3Kcx3CrjVf4yBmESU/edit?ts=5b5f17db#gid=227859291</td><td>PHESANT Transformation:6162_0 | | CAT-MUL-BINARY-VAR 3 | | NO_NAN Remove NA participants 1769 | | Removed 101 examples != 3 but with missing value (<0) | | sample 251597/107727(359324) | |-Notes:ACE touchscreen question In the last 4 weeks, which forms of transport have you used most often to get about? (Not including any journeys to and from work; you can select more than one answer) The following checks were performed: If code -7 was selected, then no additional choices were allowed. If code -3 was selected, then no additional choices were allowed. If the participant activated the Help button they were shown the message: Remember not to include journeys to and from work. ~F6162~ was collected from all participants except those who indicated they were unable to walk, as defined by their answers to ~F864~-Variable type:binary</td></tr>
<tr><td>binary.6162_4.txt</td><td>1710174270056F5forCTG.txt.gz</td><td>-0.08491</td><td>0.0429</td><td>-1.979</td><td>0.0478</td><td>0.02435</td><td>0.002065</td><td>1.031</td><td>0.01071</td><td>-0.01393</td><td>0.008466</td><td>Types of transport used (excluding work): Cycle</td><td>FALSE</td><td></td><td></td><td></td><td>359324</td><td>26059</td><td>333265</td><td>UK Biobank</td><td>https://docs.google.com/spreadsheets/d/1kvPoupSzsSFBNSztMzl04xMoSC3Kcx3CrjVf4yBmESU/edit?ts=5b5f17db#gid=227859291</td><td>PHESANT Transformation:6162_0 | | CAT-MUL-BINARY-VAR 4 | | NO_NAN Remove NA participants 1769 | | Removed 101 examples != 4 but with missing value (<0) | | sample 333265/26059(359324) | |-Notes:ACE touchscreen question In the last 4 weeks, which forms of transport have you used most often to get about? (Not including any journeys to and from work; you can select more than one answer) The following checks were performed: If code -7 was selected, then no additional choices were allowed. If code -3 was selected, then no additional choices were allowed. If the participant activated the Help button they were shown the message: Remember not to include journeys to and from work. ~F6162~ was collected from all participants except those who indicated they were unable to walk, as defined by their answers to ~F864~-Variable type:binary</td></tr>
<tr><td>binary.6164_1.txt</td><td>1710174270056F5forCTG.txt.gz</td><td>-0.2753</td><td>0.04039</td><td>-6.817</td><td>9.28E-12</td><td>0.03351</td><td>0.00235</td><td>1.042</td><td>0.0113</td><td>-0.01854</td><td>0.009829</td><td>Types of physical activity in last 4 weeks: Walking for pleasure (not as a means of transport)</td><td>FALSE</td><td>Lifestyle</td><td></td><td></td><td>359263</td><td>258574</td><td>100689</td><td>UK Biobank</td><td>https://docs.google.com/spreadsheets/d/1kvPoupSzsSFBNSztMzl04xMoSC3Kcx3CrjVf4yBmESU/edit?ts=5b5f17db#gid=227859291</td><td>PHESANT Transformation:6164_0 | | CAT-MUL-BINARY-VAR 1 | | NO_NAN Remove NA participants 1592 | | Removed 339 examples != 1 but with missing value (<0) | | sample 100689/258574(359263) | |-Notes:ACE touchscreen question In the last 4 weeks did you spend any time doing the following? (You can select more than one answer) The following checks were performed: If code -7 was selected, then no additional choices were allowed. If code -3 was selected, then no additional choices were allowed. If the participant activated the Help button they were shown the message: Strenuous sports include sports that make you sweat or breathe hard. Heavy DIY includes chopping wood, home or car maintenance, lifting heavy objects or using heavy tools.-Variable type:binary</td></tr>
<tr><td>binary.6164_100.txt</td><td>1710174270056F5forCTG.txt.gz</td><td>0.3451</td><td>0.04702</td><td>7.339</td><td>2.15E-13</td><td>0.02102</td><td>0.00203</td><td>1.023</td><td>0.01115</td><td>0.003845</td><td>0.008693</td><td>Types of physical activity in last 4 weeks: None of the above</td><td>TRUE</td><td>Lifestyle</td><td></td><td>No physical activity in the last 4 weeks</td><td>359263</td><td>21255</td><td>338008</td><td>UK Biobank</td><td>https://docs.google.com/spreadsheets/d/1kvPoupSzsSFBNSztMzl04xMoSC3Kcx3CrjVf4yBmESU/edit?ts=5b5f17db#gid=227859291</td><td>PHESANT Transformation:6164_0 | | CAT-MUL-BINARY-VAR 100 | | NO_NAN Remove NA participants 1592 | | Removed 339 examples != 100 but with missing value (<0) | | sample 338008/21255(359263) | |-Notes:ACE touchscreen question In the last 4 weeks did you spend any time doing the following? (You can select more than one answer) The following checks were performed: If code -7 was selected, then no additional choices were allowed. If code -3 was selected, then no additional choices were allowed. If the participant activated the Help button they were shown the message: Strenuous sports include sports that make you sweat or breathe hard. Heavy DIY includes chopping wood, home or car maintenance, lifting heavy objects or using heavy tools.-Variable type:binary</td></tr>
<tr><td>binary.6164_3.txt</td><td>1710174270056F5forCTG.txt.gz</td><td>-0.1829</td><td>0.04682</td><td>-3.906</td><td>9.39E-05</td><td>0.02199</td><td>0.001856</td><td>1.025</td><td>0.00991</td><td>-0.009636</td><td>0.008789</td><td>Types of physical activity in last 4 weeks: Strenuous sports</td><td>FALSE</td><td></td><td></td><td></td><td>359263</td><td>37297</td><td>321966</td><td>UK Biobank</td><td>https://docs.google.com/spreadsheets/d/1kvPoupSzsSFBNSztMzl04xMoSC3Kcx3CrjVf4yBmESU/edit?ts=5b5f17db#gid=227859291</td><td>PHESANT Transformation:6164_0 | | CAT-MUL-BINARY-VAR 3 | | NO_NAN Remove NA participants 1592 | | Removed 339 examples != 3 but with missing value (<0) | | sample 321966/37297(359263) | | SKIP_val:-3 < 0 | |-Notes:ACE touchscreen question In the last 4 weeks did you spend any time doing the following? (You can select more than one answer) The following checks were performed: If code -7 was selected, then no additional choices were allowed. If code -3 was selected, then no additional choices were allowed. If the participant activated the Help button they were shown the message: Strenuous sports include sports that make you sweat or breathe hard. Heavy DIY includes chopping wood, home or car maintenance, lifting heavy objects or using heavy tools.-Variable type:binary</td></tr>
<tr><td>binary.6164_4.txt</td><td>1710174270056F5forCTG.txt.gz</td><td>-0.2859</td><td>0.03953</td><td>-7.231</td><td>4.78E-13</td><td>0.0387</td><td>0.00239</td><td>0.992</td><td>0.01205</td><td>0.000333</td><td>0.009727</td><td>Types of physical activity in last 4 weeks: Light DIY (eg: pruning, watering the lawn)</td><td>FALSE</td><td>Lifestyle</td><td></td><td></td><td>359263</td><td>186679</td><td>172584</td><td>UK Biobank</td><td>https://docs.google.com/spreadsheets/d/1kvPoupSzsSFBNSztMzl04xMoSC3Kcx3CrjVf4yBmESU/edit?ts=5b5f17db#gid=227859291</td><td>PHESANT Transformation:6164_0 | | CAT-MUL-BINARY-VAR 4 | | NO_NAN Remove NA participants 1592 | | Removed 339 examples != 4 but with missing value (<0) | | sample 172584/186679(359263) | |-Notes:ACE touchscreen question In the last 4 weeks did you spend any time doing the following? (You can select more than one answer) The following checks were performed: If code -7 was selected, then no additional choices were allowed. If code -3 was selected, then no additional choices were allowed. If the participant activated the Help button they were shown the message: Strenuous sports include sports that make you sweat or breathe hard. Heavy DIY includes chopping wood, home or car maintenance, lifting heavy objects or using heavy tools.-Variable type:binary</td></tr>
<tr><td>binary.6164_5.txt</td><td>1710174270056F5forCTG.txt.gz</td><td>-0.134</td><td>0.0377</td><td>-3.555</td><td>0.000379</td><td>0.02977</td><td>0.002162</td><td>1.022</td><td>0.01131</td><td>-0.006338</td><td>0.00874</td><td>Types of physical activity in last 4 weeks: Heavy DIY (eg: weeding, lawn mowing, carpentry, digging)</td><td>FALSE</td><td></td><td></td><td></td><td>359263</td><td>156597</td><td>202666</td><td>UK Biobank</td><td>https://docs.google.com/spreadsheets/d/1kvPoupSzsSFBNSztMzl04xMoSC3Kcx3CrjVf4yBmESU/edit?ts=5b5f17db#gid=227859291</td><td>PHESANT Transformation:6164_0 | | CAT-MUL-BINARY-VAR 5 | | NO_NAN Remove NA participants 1592 | | Removed 339 examples != 5 but with missing value (<0) | | sample 202666/156597(359263) | |-Notes:ACE touchscreen question In the last 4 weeks did you spend any time doing the following? (You can select more than one answer) The following checks were performed: If code -7 was selected, then no additional choices were allowed. If code -3 was selected, then no additional choices were allowed. If the participant activated the Help button they were shown the message: Strenuous sports include sports that make you sweat or breathe hard. Heavy DIY includes chopping wood, home or car maintenance, lifting heavy objects or using heavy tools.-Variable type:binary</td></tr>
<tr><td>binary.6177_1.txt</td><td>1710174270056F5forCTG.txt.gz</td><td>0.1681</td><td>0.04604</td><td>3.652</td><td>0.000261</td><td>0.06649</td><td>0.01018</td><td>1.073</td><td>0.03164</td><td>0.002685</td><td>0.009316</td><td>Medication for cholesterol, blood pressure or diabetes: Cholesterol lowering medication</td><td>FALSE</td><td></td><td></td><td></td><td>165340</td><td>38057</td><td>127283</td><td>UK Biobank</td><td>https://docs.google.com/spreadsheets/d/1kvPoupSzsSFBNSztMzl04xMoSC3Kcx3CrjVf4yBmESU/edit?ts=5b5f17db#gid=227859291</td><td>PHESANT Transformation:6177_0 | | CAT-MUL-BINARY-VAR 1 | | NO_NAN Remove NA participants 194414 | | Removed 1440 examples != 1 but with missing value (<0) | | sample 127283/38057(165340) | | SKIP_val:-1 < 0 | |-Notes:ACE touchscreen question Do you regularly take any of the following medications? (you can select more than one answer) The following checks were performed: If code -7 was selected, then no additional choices were allowed. If code -1 was selected, then no additional choices were allowed. If code -3 was selected, then no additional choices were allowed. If the participant activated the Help button they were shown the message: If you are not sure if you take any of the types of medications, enter Do not know. You will be asked to provide all of the medications that you take later in the visit.-Variable type:binary</td></tr>
<tr><td>binary.6177_100.txt</td><td>1710174270056F5forCTG.txt.gz</td><td>-0.1707</td><td>0.03743</td><td>-4.56</td><td>5.10E-06</td><td>0.09128</td><td>0.007169</td><td>1.073</td><td>0.01895</td><td>-0.001973</td><td>0.008887</td><td>Medication for cholesterol, blood pressure or diabetes: None of the above</td><td>FALSE</td><td>Metabolic</td><td></td><td></td><td>165340</td><td>110372</td><td>54968</td><td>UK Biobank</td><td>https://docs.google.com/spreadsheets/d/1kvPoupSzsSFBNSztMzl04xMoSC3Kcx3CrjVf4yBmESU/edit?ts=5b5f17db#gid=227859291</td><td>PHESANT Transformation:6177_0 | | CAT-MUL-BINARY-VAR 100 | | NO_NAN Remove NA participants 194414 | | Removed 1440 examples != 100 but with missing value (<0) | | sample 54968/110372(165340) | |-Notes:ACE touchscreen question Do you regularly take any of the following medications? (you can select more than one answer) The following checks were performed: If code -7 was selected, then no additional choices were allowed. If code -1 was selected, then no additional choices were allowed. If code -3 was selected, then no additional choices were allowed. If the participant activated the Help button they were shown the message: If you are not sure if you take any of the types of medications, enter Do not know. You will be asked to provide all of the medications that you take later in the visit.-Variable type:binary</td></tr>
<tr><td>binary.6177_2.txt</td><td>1710174270056F5forCTG.txt.gz</td><td>0.1152</td><td>0.03669</td><td>3.139</td><td>0.001698</td><td>0.1043</td><td>0.00717</td><td>1.066</td><td>0.01703</td><td>0.003454</td><td>0.009153</td><td>Medication for cholesterol, blood pressure or diabetes: Blood pressure medication</td><td>FALSE</td><td></td><td></td><td></td><td>165340</td><td>40987</td><td>124353</td><td>UK Biobank</td><td>https://docs.google.com/spreadsheets/d/1kvPoupSzsSFBNSztMzl04xMoSC3Kcx3CrjVf4yBmESU/edit?ts=5b5f17db#gid=227859291</td><td>PHESANT Transformation:6177_0 | | CAT-MUL-BINARY-VAR 2 | | NO_NAN Remove NA participants 194414 | | Removed 1440 examples != 2 but with missing value (<0) | | sample 124353/40987(165340) | |-Notes:ACE touchscreen question Do you regularly take any of the following medications? (you can select more than one answer) The following checks were performed: If code -7 was selected, then no additional choices were allowed. If code -1 was selected, then no additional choices were allowed. If code -3 was selected, then no additional choices were allowed. If the participant activated the Help button they were shown the message: If you are not sure if you take any of the types of medications, enter Do not know. You will be asked to provide all of the medications that you take later in the visit.-Variable type:binary</td></tr>
</table>

<table>
<tr><td>binary.6177_3.txt</td><td>1710174270056F5forCTG.txt.gz</td><td>0.2418</td><td>0.1077</td><td>2.246</td><td>0.02472</td><td>0.008228</td><td>0.003822</td><td>1.01</td><td>0.01054</td><td>0.001519</td><td>0.00808</td><td>Medication for cholesterol, blood pressure or diabetes: Insulin</td><td>FALSE</td><td></td><td></td><td></td><td>165340</td><td>2248</td><td>163092</td><td>UK Biobank</td><td>https://docs.google.com/spreadsheets/d/1kvPoupSzsSFBNSztMzl04xMoSC3Kcx3CrjVf4yBmESU/edit?ts=5b5f17db#gid=227859291</td><td>PHESANT Transformation:6177_0 | | CAT-MUL-BINARY-VAR 3 | | NO_NAN Remove NA participants 194414 | | Removed 1440 examples != 3 but with missing value (<0) | | sample 163092/2248(165340) | | SKIP_val:-3 < 0 | | -Notes:ACE touchscreen question Do you regularly take any of the following medications? (you can select more than one answer) The following checks were performed: If code -7 was selected, then no additional choices were allowed. If code -1 was selected, then no additional choices were allowed. If code -3 was selected, then no additional choices were allowed. If the participant activated the Help button they were shown the message: If you are not sure if you take any of the types of medications, enter Do not know. You will be asked to provide all of the medications that you take later in the visit.-Variable type:binary</td></tr>
<tr><td>binary.6179_1.txt</td><td>1710174270056F5forCTG.txt.gz</td><td>0.06158</td><td>0.04493</td><td>1.37</td><td>0.1705</td><td>0.02616</td><td>0.002048</td><td>1.011</td><td>0.01078</td><td>0.008483</td><td>0.009163</td><td>Mineral and other dietary supplements: Fish oil (including cod liver oil)</td><td>FALSE</td><td></td><td></td><td></td><td>360016</td><td>114131</td><td>245885</td><td>UK Biobank</td><td>https://docs.google.com/spreadsheets/d/1kvPoupSzsSFBNSztMzl04xMoSC3Kcx3CrjVf4yBmESU/edit?ts=5b5f17db#gid=227859291</td><td>PHESANT Transformation:6179_0 | | CAT-MUL-BINARY-VAR 1 | | NO_NAN Remove NA participants 514 | | Removed 664 examples != 1 but with missing value (<0) | | sample 245885/114131(360016) | | -Notes:ACE touchscreen question Do you regularly take any of the following? (You can select more than one answer) The following checks were performed: If code -7 was selected, then no additional choices were allowed. If code -3 was selected, then no additional choices were allowed.-Variable type:binary</td></tr>
<tr><td>binary.6179_100.txt</td><td>1710174270056F5forCTG.txt.gz</td><td>-0.02471</td><td>0.04169</td><td>-0.5926</td><td>0.5534</td><td>0.02992</td><td>0.002345</td><td>1.012</td><td>0.01098</td><td>-0.008375</td><td>0.008858</td><td>Mineral and other dietary supplements: None of the above</td><td>FALSE</td><td></td><td></td><td></td><td>360016</td><td>204810</td><td>155206</td><td>UK Biobank</td><td>https://docs.google.com/spreadsheets/d/1kvPoupSzsSFBNSztMzl04xMoSC3Kcx3CrjVf4yBmESU/edit?ts=5b5f17db#gid=227859291</td><td>PHESANT Transformation:6179_0 | | CAT-MUL-BINARY-VAR 100 | | NO_NAN Remove NA participants 514 | | Removed 664 examples != 100 but with missing value (<0) | | sample 155206/204810(360016) | | -Notes:ACE touchscreen question Do you regularly take any of the following? (You can select more than one answer) The following checks were performed: If code -7 was selected, then no additional choices were allowed. If code -3 was selected, then no additional choices were allowed.-Variable type:binary</td></tr>
<tr><td>binary.6179_2.txt</td><td>1710174270056F5forCTG.txt.gz</td><td>-0.03407</td><td>0.04348</td><td>-0.7835</td><td>0.4333</td><td>0.02323</td><td>0.002144</td><td>1.014</td><td>0.0102</td><td>0.006154</td><td>0.008874</td><td>Mineral and other dietary supplements: Glucosamine</td><td>FALSE</td><td></td><td></td><td></td><td>360016</td><td>70218</td><td>289798</td><td>UK Biobank</td><td>https://docs.google.com/spreadsheets/d/1kvPoupSzsSFBNSztMzl04xMoSC3Kcx3CrjVf4yBmESU/edit?ts=5b5f17db#gid=227859291</td><td>PHESANT Transformation:6179_0 | | CAT-MUL-BINARY-VAR 2 | | NO_NAN Remove NA participants 514 | | Removed 664 examples != 2 but with missing value (<0) | | sample 289798/70218(360016) | | SKIP_val:-3 < 0 | | -Notes:ACE touchscreen question Do you regularly take any of the following? (You can select more than one answer) The following checks were performed: If code -7 was selected, then no additional choices were allowed. If code -3 was selected, then no additional choices were allowed.-Variable type:binary</td></tr>
<tr><td>binary.6179_3.txt</td><td>1710174270056F5forCTG.txt.gz</td><td>-0.04824</td><td>0.06305</td><td>-0.7651</td><td>0.4442</td><td>0.01162</td><td>0.001895</td><td>1.002</td><td>0.009772</td><td>-0.00212</td><td>0.008931</td><td>Mineral and other dietary supplements: Calcium</td><td>FALSE</td><td></td><td></td><td></td><td>360016</td><td>24026</td><td>335990</td><td>UK Biobank</td><td>https://docs.google.com/spreadsheets/d/1kvPoupSzsSFBNSztMzl04xMoSC3Kcx3CrjVf4yBmESU/edit?ts=5b5f17db#gid=227859291</td><td>PHESANT Transformation:6179_0 | | CAT-MUL-BINARY-VAR 3 | | NO_NAN Remove NA participants 514 | | Removed 664 examples != 3 but with missing value (<0) | | sample 335990/24026(360016) | | -Notes:ACE touchscreen question Do you regularly take any of the following? (You can select more than one answer) The following checks were performed: If code -7 was selected, then no additional choices were allowed. If code -3 was selected, then no additional choices were allowed.-Variable type:binary</td></tr>
<tr><td>binary.6179_4.txt</td><td>1710174270056F5forCTG.txt.gz</td><td>0.1796</td><td>0.05468</td><td>3.284</td><td>0.001023</td><td>0.01094</td><td>0.001814</td><td>0.989</td><td>0.00983</td><td>-5.70E-05</td><td>0.007658</td><td>Mineral and other dietary supplements: Zinc</td><td>FALSE</td><td></td><td></td><td></td><td>360016</td><td>14402</td><td>345614</td><td>UK Biobank</td><td>https://docs.google.com/spreadsheets/d/1kvPoupSzsSFBNSztMzl04xMoSC3Kcx3CrjVf4yBmESU/edit?ts=5b5f17db#gid=227859291</td><td>PHESANT Transformation:6179_0 | | CAT-MUL-BINARY-VAR 4 | | NO_NAN Remove NA participants 514 | | Removed 664 examples != 4 but with missing value (<0) | | sample 345614/14402(360016) | | -Notes:ACE touchscreen question Do you regularly take any of the following? (You can select more than one answer) The following checks were performed: If code -7 was selected, then no additional choices were allowed. If code -3 was selected, then no additional choices were allowed.-Variable type:binary</td></tr>
<tr><td>binary.6179_5.txt</td><td>1710174270056F5forCTG.txt.gz</td><td>0.01533</td><td>0.0697</td><td>0.2199</td><td>0.826</td><td>0.006637</td><td>0.001522</td><td>0.992</td><td>0.009527</td><td>0.005024</td><td>0.008298</td><td>Mineral and other dietary supplements: Iron</td><td>FALSE</td><td></td><td></td><td></td><td>360016</td><td>10695</td><td>349321</td><td>UK Biobank</td><td>https://docs.google.com/spreadsheets/d/1kvPoupSzsSFBNSztMzl04xMoSC3Kcx3CrjVf4yBmESU/edit?ts=5b5f17db#gid=227859291</td><td>PHESANT Transformation:6179_0 | | CAT-MUL-BINARY-VAR 5 | | NO_NAN Remove NA participants 514 | | Removed 664 examples != 5 but with missing value (<0) | | sample 349321/10695(360016) | | -Notes:ACE touchscreen question Do you regularly take any of the following? (You can select more than one answer) The following checks were performed: If code -7 was selected, then no additional choices were allowed. If code -3 was selected, then no additional choices were allowed.-Variable type:binary</td></tr>
<tr><td>binary.6179_6.txt</td><td>1710174270056F5forCTG.txt.gz</td><td>0.1516</td><td>0.07402</td><td>2.047</td><td>0.04061</td><td>0.007537</td><td>0.001642</td><td>0.992</td><td>0.009588</td><td>-0.004477</td><td>0.00829</td><td>Mineral and other dietary supplements: Selenium</td><td>FALSE</td><td></td><td></td><td></td><td>360016</td><td>8518</td><td>351498</td><td>UK Biobank</td><td>https://docs.google.com/spreadsheets/d/1kvPoupSzsSFBNSztMzl04xMoSC3Kcx3CrjVf4yBmESU/edit?ts=5b5f17db#gid=227859291</td><td>PHESANT Transformation:6179_0 | | CAT-MUL-BINARY-VAR 6 | | NO_NAN Remove NA participants 514 | | Removed 664 examples != 6 but with missing value (<0) | | sample 351498/8518(360016) | | -Notes:ACE touchscreen question Do you regularly take any of the following? (You can select more than one answer) The following checks were performed: If code -7 was selected, then no additional choices were allowed. If code -3 was selected, then no additional choices were allowed.-Variable type:binary</td></tr>
<tr><td>binary.670_1.txt</td><td>1710174270056F5forCTG.txt.gz</td><td>-0.1368</td><td>0.05074</td><td>-2.696</td><td>0.007025</td><td>0.01458</td><td>0.001924</td><td>1.005</td><td>0.01041</td><td>-0.004385</td><td>0.007831</td><td>Type of accommodation lived in: A house or bungalow</td><td>FALSE</td><td></td><td></td><td></td><td>360088</td><td>326854</td><td>33234</td><td>UK Biobank</td><td>https://docs.google.com/spreadsheets/d/1kvPoupSzsSFBNSztMzl04xMoSC3Kcx3CrjVf4yBmESU/edit?ts=5b5f17db#gid=227859291</td><td>PHESANT Transformation:670_0 | | CAT-SINGLE | | CAT-SINGLE-BINARY-VAR: 1 | | Inc(>=10): 1(326854) | | -Notes:ACE touchscreen question What type of accommodation do you live in? If the participant activated the Help button they were shown the message: Please select: A house or bungalow for any whole, detached, semi-detached or terraced (including end-terrace) house or bungalow. A flat, maisonette, or apartment for any purpose-built block of flats or tenement, part of a converted or shared house (including bed-sits) or within a commercial building (for example in an office building, or hotel, or over a shop). If none of the options apply, select None of the above.-Variable type:binary</td></tr>
<tr><td>binary.670_2.txt</td><td>1710174270056F5forCTG.txt.gz</td><td>0.1086</td><td>0.05242</td><td>2.072</td><td>0.03825</td><td>0.01305</td><td>0.001811</td><td>1.007</td><td>0.00978</td><td>0.005232</td><td>0.007697</td><td>Type of accommodation lived in: A flat, maisonette or apartment</td><td>FALSE</td><td></td><td></td><td></td><td>360088</td><td>31851</td><td>328237</td><td>UK Biobank</td><td>https://docs.google.com/spreadsheets/d/1kvPoupSzsSFBNSztMzl04xMoSC3Kcx3CrjVf4yBmESU/edit?ts=5b5f17db#gid=227859291</td><td>PHESANT Transformation:670_0 | | CAT-SINGLE | | CAT-SINGLE-BINARY-VAR: 2 | | Inc(>=10): 2(31851) | | -Notes:ACE touchscreen question What type of accommodation do you live in? If the participant activated the Help button they were shown the message: Please select: A house or bungalow for any whole, detached, semi-detached or terraced (including end-terrace) house or bungalow. A flat, maisonette, or apartment for any purpose-built block of flats or tenement, part of a converted or shared house (including bed-sits) or within a commercial building (for example in an office building, or hotel, or over a shop). If none of the options apply, select None of the above.-Variable type:binary</td></tr>
<tr><td>binary.670_4.txt</td><td>1710174270056F5forCTG.txt.gz</td><td>0.3814</td><td>0.1846</td><td>2.066</td><td>0.03885</td><td>0.00202</td><td>0.001659</td><td>1</td><td>0.0107</td><td>-0.007573</td><td>0.007178</td><td>Type of accommodation lived in: Sheltered accommodation</td><td>FALSE</td><td></td><td></td><td></td><td>360088</td><td>859</td><td>359229</td><td>UK Biobank</td><td>https://docs.google.com/spreadsheets/d/1kvPoupSzsSFBNSztMzl04xMoSC3Kcx3CrjVf4yBmESU/edit?ts=5b5f17db#gid=227859291</td><td>PHESANT Transformation:670_0 | | CAT-SINGLE | | CAT-SINGLE-BINARY-VAR: 4 | | Inc(>=10): 4(859) | | -Notes:ACE touchscreen question What type of accommodation do you live in? If the participant activated the Help button they were shown the message: Please select: A house or bungalow for any whole, detached, semi-detached or terraced (including end-terrace) house or bungalow. A flat, maisonette, or apartment for any purpose-built block of flats or tenement, part of a converted or shared house (including bed-sits) or within a commercial building (for example in an office building, or hotel, or over a shop). If none of the options apply, select None of the above.-Variable type:binary</td></tr>
<tr><td>binary.680_1.txt</td><td>1710174270056F5forCTG.txt.gz</td><td>-0.2413</td><td>0.03671</td><td>-6.574</td><td>4.90E-11</td><td>0.03262</td><td>0.002411</td><td>0.998</td><td>0.01201</td><td>-0.00399</td><td>0.007959</td><td>Own or rent accommodation lived in: Own outright (by you or someone in your household)</td><td>TRUE</td><td>Wellbeing</td><td></td><td>Home ownership</td><td>356340</td><td>193358</td><td>162982</td><td>UK Biobank</td><td>https://docs.google.com/spreadsheets/d/1kvPoupSzsSFBNSztMzl04xMoSC3Kcx3CrjVf4yBmESU/edit?ts=5b5f17db#gid=227859291</td><td>PHESANT Transformation:680_0 | | CAT-SINGLE | | CAT-SINGLE-BINARY-VAR: 1 | | Inc(>=10): 1(193358) | | -Notes:ACE touchscreen question Do you own or rent the accommodation that you live in? If the participant activated the Help button they were shown the message: Please select: - Own outright if you or someone in your household owns the accommodation that you live in. - Own with mortagage if you or someone in your household has a mortagage on the accommodation that you live in. ~F680~ was collected from participants except those who indicated they were living in a sheltered accommodation or in a care home, as defined by their answers to ~F670~-Variable type:binary</td></tr>
<tr><td>binary.680_2.txt</td><td>1710174270056F5forCTG.txt.gz</td><td>-0.001251</td><td>0.05662</td><td>-0.02209</td><td>0.9824</td><td>0.0126</td><td>0.001916</td><td>0.989</td><td>0.01108</td><td>1.84E-05</td><td>0.008104</td><td>Own or rent accommodation lived in: Own with a mortgage</td><td>FALSE</td><td></td><td></td><td></td><td>356340</td><td>131233</td><td>225107</td><td>UK Biobank</td><td>https://docs.google.com/spreadsheets/d/1kvPoupSzsSFBNSztMzl04xMoSC3Kcx3CrjVf4yBmESU/edit?ts=5b5f17db#gid=227859291</td><td>PHESANT Transformation:680_0 | | CAT-SINGLE | | CAT-SINGLE-BINARY-VAR: 2 | | Inc(>=10): 2(131233) | | -Notes:ACE touchscreen question Do you own or rent the accommodation that you live in? If the participant activated the Help button they were shown the message: Please select: - Own outright if you or someone in your household owns the accommodation that you live in. - Own with mortagage if you or someone in your household has a mortagage on the accommodation that you live in. ~F680~ was collected from participants except those who indicated they were living in a sheltered accommodation or in a care home, as defined by their answers to ~F670~-Variable type:binary</td></tr>
<tr><td>binary.680_3.txt</td><td>1710174270056F5forCTG.txt.gz</td><td>0.3934</td><td>0.03868</td><td>10.17</td><td>2.68E-24</td><td>0.03387</td><td>0.002231</td><td>1.017</td><td>0.01113</td><td>0.006109</td><td>0.00846</td><td>Own or rent accommodation lived in: Rent - from local authority, local council, housing association</td><td>FALSE</td><td>Wellbeing</td><td></td><td></td><td>356340</td><td>18968</td><td>337372</td><td>UK Biobank</td><td>https://docs.google.com/spreadsheets/d/1kvPoupSzsSFBNSztMzl04xMoSC3Kcx3CrjVf4yBmESU/edit?ts=5b5f17db#gid=227859291</td><td>PHESANT Transformation:680_0 | | CAT-SINGLE | | CAT-SINGLE-BINARY-VAR: 3 | | Inc(>=10): 3(18968) | | -Notes:ACE touchscreen question Do you own or rent the accommodation that you live in? If the participant activated the Help button they were shown the message: Please select: - Own outright if you or someone in your household owns the accommodation that you live in. - Own with mortagage if you or someone in your household has a mortagage on the accommodation that you live in. ~F680~ was collected from participants except those who indicated they were living in a sheltered accommodation or in a care home, as defined by their answers to ~F670~-Variable type:binary</td></tr>
<tr><td>binary.680_4.txt</td><td>1710174270056F5forCTG.txt.gz</td><td>0.2177</td><td>0.08036</td><td>2.708</td><td>0.00676</td><td>0.005812</td><td>0.001561</td><td>1.009</td><td>0.009192</td><td>-0.003127</td><td>0.008315</td><td>Own or rent accommodation lived in: Rent - from private landlord or letting agency</td><td>FALSE</td><td></td><td></td><td></td><td>356340</td><td>9453</td><td>346887</td><td>UK Biobank</td><td>https://docs.google.com/spreadsheets/d/1kvPoupSzsSFBNSztMzl04xMoSC3Kcx3CrjVf4yBmESU/edit?ts=5b5f17db#gid=227859291</td><td>PHESANT Transformation:680_0 | | CAT-SINGLE | | CAT-SINGLE-BINARY-VAR: 4 | | Inc(>=10): 4(9453) | | -Notes:ACE touchscreen question Do you own or rent the accommodation that you live in? If the participant activated the Help button they were shown the message: Please select: - Own outright if you or someone in your household owns the accommodation that you live in. - Own with mortagage if you or someone in your household has a mortagage on the accommodation that you live in. ~F680~ was collected from participants except those who indicated they were living in a sheltered accommodation or in a care home, as defined by their answers to ~F670~-Variable type:binary</td></tr>
</table>

binary.680_5.txt	1710174270056F5 forCTG.txt.gz	0.05967	0.09323	0.64	0.5222	0.003768	0.001469	0.989	0.009272	0.002027	0.007112	Own or rent accommodation lived in: Pay part rent and part mortgage (shared ownership)	FALSE				356340	919	355421	UK Biobank	https://docs.google.com/spreadsheets/d/1kvPoupSzsSFBNSztMzl04xMoSC3Kcx3CrjVf4yBmESU/edit?ts=5b5f17db#gid=227859291	PHESANT Transformation:680_0 | | CAT-SINGLE | | CAT-SINGLE-BINARY-VAR: 5 | | Inc(>=10): 5(919) | |-Notes:ACE touchscreen question Do you own or rent the accommodation that you live in? If the participant activated the Help button they were shown the message: Please select: - Own outright if you or someone in your household owns the accommodation that you live in. - Own with mortagage if you or someone in your household has a mortagage on the accommodation that you live in. ~F680~ was collected from participants except those who indicated they were living in a sheltered accommodation or in a care home, as defined by their answers to ~F670~-Variable type:binary
Biobank2-British-Bmd-As-C-Gwas-SumStats.txt	1710174270056F5 forCTG.txt.gz	0.05107	0.02	2.553	0.01067	0.4185	0.03943	1.208	0.1334	-0.01246	0.01426	Bone mineral density	FALSE				426824			GEFOS	http://www.gefos.org/?q=content/data-release-2018	The GEnetic Factors for OSteoporosis (GEFOS) Consortium is a large international collaboration comprising numerous research groups. Osteoporosis is a common age-related complex disease with a strong genetic component. The UK Biobank is a health resource from the United Kingdom that has genetic data and phenotype measurements in 500,000 individuals, representative of the general population. We analyzed the full batch of genetic data, including genotyping and imputed data in up to 426,824 participants. We performed genome-wide association studies on bone mineral density estimated from quantitative heel ultrasounds (eBMD) and from bone fractures.
Biobank2-British-FracA-As-C-Gwas-SumStats.txt	1710174270056F5 forCTG.txt.gz	0.17	0.03974	4.278	1.88E-05	0.02445	0.002521	1.008	0.01303	0.02098	0.008571	Bone fractures	TRUE	Skeletal	Physical health	Bone fractures	426824			GEFOS	http://www.gefos.org/?q=content/data-release-2018	The GEnetic Factors for OSteoporosis (GEFOS) Consortium is a large international collaboration comprising numerous research groups. Osteoporosis is a common age-related complex disease with a strong genetic component. The UK Biobank is a health resource from the United Kingdom that has genetic data and phenotype measurements in 500,000 individuals, representative of the general population. We analyzed the full batch of genetic data, including genotyping and imputed data in up to 426,824 participants. We performed genome-wide association studies on bone mineral density estimated from quantitative heel ultrasounds (eBMD) and from bone fractures.
biomarkers-30600-both_sexes-irnt.tsv.bgz	1710174270056F5 forCTG.txt.gz	-0.06104	0.02594	-2.353	0.01862	0.112	0.0121	1.3	0.08876	-0.008457	0.01086	Albumin	FALSE				367192	367192		UK Biobank (EUR)	https://pan-ukb-us-east-1.s3.amazonaws.com/sumstats_flat_files/biomarkers-30600-both_sexes-irnt.tsv.bgz	EUR: biomarkers : 30600 : Biological samples > Assay results > Blood assays > Blood biochemistry
biomarkers-30610-both_sexes-irnt.tsv.bgz	1710174270056F5 forCTG.txt.gz	0.1139	0.03296	3.456	0.000548	0.1918	0.03846	1.716	0.3117	0.01575	0.01574	Alkaline phosphatase	FALSE				400988	400988		UK Biobank (EUR)	https://pan-ukb-us-east-1.s3.amazonaws.com/sumstats_flat_files/biomarkers-30610-both_sexes-irnt.tsv.bgz	EUR: biomarkers : 30610 : Biological samples > Assay results > Blood assays > Blood biochemistry
biomarkers-30620-both_sexes-irnt.tsv.bgz	1710174270056F5 forCTG.txt.gz	0.1764	0.03088	5.712	1.12E-08	0.1214	0.0123	1.191	0.05017	-0.01324	0.01183	Alanine aminotransferase	FALSE	Metabolic			400822	400822		UK Biobank (EUR)	https://pan-ukb-us-east-1.s3.amazonaws.com/sumstats_flat_files/biomarkers-30620-both_sexes-irnt.tsv.bgz	EUR: biomarkers : 30620 : Biological samples > Assay results > Blood assays > Blood biochemistry
biomarkers-30630-both_sexes-irnt.tsv.bgz	1710174270056F5 forCTG.txt.gz	-0.1637	0.03854	-4.246	2.18E-05	0.132	0.03481	1.768	0.2709	-0.01197	0.01428	Apolipoprotein A	FALSE	Metabolic			364987	364987		UK Biobank (EUR)	https://pan-ukb-us-east-1.s3.amazonaws.com/sumstats_flat_files/biomarkers-30630-both_sexes-irnt.tsv.bgz	EUR: biomarkers : 30630 : Biological samples > Assay results > Blood assays > Blood biochemistry
biomarkers-30640-both_sexes-irnt.tsv.bgz	1710174270056F5 forCTG.txt.gz	0.1481	0.08599	1.722	0.08505	0.04767	0.04943	1.731	0.4308	-0.01651	0.01686	Apolipoprotein B	FALSE				399003	399003		UK Biobank (EUR)	https://pan-ukb-us-east-1.s3.amazonaws.com/sumstats_flat_files/biomarkers-30640-both_sexes-irnt.tsv.bgz	EUR: biomarkers : 30640 : Biological samples > Assay results > Blood assays > Blood biochemistry
biomarkers-30650-both_sexes-irnt.tsv.bgz	1710174270056F5 forCTG.txt.gz	0.0748	0.03007	2.487	0.01287	0.1287	0.01385	1.295	0.06983	-0.008692	0.01196	Aspartate aminotransferase	FALSE				399482	399482		UK Biobank (EUR)	https://pan-ukb-us-east-1.s3.amazonaws.com/sumstats_flat_files/biomarkers-30650-both_sexes-irnt.tsv.bgz	EUR: biomarkers : 30650 : Biological samples > Assay results > Blood assays > Blood biochemistry
biomarkers-30660-both_sexes-irnt.tsv.bgz	1710174270056F5 forCTG.txt.gz	-0.04309	0.04527	-0.9519	0.3412	0.1211	0.05998	1.203	0.05667	0.005707	0.01209	Direct bilirubin	FALSE				340934	340934		UK Biobank (EUR)	https://pan-ukb-us-east-1.s3.amazonaws.com/sumstats_flat_files/biomarkers-30660-both_sexes-irnt.tsv.bgz	EUR: biomarkers : 30660 : Biological samples > Assay results > Blood assays > Blood biochemistry
biomarkers-30670-both_sexes-irnt.tsv.bgz	1710174270056F5 forCTG.txt.gz	0.001258	0.03068	0.041	0.9673	0.1053	0.009294	1.221	0.04706	-0.000345	0.01227	Urea	FALSE				400687	400687		UK Biobank (EUR)	https://pan-ukb-us-east-1.s3.amazonaws.com/sumstats_flat_files/biomarkers-30670-both_sexes-irnt.tsv.bgz	EUR: biomarkers : 30670 : Biological samples > Assay results > Blood assays > Blood biochemistry
biomarkers-30680-both_sexes-irnt.tsv.bgz	1710174270056F5 forCTG.txt.gz	0.02072	0.02895	0.7155	0.4743	0.1126	0.01324	1.208	0.04619	0.00073	0.01128	Calcium	FALSE				367050	367050		UK Biobank (EUR)	https://pan-ukb-us-east-1.s3.amazonaws.com/sumstats_flat_files/biomarkers-30680-both_sexes-irnt.tsv.bgz	EUR: biomarkers : 30680 : Biological samples > Assay results > Blood assays > Blood biochemistry
biomarkers-30690-both_sexes-irnt.tsv.bgz	1710174270056F5 forCTG.txt.gz	-0.005007	0.04002	-0.1251	0.9004	0.07104	0.03857	1.556	0.3076	-0.01308	0.0158	Cholesterol	FALSE				400963	400963		UK Biobank (EUR)	https://pan-ukb-us-east-1.s3.amazonaws.com/sumstats_flat_files/biomarkers-30690-both_sexes-irnt.tsv.bgz	EUR: biomarkers : 30690 : Biological samples > Assay results > Blood assays > Blood biochemistry
biomarkers-30700-both_sexes-irnt.tsv.bgz	1710174270056F5 forCTG.txt.gz	-0.0204	0.02708	-0.7535	0.4511	0.2019	0.01867	1.316	0.09502	0.02686	0.01324	Creatinine	FALSE				400761	400761		UK Biobank (EUR)	https://pan-ukb-us-east-1.s3.amazonaws.com/sumstats_flat_files/biomarkers-30700-both_sexes-irnt.tsv.bgz	EUR: biomarkers : 30700 : Biological samples > Assay results > Blood assays > Blood biochemistry
biomarkers-30710-both_sexes-irnt.tsv.bgz	1710174270056F5 forCTG.txt.gz	0.1986	0.05874	3.382	0.00072	0.15	0.04454	1.441	0.2485	0.02471	0.01678	C-reactive protein	FALSE				400094	400094		UK Biobank (EUR)	https://pan-ukb-us-east-1.s3.amazonaws.com/sumstats_flat_files/biomarkers-30710-both_sexes-irnt.tsv.bgz	EUR: biomarkers : 30710 : Biological samples > Assay results > Blood assays > Blood biochemistry
biomarkers-30720-both_sexes-irnt.tsv.bgz	1710174270056F5 forCTG.txt.gz	0.114	0.02723	4.186	2.84E-05	0.235	0.03532	1.429	0.09603	0.03224	0.01326	Cystatin C	FALSE	Other (physical health)			400940	400940		UK Biobank (EUR)	https://pan-ukb-us-east-1.s3.amazonaws.com/sumstats_flat_files/biomarkers-30720-both_sexes-irnt.tsv.bgz	EUR: biomarkers : 30720 : Biological samples > Assay results > Blood assays > Blood biochemistry
biomarkers-30730-both_sexes-irnt.tsv.bgz	1710174270056F5 forCTG.txt.gz	0.146	0.0404	3.613	0.000302	0.1808	0.0239	1.439	0.1003	-0.00653	0.0145	Gamma glutamyltransferase	FALSE				400751	400751		UK Biobank (EUR)	https://pan-ukb-us-east-1.s3.amazonaws.com/sumstats_flat_files/biomarkers-30730-both_sexes-irnt.tsv.bgz	EUR: biomarkers : 30730 : Biological samples > Assay results > Blood assays > Blood biochemistry
biomarkers-30740-both_sexes-irnt.tsv.bgz	1710174270056F5 forCTG.txt.gz	0.06321	0.03506	1.803	0.07139	0.07332	0.01756	1.122	0.05434	-0.000646	0.00929	Glucose	FALSE				366759	366759		UK Biobank (EUR)	https://pan-ukb-us-east-1.s3.amazonaws.com/sumstats_flat_files/biomarkers-30740-both_sexes-irnt.tsv.bgz	EUR: biomarkers : 30740 : Biological samples > Assay results > Blood assays > Blood biochemistry
biomarkers-30750-both_sexes-irnt.tsv.bgz	1710174270056F5 forCTG.txt.gz	0.1083	0.03051	3.551	0.000384	0.1783	0.02758	1.495	0.1723	0.006451	0.01348	Glycated haemoglobin (HbA1c)	FALSE				400825	400825		UK Biobank (EUR)	https://pan-ukb-us-east-1.s3.amazonaws.com/sumstats_flat_files/biomarkers-30750-both_sexes-irnt.tsv.bgz	EUR: biomarkers : 30750 : Biological samples > Assay results > Blood assays > Blood biochemistry
biomarkers-30760-both_sexes-irnt.tsv.bgz	1710174270056F5 forCTG.txt.gz	-0.185	0.03414	-5.417	6.06E-08	0.1696	0.03888	1.936	0.304	-0.02146	0.01413	HDL cholesterol	FALSE	Metabolic			367021	367021		UK Biobank (EUR)	https://pan-ukb-us-east-1.s3.amazonaws.com/sumstats_flat_files/biomarkers-30760-both_sexes-irnt.tsv.bgz	EUR: biomarkers : 30760 : Biological samples > Assay results > Blood assays > Blood biochemistry
biomarkers-30770-both_sexes-irnt.tsv.bgz	1710174270056F5 forCTG.txt.gz	-0.05715	0.02805	-2.037	0.04162	0.2213	0.01926	1.368	0.08236	-0.01517	0.01414	IGF-1	FALSE				398797	398797		UK Biobank (EUR)	https://pan-ukb-us-east-1.s3.amazonaws.com/sumstats_flat_files/biomarkers-30770-both_sexes-irnt.tsv.bgz	EUR: biomarkers : 30770 : Biological samples > Assay results > Blood assays > Blood biochemistry
biomarkers-30780-both_sexes-irnt.tsv.bgz	1710174270056F5 forCTG.txt.gz	0.05488	0.05879	0.9335	0.3506	0.04975	0.04143	1.534	0.3481	-0.01253	0.01602	LDL direct	FALSE				400223	400223		UK Biobank (EUR)	https://pan-ukb-us-east-1.s3.amazonaws.com/sumstats_flat_files/biomarkers-30780-both_sexes-irnt.tsv.bgz	EUR: biomarkers : 30780 : Biological samples > Assay results > Blood assays > Blood biochemistry
biomarkers-30800-both_sexes-irnt.tsv.bgz	1710174270056F5 forCTG.txt.gz	-0.2245	0.1586	-1.416	0.1569	0.01114	0.008307	1.008	0.008889	0.005251	0.007516	Oestradiol	FALSE				63332	63332		UK Biobank (EUR)	https://pan-ukb-us-east-1.s3.amazonaws.com/sumstats_flat_files/biomarkers-30800-both_sexes-irnt.tsv.bgz	EUR: biomarkers : 30800 : Biological samples > Assay results > Blood assays > Blood biochemistry
biomarkers-30810-both_sexes-irnt.tsv.bgz	1710174270056F5 forCTG.txt.gz	0.01706	0.0331	0.5153	0.6063	0.09161	0.01272	1.281	0.09875	-0.01332	0.01173	Phosphate	FALSE				366484	366484		UK Biobank (EUR)	https://pan-ukb-us-east-1.s3.amazonaws.com/sumstats_flat_files/biomarkers-30810-both_sexes-irnt.tsv.bgz	EUR: biomarkers : 30810 : Biological samples > Assay results > Blood assays > Blood biochemistry
biomarkers-30820-both_sexes-irnt.tsv.bgz	1710174270056F5 forCTG.txt.gz	0.09404	0.1371	0.686	0.4927	0.01949	0.014	0.99	0.008554	-0.001194	0.00828	Rheumatoid factor	FALSE				35667	35667		UK Biobank (EUR)	https://pan-ukb-us-east-1.s3.amazonaws.com/sumstats_flat_files/biomarkers-30820-both_sexes-irnt.tsv.bgz	EUR: biomarkers : 30820 : Biological samples > Assay results > Blood assays > Blood biochemistry
biomarkers-30830-both_sexes-irnt.tsv.bgz	1710174270056F5 forCTG.txt.gz	-0.126	0.02905	-4.337	1.45E-05	0.1854	0.02481	1.448	0.1919	-0.000594	0.01359	SHBG	FALSE	Reproductive			363650	363650		UK Biobank (EUR)	https://pan-ukb-us-east-1.s3.amazonaws.com/sumstats_flat_files/biomarkers-30830-both_sexes-irnt.tsv.bgz	EUR: biomarkers : 30830 : Biological samples > Assay results > Blood assays > Blood biochemistry
biomarkers-30840-both_sexes-irnt.tsv.bgz	1710174270056F5 forCTG.txt.gz	-0.07613	0.04506	-1.69	0.09108	0.1456	0.05839	1.302	0.07799	-0.000483	0.01263	Total bilirubin	FALSE				399286	399286		UK Biobank (EUR)	https://pan-ukb-us-east-1.s3.amazonaws.com/sumstats_flat_files/biomarkers-30840-both_sexes-irnt.tsv.bgz	EUR: biomarkers : 30840 : Biological samples > Assay results > Blood assays > Blood biochemistry
biomarkers-30850-both_sexes-irnt.tsv.bgz	1710174270056F5 forCTG.txt.gz	0.004731	0.03302	0.1433	0.8861	0.07207	0.01069	1.124	0.06575	-0.01289	0.009978	Testosterone	FALSE				363203	363203		UK Biobank (EUR)	https://pan-ukb-us-east-1.s3.amazonaws.com/sumstats_flat_files/biomarkers-30850-both_sexes-irnt.tsv.bgz	EUR: biomarkers : 30850 : Biological samples > Assay results > Blood assays > Blood biochemistry

biomarkers-30860-both_sexes-irnt.tsv.bgz	1710174270056F5forCTG.txt.gz	0.02646	0.03058	0.8655	0.3868	0.119	0.01008	1.362	0.07843	-0.001961	0.01241	Total protein	FALSE					366758	366758		UK Biobank (EUR)	https://pan-ukb-us-east-1.s3.amazonaws.com/sumstats_flat_files/biomarkers-30860-both_sexes-irnt.tsv.bgz	EUR: biomarkers : 30860 : Biological samples > Assay results > Blood assays > Blood biochemistry
biomarkers-30870-both_sexes-irnt.tsv.bgz	1710174270056F5forCTG.txt.gz	0.1375	0.02755	4.99	6.03E-07	0.1796	0.0381	1.497	0.1964	0.03568	0.0148	Triglycerides	FALSE	Metabolic				400639	400639		UK Biobank (EUR)	https://pan-ukb-us-east-1.s3.amazonaws.com/sumstats_flat_files/biomarkers-30870-both_sexes-irnt.tsv.bgz	EUR: biomarkers : 30870 : Biological samples > Assay results > Blood assays > Blood biochemistry
biomarkers-30880-both_sexes-irnt.tsv.bgz	1710174270056F5forCTG.txt.gz	0.1062	0.02361	4.497	6.89E-06	0.1975	0.04386	1.217	0.1457	0.02063	0.01241	Urate	FALSE	Metabolic				400469	400469		UK Biobank (EUR)	https://pan-ukb-us-east-1.s3.amazonaws.com/sumstats_flat_files/biomarkers-30880-both_sexes-irnt.tsv.bgz	EUR: biomarkers : 30880 : Biological samples > Assay results > Blood assays > Blood biochemistry
biomarkers-30890-both_sexes-irnt.tsv.bgz	1710174270056F5forCTG.txt.gz	-0.01495	0.02873	-0.5203	0.6028	0.08635	0.02288	1.131	0.07302	-0.01775	0.01127	Vitamin D	FALSE					383324	383324		UK Biobank (EUR)	https://pan-ukb-us-east-1.s3.amazonaws.com/sumstats_flat_files/biomarkers-30890-both_sexes-irnt.tsv.bgz	EUR: biomarkers : 30890 : Biological samples > Assay results > Blood assays > Blood biochemistry
bNGF.data.freq.final	1710174270056F5forCTG.txt.gz	-0.02579	0.1327	-0.1943	0.8459	0.08072	0.07087	0.995	0.01027	0.01059	0.008038	Basic Nerve Growth Factor	FALSE					8293			Young Finns Study (YFS)	http://www.computationalmedicine.fi/data	Genome-wide Association Study Identifies 27 Loci Influencing Concentrations of Circulating Cytokines and Growth Factors
categorical.AB1_INFECTIONS.txt	1710174270056F5forCTG.txt.gz	0.6886	0.2428	2.836	0.004561	0.00247	0.001492	1.002	0.009151	-0.003523	0.00833	Certain infectious and parasitic diseases	FALSE					361194	7530	353664	UK Biobank	https://docs.google.com/spreadsheets/d/1kvPoupSzsSFBNSztMzl04xMoSC3Kcx3CrjVf4yBmESU/edit?ts=5b5f17db#gid=227859291	PHESANT Transformation:NA-Notes:NA-Variable type:categorical-Phenotype ID:AB1_INFECTIONS
categorical.ASTHMA_CHILD.txt	1710174270056F5forCTG.txt.gz	0.3849	0.107	3.599	0.00032	0.004465	0.001434	1.003	0.008651	-0.005072	0.007496	Childhood asthma (age<16)	FALSE					361194	1993	359201	UK Biobank	https://docs.google.com/spreadsheets/d/1kvPoupSzsSFBNSztMzl04xMoSC3Kcx3CrjVf4yBmESU/edit?ts=5b5f17db#gid=227859291	PHESANT Transformation:NA-Notes:NA-Variable type:categorical-Phenotype ID:ASTHMA_CHILD
categorical.ASTHMA_EOSINOPHIL_SUGG.txt	1710174270056F5forCTG.txt.gz	0.03301	0.07791	0.4237	0.6718	0.006532	0.002121	1.014	0.0114	-0.007741	0.008449	Suggestive for eosinophilic asthma	FALSE					361194	2302	358892	UK Biobank	https://docs.google.com/spreadsheets/d/1kvPoupSzsSFBNSztMzl04xMoSC3Kcx3CrjVf4yBmESU/edit?ts=5b5f17db#gid=227859291	PHESANT Transformation:NA-Notes:NA-Variable type:categorical-Phenotype ID:ASTHMA_EOSINOPHIL_SUGG
categorical.ASTHMA_HOSPITAL1.txt	1710174270056F5forCTG.txt.gz	0.3793	0.1062	3.57	0.000356	0.004467	0.001445	1.003	0.008683	-0.004448	0.007559	Asthma, hospital admissions 1	FALSE					361194	1986	359208	UK Biobank	https://docs.google.com/spreadsheets/d/1kvPoupSzsSFBNSztMzl04xMoSC3Kcx3CrjVf4yBmESU/edit?ts=5b5f17db#gid=227859291	PHESANT Transformation:NA-Notes:NA-Variable type:categorical-Phenotype ID:ASTHMA_HOSPITAL1
categorical.ASTHMA_MEDICATIO_COMORB.txt	1710174270056F5forCTG.txt.gz	0.173	0.0564	3.067	0.002163	0.01308	0.00199	1.011	0.01149	-0.01356	0.009052	Medication related adverse effects	FALSE					361194	20094	341100	UK Biobank	https://docs.google.com/spreadsheets/d/1kvPoupSzsSFBNSztMzl04xMoSC3Kcx3CrjVf4yBmESU/edit?ts=5b5f17db#gid=227859291	PHESANT Transformation:NA-Notes:NA-Variable type:categorical-Phenotype ID:ASTHMA_MEDICATIO_COMORB
categorical.ASTHMA_OPPORTUNIST_INFECTIONS.txt	1710174270056F5forCTG.txt.gz	0.009288	0.09359	0.09924	0.9209	0.004159	0.001693	0.984	0.009692	0.01058	0.008871	NA	FALSE					361194	509	360685	UK Biobank	https://docs.google.com/spreadsheets/d/1kvPoupSzsSFBNSztMzl04xMoSC3Kcx3CrjVf4yBmESU/edit?ts=5b5f17db#gid=227859291	PHESANT Transformation:NA-Notes:NA-Variable type:categorical-Phenotype ID:ASTHMA_OPPORTUNIST_INFECTIONS
categorical.ASTHMA_PNEUMONIA.txt	1710174270056F5forCTG.txt.gz	0.5908	0.2436	2.425	0.0153	0.00194	0.001412	1.007	0.008891	0.004398	0.007437	Asthma-related pneumonia	FALSE					361194	5900	355294	UK Biobank	https://docs.google.com/spreadsheets/d/1kvPoupSzsSFBNSztMzl04xMoSC3Kcx3CrjVf4yBmESU/edit?ts=5b5f17db#gid=227859291	PHESANT Transformation:NA-Notes:NA-Variable type:categorical-Phenotype ID:ASTHMA_PNEUMONIA
categorical.C_BREAST_3.txt	1710174270056F5forCTG.txt.gz	0.0546	0.05896	0.926	0.3545	0.0124	0.002403	1.029	0.0154	-0.01199	0.008261	Malignant neoplasm of breast	FALSE					361194	9721	351473	UK Biobank	https://docs.google.com/spreadsheets/d/1kvPoupSzsSFBNSztMzl04xMoSC3Kcx3CrjVf4yBmESU/edit?ts=5b5f17db#gid=227859291	PHESANT Transformation:NA-Notes:NA-Variable type:categorical-Phenotype ID:C_BREAST_3
categorical.C_BRONCHUS_LUNG.txt	1710174270056F5forCTG.txt.gz	0.05589	0.08033	0.6958	0.4866	0.00584	0.001577	0.983	0.009375	0.01554	0.008084	Malignant neoplasm of bronchus and lung	FALSE					361194	1681	359513	UK Biobank	https://docs.google.com/spreadsheets/d/1kvPoupSzsSFBNSztMzl04xMoSC3Kcx3CrjVf4yBmESU/edit?ts=5b5f17db#gid=227859291	PHESANT Transformation:NA-Notes:NA-Variable type:categorical-Phenotype ID:C_BRONCHUS_LUNG
categorical.C_COLON.txt	1710174270056F5forCTG.txt.gz	0.08463	0.0957	0.8843	0.3765	0.003904	0.001769	1.011	0.01061	-0.002997	0.008066	Malignant neoplasm of colon	FALSE					361194	2437	358757	UK Biobank	https://docs.google.com/spreadsheets/d/1kvPoupSzsSFBNSztMzl04xMoSC3Kcx3CrjVf4yBmESU/edit?ts=5b5f17db#gid=227859291	PHESANT Transformation:NA-Notes:NA-Variable type:categorical-Phenotype ID:C_COLON
categorical.C_DIGESTIVE_ORGANS.txt	1710174270056F5forCTG.txt.gz	0.09495	0.08304	1.143	0.2528	0.004854	0.00167	1.011	0.01053	0.004089	0.007358	NA	FALSE					361194	5690	355504	UK Biobank	https://docs.google.com/spreadsheets/d/1kvPoupSzsSFBNSztMzl04xMoSC3Kcx3CrjVf4yBmESU/edit?ts=5b5f17db#gid=227859291	PHESANT Transformation:NA-Notes:NA-Variable type:categorical-Phenotype ID:C_DIGESTIVE_ORGANS
categorical.C_MALE_GENITAL.txt	1710174270056F5forCTG.txt.gz	-0.008092	0.06034	-0.1341	0.8933	0.01036	0.002398	1.036	0.02089	-0.000472	0.007852	NA	FALSE					361194	6795	354399	UK Biobank	https://docs.google.com/spreadsheets/d/1kvPoupSzsSFBNSztMzl04xMoSC3Kcx3CrjVf4yBmESU/edit?ts=5b5f17db#gid=227859291	PHESANT Transformation:NA-Notes:NA-Variable type:categorical-Phenotype ID:C_MALE_GENITAL
categorical.C_MELANOMA_SKIN.txt	1710174270056F5forCTG.txt.gz	0.0557	0.1147	0.4858	0.6271	0.002993	0.00209	1.018	0.01139	-0.01288	0.008141	Malignant melanoma of skin	FALSE					361194	2534	358660	UK Biobank	https://docs.google.com/spreadsheets/d/1kvPoupSzsSFBNSztMzl04xMoSC3Kcx3CrjVf4yBmESU/edit?ts=5b5f17db#gid=227859291	PHESANT Transformation:NA-Notes:NA-Variable type:categorical-Phenotype ID:C_MELANOMA_SKIN
categorical.C_MESOTHELIOMA.txt	1710174270056F5forCTG.txt.gz	0.09296	0.172	0.5406	0.5888	0.001523	0.00163	1.005	0.01105	0.001248	0.009303	Mesothelioma	FALSE					361194	165	361029	UK Biobank	https://docs.google.com/spreadsheets/d/1kvPoupSzsSFBNSztMzl04xMoSC3Kcx3CrjVf4yBmESU/edit?ts=5b5f17db#gid=227859291	PHESANT Transformation:NA-Notes:NA-Variable type:categorical-Phenotype ID:C_MESOTHELIOMA
categorical.C_OTHER_SKIN.txt	1710174270056F5forCTG.txt.gz	0.03071	0.03953	0.7768	0.4373	0.02783	0.00499	1.019	0.02202	-0.008228	0.008277	Other malignant neoplasms of skin	FALSE					361194	14402	346792	UK Biobank	https://docs.google.com/spreadsheets/d/1kvPoupSzsSFBNSztMzl04xMoSC3Kcx3CrjVf4yBmESU/edit?ts=5b5f17db#gid=227859291	PHESANT Transformation:NA-Notes:NA-Variable type:categorical-Phenotype ID:C_OTHER_SKIN
categorical.C_PROSTATE.txt	1710174270056F5forCTG.txt.gz	-0.008035	0.06123	-0.1312	0.8956	0.01022	0.0025	1.044	0.02186	0.0003051	0.007868	Malignant neoplasm of prostate	FALSE					361194	6321	354873	UK Biobank	https://docs.google.com/spreadsheets/d/1kvPoupSzsSFBNSztMzl04xMoSC3Kcx3CrjVf4yBmESU/edit?ts=5b5f17db#gid=227859291	PHESANT Transformation:NA-Notes:NA-Variable type:categorical-Phenotype ID:C_PROSTATE
categorical.C_RESPIRATORY_INTRATHORACIC.txt	1710174270056F5forCTG.txt.gz	0.06495	0.08501	0.764	0.4448	0.004508	0.001598	0.986	0.009571	0.01525	0.007867	NA	FALSE					361194	1944	359250	UK Biobank	https://docs.google.com/spreadsheets/d/1kvPoupSzsSFBNSztMzl04xMoSC3Kcx3CrjVf4yBmESU/edit?ts=5b5f17db#gid=227859291	PHESANT Transformation:NA-Notes:NA-Variable type:categorical-Phenotype ID:C_RESPIRATORY_INTRATHORACIC
categorical.C_SKIN.txt	1710174270056F5forCTG.txt.gz	0.02982	0.04002	0.7451	0.4562	0.02647	0.005203	1.023	0.02213	-0.01152	0.008226	NA	FALSE					361194	16531	344663	UK Biobank	https://docs.google.com/spreadsheets/d/1kvPoupSzsSFBNSztMzl04xMoSC3Kcx3CrjVf4yBmESU/edit?ts=5b5f17db#gid=227859291	PHESANT Transformation:NA-Notes:NA-Variable type:categorical-Phenotype ID:C_SKIN
categorical.C_STROKE.txt	1710174270056F5forCTG.txt.gz	0.4637	0.2403	1.93	0.05363	0.001739	0.001417	1.014	0.008674	-0.004719	0.007799	STROKE	FALSE					361194	6146	355048	UK Biobank	https://docs.google.com/spreadsheets/d/1kvPoupSzsSFBNSztMzl04xMoSC3Kcx3CrjVf4yBmESU/edit?ts=5b5f17db#gid=227859291	PHESANT Transformation:NA-Notes:NA-Variable type:categorical-Phenotype ID:C_STROKE
categorical.C02.txt	1710174270056F5forCTG.txt.gz	-0.02068	0.111	-0.1863	0.8522	0.002838	0.001616	0.994	0.009654	0.005868	0.009039	Diagnoses - main ICD10: C02 Malignant neoplasm of other and unspecified parts of tongue	FALSE					361194	152	361042	UK Biobank	https://docs.google.com/spreadsheets/d/1kvPoupSzsSFBNSztMzl04xMoSC3Kcx3CrjVf4yBmESU/edit?ts=5b5f17db#gid=227859291	PHESANT Transformation:NA-Notes:NA-Variable type:categorical-Phenotype ID:C02
categorical.C15.txt	1710174270056F5forCTG.txt.gz	0.1261	0.1081	1.166	0.2434	0.003106	0.001436	1.001	0.008783	-0.000241	0.007836	Diagnoses - main ICD10: C15 Malignant neoplasm of oesophagus	FALSE					361194	519	360675	UK Biobank	https://docs.google.com/spreadsheets/d/1kvPoupSzsSFBNSztMzl04xMoSC3Kcx3CrjVf4yBmESU/edit?ts=5b5f17db#gid=227859291	PHESANT Transformation:NA-Notes:NA-Variable type:categorical-Phenotype ID:C15
categorical.C18.txt	1710174270056F5forCTG.txt.gz	0.02562	0.1051	0.2438	0.8074	0.003232	0.001695	1.017	0.01014	0.002441	0.007909	Diagnoses - main ICD10: C18 Malignant neoplasm of colon	FALSE					361194	2226	358968	UK Biobank	https://docs.google.com/spreadsheets/d/1kvPoupSzsSFBNSztMzl04xMoSC3Kcx3CrjVf4yBmESU/edit?ts=5b5f17db#gid=227859291	PHESANT Transformation:NA-Notes:NA-Variable type:categorical-Phenotype ID:C18
categorical.C25.txt	1710174270056F5forCTG.txt.gz	-0.07291	0.09306	-0.7835	0.4333	0.004124	0.001527	0.968	0.009616	0.01038	0.008231	Diagnoses - main ICD10: C25 Malignant neoplasm of pancreas	FALSE					361194	403	360791	UK Biobank	https://docs.google.com/spreadsheets/d/1kvPoupSzsSFBNSztMzl04xMoSC3Kcx3CrjVf4yBmESU/edit?ts=5b5f17db#gid=227859291	PHESANT Transformation:NA-Notes:NA-Variable type:categorical-Phenotype ID:C25
categorical.C3_BREAST_3.txt	1710174270056F5forCTG.txt.gz	0.0546	0.05896	0.926	0.3545	0.0124	0.002403	1.029	0.0154	-0.01199	0.008261	Malignant neoplasm of breast	FALSE					361194	9721	351473	UK Biobank	https://docs.google.com/spreadsheets/d/1kvPoupSzsSFBNSztMzl04xMoSC3Kcx3CrjVf4yBmESU/edit?ts=5b5f17db#gid=227859291	PHESANT Transformation:NA-Notes:NA-Variable type:categorical-Phenotype ID:C3_BREAST_3

categorical.C3_BRONCHUS_LUNG.txt	1710174270056F5forCTG.txt.gz	0.05589	0.08033	0.6958	0.4866	0.00584	0.001577	0.983	0.009375	0.01554	0.008084	Malignant neoplasm of bronchus and lung	FALSE					361194	1681	359513	UK Biobank	https://docs.google.com/spreadsheets/d/1kvPoupSzsSFBNSztMzl04xMoSC3Kcx3CrjVf4yBmESU/edit?ts=5b5f17db#gid=227859291	PHESANT Transformation:NA-Notes:NA-Variable type:categorical-Phenotype ID:C3_BRONCHUS_LUNG
categorical.C3_COLON.txt	1710174270056F5forCTG.txt.gz	0.08463	0.0957	0.8843	0.3765	0.003904	0.001769	1.011	0.01061	-0.002997	0.008066	Malignant neoplasm of colon	FALSE					361194	2437	358757	UK Biobank	https://docs.google.com/spreadsheets/d/1kvPoupSzsSFBNSztMzl04xMoSC3Kcx3CrjVf4yBmESU/edit?ts=5b5f17db#gid=227859291	PHESANT Transformation:NA-Notes:NA-Variable type:categorical-Phenotype ID:C3_COLON
categorical.C3_DIGESTIVE_ORGANS.txt	1710174270056F5forCTG.txt.gz	0.09495	0.08304	1.143	0.2528	0.004854	0.00167	1.011	0.01053	0.004089	0.007358	Malignant neoplasm of digestive organs	FALSE					361194	5690	355504	UK Biobank	https://docs.google.com/spreadsheets/d/1kvPoupSzsSFBNSztMzl04xMoSC3Kcx3CrjVf4yBmESU/edit?ts=5b5f17db#gid=227859291	PHESANT Transformation:NA-Notes:NA-Variable type:categorical-Phenotype ID:C3_DIGESTIVE_ORGANS
categorical.C3_MALE_GENITAL.txt	1710174270056F5forCTG.txt.gz	-0.008092	0.06034	-0.1341	0.8933	0.01036	0.002398	1.036	0.02089	-0.000472	0.007852	malignant neoplasm of male genital organs	FALSE					361194	6795	354399	UK Biobank	https://docs.google.com/spreadsheets/d/1kvPoupSzsSFBNSztMzl04xMoSC3Kcx3CrjVf4yBmESU/edit?ts=5b5f17db#gid=227859291	PHESANT Transformation:NA-Notes:NA-Variable type:categorical-Phenotype ID:C3_MALE_GENITAL
categorical.C3_MELANOMA_SKIN.txt	1710174270056F5forCTG.txt.gz	0.0557	0.1147	0.4858	0.6271	0.002993	0.00209	1.018	0.01139	-0.01288	0.008141	Malignant melanoma of skin	FALSE					361194	2534	358660	UK Biobank	https://docs.google.com/spreadsheets/d/1kvPoupSzsSFBNSztMzl04xMoSC3Kcx3CrjVf4yBmESU/edit?ts=5b5f17db#gid=227859291	PHESANT Transformation:NA-Notes:NA-Variable type:categorical-Phenotype ID:C3_MELANOMA_SKIN
categorical.C3_MESOTHELIOMA.txt	1710174270056F5forCTG.txt.gz	0.09296	0.172	0.5406	0.5888	0.001523	0.00163	1.005	0.01105	0.001248	0.009303	Mesothelioma	FALSE					361194	165	361029	UK Biobank	https://docs.google.com/spreadsheets/d/1kvPoupSzsSFBNSztMzl04xMoSC3Kcx3CrjVf4yBmESU/edit?ts=5b5f17db#gid=227859291	PHESANT Transformation:NA-Notes:NA-Variable type:categorical-Phenotype ID:C3_MESOTHELIOMA
categorical.C3_OTHER_SKIN.txt	1710174270056F5forCTG.txt.gz	0.03071	0.03953	0.7768	0.4373	0.02783	0.00499	1.019	0.02202	-0.008228	0.008277	Other malignant neoplasms of skin	FALSE					361194	14402	346792	UK Biobank	https://docs.google.com/spreadsheets/d/1kvPoupSzsSFBNSztMzl04xMoSC3Kcx3CrjVf4yBmESU/edit?ts=5b5f17db#gid=227859291	PHESANT Transformation:NA-Notes:NA-Variable type:categorical-Phenotype ID:C3_OTHER_SKIN
categorical.C3_PROSTATE.txt	1710174270056F5forCTG.txt.gz	-0.008035	0.06123	-0.1312	0.8956	0.01022	0.0025	1.044	0.02186	0.0003051	0.007868	Malignant neoplasm of prostate	FALSE					361194	6321	354873	UK Biobank	https://docs.google.com/spreadsheets/d/1kvPoupSzsSFBNSztMzl04xMoSC3Kcx3CrjVf4yBmESU/edit?ts=5b5f17db#gid=227859291	PHESANT Transformation:NA-Notes:NA-Variable type:categorical-Phenotype ID:C3_PROSTATE
categorical.C3_RESPIRATORY_INTRATHORACIC.txt	1710174270056F5forCTG.txt.gz	0.06495	0.08501	0.764	0.4448	0.004508	0.001598	0.986	0.009571	0.01525	0.007867	Malignant neoplasm of respiratory system and intrathoracic organs	FALSE					361194	1944	359250	UK Biobank	https://docs.google.com/spreadsheets/d/1kvPoupSzsSFBNSztMzl04xMoSC3Kcx3CrjVf4yBmESU/edit?ts=5b5f17db#gid=227859291	PHESANT Transformation:NA-Notes:NA-Variable type:categorical-Phenotype ID:C3_RESPIRATORY_INTRATHORACIC
categorical.C3_SKIN.txt	1710174270056F5forCTG.txt.gz	0.02982	0.04002	0.7451	0.4562	0.02647	0.005203	1.023	0.02213	-0.01152	0.008226	Malignant neoplasm of skin	FALSE					361194	16531	344663	UK Biobank	https://docs.google.com/spreadsheets/d/1kvPoupSzsSFBNSztMzl04xMoSC3Kcx3CrjVf4yBmESU/edit?ts=5b5f17db#gid=227859291	PHESANT Transformation:NA-Notes:NA-Variable type:categorical-Phenotype ID:C3_SKIN
categorical.C34.txt	1710174270056F5forCTG.txt.gz	0.03883	0.119	0.3262	0.7443	0.002738	0.001554	0.999	0.009287	0.01247	0.007889	Diagnoses - main ICD10: C34 Malignant neoplasm of bronchus and lung	FALSE					361194	1427	359767	UK Biobank	https://docs.google.com/spreadsheets/d/1kvPoupSzsSFBNSztMzl04xMoSC3Kcx3CrjVf4yBmESU/edit?ts=5b5f17db#gid=227859291	PHESANT Transformation:NA-Notes:NA-Variable type:categorical-Phenotype ID:C34
categorical.C43.txt	1710174270056F5forCTG.txt.gz	0.1154	0.09664	1.194	0.2326	0.003733	0.001944	1.001	0.01112	-0.01962	0.007752	Diagnoses - main ICD10: C43 Malignant melanoma of skin	FALSE					361194	1672	359522	UK Biobank	https://docs.google.com/spreadsheets/d/1kvPoupSzsSFBNSztMzl04xMoSC3Kcx3CrjVf4yBmESU/edit?ts=5b5f17db#gid=227859291	PHESANT Transformation:NA-Notes:NA-Variable type:categorical-Phenotype ID:C43
categorical.C44.txt	1710174270056F5forCTG.txt.gz	0.05518	0.03829	1.441	0.1496	0.02145	0.003815	0.988	0.01653	-0.008735	0.007571	Diagnoses - main ICD10: C44 Other malignant neoplasms of skin	FALSE					361194	9086	352108	UK Biobank	https://docs.google.com/spreadsheets/d/1kvPoupSzsSFBNSztMzl04xMoSC3Kcx3CrjVf4yBmESU/edit?ts=5b5f17db#gid=227859291	PHESANT Transformation:NA-Notes:NA-Variable type:categorical-Phenotype ID:C44
categorical.C50.txt	1710174270056F5forCTG.txt.gz	0.07137	0.06249	1.142	0.2534	0.01049	0.002199	1.023	0.01354	-0.0135	0.007972	Diagnoses - main ICD10: C50 Malignant neoplasm of breast	FALSE					361194	8304	352890	UK Biobank	https://docs.google.com/spreadsheets/d/1kvPoupSzsSFBNSztMzl04xMoSC3Kcx3CrjVf4yBmESU/edit?ts=5b5f17db#gid=227859291	PHESANT Transformation:NA-Notes:NA-Variable type:categorical-Phenotype ID:C50
categorical.C61.txt	1710174270056F5forCTG.txt.gz	-0.02623	0.07008	-0.3743	0.7082	0.006891	0.002106	1.029	0.01551	0.004934	0.007843	Diagnoses - main ICD10: C61 Malignant neoplasm of prostate	FALSE					361194	4342	356852	UK Biobank	https://docs.google.com/spreadsheets/d/1kvPoupSzsSFBNSztMzl04xMoSC3Kcx3CrjVf4yBmESU/edit?ts=5b5f17db#gid=227859291	PHESANT Transformation:NA-Notes:NA-Variable type:categorical-Phenotype ID:C61
categorical.C67.txt	1710174270056F5forCTG.txt.gz	0.06369	0.1377	0.4627	0.6436	0.002026	0.001717	1.01	0.00928	0.003448	0.007715	Diagnoses - main ICD10: C67 Malignant neoplasm of bladder	FALSE					361194	1554	359640	UK Biobank	https://docs.google.com/spreadsheets/d/1kvPoupSzsSFBNSztMzl04xMoSC3Kcx3CrjVf4yBmESU/edit?ts=5b5f17db#gid=227859291	PHESANT Transformation:NA-Notes:NA-Variable type:categorical-Phenotype ID:C67
categorical.CARDIAC_ARRHYTM.txt	1710174270056F5forCTG.txt.gz	-0.01401	0.05597	-0.2503	0.8023	0.01432	0.002429	1.014	0.01554	0.01775	0.008415	Cardiac arrhytmias, COPD co-morbidities	FALSE					361194	8801	352393	UK Biobank	https://docs.google.com/spreadsheets/d/1kvPoupSzsSFBNSztMzl04xMoSC3Kcx3CrjVf4yBmESU/edit?ts=5b5f17db#gid=227859291	PHESANT Transformation:NA-Notes:NA-Variable type:categorical-Phenotype ID:CARDIAC_ARRHYTM
categorical.CHRONNAS.txt	1710174270056F5forCTG.txt.gz	-0.01918	0.1121	-0.1711	0.8641	0.002907	0.001466	1.01	0.009083	0.01503	0.008675	Chron's disease NAS	FALSE					361194	764	360430	UK Biobank	https://docs.google.com/spreadsheets/d/1kvPoupSzsSFBNSztMzl04xMoSC3Kcx3CrjVf4yBmESU/edit?ts=5b5f17db#gid=227859291	PHESANT Transformation:NA-Notes:NA-Variable type:categorical-Phenotype ID:CHRONNAS
categorical.COLITNONINFNAS.txt	1710174270056F5forCTG.txt.gz	0.4067	0.1134	3.587	0.000335	0.004091	0.001549	1.006	0.009408	0.0006773	0.007317	Noninfectious colitis NAS	FALSE					361194	8945	352249	UK Biobank	https://docs.google.com/spreadsheets/d/1kvPoupSzsSFBNSztMzl04xMoSC3Kcx3CrjVf4yBmESU/edit?ts=5b5f17db#gid=227859291	PHESANT Transformation:NA-Notes:NA-Variable type:categorical-Phenotype ID:COLITNONINFNAS
categorical.COPD_EARLYANDLATER.txt	1710174270056F5forCTG.txt.gz	0.2627	0.07875	3.336	0.000851	0.005986	0.001609	1.006	0.009122	0.01516	0.00722	COPD, early/later onset	FALSE					361194	1897	359297	UK Biobank	https://docs.google.com/spreadsheets/d/1kvPoupSzsSFBNSztMzl04xMoSC3Kcx3CrjVf4yBmESU/edit?ts=5b5f17db#gid=227859291	PHESANT Transformation:NA-Notes:NA-Variable type:categorical-Phenotype ID:COPD_EARLYANDLATER
categorical.COPD_EXCL.txt	1710174270056F5forCTG.txt.gz	0.402	0.0592	6.79	1.12E-11	0.01232	0.001745	1.008	0.01029	0.007253	0.008718	COPD differential diagnosis	FALSE	Pulmonary				361194	26710	334484	UK Biobank	https://docs.google.com/spreadsheets/d/1kvPoupSzsSFBNSztMzl04xMoSC3Kcx3CrjVf4yBmESU/edit?ts=5b5f17db#gid=227859291	PHESANT Transformation:NA-Notes:NA-Variable type:categorical-Phenotype ID:COPD_EXCL
categorical.COPD_OPPORTUNIST_INFECTIONS.txt	1710174270056F5forCTG.txt.gz	0.02764	0.09848	0.2807	0.779	0.003507	0.001695	0.987	0.009601	0.01082	0.008736	COPD related to chronic (opportunist) infections	FALSE					361194	477	360717	UK Biobank	https://docs.google.com/spreadsheets/d/1kvPoupSzsSFBNSztMzl04xMoSC3Kcx3CrjVf4yBmESU/edit?ts=5b5f17db#gid=227859291	PHESANT Transformation:NA-Notes:NA-Variable type:categorical-Phenotype ID:COPD_OPPORTUNIST_INFECTIONS
categorical.COX_ARTHROSIS.txt	1710174270056F5forCTG.txt.gz	0.1287	0.04964	2.592	0.009555	0.01839	0.002343	1.012	0.01251	-0.00792	0.008438	Coxarthrosis [arthrosis of hip](FG)	FALSE					361194	9410	351784	UK Biobank	https://docs.google.com/spreadsheets/d/1kvPoupSzsSFBNSztMzl04xMoSC3Kcx3CrjVf4yBmESU/edit?ts=5b5f17db#gid=227859291	PHESANT Transformation:NA-Notes:NA-Variable type:categorical-Phenotype ID:COX_ARTHROSIS
categorical.D12.txt	1710174270056F5forCTG.txt.gz	0.1637	0.06337	2.584	0.009779	0.01055	0.001994	1.025	0.01103	-0.000171	0.007995	Diagnoses - main ICD10: D12 Benign neoplasm of colon, rectum, anus and anal canal	FALSE					361194	8877	352317	UK Biobank	https://docs.google.com/spreadsheets/d/1kvPoupSzsSFBNSztMzl04xMoSC3Kcx3CrjVf4yBmESU/edit?ts=5b5f17db#gid=227859291	PHESANT Transformation:NA-Notes:NA-Variable type:categorical-Phenotype ID:D12
categorical.D17.txt	1710174270056F5forCTG.txt.gz	0.4922	0.1435	3.429	0.000605	0.003012	0.001436	1.006	0.008458	-0.01285	0.007814	Diagnoses - main ICD10: D17 Benign lipomatous neoplasm	FALSE					361194	4314	356880	UK Biobank	https://docs.google.com/spreadsheets/d/1kvPoupSzsSFBNSztMzl04xMoSC3Kcx3CrjVf4yBmESU/edit?ts=5b5f17db#gid=227859291	PHESANT Transformation:NA-Notes:NA-Variable type:categorical-Phenotype ID:D17
categorical.D23.txt	1710174270056F5forCTG.txt.gz	0.113	0.1022	1.106	0.2689	0.00257	0.001549	0.995	0.009147	0.004361	0.007525	Diagnoses - main ICD10: D23 Other benign neoplasms of skin	FALSE					361194	3085	358109	UK Biobank	https://docs.google.com/spreadsheets/d/1kvPoupSzsSFBNSztMzl04xMoSC3Kcx3CrjVf4yBmESU/edit?ts=5b5f17db#gid=227859291	PHESANT Transformation:NA-Notes:NA-Variable type:categorical-Phenotype ID:D23

categorical.D25.txt	1710174270056F5forCTG.txt.gz	0.1835	0.0987	1.859	0.06307	0.003903	0.001637	1.013	0.009636	0.0001029	0.007571	Diagnoses - main ICD10: D25 Leiomyoma of uterus	FALSE				361194	5507	355687	UK Biobank	https://docs.google.com/spreadsheets/d/1kvPoupSzsSFBNSztMzl04xMoSC3Kcx3CrjVf4yBmESU/edit?ts=5b5f17db#gid=227859291	PHESANT Transformation:NA-Notes:NA-Variable type:categorical-Phenotype ID:D25
categorical.D3_ANAEMIA_IRONDEF.txt	1710174270056F5forCTG.txt.gz	0.3015	0.08384	3.596	0.000323	0.004939	0.001608	0.992	0.009571	-0.006927	0.007169	Iron deficiency anaemia	FALSE				361194	3786	357408	UK Biobank	https://docs.google.com/spreadsheets/d/1kvPoupSzsSFBNSztMzl04xMoSC3Kcx3CrjVf4yBmESU/edit?ts=5b5f17db#gid=227859291	PHESANT Transformation:NA-Notes:NA-Variable type:categorical-Phenotype ID:D3_ANAEMIA_IRONDEF
categorical.D50.txt	1710174270056F5forCTG.txt.gz	0.3015	0.08384	3.596	0.000323	0.004939	0.001608	0.992	0.009571	-0.006927	0.007169	Diagnoses - main ICD10: D50 Iron deficiency anaemia	FALSE				361194	3222	357972	UK Biobank	https://docs.google.com/spreadsheets/d/1kvPoupSzsSFBNSztMzl04xMoSC3Kcx3CrjVf4yBmESU/edit?ts=5b5f17db#gid=227859291	PHESANT Transformation:NA-Notes:NA-Variable type:categorical-Phenotype ID:D50
categorical.D86.txt	1710174270056F5forCTG.txt.gz	0.007221	0.1176	0.06142	0.951	0.003279	0.001695	0.992	0.01028	-0.005389	0.009299	Diagnoses - main ICD10: D86 Sarcoidosis	FALSE				361194	170	361024	UK Biobank	https://docs.google.com/spreadsheets/d/1kvPoupSzsSFBNSztMzl04xMoSC3Kcx3CrjVf4yBmESU/edit?ts=5b5f17db#gid=227859291	PHESANT Transformation:NA-Notes:NA-Variable type:categorical-Phenotype ID:D86
categorical.E04.txt	1710174270056F5forCTG.txt.gz	-0.01209	0.06881	-0.1757	0.8605	0.006278	0.001701	0.998	0.009357	0.006052	0.007272	Diagnoses - main ICD10: E04 Other non-toxic goitre	FALSE				361194	1052	360142	UK Biobank	https://docs.google.com/spreadsheets/d/1kvPoupSzsSFBNSztMzl04xMoSC3Kcx3CrjVf4yBmESU/edit?ts=5b5f17db#gid=227859291	PHESANT Transformation:NA-Notes:NA-Variable type:categorical-Phenotype ID:E04
categorical.E11.txt	1710174270056F5forCTG.txt.gz	0.356	0.1132	3.145	0.001662	0.003475	0.001504	0.999	0.00909	-0.01187	0.008095	Diagnoses - main ICD10: E11 Non-insulin-dependent diabetes mellitus	FALSE				361194	705	360489	UK Biobank	https://docs.google.com/spreadsheets/d/1kvPoupSzsSFBNSztMzl04xMoSC3Kcx3CrjVf4yBmESU/edit?ts=5b5f17db#gid=227859291	PHESANT Transformation:NA-Notes:NA-Variable type:categorical-Phenotype ID:E11
categorical.E4_DM2.txt	1710174270056F5forCTG.txt.gz	0.3706	0.1197	3.097	0.001958	0.003285	0.00151	1	0.0091	-0.01211	0.008051	Type 2 diabetes	FALSE				361194	888	360306	UK Biobank	https://docs.google.com/spreadsheets/d/1kvPoupSzsSFBNSztMzl04xMoSC3Kcx3CrjVf4yBmESU/edit?ts=5b5f17db#gid=227859291	PHESANT Transformation:NA-Notes:NA-Variable type:categorical-Phenotype ID:E4_DM2
categorical.E4_OBESITY.txt	1710174270056F5forCTG.txt.gz	0.3763	0.1081	3.482	0.000498	0.004484	0.001643	0.995	0.009938	0.00359	0.008401	Obesity	FALSE				361194	448	360746	UK Biobank	https://docs.google.com/spreadsheets/d/1kvPoupSzsSFBNSztMzl04xMoSC3Kcx3CrjVf4yBmESU/edit?ts=5b5f17db#gid=227859291	PHESANT Transformation:NA-Notes:NA-Variable type:categorical-Phenotype ID:E4_OBESITY
categorical.E4_OBESITYNAS.txt	1710174270056F5forCTG.txt.gz	0.3404	0.09328	3.649	0.000263	0.0053	0.00171	0.991	0.01014	0.002273	0.008241	Obesity, other/unspecified	FALSE				361194	433	360761	UK Biobank	https://docs.google.com/spreadsheets/d/1kvPoupSzsSFBNSztMzl04xMoSC3Kcx3CrjVf4yBmESU/edit?ts=5b5f17db#gid=227859291	PHESANT Transformation:NA-Notes:NA-Variable type:categorical-Phenotype ID:E4_OBESITYNAS
categorical.E66.txt	1710174270056F5forCTG.txt.gz	0.3668	0.1052	3.485	0.000492	0.004575	0.001632	0.994	0.00976	0.00397	0.008229	Diagnoses - main ICD10: E66 Obesity	FALSE				361194	353	360841	UK Biobank	https://docs.google.com/spreadsheets/d/1kvPoupSzsSFBNSztMzl04xMoSC3Kcx3CrjVf4yBmESU/edit?ts=5b5f17db#gid=227859291	PHESANT Transformation:NA-Notes:NA-Variable type:categorical-Phenotype ID:E66
categorical.E87.txt	1710174270056F5forCTG.txt.gz	0.344	0.1314	2.618	0.008854	0.002572	0.001413	0.983	0.008684	-0.01069	0.007609	Diagnoses - main ICD10: E87 Other disorders of fluid, electrolyte and acid-base balance	FALSE				361194	564	360630	UK Biobank	https://docs.google.com/spreadsheets/d/1kvPoupSzsSFBNSztMzl04xMoSC3Kcx3CrjVf4yBmESU/edit?ts=5b5f17db#gid=227859291	PHESANT Transformation:NA-Notes:NA-Variable type:categorical-Phenotype ID:E87
categorical.ENDOMETRIOSIS_NOS.txt	1710174270056F5forCTG.txt.gz	-0.0188	0.1093	-0.1721	0.8634	0.002879	0.0015	0.998	0.009865	0.01607	0.008435	Unsepcified/other endometriosis	FALSE				361194	434	360760	UK Biobank	https://docs.google.com/spreadsheets/d/1kvPoupSzsSFBNSztMzl04xMoSC3Kcx3CrjVf4yBmESU/edit?ts=5b5f17db#gid=227859291	PHESANT Transformation:NA-Notes:NA-Variable type:categorical-Phenotype ID:ENDOMETRIOSIS_NOS
categorical.F10.txt	1710174270056F5forCTG.txt.gz	-0.01094	0.09732	-0.1124	0.9105	0.004256	0.001545	0.995	0.008963	0.01634	0.007804	Diagnoses - main ICD10: F10 Mental and behavioural disorders due to use of alcohol	FALSE				361194	786	360408	UK Biobank	https://docs.google.com/spreadsheets/d/1kvPoupSzsSFBNSztMzl04xMoSC3Kcx3CrjVf4yBmESU/edit?ts=5b5f17db#gid=227859291	PHESANT Transformation:NA-Notes:NA-Variable type:categorical-Phenotype ID:F10
categorical.F31.txt	1710174270056F5forCTG.txt.gz	-0.02747	0.07489	-0.3668	0.7138	0.006909	0.001924	0.988	0.01034	0.01168	0.00876	Diagnoses - main ICD10: F31 Bipolar affective disorder	FALSE				361194	371	360823	UK Biobank	https://docs.google.com/spreadsheets/d/1kvPoupSzsSFBNSztMzl04xMoSC3Kcx3CrjVf4yBmESU/edit?ts=5b5f17db#gid=227859291	PHESANT Transformation:NA-Notes:NA-Variable type:categorical-Phenotype ID:F31
categorical.F32.txt	1710174270056F5forCTG.txt.gz	0.07088	0.1019	0.6953	0.4869	0.003132	0.001454	1.003	0.009098	-0.00245	0.007379	Diagnoses - main ICD10: F32 Depressive episode	FALSE				361194	776	360418	UK Biobank	https://docs.google.com/spreadsheets/d/1kvPoupSzsSFBNSztMzl04xMoSC3Kcx3CrjVf4yBmESU/edit?ts=5b5f17db#gid=227859291	PHESANT Transformation:NA-Notes:NA-Variable type:categorical-Phenotype ID:F32
categorical.F5_DEPRESSIO.txt	1710174270056F5forCTG.txt.gz	0.03551	0.08011	0.4433	0.6575	0.005534	0.001512	0.987	0.009094	-0.001256	0.007892	Depression	FALSE	Psychiatric			361194	1145	360049	UK Biobank	https://docs.google.com/spreadsheets/d/1kvPoupSzsSFBNSztMzl04xMoSC3Kcx3CrjVf4yBmESU/edit?ts=5b5f17db#gid=227859291	PHESANT Transformation:NA-Notes:NA-Variable type:categorical-Phenotype ID:F5_DEPRESSIO
categorical.F5_MOOD.txt	1710174270056F5forCTG.txt.gz	0.004686	0.07879	0.05948	0.9526	0.006518	0.001662	0.99	0.009621	0.004721	0.008444	Mood [affective] disorders	FALSE				361194	1546	359648	UK Biobank	https://docs.google.com/spreadsheets/d/1kvPoupSzsSFBNSztMzl04xMoSC3Kcx3CrjVf4yBmESU/edit?ts=5b5f17db#gid=227859291	PHESANT Transformation:NA-Notes:NA-Variable type:categorical-Phenotype ID:F5_MOOD
categorical.FIBRO_COMORB.txt	1710174270056F5forCTG.txt.gz	0.1664	0.1144	1.455	0.1456	0.003148	0.001514	1.006	0.009696	-0.001932	0.008048	Fibromyalgia related co-morbidities	FALSE				361194	2305	358889	UK Biobank	https://docs.google.com/spreadsheets/d/1kvPoupSzsSFBNSztMzl04xMoSC3Kcx3CrjVf4yBmESU/edit?ts=5b5f17db#gid=227859291	PHESANT Transformation:NA-Notes:NA-Variable type:categorical-Phenotype ID:FIBRO_COMORB
categorical.G37.txt	1710174270056F5forCTG.txt.gz	-0.2547	0.192	-1.327	0.1846	0.001654	0.001489	0.989	0.009952	0.01656	0.00879	Diagnoses - main ICD10: G37 Other demyelinating diseases of central nervous system	FALSE				361194	200	360994	UK Biobank	https://docs.google.com/spreadsheets/d/1kvPoupSzsSFBNSztMzl04xMoSC3Kcx3CrjVf4yBmESU/edit?ts=5b5f17db#gid=227859291	PHESANT Transformation:NA-Notes:NA-Variable type:categorical-Phenotype ID:G37
categorical.G47.txt	1710174270056F5forCTG.txt.gz	0.2912	0.07516	3.875	0.000107	0.006808	0.001582	0.997	0.009148	0.0004504	0.007938	Diagnoses - main ICD10: G47 Sleep disorders	FALSE				361194	2723	358471	UK Biobank	https://docs.google.com/spreadsheets/d/1kvPoupSzsSFBNSztMzl04xMoSC3Kcx3CrjVf4yBmESU/edit?ts=5b5f17db#gid=227859291	PHESANT Transformation:NA-Notes:NA-Variable type:categorical-Phenotype ID:G47
categorical.G56.txt	1710174270056F5forCTG.txt.gz	0.3346	0.05057	6.616	3.69E-11	0.0172	0.002024	1.021	0.01116	-0.006247	0.008537	Diagnoses - main ICD10: G56 Mononeuropathies of upper limb	FALSE	Pain			361194	8130	353064	UK Biobank	https://docs.google.com/spreadsheets/d/1kvPoupSzsSFBNSztMzl04xMoSC3Kcx3CrjVf4yBmESU/edit?ts=5b5f17db#gid=227859291	PHESANT Transformation:NA-Notes:NA-Variable type:categorical-Phenotype ID:G56
categorical.G6_CARPTU.txt	1710174270056F5forCTG.txt.gz	0.3079	0.05086	6.053	1.42E-09	0.01691	0.002007	1.024	0.01138	-0.003273	0.00869	Carpal tunnel syndrome	FALSE	Other (physical health)			361194	7973	353221	UK Biobank	https://docs.google.com/spreadsheets/d/1kvPoupSzsSFBNSztMzl04xMoSC3Kcx3CrjVf4yBmESU/edit?ts=5b5f17db#gid=227859291	PHESANT Transformation:NA-Notes:NA-Variable type:categorical-Phenotype ID:G6_CARPTU
categorical.G6_DISBROTHUNS.txt	1710174270056F5forCTG.txt.gz	0.3098	0.1203	2.576	0.01	0.003769	0.001634	0.983	0.01061	-0.03212	0.009625	Disorders of brain, other and unspecified	FALSE				361194	127	361067	UK Biobank	https://docs.google.com/spreadsheets/d/1kvPoupSzsSFBNSztMzl04xMoSC3Kcx3CrjVf4yBmESU/edit?ts=5b5f17db#gid=227859291	PHESANT Transformation:NA-Notes:NA-Variable type:categorical-Phenotype ID:G6_DISBROTHUNS
categorical.G6_EPIPAROX.txt	1710174270056F5forCTG.txt.gz	0.3557	0.08193	4.341	1.42E-05	0.006206	0.00161	1	0.01031	-0.001497	0.008126	Episodal and paroxysmal disorders	FALSE	Other (physical health)			361194	5418	355776	UK Biobank	https://docs.google.com/spreadsheets/d/1kvPoupSzsSFBNSztMzl04xMoSC3Kcx3CrjVf4yBmESU/edit?ts=5b5f17db#gid=227859291	PHESANT Transformation:NA-Notes:NA-Variable type:categorical-Phenotype ID:G6_EPIPAROX
categorical.G6_NERPLEX.txt	1710174270056F5forCTG.txt.gz	0.3615	0.05078	7.118	1.09E-12	0.01485	0.001871	1.029	0.01043	-0.002616	0.008457	Nerve, nerve root and plexus disorders	FALSE	Pain			361194	10898	350296	UK Biobank	https://docs.google.com/spreadsheets/d/1kvPoupSzsSFBNSztMzl04xMoSC3Kcx3CrjVf4yBmESU/edit?ts=5b5f17db#gid=227859291	PHESANT Transformation:NA-Notes:NA-Variable type:categorical-Phenotype ID:G6_NERPLEX
categorical.G6_POLYNEU.txt	1710174270056F5forCTG.txt.gz	-0.06769	0.1187	-0.5702	0.5685	0.002437	0.001416	0.998	0.009708	0.01438	0.007574	Polyneuropathies and other disorders of the peripheral nervous system	FALSE				361194	560	360634	UK Biobank	https://docs.google.com/spreadsheets/d/1kvPoupSzsSFBNSztMzl04xMoSC3Kcx3CrjVf4yBmESU/edit?ts=5b5f17db#gid=227859291	PHESANT Transformation:NA-Notes:NA-Variable type:categorical-Phenotype ID:G6_POLYNEU
categorical.G6_POLYOTHUNS.txt	1710174270056F5forCTG.txt.gz	-0.03019	0.09786	-0.3085	0.7577	0.003747	0.00146	0.985	0.009697	0.01263	0.007761	Other and unspecified polyneuropathies, also in other diseases	FALSE				361194	379	360815	UK Biobank	https://docs.google.com/spreadsheets/d/1kvPoupSzsSFBNSztMzl04xMoSC3Kcx3CrjVf4yBmESU/edit?ts=5b5f17db#gid=227859291	PHESANT Transformation:NA-Notes:NA-Variable type:categorical-Phenotype ID:G6_POLYOTHUNS
categorical.G6_SLEEPAPNO.txt	1710174270056F5forCTG.txt.gz	0.3003	0.08535	3.518	0.000435	0.005665	0.001655	0.998	0.009554	0.00132	0.007983	Sleep apnoea	FALSE				361194	2249	358945	UK Biobank	https://docs.google.com/spreadsheets/d/1kvPoupSzsSFBNSztMzl04xMoSC3Kcx3CrjVf4yBmESU/edit?ts=5b5f17db#gid=227859291	PHESANT Transformation:NA-Notes:NA-Variable type:categorical-Phenotype ID:G6_SLEEPAPNO

categorical.GLOMER_NEPHRITIS.txt	1710174270056F5forCTG.txt.gz	0.3224	0.1858	1.735	0.0827	0.001836	0.001397	0.998	0.008404	-0.009325	0.008076	Glomerulonephritis	FALSE					361194	629	360565	UK Biobank	https://docs.google.com/spreadsheets/d/1kvPoupSzsSFBNSztMzl04xMoSC3Kcx3CrjVf4yBmESU/edit?ts=5b5f17db#gid=227859291	PHESANT Transformation:NA-Notes:NA-Variable type:categorical-Phenotype ID:GLOMER_NEPHRITIS
categorical.H25.txt	1710174270056F5forCTG.txt.gz	0.2242	0.07034	3.187	0.001438	0.007118	0.001843	1.015	0.01072	-0.01632	0.008098	Diagnoses - main ICD10: H25 Senile cataract	FALSE					361194	6332	354862	UK Biobank	https://docs.google.com/spreadsheets/d/1kvPoupSzsSFBNSztMzl04xMoSC3Kcx3CrjVf4yBmESU/edit?ts=5b5f17db#gid=227859291	PHESANT Transformation:NA-Notes:NA-Variable type:categorical-Phenotype ID:H25
categorical.H26.txt	1710174270056F5forCTG.txt.gz	0.1229	0.06407	1.918	0.05506	0.01108	0.001928	1.004	0.01081	-0.006467	0.008738	Diagnoses - main ICD10: H26 Other cataract	FALSE					361194	11306	349888	UK Biobank	https://docs.google.com/spreadsheets/d/1kvPoupSzsSFBNSztMzl04xMoSC3Kcx3CrjVf4yBmESU/edit?ts=5b5f17db#gid=227859291	PHESANT Transformation:NA-Notes:NA-Variable type:categorical-Phenotype ID:H26
categorical.H33.txt	1710174270056F5forCTG.txt.gz	-0.07378	0.06424	-1.149	0.2507	0.008396	0.001824	1.003	0.009677	-0.008333	0.008067	Diagnoses - main ICD10: H33 Retinal detachments and breaks	FALSE					361194	2671	358523	UK Biobank	https://docs.google.com/spreadsheets/d/1kvPoupSzsSFBNSztMzl04xMoSC3Kcx3CrjVf4yBmESU/edit?ts=5b5f17db#gid=227859291	PHESANT Transformation:NA-Notes:NA-Variable type:categorical-Phenotype ID:H33
categorical.H40.txt	1710174270056F5forCTG.txt.gz	-0.09088	0.107	-0.849	0.3959	0.003028	0.001446	1.003	0.009479	0.002126	0.007842	Diagnoses - main ICD10: H40 Glaucoma	FALSE					361194	1715	359479	UK Biobank	https://docs.google.com/spreadsheets/d/1kvPoupSzsSFBNSztMzl04xMoSC3Kcx3CrjVf4yBmESU/edit?ts=5b5f17db#gid=227859291	PHESANT Transformation:NA-Notes:NA-Variable type:categorical-Phenotype ID:H40
categorical.H53.txt	1710174270056F5forCTG.txt.gz	0.05367	0.107	0.5015	0.616	0.002864	0.00144	0.987	0.008993	0.005364	0.007689	Diagnoses - main ICD10: H53 Visual disturbances	FALSE					361194	560	360634	UK Biobank	https://docs.google.com/spreadsheets/d/1kvPoupSzsSFBNSztMzl04xMoSC3Kcx3CrjVf4yBmESU/edit?ts=5b5f17db#gid=227859291	PHESANT Transformation:NA-Notes:NA-Variable type:categorical-Phenotype ID:H53
categorical.H65.txt	1710174270056F5forCTG.txt.gz	0.009755	0.1052	0.09269	0.9262	0.002765	0.001543	0.999	0.009276	0.01241	0.007337	Diagnoses - main ICD10: H65 Nonsuppurative otitis media	FALSE					361194	781	360413	UK Biobank	https://docs.google.com/spreadsheets/d/1kvPoupSzsSFBNSztMzl04xMoSC3Kcx3CrjVf4yBmESU/edit?ts=5b5f17db#gid=227859291	PHESANT Transformation:NA-Notes:NA-Variable type:categorical-Phenotype ID:H65
categorical.H7_ENTROPION.txt	1710174270056F5forCTG.txt.gz	0.1063	0.1257	0.8459	0.3976	0.002499	0.001511	1.005	0.009173	-0.005114	0.007664	Entropion and trichiasis of eyelid	FALSE					361194	552	360642	UK Biobank	https://docs.google.com/spreadsheets/d/1kvPoupSzsSFBNSztMzl04xMoSC3Kcx3CrjVf4yBmESU/edit?ts=5b5f17db#gid=227859291	PHESANT Transformation:NA-Notes:NA-Variable type:categorical-Phenotype ID:H7_ENTROPION
categorical.H7_EYELIDFUNC.txt	1710174270056F5forCTG.txt.gz	0.008895	0.1251	0.07109	0.9433	0.002549	0.001616	0.984	0.01182	0.01142	0.009706	Other disorders affecting eyelid function	FALSE					361194	134	361060	UK Biobank	https://docs.google.com/spreadsheets/d/1kvPoupSzsSFBNSztMzl04xMoSC3Kcx3CrjVf4yBmESU/edit?ts=5b5f17db#gid=227859291	PHESANT Transformation:NA-Notes:NA-Variable type:categorical-Phenotype ID:H7_EYELIDFUNC
categorical.H7_GLAUCNAS.txt	1710174270056F5forCTG.txt.gz	-0.1721	0.1562	-1.102	0.2706	0.001934	0.001573	1.004	0.009734	0.0126	0.008672	Other and unspecified glaucoma	FALSE					361194	573	360621	UK Biobank	https://docs.google.com/spreadsheets/d/1kvPoupSzsSFBNSztMzl04xMoSC3Kcx3CrjVf4yBmESU/edit?ts=5b5f17db#gid=227859291	PHESANT Transformation:NA-Notes:NA-Variable type:categorical-Phenotype ID:H7_GLAUCNAS
categorical.H7_LENS.txt	1710174270056F5forCTG.txt.gz	0.1406	0.05623	2.501	0.01239	0.01512	0.002099	1.015	0.01152	-0.01167	0.008979	Disorders of lens	FALSE					361194	17076	344118	UK Biobank	https://docs.google.com/spreadsheets/d/1kvPoupSzsSFBNSztMzl04xMoSC3Kcx3CrjVf4yBmESU/edit?ts=5b5f17db#gid=227859291	PHESANT Transformation:NA-Notes:NA-Variable type:categorical-Phenotype ID:H7_LENS
categorical.H7_RETINALDETACH.txt	1710174270056F5forCTG.txt.gz	-0.07814	0.06718	-1.163	0.2448	0.008276	0.001883	1.002	0.009887	-0.003095	0.007913	Retinal detachments and breaks	FALSE					361194	3043	358151	UK Biobank	https://docs.google.com/spreadsheets/d/1kvPoupSzsSFBNSztMzl04xMoSC3Kcx3CrjVf4yBmESU/edit?ts=5b5f17db#gid=227859291	PHESANT Transformation:NA-Notes:NA-Variable type:categorical-Phenotype ID:H7_RETINALDETACH
categorical.H7_RETINALDETACHBREAK.txt	1710174270056F5forCTG.txt.gz	0.02919	0.1046	0.2789	0.7803	0.003216	0.001386	1.005	0.008982	-0.01329	0.008152	Retinal detachment with retinal break	FALSE					361194	1198	359996	UK Biobank	https://docs.google.com/spreadsheets/d/1kvPoupSzsSFBNSztMzl04xMoSC3Kcx3CrjVf4yBmESU/edit?ts=5b5f17db#gid=227859291	PHESANT Transformation:NA-Notes:NA-Variable type:categorical-Phenotype ID:H7_RETINALDETACHBREAK
categorical.H7_RETINALDETACHOTH.txt	1710174270056F5forCTG.txt.gz	-0.1646	0.08702	-1.892	0.05856	0.003826	0.001516	1.002	0.009572	0.006367	0.007593	Other, unspecified and serous retinal detachments	FALSE					361194	923	360271	UK Biobank	https://docs.google.com/spreadsheets/d/1kvPoupSzsSFBNSztMzl04xMoSC3Kcx3CrjVf4yBmESU/edit?ts=5b5f17db#gid=227859291	PHESANT Transformation:NA-Notes:NA-Variable type:categorical-Phenotype ID:H7_RETINALDETACHOTH
categorical.H7_VISUDISTURB.txt	1710174270056F5forCTG.txt.gz	0.05367	0.107	0.5015	0.616	0.002864	0.00144	0.987	0.008993	0.005364	0.007689	Visual disturbances	FALSE					361194	611	360583	UK Biobank	https://docs.google.com/spreadsheets/d/1kvPoupSzsSFBNSztMzl04xMoSC3Kcx3CrjVf4yBmESU/edit?ts=5b5f17db#gid=227859291	PHESANT Transformation:NA-Notes:NA-Variable type:categorical-Phenotype ID:H7_VISUDISTURB
categorical.H80.txt	1710174270056F5forCTG.txt.gz	0.09107	0.1046	0.871	0.3838	0.003813	0.001679	0.996	0.00995	-0.007732	0.009113	Diagnoses - main ICD10: H80 Otosclerosis	FALSE					361194	221	360973	UK Biobank	https://docs.google.com/spreadsheets/d/1kvPoupSzsSFBNSztMzl04xMoSC3Kcx3CrjVf4yBmESU/edit?ts=5b5f17db#gid=227859291	PHESANT Transformation:NA-Notes:NA-Variable type:categorical-Phenotype ID:H80
categorical.HEARTFAIL.txt	1710174270056F5forCTG.txt.gz	0.05192	0.07438	0.6981	0.4851	0.007754	0.0015	0.974	0.009301	-0.001858	0.008236	Heart failure	FALSE					361194	1405	359789	UK Biobank	https://docs.google.com/spreadsheets/d/1kvPoupSzsSFBNSztMzl04xMoSC3Kcx3CrjVf4yBmESU/edit?ts=5b5f17db#gid=227859291	PHESANT Transformation:NA-Notes:NA-Variable type:categorical-Phenotype ID:HEARTFAIL
categorical.I_INFECT_PARASIT.txt	1710174270056F5forCTG.txt.gz	0.585	0.1745	3.352	0.000802	0.003191	0.001506	0.997	0.009118	-0.001002	0.008318	Certain infectious and parasitic diseases	FALSE					361194	9297	351897	UK Biobank	https://docs.google.com/spreadsheets/d/1kvPoupSzsSFBNSztMzl04xMoSC3Kcx3CrjVf4yBmESU/edit?ts=5b5f17db#gid=227859291	PHESANT Transformation:NA-Notes:NA-Variable type:categorical-Phenotype ID:I_INFECT_PARASIT
categorical.I10.txt	1710174270056F5forCTG.txt.gz	0.3681	0.1833	2.008	0.04462	0.002156	0.00168	1.005	0.01014	-0.01533	0.007663	Diagnoses - main ICD10: I10 Essential (primary) hypertension	FALSE					361194	787	360407	UK Biobank	https://docs.google.com/spreadsheets/d/1kvPoupSzsSFBNSztMzl04xMoSC3Kcx3CrjVf4yBmESU/edit?ts=5b5f17db#gid=227859291	PHESANT Transformation:NA-Notes:NA-Variable type:categorical-Phenotype ID:I10
categorical.I12.txt	1710174270056F5forCTG.txt.gz	0.2626	0.1242	2.115	0.03441	0.002915	0.001572	0.981	0.0102	-0.01378	0.008338	Diagnoses - main ICD10: I12 Hypertensive renal disease	FALSE					361194	176	361018	UK Biobank	https://docs.google.com/spreadsheets/d/1kvPoupSzsSFBNSztMzl04xMoSC3Kcx3CrjVf4yBmESU/edit?ts=5b5f17db#gid=227859291	PHESANT Transformation:NA-Notes:NA-Variable type:categorical-Phenotype ID:I12
categorical.I20.txt	1710174270056F5forCTG.txt.gz	0.2522	0.05764	4.376	1.21E-05	0.01426	0.002082	0.992	0.01113	0.007391	0.008992	Diagnoses - main ICD10: I20 Angina pectoris	FALSE	Cardiac				361194	6246	354948	UK Biobank	https://docs.google.com/spreadsheets/d/1kvPoupSzsSFBNSztMzl04xMoSC3Kcx3CrjVf4yBmESU/edit?ts=5b5f17db#gid=227859291	PHESANT Transformation:NA-Notes:NA-Variable type:categorical-Phenotype ID:I20
categorical.I21.txt	1710174270056F5forCTG.txt.gz	0.1723	0.05635	3.057	0.002234	0.01411	0.002034	0.997	0.01142	0.002789	0.008253	Diagnoses - main ICD10: I21 Acute myocardial infarction	FALSE					361194	5948	355246	UK Biobank	https://docs.google.com/spreadsheets/d/1kvPoupSzsSFBNSztMzl04xMoSC3Kcx3CrjVf4yBmESU/edit?ts=5b5f17db#gid=227859291	PHESANT Transformation:NA-Notes:NA-Variable type:categorical-Phenotype ID:I21
categorical.I25.txt	1710174270056F5forCTG.txt.gz	0.1683	0.04069	4.136	3.54E-05	0.02654	0.003365	1.028	0.01321	0.008467	0.007836	Diagnoses - main ICD10: I25 Chronic ischaemic heart disease	FALSE					361194	12769	348425	UK Biobank	https://docs.google.com/spreadsheets/d/1kvPoupSzsSFBNSztMzl04xMoSC3Kcx3CrjVf4yBmESU/edit?ts=5b5f17db#gid=227859291	PHESANT Transformation:NA-Notes:NA-Variable type:categorical-Phenotype ID:I25
categorical.I26.txt	1710174270056F5forCTG.txt.gz	0.1492	0.1112	1.342	0.1797	0.002827	0.001855	1.022	0.01202	0.0009594	0.008127	Diagnoses - main ICD10: I26 Pulmonary embolism	FALSE					361194	2118	359076	UK Biobank	https://docs.google.com/spreadsheets/d/1kvPoupSzsSFBNSztMzl04xMoSC3Kcx3CrjVf4yBmESU/edit?ts=5b5f17db#gid=227859291	PHESANT Transformation:NA-Notes:NA-Variable type:categorical-Phenotype ID:I26
categorical.I35.txt	1710174270056F5forCTG.txt.gz	0.03645	0.1065	0.3423	0.7321	0.002937	0.001556	1.005	0.00969	0.005517	0.007999	Diagnoses - main ICD10: I35 Nonrheumatic aortic valve disorders	FALSE					361194	901	360293	UK Biobank	https://docs.google.com/spreadsheets/d/1kvPoupSzsSFBNSztMzl04xMoSC3Kcx3CrjVf4yBmESU/edit?ts=5b5f17db#gid=227859291	PHESANT Transformation:NA-Notes:NA-Variable type:categorical-Phenotype ID:I35
categorical.I42.txt	1710174270056F5forCTG.txt.gz	-0.007615	0.1284	-0.0593	0.9527	0.002172	0.001543	0.99	0.009219	0.004275	0.008011	Diagnoses - main ICD10: I42 Cardiomyopathy	FALSE					361194	479	360715	UK Biobank	https://docs.google.com/spreadsheets/d/1kvPoupSzsSFBNSztMzl04xMoSC3Kcx3CrjVf4yBmESU/edit?ts=5b5f17db#gid=227859291	PHESANT Transformation:NA-Notes:NA-Variable type:categorical-Phenotype ID:I42
categorical.I48.txt	1710174270056F5forCTG.txt.gz	-0.05515	0.05665	-0.9734	0.3304	0.01536	0.002519	1.025	0.01682	0.01956	0.008403	Diagnoses - main ICD10: I48 Atrial fibrillation and flutter	FALSE					361194	6356	354838	UK Biobank	https://docs.google.com/spreadsheets/d/1kvPoupSzsSFBNSztMzl04xMoSC3Kcx3CrjVf4yBmESU/edit?ts=5b5f17db#gid=227859291	PHESANT Transformation:NA-Notes:NA-Variable type:categorical-Phenotype ID:I48
categorical.I50.txt	1710174270056F5forCTG.txt.gz	0.0389	0.07588	0.5126	0.6082	0.007279	0.001485	0.975	0.009383	0.0003876	0.008243	Diagnoses - main ICD10: I50 Heart failure	FALSE					361194	1088	360106	UK Biobank	https://docs.google.com/spreadsheets/d/1kvPoupSzsSFBNSztMzl04xMoSC3Kcx3CrjVf4yBmESU/edit?ts=5b5f17db#gid=227859291	PHESANT Transformation:NA-Notes:NA-Variable type:categorical-Phenotype ID:I50

categorical.I71.txt	1710174270056F5 forCTG.txt.gz	0.02151	0.1122	0.1918	0.8479	0.003126	0.001748	1.004	0.01028	-0.003927	0.008437	Diagnoses - main ICD10: I71 Aortic aneurysm and dissection	FALSE				361194	564	360630	UK Biobank	https://docs.google.com/spreadsheets/d/1kvPoupSzsSFBNSztMzl04xMoSC3Kcx3CrjVf4yBmESU/edit?ts=5b5f17db#gid=227859291	PHESANT Transformation:NA-Notes:NA-Variable type:categorical-Phenotype ID:I71
categorical.I80.txt	1710174270056F5 forCTG.txt.gz	0.1128	0.06864	1.644	0.1003	0.008039	0.002248	1.006	0.01403	-0.00416	0.008093	Diagnoses - main ICD10: I80 Phlebitis and thrombophlebitis	FALSE				361194	2289	358905	UK Biobank	https://docs.google.com/spreadsheets/d/1kvPoupSzsSFBNSztMzl04xMoSC3Kcx3CrjVf4yBmESU/edit?ts=5b5f17db#gid=227859291	PHESANT Transformation:NA-Notes:NA-Variable type:categorical-Phenotype ID:I80
categorical.I83.txt	1710174270056F5 forCTG.txt.gz	0.09403	0.05019	1.874	0.06099	0.01968	0.002366	1.042	0.0132	0.002591	0.008917	Diagnoses - main ICD10: I83 Varicose veins of lower extremities	FALSE				361194	8763	352431	UK Biobank	https://docs.google.com/spreadsheets/d/1kvPoupSzsSFBNSztMzl04xMoSC3Kcx3CrjVf4yBmESU/edit?ts=5b5f17db#gid=227859291	PHESANT Transformation:NA-Notes:NA-Variable type:categorical-Phenotype ID:I83
categorical.I84.txt	1710174270056F5 forCTG.txt.gz	0.172	0.06039	2.848	0.004396	0.01009	0.001959	1.015	0.01027	0.01362	0.008234	Diagnoses - main ICD10: I84 Haemorrhoids	FALSE				361194	12102	349092	UK Biobank	https://docs.google.com/spreadsheets/d/1kvPoupSzsSFBNSztMzl04xMoSC3Kcx3CrjVf4yBmESU/edit?ts=5b5f17db#gid=227859291	PHESANT Transformation:NA-Notes:NA-Variable type:categorical-Phenotype ID:I84
categorical.I9_AORTDIS.txt	1710174270056F5 forCTG.txt.gz	-0.0195	0.0998	-0.1954	0.8451	0.003538	0.001813	0.982	0.01125	-0.00432	0.008741	Dissection of aorta	FALSE				361194	129	361065	UK Biobank	https://docs.google.com/spreadsheets/d/1kvPoupSzsSFBNSztMzl04xMoSC3Kcx3CrjVf4yBmESU/edit?ts=5b5f17db#gid=227859291	PHESANT Transformation:NA-Notes:NA-Variable type:categorical-Phenotype ID:I9_AORTDIS
categorical.I9_CARDMPRI.txt	1710174270056F5 forCTG.txt.gz	0.05364	0.1282	0.4184	0.6757	0.002459	0.001576	0.991	0.009211	0.003107	0.008274	Cardiomyopathies, Primary/intrinsic	FALSE				361194	360	360834	UK Biobank	https://docs.google.com/spreadsheets/d/1kvPoupSzsSFBNSztMzl04xMoSC3Kcx3CrjVf4yBmESU/edit?ts=5b5f17db#gid=227859291	PHESANT Transformation:NA-Notes:NA-Variable type:categorical-Phenotype ID:I9_CARDMPRI
categorical.I9_CHD_NOREV.txt	1710174270056F5 forCTG.txt.gz	0.1923	0.05117	3.758	0.000171	0.01806	0.002505	1.009	0.01192	0.007127	0.008759	Major coronary heart disease event excluding revascularizations	FALSE				361194	10157	351037	UK Biobank	https://docs.google.com/spreadsheets/d/1kvPoupSzsSFBNSztMzl04xMoSC3Kcx3CrjVf4yBmESU/edit?ts=5b5f17db#gid=227859291	PHESANT Transformation:NA-Notes:NA-Variable type:categorical-Phenotype ID:I9_CHD_NOREV
categorical.I9_CHD.txt	1710174270056F5 forCTG.txt.gz	0.1923	0.05117	3.758	0.000171	0.01806	0.002505	1.009	0.01192	0.007127	0.008759	Major coronary heart disease event	FALSE				361194	10157	351037	UK Biobank	https://docs.google.com/spreadsheets/d/1kvPoupSzsSFBNSztMzl04xMoSC3Kcx3CrjVf4yBmESU/edit?ts=5b5f17db#gid=227859291	PHESANT Transformation:NA-Notes:NA-Variable type:categorical-Phenotype ID:I9_CHD
categorical.I9_CORATHER.txt	1710174270056F5 forCTG.txt.gz	0.1676	0.04028	4.161	3.17E-05	0.0274	0.003569	1.03	0.0137	0.007988	0.007968	Coronary atherosclerosis	TRUE	Cardiac	Physical health	Coronary atherosclerosis	361194	14334	346860	UK Biobank	https://docs.google.com/spreadsheets/d/1kvPoupSzsSFBNSztMzl04xMoSC3Kcx3CrjVf4yBmESU/edit?ts=5b5f17db#gid=227859291	PHESANT Transformation:NA-Notes:NA-Variable type:categorical-Phenotype ID:I9_CORATHER
categorical.I9_DISVEINLYMPH.txt	1710174270056F5 forCTG.txt.gz	0.1314	0.0532	2.471	0.01348	0.01935	0.002343	1.043	0.0129	-0.002519	0.009609	Diseases of veins, lymphatic vessels and lymph nodes, not elsewhere classified	FALSE				361194	11867	349327	UK Biobank	https://docs.google.com/spreadsheets/d/1kvPoupSzsSFBNSztMzl04xMoSC3Kcx3CrjVf4yBmESU/edit?ts=5b5f17db#gid=227859291	PHESANT Transformation:NA-Notes:NA-Variable type:categorical-Phenotype ID:I9_DISVEINLYMPH
categorical.I9_DVTANDPULM.txt	1710174270056F5 forCTG.txt.gz	0.141	0.0604	2.334	0.0196	0.01035	0.002411	1.009	0.01537	-0.005405	0.008254	DVT of lower extremities and pulmonary embolism	FALSE				361194	4319	356875	UK Biobank	https://docs.google.com/spreadsheets/d/1kvPoupSzsSFBNSztMzl04xMoSC3Kcx3CrjVf4yBmESU/edit?ts=5b5f17db#gid=227859291	PHESANT Transformation:NA-Notes:NA-Variable type:categorical-Phenotype ID:I9_DVTANDPULM
categorical.I9_HEARTFAIL_NS.txt	1710174270056F5 forCTG.txt.gz	0.05192	0.07438	0.6981	0.4851	0.007754	0.0015	0.974	0.009301	-0.001858	0.008236	Heart failure, not strict	FALSE				361194	1405	359789	UK Biobank	https://docs.google.com/spreadsheets/d/1kvPoupSzsSFBNSztMzl04xMoSC3Kcx3CrjVf4yBmESU/edit?ts=5b5f17db#gid=227859291	PHESANT Transformation:NA-Notes:NA-Variable type:categorical-Phenotype ID:I9_HEARTFAIL_NS
categorical.I9_HEARTFAIL.txt	1710174270056F5 forCTG.txt.gz	0.05192	0.07438	0.6981	0.4851	0.007754	0.0015	0.974	0.009301	-0.001858	0.008236	Heart failure,strict	FALSE				361194	1405	359789	UK Biobank	https://docs.google.com/spreadsheets/d/1kvPoupSzsSFBNSztMzl04xMoSC3Kcx3CrjVf4yBmESU/edit?ts=5b5f17db#gid=227859291	PHESANT Transformation:NA-Notes:NA-Variable type:categorical-Phenotype ID:I9_HEARTFAIL
categorical.I9_HYPERTENSION.txt	1710174270056F5 forCTG.txt.gz	0.3592	0.1235	2.909	0.003627	0.003604	0.001637	0.992	0.009551	-0.01654	0.008112	Hypertensive diseases	FALSE				361194	1313	359881	UK Biobank	https://docs.google.com/spreadsheets/d/1kvPoupSzsSFBNSztMzl04xMoSC3Kcx3CrjVf4yBmESU/edit?ts=5b5f17db#gid=227859291	PHESANT Transformation:NA-Notes:NA-Variable type:categorical-Phenotype ID:I9_HYPERTENSION
categorical.I9_HYPTENS.txt	1710174270056F5 forCTG.txt.gz	0.3614	0.1283	2.817	0.004851	0.003356	0.001631	0.994	0.009618	-0.01682	0.008173	Hypertension	FALSE				361194	1237	359957	UK Biobank	https://docs.google.com/spreadsheets/d/1kvPoupSzsSFBNSztMzl04xMoSC3Kcx3CrjVf4yBmESU/edit?ts=5b5f17db#gid=227859291	PHESANT Transformation:NA-Notes:NA-Variable type:categorical-Phenotype ID:I9_HYPTENS
categorical.I9_HYPTENSHR.txt	1710174270056F5 forCTG.txt.gz	0.2787	0.1093	2.551	0.01074	0.002901	0.001403	0.981	0.008588	-0.01192	0.007915	Hypertensive heart and/or renal disease	FALSE				361194	376	360818	UK Biobank	https://docs.google.com/spreadsheets/d/1kvPoupSzsSFBNSztMzl04xMoSC3Kcx3CrjVf4yBmESU/edit?ts=5b5f17db#gid=227859291	PHESANT Transformation:NA-Notes:NA-Variable type:categorical-Phenotype ID:I9_HYPTENSHR
categorical.I9_IHD.txt	1710174270056F5 forCTG.txt.gz	0.1997	0.04202	4.752	2.01E-06	0.03139	0.003698	1.037	0.01485	0.01018	0.009018	Ischaemic heart disease, wide definition	FALSE	Cardiac			361194	20857	340337	UK Biobank	https://docs.google.com/spreadsheets/d/1kvPoupSzsSFBNSztMzl04xMoSC3Kcx3CrjVf4yBmESU/edit?ts=5b5f17db#gid=227859291	PHESANT Transformation:NA-Notes:NA-Variable type:categorical-Phenotype ID:I9_IHD
categorical.I9_K_CARDIAC.txt	1710174270056F5 forCTG.txt.gz	0.1381	0.102	1.353	0.1759	0.003851	0.001637	0.99	0.009738	-0.005511	0.007727	Death due to cardiac causes	FALSE				361194	1597	359597	UK Biobank	https://docs.google.com/spreadsheets/d/1kvPoupSzsSFBNSztMzl04xMoSC3Kcx3CrjVf4yBmESU/edit?ts=5b5f17db#gid=227859291	PHESANT Transformation:NA-Notes:NA-Variable type:categorical-Phenotype ID:I9_K_CARDIAC
categorical.I9_MI_STRICT.txt	1710174270056F5 forCTG.txt.gz	0.1659	0.05507	3.013	0.002589	0.01513	0.002263	1	0.01183	0.002	0.008345	Myocardial infarction, strict	FALSE				361194	7018	354176	UK Biobank	https://docs.google.com/spreadsheets/d/1kvPoupSzsSFBNSztMzl04xMoSC3Kcx3CrjVf4yBmESU/edit?ts=5b5f17db#gid=227859291	PHESANT Transformation:NA-Notes:NA-Variable type:categorical-Phenotype ID:I9_MI_STRICT
categorical.I9_MI.txt	1710174270056F5 forCTG.txt.gz	0.1659	0.05507	3.013	0.002589	0.01513	0.002263	1	0.01183	0.002	0.008345	Myocardial infarction	FALSE				361194	7018	354176	UK Biobank	https://docs.google.com/spreadsheets/d/1kvPoupSzsSFBNSztMzl04xMoSC3Kcx3CrjVf4yBmESU/edit?ts=5b5f17db#gid=227859291	PHESANT Transformation:NA-Notes:NA-Variable type:categorical-Phenotype ID:I9_MI
categorical.I9_PAD.txt	1710174270056F5 forCTG.txt.gz	0.2599	0.1187	2.19	0.02851	0.003017	0.001631	1.004	0.009964	0.002951	0.007553	Peripheral artery disease	FALSE				361194	1230	359964	UK Biobank	https://docs.google.com/spreadsheets/d/1kvPoupSzsSFBNSztMzl04xMoSC3Kcx3CrjVf4yBmESU/edit?ts=5b5f17db#gid=227859291	PHESANT Transformation:NA-Notes:NA-Variable type:categorical-Phenotype ID:I9_PAD
categorical.I9_PHLETHROMBDVTLOW.txt	1710174270056F5 forCTG.txt.gz	0.1371	0.0772	1.776	0.07574	0.006529	0.001912	1.013	0.01251	-0.009333	0.008034	DVT of lower extremities	FALSE				361194	2116	359078	UK Biobank	https://docs.google.com/spreadsheets/d/1kvPoupSzsSFBNSztMzl04xMoSC3Kcx3CrjVf4yBmESU/edit?ts=5b5f17db#gid=227859291	PHESANT Transformation:NA-Notes:NA-Variable type:categorical-Phenotype ID:I9_PHLETHROMBDVTLOW
categorical.I9_UAP.txt	1710174270056F5 forCTG.txt.gz	0.2244	0.06178	3.632	0.000281	0.009161	0.001824	0.99	0.01037	0.008611	0.007958	Unstable angina pectoris	FALSE				361194	3439	357755	UK Biobank	https://docs.google.com/spreadsheets/d/1kvPoupSzsSFBNSztMzl04xMoSC3Kcx3CrjVf4yBmESU/edit?ts=5b5f17db#gid=227859291	PHESANT Transformation:NA-Notes:NA-Variable type:categorical-Phenotype ID:I9_UAP
categorical.I9_VTE.txt	1710174270056F5 forCTG.txt.gz	0.1404	0.06041	2.325	0.02008	0.011	0.002656	1.01	0.01654	-0.005516	0.008473	Venous thromboembolism	FALSE				361194	4620	356574	UK Biobank	https://docs.google.com/spreadsheets/d/1kvPoupSzsSFBNSztMzl04xMoSC3Kcx3CrjVf4yBmESU/edit?ts=5b5f17db#gid=227859291	PHESANT Transformation:NA-Notes:NA-Variable type:categorical-Phenotype ID:I9_VTE
categorical.IBD_ENDOMETRIOSIS.txt	1710174270056F5 forCTG.txt.gz	0.02145	0.08341	0.2571	0.7971	0.006102	0.001496	0.984	0.008561	0.01246	0.007902	Endometriosis, IBD co-morbidity	FALSE				361194	1516	359678	UK Biobank	https://docs.google.com/spreadsheets/d/1kvPoupSzsSFBNSztMzl04xMoSC3Kcx3CrjVf4yBmESU/edit?ts=5b5f17db#gid=227859291	PHESANT Transformation:NA-Notes:NA-Variable type:categorical-Phenotype ID:IBD_ENDOMETRIOSIS
categorical.ICDMAIN_ANY_ENTRY.txt	1710174270056F5 forCTG.txt.gz	0.4842	0.04357	11.11	1.07E-28	0.02285	0.00202	1.039	0.01109	0.00857	0.008135	Any ICDMAIN event in hilmo or causes of death	FALSE	Other (physical health)			361194	282009	79185	UK Biobank	https://docs.google.com/spreadsheets/d/1kvPoupSzsSFBNSztMzl04xMoSC3Kcx3CrjVf4yBmESU/edit?ts=5b5f17db#gid=227859291	PHESANT Transformation:NA-Notes:NA-Variable type:categorical-Phenotype ID:ICDMAIN_ANY_ENTRY
categorical.II_NEOPLASM.txt	1710174270056F5 forCTG.txt.gz	0.1875	0.05079	3.693	0.000222	0.01593	0.002177	1.01	0.0119	-0.000617	0.0087	Neoplasms	FALSE				361194	70178	291016	UK Biobank	https://docs.google.com/spreadsheets/d/1kvPoupSzsSFBNSztMzl04xMoSC3Kcx3CrjVf4yBmESU/edit?ts=5b5f17db#gid=227859291	PHESANT Transformation:NA-Notes:NA-Variable type:categorical-Phenotype ID:II_NEOPLASM
categorical.III_BLOOD_IMMUN.txt	1710174270056F5 forCTG.txt.gz	0.228	0.09891	2.305	0.02115	0.004086	0.001563	1	0.00854	0.004025	0.008057	Diseases of the blood and blood-forming organs and certain disorders involving the immune mechanism	FALSE				361194	10095	351099	UK Biobank	https://docs.google.com/spreadsheets/d/1kvPoupSzsSFBNSztMzl04xMoSC3Kcx3CrjVf4yBmESU/edit?ts=5b5f17db#gid=227859291	PHESANT Transformation:NA-Notes:NA-Variable type:categorical-Phenotype ID:III_BLOOD_IMMUN

categorical.ILD_DIFF_DG.txt	1710174270056F5forCTG.txt.gz	0.402	0.0592	6.79	1.12E-11	0.01232	0.001745	1.008	0.01029	0.007253	0.008718	ILD differential diagnosis	TRUE	Pulmonary	Physical health	Interstitial lung disease	361194	26710	334484	UK Biobank	https://docs.google.com/spreadsheets/d/1kvPoupSzsSFBNSztMzl04xMoSC3Kcx3CrjVf4yBmESU/edit?ts=5b5f17db#gid=227859291	PHESANT Transformation:NA-Notes:NA-Variable type:categorical-Phenotype ID:ILD_DIFF_DG
categorical.is_female.txt	1710174270056F5forCTG.txt.gz	0.0949	0.06377	1.488	0.1367	0.01091	0.00173	1.011	0.01032	0.002819	0.008415	is_female, based on inferred genetic sex	FALSE				361194	194174	167020	UK Biobank	https://docs.google.com/spreadsheets/d/1kvPoupSzsSFBNSztMzl04xMoSC3Kcx3CrjVf4yBmESU/edit?ts=5b5f17db#gid=227859291	PHESANT Transformation:NA-Notes:NA-Variable type:categorical-Phenotype ID:is_female
categorical.IV_ENDOCRIN_NUTRIT.txt	1710174270056F5forCTG.txt.gz	0.3387	0.1074	3.153	0.001614	0.004072	0.001559	1.006	0.009198	0.001243	0.007943	Endocrine, nutritional and metabolic diseases	FALSE				361194	7218	353976	UK Biobank	https://docs.google.com/spreadsheets/d/1kvPoupSzsSFBNSztMzl04xMoSC3Kcx3CrjVf4yBmESU/edit?ts=5b5f17db#gid=227859291	PHESANT Transformation:NA-Notes:NA-Variable type:categorical-Phenotype ID:IV_ENDOCRIN_NUTRIT
categorical.IX_CIRCULATORY.txt	1710174270056F5forCTG.txt.gz	0.2659	0.04468	5.952	2.66E-09	0.02214	0.002098	1.054	0.01179	0.0175	0.008535	Diseases of the circulatory system	FALSE	Cardiac			361194	60504	300690	UK Biobank	https://docs.google.com/spreadsheets/d/1kvPoupSzsSFBNSztMzl04xMoSC3Kcx3CrjVf4yBmESU/edit?ts=5b5f17db#gid=227859291	PHESANT Transformation:NA-Notes:NA-Variable type:categorical-Phenotype ID:IX_CIRCULATORY
categorical.J10_ASTHMA_MAIN.txt	1710174270056F5forCTG.txt.gz	0.3849	0.107	3.599	0.00032	0.004465	0.001434	1.003	0.008651	-0.005072	0.007496	Asthma	FALSE				361194	1993	359201	UK Biobank	https://docs.google.com/spreadsheets/d/1kvPoupSzsSFBNSztMzl04xMoSC3Kcx3CrjVf4yBmESU/edit?ts=5b5f17db#gid=227859291	PHESANT Transformation:NA-Notes:NA-Variable type:categorical-Phenotype ID:J10_ASTHMA_MAIN
categorical.J10_ASTHMA.txt	1710174270056F5forCTG.txt.gz	0.3849	0.107	3.599	0.00032	0.004465	0.001434	1.003	0.008651	-0.005072	0.007496	Asthma	FALSE				361194	1993	359201	UK Biobank	https://docs.google.com/spreadsheets/d/1kvPoupSzsSFBNSztMzl04xMoSC3Kcx3CrjVf4yBmESU/edit?ts=5b5f17db#gid=227859291	PHESANT Transformation:NA-Notes:NA-Variable type:categorical-Phenotype ID:J10_ASTHMA
categorical.J18.txt	1710174270056F5forCTG.txt.gz	0.6528	0.3275	1.994	0.0462	0.001507	0.001386	1.009	0.008622	0.003602	0.007616	Diagnoses - main ICD10: J18 Pneumonia, organism unspecified	FALSE				361194	4630	356564	UK Biobank	https://docs.google.com/spreadsheets/d/1kvPoupSzsSFBNSztMzl04xMoSC3Kcx3CrjVf4yBmESU/edit?ts=5b5f17db#gid=227859291	PHESANT Transformation:NA-Notes:NA-Variable type:categorical-Phenotype ID:J18
categorical.J33.txt	1710174270056F5forCTG.txt.gz	0.03105	0.0742	0.4185	0.6756	0.006809	0.002095	1.012	0.0115	-0.007418	0.008279	Diagnoses - main ICD10: J33 Nasal polyp	FALSE				361194	2207	358987	UK Biobank	https://docs.google.com/spreadsheets/d/1kvPoupSzsSFBNSztMzl04xMoSC3Kcx3CrjVf4yBmESU/edit?ts=5b5f17db#gid=227859291	PHESANT Transformation:NA-Notes:NA-Variable type:categorical-Phenotype ID:J33
categorical.J34.txt	1710174270056F5forCTG.txt.gz	0.1492	0.09835	1.517	0.1292	0.003591	0.001533	1	0.009076	0.00719	0.007247	Diagnoses - main ICD10: J34 Other disorders of nose and nasal sinuses	FALSE				361194	4438	356756	UK Biobank	https://docs.google.com/spreadsheets/d/1kvPoupSzsSFBNSztMzl04xMoSC3Kcx3CrjVf4yBmESU/edit?ts=5b5f17db#gid=227859291	PHESANT Transformation:NA-Notes:NA-Variable type:categorical-Phenotype ID:J34
categorical.J44.txt	1710174270056F5forCTG.txt.gz	0.2693	0.07893	3.412	0.000645	0.006183	0.00158	1.005	0.009229	0.01514	0.007512	Diagnoses - main ICD10: J44 Other chronic obstructive pulmonary disease	FALSE				361194	1531	359663	UK Biobank	https://docs.google.com/spreadsheets/d/1kvPoupSzsSFBNSztMzl04xMoSC3Kcx3CrjVf4yBmESU/edit?ts=5b5f17db#gid=227859291	PHESANT Transformation:NA-Notes:NA-Variable type:categorical-Phenotype ID:J44
categorical.J45.txt	1710174270056F5forCTG.txt.gz	0.4021	0.1238	3.249	0.001159	0.003909	0.001481	1.003	0.009045	-0.002947	0.007512	Diagnoses - main ICD10: J45 Asthma	FALSE				361194	1693	359501	UK Biobank	https://docs.google.com/spreadsheets/d/1kvPoupSzsSFBNSztMzl04xMoSC3Kcx3CrjVf4yBmESU/edit?ts=5b5f17db#gid=227859291	PHESANT Transformation:NA-Notes:NA-Variable type:categorical-Phenotype ID:J45
categorical.J47.txt	1710174270056F5forCTG.txt.gz	0.01742	0.09651	0.1805	0.8568	0.003809	0.001775	0.989	0.009555	0.01155	0.008604	Diagnoses - main ICD10: J47 Bronchiectasis	FALSE				361194	393	360801	UK Biobank	https://docs.google.com/spreadsheets/d/1kvPoupSzsSFBNSztMzl04xMoSC3Kcx3CrjVf4yBmESU/edit?ts=5b5f17db#gid=227859291	PHESANT Transformation:NA-Notes:NA-Variable type:categorical-Phenotype ID:J47
categorical.J84.txt	1710174270056F5forCTG.txt.gz	0.1399	0.107	1.307	0.1911	0.00319	0.001544	0.998	0.009784	-0.005403	0.007557	Diagnoses - main ICD10: J84 Other interstitial pulmonary diseases	FALSE				361194	342	360852	UK Biobank	https://docs.google.com/spreadsheets/d/1kvPoupSzsSFBNSztMzl04xMoSC3Kcx3CrjVf4yBmESU/edit?ts=5b5f17db#gid=227859291	PHESANT Transformation:NA-Notes:NA-Variable type:categorical-Phenotype ID:J84
categorical.K11_APPENDIX.txt	1710174270056F5forCTG.txt.gz	-0.1053	0.08749	-1.203	0.2288	0.004224	0.001606	0.997	0.00976	0.01268	0.007882	Diseases of appendix	FALSE				361194	2953	358241	UK Biobank	https://docs.google.com/spreadsheets/d/1kvPoupSzsSFBNSztMzl04xMoSC3Kcx3CrjVf4yBmESU/edit?ts=5b5f17db#gid=227859291	PHESANT Transformation:NA-Notes:NA-Variable type:categorical-Phenotype ID:K11_APPENDIX
categorical.K11_BARRET.txt	1710174270056F5forCTG.txt.gz	0.2526	0.07314	3.454	0.000552	0.006365	0.001721	0.998	0.009369	-0.01242	0.007461	Barret oesophagus	FALSE				361194	1791	359403	UK Biobank	https://docs.google.com/spreadsheets/d/1kvPoupSzsSFBNSztMzl04xMoSC3Kcx3CrjVf4yBmESU/edit?ts=5b5f17db#gid=227859291	PHESANT Transformation:NA-Notes:NA-Variable type:categorical-Phenotype ID:K11_BARRET
categorical.K11_COELIAC.txt	1710174270056F5forCTG.txt.gz	-0.03572	0.1066	-0.3349	0.7377	0.003609	0.00172	0.999	0.01008	0.007489	0.008013	Coeliac disease	FALSE				361194	842	360352	UK Biobank	https://docs.google.com/spreadsheets/d/1kvPoupSzsSFBNSztMzl04xMoSC3Kcx3CrjVf4yBmESU/edit?ts=5b5f17db#gid=227859291	PHESANT Transformation:NA-Notes:NA-Variable type:categorical-Phenotype ID:K11_COELIAC
categorical.K11_GALLBILPANC.txt	1710174270056F5forCTG.txt.gz	0.2152	0.06558	3.282	0.001031	0.0116	0.002267	1.06	0.02078	0.01724	0.008276	Disorders of gallbladder, biliary tract and pancreas	FALSE				361194	13922	347272	UK Biobank	https://docs.google.com/spreadsheets/d/1kvPoupSzsSFBNSztMzl04xMoSC3Kcx3CrjVf4yBmESU/edit?ts=5b5f17db#gid=227859291	PHESANT Transformation:NA-Notes:NA-Variable type:categorical-Phenotype ID:K11_GALLBILPANC
categorical.K11_HERNIA.txt	1710174270056F5forCTG.txt.gz	0.2891	0.05506	5.251	1.51E-07	0.01607	0.002552	1.029	0.01264	-0.01322	0.008866	Hernia	FALSE	Other (physical health)			361194	26197	334997	UK Biobank	https://docs.google.com/spreadsheets/d/1kvPoupSzsSFBNSztMzl04xMoSC3Kcx3CrjVf4yBmESU/edit?ts=5b5f17db#gid=227859291	PHESANT Transformation:NA-Notes:NA-Variable type:categorical-Phenotype ID:K11_HERNIA
categorical.K11_OESULC.txt	1710174270056F5forCTG.txt.gz	0.372	0.08807	4.224	2.40E-05	0.005255	0.001683	1.008	0.0102	-0.01353	0.007506	Ulcer of oesophagus	FALSE	Gastric			361194	3098	358096	UK Biobank	https://docs.google.com/spreadsheets/d/1kvPoupSzsSFBNSztMzl04xMoSC3Kcx3CrjVf4yBmESU/edit?ts=5b5f17db#gid=227859291	PHESANT Transformation:NA-Notes:NA-Variable type:categorical-Phenotype ID:K11_OESULC
categorical.K11_OTHGASTR.txt	1710174270056F5forCTG.txt.gz	0.2987	0.06476	4.611	4.00E-06	0.008471	0.001696	1	0.009944	0.003891	0.007759	Other gastritis (incl. Duodenitis)	FALSE	Gastric			361194	10518	350676	UK Biobank	https://docs.google.com/spreadsheets/d/1kvPoupSzsSFBNSztMzl04xMoSC3Kcx3CrjVf4yBmESU/edit?ts=5b5f17db#gid=227859291	PHESANT Transformation:NA-Notes:NA-Variable type:categorical-Phenotype ID:K11_OTHGASTR
categorical.K20.txt	1710174270056F5forCTG.txt.gz	0.4932	0.1555	3.172	0.001515	0.002963	0.001479	1.017	0.009274	0.0006688	0.007478	Diagnoses - main ICD10: K20 Oesophagitis	FALSE				361194	4799	356395	UK Biobank	https://docs.google.com/spreadsheets/d/1kvPoupSzsSFBNSztMzl04xMoSC3Kcx3CrjVf4yBmESU/edit?ts=5b5f17db#gid=227859291	PHESANT Transformation:NA-Notes:NA-Variable type:categorical-Phenotype ID:K20
categorical.K21.txt	1710174270056F5forCTG.txt.gz	0.3927	0.05862	6.699	2.10E-11	0.009391	0.001636	1.007	0.008959	-0.002701	0.006807	Diagnoses - main ICD10: K21 Gastro-oesophageal reflux disease	TRUE	Gastric		Gastro-oesophageal reflux	361194	10743	350451	UK Biobank	https://docs.google.com/spreadsheets/d/1kvPoupSzsSFBNSztMzl04xMoSC3Kcx3CrjVf4yBmESU/edit?ts=5b5f17db#gid=227859291	PHESANT Transformation:NA-Notes:NA-Variable type:categorical-Phenotype ID:K21
categorical.K22.txt	1710174270056F5forCTG.txt.gz	0.3714	0.07532	4.93	8.21E-07	0.006851	0.001779	1.012	0.01052	-0.01455	0.007868	Diagnoses - main ICD10: K22 Other diseases of oesophagus	FALSE	Gastric			361194	5494	355700	UK Biobank	https://docs.google.com/spreadsheets/d/1kvPoupSzsSFBNSztMzl04xMoSC3Kcx3CrjVf4yBmESU/edit?ts=5b5f17db#gid=227859291	PHESANT Transformation:NA-Notes:NA-Variable type:categorical-Phenotype ID:K22
categorical.K29.txt	1710174270056F5forCTG.txt.gz	0.3207	0.06354	5.047	4.49E-07	0.009161	0.001766	1.014	0.009863	0.004975	0.007778	Diagnoses - main ICD10: K29 Gastritis and duodenitis	FALSE	Gastric			361194	12678	348516	UK Biobank	https://docs.google.com/spreadsheets/d/1kvPoupSzsSFBNSztMzl04xMoSC3Kcx3CrjVf4yBmESU/edit?ts=5b5f17db#gid=227859291	PHESANT Transformation:NA-Notes:NA-Variable type:categorical-Phenotype ID:K29
categorical.K30.txt	1710174270056F5forCTG.txt.gz	0.2324	0.1365	1.703	0.08856	0.002602	0.001609	1.011	0.009549	0.01086	0.008172	Diagnoses - main ICD10: K30 Dyspepsia	FALSE				361194	7586	353608	UK Biobank	https://docs.google.com/spreadsheets/d/1kvPoupSzsSFBNSztMzl04xMoSC3Kcx3CrjVf4yBmESU/edit?ts=5b5f17db#gid=227859291	PHESANT Transformation:NA-Notes:NA-Variable type:categorical-Phenotype ID:K30
categorical.K35.txt	1710174270056F5forCTG.txt.gz	-0.05069	0.09471	-0.5352	0.5925	0.003573	0.001675	1	0.01034	0.006648	0.007701	Diagnoses - main ICD10: K35 Acute appendicitis	FALSE				361194	2404	358790	UK Biobank	https://docs.google.com/spreadsheets/d/1kvPoupSzsSFBNSztMzl04xMoSC3Kcx3CrjVf4yBmESU/edit?ts=5b5f17db#gid=227859291	PHESANT Transformation:NA-Notes:NA-Variable type:categorical-Phenotype ID:K35
categorical.K40.txt	1710174270056F5forCTG.txt.gz	0.07972	0.04577	1.742	0.08153	0.02049	0.002805	1.009	0.01139	-0.01473	0.008514	Diagnoses - main ICD10: K40 Inguinal hernia	FALSE				361194	13147	348047	UK Biobank	https://docs.google.com/spreadsheets/d/1kvPoupSzsSFBNSztMzl04xMoSC3Kcx3CrjVf4yBmESU/edit?ts=5b5f17db#gid=227859291	PHESANT Transformation:NA-Notes:NA-Variable type:categorical-Phenotype ID:K40
categorical.K42.txt	1710174270056F5forCTG.txt.gz	0.1937	0.08735	2.218	0.02657	0.005215	0.001994	1.016	0.01131	0.006581	0.007661	Diagnoses - main ICD10: K42 Umbilical hernia	FALSE				361194	2528	358666	UK Biobank	https://docs.google.com/spreadsheets/d/1kvPoupSzsSFBNSztMzl04xMoSC3Kcx3CrjVf4yBmESU/edit?ts=5b5f17db#gid=227859291	PHESANT Transformation:NA-Notes:NA-Variable type:categorical-Phenotype ID:K42
categorical.K43.txt	1710174270056F5forCTG.txt.gz	0.4439	0.0936	4.743	2.11E-06	0.005277	0.001661	0.996	0.009382	-0.01623	0.007605	Diagnoses - main ICD10: K43 Ventral hernia	FALSE	Other (physical health)			361194	2249	358945	UK Biobank	https://docs.google.com/spreadsheets/d/1kvPoupSzsSFBNSztMzl04xMoSC3Kcx3CrjVf4yBmESU/edit?ts=5b5f17db#gid=227859291	PHESANT Transformation:NA-Notes:NA-Variable type:categorical-Phenotype ID:K43

categorical.K44.txt	1710174270056F5forCTG.txt.gz	0.3906	0.09176	4.257	2.07E-05	0.005959	0.001468	1.014	0.009479	-0.001013	0.009035	Diagnoses - main ICD10: K44 Diaphragmatic hernia	FALSE	Other (physical health)			361194	8042	353152	UK Biobank	https://docs.google.com/spreadsheets/d/1kvPoupSzsSFBNSztMzl04xMoSC3Kcx3CrjVf4yBmESU/edit?ts=5b5f17db#gid=227859291	PHESANT Transformation:NA-Notes:NA-Variable type:categorical-Phenotype ID:K44
categorical.K50.txt	1710174270056F5forCTG.txt.gz	0.02981	0.08975	0.3322	0.7398	0.004465	0.001477	1.007	0.009566	0.01332	0.008663	Diagnoses - main ICD10: K50 Crohn's disease [regional enteritis]	FALSE				361194	968	360226	UK Biobank	https://docs.google.com/spreadsheets/d/1kvPoupSzsSFBNSztMzl04xMoSC3Kcx3CrjVf4yBmESU/edit?ts=5b5f17db#gid=227859291	PHESANT Transformation:NA-Notes:NA-Variable type:categorical-Phenotype ID:K50
categorical.K51.txt	1710174270056F5forCTG.txt.gz	0.007122	0.07503	0.09492	0.9244	0.006036	0.001873	1.016	0.01155	0.005265	0.007799	Diagnoses - main ICD10: K51 Ulcerative colitis	FALSE				361194	2143	359051	UK Biobank	https://docs.google.com/spreadsheets/d/1kvPoupSzsSFBNSztMzl04xMoSC3Kcx3CrjVf4yBmESU/edit?ts=5b5f17db#gid=227859291	PHESANT Transformation:NA-Notes:NA-Variable type:categorical-Phenotype ID:K51
categorical.K52.txt	1710174270056F5forCTG.txt.gz	0.3733	0.1048	3.561	0.00037	0.004554	0.001647	1.004	0.009623	0.0008636	0.007274	Diagnoses - main ICD10: K52 Other non-infective gastro-enteritis and colitis	FALSE				361194	8757	352437	UK Biobank	https://docs.google.com/spreadsheets/d/1kvPoupSzsSFBNSztMzl04xMoSC3Kcx3CrjVf4yBmESU/edit?ts=5b5f17db#gid=227859291	PHESANT Transformation:NA-Notes:NA-Variable type:categorical-Phenotype ID:K52
categorical.K57.txt	1710174270056F5forCTG.txt.gz	0.2177	0.04415	4.93	8.22E-07	0.0218	0.002216	1.011	0.01158	-0.001033	0.008129	Diagnoses - main ICD10: K57 Diverticular disease of intestine	FALSE	Gastric			361194	12662	348532	UK Biobank	https://docs.google.com/spreadsheets/d/1kvPoupSzsSFBNSztMzl04xMoSC3Kcx3CrjVf4yBmESU/edit?ts=5b5f17db#gid=227859291	PHESANT Transformation:NA-Notes:NA-Variable type:categorical-Phenotype ID:K57
categorical.K60.txt	1710174270056F5forCTG.txt.gz	0.217	0.07387	2.938	0.0033	0.006104	0.001624	0.986	0.01005	-0.006472	0.007579	Diagnoses - main ICD10: K60 Fissure and fistula of anal and rectal regions	FALSE				361194	2109	359085	UK Biobank	https://docs.google.com/spreadsheets/d/1kvPoupSzsSFBNSztMzl04xMoSC3Kcx3CrjVf4yBmESU/edit?ts=5b5f17db#gid=227859291	PHESANT Transformation:NA-Notes:NA-Variable type:categorical-Phenotype ID:K60
categorical.K62.txt	1710174270056F5forCTG.txt.gz	0.4179	0.09637	4.336	1.45E-05	0.005192	0.00168	0.996	0.009483	-0.004399	0.008397	Diagnoses - main ICD10: K62 Other diseases of anus and rectum	FALSE	Other (physical health)			361194	13882	347312	UK Biobank	https://docs.google.com/spreadsheets/d/1kvPoupSzsSFBNSztMzl04xMoSC3Kcx3CrjVf4yBmESU/edit?ts=5b5f17db#gid=227859291	PHESANT Transformation:NA-Notes:NA-Variable type:categorical-Phenotype ID:K62
categorical.K63.txt	1710174270056F5forCTG.txt.gz	0.2673	0.09889	2.703	0.006876	0.004245	0.001569	1.014	0.01014	0.007966	0.007008	Diagnoses - main ICD10: K63 Other diseases of intestine	FALSE				361194	8041	353153	UK Biobank	https://docs.google.com/spreadsheets/d/1kvPoupSzsSFBNSztMzl04xMoSC3Kcx3CrjVf4yBmESU/edit?ts=5b5f17db#gid=227859291	PHESANT Transformation:NA-Notes:NA-Variable type:categorical-Phenotype ID:K63
categorical.K76.txt	1710174270056F5forCTG.txt.gz	0.1667	0.09216	1.808	0.07056	0.004759	0.001646	0.987	0.01068	-0.004264	0.007724	Diagnoses - main ICD10: K76 Other diseases of liver	FALSE				361194	576	360618	UK Biobank	https://docs.google.com/spreadsheets/d/1kvPoupSzsSFBNSztMzl04xMoSC3Kcx3CrjVf4yBmESU/edit?ts=5b5f17db#gid=227859291	PHESANT Transformation:NA-Notes:NA-Variable type:categorical-Phenotype ID:K76
categorical.K80.txt	1710174270056F5forCTG.txt.gz	0.1993	0.0716	2.783	0.005388	0.0104	0.002249	1.059	0.0207	0.01472	0.008414	Diagnoses - main ICD10: K80 Cholelithiasis	FALSE				361194	10520	350674	UK Biobank	https://docs.google.com/spreadsheets/d/1kvPoupSzsSFBNSztMzl04xMoSC3Kcx3CrjVf4yBmESU/edit?ts=5b5f17db#gid=227859291	PHESANT Transformation:NA-Notes:NA-Variable type:categorical-Phenotype ID:K80
categorical.K81.txt	1710174270056F5forCTG.txt.gz	0.2403	0.1751	1.373	0.1699	0.001662	0.001537	1.007	0.01045	0.007797	0.007818	Diagnoses - main ICD10: K81 Cholecystitis	FALSE				361194	1930	359264	UK Biobank	https://docs.google.com/spreadsheets/d/1kvPoupSzsSFBNSztMzl04xMoSC3Kcx3CrjVf4yBmESU/edit?ts=5b5f17db#gid=227859291	PHESANT Transformation:NA-Notes:NA-Variable type:categorical-Phenotype ID:K81
categorical.K85.txt	1710174270056F5forCTG.txt.gz	0.1476	0.09634	1.532	0.1254	0.00395	0.001558	0.994	0.009055	0.006103	0.007871	Diagnoses - main ICD10: K85 Acute pancreatitis	FALSE				361194	1292	359902	UK Biobank	https://docs.google.com/spreadsheets/d/1kvPoupSzsSFBNSztMzl04xMoSC3Kcx3CrjVf4yBmESU/edit?ts=5b5f17db#gid=227859291	PHESANT Transformation:NA-Notes:NA-Variable type:categorical-Phenotype ID:K85
categorical.K90.txt	1710174270056F5forCTG.txt.gz	0.008531	0.1039	0.08211	0.9346	0.003587	0.001732	0.999	0.009828	0.003505	0.00796	Diagnoses - main ICD10: K90 Intestinal malabsorption	FALSE				361194	922	360272	UK Biobank	https://docs.google.com/spreadsheets/d/1kvPoupSzsSFBNSztMzl04xMoSC3Kcx3CrjVf4yBmESU/edit?ts=5b5f17db#gid=227859291	PHESANT Transformation:NA-Notes:NA-Variable type:categorical-Phenotype ID:K90
categorical.KNEE_ARTHROSIS.txt	1710174270056F5forCTG.txt.gz	0.2735	0.04554	6.007	1.90E-09	0.02257	0.00232	1.009	0.01198	0.001856	0.009569	Gonarthrosis [arthrosis of knee](FG)	FALSE	Skeletal			361194	11900	349294	UK Biobank	https://docs.google.com/spreadsheets/d/1kvPoupSzsSFBNSztMzl04xMoSC3Kcx3CrjVf4yBmESU/edit?ts=5b5f17db#gid=227859291	PHESANT Transformation:NA-Notes:NA-Variable type:categorical-Phenotype ID:KNEE_ARTHROSIS
categorical.KRA_PSY_ANYMENTAL.txt	1710174270056F5forCTG.txt.gz	0.1779	0.07716	2.306	0.02111	0.006734	0.001686	0.999	0.009974	0.002619	0.008283	Any mental disorder	FALSE				361194	4304	356890	UK Biobank	https://docs.google.com/spreadsheets/d/1kvPoupSzsSFBNSztMzl04xMoSC3Kcx3CrjVf4yBmESU/edit?ts=5b5f17db#gid=227859291	PHESANT Transformation:NA-Notes:NA-Variable type:categorical-Phenotype ID:KRA_PSY_ANYMENTAL
categorical.KRA_PSY_MOOD.txt	1710174270056F5forCTG.txt.gz	0.004686	0.07879	0.05948	0.9526	0.006518	0.001662	0.99	0.009621	0.004721	0.008444	Mood disorders	FALSE				361194	1546	359648	UK Biobank	https://docs.google.com/spreadsheets/d/1kvPoupSzsSFBNSztMzl04xMoSC3Kcx3CrjVf4yBmESU/edit?ts=5b5f17db#gid=227859291	PHESANT Transformation:NA-Notes:NA-Variable type:categorical-Phenotype ID:KRA_PSY_MOOD
categorical.L03.txt	1710174270056F5forCTG.txt.gz	0.2952	0.07998	3.691	0.000224	0.006736	0.001842	0.993	0.01022	-0.003871	0.008155	Diagnoses - main ICD10: L03 Cellulitis	FALSE				361194	4247	356947	UK Biobank	https://docs.google.com/spreadsheets/d/1kvPoupSzsSFBNSztMzl04xMoSC3Kcx3CrjVf4yBmESU/edit?ts=5b5f17db#gid=227859291	PHESANT Transformation:NA-Notes:NA-Variable type:categorical-Phenotype ID:L03
categorical.L12_ACTINKERA.txt	1710174270056F5forCTG.txt.gz	0.01893	0.09586	0.1975	0.8435	0.003981	0.001545	0.995	0.009595	-0.005935	0.00774	Actinic keratosis	FALSE				361194	1349	359845	UK Biobank	https://docs.google.com/spreadsheets/d/1kvPoupSzsSFBNSztMzl04xMoSC3Kcx3CrjVf4yBmESU/edit?ts=5b5f17db#gid=227859291	PHESANT Transformation:NA-Notes:NA-Variable type:categorical-Phenotype ID:L12_ACTINKERA
categorical.L12_NONIONRADISKIN.txt	1710174270056F5forCTG.txt.gz	0.04197	0.09136	0.4593	0.646	0.004621	0.001578	0.989	0.009439	-0.007374	0.007876	Skin changes due to chronic exposure to nonionizing radiation	FALSE				361194	1501	359693	UK Biobank	https://docs.google.com/spreadsheets/d/1kvPoupSzsSFBNSztMzl04xMoSC3Kcx3CrjVf4yBmESU/edit?ts=5b5f17db#gid=227859291	PHESANT Transformation:NA-Notes:NA-Variable type:categorical-Phenotype ID:L12_NONIONRADISKIN
categorical.L12_PSORIASIS.txt	1710174270056F5forCTG.txt.gz	0.227	0.1048	2.167	0.03021	0.00357	0.001628	0.996	0.01008	-0.006629	0.007278	Psoriasis	FALSE				361194	487	360707	UK Biobank	https://docs.google.com/spreadsheets/d/1kvPoupSzsSFBNSztMzl04xMoSC3Kcx3CrjVf4yBmESU/edit?ts=5b5f17db#gid=227859291	PHESANT Transformation:NA-Notes:NA-Variable type:categorical-Phenotype ID:L12_PSORIASIS
categorical.L40.txt	1710174270056F5forCTG.txt.gz	0.227	0.1048	2.167	0.03021	0.00357	0.001628	0.996	0.01008	-0.006629	0.007278	Diagnoses - main ICD10: L40 Psoriasis	FALSE				361194	474	360720	UK Biobank	https://docs.google.com/spreadsheets/d/1kvPoupSzsSFBNSztMzl04xMoSC3Kcx3CrjVf4yBmESU/edit?ts=5b5f17db#gid=227859291	PHESANT Transformation:NA-Notes:NA-Variable type:categorical-Phenotype ID:L40
categorical.L57.txt	1710174270056F5forCTG.txt.gz	0.04197	0.09136	0.4593	0.646	0.004621	0.001578	0.989	0.009439	-0.007374	0.007876	Diagnoses - main ICD10: L57 Skin changes due to chronic exposure to nonionising radiation	FALSE				361194	1447	359747	UK Biobank	https://docs.google.com/spreadsheets/d/1kvPoupSzsSFBNSztMzl04xMoSC3Kcx3CrjVf4yBmESU/edit?ts=5b5f17db#gid=227859291	PHESANT Transformation:NA-Notes:NA-Variable type:categorical-Phenotype ID:L57
categorical.L72.txt	1710174270056F5forCTG.txt.gz	0.09851	0.08722	1.129	0.2587	0.00479	0.001914	1.012	0.0122	0.001634	0.008409	Diagnoses - main ICD10: L72 Follicular cysts of skin and subcutaneous tissue	FALSE				361194	6644	354550	UK Biobank	https://docs.google.com/spreadsheets/d/1kvPoupSzsSFBNSztMzl04xMoSC3Kcx3CrjVf4yBmESU/edit?ts=5b5f17db#gid=227859291	PHESANT Transformation:NA-Notes:NA-Variable type:categorical-Phenotype ID:L72
categorical.LUNG_CANCER_MESOT.txt	1710174270056F5forCTG.txt.gz	0.04233	0.09618	0.4401	0.6598	0.004089	0.001496	0.998	0.009111	0.01442	0.007985	Lung cancer and mesothelioma	FALSE				361194	2007	359187	UK Biobank	https://docs.google.com/spreadsheets/d/1kvPoupSzsSFBNSztMzl04xMoSC3Kcx3CrjVf4yBmESU/edit?ts=5b5f17db#gid=227859291	PHESANT Transformation:NA-Notes:NA-Variable type:categorical-Phenotype ID:LUNG_CANCER_MESOT
categorical.LUNG_CANCER.txt	1710174270056F5forCTG.txt.gz	0.04233	0.09618	0.4401	0.6598	0.004089	0.001496	0.998	0.009111	0.01442	0.007985	Lung cancer and mesothelioma	FALSE				361194	2007	359187	UK Biobank	https://docs.google.com/spreadsheets/d/1kvPoupSzsSFBNSztMzl04xMoSC3Kcx3CrjVf4yBmESU/edit?ts=5b5f17db#gid=227859291	PHESANT Transformation:NA-Notes:NA-Variable type:categorical-Phenotype ID:LUNG_CANCER
categorical.M13_ADHCAPSULITIS.txt	1710174270056F5forCTG.txt.gz	0.2127	0.111	1.915	0.05544	0.0031	0.001539	1.003	0.009348	-0.002299	0.007781	Adhesive capsulitis of shoulder	FALSE				361194	1198	359996	UK Biobank	https://docs.google.com/spreadsheets/d/1kvPoupSzsSFBNSztMzl04xMoSC3Kcx3CrjVf4yBmESU/edit?ts=5b5f17db#gid=227859291	PHESANT Transformation:NA-Notes:NA-Variable type:categorical-Phenotype ID:M13_ADHCAPSULITIS

categorical.M13_ARTHRITISNAS.txt	1710174270056F5forCTG.txt.gz	0.5237	0.2622	1.997	0.0458	0.001859	0.001577	0.999	0.009502	-0.01115	0.008012	Ohter specific/unspecified arthritis	FALSE				361194	1082	360112	UK Biobank	https://docs.google.com/spreadsheets/d/1kvPoupSzsSFBNSztMzl04xMoSC3Kcx3CrjVf4yBmESU/edit?ts=5b5f17db#gid=227859291	PHESANT Transformation:NA-Notes:NA-Variable type:categorical-Phenotype ID:M13_ARTHRITISNAS
categorical.M13_ARTHROSIS_OTH.txt	1710174270056F5forCTG.txt.gz	0.1847	0.09448	1.955	0.05064	0.006062	0.001861	1.009	0.01119	0.01825	0.008533	Other arthrosis	FALSE				361194	5168	356026	UK Biobank	https://docs.google.com/spreadsheets/d/1kvPoupSzsSFBNSztMzl04xMoSC3Kcx3CrjVf4yBmESU/edit?ts=5b5f17db#gid=227859291	PHESANT Transformation:NA-Notes:NA-Variable type:categorical-Phenotype ID:M13_ARTHROSIS_OTH
categorical.M13_ARTHROSIS.txt	1710174270056F5forCTG.txt.gz	0.2629	0.04126	6.371	1.88E-10	0.02968	0.002571	1.014	0.01296	-0.001933	0.009545	#Arthrosis	TRUE	Skeletal	Physical health	Arthrosis	361194	24977	336217	UK Biobank	https://docs.google.com/spreadsheets/d/1kvPoupSzsSFBNSztMzl04xMoSC3Kcx3CrjVf4yBmESU/edit?ts=5b5f17db#gid=227859291	PHESANT Transformation:NA-Notes:NA-Variable type:categorical-Phenotype ID:M13_ARTHROSIS
categorical.M13_DISSYNOTENDNAS.txt	1710174270056F5forCTG.txt.gz	0.2134	0.1216	1.756	0.0791	0.003426	0.001549	0.981	0.01007	-0.001867	0.009189	Other specified/unspecified disorders of synovium and tendon +Other specified/unspecified bursopathies	FALSE				361194	343	360851	UK Biobank	https://docs.google.com/spreadsheets/d/1kvPoupSzsSFBNSztMzl04xMoSC3Kcx3CrjVf4yBmESU/edit?ts=5b5f17db#gid=227859291	PHESANT Transformation:NA-Notes:NA-Variable type:categorical-Phenotype ID:M13_DISSYNOTENDNAS
categorical.M13_DORSALGIA.txt	1710174270056F5forCTG.txt.gz	0.39	0.05437	7.173	7.33E-13	0.01454	0.001959	0.992	0.01109	0.009998	0.008606	Dorsalgia	FALSE	Pain			361194	8799	352395	UK Biobank	https://docs.google.com/spreadsheets/d/1kvPoupSzsSFBNSztMzl04xMoSC3Kcx3CrjVf4yBmESU/edit?ts=5b5f17db#gid=227859291	PHESANT Transformation:NA-Notes:NA-Variable type:categorical-Phenotype ID:M13_DORSALGIA
categorical.M13_DORSALGIANAS.txt	1710174270056F5forCTG.txt.gz	0.2965	0.09211	3.219	0.001286	0.004784	0.001637	0.993	0.009559	0.01157	0.0077	Other/unspecified dorsalgia	FALSE				361194	2118	359076	UK Biobank	https://docs.google.com/spreadsheets/d/1kvPoupSzsSFBNSztMzl04xMoSC3Kcx3CrjVf4yBmESU/edit?ts=5b5f17db#gid=227859291	PHESANT Transformation:NA-Notes:NA-Variable type:categorical-Phenotype ID:M13_DORSALGIANAS
categorical.M13_DORSOPATHYOTH.txt	1710174270056F5forCTG.txt.gz	0.001773	0.1119	0.01584	0.9874	0.002634	0.001499	0.985	0.009881	0.008261	0.007116	Other dorsopathies, not elsewhere classified	FALSE				361194	360	360834	UK Biobank	https://docs.google.com/spreadsheets/d/1kvPoupSzsSFBNSztMzl04xMoSC3Kcx3CrjVf4yBmESU/edit?ts=5b5f17db#gid=227859291	PHESANT Transformation:NA-Notes:NA-Variable type:categorical-Phenotype ID:M13_DORSOPATHYOTH
categorical.M13_DUPUTRYEN.txt	1710174270056F5forCTG.txt.gz	0.1068	0.05707	1.871	0.0614	0.01531	0.003324	1.027	0.02171	-0.007026	0.00846	Palmar fascial fibromatosis [Dupuytren]	FALSE				361194	2948	358246	UK Biobank	https://docs.google.com/spreadsheets/d/1kvPoupSzsSFBNSztMzl04xMoSC3Kcx3CrjVf4yBmESU/edit?ts=5b5f17db#gid=227859291	PHESANT Transformation:NA-Notes:NA-Variable type:categorical-Phenotype ID:M13_DUPUTRYEN
categorical.M13_FIBROBLASTIC.txt	1710174270056F5forCTG.txt.gz	0.1051	0.05974	1.76	0.07842	0.01436	0.003357	1.028	0.02144	-0.004748	0.008437	Fibroblastic disorders	FALSE				361194	3190	358004	UK Biobank	https://docs.google.com/spreadsheets/d/1kvPoupSzsSFBNSztMzl04xMoSC3Kcx3CrjVf4yBmESU/edit?ts=5b5f17db#gid=227859291	PHESANT Transformation:NA-Notes:NA-Variable type:categorical-Phenotype ID:M13_FIBROBLASTIC
categorical.M13_GANGLION.txt	1710174270056F5forCTG.txt.gz	0.2596	0.1287	2.017	0.04367	0.002846	0.001553	0.996	0.01003	-0.000798	0.007274	Ganglion	FALSE				361194	2239	358955	UK Biobank	https://docs.google.com/spreadsheets/d/1kvPoupSzsSFBNSztMzl04xMoSC3Kcx3CrjVf4yBmESU/edit?ts=5b5f17db#gid=227859291	PHESANT Transformation:NA-Notes:NA-Variable type:categorical-Phenotype ID:M13_GANGLION
categorical.M13_HALLUXRIGIDUS.txt	1710174270056F5forCTG.txt.gz	0.1458	0.07457	1.956	0.0505	0.006914	0.001649	0.985	0.009802	-0.006952	0.008234	Hallux rigidus	FALSE				361194	1130	360064	UK Biobank	https://docs.google.com/spreadsheets/d/1kvPoupSzsSFBNSztMzl04xMoSC3Kcx3CrjVf4yBmESU/edit?ts=5b5f17db#gid=227859291	PHESANT Transformation:NA-Notes:NA-Variable type:categorical-Phenotype ID:M13_HALLUXRIGIDUS
categorical.M13_HALLUXVALGUS.txt	1710174270056F5forCTG.txt.gz	0.05833	0.05426	1.075	0.2823	0.01283	0.001999	1.003	0.01137	0.00765	0.008274	Hallux valgus (acquired)	FALSE				361194	5370	355824	UK Biobank	https://docs.google.com/spreadsheets/d/1kvPoupSzsSFBNSztMzl04xMoSC3Kcx3CrjVf4yBmESU/edit?ts=5b5f17db#gid=227859291	PHESANT Transformation:NA-Notes:NA-Variable type:categorical-Phenotype ID:M13_HALLUXVALGUS
categorical.M13_HAMMERTOE.txt	1710174270056F5forCTG.txt.gz	0.1575	0.1303	1.208	0.2269	0.002018	0.001398	0.994	0.009389	-0.004779	0.007836	Other hammer toe(s) (acquired)	FALSE				361194	838	360356	UK Biobank	https://docs.google.com/spreadsheets/d/1kvPoupSzsSFBNSztMzl04xMoSC3Kcx3CrjVf4yBmESU/edit?ts=5b5f17db#gid=227859291	PHESANT Transformation:NA-Notes:NA-Variable type:categorical-Phenotype ID:M13_HAMMERTOE
categorical.M13_IMPINGEMENT.txt	1710174270056F5forCTG.txt.gz	0.1982	0.07884	2.514	0.01193	0.004158	0.001465	1.002	0.009769	0.01594	0.006693	Impingement syndrome of shoulder	FALSE				361194	3420	357774	UK Biobank	https://docs.google.com/spreadsheets/d/1kvPoupSzsSFBNSztMzl04xMoSC3Kcx3CrjVf4yBmESU/edit?ts=5b5f17db#gid=227859291	PHESANT Transformation:NA-Notes:NA-Variable type:categorical-Phenotype ID:M13_IMPINGEMENT
categorical.M13_JOINTOTH.txt	1710174270056F5forCTG.txt.gz	0.6939	0.1517	4.576	4.74E-06	0.004283	0.001729	1.009	0.009939	-0.01527	0.00752	Other specific joint derangements/joint disorders	FALSE	Skeletal			361194	7943	353251	UK Biobank	https://docs.google.com/spreadsheets/d/1kvPoupSzsSFBNSztMzl04xMoSC3Kcx3CrjVf4yBmESU/edit?ts=5b5f17db#gid=227859291	PHESANT Transformation:NA-Notes:NA-Variable type:categorical-Phenotype ID:M13_JOINTOTH
categorical.M13_LATERALEPICOND.txt	1710174270056F5forCTG.txt.gz	0.2567	0.1131	2.27	0.0232	0.003183	0.001484	0.989	0.009456	-0.002371	0.007728	Lateral epicondylitis	FALSE				361194	525	360669	UK Biobank	https://docs.google.com/spreadsheets/d/1kvPoupSzsSFBNSztMzl04xMoSC3Kcx3CrjVf4yBmESU/edit?ts=5b5f17db#gid=227859291	PHESANT Transformation:NA-Notes:NA-Variable type:categorical-Phenotype ID:M13_LATERALEPICOND
categorical.M13_LOWBACKPAIN.txt	1710174270056F5forCTG.txt.gz	0.3697	0.05607	6.593	4.30E-11	0.01079	0.001821	0.987	0.01079	0.002208	0.008712	Low back pain	FALSE	Pain			361194	5423	355771	UK Biobank	https://docs.google.com/spreadsheets/d/1kvPoupSzsSFBNSztMzl04xMoSC3Kcx3CrjVf4yBmESU/edit?ts=5b5f17db#gid=227859291	PHESANT Transformation:NA-Notes:NA-Variable type:categorical-Phenotype ID:M13_LOWBACKPAIN
categorical.M13_MENISCUSDERANGEMENTS.txt	1710174270056F5forCTG.txt.gz	0.2655	0.05288	5.021	5.14E-07	0.013	0.002409	0.995	0.01145	-0.01003	0.00827	Meniscus derangement	TRUE	Skeletal	Physical health	Deranged meniscus	361194	10831	350363	UK Biobank	https://docs.google.com/spreadsheets/d/1kvPoupSzsSFBNSztMzl04xMoSC3Kcx3CrjVf4yBmESU/edit?ts=5b5f17db#gid=227859291	PHESANT Transformation:NA-Notes:NA-Variable type:categorical-Phenotype ID:M13_MENISCUSDERANGEMENTS
categorical.M13_MUSCLE.txt	1710174270056F5forCTG.txt.gz	0.1304	0.1047	1.246	0.2128	0.003542	0.001499	0.979	0.009489	0.003597	0.008205	Disorders of muscles	FALSE				361194	487	360707	UK Biobank	https://docs.google.com/spreadsheets/d/1kvPoupSzsSFBNSztMzl04xMoSC3Kcx3CrjVf4yBmESU/edit?ts=5b5f17db#gid=227859291	PHESANT Transformation:NA-Notes:NA-Variable type:categorical-Phenotype ID:M13_MUSCLE
categorical.M13_OLECRANONBURSITIS.txt	1710174270056F5forCTG.txt.gz	0.1385	0.1311	1.057	0.2907	0.002186	0.00139	0.994	0.009585	-0.005806	0.008414	Olecranon bursitis	FALSE				361194	285	360909	UK Biobank	https://docs.google.com/spreadsheets/d/1kvPoupSzsSFBNSztMzl04xMoSC3Kcx3CrjVf4yBmESU/edit?ts=5b5f17db#gid=227859291	PHESANT Transformation:NA-Notes:NA-Variable type:categorical-Phenotype ID:M13_OLECRANONBURSITIS
categorical.M13_OSTEOPOROSIS.txt	1710174270056F5forCTG.txt.gz	0.06228	0.08124	0.7666	0.4433	0.005284	0.001669	0.995	0.01	0.002761	0.007874	Osteoporosis	FALSE				361194	933	360261	UK Biobank	https://docs.google.com/spreadsheets/d/1kvPoupSzsSFBNSztMzl04xMoSC3Kcx3CrjVf4yBmESU/edit?ts=5b5f17db#gid=227859291	PHESANT Transformation:NA-Notes:NA-Variable type:categorical-Phenotype ID:M13_OSTEOPOROSIS
categorical.M13_OTHERJOINT.txt	1710174270056F5forCTG.txt.gz	0.3778	0.04566	8.274	1.29E-16	0.01964	0.002502	1.003	0.01242	-0.01521	0.008831	#Other joint disorders	FALSE	Skeletal			361194	27347	333847	UK Biobank	https://docs.google.com/spreadsheets/d/1kvPoupSzsSFBNSztMzl04xMoSC3Kcx3CrjVf4yBmESU/edit?ts=5b5f17db#gid=227859291	PHESANT Transformation:NA-Notes:NA-Variable type:categorical-Phenotype ID:M13_OTHERJOINT
categorical.M13_POLYARTHROPATHIES.txt	1710174270056F5forCTG.txt.gz	0.3659	0.108	3.387	0.000707	0.004109	0.001716	0.999	0.0103	-0.00142	0.008223	#Polyarthropathies	FALSE				361194	3275	357919	UK Biobank	https://docs.google.com/spreadsheets/d/1kvPoupSzsSFBNSztMzl04xMoSC3Kcx3CrjVf4yBmESU/edit?ts=5b5f17db#gid=227859291	PHESANT Transformation:NA-Notes:NA-Variable type:categorical-Phenotype ID:M13_POLYARTHROPATHIES
categorical.M13_RHEUMA.txt	1710174270056F5forCTG.txt.gz	0.1977	0.1329	1.488	0.1368	0.002451	0.001615	1.006	0.009763	0.004897	0.007407	Rheumatoid arthritis	FALSE				361194	1605	359589	UK Biobank	https://docs.google.com/spreadsheets/d/1kvPoupSzsSFBNSztMzl04xMoSC3Kcx3CrjVf4yBmESU/edit?ts=5b5f17db#gid=227859291	PHESANT Transformation:NA-Notes:NA-Variable type:categorical-Phenotype ID:M13_RHEUMA
categorical.M13_ROTATORCUFF.txt	1710174270056F5forCTG.txt.gz	0.2526	0.0675	3.743	0.000182	0.006612	0.001538	0.997	0.00908	0.0008711	0.007117	Rotator cuff syndrome	FALSE				361194	2285	358909	UK Biobank	https://docs.google.com/spreadsheets/d/1kvPoupSzsSFBNSztMzl04xMoSC3Kcx3CrjVf4yBmESU/edit?ts=5b5f17db#gid=227859291	PHESANT Transformation:NA-Notes:NA-Variable type:categorical-Phenotype ID:M13_ROTATORCUFF
categorical.M13_SHOULDER.txt	1710174270056F5forCTG.txt.gz	0.2954	0.0565	5.229	1.70E-07	0.009463	0.001705	0.999	0.01049	0.008629	0.006725	Shoulder lesions	TRUE	Skeletal	Physical health	Shoulder lesions	361194	7243	353951	UK Biobank	https://docs.google.com/spreadsheets/d/1kvPoupSzsSFBNSztMzl04xMoSC3Kcx3CrjVf4yBmESU/edit?ts=5b5f17db#gid=227859291	PHESANT Transformation:NA-Notes:NA-Variable type:categorical-Phenotype ID:M13_SHOULDER
categorical.M13_SOFTTISSUENAS.txt	1710174270056F5forCTG.txt.gz	0.2137	0.09066	2.358	0.0184	0.00442	0.002064	0.999	0.01167	-0.01154	0.007643	Other specified/unspecified soft tissue disorders	FALSE				361194	2930	358264	UK Biobank	https://docs.google.com/spreadsheets/d/1kvPoupSzsSFBNSztMzl04xMoSC3Kcx3CrjVf4yBmESU/edit?ts=5b5f17db#gid=227859291	PHESANT Transformation:NA-Notes:NA-Variable type:categorical-Phenotype ID:M13_SOFTTISSUENAS
categorical.M13_SOFTTISSUEOTH.txt	1710174270056F5forCTG.txt.gz	0.4671	0.1203	3.882	0.000104	0.004134	0.00162	1.011	0.01022	-0.01101	0.007664	Other soft tissue disorders, not elsewhere classified	FALSE				361194	7233	353961	UK Biobank	https://docs.google.com/spreadsheets/d/1kvPoupSzsSFBNSztMzl04xMoSC3Kcx3CrjVf4yBmESU/edit?ts=5b5f17db#gid=227859291	PHESANT Transformation:NA-Notes:NA-Variable type:categorical-Phenotype ID:M13_SOFTTISSUEOTH
categorical.M13_SPINSTENOSIS.txt	1710174270056F5forCTG.txt.gz	0.2996	0.08938	3.352	0.000802	0.005498	0.001591	1.003	0.00922	-0.0103	0.008317	Spinal stenosis	FALSE				361194	1910	359284	UK Biobank	https://docs.google.com/spreadsheets/d/1kvPoupSzsSFBNSztMzl04xMoSC3Kcx3CrjVf4yBmESU/edit?ts=5b5f17db#gid=227859291	PHESANT Transformation:NA-Notes:NA-Variable type:categorical-Phenotype ID:M13_SPINSTENOSIS

categorical.M13_SPONDYLOPATHY.txt	1710174270056F5forCTG.txt.gz	0.3732	0.08031	4.646	3.38E-06	0.006746	0.001755	1.005	0.01013	-0.000519	0.008672	#Spondylopathies	FALSE	Skeletal			361194	4393	356801	UK Biobank	https://docs.google.com/spreadsheets/d/1kvPoupSzsSFBNSztMzl04xMoSC3Kcx3CrjVf4yBmESU/edit?ts=5b5f17db#gid=227859291	PHESANT Transformation:NA-Notes:NA-Variable type:categorical-Phenotype ID:M13_SPONDYLOPATHY
categorical.M13_SYNOTEND.txt	1710174270056F5forCTG.txt.gz	0.2243	0.09861	2.275	0.02291	0.004465	0.001464	1.003	0.009091	0.01262	0.008206	Disorders of synovium and tendon	FALSE				361194	5894	355300	UK Biobank	https://docs.google.com/spreadsheets/d/1kvPoupSzsSFBNSztMzl04xMoSC3Kcx3CrjVf4yBmESU/edit?ts=5b5f17db#gid=227859291	PHESANT Transformation:NA-Notes:NA-Variable type:categorical-Phenotype ID:M13_SYNOTEND
categorical.M13_TRIGGERFINGER.txt	1710174270056F5forCTG.txt.gz	0.08279	0.09755	0.8487	0.3961	0.004298	0.001715	1.007	0.009731	0.0147	0.008765	Trigger finger	FALSE				361194	1999	359195	UK Biobank	https://docs.google.com/spreadsheets/d/1kvPoupSzsSFBNSztMzl04xMoSC3Kcx3CrjVf4yBmESU/edit?ts=5b5f17db#gid=227859291	PHESANT Transformation:NA-Notes:NA-Variable type:categorical-Phenotype ID:M13_TRIGGERFINGER
categorical.M13.txt	1710174270056F5forCTG.txt.gz	0.4309	0.163	2.643	0.008221	0.002641	0.001532	0.994	0.009491	-0.009581	0.008216	Diagnoses - main ICD10: M13 Other arthritis	FALSE				361194	1110	360084	UK Biobank	https://docs.google.com/spreadsheets/d/1kvPoupSzsSFBNSztMzl04xMoSC3Kcx3CrjVf4yBmESU/edit?ts=5b5f17db#gid=227859291	PHESANT Transformation:NA-Notes:NA-Variable type:categorical-Phenotype ID:M13
categorical.M15.txt	1710174270056F5forCTG.txt.gz	0.2355	0.1221	1.929	0.05376	0.00287	0.001629	1.002	0.009349	0.0005074	0.00732	Diagnoses - main ICD10: M15 Polyarthrosis	FALSE				361194	1264	359930	UK Biobank	https://docs.google.com/spreadsheets/d/1kvPoupSzsSFBNSztMzl04xMoSC3Kcx3CrjVf4yBmESU/edit?ts=5b5f17db#gid=227859291	PHESANT Transformation:NA-Notes:NA-Variable type:categorical-Phenotype ID:M15
categorical.M16.txt	1710174270056F5forCTG.txt.gz	0.1287	0.04964	2.592	0.009555	0.01839	0.002343	1.012	0.01251	-0.00792	0.008438	Diagnoses - main ICD10: M16 Coxarthrosis [arthrosis of hip]	FALSE				361194	9136	352058	UK Biobank	https://docs.google.com/spreadsheets/d/1kvPoupSzsSFBNSztMzl04xMoSC3Kcx3CrjVf4yBmESU/edit?ts=5b5f17db#gid=227859291	PHESANT Transformation:NA-Notes:NA-Variable type:categorical-Phenotype ID:M16
categorical.M17.txt	1710174270056F5forCTG.txt.gz	0.2735	0.04554	6.007	1.90E-09	0.02257	0.00232	1.009	0.01198	0.001856	0.009569	Diagnoses - main ICD10: M17 Gonarthrosis [arthrosis of knee]	FALSE	Skeletal			361194	11497	349697	UK Biobank	https://docs.google.com/spreadsheets/d/1kvPoupSzsSFBNSztMzl04xMoSC3Kcx3CrjVf4yBmESU/edit?ts=5b5f17db#gid=227859291	PHESANT Transformation:NA-Notes:NA-Variable type:categorical-Phenotype ID:M17
categorical.M19.txt	1710174270056F5forCTG.txt.gz	0.2073	0.1153	1.797	0.07226	0.003971	0.001708	1.015	0.01047	0.01401	0.008053	Diagnoses - main ICD10: M19 Other arthrosis	FALSE				361194	4165	357029	UK Biobank	https://docs.google.com/spreadsheets/d/1kvPoupSzsSFBNSztMzl04xMoSC3Kcx3CrjVf4yBmESU/edit?ts=5b5f17db#gid=227859291	PHESANT Transformation:NA-Notes:NA-Variable type:categorical-Phenotype ID:M19
categorical.M20.txt	1710174270056F5forCTG.txt.gz	0.1336	0.05359	2.493	0.01265	0.01437	0.00177	0.989	0.01061	-0.001244	0.008288	Diagnoses - main ICD10: M20 Acquired deformities of fingers and toes	FALSE				361194	7773	353421	UK Biobank	https://docs.google.com/spreadsheets/d/1kvPoupSzsSFBNSztMzl04xMoSC3Kcx3CrjVf4yBmESU/edit?ts=5b5f17db#gid=227859291	PHESANT Transformation:NA-Notes:NA-Variable type:categorical-Phenotype ID:M20
categorical.M23.txt	1710174270056F5forCTG.txt.gz	0.266	0.05243	5.073	3.91E-07	0.01307	0.002476	0.998	0.01192	-0.009667	0.008314	Diagnoses - main ICD10: M23 Internal derangement of knee	FALSE	Skeletal			361194	11831	349363	UK Biobank	https://docs.google.com/spreadsheets/d/1kvPoupSzsSFBNSztMzl04xMoSC3Kcx3CrjVf4yBmESU/edit?ts=5b5f17db#gid=227859291	PHESANT Transformation:NA-Notes:NA-Variable type:categorical-Phenotype ID:M23
categorical.M25.txt	1710174270056F5forCTG.txt.gz	0.7305	0.1713	4.265	2.00E-05	0.00386	0.00168	1.008	0.009696	-0.01654	0.007708	Diagnoses - main ICD10: M25 Other joint disorders, not elsewhere classified	TRUE	Skeletal	Physical health	Other joint disorders	361194	7218	353976	UK Biobank	https://docs.google.com/spreadsheets/d/1kvPoupSzsSFBNSztMzl04xMoSC3Kcx3CrjVf4yBmESU/edit?ts=5b5f17db#gid=227859291	PHESANT Transformation:NA-Notes:NA-Variable type:categorical-Phenotype ID:M25
categorical.M47.txt	1710174270056F5forCTG.txt.gz	0.4238	0.1526	2.777	0.005493	0.002761	0.001692	1.005	0.01031	0.008306	0.008082	Diagnoses - main ICD10: M47 Spondylosis	FALSE				361194	2004	359190	UK Biobank	https://docs.google.com/spreadsheets/d/1kvPoupSzsSFBNSztMzl04xMoSC3Kcx3CrjVf4yBmESU/edit?ts=5b5f17db#gid=227859291	PHESANT Transformation:NA-Notes:NA-Variable type:categorical-Phenotype ID:M47
categorical.M48.txt	1710174270056F5forCTG.txt.gz	0.3085	0.09558	3.227	0.001249	0.004958	0.001593	1.003	0.009462	-0.007406	0.008321	Diagnoses - main ICD10: M48 Other spondylopathies	FALSE				361194	1890	359304	UK Biobank	https://docs.google.com/spreadsheets/d/1kvPoupSzsSFBNSztMzl04xMoSC3Kcx3CrjVf4yBmESU/edit?ts=5b5f17db#gid=227859291	PHESANT Transformation:NA-Notes:NA-Variable type:categorical-Phenotype ID:M48
categorical.M51.txt	1710174270056F5forCTG.txt.gz	0.3112	0.09015	3.452	0.000557	0.005541	0.001625	1.018	0.009864	0.01689	0.007873	Diagnoses - main ICD10: M51 Other intervertebral disk disorders	FALSE				361194	4690	356504	UK Biobank	https://docs.google.com/spreadsheets/d/1kvPoupSzsSFBNSztMzl04xMoSC3Kcx3CrjVf4yBmESU/edit?ts=5b5f17db#gid=227859291	PHESANT Transformation:NA-Notes:NA-Variable type:categorical-Phenotype ID:M51
categorical.M53.txt	1710174270056F5forCTG.txt.gz	0.001773	0.1119	0.01584	0.9874	0.002634	0.001499	0.985	0.009881	0.008261	0.007116	Diagnoses - main ICD10: M53 Other dorsopathies, not elsewhere classified	FALSE				361194	342	360852	UK Biobank	https://docs.google.com/spreadsheets/d/1kvPoupSzsSFBNSztMzl04xMoSC3Kcx3CrjVf4yBmESU/edit?ts=5b5f17db#gid=227859291	PHESANT Transformation:NA-Notes:NA-Variable type:categorical-Phenotype ID:M53
categorical.M54.txt	1710174270056F5forCTG.txt.gz	0.39	0.05437	7.173	7.33E-13	0.01454	0.001959	0.992	0.01109	0.009998	0.008606	Diagnoses - main ICD10: M54 Dorsalgia	FALSE	Pain			361194	8361	352833	UK Biobank	https://docs.google.com/spreadsheets/d/1kvPoupSzsSFBNSztMzl04xMoSC3Kcx3CrjVf4yBmESU/edit?ts=5b5f17db#gid=227859291	PHESANT Transformation:NA-Notes:NA-Variable type:categorical-Phenotype ID:M54
categorical.M65.txt	1710174270056F5forCTG.txt.gz	0.08062	0.102	0.7906	0.4292	0.004158	0.001612	1.006	0.008888	0.01531	0.008645	Diagnoses - main ICD10: M65 Synovitis and tenosynovitis	FALSE				361194	2812	358382	UK Biobank	https://docs.google.com/spreadsheets/d/1kvPoupSzsSFBNSztMzl04xMoSC3Kcx3CrjVf4yBmESU/edit?ts=5b5f17db#gid=227859291	PHESANT Transformation:NA-Notes:NA-Variable type:categorical-Phenotype ID:M65
categorical.M67.txt	1710174270056F5forCTG.txt.gz	0.2836	0.1089	2.603	0.009237	0.003752	0.001564	0.993	0.01006	-0.000109	0.007331	Diagnoses - main ICD10: M67 Other disorders of synovium and tendon	FALSE				361194	2613	358581	UK Biobank	https://docs.google.com/spreadsheets/d/1kvPoupSzsSFBNSztMzl04xMoSC3Kcx3CrjVf4yBmESU/edit?ts=5b5f17db#gid=227859291	PHESANT Transformation:NA-Notes:NA-Variable type:categorical-Phenotype ID:M67
categorical.M72.txt	1710174270056F5forCTG.txt.gz	0.111	0.061	1.82	0.06875	0.01405	0.003324	1.03	0.0213	-0.004183	0.00842	Diagnoses - main ICD10: M72 Fibroblastic disorders	FALSE				361194	3193	358001	UK Biobank	https://docs.google.com/spreadsheets/d/1kvPoupSzsSFBNSztMzl04xMoSC3Kcx3CrjVf4yBmESU/edit?ts=5b5f17db#gid=227859291	PHESANT Transformation:NA-Notes:NA-Variable type:categorical-Phenotype ID:M72
categorical.M75.txt	1710174270056F5forCTG.txt.gz	0.2965	0.05654	5.243	1.58E-07	0.009442	0.001705	0.999	0.01048	0.008465	0.006722	Diagnoses - main ICD10: M75 Shoulder lesions	FALSE	Skeletal			361194	7040	354154	UK Biobank	https://docs.google.com/spreadsheets/d/1kvPoupSzsSFBNSztMzl04xMoSC3Kcx3CrjVf4yBmESU/edit?ts=5b5f17db#gid=227859291	PHESANT Transformation:NA-Notes:NA-Variable type:categorical-Phenotype ID:M75
categorical.M79.txt	1710174270056F5forCTG.txt.gz	0.4671	0.1203	3.882	0.000104	0.004134	0.00162	1.011	0.01022	-0.01101	0.007664	Diagnoses - main ICD10: M79 Other soft tissue disorders, not elsewhere classified	FALSE				361194	6946	354248	UK Biobank	https://docs.google.com/spreadsheets/d/1kvPoupSzsSFBNSztMzl04xMoSC3Kcx3CrjVf4yBmESU/edit?ts=5b5f17db#gid=227859291	PHESANT Transformation:NA-Notes:NA-Variable type:categorical-Phenotype ID:M79
categorical.M81.txt	1710174270056F5forCTG.txt.gz	0.09727	0.1142	0.8514	0.3946	0.002898	0.001561	1.001	0.009319	-0.004576	0.007923	Diagnoses - main ICD10: M81 Osteoporosis without pathological fracture	FALSE				361194	737	360457	UK Biobank	https://docs.google.com/spreadsheets/d/1kvPoupSzsSFBNSztMzl04xMoSC3Kcx3CrjVf4yBmESU/edit?ts=5b5f17db#gid=227859291	PHESANT Transformation:NA-Notes:NA-Variable type:categorical-Phenotype ID:M81
categorical.N20.txt	1710174270056F5forCTG.txt.gz	0.1058	0.06112	1.731	0.08342	0.01116	0.00174	1.014	0.01069	0.007714	0.008072	Diagnoses - main ICD10: N20 Calculus of kidney and ureter	FALSE				361194	3540	357654	UK Biobank	https://docs.google.com/spreadsheets/d/1kvPoupSzsSFBNSztMzl04xMoSC3Kcx3CrjVf4yBmESU/edit?ts=5b5f17db#gid=227859291	PHESANT Transformation:NA-Notes:NA-Variable type:categorical-Phenotype ID:N20
categorical.N32.txt	1710174270056F5forCTG.txt.gz	0.08057	0.1222	0.6593	0.5097	0.002367	0.001556	1.012	0.009656	0.006384	0.007489	Diagnoses - main ICD10: N32 Other disorders of bladder	FALSE				361194	4238	356956	UK Biobank	https://docs.google.com/spreadsheets/d/1kvPoupSzsSFBNSztMzl04xMoSC3Kcx3CrjVf4yBmESU/edit?ts=5b5f17db#gid=227859291	PHESANT Transformation:NA-Notes:NA-Variable type:categorical-Phenotype ID:N32

categorical.N39.txt	1710174270056F5forCTG.txt.gz	0.4407	0.07168	6.148	7.84E-10	0.009073	0.00179	0.994	0.009816	-0.01038	0.008877	Diagnoses - main ICD10: N39 Other disorders of urinary system	FALSE	Other (physical health)			361194	10551	350643	UK Biobank	https://docs.google.com/spreadsheets/d/1kvPoupSzsSFBNSztMzl04xMoSC3Kcx3CrjVf4yBmESU/edit?ts=5b5f17db#gid=227859291	PHESANT Transformation:NA-Notes:NA-Variable type:categorical-Phenotype ID:N39
categorical.N40.txt	1710174270056F5forCTG.txt.gz	0.07616	0.07357	1.035	0.3006	0.006208	0.0019	1.027	0.01045	-0.001224	0.008086	Diagnoses - main ICD10: N40 Hyperplasia of prostate	FALSE				361194	5109	356085	UK Biobank	https://docs.google.com/spreadsheets/d/1kvPoupSzsSFBNSztMzl04xMoSC3Kcx3CrjVf4yBmESU/edit?ts=5b5f17db#gid=227859291	PHESANT Transformation:NA-Notes:NA-Variable type:categorical-Phenotype ID:N40
categorical.N43.txt	1710174270056F5forCTG.txt.gz	-0.1008	0.07496	-1.344	0.1788	0.006575	0.001623	0.995	0.00946	0.02421	0.007887	Diagnoses - main ICD10: N43 Hydrocele and spermatocele	FALSE				361194	884	360310	UK Biobank	https://docs.google.com/spreadsheets/d/1kvPoupSzsSFBNSztMzl04xMoSC3Kcx3CrjVf4yBmESU/edit?ts=5b5f17db#gid=227859291	PHESANT Transformation:NA-Notes:NA-Variable type:categorical-Phenotype ID:N43
categorical.N47.txt	1710174270056F5forCTG.txt.gz	0.1598	0.1211	1.32	0.1869	0.003229	0.001645	0.996	0.009824	0.000181	0.008276	Diagnoses - main ICD10: N47 Redundant prepuce, phimosis and paraphimosis	FALSE				361194	1543	359651	UK Biobank	https://docs.google.com/spreadsheets/d/1kvPoupSzsSFBNSztMzl04xMoSC3Kcx3CrjVf4yBmESU/edit?ts=5b5f17db#gid=227859291	PHESANT Transformation:NA-Notes:NA-Variable type:categorical-Phenotype ID:N47
categorical.N60.txt	1710174270056F5forCTG.txt.gz	-0.04087	0.1251	-0.3267	0.7439	0.002245	0.001413	0.994	0.009156	0.008513	0.007248	Diagnoses - main ICD10: N60 Benign mammary dysplasia	FALSE				361194	1157	360037	UK Biobank	https://docs.google.com/spreadsheets/d/1kvPoupSzsSFBNSztMzl04xMoSC3Kcx3CrjVf4yBmESU/edit?ts=5b5f17db#gid=227859291	PHESANT Transformation:NA-Notes:NA-Variable type:categorical-Phenotype ID:N60
categorical.N80.txt	1710174270056F5forCTG.txt.gz	0.02145	0.08341	0.2571	0.7971	0.006102	0.001496	0.984	0.008561	0.01246	0.007902	Diagnoses - main ICD10: N80 Endometriosis	FALSE				361194	1496	359698	UK Biobank	https://docs.google.com/spreadsheets/d/1kvPoupSzsSFBNSztMzl04xMoSC3Kcx3CrjVf4yBmESU/edit?ts=5b5f17db#gid=227859291	PHESANT Transformation:NA-Notes:NA-Variable type:categorical-Phenotype ID:N80
categorical.N81.txt	1710174270056F5forCTG.txt.gz	0.2046	0.06429	3.183	0.001456	0.01144	0.001824	1.038	0.009971	0.001304	0.008782	Diagnoses - main ICD10: N81 Female genital prolapse	FALSE				361194	7511	353683	UK Biobank	https://docs.google.com/spreadsheets/d/1kvPoupSzsSFBNSztMzl04xMoSC3Kcx3CrjVf4yBmESU/edit?ts=5b5f17db#gid=227859291	PHESANT Transformation:NA-Notes:NA-Variable type:categorical-Phenotype ID:N81
categorical.N84.txt	1710174270056F5forCTG.txt.gz	-0.01888	0.07769	-0.243	0.808	0.006921	0.001799	0.973	0.009798	0.01376	0.007562	Diagnoses - main ICD10: N84 Polyp of female genital tract	FALSE				361194	6986	354208	UK Biobank	https://docs.google.com/spreadsheets/d/1kvPoupSzsSFBNSztMzl04xMoSC3Kcx3CrjVf4yBmESU/edit?ts=5b5f17db#gid=227859291	PHESANT Transformation:NA-Notes:NA-Variable type:categorical-Phenotype ID:N84
categorical.N92.txt	1710174270056F5forCTG.txt.gz	0.3748	0.07334	5.11	3.22E-07	0.007544	0.001847	1.002	0.01071	-0.003177	0.00809	Diagnoses - main ICD10: N92 Excessive, frequent and irregular menstruation	TRUE	Reproductive		Excessive, frequent or irregular menstruation	361194	8475	352719	UK Biobank	https://docs.google.com/spreadsheets/d/1kvPoupSzsSFBNSztMzl04xMoSC3Kcx3CrjVf4yBmESU/edit?ts=5b5f17db#gid=227859291	PHESANT Transformation:NA-Notes:NA-Variable type:categorical-Phenotype ID:N92
categorical.OTHER_ILD_CVD_COMORB.txt	1710174270056F5forCTG.txt.gz	0.1302	0.1004	1.297	0.1947	0.003377	0.001826	1.018	0.01182	0.001826	0.008291	Other ILD-related CVD-co-morbidities	FALSE				361194	2507	358687	UK Biobank	https://docs.google.com/spreadsheets/d/1kvPoupSzsSFBNSztMzl04xMoSC3Kcx3CrjVf4yBmESU/edit?ts=5b5f17db#gid=227859291	PHESANT Transformation:NA-Notes:NA-Variable type:categorical-Phenotype ID:OTHER_ILD_CVD_COMORB
categorical.PNEUMONIA.txt	1710174270056F5forCTG.txt.gz	0.5908	0.2436	2.425	0.0153	0.00194	0.001412	1.007	0.008891	0.004398	0.007437	Pneumonias (Asthma/COPD co-morbidities)	FALSE				361194	5900	355294	UK Biobank	https://docs.google.com/spreadsheets/d/1kvPoupSzsSFBNSztMzl04xMoSC3Kcx3CrjVf4yBmESU/edit?ts=5b5f17db#gid=227859291	PHESANT Transformation:NA-Notes:NA-Variable type:categorical-Phenotype ID:PNEUMONIA
categorical.PRIM_KNEEARTHROSIS.txt	1710174270056F5forCTG.txt.gz	0.2958	0.1217	2.43	0.01511	0.003088	0.001379	1.002	0.008707	0.005037	0.008391	Primary gonarthrosis, bilateral	FALSE				361194	849	360345	UK Biobank	https://docs.google.com/spreadsheets/d/1kvPoupSzsSFBNSztMzl04xMoSC3Kcx3CrjVf4yBmESU/edit?ts=5b5f17db#gid=227859291	PHESANT Transformation:NA-Notes:NA-Variable type:categorical-Phenotype ID:PRIM_KNEEARTHROSIS
categorical.PULM_MEDICATIO_COMORB.txt	1710174270056F5forCTG.txt.gz	0.1661	0.05883	2.824	0.00475	0.01258	0.001892	1.013	0.01137	-0.01229	0.009214	Medication related adverse effects (Asthma/COPD)	FALSE				361194	21706	339488	UK Biobank	https://docs.google.com/spreadsheets/d/1kvPoupSzsSFBNSztMzl04xMoSC3Kcx3CrjVf4yBmESU/edit?ts=5b5f17db#gid=227859291	PHESANT Transformation:NA-Notes:NA-Variable type:categorical-Phenotype ID:PULM_MEDICATIO_COMORB
categorical.PULMONARYDG.txt	1710174270056F5forCTG.txt.gz	0.4303	0.06172	6.972	3.12E-12	0.0114	0.001733	1.011	0.01044	0.003626	0.008563	Other pulmonary diagnosis	FALSE	Pulmonary			361194	25381	335813	UK Biobank	https://docs.google.com/spreadsheets/d/1kvPoupSzsSFBNSztMzl04xMoSC3Kcx3CrjVf4yBmESU/edit?ts=5b5f17db#gid=227859291	PHESANT Transformation:NA-Notes:NA-Variable type:categorical-Phenotype ID:PULMONARYDG
categorical.R04.txt	1710174270056F5forCTG.txt.gz	0.3859	0.1516	2.545	0.01094	0.002904	0.001602	1.012	0.009384	-0.000924	0.008424	Diagnoses - main ICD10: R04 Haemorrhage from respiratory passages	FALSE				361194	2836	358358	UK Biobank	https://docs.google.com/spreadsheets/d/1kvPoupSzsSFBNSztMzl04xMoSC3Kcx3CrjVf4yBmESU/edit?ts=5b5f17db#gid=227859291	PHESANT Transformation:NA-Notes:NA-Variable type:categorical-Phenotype ID:R04
categorical.R07.txt	1710174270056F5forCTG.txt.gz	0.5322	0.04742	11.22	3.18E-29	0.02048	0.002122	1.008	0.01116	-0.01378	0.008479	Diagnoses - main ICD10: R07 Pain in throat and chest	FALSE	Pain			361194	24530	336664	UK Biobank	https://docs.google.com/spreadsheets/d/1kvPoupSzsSFBNSztMzl04xMoSC3Kcx3CrjVf4yBmESU/edit?ts=5b5f17db#gid=227859291	PHESANT Transformation:NA-Notes:NA-Variable type:categorical-Phenotype ID:R07
categorical.R10.txt	1710174270056F5forCTG.txt.gz	0.5032	0.06502	7.739	1.00E-14	0.01104	0.00173	1.018	0.009949	-0.00566	0.008999	Diagnoses - main ICD10: R10 Abdominal and pelvic pain	FALSE	Pain			361194	20240	340954	UK Biobank	https://docs.google.com/spreadsheets/d/1kvPoupSzsSFBNSztMzl04xMoSC3Kcx3CrjVf4yBmESU/edit?ts=5b5f17db#gid=227859291	PHESANT Transformation:NA-Notes:NA-Variable type:categorical-Phenotype ID:R10
categorical.R14.txt	1710174270056F5forCTG.txt.gz	0.01158	0.08717	0.1329	0.8943	0.005042	0.001531	0.972	0.009836	0.008892	0.008568	Diagnoses - main ICD10: R14 Flatulence and related conditions	FALSE				361194	331	360863	UK Biobank	https://docs.google.com/spreadsheets/d/1kvPoupSzsSFBNSztMzl04xMoSC3Kcx3CrjVf4yBmESU/edit?ts=5b5f17db#gid=227859291	PHESANT Transformation:NA-Notes:NA-Variable type:categorical-Phenotype ID:R14
categorical.R19.txt	1710174270056F5forCTG.txt.gz	0.3787	0.1408	2.69	0.007151	0.003237	0.001575	1.005	0.009721	-0.008993	0.008184	Diagnoses - main ICD10: R19 Other symptoms and signs involving the digestive system and abdomen	FALSE				361194	8796	352398	UK Biobank	https://docs.google.com/spreadsheets/d/1kvPoupSzsSFBNSztMzl04xMoSC3Kcx3CrjVf4yBmESU/edit?ts=5b5f17db#gid=227859291	PHESANT Transformation:NA-Notes:NA-Variable type:categorical-Phenotype ID:R19
categorical.R31.txt	1710174270056F5forCTG.txt.gz	0.3322	0.09232	3.598	0.000321	0.005118	0.001671	1.013	0.009659	0.001691	0.007444	Diagnoses - main ICD10: R31 Unspecified haematuria	FALSE				361194	11283	349911	UK Biobank	https://docs.google.com/spreadsheets/d/1kvPoupSzsSFBNSztMzl04xMoSC3Kcx3CrjVf4yBmESU/edit?ts=5b5f17db#gid=227859291	PHESANT Transformation:NA-Notes:NA-Variable type:categorical-Phenotype ID:R31
categorical.R32.txt	1710174270056F5forCTG.txt.gz	0.3189	0.1673	1.907	0.05654	0.001897	0.001543	0.999	0.009767	0.005655	0.0077	Diagnoses - main ICD10: R32 Unspecified urinary incontinence	FALSE				361194	871	360323	UK Biobank	https://docs.google.com/spreadsheets/d/1kvPoupSzsSFBNSztMzl04xMoSC3Kcx3CrjVf4yBmESU/edit?ts=5b5f17db#gid=227859291	PHESANT Transformation:NA-Notes:NA-Variable type:categorical-Phenotype ID:R32
categorical.R35.txt	1710174270056F5forCTG.txt.gz	0.1952	0.1188	1.642	0.1005	0.003497	0.001762	0.993	0.01002	-0.008608	0.007472	Diagnoses - main ICD10: R35 Polyuria	FALSE				361194	1773	359421	UK Biobank	https://docs.google.com/spreadsheets/d/1kvPoupSzsSFBNSztMzl04xMoSC3Kcx3CrjVf4yBmESU/edit?ts=5b5f17db#gid=227859291	PHESANT Transformation:NA-Notes:NA-Variable type:categorical-Phenotype ID:R35
categorical.R51.txt	1710174270056F5forCTG.txt.gz	0.5102	0.1166	4.376	1.21E-05	0.004788	0.00145	1.004	0.009496	-0.001498	0.008009	Diagnoses - main ICD10: R51 Headache	FALSE	Pain			361194	4271	356923	UK Biobank	https://docs.google.com/spreadsheets/d/1kvPoupSzsSFBNSztMzl04xMoSC3Kcx3CrjVf4yBmESU/edit?ts=5b5f17db#gid=227859291	PHESANT Transformation:NA-Notes:NA-Variable type:categorical-Phenotype ID:R51
categorical.R53.txt	1710174270056F5forCTG.txt.gz	0.206	0.1063	1.938	0.05257	0.003877	0.001616	0.985	0.009568	-0.007419	0.008093	Diagnoses - main ICD10: R53 Malaise and fatigue	FALSE				361194	818	360376	UK Biobank	https://docs.google.com/spreadsheets/d/1kvPoupSzsSFBNSztMzl04xMoSC3Kcx3CrjVf4yBmESU/edit?ts=5b5f17db#gid=227859291	PHESANT Transformation:NA-Notes:NA-Variable type:categorical-Phenotype ID:R53
categorical.R55.txt	1710174270056F5forCTG.txt.gz	0.1911	0.06655	2.872	0.004082	0.007172	0.001499	0.996	0.009679	-0.000567	0.007405	Diagnoses - main ICD10: R55 Syncope and collapse	FALSE				361194	5183	356011	UK Biobank	https://docs.google.com/spreadsheets/d/1kvPoupSzsSFBNSztMzl04xMoSC3Kcx3CrjVf4yBmESU/edit?ts=5b5f17db#gid=227859291	PHESANT Transformation:NA-Notes:NA-Variable type:categorical-Phenotype ID:R55

categorical.R69.txt	1710174270056F5forCTG.txt.gz	0.3022	0.1261	2.397	0.01653	0.003625	0.001603	1.001	0.009075	-0.00136	0.008307	Diagnoses - main ICD10: R69 Unknown and unspecified causes of morbidity	FALSE				361194	8280	352914	UK Biobank	https://docs.google.com/spreadsheets/d/1kvPoupSzsSFBNSztMzl04xMoSC3Kcx3CrjVf4yBmESU/edit?ts=5b5f17db#gid=227859291	PHESANT Transformation:NA-Notes:NA-Variable type:categorical-Phenotype ID:R69
categorical.R79.txt	1710174270056F5forCTG.txt.gz	-0.05278	0.09393	-0.5619	0.5742	0.00402	0.001645	1.003	0.01047	0.006368	0.007264	Diagnoses - main ICD10: R79 Other abnormal findings of blood chemistry	FALSE				361194	2622	358572	UK Biobank	https://docs.google.com/spreadsheets/d/1kvPoupSzsSFBNSztMzl04xMoSC3Kcx3CrjVf4yBmESU/edit?ts=5b5f17db#gid=227859291	PHESANT Transformation:NA-Notes:NA-Variable type:categorical-Phenotype ID:R79
categorical.R91.txt	1710174270056F5forCTG.txt.gz	0.2442	0.1051	2.322	0.02022	0.003065	0.001466	0.982	0.008955	-0.001419	0.006843	Diagnoses - main ICD10: R91 Abnormal findings on diagnostic imaging of lung	FALSE				361194	1130	360064	UK Biobank	https://docs.google.com/spreadsheets/d/1kvPoupSzsSFBNSztMzl04xMoSC3Kcx3CrjVf4yBmESU/edit?ts=5b5f17db#gid=227859291	PHESANT Transformation:NA-Notes:NA-Variable type:categorical-Phenotype ID:R91
categorical.RHEU_ARTHRITIS_OTH.txt	1710174270056F5forCTG.txt.gz	0.4309	0.163	2.643	0.008221	0.002641	0.001532	0.994	0.009491	-0.009581	0.008216	Other arthritis (FG)	FALSE				361194	1212	359982	UK Biobank	https://docs.google.com/spreadsheets/d/1kvPoupSzsSFBNSztMzl04xMoSC3Kcx3CrjVf4yBmESU/edit?ts=5b5f17db#gid=227859291	PHESANT Transformation:NA-Notes:NA-Variable type:categorical-Phenotype ID:RHEU_ARTHRITIS_OTH
categorical.S01.txt	1710174270056F5forCTG.txt.gz	0.08832	0.1095	0.8063	0.42	0.002975	0.001645	0.989	0.01021	0.0005119	0.008325	Diagnoses - main ICD10: S01 Open wound of head	FALSE				361194	1694	359500	UK Biobank	https://docs.google.com/spreadsheets/d/1kvPoupSzsSFBNSztMzl04xMoSC3Kcx3CrjVf4yBmESU/edit?ts=5b5f17db#gid=227859291	PHESANT Transformation:NA-Notes:NA-Variable type:categorical-Phenotype ID:S01
categorical.S09.txt	1710174270056F5forCTG.txt.gz	0.2389	0.08204	2.912	0.003593	0.005019	0.001556	0.991	0.00835	-0.01526	0.007467	Diagnoses - main ICD10: S09 Other and unspecified injuries of head	FALSE				361194	1333	359861	UK Biobank	https://docs.google.com/spreadsheets/d/1kvPoupSzsSFBNSztMzl04xMoSC3Kcx3CrjVf4yBmESU/edit?ts=5b5f17db#gid=227859291	PHESANT Transformation:NA-Notes:NA-Variable type:categorical-Phenotype ID:S09
categorical.S42.txt	1710174270056F5forCTG.txt.gz	0.1739	0.08532	2.038	0.04152	0.004142	0.001573	0.985	0.009331	-0.002675	0.00817	Diagnoses - main ICD10: S42 Fracture of shoulder and upper arm	FALSE				361194	1791	359403	UK Biobank	https://docs.google.com/spreadsheets/d/1kvPoupSzsSFBNSztMzl04xMoSC3Kcx3CrjVf4yBmESU/edit?ts=5b5f17db#gid=227859291	PHESANT Transformation:NA-Notes:NA-Variable type:categorical-Phenotype ID:S42
categorical.S52.txt	1710174270056F5forCTG.txt.gz	-0.1647	0.0649	-2.537	0.01118	0.008334	0.001807	0.996	0.009359	0.01571	0.008341	Diagnoses - main ICD10: S52 Fracture of forearm	FALSE				361194	5080	356114	UK Biobank	https://docs.google.com/spreadsheets/d/1kvPoupSzsSFBNSztMzl04xMoSC3Kcx3CrjVf4yBmESU/edit?ts=5b5f17db#gid=227859291	PHESANT Transformation:NA-Notes:NA-Variable type:categorical-Phenotype ID:S52
categorical.S62.txt	1710174270056F5forCTG.txt.gz	0.05556	0.1255	0.4426	0.658	0.002512	0.001694	0.997	0.01021	0.01345	0.008528	Diagnoses - main ICD10: S62 Fracture at wrist and hand level	FALSE				361194	1763	359431	UK Biobank	https://docs.google.com/spreadsheets/d/1kvPoupSzsSFBNSztMzl04xMoSC3Kcx3CrjVf4yBmESU/edit?ts=5b5f17db#gid=227859291	PHESANT Transformation:NA-Notes:NA-Variable type:categorical-Phenotype ID:S62
categorical.S66.txt	1710174270056F5forCTG.txt.gz	0.2698	0.2372	1.138	0.2553	0.001248	0.00151	1.01	0.009542	5.20E-05	0.008359	Diagnoses - main ICD10: S66 Injury of muscle and tendon at wrist and hand level	FALSE				361194	789	360405	UK Biobank	https://docs.google.com/spreadsheets/d/1kvPoupSzsSFBNSztMzl04xMoSC3Kcx3CrjVf4yBmESU/edit?ts=5b5f17db#gid=227859291	PHESANT Transformation:NA-Notes:NA-Variable type:categorical-Phenotype ID:S66
categorical.S82.txt	1710174270056F5forCTG.txt.gz	0.06756	0.08908	0.7584	0.4482	0.004085	0.00152	0.999	0.00849	0.01548	0.007745	Diagnoses - main ICD10: S82 Fracture of lower leg, including ankle	FALSE				361194	4557	356637	UK Biobank	https://docs.google.com/spreadsheets/d/1kvPoupSzsSFBNSztMzl04xMoSC3Kcx3CrjVf4yBmESU/edit?ts=5b5f17db#gid=227859291	PHESANT Transformation:NA-Notes:NA-Variable type:categorical-Phenotype ID:S82
categorical.SLEEP.txt	1710174270056F5forCTG.txt.gz	0.2898	0.07436	3.897	9.72E-05	0.006958	0.001576	0.997	0.009126	0.0005625	0.007919	Sleep disorders (combined)	FALSE				361194	2951	358243	UK Biobank	https://docs.google.com/spreadsheets/d/1kvPoupSzsSFBNSztMzl04xMoSC3Kcx3CrjVf4yBmESU/edit?ts=5b5f17db#gid=227859291	PHESANT Transformation:NA-Notes:NA-Variable type:categorical-Phenotype ID:SLEEP
categorical.SPONDYLOPATHY_FG.txt	1710174270056F5forCTG.txt.gz	0.2085	0.134	1.556	0.1196	0.002495	0.001581	0.997	0.01004	-0.004996	0.008216	Spondylopathies (FG)	FALSE				361194	336	360858	UK Biobank	https://docs.google.com/spreadsheets/d/1kvPoupSzsSFBNSztMzl04xMoSC3Kcx3CrjVf4yBmESU/edit?ts=5b5f17db#gid=227859291	PHESANT Transformation:NA-Notes:NA-Variable type:categorical-Phenotype ID:SPONDYLOPATHY_FG
categorical.T39.txt	1710174270056F5forCTG.txt.gz	0.3589	0.2272	1.58	0.1141	0.001769	0.00181	1.01	0.01065	-0.004745	0.007524	Diagnoses - main ICD10: T39 Poisoning by nonopioid analgesics, antipyretics and antirheumatics	FALSE				361194	1161	360033	UK Biobank	https://docs.google.com/spreadsheets/d/1kvPoupSzsSFBNSztMzl04xMoSC3Kcx3CrjVf4yBmESU/edit?ts=5b5f17db#gid=227859291	PHESANT Transformation:NA-Notes:NA-Variable type:categorical-Phenotype ID:T39
categorical.T50.txt	1710174270056F5forCTG.txt.gz	0.1669	0.1054	1.583	0.1134	0.00348	0.001552	0.983	0.01003	0.0002172	0.008045	Diagnoses - main ICD10: T50 Poisoning by diuretics and other and unspecified drugs, medicaments and biological substances	FALSE				361194	231	360963	UK Biobank	https://docs.google.com/spreadsheets/d/1kvPoupSzsSFBNSztMzl04xMoSC3Kcx3CrjVf4yBmESU/edit?ts=5b5f17db#gid=227859291	PHESANT Transformation:NA-Notes:NA-Variable type:categorical-Phenotype ID:T50
categorical.T81.txt	1710174270056F5forCTG.txt.gz	0.4612	0.1125	4.099	4.14E-05	0.003889	0.001392	0.999	0.008337	-0.008142	0.007803	Diagnoses - main ICD10: T81 Complications of procedures, not elsewhere classified	FALSE				361194	5550	355644	UK Biobank	https://docs.google.com/spreadsheets/d/1kvPoupSzsSFBNSztMzl04xMoSC3Kcx3CrjVf4yBmESU/edit?ts=5b5f17db#gid=227859291	PHESANT Transformation:NA-Notes:NA-Variable type:categorical-Phenotype ID:T81
categorical.T84.txt	1710174270056F5forCTG.txt.gz	0.3156	0.112	2.818	0.004838	0.004084	0.00136	1.006	0.008271	0.001005	0.008104	Diagnoses - main ICD10: T84 Complications of internal orthopaedic prosthetic devices, implants and grafts	FALSE				361194	3719	357475	UK Biobank	https://docs.google.com/spreadsheets/d/1kvPoupSzsSFBNSztMzl04xMoSC3Kcx3CrjVf4yBmESU/edit?ts=5b5f17db#gid=227859291	PHESANT Transformation:NA-Notes:NA-Variable type:categorical-Phenotype ID:T84
categorical.T86.txt	1710174270056F5forCTG.txt.gz	0.1095	0.09895	1.107	0.2684	0.003827	0.001543	0.985	0.009664	-0.01393	0.009069	Diagnoses - main ICD10: T86 Failure and rejection of transplanted organs and tissues	FALSE				361194	163	361031	UK Biobank	https://docs.google.com/spreadsheets/d/1kvPoupSzsSFBNSztMzl04xMoSC3Kcx3CrjVf4yBmESU/edit?ts=5b5f17db#gid=227859291	PHESANT Transformation:NA-Notes:NA-Variable type:categorical-Phenotype ID:T86
categorical.ULCERNAS.txt	1710174270056F5forCTG.txt.gz	-0.01551	0.07174	-0.2162	0.8288	0.006003	0.001714	1.016	0.01039	0.01109	0.007579	Ulcerative colitis, NAS	FALSE				361194	1903	359291	UK Biobank	https://docs.google.com/spreadsheets/d/1kvPoupSzsSFBNSztMzl04xMoSC3Kcx3CrjVf4yBmESU/edit?ts=5b5f17db#gid=227859291	PHESANT Transformation:NA-Notes:NA-Variable type:categorical-Phenotype ID:ULCERNAS
categorical.V_MENTAL_BEHAV.txt	1710174270056F5forCTG.txt.gz	0.1541	0.07876	1.956	0.05043	0.006476	0.001658	1.003	0.009952	0.004199	0.008433	Mental and behavioural disorders	FALSE				361194	4302	356892	UK Biobank	https://docs.google.com/spreadsheets/d/1kvPoupSzsSFBNSztMzl04xMoSC3Kcx3CrjVf4yBmESU/edit?ts=5b5f17db#gid=227859291	PHESANT Transformation:NA-Notes:NA-Variable type:categorical-Phenotype ID:V_MENTAL_BEHAV
categorical.VI_NERVOUS.txt	1710174270056F5forCTG.txt.gz	0.4317	0.0554	7.793	6.56E-15	0.01303	0.00163	1.028	0.008952	-0.001037	0.007995	Diseases of the nervous system	TRUE	Other (physical health)	Physical health	Any disease of the nervous system	361194	21323	339871	UK Biobank	https://docs.google.com/spreadsheets/d/1kvPoupSzsSFBNSztMzl04xMoSC3Kcx3CrjVf4yBmESU/edit?ts=5b5f17db#gid=227859291	PHESANT Transformation:NA-Notes:NA-Variable type:categorical-Phenotype ID:VI_NERVOUS

categorical.VII_EYE_ADNEXA.txt	1710174270056F5forCTG.txt.gz	0.1535	0.05605	2.738	0.006176	0.01293	0.001827	1.008	0.01086	-0.004649	0.008476	Diseases of the eye and adnexa	FALSE				361194	29878	331316	UK Biobank	https://docs.google.com/spreadsheets/d/1kvPoupSzsSFBNSztMzl04xMoSC3Kcx3CrjVf4yBmESU/edit?ts=5b5f17db#gid=227859291	PHESANT Transformation:NA-Notes:NA-Variable type:categorical-Phenotype ID:VII_EYE_ADNEXA
categorical.VIII_EAR_MASTOID.txt	1710174270056F5forCTG.txt.gz	0.304	0.09357	3.249	0.00116	0.004413	0.001491	1	0.009622	-0.000966	0.007443	Diseases of the ear and mastoid process	FALSE				361194	5252	355942	UK Biobank	https://docs.google.com/spreadsheets/d/1kvPoupSzsSFBNSztMzl04xMoSC3Kcx3CrjVf4yBmESU/edit?ts=5b5f17db#gid=227859291	PHESANT Transformation:NA-Notes:NA-Variable type:categorical-Phenotype ID:VIII_EAR_MASTOID
categorical.X_RESPIRATORY.txt	1710174270056F5forCTG.txt.gz	0.4303	0.06172	6.972	3.12E-12	0.0114	0.001733	1.011	0.01044	0.003626	0.008563	Diseases of the respiratory system	FALSE	Pulmonary			361194	25381	335813	UK Biobank	https://docs.google.com/spreadsheets/d/1kvPoupSzsSFBNSztMzl04xMoSC3Kcx3CrjVf4yBmESU/edit?ts=5b5f17db#gid=227859291	PHESANT Transformation:NA-Notes:NA-Variable type:categorical-Phenotype ID:X_RESPIRATORY
categorical.XII_SKIN_SUBCUTAN.txt	1710174270056F5forCTG.txt.gz	0.348	0.1007	3.457	0.000546	0.005556	0.001938	1.012	0.01057	0.000828	0.00799	Diseases of the skin and subcutaneous tissue	FALSE				361194	27074	334120	UK Biobank	https://docs.google.com/spreadsheets/d/1kvPoupSzsSFBNSztMzl04xMoSC3Kcx3CrjVf4yBmESU/edit?ts=5b5f17db#gid=227859291	PHESANT Transformation:NA-Notes:NA-Variable type:categorical-Phenotype ID:XII_SKIN_SUBCUTAN
categorical.XIII_MUSCULOSKELET.txt	1710174270056F5forCTG.txt.gz	0.4122	0.03598	11.46	2.17E-30	0.0422	0.00302	1.018	0.01442	-0.01061	0.009213	Diseases of the musculoskeletal system and connective tissue	FALSE	Skeletal			361194	77099	284095	UK Biobank	https://docs.google.com/spreadsheets/d/1kvPoupSzsSFBNSztMzl04xMoSC3Kcx3CrjVf4yBmESU/edit?ts=5b5f17db#gid=227859291	PHESANT Transformation:NA-Notes:NA-Variable type:categorical-Phenotype ID:XIII_MUSCULOSKELET
categorical.XIV_GENITOURINARY.txt	1710174270056F5forCTG.txt.gz	0.4191	0.05004	8.377	5.44E-17	0.01757	0.00208	1.003	0.0104	-0.000174	0.00846	Diseases of the genitourinary system	FALSE	Other (physical health)			361194	71620	289574	UK Biobank	https://docs.google.com/spreadsheets/d/1kvPoupSzsSFBNSztMzl04xMoSC3Kcx3CrjVf4yBmESU/edit?ts=5b5f17db#gid=227859291	PHESANT Transformation:NA-Notes:NA-Variable type:categorical-Phenotype ID:XIV_GENITOURINARY
categorical.XIX_INJURY_POISON.txt	1710174270056F5forCTG.txt.gz	0.3747	0.04884	7.673	1.68E-14	0.01639	0.001983	1.004	0.01032	0.00145	0.008532	Injury, poisoning and certain other consequences of external causes	FALSE	Wellbeing			361194	44796	316398	UK Biobank	https://docs.google.com/spreadsheets/d/1kvPoupSzsSFBNSztMzl04xMoSC3Kcx3CrjVf4yBmESU/edit?ts=5b5f17db#gid=227859291	PHESANT Transformation:NA-Notes:NA-Variable type:categorical-Phenotype ID:XIX_INJURY_POISON
categorical.XV_PREGNANCY_BIRTH.txt	1710174270056F5forCTG.txt.gz	-0.3296	0.1303	-2.53	0.0114	0.002729	0.001423	1.006	0.008632	0.006301	0.00757	Pregnancy, childbirth and the puerperium	FALSE				361194	11959	349235	UK Biobank	https://docs.google.com/spreadsheets/d/1kvPoupSzsSFBNSztMzl04xMoSC3Kcx3CrjVf4yBmESU/edit?ts=5b5f17db#gid=227859291	PHESANT Transformation:NA-Notes:NA-Variable type:categorical-Phenotype ID:XV_PREGNANCY_BIRTH
categorical.XVIII_MISCFINDINGS.txt	1710174270056F5forCTG.txt.gz	0.5263	0.04253	12.37	3.60E-35	0.02925	0.002295	1.023	0.0116	-0.008414	0.008582	Symptoms, signs and abnormal clinical and laboratory findings, not elsewhere classified	FALSE	Other (physical health)			361194	97602	263592	UK Biobank	https://docs.google.com/spreadsheets/d/1kvPoupSzsSFBNSztMzl04xMoSC3Kcx3CrjVf4yBmESU/edit?ts=5b5f17db#gid=227859291	PHESANT Transformation:NA-Notes:NA-Variable type:categorical-Phenotype ID:XVIII_MISCFINDINGS
categorical.XXI_HEALTHFACTORS.txt	1710174270056F5forCTG.txt.gz	0.3922	0.08994	4.36	1.30E-05	0.006015	0.00169	1.02	0.009798	0.008095	0.00741	Factors influencing health status and contact with health services	FALSE	Wellbeing			361194	45947	315247	UK Biobank	https://docs.google.com/spreadsheets/d/1kvPoupSzsSFBNSztMzl04xMoSC3Kcx3CrjVf4yBmESU/edit?ts=5b5f17db#gid=227859291	PHESANT Transformation:NA-Notes:NA-Variable type:categorical-Phenotype ID:XXI_HEALTHFACTORS
categorical.Z03.txt	1710174270056F5forCTG.txt.gz	0.3041	0.1043	2.915	0.003562	0.003541	0.001425	0.993	0.008895	3.19E-05	0.007997	Diagnoses - main ICD10: Z03 Medical observation and evaluation for suspected diseases and conditions	FALSE				361194	4951	356243	UK Biobank	https://docs.google.com/spreadsheets/d/1kvPoupSzsSFBNSztMzl04xMoSC3Kcx3CrjVf4yBmESU/edit?ts=5b5f17db#gid=227859291	PHESANT Transformation:NA-Notes:NA-Variable type:categorical-Phenotype ID:Z03
categorical.Z09.txt	1710174270056F5forCTG.txt.gz	0.1813	0.09224	1.965	0.0494	0.004669	0.001427	1.001	0.009067	0.003435	0.008476	Diagnoses - main ICD10: Z09 Follow-up examination after treatment for conditions other than malignant neoplasms	FALSE				361194	8464	352730	UK Biobank	https://docs.google.com/spreadsheets/d/1kvPoupSzsSFBNSztMzl04xMoSC3Kcx3CrjVf4yBmESU/edit?ts=5b5f17db#gid=227859291	PHESANT Transformation:NA-Notes:NA-Variable type:categorical-Phenotype ID:Z09
categorical.Z36.txt	1710174270056F5forCTG.txt.gz	-0.1418	0.08779	-1.615	0.1063	0.004418	0.00171	0.984	0.009823	0.003442	0.007514	Diagnoses - main ICD10: Z36 Antenatal screening	FALSE				361194	841	360353	UK Biobank	https://docs.google.com/spreadsheets/d/1kvPoupSzsSFBNSztMzl04xMoSC3Kcx3CrjVf4yBmESU/edit?ts=5b5f17db#gid=227859291	PHESANT Transformation:NA-Notes:NA-Variable type:categorical-Phenotype ID:Z36
categorical.Z42.txt	1710174270056F5forCTG.txt.gz	0.1721	0.08811	1.954	0.05074	0.003968	0.001594	1.002	0.009832	-0.004485	0.007281	Diagnoses - main ICD10: Z42 Follow-up care involving plastic surgery	FALSE				361194	1963	359231	UK Biobank	https://docs.google.com/spreadsheets/d/1kvPoupSzsSFBNSztMzl04xMoSC3Kcx3CrjVf4yBmESU/edit?ts=5b5f17db#gid=227859291	PHESANT Transformation:NA-Notes:NA-Variable type:categorical-Phenotype ID:Z42
categorical.Z47.txt	1710174270056F5forCTG.txt.gz	0.1746	0.1028	1.698	0.08959	0.003553	0.001626	0.999	0.01005	0.01299	0.008014	Diagnoses - main ICD10: Z47 Other orthopaedic follow-up care	FALSE				361194	2774	358420	UK Biobank	https://docs.google.com/spreadsheets/d/1kvPoupSzsSFBNSztMzl04xMoSC3Kcx3CrjVf4yBmESU/edit?ts=5b5f17db#gid=227859291	PHESANT Transformation:NA-Notes:NA-Variable type:categorical-Phenotype ID:Z47
CD_Lange_et_al.ctgvl	1710174270056F5forCTG.txt.gz	0.06063	0.03308	1.833	0.06686	0.4614	0.06072	1.086	0.02718	-0.009696	0.008927	Crohn's disease (CD)	FALSE				40266	12194	28072	IIBDGC + UK	ftp://ftp.sanger.ac.uk/pub/project/humgen/summary_statistics/human/2016-11-07/	https://www.nature.com/articles/ng.3760
CD_Liu_et_al.tsv	1710174270056F5forCTG.txt.gz	0.05789	0.0397	1.458	0.1448	0.3043	0.04192	1.023	0.0184	0.001573	0.008736	Crohn's disease (CD)	FALSE				20883	5956	14927	IIBDGC	ftp://ftp.sanger.ac.uk/pub/consortia/ibdgenetics/iibdgc-trans-ancestry-filtered-summary-stats.tgz	https://www.nature.com/articles/ng.3359
CD_prognosis_GWA_results.csv.ctg2	1710174270056F5forCTG.txt.gz	0.1167	0.1554	0.7505	0.4529	0.03132	0.02874	1.013	0.01015	-0.000236	0.008018	Poor prognosis in Crohn's disease	FALSE				2734	2734		European	https://www.ebi.ac.uk/gwas/publications/28067912	Poor prognosis in Crohn's disease
chronic_pain-bgen.stats.all	1710174270056F5forCTG.txt.gz	0.4867	0.02944	16.54	2.03E-61	0.07075	0.003297	1.041	0.01555	0.006181	0.01026	Multisite chronic pain	TRUE	Pain	Physical health	Chronic pain	380000			UK Biobank	http://researchdata.gla.ac.uk/822/	large-scale genome-wide association study (GWAS) of Multisite chronic pain (MCP) in ~380,000 UK Biobank participants. MCP was defined as the sum of body sites at which chronic pain (at least 3 months duration) was recorded: 0 to 7 sites. Those who answered that they had chronic pain ‚Äòall over the body‚Äô were excluded from the GWAS as there is some evidence that this phenotype relating to widespread pain can be substantially different from more localised chronic pain and should not, therefore, be considered a logical extension of the multisite scale.
ckqny.scz2snpres.ctgvl	1710174270056F5forCTG.txt.gz	0.1292	0.02867	4.508	6.55E-06	0.2225	0.01062	1.104	0.018	0.004562	0.0113	Schizophrenia	FALSE	Psychiatric			150064	36989	113075	PGC	https://www.med.unc.edu/pgc/results-and-downloads/	GWAS made available by PGC. Please go to reference for details
COGNITIVE_PERFORMANCE.Lee_et_al_2018.txt	1710174270056F5forCTG.txt.gz	-0.5289	0.02404	-22	2.68E-107	0.1949	0.008034	0.997	0.02052	-0.008748	0.01122	Cognitive Performance	TRUE	Cognitive	Cognitive	Cognitive performance	257841			SSGAC	https://www.thessgac.org/data	Cognitive Performance (CP) meta-analysis of all discovery cohorts.
COPD_Bothsex_eur_inv_var_meta_GBMI_052021_nbbkgt1.txt.gz.ctgvl	1710174270056F5forCTG.txt.gz	0.374	0.03681	10.16	2.96E-24	0.01758	0.001351	1.028	0.0144	0.005004	0.009385	COPD (EUR Biobanks)	TRUE	Pulmonary	Physical health	Chronic obstructive pulmonary disease	995917	58559	937358	Global Biobank Meta-analysis Initiative Meta-analysis Initiative: powering genetic discovery across human diseases. 2029	https://www.globalbiobankmeta.org/resources	https://www.sciencedirect.com/science/article/pii/S2666979X22001410?via%3Dihub

COPD_Bothsex_inv_var_meta_GBMI_052021_nbbkgt1.txt.gz.ctgvl	1710174270056F5forCTG.txt.gz	0.3447	0.03586	9.612	7.13E-22	0.01263	0.00103	1.031	0.01463	0.01018	0.009325	COPD (All Biobanks)	FALSE	Pulmonary			1392366	81568	1310798	Global Biobank Meta-analysis Initiative Meta-analysis Initiative: powering genetic discovery across human diseases. 2030	https://www.globalbiobankmeta.org/resources	https://www.sciencedirect.com/science/article/pii/S2666979X22001410?via%3Dihub
CTACK.data.freq.final	1710174270056F5forCTG.txt.gz	0.226	0.2045	1.105	0.2692	0.04932	0.06178	0.993	0.009778	-0.00648	0.007711	Cutaneous T-cell-attracting chemokine	FALSE				8293			Young Finns Study (YFS)	http://www.computationalmedicine.fi/data	Genome-wide Association Study Identifies 27 Loci Influencing Concentrations of Circulating Cytokines and Growth Factors
D22.gwas.imputed_v3.both_sexes.txt	1710174270056F5forCTG.txt.gz	-0.05106	0.3488	-0.1464	0.8836	0.000599	0.001633	1.012	0.009282	0.009165	0.008037	Diagnoses - main ICD10: D22 Melanocytic naevi	FALSE				361194	3501	357693	UK Biobank	http://www.nealelab.is/uk-biobank/	D22 UK Biobank
daner_PGC_BIP32b_mds7a_0416a.tsv	1710174270056F5forCTG.txt.gz	0.1054	0.03685	2.86	0.004235	0.7214	0.04408	1.049	0.01369	0.004351	0.00938	Bipolar Disorder	FALSE				51710	20352	31358	Psychiatric Genomics Consortium	https://www.med.unc.edu/pgc/results-and-downloads/	
dmfs_dentures_combined.txt.ctgvl	1710174270056F5forCTG.txt.gz	0.1905	0.03288	5.795	6.81E-09	0.08058	0.004231	1.017	0.01671	0.0192	0.009349	Decayed, Missing and Filled tooth Surfaces (DMFS) + Dentures	FALSE	Dental			487823	104506	383317	UK Biobank + GLIDE	https://data.bris.ac.uk/data/dataset/2j2rqgzedxlq02oqbb4vmycnc2	Single-variant association statistics from GLIDE and UKB were combined using a z-score genome-wide meta-analysis weighted by effective sample size. There were two principal analyses; one combining DMFS (n,Äâ=,Äâ26,792 from nine studies) and dentures (ncases,Äâ=,Äâ77,714, ncontrols,Äâ=,Äâ383,317)
EDUCATION_ATTAINMENT.Lee_et_al_2018.txt	1710174270056F5forCTG.txt.gz	-0.3189	0.02279	-13.99	1.71E-44	0.1045	0.003551	1.04	0.02624	-0.008079	0.01179	Educational Attainment	FALSE	Education			766000			SSGAC	https://www.thessgac.org/data	Educational attainment (EA) meta-analysis of all discovery cohorts except 23andMe.
ENIGMA2_ICV.txt	1710174270056F5forCTG.txt.gz	-0.2306	0.09604	-2.401	0.01637	0.1288	0.04544	1.009	0.009701	0.003809	0.008115	Intracranial volume (ICV)	FALSE				30717			ENIGMA	http://enigma.ini.usc.edu/	GWAS of subcortical volumes (ENIGMA2)
ENIGMA2_MeanAccumbens.txt	1710174270056F5forCTG.txt.gz	0.04269	0.09685	0.4408	0.6593	0.1172	0.04615	0.973	0.009942	-0.002276	0.008378	Accumbens volume	FALSE				30718			ENIGMA	http://enigma.ini.usc.edu/	GWAS of subcortical volumes (ENIGMA2)
ENIGMA2_MeanCaudate.txt	1710174270056F5forCTG.txt.gz	-0.05452	0.05968	-0.9136	0.3609	0.2626	0.04916	0.964	0.01065	0.01134	0.007929	Caudate volume	FALSE				30720			ENIGMA	http://enigma.ini.usc.edu/	GWAS of subcortical volumes (ENIGMA2)
ENIGMA2_MeanHippocampus.txt	1710174270056F5forCTG.txt.gz	-0.1159	0.09215	-1.257	0.2087	0.1243	0.04946	0.991	0.01	0.005287	0.008249	Hippocampus volume	FALSE				30721			ENIGMA	http://enigma.ini.usc.edu/	GWAS of subcortical volumes (ENIGMA2)
ENIGMA2_MeanPallidum.txt	1710174270056F5forCTG.txt.gz	-0.0654	0.07601	-0.8604	0.3895	0.1536	0.0544	0.98	0.01122	0.0001547	0.007935	Pallidum volume	FALSE				30722			ENIGMA	http://enigma.ini.usc.edu/	GWAS of subcortical volumes (ENIGMA2)
ENIGMA2_MeanPutamen.txt	1710174270056F5forCTG.txt.gz	0.02043	0.06386	0.3198	0.7491	0.2645	0.05075	0.96	0.009808	-0.006079	0.00862	Putamen volume	FALSE				30723			ENIGMA	http://enigma.ini.usc.edu/	GWAS of subcortical volumes (ENIGMA2)
ENIGMA2_MeanThalamus.txt	1710174270056F5forCTG.txt.gz	-0.001193	0.08104	-0.01473	0.9883	0.1135	0.04435	0.991	0.00988	-0.000808	0.007306	Thalamus volume	FALSE				30724			ENIGMA	http://enigma.ini.usc.edu/	GWAS of subcortical volumes (ENIGMA2)
eo_baso_sum_N171771_narrow_form.txt.common	1710174270056F5forCTG.txt.gz	-0.07048	0.02877	-2.45	0.01429	0.1768	0.02872	0.957	0.05076	0.01624	0.01006	Sum eosinophil basophil counts	FALSE				173480			UK Biobank and INTERVAL	http://www.bloodcellgenetics.org/	Aggregate count of eosinophils and basophils per unit volume of blood
eo_N172275_narrow_form.txt.common	1710174270056F5forCTG.txt.gz	-0.06808	0.02932	-2.322	0.02025	0.1842	0.0307	0.959	0.05506	0.01635	0.01026	Eosinophil count	FALSE				173481			UK Biobank and INTERVAL	http://www.bloodcellgenetics.org/	Count of eosinophils per unit volume of blood
eo_p_gran_N170536_narrow_form.txt.common	1710174270056F5forCTG.txt.gz	-0.0764	0.02923	-2.613	0.008963	0.1713	0.02666	0.961	0.04949	0.01453	0.01019	Eosinophil percentage of granulocytes	FALSE				173482			UK Biobank and INTERVAL	http://www.bloodcellgenetics.org/	Percentage of granulocytes that are eosinophils
eo_p_N172378_narrow_form.txt.common	1710174270056F5forCTG.txt.gz	-0.07616	0.02874	-2.65	0.008057	0.1771	0.02846	0.964	0.05223	0.01571	0.01019	Eosinophil percentage of white cells	FALSE				173483			UK Biobank and INTERVAL	http://www.bloodcellgenetics.org/	Percentage of white cells that are eosinophils
Eotaxin.data.freq.final	1710174270056F5forCTG.txt.gz	-0.1246	0.09455	-1.318	0.1875	0.1479	0.0759	0.998	0.01091	0.01072	0.007788	Eotaxin	FALSE				8293			Young Finns Study (YFS)	http://www.computationalmedicine.fi/data	Genome-wide Association Study Identifies 27 Loci Influencing Concentrations of Circulating Cytokines and Growth Factors
EUR_bleedinggums.txt.pre_ctgvl.freqs.ctgvl	1710174270056F5forCTG.txt.gz	0.1378	0.04335	3.179	0.001475	0.04454	0.00388	1.034	0.01259	-0.006095	0.008745	Bleeding gums	FALSE				461031	60210	400821	UK Biobank	https://data.bris.ac.uk/data/dataset/2j2rqgzedxlq02oqbb4vmycnc2	UK Biobank GWAS on having bleeding gums
EUR_dentures.txt.pre_ctgvl.freqs.ctgvl	1710174270056F5forCTG.txt.gz	0.2072	0.0342	6.06	1.36E-09	0.0892	0.004806	1.092	0.01753	0.01851	0.009754	Dentures	FALSE	Dental			461031	77714	383317	UK Biobank	https://data.bris.ac.uk/data/dataset/2j2rqgzedxlq02oqbb4vmycnc2	UK Biobank GWAS on having dentures
EUR_looseteeth.txt.pre_ctgvl.freqs.ctgvl	1710174270056F5forCTG.txt.gz	0.3784	0.05059	7.481	7.39E-14	0.07839	0.01152	1.042	0.01172	-0.000417	0.008406	Loose teeth	FALSE	Dental			461031	18979	442052	UK Biobank	https://data.bris.ac.uk/data/dataset/2j2rqgzedxlq02oqbb4vmycnc2	UK Biobank GWAS on loose teeth
EUR_painfulgums.txt.pre_ctgvl.freqs.ctgvl	1710174270056F5forCTG.txt.gz	0.2576	0.06798	3.79	0.000151	0.05265	0.01179	1.027	0.01019	0.003846	0.007808	Painful gums	FALSE				461031	13311	447720	UK Biobank	https://data.bris.ac.uk/data/dataset/2j2rqgzedxlq02oqbb4vmycnc2	UK Biobank GWAS on painful gums
EUR_toothache.txt.pre_ctgvl.freqs.ctgvl	1710174270056F5forCTG.txt.gz	0.3141	0.06186	5.078	3.82E-07	0.05079	0.008366	1.019	0.009984	0.02099	0.007706	Toothache	FALSE	Dental			461031	18959	442072	UK Biobank	https://data.bris.ac.uk/data/dataset/2j2rqgzedxlq02oqbb4vmycnc2	UK Biobank GWAS on toothache
EUR_ulcers.txt.pre_ctgvl.freqs.ctgvl	1710174270056F5forCTG.txt.gz	0.1315	0.04169	3.153	0.001617	0.07913	0.009999	1.041	0.02352	0.006833	0.009491	Mouth ulcers	FALSE				461031	47091	413940	UK Biobank	https://data.bris.ac.uk/data/dataset/2j2rqgzedxlq02oqbb4vmycnc2	UK Biobank GWAS on mouth ulcerls
FGFBasic.data.freq.final	1710174270056F5forCTG.txt.gz	-0.0348	0.2786	-0.1249	0.9006	0.02008	0.06644	1.006	0.01055	-0.002418	0.007183	Fibroblast growth factor (FGF-basic)	FALSE				8293			Young Finns Study (YFS)	http://www.computationalmedicine.fi/data	Genome-wide Association Study Identifies 27 Loci Influencing Concentrations of Circulating Cytokines and Growth Factors
GCSF.data.freq.final	1710174270056F5forCTG.txt.gz	0.05752	0.234	0.2458	0.8059	0.02767	0.06765	1.008	0.01118	-0.000386	0.007975	Granulocyte-colony stimulating factor	FALSE				8293			Young Finns Study (YFS)	http://www.computationalmedicine.fi/data	Genome-wide Association Study Identifies 27 Loci Influencing Concentrations of Circulating Cytokines and Growth Factors
GCST011096_buildGRCh37.tsv.withFREQ	1710174270056F5forCTG.txt.gz	0.009366	0.04918	0.1904	0.849	0.2496	0.05087	1.105	0.02693	0.007218	0.009198	Systemic lupus erythematosus	FALSE				30604	30604		Eeast Asian and European	https://www.ebi.ac.uk/gwas/studies/GCST90100586	GWAS Meta-analysis Systemic lupus erythematosus
Gout_Bothsex_eur_inv_var_meta_GBMI_052021_nbbkgt1.txt.gz.ctgvl	1710174270056F5forCTG.txt.gz	0.0986	0.03575	2.758	0.00581	0.01677	0.005618	0.941	0.06381	0.006232	0.00863	Gout (EUR Biobanks)	FALSE				1051653	24278	1027375	Global Biobank Meta-analysis Initiative Meta-analysis Initiative: powering genetic discovery across human diseases. 2031	https://www.globalbiobankmeta.org/resources	https://www.sciencedirect.com/science/article/pii/S2666979X22001410?via%3Dihub
Gout_Bothsex_inv_var_meta_GBMI_052021_nbbkgt1.txt.gz.ctgvl	1710174270056F5forCTG.txt.gz	0.09961	0.03354	2.97	0.00298	0.01409	0.004135	0.953	0.05407	0.002512	0.009225	Gout (All Biobanks)	FALSE				1485233	37105	1448128	Global Biobank Meta-analysis Initiative Meta-analysis Initiative: powering genetic discovery across human diseases. 2032	https://www.globalbiobankmeta.org/resources	https://www.sciencedirect.com/science/article/pii/S2666979X22001410?via%3Dihub

gran_N169822_narrow_form.txt.common	1710174270056F5forCTG.txt.gz	0.003838	0.03278	0.1171	0.9068	0.156	0.0238	0.971	0.03955	0.003273	0.00955	Granulocyte count	FALSE				173484			UK Biobank and INTERVAL	http://www.bloodcellgenetics.org/	Aggregate count of granulocytes per unit volume of blood
gran_p_myeloid_wbc_N169545_narrow_form.txt.common	1710174270056F5forCTG.txt.gz	-0.01774	0.03277	-0.5413	0.5883	0.1442	0.02171	1.065	0.05275	0.006066	0.01047	Granulocyte percentage of myeloid white cells	FALSE				173485			UK Biobank and INTERVAL	http://www.bloodcellgenetics.org/	Percentage of myeloid white cells that are granulocytes
HCM_Bothsex_eur_inv_var_meta_GBMI_052021_nbbkgt1.txt.gz.ctgvl	1710174270056F5forCTG.txt.gz	0.0697	0.2295	0.3038	0.7613	0.000257	0.00054	0.997	0.008686	-0.000638	0.007432	Cardiomyopathy(hypertrophic, obstructive) (HCM) (EUR Biobanks)	FALSE				922988	1883	921105	Global Biobank Meta-analysis Initiative Meta-analysis Initiative: powering genetic discovery across human diseases. 2033	https://www.globalbiobankmeta.org/resources	https://www.sciencedirect.com/science/article/pii/S2666979X22001410?via%3Dihub
HCM_Bothsex_inv_var_meta_GBMI_052021_nbbkgt1.txt.gz.ctgvl	1710174270056F5forCTG.txt.gz	0.1736	0.2268	0.7653	0.4441	0.000255	0.000417	0.999	0.008672	-0.006285	0.006962	Cardiomyopathy(hypertrophic, obstructive) (HCM) (All Biobanks)	FALSE				1193060	2993	1190067	Global Biobank Meta-analysis Initiative Meta-analysis Initiative: powering genetic discovery across human diseases. 2034	https://www.globalbiobankmeta.org/resources	https://www.sciencedirect.com/science/article/pii/S2666979X22001410?via%3Dihub
hct_N173039_narrow_form.txt.common	1710174270056F5forCTG.txt.gz	-0.02135	0.03581	-0.5962	0.5511	0.1216	0.01566	1.041	0.04261	0.006141	0.01043	Hematocrit	FALSE				173486			UK Biobank and INTERVAL	http://www.bloodcellgenetics.org/	Volume fraction of blood occupied by red cells
HF_Bothsex_eur_inv_var_meta_GBMI_052021_nbbkgt1.txt.gz.ctgvl	1710174270056F5forCTG.txt.gz	0.2756	0.04232	6.512	7.40E-11	0.009029	0.000727	1.015	0.01212	-0.002403	0.008508	Heart Failure (HF) (EUR Biobanks)	TRUE	Cardiac	Physical health	Heart failure	1020441	52496	967945	Global Biobank Meta-analysis Initiative Meta-analysis Initiative: powering genetic discovery across human diseases. 2035	https://www.globalbiobankmeta.org/resources	https://www.sciencedirect.com/science/article/pii/S2666979X22001410?via%3Dihub
HF_Bothsex_inv_var_meta_GBMI_052021_nbbkgt1.txt.gz.ctgvl	1710174270056F5forCTG.txt.gz	0.2623	0.03965	6.615	3.70E-11	0.007226	0.000571	1.023	0.01261	-0.004026	0.008439	Heart Failure (HF) (All Biobanks)	FALSE	Cardiac			1354739	68408	1286331	Global Biobank Meta-analysis Initiative Meta-analysis Initiative: powering genetic discovery across human diseases. 2036	https://www.globalbiobankmeta.org/resources	https://www.sciencedirect.com/science/article/pii/S2666979X22001410?via%3Dihub
hgb_N172925_narrow_form.txt.common	1710174270056F5forCTG.txt.gz	-0.01531	0.03617	-0.4233	0.6721	0.1205	0.01508	1.043	0.04465	0.004812	0.01031	Hemoglobin concentration	FALSE				173487			UK Biobank and INTERVAL	http://www.bloodcellgenetics.org/	Concentration of hemoglobin with respect to unit of volume of blood
hlr_N170761_narrow_form.txt.common	1710174270056F5forCTG.txt.gz	0.02429	0.03425	0.7092	0.4782	0.1523	0.02442	1.083	0.07733	0.001366	0.01099	High light scatter reticulocyte count	FALSE	Ophthalmic			173488			UK Biobank and INTERVAL	http://www.bloodcellgenetics.org/	Count of high RNA content (immature) reticulocytes per unit volume of blood
hlr_p_N170763_narrow_form.txt.common	1710174270056F5forCTG.txt.gz	0.02811	0.03392	0.8288	0.4072	0.1523	0.02419	1.082	0.07744	0.001891	0.01106	High light scatter reticulocyte percentage of red cells	FALSE				173489			UK Biobank and INTERVAL	http://www.bloodcellgenetics.org/	Immature reticulocyte count as a percentage of red blood cell count
ibd_EAS_EUR_SiKJEF_meta_CD.TBL.txt.eas.ctg	1710174270056F5forCTG.txt.gz	0.03067	0.04201	0.73	0.4654	0.346	0.05352	1.086	0.02174	-0.001777	0.007702	CD (EAS) 2023	FALSE				22828	7372	15456	IIBDGC	https://www.ibdgenetics.org/	Crohns disease suceptibility East Asian GWAS Meta-analysis
ibd_EAS_EUR_SiKJEF_meta_CD.TBL.txt.eur.ctg	1710174270056F5forCTG.txt.gz	0.05837	0.0343	1.702	0.08876	0.9012	0.1286	1.091	0.02838	-0.005506	0.009198	CD (EUR) 2023	FALSE				40266	12194	28072	IIBDGC	https://www.ibdgenetics.org/	Crohns disease suceptibility EUR GWAS Meta-analysis
ibd_EAS_EUR_SiKJEF_meta_IBD.TBL.txt.eas.ctg	1710174270056F5forCTG.txt.gz	0.09636	0.04073	2.366	0.01798	0.3036	0.06069	1.104	0.0199	-0.009793	0.007255	IBD (EAS) 2023	FALSE				29849	14393	15456	IIBDGC	https://www.ibdgenetics.org/	IBD suceptibility East Asian GWAS Meta-analysis
ibd_EAS_EUR_SiKJEF_meta_IBD.TBL.txt.eur.ctg	1710174270056F5forCTG.txt.gz	0.02769	0.03581	0.7734	0.4393	0.8966	0.1085	1.135	0.02785	0.001687	0.009202	IBD (EUR) 2023	FALSE				59957	25042	34915	IIBDGC	https://www.ibdgenetics.org/	IBD suceptibility EUR GWAS Meta-analysis
ibd_EAS_EUR_SiKJEF_meta_UC.TBL.txt.eas.ctg	1710174270056F5forCTG.txt.gz	0.1593	0.05071	3.142	0.001681	0.1839	0.04823	1.067	0.01657	-0.01272	0.007399	UC (EAS) 2023	FALSE				22318	6862	15456	IIBDGC	https://www.ibdgenetics.org/	Ulcerative colitis suceptibility East Asian GWAS Meta-analysis
ibd_EAS_EUR_SiKJEF_meta_UC.TBL.txt.eur.ctg	1710174270056F5forCTG.txt.gz	-0.02143	0.04219	-0.5079	0.6116	0.5123	0.07488	1.114	0.02589	0.008024	0.008754	UC (EUR) 2023	FALSE				45975	12366	33609	IIBDGC	https://www.ibdgenetics.org/	Ulcerative colitis suceptibility EUR GWAS Meta-analysis
IBD_Lange_et_al.ctgvl	1710174270056F5forCTG.txt.gz	0.02876	0.03488	0.8247	0.4095	0.3083	0.03554	1.131	0.02637	-0.002074	0.009068	Inflammatory bowel disease (IBD)	FALSE				53124	25052	28072	IIBDGC + UK	ftp://ftp.sanger.ac.uk/pub/project/humgen/summary_statistics/human/2016-11-07/	https://www.nature.com/articles/ng.3760
IBD_Liu_et_al.tsv	1710174270056F5forCTG.txt.gz	0.04072	0.04066	1.001	0.3166	0.302	0.03987	1.075	0.01781	0.003299	0.008565	Inflammatory bowel disease (IBD)	FALSE				34652	12882	21770	IIBDGC	ftp://ftp.sanger.ac.uk/pub/consortia/ibdgenetics/iibdgc-trans-ancestry-filtered-summary-stats.tgz	https://www.nature.com/articles/ng.3359
IFNg.data.freq.final	1710174270056F5forCTG.txt.gz	0.01792	0.1021	0.1756	0.8606	0.1056	0.068	0.979	0.009847	-0.003333	0.007202	Interferon-gamma	FALSE				8293			Young Finns Study (YFS)	http://www.computationalmedicine.fi/data	Genome-wide Association Study Identifies 27 Loci Influencing Concentrations of Circulating Cytokines and Growth Factors
IL6.data.freq.final	1710174270056F5forCTG.txt.gz	-0.01566	0.1684	-0.093	0.9259	0.03871	0.06536	0.999	0.00983	-0.000104	0.007795	IL6	FALSE				8293			Young Finns Study (YFS)	http://www.computationalmedicine.fi/data	Genome-wide Association Study Identifies 27 Loci Influencing Concentrations of Circulating Cytokines and Growth Factors
IP10.data.freq.final	1710174270056F5forCTG.txt.gz	-0.2066	0.1585	-1.303	0.1926	0.07584	0.06156	1.002	0.01019	0.01604	0.00765	Interferon gamma-induced Protein 10 (IP10)	FALSE				8293			Young Finns Study (YFS)	http://www.computationalmedicine.fi/data	Genome-wide Association Study Identifies 27 Loci Influencing Concentrations of Circulating Cytokines and Growth Factors
IPF_Bothsex_eur_inv_var_meta_GBMI_052021_nbbkgt1.txt.ctgvl	1710174270056F5forCTG.txt.gz	0.2127	0.07389	2.879	0.003994	0.002628	0.000728	1.004	0.01189	-0.008276	0.008026	IPF (EUR Biobanks)	FALSE				953873	6257	947616	Global Biobank Meta-analysis Initiative	https://www.globalbiobankmeta.org/resources	https://www.sciencedirect.com/science/article/pii/S2666979X22001410?via%3Dihub
IPF_Bothsex_inv_var_meta_GBMI_052021_nbbkgt1.txt.ctgvl	1710174270056F5forCTG.txt.gz	0.1904	0.06687	2.846	0.004421	0.002337	0.000545	1.007	0.0112	-0.008951	0.007851	IPF (All Biobanks)	FALSE				1254748	8006	1246742	Global Biobank Meta-analysis Initiative	https://www.globalbiobankmeta.org/resources	https://www.sciencedirect.com/science/article/pii/S2666979X22001410?via%3Dihub

IPF_Bothsex_inv_var_meta_GBMI_with_Allen_021121.txt.ctgvl	1710174270056F5forCTG.txt.gz	0.1814	0.05624	3.225	0.001261	0.003785	0.000724	1.018	0.01554	-0.01483	0.00842	IPF (All Biobanks + Allen et al)	FALSE				1375570	11160	1364410	Global Biobank Meta-analysis Initiative	https://www.globalbiobankmeta.org/resources	https://www.sciencedirect.com/science/article/pii/S2666979X22001410?via%3Dihub
irf_N170548_narrow_form.txt.common	1710174270056F5forCTG.txt.gz	0.03352	0.03848	0.8711	0.3837	0.1087	0.01977	1.069	0.06992	-0.000102	0.01058	Immature fraction of reticulocytes	FALSE				173490			UK Biobank and INTERVAL	http://www.bloodcellgenetics.org/	Fraction of reticulocytes with high RNA content, as measured by light scatter
jointGwasMc_HDL.txt.clean	1710174270056F5forCTG.txt.gz	-0.1875	0.05925	-3.165	0.00155	0.05023	0.03047	1.046	0.1391	-0.001847	0.008894	High-density lipoprotein (HDL)	FALSE				188577			GLGC	http://csg.sph.umich.edu/willer/public/lipids2013/	Global LipidsGenetics Consortium
jointGwasMc_LDL.txt.clean	1710174270056F5forCTG.txt.gz	0.1237	0.109	1.135	0.2566	0.02745	0.035	1.107	0.1455	-0.005406	0.01146	Low-density lipoprotein (LDL)	FALSE				188578			GLGC	http://csg.sph.umich.edu/willer/public/lipids2013/	Global Lipids Genetics Consortium
jointGwasMc_TC.txt.clean	1710174270056F5forCTG.txt.gz	0.09793	0.06568	1.491	0.136	0.0471	0.029	1.053	0.1121	-0.01126	0.01101	Total cholesterol	FALSE				188579			GLGC	http://csg.sph.umich.edu/willer/public/lipids2013/	Global LipidsGenetics Consortium
jointGwasMc_TG.txt.clean	1710174270056F5forCTG.txt.gz	0.1428	0.03611	3.955	7.64E-05	0.09609	0.02724	0.869	0.04613	0.001478	0.00967	Triglycerides	FALSE	Metabolic			188580			GLGC	http://csg.sph.umich.edu/willer/public/lipids2013/	Global LipidsGenetics Consortium
Liu_2022_CD.tsv.rsid.ctg2	1710174270056F5forCTG.txt.gz	0.05912	0.03184	1.857	0.0633	1.066	0.1433	1.11	0.03036	-0.003857	0.00903	Crohns 2023	FALSE				367592	20873	346719	IIBDGC	https://www.nature.com/articles/s41588-023-01384-0	https://www.ibdgenetics.org/
Liu_2022_IBD.tsv.rsid.ctg2	1710174270056F5forCTG.txt.gz	0.03975	0.03185	1.248	0.2121	1.275	0.157	1.165	0.04028	-0.001654	0.009477	Inflammatory bowel disease 2023	FALSE				398668	45106	353562	IIBDGC	https://www.nature.com/articles/s41588-023-01384-0	https://www.ibdgenetics.org/
Liu_2022_UC.tsv.rsid.ctg2	1710174270056F5forCTG.txt.gz	0.01579	0.03578	0.4414	0.6589	0.8173	0.1171	1.137	0.03945	0.001326	0.009291	Ulcerative Colitis 2023	FALSE				375508	23252	352256	IIBDGC	null	https://www.nature.com/articles/s41588-023-01384-0
lymph_N171643_narrow_form.txt.common	1710174270056F5forCTG.txt.gz	-0.000587	0.03352	-0.0175	0.986	0.1771	0.01583	0.961	0.0305	0.002351	0.01073	Lymphocyte count	FALSE				173495			UK Biobank and INTERVAL	http://www.bloodcellgenetics.org/	Aggregate count of lymphoid cells per unit volume of blood
lymph_p_N171748_narrow_form.txt.common	1710174270056F5forCTG.txt.gz	-0.00152	0.03048	-0.04987	0.9602	0.1418	0.01364	0.963	0.02529	-0.000331	0.009473	Lymphocyte percentage of white cells	FALSE				173496			UK Biobank and INTERVAL	http://www.bloodcellgenetics.org/	Percentage of white cells that are lymphocytes
mch_N172332_narrow_form.txt.common	1710174270056F5forCTG.txt.gz	0.007977	0.02614	0.3052	0.7602	0.2178	0.03107	1.055	0.08147	0.008261	0.01055	Mean corpuscular hemoglobin	FALSE				173498			UK Biobank and INTERVAL	http://www.bloodcellgenetics.org/	Average mass of hemoglobin per red cell
mchc_N172851_narrow_form.txt.common	1710174270056F5forCTG.txt.gz	0.02074	0.03499	0.5927	0.5534	0.08447	0.01211	1.015	0.03276	-0.001238	0.009192	Mean corpuscular hemoglobin concentration	FALSE				173497			UK Biobank and INTERVAL	http://www.bloodcellgenetics.org/	Concentration of hemoglobin with respect to unit of volume occupied by red cells
MCP1.data.freq.final	1710174270056F5forCTG.txt.gz	0.1023	0.1143	0.895	0.3708	0.1549	0.104	0.995	0.01187	-0.001496	0.007806	Monocyte Chemotactic Protein-1 (MCP1)	FALSE				8293			Young Finns Study (YFS)	http://www.computationalmedicine.fi/data	Genome-wide Association Study Identifies 27 Loci Influencing Concentrations of Circulating Cytokines and Growth Factors
mcv_N172433_narrow_form.txt.common	1710174270056F5forCTG.txt.gz	0.003286	0.02629	0.125	0.9005	0.2317	0.02948	1.052	0.07969	0.01107	0.01116	Mean corpuscular volume	FALSE				173499			UK Biobank and INTERVAL	http://www.bloodcellgenetics.org/	Mean volume of red blood cells
MIF.data.freq.final	1710174270056F5forCTG.txt.gz	-0.0689	0.1803	-0.3822	0.7023	0.0422	0.06114	1.003	0.009548	0.01729	0.007593	Macrophage migration Inhibitory Factor (MIF)	FALSE				8293			Young Finns Study (YFS)	http://www.computationalmedicine.fi/data	Genome-wide Association Study Identifies 27 Loci Influencing Concentrations of Circulating Cytokines and Growth Factors
mono_N170721_narrow_form.txt.common	1710174270056F5forCTG.txt.gz	0.01429	0.03407	0.4193	0.675	0.1709	0.02573	1.117	0.06644	-0.002166	0.01068	Monocyte count	FALSE				173500			UK Biobank and INTERVAL	http://www.bloodcellgenetics.org/	Count of monocytes per unit volume of blood
mono_p_N170494_narrow_form.txt.common	1710174270056F5forCTG.txt.gz	0.01527	0.03377	0.4522	0.6511	0.1518	0.02466	1.104	0.06423	-0.004438	0.01089	Monocyte percentage of white cells	FALSE				173501			UK Biobank and INTERVAL	http://www.bloodcellgenetics.org/	Percentage of white cells that are monocytes
mpv_N164454_narrow_form.txt.common	1710174270056F5forCTG.txt.gz	-0.008097	0.02896	-0.2796	0.7798	0.2485	0.04414	1.056	0.1064	-0.003109	0.01027	Mean platelet volume	FALSE				173502			UK Biobank and INTERVAL	http://www.bloodcellgenetics.org/	Mean volume of platelets
myeloid_wbc_N169219_narrow_form.txt.common	1710174270056F5forCTG.txt.gz	0.004839	0.03319	0.1458	0.8841	0.1591	0.0229	0.977	0.03904	0.002322	0.009627	Myeloid white cell count	FALSE				173503			UK Biobank and INTERVAL	http://www.bloodcellgenetics.org/	Aggregate count of myeloid white cells
neut_eo_sum_N170384_narrow_form.txt.common	1710174270056F5forCTG.txt.gz	0.002518	0.03268	0.07707	0.9386	0.1551	0.02354	0.972	0.03934	0.003117	0.00953	Sum neutrophil eosinophil counts	FALSE				173504			UK Biobank and INTERVAL	http://www.bloodcellgenetics.org/	Aggregate count of neutrophils and eosinophils per unit volume of blood.
neut_N170702_narrow_form.txt.common	1710174270056F5forCTG.txt.gz	0.01412	0.03311	0.4264	0.6698	0.1545	0.02365	0.971	0.03952	0.0006338	0.009611	Neutrophil count	FALSE				173505			UK Biobank and INTERVAL	http://www.bloodcellgenetics.org/	Count of neutrophils per unit volume of blood
neut_p_gran_N170672_narrow_form.txt.common	1710174270056F5forCTG.txt.gz	0.08001	0.02875	2.783	0.005387	0.1619	0.02395	0.955	0.04414	-0.01597	0.01001	Neutrophil percentage of granulocytes	FALSE				173506			UK Biobank and INTERVAL	http://www.bloodcellgenetics.org/	Percentage of granulocytes that are neutrophils
neut_p_N171542_narrow_form.txt.common	1710174270056F5forCTG.txt.gz	0.01818	0.03138	0.5793	0.5624	0.138	0.01509	0.953	0.02835	-0.001548	0.009495	Neutrophil percentage of white cells	FALSE				173507			UK Biobank and INTERVAL	http://www.bloodcellgenetics.org/	Percentage of white cells that are neutrophils
pct_N164339_narrow_form.txt.common	1710174270056F5forCTG.txt.gz	0.001911	0.03155	0.06057	0.9517	0.1799	0.01813	1.071	0.06386	0.001217	0.01083	Plateletcrit	FALSE				173508			UK Biobank and INTERVAL	http://www.bloodcellgenetics.org/	Volume fraction of blood occupied by platelets
PDGFbb.data.freq.final	1710174270056F5forCTG.txt.gz	0.0328	0.1629	0.2013	0.8405	0.04854	0.07067	1.013	0.01278	-0.00639	0.007535	Platelet Derived Growth Factor BB	FALSE				8293			Young Finns Study (YFS)	http://www.computationalmedicine.fi/data	Genome-wide Association Study Identifies 27 Loci Influencing Concentrations of Circulating Cytokines and Growth Factors
pdw_N164433_narrow_form.txt.common	1710174270056F5forCTG.txt.gz	-0.01308	0.02975	-0.4396	0.6602	0.1752	0.02797	0.967	0.05381	-0.004809	0.009703	Platelet distribution width	FALSE				173509			UK Biobank and INTERVAL	http://www.bloodcellgenetics.org/	The spread of the platelet volume distribution. Note that Sysmex and Coulter use different statistics to measure spread.
perio_looseteeth_combined.ctgvl	1710174270056F5forCTG.txt.gz	0.2986	0.05312	5.621	1.90E-08	0.04335	0.006242	1.004	0.01082	0.007415	0.008463	Periodontitis + loose teeth	FALSE	Dental			506594	36332	470262	UK Biobank +GLIDE	https://data.bris.ac.uk/data/dataset/2j2rqgzedxlq02oqbb4vmycnc2	Single-variant association statistics from GLIDE and UKB were combined using a z-score genome-wide meta-analysis weighted by effective sample size combining periodontitis (ncases,Äâ=,Äâ17,353, ncontrols,Äâ=,Äâ28,210 from seven studies) and loose teeth (ncases,Äâ=,Äâ18,979, ncontrols,Äâ=,Äâ442,052)
plt_N166066_narrow_form.txt.common	1710174270056F5forCTG.txt.gz	0.00967	0.0307	0.315	0.7528	0.2101	0.02022	1.046	0.06303	0.0009889	0.01125	Platelet count	FALSE				173510			UK Biobank and INTERVAL	http://www.bloodcellgenetics.org/	Count of platelets per unit volume of blood
POAG_Bothsex_eur_inv_var_meta_GBMI_052021_nbbkgt1.txt.gz.ctgvl	1710174270056F5forCTG.txt.gz	-0.0429	0.04391	-0.9771	0.3285	0.007862	0.00091	1.022	0.01736	0.01123	0.008614	POAG (EUR Biobanks)	FALSE				1172905	16355	1156550	Global Biobank Meta-analysis Initiative Meta-analysis Initiative: powering genetic discovery across human diseases. 2037	https://www.globalbiobankmeta.org/resources	https://www.sciencedirect.com/science/article/pii/S2666979X22001410?via%3Dihub

POAG_Bothsex_inv_var_meta_GBMI_052021_nbbkgt1.txt.gz.ctgvl	1710174270056F5forCTG.txt.gz	-0.04408	0.04101	-1.075	0.2824	0.007104	0.000773	1.028	0.01717	0.01034	0.00862	POAG (All Biobanks)	FALSE				1487447	26848	1460599	Global Biobank Meta-analysis Initiative Meta-analysis Initiative: powering genetic discovery across human diseases. 2038	https://www.globalbiobankmeta.org/resources	https://www.sciencedirect.com/science/article/pii/S2666979X22001410?via%3Dihub
RANTES.data.freq.final	1710174270056F5forCTG.txt.gz	0.03517	0.1577	0.2229	0.8236	0.0486	0.05986	0.996	0.009583	-0.007158	0.007217	RANTES	FALSE				8293			Young Finns Study (YFS)	http://www.computationalmedicine.fi/data	Genome-wide Association Study Identifies 27 Loci Influencing Concentrations of Circulating Cytokines and Growth Factors
rbc_N172952_narrow_form.txt.common	1710174270056F5forCTG.txt.gz	-0.02211	0.02868	-0.7709	0.4408	0.1838	0.02313	1.036	0.04352	-0.001608	0.0111	Red blood cell count	FALSE				173511			UK Biobank and INTERVAL	http://www.bloodcellgenetics.org/	Count of red blood cells per unit volume of blood
rdw_N171529_narrow_form.txt.common	1710174270056F5forCTG.txt.gz	0.000955	0.03	0.03184	0.9746	0.1953	0.02855	0.951	0.05393	0.003494	0.01074	Red cell distribution width	FALSE				173512			UK Biobank and INTERVAL	http://www.bloodcellgenetics.org/	Coefficient of variation of red cell volume distribution
ret_N170641_narrow_form.txt.common	1710174270056F5forCTG.txt.gz	0.02314	0.03221	0.7185	0.4724	0.1589	0.02769	1.074	0.08103	0.000412	0.01045	Reticulocyte count	FALSE				173513			UK Biobank and INTERVAL	http://www.bloodcellgenetics.org/	Count of reticulocytes per unit volume of blood
ret_p_N170690_narrow_form.txt.common	1710174270056F5forCTG.txt.gz	0.02924	0.03192	0.9159	0.3597	0.1594	0.02767	1.066	0.08122	0.0007647	0.01055	Reticulocyte fraction of red cells	FALSE				173514			UK Biobank and INTERVAL	http://www.bloodcellgenetics.org/	Percentage of red blood cells that are reticulocytes
SCGFb.data.freq.final	1710174270056F5forCTG.txt.gz	-0.1148	0.1614	-0.7117	0.4767	0.0577	0.06153	1	0.01029	0.008317	0.007747	Stem Cell Growth Factor beta	FALSE				8293			Young Finns Study (YFS)	http://www.computationalmedicine.fi/data	Genome-wide Association Study Identifies 27 Loci Influencing Concentrations of Circulating Cytokines and Growth Factors
SDF1a.data.freq.final	1710174270056F5forCTG.txt.gz	0.1945	0.2779	0.7	0.4839	0.03415	0.06178	0.989	0.00954	-0.01271	0.007461	Stromal Cell-Derived Factor 1alpha	FALSE				8293			Young Finns Study (YFS)	http://www.computationalmedicine.fi/data	Genome-wide Association Study Identifies 27 Loci Influencing Concentrations of Circulating Cytokines and Growth Factors
Stroke_Bothsex_eur_inv_var_meta_GBMI_052021_nbbkgt1.txt.gz.ctgvl	1710174270056F5forCTG.txt.gz	0.2891	0.05709	5.063	4.13E-07	0.004	0.000595	1.015	0.009843	0.01192	0.007913	Stroke (EUR Biobanks)	TRUE	Cardiac	Physical health	Stroke	1039382	34503	1004879	Global Biobank Meta-analysis Initiative Meta-analysis Initiative: powering genetic discovery across human diseases. 2039	https://www.globalbiobankmeta.org/resources	https://www.sciencedirect.com/science/article/pii/S2666979X22001410?via%3Dihub
Stroke_Bothsex_inv_var_meta_GBMI_052021_nbbkgt1.txt.gz.ctgvl	1710174270056F5forCTG.txt.gz	0.2123	0.04955	4.284	1.84E-05	0.004391	0.000514	1.017	0.01054	0.01293	0.008397	Stroke (All Biobanks)	FALSE	Cardiac			1370901	60176	1310725	Global Biobank Meta-analysis Initiative Meta-analysis Initiative: powering genetic discovery across human diseases. 2040	https://www.globalbiobankmeta.org/resources	https://www.sciencedirect.com/science/article/pii/S2666979X22001410?via%3Dihub
ThC_Bothsex_eur_inv_var_meta_GBMI_052021_nbbkgt1.txt.gz.ctgvl	1710174270056F5forCTG.txt.gz	0.06646	0.0558	1.191	0.2336	0.004307	0.001388	0.984	0.01427	-0.00875	0.008949	Thyroid cancer (ThC) (EUR Biobanks)	FALSE				1339769	6015	1333754	Global Biobank Meta-analysis Initiative Meta-analysis Initiative: powering genetic discovery across human diseases. 2041	https://www.globalbiobankmeta.org/resources	https://www.sciencedirect.com/science/article/pii/S2666979X22001410?via%3Dihub
ThC_Bothsex_inv_var_meta_GBMI_052021_nbbkgt1.txt.gz.ctgvl	1710174270056F5forCTG.txt.gz	0.06265	0.05308	1.18	0.2379	0.003914	0.001154	0.981	0.01444	-0.008934	0.008676	Thyroid cancer (ThC) (All Biobanks)	FALSE				1620354	6699	1613655	Global Biobank Meta-analysis Initiative Meta-analysis Initiative: powering genetic discovery across human diseases. 2042	https://www.globalbiobankmeta.org/resources	https://www.sciencedirect.com/science/article/pii/S2666979X22001410?via%3Dihub
UC_Lange_et_al.ctgvl	1710174270056F5forCTG.txt.gz	-0.01767	0.04194	-0.4212	0.6736	0.2246	0.03109	1.111	0.02475	0.005525	0.00849	Ulcerative colitis (UC)	FALSE				40438	12366	28072	IIBDGC + UK	ftp://ftp.sanger.ac.uk/pub/project/humgen/summary_statistics/human/2016-11-07/	https://www.nature.com/articles/ng.3760
UC_Liu_et_al.tsv	1710174270056F5forCTG.txt.gz	0.01082	0.0525	0.2061	0.8367	0.1661	0.0297	1.073	0.01653	0.004172	0.008149	Ulcerative colitis (UC)	FALSE				27432	6968	20464	IIBDGC	ftp://ftp.sanger.ac.uk/pub/consortia/ibdgenetics/iibdgc-trans-ancestry-filtered-summary-stats.tgz	https://www.nature.com/articles/ng.3359
UC_vs_CD.ctg	1710174270056F5forCTG.txt.gz	0.07239	0.05803	1.247	0.2122	2.305	0.4049	1.07	0.01442	-0.002764	0.008464	Crohn's disease vs ulcerative colitis	FALSE				12924	6968	5956	European	https://www.ebi.ac.uk/gwas/studies/GCST90016611	Crohn's disease vs ulcerative colitis
VTE_Bothsex_eur_inv_var_meta_GBMI_052021_nbbkgt1.txt.gz.ctgvl	1710174270056F5forCTG.txt.gz	0.1859	0.04613	4.031	5.56E-05	0.009042	0.001465	1.053	0.02877	-0.005116	0.008554	VTE (EUR Biobanks)	FALSE				915868	25146	890722	Global Biobank Meta-analysis Initiative Meta-analysis Initiative: powering genetic discovery across human diseases. 2043	https://www.globalbiobankmeta.org/resources	https://www.sciencedirect.com/science/article/pii/S2666979X22001410?via%3Dihub
VTE_Bothsex_inv_var_meta_GBMI_052021_nbbkgt1.txt.gz.ctgvl	1710174270056F5forCTG.txt.gz	0.1808	0.04551	3.974	7.07E-05	0.007885	0.001216	1.052	0.02795	-0.00312	0.008642	VTE (All Biobanks)	FALSE				1063277	27987	1035290	Global Biobank Meta-analysis Initiative Meta-analysis Initiative: powering genetic discovery across human diseases. 2044	https://www.globalbiobankmeta.org/resources	https://www.sciencedirect.com/science/article/pii/S2666979X22001410?via%3Dihub
wbc_N172435_narrow_form.txt.common	1710174270056F5forCTG.txt.gz	0.009175	0.03429	0.2676	0.789	0.1724	0.02088	0.98	0.03707	-0.000574	0.01012	White blood cell count	FALSE				173515			UK Biobank and INTERVAL	http://www.bloodcellgenetics.org/	Aggregate count of white cells per unit volume of blood
1710174270056F5forCTG.txt.gz.checked.sumstats.gz	1710174270056F5forCTG.txt.gz.checked.sumstats.gz	1	6.17E-08	16196000	0	0.095	0.0039	0.972	0.0124	0.9724	0.0124	Factor 5	FALSE	Psychiatric								

1710177371939ADHDforCTG.txt.gz.checked.sumstats.gz	1710174270056F5forCTG.txt.gz.checked.sumstats.gz	0.7222	0.026008	27.769	1.04E-169	0.1042	0.0061	1.002	0.0092	0.488	0.0089	ADHD_mine	TRUE	Psychiatric								
1710177475398ANforCTG.txt.gz.checked.sumstats.gz	1710174270056F5forCTG.txt.gz.checked.sumstats.gz	9.00E-04	0.036229	0.024219	0.98068	0.2711	0.0214	1.033	0.0129	0.0244	0.0085	AN_mine	FALSE									
1710177601094ANXforCTG.txt.gz.checked.sumstats.gz	1710174270056F5forCTG.txt.gz.checked.sumstats.gz	0.3009	0.042327	7.1077	1.18E-12	0.0455	0.0036	1.015	0.0126	-0.0034	0.0094	ANX_mine	TRUE	Psychiatric								
1710177690641AUTforCTG.txt.gz.checked.sumstats.gz	1710174270056F5forCTG.txt.gz.checked.sumstats.gz	0.1646	0.051181	3.2168	0.001296	0.1899	0.0187	1.012	0.0114	0.1393	0.0088	AUT_mine	FALSE									
1710177786024BIPforCTG.txt.gz.checked.sumstats.gz	1710174270056F5forCTG.txt.gz.checked.sumstats.gz	0.1653	0.033344	4.9577	7.13E-07	0.2707	0.0131	1.057	0.0158	0.0195	0.0109	BIP_mine	TRUE	Psychiatric								
1710177876789DYXforCTG.txt.gz.checked.sumstats.gz	1710174270056F5forCTG.txt.gz.checked.sumstats.gz	0.9471	0.003964	238.89	0	0.2561	0.0121	0.87	0.0158	0.7996	0.0132	DYX_mine	TRUE	Psychiatric								
1710177974233MDDforCTG.txt.gz.checked.sumstats.gz	1710174270056F5forCTG.txt.gz.checked.sumstats.gz	0.2867	0.027839	10.3	7.06E-25	0.0676	0.0034	1.015	0.017	0.0325	0.0095	MDD_mine	TRUE	Psychiatric								
1710178067898OCDforCTG.txt.gz.checked.sumstats.gz	1710174270056F5forCTG.txt.gz.checked.sumstats.gz	-0.1563	0.065907	-2.372	0.017694	0.4223	0.0811	0.995	0.0097	-0.0018	0.0078	OCD_mine	FALSE									
1710178156969SCZforCTG.txt.gz.checked.sumstats.gz	1710174270056F5forCTG.txt.gz.checked.sumstats.gz	0.1299	0.026567	4.8881	1.02E-06	0.7925	0.0335	1.098	0.0229	0.0102	0.0115	SCZ_mine	TRUE	Psychiatric								
1710178251076TSforCTG.txt.gz.checked.sumstats.gz	1710174270056F5forCTG.txt.gz.checked.sumstats.gz	0.026	0.051883	0.50045	0.61676	0.4293	0.0531	1.01	0.0105	0.0056	0.008	TS_mine	FALSE									
