# Supplementary material for: Genetic neurodevelopmental clustering and dyslexia
Source: Mol Psychiatry. 2024 Jul 15;30(1):140–50. doi: 10.1038/s41380-024-02649-8 (PMC11649571; doi:10.1038/s41380-024-02649-8)
Supplement: Supplementary file 4 — Supplementary Table 4 [file 41380_2024_2649_MOESM4_ESM.pdf]

Supplementary Table 4. Genomic risk loci that associate with both ADHD and dyslexia. Loci in bold were previously identified in the dyslexia GWAS, loci in red were previously identified in the ADHD GWAS.

														GWAS Catalog associations in locus		
**GenomicLocus**	**uniqID**	**rsID**	**chr**	**pos**	**p**	**start**	**end**	**nSNPs**	**nGWASSNPs**	**nIndSigSNPs**	**IndSigSNPs**	**nLeadSNPs**	**LeadSNPs**	**Primary**	**Related**	**Other**
1	1:43782846:A:G	rs1198982	1	43782846	3.668E-07	43759733	43949718	136	105	7	rs1198982;rs1199039;rs7527092;rs2282225;rs6694318;rs3120276;rs2991990	1	rs1198982	Attention deficit hyperactivity disorder or autism spectrum disorder or intelligence (pleiotropy); Attention deficit hyperactivity disorder (MTAG); Anorexia nervosa, attention-deficit/hyperactivity disorder, autism spectrum disorder, bipolar disorder, major depression, obsessive-compulsive disorder, schizophrenia, or Tourette syndrome (pleiotropy)	Brain region volumes; Whole brain restricted isotropic diffusion (multivariate analysis); Brain morphology (MOSTest)	IDP dMRI ProbtrackX FA ar r; White blood cell count; Hematocrit; Hemoglobin concentration; Blood protein levels; Serum levels of protein TIE1; Systolic blood pressure; Insulin-like growth factor 1 levels; Diastolic blood pressure; Hypertension; Monocyte count; Mean arterial pressure; Height
2	2:22466025:A:C	rs7559944	2	22466025	8.43E-06	22430795	22606275	206	153	8	rs7559944;rs4300861;rs57537843;rs12620364;rs2339515;rs12614708;rs1995812;rs4416248	1	rs7559944	Attention deficit hyperactivity disorder (MTAG); General cognitive ability; Educational attainment; Externalizing behaviour (multivariate analysis); Smoking variables; Risk-taking behaviours	Depression/depressive symptoms; Wellbeing variables; Insomnia, Neuroticism; Schizophrenia; Neuropsychiatric disorders; Bipolar; PTSD; Trauma exposure	Type 2 diabetes; Systemic lupus erythematosus; Docosapentaenoate (n3 DPA; 22:5n3) levels in elite athletes; Biological sex; Low density lipoprotein cholesterol levels
3	2:99511774:G:T	rs10178956	2	99511774	2.064E-05	99381405	100141669	355	235	71	rs10178956;rs12475639;rs12464785;rs17022564;rs57242893;rs55815229;rs62153855;rs58086269;rs6713608;rs2309576;rs2309617;rs3791212;rs7589365;rs7602923;rs4851204;rs7606977;rs7597045;rs7558062;rs59178618;rs12052986;rs11884686;rs2290255;rs1584648;rs1584647;rs7584127;rs6732750;rs11892441;rs61103312;rs17022629;rs7593274;rs58958283;rs2290257;rs6711073;rs2290261;rs1053544;rs717454;rs7560996;rs3749087;rs28382946;rs7597141;rs3792144;rs3792142;rs57977991;rs3792137;rs7563455;rs7563462;rs7572779;rs10865031;rs11899745;rs1839667;rs2122748;rs4851206;rs2053917;rs12465846;rs60257640;rs7585019;rs769105;rs4476396;rs62155809;rs1973011;rs896249;rs6542881;rs2309604;rs7564587;rs1011633;rs6737560;rs4143760;rs1839666;rs4851208;rs6761390;rs6715321	1	rs10178956	Self-reported math ability (MTAG); Cognitive performance (MTAG); Educational attainment	Bipolar disorder; Fear of minor pain	Eosinophil counts; Idiopathic inflammatory myopathy; Heel bone mineral density; Uterine fibroids
4	2:140473877:C:T	rs4954797	2	140473877	1.017E-05	140067949	140476040	156	111	17	rs4954797;rs36051104;rs7597242;rs13025522;rs12053077;rs62172365;rs34150606;rs35835668;rs34147551;rs7596468;rs62172389;rs17783971;rs2164855;rs62172411;rs1837160;rs9646701;rs10928723	1	rs4954797	Educational attainment; Adventurousness; General risk tolerance (MTAG)		
5	2:179017549:G:T	rs334051	2	179017549	7.937E-05	178979137	179092195	54	33	3	rs334051;rs1900251;rs1374257	1	rs334051			
6	2:191504467:C:G	rs6706715	2	191504467	7.335E-06	191503641	191710069	141	103	37	rs6706715;rs13004849;rs6719351;rs6737278;rs12693582;rs13382604;rs10202642;rs13010534;rs11684257;rs3821235;rs1989344;rs6708151;rs2109959;rs1990461;rs10200534;rs34446204;rs6716985;rs6434410;rs6744503;rs2192011;rs4458238;rs66886317;rs35295960;rs6434411;rs16832848;rs7563403;rs10153559;rs6721964;rs7602459;rs7590384;rs887697;rs887696;rs1465302;rs13022570;rs10931469;rs1263145;rs72917135	1	rs6706715	Educational attainment	Morningness; Chronotype; Longevity	Systolic blood pressure; Generalized epilepsy

7	2:198841329:C:T	rs7571545	2	198841329	1.584E-07	198146381	198954774	593	436	151	rs7571545;rs11891555;rs6434949;rs6724526;rs893808;rs1976772;rs6748683;rs62277902;rs976180;rs1813106;rs1836234;rs12693829;rs11689393;rs116165148;rs2342557;rs1978889;rs7592556;rs10207232;rs10184395;rs6715070;rs1371663;rs1147169;rs10190226;rs2196176;rs892514;rs882954;rs5007118;rs10497812;rs6732517;rs4850441;rs2164071;rs7420608;rs11895260;rs11903129;rs2342558;rs13382697;rs1837495;rs35563720;rs7600269;rs11889006;rs7590828;rs1371665;rs2033570;rs1979239;rs2045242;rs10497806;rs10166328;rs7564924;rs1598469;rs10203581;rs55656916;rs10931787;rs1902249;rs13000656;rs13404366;rs67492914;rs6737060;rs6708239;rs6741205;rs6741314;rs13394214;rs11690163;rs61155920;rs6757669;rs10931788;rs9967823;rs6719832;rs7600862;rs4850436;rs34632716;rs13018267;rs10460394;rs10931791;rs4850807;rs10931793;rs4850809;rs2037590;rs700638;rs700642;rs700643;rs700645;rs700646;rs1068682;rs700651;rs700656;rs700658;rs700660;rs700662;rs770659;rs700674;rs7604700;rs1435569;rs700686;rs700687;rs696817;rs700688;rs770661;rs700690;rs700692;rs770662;rs1436131;rs1435570;rs11899188;rs4850812;rs9288280;rs734037;rs13395030;rs2060488;rs7561950;rs2043545;rs1865586;rs6734781;rs6434943;rs1435568;rs6729473;rs6760891;rs6706937;rs7572123;rs10211202;rs4850813;rs10931794;rs2341778;rs1401095;rs1589162;rs13026569;rs4850815;rs12619300;rs1401091;rs1401092;rs7557203;rs1464211;rs1607375;rs1607374;rs4369854;rs1983359;rs11887138;rs1518367;rs1518365;rs1518363;rs1518361;rs1464209;rs11890137;rs35730302;rs58224025;rs1401096;rs13408411;rs1850631;rs6434948;rs6752458;rs1356542;rs1518368	1	rs7571545	Educational attainment; General cognitive ability; Cognitive ability, years of educational attainment or schizophrenia (pleiotropy), Anorexia nervosa, attention-deficit/hyperactivity disorder, autism spectrum disorder, bipolar disorder, major depression, obsessive-compulsive disorder, schizophrenia, or Tourette syndrome (pleiotropy)	Autism spectrum disorder or schizophrenia; Schizophrenia; Bipolar; Cortical Thickness; Sulcal depth; Depression; Alcohol dependence/consumption; Schizophrenia vs anorexia nervosa (ordinary least squares (OLS)); Whole brain free water diffusion (multivariate analysis); Brain morphology (MOSTest); Cortical surface area; Major depression and alcohol dependence' Insomnia; Whole brain restricted diffusion variables; Chronotype variables; Bipolar disorder or major depressive disorder	Metabolic biomarkers (multivariate analysis); Body fat %; Intracranial, abdominal aortic or thoracic aortic aneurysm (pleiotropy); Nonsyndromic orofacial cleft x sex interaction; Platelet count; Hay fever and/or eczema; Rheumatoid arthritis; BMI and weight measures; Red blood cell count; Asthma; Electrocardiogram morphology (amplitude at temporal datapoints); Total body bone mineral density; Eczema; Allergy; Crohn's Disease; Systemic lupus erythematosus; Low high density lipoprotein cholesterol levels; Dermatomyositis; Mean corpuscular hemoglobin
8	3:20480777:A:G	rs7652099	3	20480777	3.195E-05	20428079	20605021	190	151	10	rs7652099;rs4857954;rs4858204;rs1356795;rs35672661;rs11128956;rs4282078;rs1829499;rs9310619;rs7640520	1	rs7652099	Educational attainment; Highest math class taken	Schizophrenia, bipolar disorder or major depressive disorder; Insomnia	BMI; body size
9	3:37116386:G:T	rs2302504	3	37116386	0.0001051	36951898	37457448	481	356	1	rs2302504	1	rs2302504		Vertex-wise sulcal depth; Cortical surface areal Vertex-wise cortical surface area; Brain morphology (MOSTest); Cortical Thickness	Iris color (L* coordinate); Mean platelet volume; Hypothyroidism; High density lipoprotein cholesterol levels; Pulse pressure; HDL cholesterol; Triglycerides; C-reactive protein levels; Gamma glutamyl transpeptidase; Alanine aminotransferase levels; Aspartate aminotransferase to alanine aminotransferase ratio; Liver enzyme levels (gamma-glutamyl transferase); height; Adolescent idiopathic scoliosis; Cerebrospinal P-tau181p levels; Medication use (thyroid preparations)
10	3:50210289:C:G	rs1005678	3	50210289	2.157E-08	48719638	50399695	855	451	55	rs4364202;rs4279134;rs6779394;rs6770112;rs6784111;rs4955410;rs12493001;rs12636030;rs12487580;rs36133651;rs7632267;rs7645551;rs61583136;rs12493284;rs3774799;rs11720964;rs4554002;rs7640903;rs11709788;rs11920251;rs1865741;rs34142492;rs12497569;rs6795772;rs4955431;rs9682444;rs9311433;rs4974084;rs4521268;rs4513485;rs3212;rs1005678;rs2624833;rs13059311;rs9858297;rs13067082;rs1046956;rs12632110;rs12637671;rs14321;rs11919418;rs13064381;rs6807194;rs35137368;rs2236940;rs2236941;rs6800021;rs2526389;rs1046953;rs2188151;rs2624839;rs2624838;rs2518796;rs2518795;rs2624835	2	rs4364202;rs1005678	Attention deficit hyperactivity disorder or autism spectrum disorder or intelligence (pleiotropy); Cognitive ability/performance/intelligence; Common executive function; Education attainment; Disruptive behaviour; Highest math class taken	Externalising Behaviours; Risk-taking behaviour variables; Smoking variables; Alzheimer's disease; Brain morphology/structural/diffusion variables; Diet variables; Depressive symptoms; Household income; Insomnia; Mood; Leisure screen time/sedentary behaviour; Life satisfaction; Chronotype; Neuroticism; Noncognitive aspects of educational attainment; Opioid use disorder (MTAG); Occupational attainment; Refractive error;	BMI and related variables; Adiponectin levels; Apolipoprotein A1 levels; Asthmal Behcet's disease; Childhood ear infection; C-reactive protein levels; Eosinophil counts; Frailty index; Gastroesophageal reflux disease, peptic ulcer disease and/or corresponding medications and treatment; Glycated hemoglobin levels; Glycine levels; Cholesterol; Hepatocyte growth factor-like protein levels; Mean corpuscular hemoglobin; Mean corpuscular volume; Mean platelet count; Medication use; Menarche (age onset); Metabolic markers, Mitochondrial DNA copy number; Monocyte count; Multisite chronic pain; Neutrophil count; Non-melanoma skin cancer; Osteoarthritis; Parental longevity; Parkinson's Disease; Red cell distribution width; Serum alkaline phosphatase levels; Sex hormone-binding globulin levels; Sunburns; Thioredoxin domain-containing protein 12 levels; Triglycerides; Type

															[illegible] levels; Triglycerides; Type 2 diabetes; Walking pace; White blood cell count; Youthful appearance
11	3:84517716:C:G	rs79273178	3	84517716	3.517E-05	83631491	84728016	131	91	1	rs79273178	1	rs79273178		Risk-taking behavior (multivariate analysis)	Response to selenium supplementation (change in plasma selenium concentration); Predicted visceral adipose tissue; Adult body size; BMI; Lead levels in blood
**12**	**3:85653460:A:G**	**rs10511073**	**3**	**85653460**	**6.638E-08**	**85396592**	**85958954**	**890**	**666**	**427**	**rs10511073;rs62263914;rs1375564;rs73141547;rs4856600;rs1375561;rs12637027;rs7632056;rs1449400;rs1449398;rs11**	**1**	**rs10511073**	**Cognitive function/performance; Educational attainment; Highest**	**Risk taking behaviours; Smoking, cannabis and alcohol variables;**	**BMI and related variables; Dietary preferences; Core binding factor**
13	3:117535656:C:T	rs4688050	3	117535656	6.86E-06	117459549	117623116	18	10	1	rs4688050	1	rs4688050		Insomnia	
14	3:123594419:G:T	rs34067381	3	123594419	8.649E-05	123524036	123745050	91	57	1	rs34067381	1	rs34067381	Educational attainment; General cognitive ability; Intelligence; High math class taken	Household income	
**15**	**3:135668716:A:G**	**rs1403767**	**3**	**135668716**	**8.316E-11**	**135619585**	**136752590**	**764**	**534**	**77**	**rs1403767;rs73230099;rs1052620;rs9867325;rs9840500;rs9852406;rs56308637;rs28875187;rs9854084;rs1394092;rs12637102;rs12489577;rs699165;rs711976;rs1070230;rs1070232;rs833752;rs4678433;rs7621025;rs6439646;rs6794351;rs12635723;rs12630999;rs4452981;rs1471740;rs1017881;rs4456810;rs61791757;rs6779146;rs61789561;rs1154988;rs645040;rs687339;rs511154;rs1153871;rs61789601;rs895893;rs684773;rs534944;rs1278719;rs523118;rs3755636;rs548288;rs632016;rs866782;rs606725;rs4678428;rs576771;rs665200;rs7628146;rs7653249;rs1279840;rs17252498;rs1711171;rs1618069;rs1291921;rs483465;rs610860;rs696517;rs696516;rs1145101;rs667920;rs571513;rs33999043;rs655720;rs698270;rs1145106;rs1964675;rs28478252;rs2655008;rs6779727;rs7644541;rs6789488;rs9867368;rs11715257;rs9861150;rs6439640**	**2**	**rs1403767;rs9854084**	**Non-word reading; Attention deficit hyperactivity disorder or autism spectrum disorder or intelligence (pleiotropy); Cognitive performance/general cognitive ability/intelligence; Cognitive aspects of Education attainment;**	**Brain variables; Smoking variables; Neuroticism; Schizophrenia; Sleep duration;**	**Body size/weight variables; Age at Menopause; Alanine aminotransferase levels; Apolipoprotein A1 levels; Apolipoprotein B levels; Aspartate aminotransferase levels; B-cell malignancies (chronic lymphocytic leukemia, Hodgkin lymphoma or multiple myeloma) (pleiotropy); testosterone levels; Blood urea nitrogen levels; Calcium leveles; CVD; Central corneal thickness; Cholesterol levels; Concentration of chylomicrons and VLDL particles; C-reactive protein; Direct bilirubin levels; Dyslipidemia; Cystatic c; Fibrinogen; Gamma glutamyl transferase levels; Glycine levels; Insulin-like growth factor 1 levels; Intraocular pressure; Dietary preferences; Leucine levels; Liver enzyme levels; Male-pattern baldness; Medication use; Metabolic biomarkers; Lymphocyte percentage of white cells; Monocyte count; Monounsaturated fatty acid levels and ratios; Multisite chronic pain; Neutrophil percentage of white cells; Phospholipid levels; Platelet count; Red cell distribution width; Serum alkaline phosphatase levels; Serum creatinine levels; Sex hormone-binding globulin levels; Systolic blood pressure; Total bilirubin levels; Triglycerides; Valine levels; Walking pace; Type 2 Diabetes**
16	3:138154795:A:G	rs1109088	3	138154795	1.968E-05	138059966	138347164	183	119	2	rs1109088;rs7609893	1	rs1109088	Self-reported math ability	Chronotype; Morningness	Total testosterone levels; Red cell distribution width; Bioavailable testosterone levels; Waist-to-hip ratio adjusted for BMI
17	3:197032224:C:G	rs338222	3	197032224	2.376E-06	196788438	197057406	100	68	3	rs338222;rs395572;rs338217	1	rs338222		Vertex-wise sulcal depth	Cleft lip with or without cleft palate or cleft palate (pleiotropy); Heel bone mineral density; Estimated glomerular filtration rate (creatinine); Lung function (FVC); acne vulgaris; Urinary Tract Infection frequency
18	4:91732183:C:T	rs7671307	4	91732183	1.354E-05	91624515	91783081	191	133	2	rs7671307;rs7693414	1	rs7671307			Gut microbiome composition (summer); Theophylline levels in elite athletes; 5-acetylamino-6-amino-3-methyluracil levels in elite athletes
19	4:130923486:A:G	rs2011980	4	130923486	7.708E-05	130724740	130950008	390	207	1	rs2011980	1	rs2011980		Insomnia; Chronotype	BMI/body size/obesity; Type 2 Diabetes
20	5:92316380:C:T	rs34508	5	92316380	9.556E-06	92255166	92493318	46	30	3	rs34508;rs34517;rs345909	1	rs34508	Autism spectrum disorder	Brain rsFMRI measurement (functional connectivity); rfMRI connectivity ICA-features 2; Smoking initiation	Total PHF-tau (SNP x SNP interaction); Essential tremor; Obesity related traits

21	6:98518518:C:T	rs9375138	6	98518518	3.762E-05	98310091	98785796	247	191	1	rs9375138	1	rs9375138	Attention deficit hyperactivity disorder or autism spectrum disorder or intelligence (pleiotropy); General cognitive ability/intelligence; Educational attainment	Externalizing behaviour; Neuroticism traits; Number of sexual partners	Body mass index; Diastolic blood pressure
22	7:11501136:C:T	rs7803385	7	11501136	2.849E-05	11475819	11505293	21	18	6	rs7803385;rs12055997;rs7807369;rs11979605;rs17164600;rs118134876	1	rs7803385	General cognitive ability/cognitive performance; Educational attainment; Math ability	Household income (MTAG)	Champagne or white wine consumption (glasses per month) (UKB data field 1578, 4418)
**23**	**7:69896122:C:T**	**rs73175930**	**7**	**69896122**	**7.164E-06**	**68914449**	**69896122**	**387**	**284**	**44**	**rs73175930;rs6960938;rs10242297;rs60417126;rs56134694;rs10261510;rs59342262;rs55901635;rs10241446;rs28524600;rs7357113;rs10256311;rs12334231;rs12334234;rs7787342;rs73155227;rs17140663;rs10215282;rs11972124;rs11972857;rs11764821;rs62454937;rs62454938;rs55693719;rs10238487;rs7797529;rs7798800;rs7798996;rs11764559;rs9886324;rs7797273;rs61171607;rs56107513;rs73175903;rs73175910;rs118084667;rs73175914;rs73175915;rs7795355;rs73175921;rs143039974;rs11767563;rs6946732;rs73175928**	**1**	**rs73175930**			**Medication use (agents acting on the renin-angiotensin system); Hand grip strength**
24	7:104605530:A:C	rs7776707	7	104605530	2.52E-05	104497591	105069629	421	212	26	rs7776707;rs10238507;rs10266871;rs6943183;rs2299308;rs3779210;rs4730072;rs10281886;rs3801285;rs2240463;rs10281422;rs41562;rs917114;rs7801804;rs7811681;rs6466055;rs6466056;rs12705304;rs2057883;rs2057884;rs9649275;rs113905912;rs56016333;rs13237211;rs4727614;rs6955349	1	rs7776707	Attention deficit hyperactivity disorder or autism spectrum disorder or intelligence (pleiotropy); Anorexia nervosa, attention-deficit/hyperactivity disorder, autism spectrum disorder, bipolar disorder, major depression, obsessive-compulsive disorder, schizophrenia, or Tourette syndrome (pleiotropy); Cognitive traits (MTAG)/general cognitive ability; Common executive function; Math ability; Educational attainment; Cognitive aspects of educational attainment	Schizophrenia; Household income; Insomnia; General risk tolerance; Autism spectrum disorder or schizophrenia; Schizophrenia vs anorexia nervosa (ordinary least squares (OLS))	Lung function (FVC); Serum 25-Hydroxyvitamin D levels; Body size variables; Sunburns; Creatinine; Advanced age-related macular degeneration; Melanoma; Biological sex; Youthful appearance (self-reported); Smoking cessation; Drinks usually with meals in current drinkers (yes vs no) (UKB data field 1618)
25	8:34225030:A:G	rs117396993	8	34225030	4.015E-06	33292416	35161035	225	151	8	rs117396993;rs80318442;rs74427054;rs76013678;rs146649743;rs75836205;rs6990255;rs118041269	1	rs117396993	Attention deficit hyperactivity disorder; Attention deficit hyperactivity disorder or autism spectrum disorder or intelligence (pleiotropy); Cognitive ability, years of educational attainment or schizophrenia (pleiotropy); Attention deficit hyperactivity disorder or cannabis use; Autism spectrum disorder, attention deficit-hyperactivity disorder, bipolar disorder, major depressive disorder, and schizophrenia (combined); Anorexia nervosa, attention-deficit/hyperactivity disorder, autism spectrum disorder, bipolar disorder, major depression, obsessive-compulsive disorder, schizophrenia, or Tourette syndrome (pleiotropy); Major depressive disorder vs ADHD (ordinary least squares (OLS))	Schizophrenia; Bipolar Disorder; Insomnia	

26	9:15641978:A:G	rs1848582	9	15641978	9.68E-07	15529852	16053793	904	514	162	rs1848582;rs7042475;rs10810419;rs7031365;rs10962106;rs13301516;rs7851056;rs10123042;rs7046351;rs7030846;rs7034484;rs10810424;rs6474946;rs10118359;rs10121591;rs10810426;rs1355171;rs7035863;rs10810427;rs10121393;rs28403566;rs954663;rs954664;rs10962118;rs10756691;rs35057009;rs12352779;rs10810429;rs7037662;rs7037908;rs62573118;rs10962121;rs28785887;rs10810430;rs12004358;rs10962123;rs11793900;rs6474951;rs10119975;rs28410434;rs7036285;rs6474952;rs36086644;rs2175080;rs12349323;rs10962125;rs10962126;rs1396706;rs7028999;rs7860869;rs12377371;rs10810438;rs7860024;rs12341679;rs7467156;rs7467207;rs6474962;rs6474963;rs6474964;rs6474965;rs7025420;rs36013000;rs7034781;rs10810440;rs62571268;rs1355173;rs7869624;rs6474967;rs7872912;rs7036775;rs1341734;rs2096121;rs7019143;rs7019323;rs7019851;rs10962147;rs13283620;rs58517712;rs7018872;rs10962152;rs1539171;rs1970497;rs10962153;rs12378499;rs13287834;rs10810446;rs2185663;rs7861802;rs2382540;rs1539172;rs1539173;rs1341736;rs11521158;rs11515218;rs56401042;rs1341737;rs1341738;rs7866894;rs10962169;rs9406534;rs13296360;rs4146293;rs7849380;rs7866641;rs10962170;rs10962171;rs9406535;rs9407645;rs7857495;rs7871232;rs7857601;rs2382553;rs7468344;rs7470881;rs9406536;rs9406537;rs76680143;rs9406539;rs7019205;rs9406540;rs9407647;rs1572978;rs1009468;rs9407648;rs9407649;rs7032457;rs7047045;rs7032634;rs9406541;rs9407650;rs9407651;rs9407652;rs9406542;rs7024440;rs2185665;rs2153725;rs2153726;rs9407653;rs9407654;rs2153727;rs7857126;rs7853744;rs9407655;rs9407656;rs9298738;rs9298739;rs9407658;rs9298740;rs9298741;rs2457637;rs4740614;rs7873152;rs6474930;rs2821545;rs9407624;rs7875367;rs276453;rs276449;rs276447;rs276444;rs2457265;rs11792937	1	rs1848582	Educational attainment	General risk tolerance; Cortical thickness; Vertex-wise sulcal depth; Vertical cup-disc ratio	Acceptance of an invitation to participate in a mental health questionnaire; Body size and related weight variables; Blood urea nitrogen levels; Dietary preferences; C-reactive protein; Metabolic biomarkers; Parental longevity; Dietary intake variables
27	9:86753395:G:T	rs1246268	9	86753395	1.514E-06	86727865	86766265	45	28	7	rs1246268;rs1030856;rs1246265;rs1246264;rs1246263;rs1246292;rs2799849	1	rs1246268		Risk-taking tendency (4-domain principal component model); Smoking variables; Cannabis smoking or cigarette smoking or schizophrenia	Biological sex; Skeletal age; Cereal consumption (bowls per week) (UKB data field 1458)
28	9:96352243:A:T	rs10992792	9	96352243	2.197E-05	96120842	96481360	319	250	60	rs10992792;rs10821168;rs10821140;rs10992756;rs10992757;rs4743924;rs4744247;rs1556416;rs2150749;rs4744249;rs10992759;rs10512223;rs10821141;rs2297376;rs10821142;rs10821143;rs16909199;rs10821145;rs1412050;rs10821146;rs10821147;rs4744250;rs4744251;rs4744252;rs882851;rs10992768;rs10992769;rs4744253;rs2183760;rs4743928;rs10992770;rs10761231;rs10821148;rs12377707;rs13285064;rs4744254;rs10992772;rs10821151;rs11790449;rs10739950;rs10761232;rs10761233;rs10821152;rs4307405;rs3813388;rs3813387;rs10821153;rs34883241;rs36016074;rs12380167;rs10821154;rs10992779;rs10992780;rs10821157;rs10821158;rs10992781;rs10761235;rs564;rs10992790;rs10761238	1	rs10992792	Cognitive aspects of educational attainment; Cognitive performance; Educational attainment	Age at first sexual intercourse; Depressive symptoms; Generalized anxiety disorder (mental health questionnaire or predicted); Insomnia; Neuroticism; PTSD	Ability to confide in someone; Body size and mass variables; Platelet count; Multisite chronic pain; Lung function; Irritable bowel syndrome; Itch intensity from mosquito bite; Coronary artery disease in hypertension
29	9:122596593:A:C	rs944956	9	122596593	9.721E-05	122548040	122619905	93	76	3	rs944956;rs1333859;rs1333915	1	rs944956	Self-reported math ability	Major depressive disorder	
30	10:106559287:C:T	rs11599313	10	106559287	1.034E-06	106451870	106832251	395	312	92	rs11599313;rs2451483;rs2496011;rs76105184;rs80119540;rs2451468;rs79910196;rs77201367;rs2491380;rs2451493;rs78443007;rs7904927;rs7901771;rs6584638;rs6584642;rs59923784;rs57709811;rs59822520;rs73342180;rs57695054;rs7073896;rs7078084;rs7083880;rs11812127;rs59262096;rs7088485;rs58578339;rs7090127;rs150220347;rs7082914;rs59715667;rs73351631;rs73351633;rs73351634;rs60083048;rs73351635;rs73351636;rs73351639;rs73351644;rs73351645;rs73351648;rs73351649;rs73351650;rs78161238;rs73351651;rs73334023;rs76828960;rs73334025;rs73334026;rs73334027;rs80126900;rs73334028;rs113602308;rs73334032;rs73334034;rs73334037;rs73334042;rs61372005;rs73334043;rs73334045;rs73334048;rs73334050;rs73334052;rs73334054;rs73334057;rs73334060;rs73334065;rs73334066;rs73334068;rs73334069;rs73334073;rs73334074;rs1997066;rs1997065;rs1997064;rs73335942;rs4350297;rs11594644;rs61867284;rs61867285;rs74317294;rs79883993;rs61867287;rs17185757;rs17766570;rs11593333;rs11593349;rs61867290;rs113316302;rs11596241;rs11596250;rs17186548	1	rs11599313	Attention deficit hyperactivity disorder; Attention deficit hyperactivity disorder or autism spectrum disorder or intelligence (pleiotropy); Anorexia nervosa, attention-deficit/hyperactivity disorder, autism spectrum disorder, bipolar disorder, major depression, obsessive-compulsive disorder, schizophrenia, or Tourette syndrome (pleiotropy); Educational attainment; Math ability; Neuropsychiatric disorders	Autism and major depressive disorder (MTAG); Autism spectrum disorder (MTAG); Bipolar disorder or major depressive disorder; Depression/Depressive symptoms; Insomnia; Well being; Schizophrenia; Neuroticism	Cheese consumption; Diabetic kidney disease; Metabolite levels; Non-alcoholic fatty liver disease activity score in non-alcoholic fatty liver disease

31	11:28694440:C:G	rs10835391	11	28694440	3.287E-08	28591168	28710118	197	161	10	rs10835391;rs7940384;rs11030410;rs1317526;rs7944200;rs10767742;rs10767744;rs4922808;rs10767746;rs4414202	1	rs10835391	Attention deficit hyperactivity disorder; Attention deficit hyperactivity disorder or autism spectrum disorder or intelligence (pleiotropy); Cognitive performance/general cognitive ability; Educational attainment; Highest math class taken (MTAG)	Age at first sexual intercourse/number of partners; Smoking variables; Alcohol consumption; Disruptive Behaviour; Externalising behaviour; Risky behaviours; depression; Neuroticismt items; Neuropsychiatric disorders; Schizophrenia; Refractive error	BMI and related variables; Intraocular pressure
32	11:113749973:A:C	rs12289874	11	113749973	6.84E-05	113494864	113786539	17	15	1	rs12289874	1	rs12289874			
33	12:49398862:A:G	rs2293445	12	49398862	3.227E-05	49389320	49479968	55	37	14	rs2293445;rs10875908;rs1138908;rs10783300;rs2117029;rs2293446;rs10875913;rs11168827;rs3782357;rs10875915;rs10783301;rs2241726;rs11168839;rs10783299	1	rs2293445	Attention deficit hyperactivity disorder or autism spectrum disorder or intelligence (pleiotropy); Bipolar disorder vs ADHD (ordinary least squares (OLS)); Cognitive ability/perfromance/general cognitive ability/intelligence; Education attainment; Cognitive aspects of educational attainment; Cognitive ability, years of educational attainment or schizophrenia (pleiotropy); Reaction time; Math ability; Verbal-numerical reasoning	Bipolar disorder	Hair color; HDL cholesterol levels in HIV infection; Lumbar spine bone mineral density; Menarche (age at onset); Percentage gas trapping; Serum total protein level
34	12:123710835:A:C	rs1626703	12	123710835	5.666E-05	123447928	123916097	439	256	2	rs1626703;rs1727295	1	rs1626703	Attention deficit hyperactivity disorder or autism spectrum disorder or intelligence (pleiotropy); Anorexia nervosa, attention-deficit/hyperactivity disorder, autism spectrum disorder, bipolar disorder, major depression, obsessive-compulsive disorder, schizophrenia, or Tourette syndrome (pleiotropy); Alzheimer's disease or educational attainment (pleiotropy); Cognitive ability, years of educational attainment or schizophrenia (pleiotropy); Cognitive ability/performance/general cognitive ability/intelligence; Educational attainment; Math ability;	Autism spectrum disorder or schizophrenia; Brain morphology, volumes, diffusion; Household income; Insomnia; Schizophrenia/vs ADHD, AN, ASD, TS; Tourette syndrome	Body shape and weight measures; Age at menopause; Alanine aminotransferase levels; Albumin-globulin ratio; Allergy; Asthma; Biological sex; Blood protein levels; Cholesterol variables; Hair colour; Hayfever/eczema; Heel bone mineral density; Diastolic blood pressure; Early-onset ischemic stroke; Eosinophil counts; High light scatter reticulocyte count; Knee osteoarthritis; Lung function; Lymphocyte count; Macular thickness; Platelet volume; Multiple Sclerosis; Serum albumin levels; Triglyceride levels; Type 2 Diabetes; Uterine fibroids; Venous thromboembolism
35	14:58838668:A:G	rs1051860	14	58838668	3.917E-05	58643368	58894577	183	119	14	rs1051860;rs1051861;rs28398380;rs1957944;rs2147609;rs2348075;rs6573194;rs12892257;rs10151752;rs7147757;rs28637941;rs2145598;rs58231973;rs10137475	1	rs1051860	Educational attainment; Highest math class taken (MTAG)	Age at first sexual intercourse; Alcohol use variables; Externalising behaviour; Smoking variables; Risk-taking behaviours	Coronary artery disease; Mean corpuscular hemoglobin; Platelet volume; Migraine; Non-melanoma skin cancer; Red blood cell count; Type 2 Diabetes; Venous thromboembolism; Waist-hip ratio
36	14:98643863:A:G	rs77653640	14	98643863	1.506E-07	98530190	98670849	140	113	110	rs77653640;rs17701905;rs17701958;rs2008260;rs17775184;rs79470265;rs7144406;rs7141014;rs12435486;rs17692512;rs1537127;rs10484133;rs10137240;rs17698383;rs79291406;rs75243882;rs734952;rs2004669;rs2004670;rs751441;rs17698510;rs751440;rs751439;rs17698580;rs10145335;rs113232967;rs10484131;rs17771980;rs17698811;rs1381285;rs1824578;rs12050206;rs78923584;rs17699117;rs17772412;rs77052509;rs17772567;rs17772697;rs76675781;rs28856685;rs17699522;rs76115697;rs8008425;rs8007501;rs8019512;rs1461576;rs1461575;rs1381272;rs17096452;rs79374765;rs17773233;rs965770;rs79504488;rs77556698;rs7152970;rs8020355;rs7160386;rs78154752;rs76659130;rs1381286;rs79310499;rs79108591;rs12050469;rs1461587;rs7160893;rs7161307;rs8013699;rs10133228;rs28415454;rs12147808;rs10147461;rs8006659;rs10143433;rs12323602;rs10151485;rs17701424;rs76247422;rs10151901;rs8009025;rs8007838;rs17701568;rs75791745;rs76905624;rs10139183;rs10141336;rs28488824;rs28691243;rs17096587;rs72478863;rs12100628;rs7155953;rs7154993;rs7159827;rs7159904;rs7160371;rs7160112;rs7160439;rs7160630;rs1461583;rs1461584;rs7145698;rs17096600;rs17096602;rs17701821;rs7145913;rs7146746;rs10144091;rs76381532;rs7159336;rs7159716	1	rs77653640	Cognitive performance/general cognitive ability/Intelligence; Verbal-numerical reasoning; Cognitive aspects of educational attainment; Math ability; Attention deficit hyperactivity disorder or autism spectrum disorder or intelligence (pleiotropy)	Vertex-wise sulcal depth/cortical thickness/surface area; Age at first intercourse; Insomnia	Lymphocyte count; Medication use (calcium channel blockers); Systolic blood pressure; Biological sex; Pulse pressure; Staphylococcus aureus infection; Systolic blood pressure
37	15:33063140:C:T	rs75606001	15	33063140	2.285E-05	33035284	33079610	28	17	4	rs75606001;rs8031239;rs79070500;rs75406947	1	rs75606001			Facial skin gloss

38	15:57354415:A:G	rs2703577	15	57354415	7.232E-06	56825370	57622926	614	310	18	rs2703577;rs12900874;rs2733170;rs2920264;rs2470079;rs2585088;rs1820995;rs2435907;rs2951901;rs2957574;rs2470082;rs2920265;rs2951906;rs2439918;rs2962992;rs2464429;rs2962990;rs2703580	1	rs2703577	Educational attainment	Insomnia; Predicted developmental stuttering; Vertex-wise sulcal depth	Hand grip strength; Intraocular pressure; Biological sex; Testosterone levels; UTI frequency; Cholesterol; Type 2 Diabetes; BMI; Glaucoma
39	15:81021182:A:G	rs60567504	15	81021182	1.398E-05	80978247	81061697	70	51	11	rs60567504;rs10851939;rs10851940;rs11072938;rs12900485;rs12708529;rs13380029;rs7177476;rs13380392;rs12441947;rs79348488	1	rs60567504		Externalizing behaviour (multivariate analysis)	Predicted visceral adipose tissue; Hand grip strength; BMI; Migraine; Waist-hip ratio; Adult body size; Immune response to smallpox vaccine (IL-6)
40	16:9634281:C:G	rs4781389	16	9634281	2.929E-05	9568649	9649596	83	67	1	rs4781389	1	rs4781389			Facial morphology (factor 17, height of vermillion upper lip); Visceral fat
41	16:17967378:A:G	rs6498749	16	17967378	2.037E-06	17886842	18051710	169	125	57	rs6498749;rs55921186;rs7185305;rs12598612;rs56339039;rs8054010;rs113571972;rs111379377;rs12444455;rs8053346;rs8052057;rs62046413;rs9940860;rs4780665;rs4781897;rs891160;rs12599542;rs12596698;rs12448269;rs9925175;rs7206464;rs9302534;rs4781927;rs6498741;rs4465598;rs1353164;rs1353163;rs1353162;rs9933645;rs12708836;rs11861766;rs7201512;rs1493868;rs7194444;rs4780668;rs4781923;rs4280221;rs4553614;rs4541060;rs11647916;rs7500242;rs4264378;rs4594247;rs4480791;rs9940791;rs9930620;rs9930639;rs4781922;rs4781921;rs4781920;rs4781919;rs1389504;rs2015573;rs4140666;rs11075382;rs7192582;rs72771765	1	rs6498749	ADHD; Self-reported math ability	Cognitive empathy; Disruptive behavior (multivariate analysis); Externalizing Behaviour; Smoking initiation	
42	16:87502493:C:T	rs9927618	16	87502493	5.487E-05	87384328	87532770	94	42	9	rs9927618;rs9936994;rs28562609;rs4843242;rs59002427;rs11642390;rs28612602;rs28503604;rs28485311	1	rs9927618	Highest math class taken (MTAG)	Sensitivity to environmental stress and adversity; Anxiety; Neuroticism; Well being; Major depression; Schizophrenia	
43	18:22648505:A:C	rs8089996	18	22648505	9.767E-05	22589299	22654067	27	20	1	rs8089996	1	rs8089996	Educational attainment	Chronotype/morningness; Age at first sexual intercourse	
44	18:40225585:C:T	rs8089174	18	40225585	7.282E-05	40197660	40320317	254	123	5	rs8089174;rs58137875;rs7234212;rs1346889;rs12457128	1	rs8089174	Educational attainment; Cognitive performance; Math ability;	IDP dMRI TBSS OD Superior corona radiata variables; Brain morphology (MOSTest)/volumetric/diffusion variables; Externalising Behaviour; Smoking initiation (ever regular vs never regular) (MTAG)	Iris color (b* coordinate)
45	18:42754468:C:T	rs11877152	18	42754468	2.274E-05	42632652	42785997	31	24	1	rs11877152	1	rs11877152	Educational attainment; Cognitive performance (MTAG)	Smoking initiation (ever regular vs never regular) (MTAG)	Waist-hip variables; Biological sex; Parental Longevity
46	20:21467208:A:G	rs11697152	20	21467208	4.21E-07	21154234	21549630	177	127	7	rs11697152;rs1885284;rs4426587;rs6137363;rs6132437;rs6132438;rs2180965	1	rs11697152	Attention deficit hyperactivity disorder (childhood); Educational attainment	Autism spectrum disorder (MTAG); Insomnia; Schizophrenia; Refractive error; Retinal vascular fractal variables	Biological sex; Unilateral cleft lip and palate; Hip circumference adjusted for BMI
47	20:30746248:C:T	rs6061195	20	30746248	1.023E-06	30660621	31163052	367	159	5	rs6061195;rs2424874;rs2424877;rs2424878;rs6061182	1	rs6061195	Cognitive aspects of educational attainment; Cognitive performance; Common executive function	Insomnia; Smoking variables; Chronotype/morningness	Inflammatory bowel disease; Adolescent idiopathic scoliosis; Microalbuminuria; Urinary albumin-to-creatinine ratio; Chronic inflammatory diseases (ankylosing spondylitis, Crohn's disease, psoriasis, primary sclerosing cholangitis, ulcerative colitis) (pleiotropy); Crohn's disease; IBD; Monocyte percentage of white cells; Red cell distribution width; Aging traits (healthspan, parental lifespan or longevity) (multivariate analysis); Thymol sulfate levels in elite athletes
48	20:50966307:C:T	rs17794954	20	50966307	0.0001093	50809213	51263833	430	253	1	rs17794954	1	rs17794954		Depressive symptoms; Insomnia; Neuroticism; Well being; Stress sensitivity (neuroticism score x major depressive disorder status interaction)	Adolescent idiopathic scoliosis; Body size and related waist variables; Hand grip strength; Urinary sodium excretion; Type 2 Diabetes; Moderate-to-vigorous intensity physical activity during leisure time (MTAG); Neurofibrillary tangles (SNP x SNP interaction)
49	21:46570896:A:C	rs427943	21	46570896	5.084E-05	46496949	46699113	113	84	8	rs427943;rs407133;rs372519;rs394872;rs395761;rs397092;rs400997;rs403694	1	rs427943	Educational attainment (years of education)	Insomnia; Smoking variables; Major Depression; Age at first birth	HDL cholesterol levels; Gastroesophageal reflux disease; BMI and related variables; Metabolic biomarkers; Triglycerides; Sex hormone-binding globulin levels; Pulmonary function
