# Supplementary material for: Genetic neurodevelopmental clustering and dyslexia
Source: Mol Psychiatry. 2024 Jul 15;30(1):140–50. doi: 10.1038/s41380-024-02649-8 (PMC11649571; doi:10.1038/s41380-024-02649-8)
Supplement: Supplementary file 5 — Supplementary Table 5 [file 41380_2024_2649_MOESM5_ESM.pdf]

Supplementary Table 5. Prioritised genes within genomic loci that associate with both ADHD and dyslexia. Previous gene associations with dyslexia are shown in bold and in red for ADHD.

ensg	symbol	chr	start	end	strand	type	entrezID	HUGO	pLI	ncRVIS	posMapSNPs	posMapMaxCADD	minGwasP	IndSigSNPs	GenomicLocus
ENSG00000253313	C1orf210	1	43747554	43751288	-1	protein_coding	149466	C1orf210	0.001219636	0.676593983	3	16.67	NA	rs3120276;rs2991990;rs1198982;rs1199039;rs7527092;rs2282225;rs6694318	1
ENSG00000066056	TIE1	1	43766664	43788779	1	protein_coding	7075	TIE1	8.71417E-12	-0.019633805	37	16.67	3.66768E-07	rs3120276;rs2991990;rs1198982;rs1199039;rs7527092;rs2282225;rs6694318	1
ENSG00000117400	MPL	1	43803478	43818443	1	protein_coding	4352	MPL	1.43928E-06	-0.172843809	19	22.4	4.58724E-06	rs7527092;rs2282225;rs6694318;rs3120276;rs2991990;rs1198982;rs1199039	1
ENSG00000117399	CDC20	1	43824626	43828874	1	protein_coding	991	CDC20	0.000181854	-0.322700115	13	22.4	4.27147E-06	rs7527092;rs2282225;rs6694318;rs3120276;rs2991990;rs1198982;rs1199039	1
ENSG00000066322	ELOVL1	1	43829068	43833696	-1	protein_coding	64834	ELOVL1	0.22341453	NA	17	22.4	3.97068E-06	rs7527092;rs2282225;rs6694318;rs3120276;rs2991990;rs1198982;rs1199039	1
ENSG00000159479	MED8	1	43849588	43855479	-1	protein_coding	112950	MED8	8.15826E-06	0.403723391	18	18.26	3.7126E-06	rs7527092;rs2282225;rs6694318;rs3120276;rs2991990;rs1198982;rs1199039	1
ENSG00000198198	SZT2	1	43855553	43918321	1	protein_coding	23334	SZT2	1.51747E-07	0.623916217	58	18.87	4.18072E-06	rs7527092;rs2282225;rs6694318;rs3120276;rs2991990;rs1198982;rs1199039	1
ENSG00000178922	HYI	1	43916824	43919660	-1	protein_coding	81888	HYI	3.15819E-08	-0.187049626	16	18.87	2.3296E-05	rs3120276;rs2991990;rs1198982;rs1199039	1
ENSG00000196872	KIAA1211L	2	99410309	99552722	-1	protein_coding	343990	KIAA1211L	NA	NA	18	14.04	2.06415E-05	rs10178956	3
ENSG00000135951	TSGA10	2	99613724	99771427	-1	protein_coding	80705	TSGA10	0.894352624	0.321841592	8	2.969	NA	rs12475639;rs12464785;rs17022564;rs57242893;rs55815229;rs62153855;rs58086269;rs6713608;rs23	3
ENSG00000241962	C2orf15	2	99757948	99939204	1	protein_coding	150590	C2orf15	0.543252453	-0.867773362	83	13.29	4.78241E-05	rs12475639;rs12464785;rs17022564;rs57242893;rs55815229;rs62153855;rs58086269;rs6713608;rs23	3
ENSG00000185674	LYG2	2	99858709	99871745	-1	protein_coding	254773	LYG2	5.77012E-06	-0.086292636	10	5.684	0.000357423	rs12475639;rs12464785;rs17022564;rs57242893;rs55815229;rs62153855;rs58086269;rs6713608;rs23	3
ENSG00000144214	LYG1	2	99900701	99921205	-1	protein_coding	129530	LYG1	0.000104428	0.013563013	46	13.29	4.78241E-05	rs12475639;rs12464785;rs17022564;rs57242893;rs55815229;rs62153855;rs58086269;rs6713608;rs23	3
ENSG00000115514	TXNDC9	2	99935445	99957165	-1	protein_coding	10190	TXNDC9	0.223930351	-0.642788538	47	13.29	4.78241E-05	rs12475639;rs12464785;rs17022564;rs57242893;rs55815229;rs62153855;rs58086269;rs6713608;rs23	3
ENSG00000158417	EIF5B	2	99953816	100017789	1	protein_coding	9669	EIF5B	0.895387513	-0.523223638	118	28.6	4.71513E-05	rs12475639;rs12464785;rs17022564;rs57242893;rs55815229;rs62153855;rs58086269;rs6713608;rs23	3
ENSG00000135945	REV1	2	100016938	100106497	-1	protein_coding	51455	REV1	0.997023541	NA	140	17.02	4.88481E-05	rs12475639;rs12464785;rs17022564;rs57242893;rs55815229;rs62153855;rs58086269;rs6713608;rs23	3
ENSG00000128655	PDE11A	2	178487980	178973066	-1	protein_coding	50940	PDE11A	4.16725E-31	1.159768379	1	1.42	0.001110178	rs1900251;rs1374257;rs334051	5
ENSG00000155636	RBM45	2	178977151	179003738	1	protein_coding	129831	RBM45	0.000125349	-0.549454408	8	4.684	0.000295285	rs1900251;rs1374257;rs334051	5
ENSG00000079156	OSBPL6	2	179059208	179264160	1	protein_coding	114880	OSBPL6	0.999012304	NA	10	10.78	0.00844867	rs1900251;rs1374257;rs334051	5
ENSG00000138386	NAB1	2	191511472	191557492	1	protein_coding	4664	NAB1	0.848508408	-1.336164452	33	15.59	7.33548E-06	rs72917135;rs6706715;rs13004849;rs6719351;rs6737278;rs12693582;rs13382604;rs10202642;rs1301	6
ENSG00000065413	ANKRD44	2	197831741	198175897	-1	protein_coding	91526	ANKRD44	0.775902812	NA	48	18.47	4.98984E-05	rs1979239;rs2045242;rs10497806;rs10166328;rs7564924;rs1598469;rs10203581;rs55656916;rs10931	7
ENSG00000115524	**SF3B1**	2	198254508	198299815	-1	protein_coding	23451	SF3B1	0.999999973	-0.619089113	26	18.96	6.84336E-06	rs1979239;rs2045242;rs10497806;rs10166328;rs7564924;rs1598469;rs10203581;rs55656916;rs10931	7
ENSG00000115520	**COQ10B**	2	198318147	198340032	1	protein_coding	80219	COQ10B	0.140032717	-0.989155849	10	9.007	6.99538E-06	rs1979239;rs2045242;rs10497806;rs10166328;rs7564924;rs1598469;rs10203581;rs55656916;rs10931	7
ENSG00000144381	**HSPD1**	2	198351305	198381461	-1	protein_coding	3329	HSPD1	0.985208244	-0.813832273	22	18.44	6.99538E-06	rs1979239;rs2045242;rs10497806;rs10166328;rs7564924;rs1598469;rs10203581;rs55656916;rs10931	7
ENSG00000115541	**HSPE1**	2	198364718	198368181	1	protein_coding	3336	HSPE1	0.787528048	-0.835658197	15	18.44	7.544E-06	rs1979239;rs2045242;rs10497806;rs10166328;rs7564924;rs1598469;rs10203581;rs55656916;rs10931	7
ENSG00000270757	**HSPE1-MOB4**	2	198365137	198415450	1	protein_coding	100529241	HSPE1-MOB4	0.889798358	NA	32	18.44	6.0053E-06	rs1979239;rs2045242;rs10497806;rs10166328;rs7564924;rs1598469;rs10203581;rs55656916;rs10931	7
ENSG00000115540	**MOB4**	2	198380295	198418423	1	protein_coding	25843	MOB4	0.77783291	-1.626843079	27	15.71	5.30972E-06	rs1979239;rs2045242;rs10497806;rs10166328;rs7564924;rs1598469;rs10203581;rs55656916;rs10931	7
ENSG00000162944	**RFTN2**	2	198432948	198540769	-1	protein_coding	130132	RFTN2	0.002076962	0.166398078	75	13.6	2.32485E-07	rs1979239;rs2045242;rs10497806;rs10166328;rs7564924;rs1598469;rs10203581;rs55656916;rs10931	7
ENSG00000222017	**AC011997.1**	2	198557830	198639547	1	protein_coding	NA	NA	NA	NA	57	12.07	1.42089E-07	rs1979239;rs2045242;rs10497806;rs10166328;rs7564924;rs1598469;rs10203581;rs55656916;rs10931	7
ENSG00000247626	MARS2	2	198570087	198573113	1	protein_coding	92935	MARS2	1.41647E-06	-0.302062448	13	12.07	4.22627E-07	rs1979239;rs2045242;rs10497806;rs10166328;rs7564924;rs1598469;rs10203581;rs55656916;rs10931	7
ENSG00000152430	**BOLL**	2	198591603	198651486	-1	protein_coding	66037	BOLL	0.007902184	-1.241883899	48	11.55	1.42089E-07	rs1979239;rs2045242;rs10497806;rs10166328;rs7564924;rs1598469;rs10203581;rs55656916;rs10931	7
ENSG00000115896	**PLCL1**	2	198669426	199437305	1	protein_coding	5334	PLCL1	0.011052371	NA	284	26.7	1.38737E-07	rs1865586;rs6734781;rs1979239;rs2045242;rs10497806;rs10166328;rs7564924;rs1598469;rs1020358	7
ENSG00000168016	TRANK1	3	36868311	36986548	-1	protein_coding	9881	TRANK1	2.79379E-10	-0.699749511	28	9.982	0.000472506	rs2302504	9
ENSG00000178567	EPM2AIP1	3	37027357	37034795	-1	protein_coding	9852	EPM2AIP1	0.164415268	0.811082225	31	13.44	0.000144501	rs2302504	9
ENSG00000076242	MLH1	3	37034823	37107380	1	protein_coding	4292	MLH1	0.739578311	-0.437927667	107	15.12	0.000105143	rs2302504	9
ENSG00000093167	LRRFIP2	3	37094117	37225180	-1	protein_coding	9209	LRRFIP2	4.8006E-08	-0.734402353	188	16.52	0.000105143	rs2302504	9
ENSG00000144674	GOLGA4	3	37284668	37408242	1	protein_coding	2803	GOLGA4	2.26165E-09	-0.619794716	104	24.6	0.003869418	rs2302504	9
ENSG00000198590	C3orf35	3	37427760	37476988	1	protein_coding	339883	C3orf35	0.01413532	0.030004299	34	10.03	0.004209688	rs2302504	9
ENSG00000213672	NCKIPSD	3	48701364	48723797	-1	protein_coding	51517	NCKIPSD	0.007131362	-0.551018211	10	16.5	0.001577987	rs9682444;rs9311433;rs4974084;rs4521268;rs4513485;rs3212;rs4364202;rs4279134;rs6779394;rs677	10
ENSG00000068745	IP6K2	3	48725436	48777786	-1	protein_coding	51447	IP6K2	0.989457703	-0.392798697	40	16.5	0.000243483	rs9682444;rs9311433;rs4974084;rs4521268;rs4513485;rs3212;rs4364202;rs4279134;rs6779394;rs677	10
ENSG00000114302	PRKAR2A	3	48782030	48885279	-1	protein_coding	5576	PRKAR2A	0.983250576	-2.868402322	55	12.14	0.000104886	rs9682444;rs9311433;rs4974084;rs4521268;rs4513485;rs3212;rs4364202;rs4279134;rs6779394;rs677	10
ENSG00000178537	SLC25A20	3	48894369	48936426	-1	protein_coding	788	SLC25A20	2.04567E-07	-0.773546303	41	11.65	2.25781E-05	rs9682444;rs9311433;rs4974084;rs4521268;rs4513485;rs3212;rs4364202;rs4279134;rs6779394;rs677	10
ENSG00000221883	ARIH2OS	3	48955221	48956818	-1	protein_coding	646450	ARIH2OS	0.437613435	-0.628345895	16	11.63	0.000117023	rs9682444;rs9311433;rs4974084;rs4521268;rs4513485;rs3212;rs4364202;rs4279134;rs6779394;rs677	10
ENSG00000177479	ARIH2	3	48956254	49023815	1	protein_coding	10425	ARIH2	0.987412495	-1.699919822	50	11.63	0.000117023	rs9682444;rs9311433;rs4974084;rs4521268;rs4513485;rs3212;rs4364202;rs4279134;rs6779394;rs677	10
ENSG00000178467	P4HTM	3	49027319	49044587	1	protein_coding	54681	P4HTM	0.023274936	NA	12	13.56	0.000143285	rs9682444;rs9311433;rs4974084;rs4521268;rs4513485;rs3212;rs4364202;rs4279134;rs6779394;rs677	10
ENSG00000178252	WDR6	3	49044495	49053386	1	protein_coding	11180	WDR6	9.19648E-07	-0.058949543	10	15.78	0.000171021	rs9682444;rs9311433;rs4974084;rs4521268;rs4513485;rs3212;rs4364202;rs4279134;rs6779394;rs677	10
ENSG00000178149	DALRD3	3	49052921	49059726	-1	protein_coding	55152	DALRD3	1.84814E-05	0.072306759	6	15.78	0.000173578	rs9682444;rs9311433;rs4974084;rs4521268;rs4513485;rs3212;rs4364202;rs4279134;rs6779394;rs677	10
ENSG00000178057	NDUFAF3	3	49057892	49060928	1	protein_coding	25915	NDUFAF3	4.85973E-05	-2.090068809	5	15.78	0.000173578	rs9682444;rs9311433;rs4974084;rs4521268;rs4513485;rs3212;rs4364202;rs4279134;rs6779394;rs677	10
ENSG00000178035	IMPDH2	3	49061758	49066841	-1	protein_coding	3615	IMPDH2	0.001353166	-0.509342888	10	15.78	0.000173578	rs9682444;rs9311433;rs4974084;rs4521268;rs4513485;rs3212;rs4364202;rs4279134;rs6779394;rs677	10
ENSG00000198218	QRICH1	3	49067140	49131796	-1	protein_coding	54870	QRICH1	0.996069748	-0.27929443	35	10.95	1.00394E-05	rs9682444;rs9311433;rs4974084;rs4521268;rs4513485;rs3212;rs4364202;rs4279134;rs6779394;rs677	10
ENSG00000172053	QARS	3	49133365	49142553	-1	protein_coding	5859	QARS	8.7897E-08	-0.634755548	12	12.85	8.90558E-06	rs9682444;rs9311433;rs4974084;rs4521268;rs4513485;rs3212;rs4364202;rs4279134;rs6779394;rs677	10
ENSG00000172046	USP19	3	49145479	49158371	-1	protein_coding	10869	USP19	0.999991981	-0.429632772	11	21.3	8.90558E-06	rs9682444;rs9311433;rs4974084;rs4521268;rs4513485;rs3212;rs4364202;rs4279134;rs6779394;rs677	10
ENSG00000172037	LAMB2	3	49158547	49170551	-1	protein_coding	3913	LAMB2	7.45007E-11	-0.451081722	7	21.3	8.90558E-06	rs9682444;rs9311433;rs4974084;rs4521268;rs4513485;rs3212;rs4364202;rs4279134;rs6779394;rs677	10
ENSG00000177352	CCDC71	3	49199968	49203754	-1	protein_coding	64925	CCDC71	0.016989639	NA	13	10.2	0.000147538	rs9682444;rs9311433;rs4974084;rs4521268;rs4513485;rs3212;rs4364202;rs4279134;rs6779394;rs677	10
ENSG00000185909	KLHDC8B	3	49209044	49213917	1	protein_coding	200942	KLHDC8B	0.01042142	-0.077352225	13	10.2	0.000152433	rs9682444;rs9311433;rs4974084;rs4521268;rs4513485;rs3212;rs4364202;rs4279134;rs6779394;rs677	10
ENSG00000236980	C3orf84	3	49215065	49229291	-1	protein_coding	646498	C3orf84	0.000221292	0.059049684	23	15.24	0.000137858	rs9682444;rs9311433;rs4974084;rs4521268;rs4513485;rs3212;rs4364202;rs4279134;rs6779394;rs677	10
ENSG00000173421	CCDC36	3	49235861	49295537	1	protein_coding	339834	CCDC36	0.509348612	-0.452712817	48	15.24	0.000118108	rs4513485;rs4364202;rs6779394;rs6770112;rs6784111;rs4955410;rs12493001;rs12636030;rs1248758	10
ENSG00000225399	RP11-3B7.1	3	49297518	49298744	1	protein_coding	NA	NA	NA	NA	10	11.88	0.000166687	rs9682444;rs9311433;rs4974084;rs4521268;rs4513485;rs3212;rs4364202;rs4279134;rs6779394;rs677	10
ENSG00000188315	C3orf62	3	49306035	49315342	-1	protein_coding	375341	C3orf62	0.007124659	-1.950313785	14	11.88	2.91873E-05	rs9682444;rs9311433;rs4974084;rs4521268;rs4513485;rs3212;rs4364202;rs4279134;rs6779394;rs677	10
ENSG00000114316	USP4	3	49315264	49378145	-1	protein_coding	7375	USP4	6.20194E-07	NA	45	9.802	1.32751E-05	rs4521268;rs4513485;rs3212;rs4364202;rs4279134;rs6779394;rs6770112;rs6784111;rs4955410;rs124	10
ENSG00000233276	GPX1	3	49394609	49396033	-1	protein_coding	2876	GPX1	0.006931651	-0.616399091	14	7.094	0.000122242	rs12493284;rs3774799;rs11720964;rs4554002;rs7640903;rs11709788;rs11920251;rs1865741;rs34142	10
ENSG00000067560	RHOA	3	49396578	49450431	-1	protein_coding	387	RHOA	0.804697934	-0.430262742	75	16.24	3.54206E-05	rs4974084;rs4521268;rs4513485;rs3212;rs4364202;rs4279134;rs6779394;rs6770112;rs6784111;rs495	10
ENSG00000145022	**TCTA**	3	49449639	49453908	1	protein_coding	6988	TCTA	0.013264848	-0.59594101	24	16.24	0.000114845	rs4974084;rs4521268;rs4513485;rs3212;rs4364202;rs4279134;rs6779394;rs6770112;rs6784111;rs495	10
ENSG00000145020	**AMT**	3	49454211	49460186	-1	protein_coding	275	AMT	0.000569475	-0.022637077	25	16.24	0.000114845	rs9682444;rs9311433;rs4974084;rs4521268;rs4513485;rs3212;rs4364202;rs4279134;rs6779394;rs677	10
ENSG00000145029	NICN1	3	49460379	49466759	-1	protein_coding	84276	NICN1	0.257245529	-1.087291051	22	13.86	0.000114845	rs12493284;rs3774799;rs11720964;rs4554002;rs7640903;rs11709788;rs11920251;rs1865741;rs34142	10
ENSG00000173402	DAG1	3	49506146	49573048	1	protein_coding	1605	DAG1	0.940921791	-0.838742424	80	19.42	0.000141428	rs4974084;rs4521268;rs4513485;rs3212;rs4364202;rs4279134;rs6779394;rs6770112;rs6784111;rs495	10
ENSG00000164061	BSN	3	49591922	49708978	1	protein_coding	8927	BSN	0.999999992	-1.285114003	17	13.05	0.000447553	rs9682444;rs9311433;rs4974084;rs4521268;rs4513485;rs3212;rs4364202;rs4279134;rs6779394;rs677	10
ENSG00000183763	**TRAIP**	3	49866034	49894007	-1	protein_coding	10293	TRAIP	0.125103592	-0.132254904	7	17.61	0.000204775	rs6800021;rs2526389;rs1046953;rs2188151;rs2518796;rs2518795;rs2624835;rs2624833;rs13059311;	10
ENSG00000164076	CAMKV	3	49895421	49907655	-1	protein_coding	79012	CAMKV	0.996846171	-0.638322632	16	18.51	0.000204775	rs6800021;rs2526389;rs1046953;rs2188151;rs2518796;rs2518795;rs2624835;rs2624833;rs13059311;	10
ENSG00000164078	MST1R	3	49924435	49941299	-1	protein_coding	4486	MST1R	1.69349E-20	-0.377644955	20	18.51	0.002642051	rs6800021;rs2526389;rs1046953;rs2188151;rs2624839;rs2518796;rs2518795;rs1005678;rs2624838;rs	10
ENSG00000228008	CTD-2330K9.3	3	49941278	49954370	1	protein_coding	NA	NA	NA	NA	19	13.15	NA	rs6800021;rs2526389;rs1046953;rs2188151;rs2624839;rs2518796;rs2518795;rs1005678;rs2624838;rs	10
ENSG00000164077	MON1A	3	49946302	49967606	-1	protein_coding	84315	MON1A	0.00636811	-0.629673162	26	13.15	0.000460643	rs6800021;rs2526389;rs1046953;rs2188151;rs2624839;rs2518796;rs2518795;rs1005678;rs2624838;rs	10
ENSG00000004534	RBM6	3	49977440	50137478	1	protein_coding	10180	RBM6	0.999999964	-0.685653183	188	17.77	0.000460643	rs6800021;rs2526389;rs1046953;rs2188151;rs2518796;rs2518795;rs2624838;rs2624835;rs2624833;rs	10
ENSG00000003756	RBM5	3	50126341	50156454	1	protein_coding	10181	RBM5	0.99999947	-0.605888711	39	18.47	0.007033498	rs6800021;rs2526389;rs1046953;rs2188151;rs2518796;rs2518795;rs1005678;rs2624835;rs2624833;rs	10
ENSG00000001617	**SEMA3F**	3	50192478	50226508	1	protein_coding	6405	SEMA3F	0.9999177	NA	43	19.09	2.15695E-08	rs6800021;rs2526389;rs1046953;rs2188151;rs2624839;rs2624838;rs2518796;rs2518795;rs2624835;rs	10
ENSG00000114349	GNAT1	3	50229045	50233949	1	protein_coding	2779	GNAT1	2.50523E-06	-0.274733411	20	14.65	2.35825E-06	rs6800021;rs2526389;rs2188151;rs2624839;rs2624838;rs2518796;rs2518795;rs2624835;rs1005678;rs	10
ENSG00000114353	GNAI2	3	50263724	50296787	1	protein_coding	2771	GNAI2	0.86116029	-0.965006324	2	3.571	0.00112475	rs2624838;rs2624835;rs2624833;rs13059311;rs9858297;rs13067082;rs1046956;rs12632110;rs126376	10

ENSG00000179564	*LSMEM2*	3	50316458	50325545	1	protein_coding	132228	*LSMEM2*	2.89144E-05	-0.548022292	1	1.468	NA	rs35137368	10
ENSG00000214706	*IFRD2*	3	50325163	50330349	-1	protein_coding	7866	*IFRD2*	5.5261E-06	-0.616737733	1	5.353	NA	rs2624838;rs2624835;rs2624833;rs13059311;rs9858297;rs13067082;rs1046956;rs12632110;rs126376	10
ENSG00000186792	*HYAL3*	3	50330262	50336899	-1	protein_coding	8372	*HYAL3*	1.52466E-05	-0.353149956	1	5.353	NA	rs2624838;rs2624835;rs2624833;rs13059311;rs9858297;rs13067082;rs1046956;rs12632110;rs126376	10
ENSG00000243477	*NAT6*	3	50333833	50336852	-1	protein_coding	24142	*NAT6*	0.402945153	-0.113189262	1	5.353	NA	rs2624838;rs2624835;rs2624833;rs13059311;rs9858297;rs13067082;rs1046956;rs12632110;rs126376	10
ENSG00000114378	*HYAL1*	3	50337320	50349812	-1	protein_coding	3373	*HYAL1*	0.005536706	0.476164437	1	5.353	NA	rs2624838;rs2624835;rs2624833;rs13059311;rs9858297;rs13067082;rs1046956;rs12632110;rs126376	10
ENSG00000114395	*CYB561D2*	3	50388126	50395891	1	protein_coding	11068	*CYB561D2*	0.014710475	-0.298456388	1	1.163	NA	rs2624838;rs2624835;rs2624833;rs13059311;rs9858297;rs13067082;rs1046956;rs12632110;rs126376	10
ENSG00000272104	*XXcos-LUCA11.5*	3	50388323	50405354	1	protein_coding	NA	*NA*	NA	NA	1	1.163	NA	rs2624838;rs2624835;rs2624833;rs13059311;rs9858297;rs13067082;rs1046956;rs12632110;rs126376	10
ENSG00000126062	*TMEM115*	3	50392180	50397041	-1	protein_coding	11070	*TMEM115*	0.876895269	-0.780371829	1	1.163	NA	rs2624838;rs2624835;rs2624833;rs13059311;rs9858297;rs13067082;rs1046956;rs12632110;rs126376	10
ENSG00000007402	*CACNA2D2*	3	50400233	50541675	-1	protein_coding	9254	*CACNA2D2*	0.999693297	NA	1	1.163	NA	rs2624838;rs2624835;rs2624833;rs13059311;rs9858297;rs13067082;rs1046956;rs12632110;rs126376	10
ENSG00000175161	***CADM2***	3	85008132	86123579	1	protein_coding	253559	*CADM2*	0.98466891	0.117806454	890	20.6	6.63827E-08	rs2163971;rs11921010;rs10511087;rs1368742;rs12637798;rs12638482;rs62250685;rs11918899;rs154	12
ENSG00000185565	***LSAMP***	3	115521235	117716095	-1	protein_coding	4045	*LSAMP*	0.946283747	NA	18	16.88	6.85962E-06	rs4688050	13
ENSG00000065534	*MYLK*	3	123328896	123603178	-1	protein_coding	4638	*MYLK*	9.3792E-06	0.303166207	25	14.66	6.33831E-05	rs34067381	14
ENSG00000175455	*CCDC14*	3	123616152	123680564	-1	protein_coding	64770	*CCDC14*	3.40559E-07	0.11388799	52	14.66	6.33831E-05	rs34067381	14
ENSG00000065371	*ROPN1*	3	123687368	123711025	-1	protein_coding	54763	*ROPN1*	0.008185352	-0.715620742	14	9.294	0.000317035	rs34067381	14
ENSG00000073711	***PPP2R3A***	3	135684515	135866733	1	protein_coding	5523	*PPP2R3A*	0.920487875	-0.863679102	144	19.34	2.5098E-11	rs9852406;rs56308637;rs28875187;rs1403767;rs73230099;rs1052620;rs9867325;rs9840500;rs617917	15
ENSG00000174579	***MSL2***	3	135867764	135916083	-1	protein_coding	55167	*MSL2*	0.889663742	-1.742045868	39	22.4	4.17082E-10	rs9852406;rs56308637;rs28875187;rs1403767;rs73230099;rs1052620;rs9867325;rs9840500;rs617917	15
ENSG00000114054	***PCCB***	3	135969148	136056738	1	protein_coding	5096	*PCCB*	0.000735839	-0.385160254	77	12.17	5.85097E-10	rs61791757;rs6779146;rs61789561;rs1154988;rs645040;rs687339;rs511154;rs1153871;rs61789601;rs	15
ENSG00000118007	***STAG1***	3	136055077	136471220	-1	protein_coding	10274	*STAG1*	0.99999991	-0.647637604	329	16.64	4.70852E-10	rs61791757;rs6779146;rs61789561;rs1154988;rs645040;rs687339;rs511154;rs1153871;rs61789601;rs	15
ENSG00000168917	***SLC35G2***	3	136537489	136574734	1	protein_coding	80723	*SLC35G2*	0.066395336	-0.300343122	20	16.5	5.96014E-10	rs73230099;rs1052620;rs9867325;rs9840500;rs9852406;rs56308637;rs28875187;rs1403767;rs617895	15
ENSG00000158092	*NCK1*	3	136581050	136668665	1	protein_coding	4690	*NCK1*	0.001035026	-0.568989094	24	16.5	8.85042E-10	rs9852406;rs56308637;rs28875187;rs1403767;rs73230099;rs1052620;rs9867325;rs9840500;rs617895	15
ENSG00000174564	*IL20RB*	3	136665072	136729927	1	protein_coding	53833	*IL20RB*	0.000354183	0.165775461	3	7.133	6.43199E-05	rs1403767;rs73230099;rs1052620;rs9867325;rs9840500;rs61789561;rs1154988;rs645040;rs687339;rs	15
ENSG00000158186	***MRAS***	3	138066539	138124375	1	protein_coding	22808	*MRAS*	0.859946833	-0.413633907	10	7.011	0.000331519	rs7609893;rs1109088	16
ENSG00000158220	*ESYT3*	3	138153428	138200528	1	protein_coding	83850	*ESYT3*	2.43267E-11	-0.947164338	22	22.4	1.96755E-05	rs7609893;rs1109088	16
ENSG00000114107	*CEP70*	3	138213186	138313380	-1	protein_coding	80321	*CEP70*	5.95204E-11	-0.571935395	123	13.84	0.006214534	rs7609893;rs1109088	16
ENSG00000158234	*FAIM*	3	138327448	138352218	1	protein_coding	55179	*FAIM*	0.068117194	-0.930130513	36	12.67	0.002172315	rs7609893;rs1109088	16
ENSG00000075711	*DLG1*	3	196769431	197026171	-1	protein_coding	1739	*DLG1*	0.998742049	-0.290718549	95	18.92	2.37639E-06	rs338217;rs338222;rs395572	17
ENSG00000184305	***CCSER1***	4	91048686	92523064	1	protein_coding	401145	*CCSER1*	0.211350847	0.195161488	191	12.35	8.87092E-06	rs7693414;rs7671307	18
ENSG00000005108	*THSD7A*	7	11409984	11871824	-1	protein_coding	221981	*THSD7A*	0.999280749	-0.675169844	21	23.8	2.84928E-05	rs17164600;rs7803385;rs12055997;rs7807369;rs11979605;rs118134876	22
ENSG00000158321	***AUTS2***	7	69063905	70258054	1	protein_coding	26053	*AUTS2*	0.996187482	-0.774735851	331	19.56	7.16394E-06	rs6960938;rs10242297;rs60417126;rs56134694;rs10261510;rs59342262;rs55901635;rs10241446;rs28	23
ENSG00000187416	*LHFPL3*	7	103969104	104549001	1	protein_coding	375612	*LHFPL3*	0.795069316	NA	9	11.21	0.000805458	rs113905912;rs56016333;rs13237211	24
ENSG00000005483	*KMT2E*	7	104654626	104754808	1	protein_coding	55904	*KMT2E*	0.999999995	-0.608755693	37	22.3	8.32788E-05	rs113905912;rs56016333;rs4727614;rs6955349;rs7776707;rs10238507;rs10266871;rs6943183;rs2299	24
ENSG00000135250	***SRPK2***	7	104751151	105039755	-1	protein_coding	6733	*SRPK2*	0.997504054	-0.650865281	301	15.83	2.72121E-05	rs4727614;rs6955349;rs7776707;rs10238507;rs10266871;rs6943183;rs2299308;rs3779210;rs4730072	24
ENSG00000172728	*FUT10*	8	33228342	33330940	-1	protein_coding	84750	*FUT10*	3.07919E-07	-0.077987793	1	1.753	NA	rs75836205	25
ENSG00000133874	*RNF122*	8	33405273	33424643	-1	protein_coding	79845	*RNF122*	0.076386253	1.169679086	1	4.689	NA	rs75836205;rs6990255;rs118041269;rs117396993;rs80318442;rs74427054	25
ENSG00000133878	*DUSP26*	8	33448856	33457624	-1	protein_coding	78986	*DUSP26*	0.003914765	-0.465430541	1	3.25	NA	rs146649743	25
ENSG00000156687	***UNC5D***	8	35092975	35654068	1	protein_coding	137970	*UNC5D*	0.849201861	NA	1	6.735	NA	rs118041269;rs117396993;rs80318442;rs74427054;rs76013678	25
ENSG00000164989	***CCDC171***	9	15552895	16061661	1	protein_coding	203238	*CCDC171*	NA	0.216049891	884	21.6	9.67976E-07	rs2457637;rs4740614;rs7873152;rs6474930;rs2821545;rs9407624;rs7875367;rs276453;rs276449;rs27	26
ENSG00000188938	*FAM120AOS*	9	96208776	96215874	-1	protein_coding	158293	*FAM120AOS*	0.514390707	0.007431442	19	10.73	0.000393593	rs10821140;rs10992756;rs10992757;rs4743924;rs4744247;rs1556416;rs2150749;rs4744249;rs109927	28
ENSG00000048828	***FAM120A***	9	96214004	96328397	1	protein_coding	23196	*FAM120A*	0.99990705	-0.298117336	133	18.33	3.73229E-05	rs10821140;rs10992756;rs10992757;rs4743924;rs4744247;rs1556416;rs2150749;rs4744249;rs109927	28
ENSG00000197724	***PHF2***	9	96338689	96441869	1	protein_coding	5253	*PHF2*	0.994132137	0.745197979	145	11.57	2.19689E-05	rs10821140;rs10992756;rs10992757;rs4743924;rs4744247;rs1556416;rs2150749;rs4744249;rs109927	28
ENSG00000156395	***SORCS3***	10	106400859	107024993	1	protein_coding	22986	*SORCS3*	0.325134264	0.170375294	395	20.1	1.03405E-06	rs4350297;rs61867284;rs74317294;rs17766570;rs11594644;rs61867285;rs79883993;rs61867287;rs17	30
ENSG00000086827	*ZW10*	11	113603909	113644533	-1	protein_coding	9183	*ZW10*	0.047262892	-0.861238849	1	0.571	0.0019016	rs12289874	32
ENSG00000228607	*CLDN25*	11	113650469	113651222	1	protein_coding	644672	*CLDN25*	2.36412E-09	-0.159950836	1	0.571	0.0019016	rs12289874	32
ENSG00000048028	*USP28*	11	113668596	113746292	-1	protein_coding	57646	*USP28*	0.031657505	0.869552466	6	7.004	6.84017E-05	rs12289874	32
ENSG00000149305	*HTR3B*	11	113775399	113817287	1	protein_coding	9177	*HTR3B*	6.14953E-07	0.319177556	7	20.7	0.000138397	rs12289874	32
ENSG00000181418	*DDN*	12	49388932	49393092	-1	protein_coding	23109	*DDN*	0.986679784	-0.593068326	7	10.27	3.22741E-05	rs10783299;rs2293445;rs10875908;rs1138908;rs10783300;rs2117029;rs2293446;rs10875913;rs11168	33
ENSG00000181929	*PRKAG1*	12	49396057	49412980	-1	protein_coding	5571	*PRKAG1*	0.001637087	-0.36111794	20	18.98	3.22741E-05	rs10783299;rs2293445;rs10875908;rs1138908;rs10783300;rs2117029;rs2293446;rs10875913;rs11168	33
ENSG00000167548	*KMT2D*	12	49412758	49453557	-1	protein_coding	8085	*KMT2D*	1	-1.38903777	31	18.98	3.3759E-05	rs10783299;rs2293445;rs10875908;rs1138908;rs10783300;rs2117029;rs2293446;rs10875913;rs11168	33
ENSG00000167550	*RHEBL1*	12	49458468	49463808	-1	protein_coding	121268	*RHEBL1*	3.29299E-06	-0.739691541	16	10.56	4.86243E-05	rs10783299;rs2293445;rs10875908;rs1138908;rs10783300;rs2117029;rs2293446;rs10875913;rs11168	33
ENSG00000139549	*DHH*	12	49483204	49488602	-1	protein_coding	50846	*DHH*	0.259714235	-0.226342192	7	3.455	0.000148696	rs10783299;rs2293445;rs10875908;rs1138908;rs10783300;rs2117029;rs2293446;rs10875913;rs11168	33
ENSG00000150967	*ABCB9*	12	123405498	123466196	-1	protein_coding	23457	*ABCB9*	0.002901108	NA	15	16.51	0.000582599	rs1727295;rs1626703	34
ENSG00000111325	*OGFOD2*	12	123459127	123464590	1	protein_coding	79676	*OGFOD2*	3.80506E-07	-0.495075683	14	16.51	0.000582599	rs1727295;rs1626703	34
ENSG00000182196	*ARL6IP4*	12	123464607	123467456	1	protein_coding	51329	*ARL6IP4*	0.011234201	-0.527874207	13	16.51	0.000428405	rs1727295;rs1626703	34
ENSG00000090975	*PITPNM2*	12	123468027	123634562	-1	protein_coding	57605	*PITPNM2*	0.999993632	-0.297418703	100	16.51	8.17138E-05	rs1727295;rs1626703	34
ENSG00000051825	*MPHOSPH9*	12	123636867	123728561	-1	protein_coding	10198	*MPHOSPH9*	0.000759987	-0.13148433	215	17.2	5.666E-05	rs1727295;rs1626703	34
ENSG00000130921	*C12orf65*	12	123717463	123742506	1	protein_coding	91574	*C12orf65*	0.17232859	0.226571423	92	17.2	5.666E-05	rs1727295;rs1626703	34
ENSG00000111328	*CDK2AP1*	12	123745528	123756881	-1	protein_coding	8099	*CDK2AP1*	0.742361028	NA	38	14.16	0.000167876	rs1727295;rs1626703	34
ENSG00000139697	*SBNO1*	12	123773656	123849390	-1	protein_coding	55206	*SBNO1*	0.999999984	-1.467513986	64	22.9	0.000354145	rs1727295;rs1626703	34
ENSG00000183955	*SETD8*	12	123868320	123893905	1	protein_coding	387893	*SETD8*	0.945359271	0.950255994	38	14.37	0.000143676	rs1727295;rs1626703	34
ENSG00000150977	*RILPL2*	12	123899936	123921264	-1	protein_coding	196383	*RILPL2*	0.060872187	-0.185558551	27	14.37	NA	rs1727295;rs1626703	34
ENSG00000139971	*C14orf37*	14	58466453	58764857	-1	protein_coding	145407	*C14orf37*	8.27236E-05	2.430974882	129	17.81	5.27665E-05	rs6573194;rs12892257;rs10151752;rs7147757;rs28637941;rs2145598;rs58231973;rs10137475;rs1051	35
ENSG00000131966	*ACTR10*	14	58666798	58701750	1	protein_coding	55860	*ACTR10*	0.004736237	NA	40	13.26	0.001289851	rs6573194;rs12892257;rs10151752;rs7147757;rs28637941;rs2145598;rs58231973;rs10137475;rs1051	35
ENSG00000100567	*PSMA3*	14	58711549	58738730	1	protein_coding	5684	*PSMA3*	0.990281922	-0.459951181	40	11	9.7463E-05	rs6573194;rs12892257;rs10151752;rs7147757;rs28637941;rs2145598;rs58231973;rs10137475;rs1051	35
ENSG00000268466	*AL132989.1*	14	58754751	58755865	1	protein_coding	NA	*NA*	NA	NA	26	7.57	5.27665E-05	rs6573194;rs12892257;rs10151752;rs7147757;rs28637941;rs2145598;rs58231973;rs10137475;rs1051	35
ENSG00000032219	*ARID4A*	14	58765103	58840605	1	protein_coding	5926	*ARID4A*	0.999999263	-1.459535231	88	19.94	3.91727E-05	rs6573194;rs12892257;rs10151752;rs7147757;rs28637941;rs2145598;rs58231973;rs10137475;rs1051	35
ENSG00000196860	*TOMM20L*	14	58862634	58875419	1	protein_coding	387990	*TOMM20L*	0.000787655	NA	4	7.395	0.000111591	rs6573194;rs12892257;rs10151752;rs7147757;rs28637941;rs2145598;rs58231973;rs10137475;rs1051	35
ENSG00000100575	*TIMM9*	14	58875212	58894332	-1	protein_coding	26520	*TIMM9*	0.437845525	-0.428959584	1	8.624	NA	rs28398380;rs1957944;rs2147609;rs2348075	35
ENSG00000100578	*KIAA0586*	14	58894103	59015216	1	protein_coding	9786	*KIAA0586*	1.44868E-12	-0.513924372	1	8.624	NA	rs28398380;rs1957944;rs2147609;rs2348075	35
ENSG00000166923	*GREM1*	15	33010175	33026870	1	protein_coding	26585	*GREM1*	0.711099436	1.726139501	2	6.582	0.000200046	rs75406947;rs75606001;rs8031239;rs79070500	37
ENSG00000248905	*FMN1*	15	33057747	33486897	-1	protein_coding	342184	*FMN1*	1.72654E-13	12.27816947	14	12.91	2.28464E-05	rs75406947;rs75606001;rs8031239;rs79070500	37
ENSG00000137871	*ZNF280D*	15	56922379	57210769	-1	protein_coding	54816	*ZNF280D*	0.820128047	-0.120447238	145	15.08	0.000835369	rs2733170;rs2920264;rs2470079;rs2585088;rs1820995;rs2435907;rs2951901;rs2957574;rs2470082;rs	38
ENSG00000140262	*TCF12*	15	57210821	57591479	1	protein_coding	6938	*TCF12*	0.973899934	-0.962372862	472	18.6	7.23156E-06	rs2733170;rs2920264;rs2470079;rs2585088;rs1820995;rs2435907;rs2951901;rs2957574;rs2470082;rs	38
ENSG00000136379	*ABHD17C*	15	80972025	81047962	1	protein_coding	58489	*ABHD17C*	0.84082863	0.101230357	63	10.21	1.39827E-05	rs79348488;rs60567504;rs10851939;rs10851940;rs11072938;rs12900485;rs12708529;rs13380029;rs7	39
ENSG00000103888	*KIAA1199*	15	81071684	81244117	1	protein_coding	57214	*KIAA1199*	1.74567E-09	-0.795939316	1	4.98	7.614E-05	rs79348488;rs60567504;rs10851939;rs10851940;rs11072938;rs12900485;rs12708529;rs13380029;rs7	39
ENSG00000103264	*FBXO31*	16	87360593	87425748	-1	protein_coding	79791	*FBXO31*	0.911167117	-0.052861849	11	10.05	0.00010879	rs59002427;rs11642390;rs28612602;rs28503604;rs28485311;rs9927618;rs9936994;rs28562609;rs484	42
ENSG00000140941	***MAP1LC3B***	16	87417601	87438385	1	protein_coding	81631	*MAP1LC3B*	0.061760877	1.467386741	17	23.8	8.34717E-05	rs59002427;rs11642390;rs28612602;rs28503604;rs28485311;rs9927618;rs9936994;rs28562609;rs484	42
ENSG00000140948	*ZCCHC14*	16	87439852	87525651	-1	protein_coding	23174	*ZCCHC14*	0.948835727	-1.830202428	87	23.8	5.48738E-05	rs59002427;rs9927618;rs28562609;rs4843242;rs11642390;rs28612602;rs28503604;rs28485311;rs993	42
ENSG00000198795	*ZNF521*	18	22641890	22932154	-1	protein_coding	25925	*ZNF521*	0.999979351	NA	14	18.85	9.7666E-05	rs8089996	43
ENSG00000152214	*RIT2*	18	40323192	40695657	-1	protein_coding	6014	*RIT2*	0.060048528	0.384223621	5	1.747	NA	rs8089174;rs58137875;rs7234212;rs1346889;rs12457128	44
ENSG00000152217	*SETBP1*	18	42260138	42648475	1	protein_coding	26040	*SETBP1*	0.999002929	-2.185354394	4	12.01	NA	rs11877152	45

ENSG00000132874	*SLC14A2*	18	42792960	43263072	1	protein_coding	8170	*SLC14A2*	8.42741E-14	0.719949743	4	1.751	0.000509007	rs11877152	45
ENSG00000088930	*XRN2*	20	21283942	21370463	1	protein_coding	22803	*XRN2*	0.813170186	-0.511318347	63	13.49	1.47451E-07	rs11697152;rs6132437;rs1885284;rs4426587;rs6137363;rs6132438;rs2180965	46
ENSG00000125816	*NKX2-4*	20	21376005	21378666	-1	protein_coding	644524	*NKX2-4*	0.278447692	-0.618232094	7	11.39	1.88082E-07	rs11697152;rs6132437;rs1885284;rs4426587;rs6137363;rs6132438;rs2180965	46
ENSG00000125820	*NKX2-2*	20	21491648	21494664	-1	protein_coding	4821	*NKX2-2*	0.337334272	0.403260334	5	15.42	0.000135399	rs11697152;rs1885284;rs4426587;rs6137363;rs6132437;rs6132438;rs2180965	46
ENSG00000101336	*HCK*	20	30639991	30689659	1	protein_coding	3055	*HCK*	NA	-0.488054047	18	8.449	2.01611E-06	rs6061182;rs6061195;rs2424874;rs2424877;rs2424878	47
ENSG00000101337	***TM9SF4***	20	30697309	30755061	1	protein_coding	9777	*TM9SF4*	0.999970772	-0.454321947	101	14.53	9.52979E-07	rs6061182;rs6061195;rs2424874;rs2424877;rs2424878	47
ENSG00000126003	***PLAGL2***	20	30780306	30795594	-1	protein_coding	5326	*PLAGL2*	0.869795638	-1.454407449	14	10.18	2.21643E-06	rs6061182;rs6061195;rs2424874;rs2424877;rs2424878	47
ENSG00000101346	*POFUT1*	20	30795683	30826470	1	protein_coding	23509	*POFUT1*	0.987586067	-0.933580252	18	10.18	2.21643E-06	rs6061182;rs6061195;rs2424874;rs2424877;rs2424878	47
ENSG00000101350	***KIF3B***	20	30865467	30922814	1	protein_coding	9371	*KIF3B*	0.299149041	1.216030949	79	12.8	1.25826E-06	rs6061182;rs6061195;rs2424874;rs2424877;rs2424878	47
ENSG00000171456	***ASXL1***	20	30946155	31027122	1	protein_coding	171023	*ASXL1*	1.3737E-18	-0.102032696	104	14.34	2.18884E-06	rs6061182;rs6061195;rs2424874;rs2424877;rs2424878	47
ENSG00000197183	***C20orf112***	20	31030862	31172876	-1	protein_coding	140688	*C20orf112*	0.973021414	-1.221048481	35	14.34	NA	rs6061182;rs6061195;rs2424874;rs2424877;rs2424878	47
ENSG00000020256	*ZFP64*	20	50668202	50820847	-1	protein_coding	55734	*ZFP64*	0.005189853	0.080832412	19	11.47	0.006898793	rs17794954	48
ENSG00000268856	*AP001579.1*	21	46492796	46492927	-1	protein_coding	NA	*NA*	NA	NA	6	11.98	0.001747425	rs400997;rs403694;rs427943;rs407133;rs372519;rs394872;rs395761;rs397092	49
ENSG00000197381	*ADARB1*	21	46493768	46646478	1	protein_coding	104	*ADARB1*	0.843939347	0.94107657	112	13.31	5.0836E-05	rs400997;rs403694;rs427943;rs407133;rs372519;rs394872;rs395761;rs397092	49
ENSG00000268805	*PRED57*	21	46511596	46515493	-1	protein_coding	728039	*SSR4P1*	NA	NA	3	11.44	0.002077283	rs400997;rs403694;rs427943;rs407133;rs372519;rs394872;rs395761;rs397092	49
ENSG00000267857	*PRED58*	21	46520728	46534873	-1	protein_coding	NA	*NA*	NA	NA	5	2.813	0.00184114	rs400997;rs403694;rs427943;rs407133;rs372519;rs394872;rs395761;rs397092	49
ENSG00000186866	*POFUT2*	21	46683843	46707813	-1	protein_coding	23275	*POFUT2*	0.010840872	-0.159699322	1	3.07	NA	rs427943;rs407133	49
