# Supplementary material for: Genetic neurodevelopmental clustering and dyslexia
Source: Mol Psychiatry. 2024 Jul 15;30(1):140–50. doi: 10.1038/s41380-024-02649-8 (PMC11649571; doi:10.1038/s41380-024-02649-8)
Supplement: Supplementary file 6 — Supplementary Table 6 [file 41380_2024_2649_MOESM6_ESM.pdf]

**Supplementary Table 6. Results of Gene Ontology enrichment analysis for 174 genes mapped to pleiotropic risk loci for dyslexia and ADHD.**

| source | term_name | term_id | highlighted | adjusted_p_value | negative_log10_of_adjusted_p_value | term_size | query_size | intersection_size | effective_domain_size | intersections |
|---|---|---|---|---|---|---|---|---|---|---|
| GO:MF | protein binding | GO:0005515 | TRUE | 2.77E-04 | 3.556851028 | 14881 | 158 | 142 | 20195 | ENSG00000066056,ENSG00000117400,ENSG00000117399,ENSG00000066322,ENSG00000159479,ENSG00000198198,ENSG00000178922,ENSG00000135951,ENSG00000185674,ENSG00000144214,ENSG00000115514,ENSG00000158417,ENSG00000135945,ENSG00000128655,ENSG00000155636,ENSG00000079156,ENSG00000065413,ENSG00000115524,ENSG00000144381,ENSG00000115541,ENSG00000115540,ENSG00000162944,ENSG00000152430,ENSG00000115896,ENSG00000168016,ENSG00000178567,ENSG00000076242,ENSG00000093167,ENSG00000144674,ENSG00000213672,ENSG00000068745,ENSG00000114302,ENSG00000178537,ENSG00000177479,ENSG00000178252,ENSG00000178149,ENSG00000178057,ENSG00000178035,ENSG00000198218,ENSG00000172053,ENSG00000172046,ENSG00000172037,ENSG00000185909,ENSG00000173421,ENSG00000188315,ENSG00000114316,ENSG00000233276,ENSG00000067560,ENSG00000145022,ENSG00000145020,ENSG00000145029,ENSG00000173402,ENSG00000164061,ENSG00000183763,ENSG00000164076,ENSG00000164078,ENSG00000164077,ENSG00000004534,ENSG00000003756,ENSG00000001617,ENSG00000114349,ENSG00000114353,ENSG00000179564,ENSG00000214706,ENSG00000186792,ENSG00000243477,ENSG00000114395,ENSG00000126062,ENSG00000185565,ENSG00000065534,ENSG00000175455,ENSG00000065371,ENSG00000073711,ENSG00000174579,ENSG00000114054,ENSG00000118007,ENSG00000168917,ENSG00000158092,ENSG00000174564,ENSG00000158186,ENSG00000158220,ENSG00000114107,ENSG00000158234,ENSG00000075711,ENSG00000184305,ENSG00000005108,ENSG00000158321,ENSG00000187416,ENSG00000005483,ENSG00000135250,ENSG00000133874,ENSG00000133878,ENSG00000156687,ENSG00000188938,ENSG00000197724,ENSG00000156395,ENSG00000086827,ENSG00000048028,ENSG00000181418,ENSG00000181929,ENSG00000167548,ENSG00000167550,ENSG00000139549,ENSG00000150967,ENSG00000111325,ENSG00000182196,ENSG00000090975,ENSG00000051825,ENSG00000130921,ENSG00000111328,ENSG00000139697,ENSG00000183955,ENSG00000150977,ENSG00000131966,ENSG00000100567,ENSG00000196860,ENSG00000100575,ENSG00000100578,ENSG00000166923,ENSG00000248905,ENSG00000137871,ENSG00000140262,ENSG00000136379,ENSG00000103888,ENSG00000103264,ENSG00000140941,ENSG00000140948,ENSG00000198795,ENSG00000152214,ENSG00000152217,ENSG00000132874,ENSG00000088930,ENSG00000125816,ENSG00000125820,ENSG00000101336,ENSG00000101337,ENSG00000126003,ENSG00000101350,ENSG00000171456,ENSG00000197183,ENSG00000020256,ENSG00000197381 |
| GO:MF | hyaluronoglucosaminidase activity | GO:0004415 | TRUE | 2.10E-02 | 1.678686586 | 8 | 158 | 3 | 20195 | ENSG00000186792,ENSG00000114378,ENSG00000103888 |
| GO:MF | peptide-O-fucosyltransferase activity | GO:0046922 | TRUE | 4.99E-02 | 1.302269301 | 2 | 158 | 2 | 20195 | ENSG00000101346,ENSG00000186866 |
| GO:BP | glycosaminoglycan catabolic process | GO:0006027 | TRUE | 1.95E-03 | 2.709160894 | 27 | 146 | 5 | 21110 | ENSG00000185674,ENSG00000144214,ENSG00000186792,ENSG00000114378,ENSG00000103888 |
| GO:BP | macromolecule modification | GO:0043412 | TRUE | 4.69E-03 | 2.329034241 | 3840 | 146 | 50 | 21110 | ENSG00000066056,ENSG00000117399,ENSG00000159479,ENSG00000115524,ENSG00000114302,ENSG00000177479,ENSG00000178467,ENSG00000178252,ENSG00000178149,ENSG00000172053,ENSG00000172046,ENSG00000114316,ENSG00000233276,ENSG00000067560,ENSG00000173402,ENSG00000183763,ENSG00000164076,ENSG00000164078,ENSG00000114353,ENSG00000243477,ENSG00000065534,ENSG00000065371,ENSG00000073711,ENSG00000174579,ENSG00000158092,ENSG00000075711,ENSG00000158321,ENSG00000005483,ENSG00000135250,ENSG00000172728,ENSG00000133874,ENSG00000133878,ENSG00000197724,ENSG00000048028,ENSG00000181929,ENSG00000167548,ENSG00000111328,ENSG00000183955,ENSG00000032219,ENSG00000166923,ENSG00000136379,ENSG00000103888,ENSG00000103264,ENSG00000152214,ENSG00000152217,ENSG00000101336,ENSG00000101346,ENSG00000171456,ENSG00000197381,ENSG00000186866 |
| GO:BP | protein modification process | GO:0036211 | FALSE | 4.82E-03 | 2.316898818 | 3617 | 146 | 48 | 21110 | ENSG00000066056,ENSG00000117399,ENSG00000159479,ENSG00000115524,ENSG00000114302,ENSG00000177479,ENSG00000178467,ENSG00000172053,ENSG00000172046,ENSG00000114316,ENSG00000233276,ENSG00000067560,ENSG00000173402,ENSG00000183763,ENSG00000164076,ENSG00000164078,ENSG00000114353,ENSG00000243477,ENSG00000065534,ENSG00000065371,ENSG00000073711,ENSG00000174579,ENSG00000158092,ENSG00000075711,ENSG00000158321,ENSG00000005483,ENSG00000135250,ENSG00000172728,ENSG00000133874,ENSG00000133878,ENSG00000197724,ENSG00000048028,ENSG00000181929,ENSG00000167548,ENSG00000111328,ENSG00000183955,ENSG00000032219,ENSG00000166923,ENSG00000136379,ENSG00000103888,ENSG00000103264,ENSG00000152214,ENSG00000152217,ENSG00000101336,ENSG00000101346,ENSG00000171456,ENSG00000197381,ENSG00000186866 |
| GO:BP | aminoglycan catabolic process | GO:0006026 | FALSE | 5.55E-03 | 2.255336548 | 33 | 146 | 5 | 21110 | ENSG00000185674,ENSG00000144214,ENSG00000186792,ENSG00000114378,ENSG00000103888 |
| GO:BP | developmental process | GO:0032502 | TRUE | 8.02E-03 | 2.09563908 | 6489 | 146 | 71 | 21110 | ENSG00000066056,ENSG00000117400,ENSG00000117399,ENSG00000066322,ENSG00000198198,ENSG00000135951,ENSG00000155636,ENSG00000138386,ENSG00000144381,ENSG00000115541,ENSG00000152430,ENSG00000076242,ENSG00000144674,ENSG00000213672,ENSG00000178537,ENSG00000177479,ENSG00000178467,ENSG00000178035,ENSG00000172053,ENSG00000172046,ENSG00000172037,ENSG00000173421,ENSG00000188315,ENSG00000233276,ENSG00000067560,ENSG00000145022,ENSG00000173402,ENSG00000164061,ENSG00000164078,ENSG00000001617,ENSG00000114349,ENSG00000186792,ENSG00000114378,ENSG00000175161,ENSG00000185565,ENSG00000065534,ENSG00000175455,ENSG00000065371,ENSG00000073711,ENSG00000158092,ENSG00000158234,ENSG00000075711,ENSG00000005108,ENSG00000158321,ENSG00000005483,ENSG00000135250,ENSG00000172728,ENSG00000156687,ENSG00000197724,ENSG00000181929,ENSG00000167548,ENSG00000139549,ENSG00000150977,ENSG00000032219,ENSG00000166923,ENSG00000248905,ENSG00000140262,ENSG00000103264,ENSG00000198795,ENSG00000152214,ENSG00000088930,ENSG00000125816,ENSG00000125820,ENSG00000101336,ENSG00000126003,ENSG00000101346,ENSG00000101350,ENSG00000171456,ENSG00000020256,ENSG00000197381,ENSG00000186866 |
| GO:BP | anatomical structure development | GO:0048856 | FALSE | 2.36E-02 | 1.627846756 | 5896 | 146 | 65 | 21110 | ENSG00000066056,ENSG00000117400,ENSG00000117399,ENSG00000066322,ENSG00000198198,ENSG00000155636,ENSG00000138386,ENSG00000144381,ENSG00000076242,ENSG00000144674,ENSG00000213672,ENSG00000178537,ENSG00000177479,ENSG00000178467,ENSG00000178035,ENSG00000172053,ENSG00000172046,ENSG00000172037,ENSG00000173421,ENSG00000233276,ENSG00000067560,ENSG00000145022,ENSG00000173402,ENSG00000164061,ENSG00000164078,ENSG00000001617,ENSG00000114349,ENSG00000186792,ENSG00000114378,ENSG00000175161,ENSG00000185565,ENSG00000065534,ENSG00000175455,ENSG00000065371,ENSG00000073711,ENSG00000158092,ENSG00000158234,ENSG00000075711,ENSG00000005108,ENSG00000158321,ENSG00000005483,ENSG00000135250,ENSG00000172728,ENSG00000156687,ENSG00000197724,ENSG00000167548,ENSG00000139549,ENSG00000150977,ENSG00000032219,ENSG00000166923,ENSG00000248905,ENSG00000140262,ENSG00000103264,ENSG00000198795,ENSG00000152214,ENSG00000088930,ENSG00000125820,ENSG00000101336,ENSG00000126003,ENSG00000101346,ENSG00000101350,ENSG00000171456,ENSG00000020256,ENSG00000197381,ENSG00000186866 |
| GO:BP | multicellular organism development | GO:0007275 | FALSE | 2.42E-02 | 1.617023449 | 4658 | 146 | 55 | 21110 | ENSG00000066056,ENSG00000117400,ENSG00000117399,ENSG00000198198,ENSG00000155636,ENSG00000138386,ENSG00000144381,ENSG00000076242,ENSG00000144674,ENSG00000213672,ENSG00000178537,ENSG00000178467,ENSG00000178035,ENSG00000172053,ENSG00000172037,ENSG00000233276,ENSG00000067560,ENSG00000145022,ENSG00000173402,ENSG00000164061,ENSG00000164078,ENSG00000001617,ENSG00000114349,ENSG00000186792,ENSG00000114378,ENSG00000175161,ENSG00000185565,ENSG00000065534,ENSG00000175455,ENSG00000073711,ENSG00000158092,ENSG00000158234,ENSG00000075711,ENSG00000005108,ENSG00000158321,ENSG00000135250,ENSG00000172728,ENSG00000156687,ENSG00000197724,ENSG00000139549,ENSG00000032219,ENSG00000166923,ENSG00000248905,ENSG00000140262,ENSG00000103264,ENSG00000198795,ENSG00000152214,ENSG00000088930,ENSG00000125820,ENSG00000126003,ENSG00000101346,ENSG00000101350,ENSG00000171456,ENSG00000197381,ENSG00000186866 |
| TF | Factor: ETF; motif: GVGGMGG | TF:M00695 | FALSE | 1.22E-04 | 3.914956249 | 10683 | 165 | 122 | 20011 | ENSG00000117399,ENSG00000066322,ENSG00000159479,ENSG00000198198,ENSG00000178922,ENSG00000196872,ENSG00000135951,ENSG00000241962,ENSG00000158417,ENSG00000135945,ENSG00000128655,ENSG00000155636,ENSG00000079156,ENSG00000138386,ENSG00000065413,ENSG00000115524,ENSG00000144381,ENSG00000115541,ENSG00000270757,ENSG00000152430,ENSG00000115896,ENSG00000168016,ENSG00000178567,ENSG00000076242,ENSG00000093167,ENSG00000144674,ENSG00000213672,ENSG00000114302,ENSG00000178537,ENSG00000177479,ENSG00000178467,ENSG00000178252,ENSG00000178035,ENSG00000198218,ENSG00000172046,ENSG00000172037,ENSG00000177352,ENSG00000188315,ENSG00000233276,ENSG00000067560,ENSG00000145022,ENSG00000145029,ENSG00000173402,ENSG00000164061,ENSG00000164078,ENSG00000004534,ENSG00000003756,ENSG00000114353,ENSG00000214706,ENSG00000186792,ENSG00000243477,ENSG00000114395,ENSG00000272104,ENSG00000126062,ENSG00000007402,ENSG00000175161,ENSG00000065534,ENSG00000065371,ENSG00000073711,ENSG00000174579,ENSG00000114054,ENSG00000118007,ENSG00000168917,ENSG00000158092,ENSG00000158186,ENSG00000158220,ENSG00000114107,ENSG00000075711,ENSG00000184305,ENSG00000005108,ENSG00000158321,ENSG00000187416,ENSG00000005483,ENSG00000135250,ENSG00000133874,ENSG00000133878,ENSG00000156687,ENSG00000164989,ENSG00000188938,ENSG00000048828,ENSG00000197724,ENSG00000156395,ENSG00000086827,ENSG00000048028,ENSG00000181418,ENSG00000181929,ENSG00000167548,ENSG00000167550,ENSG00000150967,ENSG00000111325,ENSG00000182196,ENSG00000090975,ENSG00000051825,ENSG00000111328,ENSG00000139697,ENSG00000183955,ENSG00000139971,ENSG00000131966,ENSG00000032219,ENSG00000196860,ENSG00000100575,ENSG00000100578,ENSG00000166923,ENSG00000137871,ENSG00000140262,ENSG00000136379,ENSG00000103888,ENSG00000103264,ENSG00000140941,ENSG00000140948,ENSG00000198795,ENSG00000088930,ENSG00000125816,ENSG00000101336,ENSG00000126003,ENSG00000101346,ENSG00000101350,ENSG00000171456,ENSG00000197183,ENSG00000020256,ENSG00000197381,ENSG00000186866 |

| | | | | | | | | | | |
|---|---|---|---|---|---|---|---|---|---|---|
| TF | Factor: E2F-2; motif: NWTTTGGCGCCAWWNN; match class: 1 | TF:M11530_1 | FALSE | 6.11E-04 | 3.213933363 | 13761 | 165 | 142 | 20011 | ENSG00000253313,ENSG00000066056,ENSG00000117399,ENSG00000066322,ENSG00000159479,ENSG00000198198,ENSG00000178922,ENSG00000196872,ENSG00000135951,ENSG00000241962,ENSG00000144214,ENSG00000115514,ENSG00000135945,ENSG00000128655,ENSG00000155636,ENSG00000079156,ENSG00000138386,ENSG00000065413,ENSG00000115524,ENSG00000115520,ENSG00000144381,ENSG00000115541,ENSG00000270757,ENSG00000115540,ENSG00000247626,ENSG00000152430,ENSG00000115896,ENSG00000168016,ENSG00000178567,ENSG00000076242,ENSG00000093167,ENSG00000144674,ENSG00000213672,ENSG00000114302,ENSG00000178537,ENSG00000177479,ENSG00000178467,ENSG00000178252,ENSG00000178149,ENSG00000178057,ENSG00000178035,ENSG00000198218,ENSG00000172046,ENSG00000172037,ENSG00000177352,ENSG00000185909,ENSG00000173421,ENSG00000114316,ENSG00000233276,ENSG00000067560,ENSG00000145022,ENSG00000145029,ENSG00000173402,ENSG00000164061,ENSG00000183763,ENSG00000164076,ENSG00000164078,ENSG00000164077,ENSG00000004534,ENSG00000003756,ENSG00000114349,ENSG00000114353,ENSG00000179564,ENSG00000214706,ENSG00000186792,ENSG00000243477,ENSG00000114378,ENSG00000114395,ENSG00000272104,ENSG00000126062,ENSG00000007402,ENSG00000065534,ENSG00000175455,ENSG00000065371,ENSG00000073711,ENSG00000174579,ENSG00000118007,ENSG00000168917,ENSG00000158092,ENSG00000158186,ENSG00000158220,ENSG00000114107,ENSG00000158234,ENSG00000075711,ENSG00000184305,ENSG00000005108,ENSG00000158321,ENSG00000187416,ENSG00000005483,ENSG00000135250,ENSG00000172728,ENSG00000133874,ENSG00000133878,ENSG00000156687,ENSG00000048828,ENSG00000197724,ENSG00000156395,ENSG00000086827,ENSG00000048028,ENSG00000181418,ENSG00000181929,ENSG00000167548,ENSG00000167550,ENSG00000139549,ENSG00000150967,ENSG00000111325,ENSG00000182196,ENSG00000090975,ENSG00000051825,ENSG00000130921,ENSG00000111328,ENSG00000139697,ENSG00000183955,ENSG00000150977,ENSG00000139971,ENSG00000131966,ENSG00000100567,ENSG00000032219,ENSG00000196860,ENSG00000100575,ENSG00000100578,ENSG00000166923,ENSG00000140262,ENSG00000136379,ENSG00000103888,ENSG00000103264,ENSG00000140941,ENSG00000140948,ENSG00000198795,ENSG00000132874,ENSG00000088930,ENSG00000125816,ENSG00000125820,ENSG00000101336,ENSG00000101337,ENSG00000126003,ENSG00000101346,ENSG00000101350,ENSG00000171456,ENSG00000020256,ENSG00000197381,ENSG00000186866 |
| TF | Factor: E2F-2; motif: NWTTTGGCGCCAWWNN | TF:M11530 | FALSE | 2.65E-03 | 2.577193037 | 14927 | 165 | 148 | 20011 | ENSG00000253313,ENSG00000066056,ENSG00000117399,ENSG00000066322,ENSG00000159479,ENSG00000198198,ENSG00000178922,ENSG00000196872,ENSG00000135951,ENSG00000241962,ENSG00000144214,ENSG00000115514,ENSG00000158417,ENSG00000135945,ENSG00000128655,ENSG00000155636,ENSG00000079156,ENSG00000138386,ENSG00000065413,ENSG00000115524,ENSG00000115520,ENSG00000144381,ENSG00000115541,ENSG00000270757,ENSG00000115540,ENSG00000247626,ENSG00000152430,ENSG00000115896,ENSG00000168016,ENSG00000178567,ENSG00000076242,ENSG00000093167,ENSG00000144674,ENSG00000213672,ENSG00000114302,ENSG00000178537,ENSG00000177479,ENSG00000178467,ENSG00000178252,ENSG00000178149,ENSG00000178057,ENSG00000178035,ENSG00000198218,ENSG00000172053,ENSG00000172046,ENSG00000172037,ENSG00000177352,ENSG00000185909,ENSG00000173421,ENSG00000114316,ENSG00000233276,ENSG00000067560,ENSG00000145022,ENSG00000145029,ENSG00000173402,ENSG00000164061,ENSG00000183763,ENSG00000164076,ENSG00000164078,ENSG00000164077,ENSG00000004534,ENSG00000003756,ENSG00000001617,ENSG00000114349,ENSG00000114353,ENSG00000179564,ENSG00000214706,ENSG00000186792,ENSG00000243477,ENSG00000114378,ENSG00000114395,ENSG00000272104,ENSG00000126062,ENSG00000007402,ENSG00000175161,ENSG00000185565,ENSG00000065534,ENSG00000175455,ENSG00000065371,ENSG00000073711,ENSG00000174579,ENSG00000118007,ENSG00000168917,ENSG00000158092,ENSG00000158186,ENSG00000158220,ENSG00000114107,ENSG00000158234,ENSG00000075711,ENSG00000184305,ENSG00000005108,ENSG00000158321,ENSG00000187416,ENSG00000005483,ENSG00000135250,ENSG00000172728,ENSG00000133874,ENSG00000133878,ENSG00000156687,ENSG00000048828,ENSG00000197724,ENSG00000156395,ENSG00000086827,ENSG00000048028,ENSG00000181418,ENSG00000181929,ENSG00000167548,ENSG00000167550,ENSG00000139549,ENSG00000150967,ENSG00000111325,ENSG00000182196,ENSG00000090975,ENSG00000051825,ENSG00000130921,ENSG00000111328,ENSG00000139697,ENSG00000183955,ENSG00000150977,ENSG00000139971,ENSG00000131966,ENSG00000100567,ENSG00000032219,ENSG00000196860,ENSG00000100575,ENSG00000100578,ENSG00000166923,ENSG00000140262,ENSG00000136379,ENSG00000103888,ENSG00000103264,ENSG00000140941,ENSG00000140948,ENSG00000198795,ENSG00000132874,ENSG00000088930,ENSG00000125816,ENSG00000125820,ENSG00000101336,ENSG00000101337,ENSG00000126003,ENSG00000101346,ENSG00000101350,ENSG00000171456,ENSG00000197183,ENSG00000020256,ENSG00000197381,ENSG00000186866 |
| TF | Factor: NRF-1; motif: SYGCGCMTGCGCRNNGSN | TF:M09641 | FALSE | 3.94E-03 | 2.404473831 | 3303 | 165 | 52 | 20011 | ENSG00000066322,ENSG00000196872,ENSG00000135951,ENSG00000241962,ENSG00000115514,ENSG00000158417,ENSG00000135945,ENSG00000079156,ENSG00000115524,ENSG00000115520,ENSG00000144381,ENSG00000115541,ENSG00000270757,ENSG00000247626,ENSG00000178567,ENSG00000076242,ENSG00000144674,ENSG00000213672,ENSG00000114302,ENSG00000188315,ENSG00000067560,ENSG00000145022,ENSG00000173402,ENSG00000164061,ENSG00000164077,ENSG00000186792,ENSG00000243477,ENSG00000114395,ENSG00000272104,ENSG00000126062,ENSG00000114054,ENSG00000118007,ENSG00000168917,ENSG00000158186,ENSG00000158321,ENSG00000187416,ENSG00000005483,ENSG00000135250,ENSG00000133874,ENSG00000164989,ENSG00000156395,ENSG00000048028,ENSG00000167550,ENSG00000182196,ENSG00000051825,ENSG00000139697,ENSG00000150977,ENSG00000032219,ENSG00000166923,ENSG00000103264,ENSG00000140941,ENSG00000186866 |
| TF | Factor: LHX9; motif: NTCGTTAN | TF:M10974 | FALSE | 4.94E-03 | 2.306689582 | 259 | 165 | 12 | 20011 | ENSG00000115514,ENSG00000155636,ENSG00000115540,ENSG00000093167,ENSG00000067560,ENSG00000145022,ENSG00000150967,ENSG00000111325,ENSG00000150977,ENSG00000103264,ENSG00000140941,ENSG00000101337 |
| TF | Factor: lhx6; motif: CTCGTTAR | TF:M10980 | FALSE | 4.94E-03 | 2.306689582 | 259 | 165 | 12 | 20011 | ENSG00000115514,ENSG00000155636,ENSG00000115540,ENSG00000093167,ENSG00000067560,ENSG00000145022,ENSG00000150967,ENSG00000111325,ENSG00000150977,ENSG00000103264,ENSG00000140941,ENSG00000101337 |
| TF | Factor: E2F-1; motif: WWTGGCGCCAAA; match class: 1 | TF:M04515_1 | FALSE | 1.19E-02 | 1.923220363 | 12480 | 165 | 130 | 20011 | ENSG00000253313,ENSG00000117399,ENSG00000066322,ENSG00000178922,ENSG00000196872,ENSG00000135951,ENSG00000241962,ENSG00000135945,ENSG00000128655,ENSG00000155636,ENSG00000079156,ENSG00000138386,ENSG00000065413,ENSG00000115524,ENSG00000115520,ENSG00000144381,ENSG00000115541,ENSG00000270757,ENSG00000115540,ENSG00000247626,ENSG00000152430,ENSG00000115896,ENSG00000178567,ENSG00000076242,ENSG00000093167,ENSG00000144674,ENSG00000213672,ENSG00000178537,ENSG00000177479,ENSG00000178467,ENSG00000178252,ENSG00000178149,ENSG00000178057,ENSG00000178035,ENSG00000198218,ENSG00000172046,ENSG00000172037,ENSG00000177352,ENSG00000185909,ENSG00000173421,ENSG00000114316,ENSG00000233276,ENSG00000067560,ENSG00000145022,ENSG00000173402,ENSG00000164061,ENSG00000164076,ENSG00000164078,ENSG00000164077,ENSG00000004534,ENSG00000003756,ENSG00000114353,ENSG00000179564,ENSG00000214706,ENSG00000186792,ENSG00000243477,ENSG00000114378,ENSG00000114395,ENSG00000272104,ENSG00000126062,ENSG00000007402,ENSG00000175161,ENSG00000065534,ENSG00000065371,ENSG00000073711,ENSG00000174579,ENSG00000118007,ENSG00000168917,ENSG00000158092,ENSG00000158186,ENSG00000158220,ENSG00000075711,ENSG00000184305,ENSG00000005108,ENSG00000158321,ENSG00000187416,ENSG00000005483,ENSG00000135250,ENSG00000172728,ENSG00000133874,ENSG00000133878,ENSG00000156687,ENSG00000048828,ENSG00000197724,ENSG00000156395,ENSG00000086827,ENSG00000048028,ENSG00000181418,ENSG00000181929,ENSG00000167548,ENSG00000167550,ENSG00000139549,ENSG00000150967,ENSG00000111325,ENSG00000182196,ENSG00000090975,ENSG00000051825,ENSG00000130921,ENSG00000111328,ENSG00000139697,ENSG00000183955,ENSG00000150977,ENSG00000139971,ENSG00000100567,ENSG00000032219,ENSG00000196860,ENSG00000100575,ENSG00000100578,ENSG00000166923,ENSG00000140262,ENSG00000136379,ENSG00000103888,ENSG00000103264,ENSG00000140941,ENSG00000140948,ENSG00000198795,ENSG00000132874,ENSG00000088930,ENSG00000125816,ENSG00000125820,ENSG00000101336,ENSG00000101337,ENSG00000126003,ENSG00000101346,ENSG00000101350,ENSG00000171456,ENSG00000197183,ENSG00000020256,ENSG00000197381,ENSG00000186866 |
| TF | Factor: E2F-2; motif: GCGCGCGCGYW; match class: 1 | TF:M11531_1 | FALSE | 1.37E-02 | 1.862066615 | 12643 | 165 | 131 | 20011 | ENSG00000253313,ENSG00000117399,ENSG00000066322,ENSG00000159479,ENSG00000198198,ENSG00000178922,ENSG00000196872,ENSG00000135951,ENSG00000241962,ENSG00000115514,ENSG00000158417,ENSG00000135945,ENSG00000079156,ENSG00000138386,ENSG00000065413,ENSG00000115524,ENSG00000115520,ENSG00000144381,ENSG00000115541,ENSG00000270757,ENSG00000115540,ENSG00000247626,ENSG00000152430,ENSG00000115896,ENSG00000168016,ENSG00000178567,ENSG00000076242,ENSG00000093167,ENSG00000144674,ENSG00000213672,ENSG00000068745,ENSG00000114302,ENSG00000177479,ENSG00000178467,ENSG00000178252,ENSG00000178149,ENSG00000178057,ENSG00000178035,ENSG00000198218,ENSG00000172053,ENSG00000172046,ENSG00000172037,ENSG00000177352,ENSG00000173421,ENSG00000188315,ENSG00000114316,ENSG00000233276,ENSG00000067560,ENSG00000145022,ENSG00000145029,ENSG00000173402,ENSG00000164061,ENSG00000183763,ENSG00000164076,ENSG00000164078,ENSG00000004534,ENSG00000003756,ENSG00000214706,ENSG00000186792,ENSG00000243477,ENSG00000114395,ENSG00000272104,ENSG00000126062,ENSG00000007402,ENSG00000175161,ENSG00000185565,ENSG00000065534,ENSG00000065371,ENSG00000073711,ENSG00000174579,ENSG00000114054,ENSG00000118007,ENSG00000168917,ENSG00000158092,ENSG00000158186,ENSG00000158220,ENSG00000075711,ENSG00000184305,ENSG00000005108,ENSG00000158321,ENSG00000187416,ENSG00000005483,ENSG00000135250,ENSG00000172728,ENSG00000133874,ENSG00000133878,ENSG00000156687,ENSG00000164989,ENSG00000048828,ENSG00000197724,ENSG00000156395,ENSG00000086827,ENSG00000048028,ENSG00000181418,ENSG00000167548,ENSG00000167550,ENSG00000150967,ENSG00000111325,ENSG00000182196,ENSG00000090975,ENSG00000051825,ENSG00000111328,ENSG00000139697,ENSG00000183955,ENSG00000150977,ENSG00000139971,ENSG00000131966,ENSG00000032219,ENSG00000196860,ENSG00000166923,ENSG00000137871,ENSG00000140262,ENSG00000136379,ENSG00000103888,ENSG00000103264,ENSG00000140941,ENSG00000140948,ENSG00000198795,ENSG00000088930,ENSG00000125816,ENSG00000125820,ENSG00000101336,ENSG00000101337,ENSG00000126003,ENSG00000101346,ENSG00000101350,ENSG00000171456,ENSG00000197183,ENSG00000020256,ENSG00000197381,ENSG00000186866 |
| TF | Factor: LHX8; motif: CTCGTTAN | TF:M10985 | FALSE | 2.05E-02 | 1.688453063 | 522 | 165 | 16 | 20011 | ENSG00000253313,ENSG00000115514,ENSG00000155636,ENSG00000115540,ENSG00000093167,ENSG00000067560,ENSG00000145022,ENSG00000158186,ENSG00000149305,ENSG00000150967,ENSG00000111325,ENSG00000111328,ENSG00000150977,ENSG00000103264,ENSG00000140941,ENSG00000101337 |

| | | | | | | | | | | |
|---|---|---|---|---|---|---|---|---|---|---|
| TF | Factor: TCF-1; motif: ACATCGRGRCGCTGW; match class: 1 | TF:M11603_1 | FALSE | 2.95E-02 | 1.530484642 | 13482 | 165 | 136 | 20011 | ENSG00000253313,ENSG00000117399,ENSG00000066322,ENSG00000196872,ENSG00000135951,ENSG00000241962,ENSG00000144214,ENSG00000115514,ENSG00000158417,ENSG00000135945,ENSG00000128655,ENSG00000155636,ENSG00000079156,ENSG00000138386,ENSG00000065413,ENSG00000115524,ENSG00000144381,ENSG00000115541,ENSG00000270757,ENSG00000115540,ENSG00000247626,ENSG00000152430,ENSG00000115896,ENSG00000168016,ENSG00000178567,ENSG00000076242,ENSG00000093167,ENSG00000144674,ENSG00000213672,ENSG00000068745,ENSG00000114302,ENSG00000178537,ENSG00000177479,ENSG00000178467,ENSG00000178252,ENSG00000178149,ENSG00000178057,ENSG00000178035,ENSG00000198218,ENSG00000172053,ENSG00000172046,ENSG00000172037,ENSG00000177352,ENSG00000173421,ENSG00000188315,ENSG00000114316,ENSG00000233276,ENSG00000067560,ENSG00000145022,ENSG00000173402,ENSG00000164061,ENSG00000183763,ENSG00000164076,ENSG00000164078,ENSG00000164077,ENSG00000004534,ENSG00000003756,ENSG00000214706,ENSG00000186792,ENSG00000243477,ENSG00000114395,ENSG00000272104,ENSG00000126062,ENSG00000007402,ENSG00000175161,ENSG00000185565,ENSG00000065534,ENSG00000065371,ENSG00000073711,ENSG00000174579,ENSG00000114054,ENSG00000118007,ENSG00000168917,ENSG00000158092,ENSG00000158186,ENSG00000158220,ENSG00000114107,ENSG00000075711,ENSG00000184305,ENSG00000005108,ENSG00000158321,ENSG00000187416,ENSG00000005483,ENSG00000135250,ENSG00000172728,ENSG00000133874,ENSG00000133878,ENSG00000156687,ENSG00000188938,ENSG00000048828,ENSG00000197724,ENSG00000156395,ENSG00000086827,ENSG00000048028,ENSG00000181418,ENSG00000181929,ENSG00000167548,ENSG00000167550,ENSG00000139549,ENSG00000150967,ENSG00000111325,ENSG00000182196,ENSG00000090975,ENSG00000111328,ENSG00000139697,ENSG00000183955,ENSG00000150977,ENSG00000139971,ENSG00000131966,ENSG00000100567,ENSG00000032219,ENSG00000196860,ENSG00000100575,ENSG00000100578,ENSG00000166923,ENSG00000137871,ENSG00000140262,ENSG00000136379,ENSG00000103888,ENSG00000103264,ENSG00000140941,ENSG00000140948,ENSG00000198795,ENSG00000088930,ENSG00000125816,ENSG00000125820,ENSG00000101336,ENSG00000101337,ENSG00000126003,ENSG00000101346,ENSG00000101350,ENSG00000171456,ENSG00000197183,ENSG00000020256,ENSG00000197381,ENSG00000186866 |
| TF | Factor: E2F-1; motif: WWTGGCGCCAAA | TF:M04515 | FALSE | 3.58E-02 | 1.44565023 | 13660 | 165 | 137 | 20011 | ENSG00000253313,ENSG00000117399,ENSG00000066322,ENSG00000159479,ENSG00000198198,ENSG00000178922,ENSG00000196872,ENSG00000135951,ENSG00000241962,ENSG00000135945,ENSG00000128655,ENSG00000155636,ENSG00000079156,ENSG00000138386,ENSG00000065413,ENSG00000115524,ENSG00000115520,ENSG00000144381,ENSG00000115541,ENSG00000270757,ENSG00000115540,ENSG00000247626,ENSG00000152430,ENSG00000115896,ENSG00000168016,ENSG00000178567,ENSG00000076242,ENSG00000093167,ENSG00000144674,ENSG00000213672,ENSG00000178537,ENSG00000177479,ENSG00000178467,ENSG00000178252,ENSG00000178149,ENSG00000178057,ENSG00000178035,ENSG00000198218,ENSG00000172053,ENSG00000172046,ENSG00000172037,ENSG00000177352,ENSG00000185909,ENSG00000173421,ENSG00000114316,ENSG00000233276,ENSG00000067560,ENSG00000145022,ENSG00000173402,ENSG00000164061,ENSG00000164076,ENSG00000164078,ENSG00000164077,ENSG00000004534,ENSG00000003756,ENSG00000114353,ENSG00000179564,ENSG00000214706,ENSG00000186792,ENSG00000243477,ENSG00000114378,ENSG00000114395,ENSG00000272104,ENSG00000126062,ENSG00000007402,ENSG00000175161,ENSG00000065534,ENSG00000065371,ENSG00000073711,ENSG00000174579,ENSG00000118007,ENSG00000168917,ENSG00000158092,ENSG00000158186,ENSG00000158220,ENSG00000114107,ENSG00000075711,ENSG00000184305,ENSG00000005108,ENSG00000158321,ENSG00000187416,ENSG00000005483,ENSG00000135250,ENSG00000172728,ENSG00000133874,ENSG00000133878,ENSG00000156687,ENSG00000048828,ENSG00000197724,ENSG00000156395,ENSG00000086827,ENSG00000048028,ENSG00000181418,ENSG00000181929,ENSG00000167548,ENSG00000167550,ENSG00000139549,ENSG00000150967,ENSG00000111325,ENSG00000182196,ENSG00000090975,ENSG00000051825,ENSG00000130921,ENSG00000111328,ENSG00000139697,ENSG00000183955,ENSG00000150977,ENSG00000139971,ENSG00000131966,ENSG00000100567,ENSG00000032219,ENSG00000196860,ENSG00000100575,ENSG00000100578,ENSG00000166923,ENSG00000248905,ENSG00000140262,ENSG00000136379,ENSG00000103888,ENSG00000103264,ENSG00000140941,ENSG00000140948,ENSG00000198795,ENSG00000132874,ENSG00000088930,ENSG00000125816,ENSG00000125820,ENSG00000101336,ENSG00000101337,ENSG00000126003,ENSG00000101346,ENSG00000101350,ENSG00000171456,ENSG00000197183,ENSG00000020256,ENSG00000197381,ENSG00000186866 |
| TF | Factor: E2F-1; motif: NTTTTGGCGCCAWWWN | TF:M11533 | FALSE | 4.75E-02 | 1.323553542 | 13421 | 165 | 135 | 20011 | ENSG00000253313,ENSG00000066056,ENSG00000117399,ENSG00000066322,ENSG00000159479,ENSG00000198198,ENSG00000178922,ENSG00000196872,ENSG00000135951,ENSG00000241962,ENSG00000144214,ENSG00000135945,ENSG00000128655,ENSG00000155636,ENSG00000079156,ENSG00000138386,ENSG00000065413,ENSG00000115524,ENSG00000144381,ENSG00000115541,ENSG00000270757,ENSG00000115540,ENSG00000247626,ENSG00000152430,ENSG00000115896,ENSG00000168016,ENSG00000178567,ENSG00000076242,ENSG00000093167,ENSG00000144674,ENSG00000213672,ENSG00000178537,ENSG00000177479,ENSG00000178467,ENSG00000178149,ENSG00000178057,ENSG00000178035,ENSG00000198218,ENSG00000172053,ENSG00000172046,ENSG00000172037,ENSG00000177352,ENSG00000185909,ENSG00000173421,ENSG00000188315,ENSG00000114316,ENSG00000233276,ENSG00000067560,ENSG00000145022,ENSG00000145029,ENSG00000173402,ENSG00000164061,ENSG00000164076,ENSG00000164078,ENSG00000164077,ENSG00000004534,ENSG00000003756,ENSG00000001617,ENSG00000114353,ENSG00000214706,ENSG00000186792,ENSG00000243477,ENSG00000114395,ENSG00000272104,ENSG00000126062,ENSG00000007402,ENSG00000175161,ENSG00000065534,ENSG00000175455,ENSG00000065371,ENSG00000073711,ENSG00000174579,ENSG00000118007,ENSG00000168917,ENSG00000158092,ENSG00000158186,ENSG00000158220,ENSG00000114107,ENSG00000075711,ENSG00000184305,ENSG00000005108,ENSG00000158321,ENSG00000187416,ENSG00000005483,ENSG00000135250,ENSG00000172728,ENSG00000133874,ENSG00000133878,ENSG00000156687,ENSG00000188938,ENSG00000048828,ENSG00000197724,ENSG00000156395,ENSG00000086827,ENSG00000048028,ENSG00000181418,ENSG00000181929,ENSG00000167548,ENSG00000139549,ENSG00000150967,ENSG00000111325,ENSG00000182196,ENSG00000051825,ENSG00000130921,ENSG00000111328,ENSG00000139697,ENSG00000183955,ENSG00000150977,ENSG00000139971,ENSG00000100567,ENSG00000032219,ENSG00000196860,ENSG00000100575,ENSG00000100578,ENSG00000166923,ENSG00000248905,ENSG00000140262,ENSG00000136379,ENSG00000103264,ENSG00000140941,ENSG00000140948,ENSG00000198795,ENSG00000132874,ENSG00000088930,ENSG00000125816,ENSG00000125820,ENSG00000101336,ENSG00000101337,ENSG00000126003,ENSG00000101346,ENSG00000101350,ENSG00000171456,ENSG00000020256,ENSG00000197381,ENSG00000186866 |
| HPA | parathyroid gland; glandular cells[тЙеMedium] | HPA:0360052 | FALSE | 7.48E-04 | 3.125995252 | 4410 | 106 | 66 | 11006 | ENSG00000066322,ENSG00000159479,ENSG00000198198,ENSG00000178922,ENSG00000196872,ENSG00000115514,ENSG00000158417,ENSG00000128655,ENSG00000155636,ENSG00000115524,ENSG00000115520,ENSG00000144381,ENSG00000115541,ENSG00000270757,ENSG00000115540,ENSG00000115896,ENSG00000168016,ENSG00000076242,ENSG00000144674,ENSG00000178537,ENSG00000178467,ENSG00000178252,ENSG00000178149,ENSG00000178057,ENSG00000178035,ENSG00000198218,ENSG00000172053,ENSG00000185909,ENSG00000114316,ENSG00000233276,ENSG00000145020,ENSG00000183763,ENSG00000164078,ENSG00000004534,ENSG00000003756,ENSG00000114353,ENSG00000214706,ENSG00000243477,ENSG00000114395,ENSG00000114054,ENSG00000118007,ENSG00000158092,ENSG00000158186,ENSG00000114107,ENSG00000158234,ENSG00000075711,ENSG00000188938,ENSG00000048828,ENSG00000197724,ENSG00000149305,ENSG00000167548,ENSG00000150967,ENSG00000111325,ENSG00000182196,ENSG00000090975,ENSG00000051825,ENSG00000111328,ENSG00000139697,ENSG00000139971,ENSG00000100567,ENSG00000100575,ENSG00000248905,ENSG00000103264,ENSG00000198795,ENSG00000088930,ENSG00000101337 |
| HPA | pancreas; pancreatic endocrine cells[тЙеLow] | HPA:0351181 | FALSE | 1.07E-03 | 2.970348969 | 5617 | 106 | 77 | 11006 | ENSG00000066322,ENSG00000159479,ENSG00000178922,ENSG00000196872,ENSG00000115514,ENSG00000158417,ENSG00000128655,ENSG00000155636,ENSG00000115524,ENSG00000115520,ENSG00000144381,ENSG00000115541,ENSG00000270757,ENSG00000115540,ENSG00000115896,ENSG00000168016,ENSG00000076242,ENSG00000144674,ENSG00000068745,ENSG00000178537,ENSG00000178467,ENSG00000178252,ENSG00000178149,ENSG00000178057,ENSG00000178035,ENSG00000198218,ENSG00000172053,ENSG00000185909,ENSG00000114316,ENSG00000233276,ENSG00000067560,ENSG00000145022,ENSG00000145020,ENSG00000183763,ENSG00000164078,ENSG00000004534,ENSG00000003756,ENSG00000001617,ENSG00000114353,ENSG00000214706,ENSG00000243477,ENSG00000114395,ENSG00000126062,ENSG00000114054,ENSG00000118007,ENSG00000168917,ENSG00000158092,ENSG00000114107,ENSG00000158234,ENSG00000075711,ENSG00000158321,ENSG00000188938,ENSG00000048828,ENSG00000197724,ENSG00000086827,ENSG00000048028,ENSG00000149305,ENSG00000167548,ENSG00000111325,ENSG00000182196,ENSG00000090975,ENSG00000051825,ENSG00000111328,ENSG00000139697,ENSG00000150977,ENSG00000139971,ENSG00000100567,ENSG00000100575,ENSG00000137871,ENSG00000140262,ENSG00000103264,ENSG00000088930,ENSG00000125820,ENSG00000101337,ENSG00000101346,ENSG00000101350,ENSG00000197183 |
| HPA | prostate; glandular cells[тЙеMedium] | HPA:0390052 | FALSE | 2.10E-02 | 1.678218204 | 4806 | 106 | 66 | 11006 | ENSG00000159479,ENSG00000198198,ENSG00000178922,ENSG00000196872,ENSG00000115514,ENSG00000158417,ENSG00000128655,ENSG00000155636,ENSG00000115524,ENSG00000115520,ENSG00000144381,ENSG00000115541,ENSG00000270757,ENSG00000115540,ENSG00000115896,ENSG00000076242,ENSG00000144674,ENSG00000068745,ENSG00000178467,ENSG00000178149,ENSG00000178057,ENSG00000178035,ENSG00000198218,ENSG00000172053,ENSG00000185909,ENSG00000233276,ENSG00000145022,ENSG00000145020,ENSG00000164078,ENSG00000004534,ENSG00000003756,ENSG00000114353,ENSG00000214706,ENSG00000243477,ENSG00000114395,ENSG00000126062,ENSG00000114054,ENSG00000118007,ENSG00000158092,ENSG00000158186,ENSG00000114107,ENSG00000158234,ENSG00000075711,ENSG00000188938,ENSG00000048828,ENSG00000197724,ENSG00000086827,ENSG00000048028,ENSG00000149305,ENSG00000150967,ENSG00000111325,ENSG00000182196,ENSG00000090975,ENSG00000051825,ENSG00000130921,ENSG00000111328,ENSG00000139697,ENSG00000150977,ENSG00000139971,ENSG00000100575,ENSG00000248905,ENSG00000137871,ENSG00000140262,ENSG00000198795,ENSG00000088930,ENSG00000101337 |

| | | | | | | | | | | |
|---|---|---|---|---|---|---|---|---|---|---|
| HPA | endometrium 1; glandular cells[тЙeMedium] | HPA:0160052 | FALSE | 2.53E-02 | 1.597140782 | 5144 | 106 | 69 | 11006 | ENSG00000117399,ENSG00000159479,ENSG00000198198,ENSG00000178922,ENSG00000196872,ENSG00000115514,ENSG00000158417,ENSG00000128655,ENSG00000155636,ENSG00000115524,ENSG00000144381,ENSG00000115541,ENSG00000270757,ENSG00000115540,ENSG00000115896,ENSG00000168016,ENSG00000076242,ENSG00000144674,ENSG00000068745,ENSG00000178467,ENSG00000178149,ENSG00000178057,ENSG00000178035,ENSG00000198218,ENSG00000172053,ENSG00000172037,ENSG00000185909,ENSG00000114316,ENSG00000233276,ENSG00000145022,ENSG00000145020,ENSG00000183763,ENSG00000164078,ENSG00000004534,ENSG00000003756,ENSG00000114353,ENSG00000214706,ENSG00000243477,ENSG00000114395,ENSG00000126062,ENSG00000114054,ENSG00000118007,ENSG00000158092,ENSG00000158186,ENSG00000114107,ENSG00000158234,ENSG00000075711,ENSG00000158321,ENSG00000188938,ENSG00000048828,ENSG00000197724,ENSG00000086827,ENSG00000048028,ENSG00000167548,ENSG00000150967,ENSG00000111325,ENSG00000182196,ENSG00000051825,ENSG00000139697,ENSG00000150977,ENSG00000100567,ENSG00000100575,ENSG00000248905,ENSG00000140262,ENSG00000103264,ENSG00000198795,ENSG00000088930,ENSG00000101337,ENSG00000101346 |
| HPA | cerebral cortex; endothelial cells[тЙeLow] | HPA:0100201 | FALSE | 2.68E-02 | 1.571503809 | 4633 | 106 | 64 | 11006 | ENSG00000159479,ENSG00000198198,ENSG00000178922,ENSG00000115514,ENSG00000158417,ENSG00000128655,ENSG00000065413,ENSG00000115524,ENSG00000115541,ENSG00000270757,ENSG00000115540,ENSG00000115896,ENSG00000076242,ENSG00000144674,ENSG00000068745,ENSG00000178537,ENSG00000178149,ENSG00000178057,ENSG00000178035,ENSG00000198218,ENSG00000172053,ENSG00000172037,ENSG00000114316,ENSG00000233276,ENSG00000067560,ENSG00000145022,ENSG00000145020,ENSG00000173402,ENSG00000164061,ENSG00000183763,ENSG00000164078,ENSG00000004534,ENSG00000003756,ENSG00000114353,ENSG00000243477,ENSG00000126062,ENSG00000114054,ENSG00000118007,ENSG00000158092,ENSG00000158186,ENSG00000114107,ENSG00000158234,ENSG00000075711,ENSG00000048828,ENSG00000197724,ENSG00000086827,ENSG00000048028,ENSG00000150967,ENSG00000111325,ENSG00000182196,ENSG00000090975,ENSG00000139697,ENSG00000150977,ENSG00000100567,ENSG00000100575,ENSG00000137871,ENSG00000140262,ENSG00000103264,ENSG00000198795,ENSG00000088930,ENSG00000101337,ENSG00000101346,ENSG00000101350,ENSG00000197183 |
| HPA | hippocampus; glial cells[тЙeLow] | HPA:0250121 | FALSE | 2.82E-02 | 1.54945497 | 4138 | 106 | 59 | 11006 | ENSG00000066322,ENSG00000159479,ENSG00000198198,ENSG00000178922,ENSG00000196872,ENSG00000158417,ENSG00000128655,ENSG00000155636,ENSG00000115524,ENSG00000144381,ENSG00000115541,ENSG00000270757,ENSG00000115540,ENSG00000162944,ENSG00000115896,ENSG00000076242,ENSG00000144674,ENSG00000068745,ENSG00000178467,ENSG00000178057,ENSG00000178035,ENSG00000198218,ENSG00000185909,ENSG00000233276,ENSG00000067560,ENSG00000145022,ENSG00000145020,ENSG00000164076,ENSG00000164078,ENSG00000004534,ENSG00000003756,ENSG00000001617,ENSG00000114353,ENSG00000243477,ENSG00000114395,ENSG00000126062,ENSG00000118007,ENSG00000158220,ENSG00000114107,ENSG00000158321,ENSG00000048828,ENSG00000197724,ENSG00000086827,ENSG00000167548,ENSG00000150967,ENSG00000182196,ENSG00000090975,ENSG00000051825,ENSG00000111328,ENSG00000139697,ENSG00000100575,ENSG00000137871,ENSG00000140262,ENSG00000198795,ENSG00000088930,ENSG00000125820,ENSG00000101337,ENSG00000101346,ENSG00000197183 |
| HPA | thyroid gland; glandular cells[High] | HPA:0590053 | FALSE | 2.87E-02 | 1.541658353 | 1882 | 106 | 34 | 11006 | ENSG00000196872,ENSG00000158417,ENSG00000115541,ENSG00000270757,ENSG00000115540,ENSG00000076242,ENSG00000144674,ENSG00000178467,ENSG00000178057,ENSG00000178035,ENSG00000198218,ENSG00000185909,ENSG00000145020,ENSG00000164078,ENSG00000004534,ENSG00000003756,ENSG00000114353,ENSG00000114395,ENSG00000114054,ENSG00000118007,ENSG00000158092,ENSG00000158186,ENSG00000075711,ENSG00000048828,ENSG00000197724,ENSG00000086827,ENSG00000048028,ENSG00000182196,ENSG00000139697,ENSG00000137871,ENSG00000140262,ENSG00000103264,ENSG00000088930,ENSG00000101346 |
| HPA | heart muscle; cardiomyocytes[тЙeMedium] | HPA:0241102 | FALSE | 3.56E-02 | 1.44879575 | 4367 | 106 | 61 | 11006 | ENSG00000066322,ENSG00000159479,ENSG00000198198,ENSG00000178922,ENSG00000196872,ENSG00000158417,ENSG00000128655,ENSG00000155636,ENSG00000115524,ENSG00000115541,ENSG00000270757,ENSG00000115540,ENSG00000115896,ENSG00000076242,ENSG00000144674,ENSG00000068745,ENSG00000178537,ENSG00000178467,ENSG00000178252,ENSG00000178149,ENSG00000178057,ENSG00000198218,ENSG00000172053,ENSG00000172037,ENSG00000185909,ENSG00000114316,ENSG00000145022,ENSG00000145020,ENSG00000173402,ENSG00000183763,ENSG00000164078,ENSG00000004534,ENSG00000003756,ENSG00000214706,ENSG00000243477,ENSG00000114395,ENSG00000114054,ENSG00000118007,ENSG00000158186,ENSG00000114107,ENSG00000158234,ENSG00000158321,ENSG00000188938,ENSG00000048828,ENSG00000197724,ENSG00000086827,ENSG00000048028,ENSG00000149305,ENSG00000150967,ENSG00000182196,ENSG00000111328,ENSG00000139697,ENSG00000100567,ENSG00000100575,ENSG00000248905,ENSG00000140262,ENSG00000103264,ENSG00000198795,ENSG00000088930,ENSG00000101337,ENSG00000101350 |
| HPA | stomach 2; glandular cells[High] | HPA:0550053 | FALSE | 4.29E-02 | 1.367255865 | 2427 | 106 | 40 | 11006 | ENSG00000117399,ENSG00000178922,ENSG00000196872,ENSG00000158417,ENSG00000128655,ENSG00000155636,ENSG00000115520,ENSG00000115541,ENSG00000270757,ENSG00000115540,ENSG00000115896,ENSG00000076242,ENSG00000144674,ENSG00000068745,ENSG00000178467,ENSG00000178149,ENSG00000178057,ENSG00000178035,ENSG00000172053,ENSG00000114316,ENSG00000145022,ENSG00000145020,ENSG00000004534,ENSG00000003756,ENSG00000243477,ENSG00000114395,ENSG00000114054,ENSG00000158186,ENSG00000114107,ENSG00000158234,ENSG00000075711,ENSG00000048828,ENSG00000197724,ENSG00000086827,ENSG00000048028,ENSG00000182196,ENSG00000139697,ENSG00000140262,ENSG00000103264,ENSG00000197183 |
| HPA | pancreas; pancreatic endocrine cells[тЙeMedium] | HPA:0351182 | FALSE | 4.58E-02 | 1.339443621 | 3618 | 106 | 53 | 11006 | ENSG00000159479,ENSG00000178922,ENSG00000196872,ENSG00000115514,ENSG00000158417,ENSG00000128655,ENSG00000155636,ENSG00000115524,ENSG00000115520,ENSG00000115541,ENSG00000270757,ENSG00000115540,ENSG00000115896,ENSG00000076242,ENSG00000144674,ENSG00000068745,ENSG00000178467,ENSG00000178149,ENSG00000178057,ENSG00000178035,ENSG00000198218,ENSG00000233276,ENSG00000067560,ENSG00000145020,ENSG00000004534,ENSG00000003756,ENSG00000001617,ENSG00000114353,ENSG00000243477,ENSG00000126062,ENSG00000118007,ENSG00000168917,ENSG00000158092,ENSG00000114107,ENSG00000158234,ENSG00000075711,ENSG00000188938,ENSG00000048828,ENSG00000197724,ENSG00000086827,ENSG00000048028,ENSG00000167548,ENSG00000182196,ENSG00000090975,ENSG00000051825,ENSG00000139697,ENSG00000100575,ENSG00000137871,ENSG00000088930,ENSG00000101337,ENSG00000101346,ENSG00000101350,ENSG00000197183 |
| HPA | heart muscle | HPA:0240000 | FALSE | 0.045877462 | 1.338400612 | 6323 | 106 | 79 | 11006 | ENSG00000066322,ENSG00000159479,ENSG00000198198,ENSG00000178922,ENSG00000196872,ENSG00000115514,ENSG00000158417,ENSG00000128655,ENSG00000155636,ENSG00000065413,ENSG00000115524,ENSG00000115520,ENSG00000144381,ENSG00000115541,ENSG00000270757,ENSG00000115540,ENSG00000162944,ENSG00000115896,ENSG00000076242,ENSG00000144674,ENSG00000068745,ENSG00000178537,ENSG00000178467,ENSG00000178252,ENSG00000178149,ENSG00000178057,ENSG00000178035,ENSG00000198218,ENSG00000172053,ENSG00000172037,ENSG00000185909,ENSG00000114316,ENSG00000233276,ENSG00000067560,ENSG00000145022,ENSG00000145020,ENSG00000173402,ENSG00000164061,ENSG00000183763,ENSG00000164078,ENSG00000004534,ENSG00000003756,ENSG00000114353,ENSG00000214706,ENSG00000243477,ENSG00000114395,ENSG00000126062,ENSG00000114054,ENSG00000118007,ENSG00000168917,ENSG00000158092,ENSG00000158186,ENSG00000114107,ENSG00000158234,ENSG00000075711,ENSG00000158321,ENSG00000188938,ENSG00000048828,ENSG00000197724,ENSG00000086827,ENSG00000048028,ENSG00000149305,ENSG00000150967,ENSG00000182196,ENSG00000051825,ENSG00000111328,ENSG00000139697,ENSG00000150977,ENSG00000100567,ENSG00000100575,ENSG00000248905,ENSG00000137871,ENSG00000140262,ENSG00000103264,ENSG00000198795,ENSG00000088930,ENSG00000101337,ENSG00000101346,ENSG00000101350 |
| HPA | heart muscle; cardiomyocytes[тЙeLow] | HPA:0241101 | FALSE | 0.045877462 | 1.338400612 | 6323 | 106 | 79 | 11006 | ENSG00000066322,ENSG00000159479,ENSG00000198198,ENSG00000178922,ENSG00000196872,ENSG00000115514,ENSG00000158417,ENSG00000128655,ENSG00000155636,ENSG00000065413,ENSG00000115524,ENSG00000115520,ENSG00000144381,ENSG00000115541,ENSG00000270757,ENSG00000115540,ENSG00000162944,ENSG00000115896,ENSG00000076242,ENSG00000144674,ENSG00000068745,ENSG00000178537,ENSG00000178467,ENSG00000178252,ENSG00000178149,ENSG00000178057,ENSG00000178035,ENSG00000198218,ENSG00000172053,ENSG00000172037,ENSG00000185909,ENSG00000114316,ENSG00000233276,ENSG00000067560,ENSG00000145022,ENSG00000145020,ENSG00000173402,ENSG00000164061,ENSG00000183763,ENSG00000164078,ENSG00000004534,ENSG00000003756,ENSG00000114353,ENSG00000214706,ENSG00000243477,ENSG00000114395,ENSG00000126062,ENSG00000114054,ENSG00000118007,ENSG00000168917,ENSG00000158092,ENSG00000158186,ENSG00000114107,ENSG00000158234,ENSG00000075711,ENSG00000158321,ENSG00000188938,ENSG00000048828,ENSG00000197724,ENSG00000086827,ENSG00000048028,ENSG00000149305,ENSG00000150967,ENSG00000182196,ENSG00000051825,ENSG00000111328,ENSG00000139697,ENSG00000150977,ENSG00000100567,ENSG00000100575,ENSG00000248905,ENSG00000137871,ENSG00000140262,ENSG00000103264,ENSG00000198795,ENSG00000088930,ENSG00000101337,ENSG00000101346,ENSG00000101350 |
